# DJ-1 promotes colorectal cancer progression through activating PLAGL2/Wnt/BMP4 axis

**DOI:** 10.1038/s41419-018-0883-4

**Published:** 2018-08-29

**Authors:** Jing Zhou, Hao Liu, Lian Zhang, Xin Liu, Chundong Zhang, Yitao Wang, Qing He, Ying Zhang, Yi Li, Quanmei Chen, Lu Zhang, Kui Wang, Youquan Bu, Yunlong Lei

**Affiliations:** 1 Department of Biochemistry and Molecular Biology, and Molecular Medicine and Cancer Research Center, Chongqing Medical University, 400016 Chongqing, China; 2 State Key Laboratory of Biotherapy and Cancer Center, West China Hospital, West China School of Basic Medical Sciences & Forensic Medicine, Sichuan University and Collaborative Innovation Center for Biotherapy, 610041 Chengdu, China

## Abstract

Metastasis remains a big barrier for the clinical treatment of colorectal cancer (CRC). Our previous proteomics analysis identified DJ-1 as a potential metastasis biomarker of CRC. In this study, we found that DJ-1 was upregulated in CRC. The levels of DJ-1 were closely correlated with the depths of invasion and predicted patient outcome. Enforced expression of DJ-1 could enhance CRC proliferation and metastasis in vitro and in vivo by stimulating Wnt-β-catenin signaling. Specifically, DJ-1-induced β-catenin nuclear translocation stimulated TCF transcription activity, which promoted BMP4 expression for CRC cell migration and invasion, and elevated CCND1 expression for CRC cell proliferation, respectively. Furthermore, DJ-1-induced Wnt signaling activation was dependent on PLAGL2 expression. In conclusion, our study demonstrates that DJ-1 can promote CRC metastasis by activating PLAGL2–Wnt–BMP4 axis, suggesting novel therapeutic opportunities for postoperative adjuvant therapy in CRC patients.

## Introduction

Colorectal cancer (CRC) is the third most commonly diagnosed type of cancer and the fourth leading cause of cancer death worldwide^1^. A large proportion of CRC patients are diagnosed at advanced stage, the 5-year survival rate of which is less than 10%^1^. Although lots of achievements have been obtained to uncover the mystery of CRC metastasis in the past decades, unfortunately options for the clinical treatment of patients with metastatic CRC are still rare currently. Thus, unraveling more detailed and special molecular mechanisms underlying CRC metastasis is required.

DJ-1 (PARK7/CAP1/RS) is a multifunctional protein which protects neurons from oxidative stress, and is largely linked to Parkinson’s disease^2,3^. DJ-1 is first cloned as an oncogene capable of transforming NIH-3T3 cells alone or cooperation with other oncogenes, such as H-Ras and c-Myc^2^. DJ-1 has been demonstrated to be overexpressed in many types of tumor, including uveal melanoma, non-small cell lung carcinoma (NSCLC), hepatocellular carcinoma, pancreatic ductal adenocarcinoma (PDAC), ovarian carcinoma, breast cancer, and esophageal squamous cell carcinoma (ESCC)^3^. High DJ-1 levels are significantly correlated with metastasis or worsen prognosis in some cancers, such as endometrial cancer, NSCLC, pancreatic cancer, ESCC, and cervical cancer^2^. Accumulating evidence has shown that DJ-1 can promote cancer cell survival, proliferation, and metastasis by multiple mechanisms, such as regulating redox balance, activating Akt/mTOR, MEK/ERK, NF-κB, and HIFα signaling pathways, or repressing p53, JNK, and ASK1 signaling pathways^2^. In addition, a high concentration of DJ-1 can be detected in body fluids such as serum, pancreatic juice, and nipple fluid in patients with breast cancer, PDAC, melanoma, and Parkinson’s disease, suggesting that DJ-1 can act as a non-invasive biomarker for cancer diagnosis and prognosis^2,4,5^. However, the role of DJ-1 in CRC progression remains unclear. In the previous study, our proteomics analysis results showed that the expression of DJ-1 was significantly increased in the highly metastatic cell line (SW620) compared with the weakly metastatic CRC cell line (SW480)^6^, suggesting that DJ-1 may play a role in CRC progression.

Aberrant activation of the Wnt signaling pathway has been observed in most of CRC patients, evidenced by mutations in Adenomatous polyposis coli (APC) or β-catenin^7–9^. Activation of Wnt signaling leads to nuclear translocation of β-catenin, which then interacts with the TCF/LEF family transcription factors to stimulate the expression of target genes such as c-Myc and CCND1, ultimately contributing to CRC initiation and progression^7–9^. Moreover, recent studies have shown that Wnt activators or repressors could regulate CRC metastasis by manipulating the activity of Wnt signaling^10–13^.

Pleomorphic adenoma gene like-2 (PLAGL2) belongs to the PLAG gene family, which are C2H2 zinc-finger transcriptional factors and normally locate in the nucleus^14,15^. The role of PLAGL2 in cancer cell is paradoxical. It can either induce apoptosis in human promonocytic U937 cells and Neuro2a cells^14,15^, or promote progression of various cancers such as acute myeloid leukemia, lung adenocarcinoma, prostate, breast, gastric, and CRC^14–17^. It has been reported that PLAGL2 could impede the differentiation of neural stem cells and glioma-initiating cells by activating Wnt signaling and thus contribute to tumor progression^16^. However, it remains poorly understood about the precise molecular mechanisms of PLAGL2-drived tumorigenesis and metastasis.

In this study, we show that DJ-1 is overexpressed in advanced CRC and promotes the growth and metastasis of CRC cells by increasing PLAGL2 expression. The enhanced expression of PLAGL2 activates Wnt signaling to induce BMP4 expression for CRC cell migration, and elicit CCND1 expression for CRC cell proliferation, respectively. These results suggest that DJ-1 is a potential therapeutic target in CRC.

## Results

### DJ-1 is overexpressed in human CRC and positively correlated with tumor progression

**Fig. 1:**
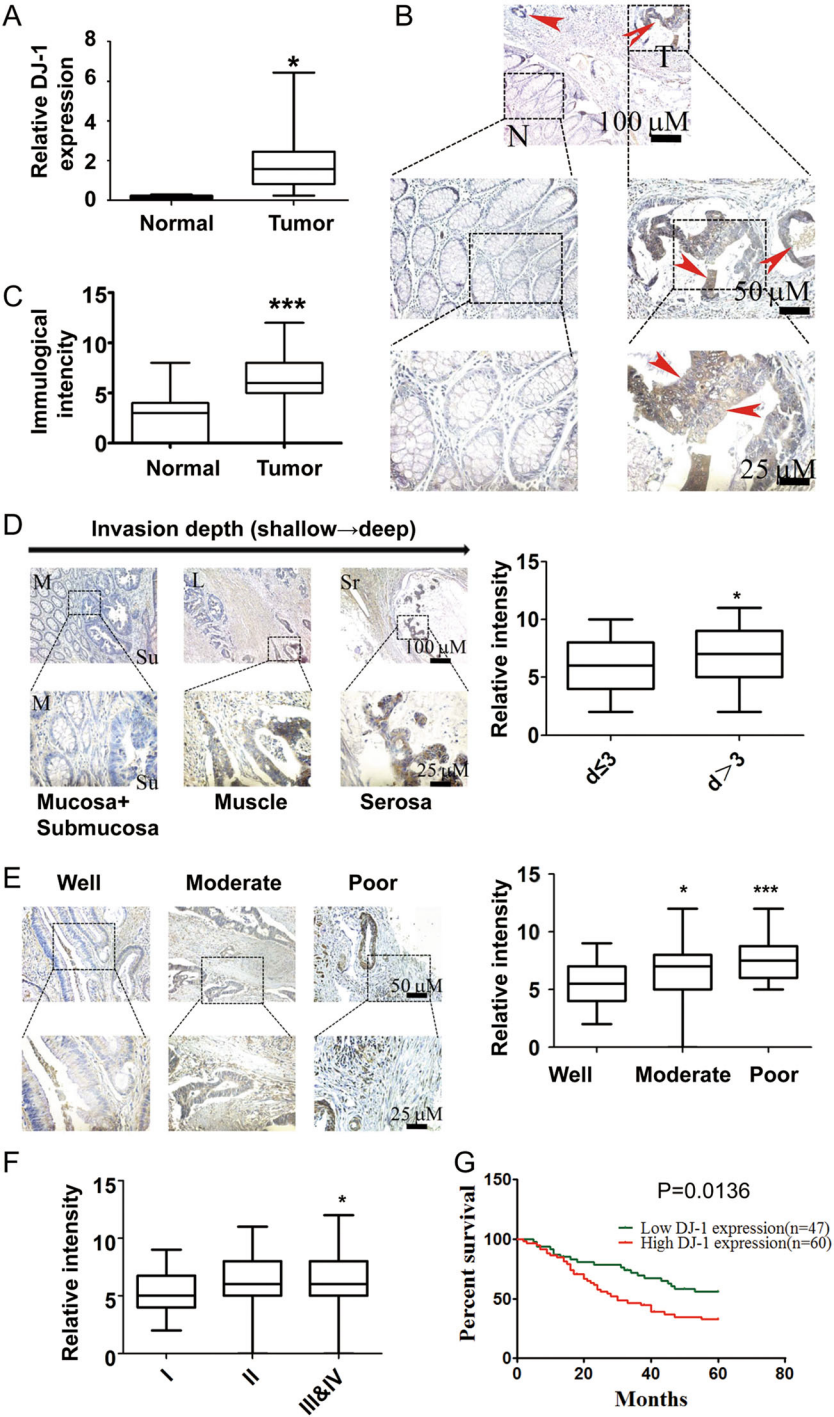
DJ-1 is upregulated in human colorectal cancers and associated with CRC progression. **a** qRT**-**PCR was conducted on CRC samples in the Tissue Scan Colon Cancer cDNA Array V to determine DJ-1 mRNA expression. **b** Immunohistochemical staining of DJ-1 in paraffin-embedded human CRC tissues. **c** Immunohistochemical scores for DJ-1 in normal colorectal mucosa and CRC tissues. **d** Immunohistochemical staining of DJ-1 in mucosa plus submucosa, muscle, and serosa tissues, which exhibit different invasion depth (left). The relative intensity of DJ-1 in different tumor size (≤3 or >3 cm) (right). **e** Immunohistochemical staining of DJ-1 in well, moderately, and poorly differentiated CRC tissues. **f** Immunohistochemical staining of DJ-1 in different stage (I, II, III/IV) CRC tissues. **g** Kaplan–Meier survival curves of CRC patients with low (*n* = 47) and high (*n* = 60) DJ-1 expression. *P < 0.05, ***P < 0.001

To investigate the expression pattern of DJ-1 in human CRC, DJ-1 mRNA expression was first identified by Gene Expression Profiling Interactive Analysis (GEPIA, http://gepia.cancer-pku.cn/)^18^. The mRNA levels of DJ-1 were higher in CRC samples than that in normal mucosal tissues (Supplementary Fig. 1A). Consistently, elevated DJ-1 mRNA was also observed in CRC tissues using qRT-PCR in an OriGene Colon Cancer cDNA Array (Fig. 1a, *P* = 0.016). Furthermore, the protein levels of DJ-1 in paired non-tumor and tumor tissues (*n* = 3) from frozen tissue samples were analyzed by Western blot analysis. Elevated DJ-1 protein expression was also found in CRC tissues compared with adjacent non-tumor tissues (Supplementary Fig. 1B). Immunohistochemistry staining was then performed on 181 CRC specimens and 117 matched adjacent normal colorectal mucosa specimens to evaluate the potential clinical relevance of DJ-1 in CRC (Supplementary Table 1). As shown in Fig. 1b and c, DJ-1 immunoreactivity was more intense in tumors than that in adjacent normal mucosal tissues (*P* < 0.001). These results demonstrated that both DJ-1 mRNA and protein levels were enhanced in human CRC.

Next, we analyzed the correlation between DJ-1 expression in tumor tissues and the clinic-pathological parameters. The results showed that tumor cells in the regions of the muscularis propria and subserosa had more intense DJ-1 staining than those in mucosal regions (Fig. 1d). In line with this, DJ-1 expression was positively correlated with tumor size (Fig. 1d, *P* = 0.0145). In addition, DJ-1 expression was reversely associated with histodifferentiation degree (Fig. 1e, *P* = 0.0016), and was elevated in stage II (*P* = 0.072) and late-stage (III/IV) (*P* = 0.026) CRC compared with that in stage I (Fig. 1f). Finally, CRC specimens from 107 patients who had survival information were stratified into two groups with 67 high-DJ-1-expressing and 40 low-DJ-1-expressing tumors, respectively. Kaplan–Meier survival analysis indicated that high levels of DJ-1 were inversely correlated with survival time of the patients (Fig. 1g). These results suggested that DJ-1 expression contributed to CRC progression and was associated with patients outcome.

### DJ-1 promotes proliferation and invasion of CRC cells in vitro

**Fig. 2:**
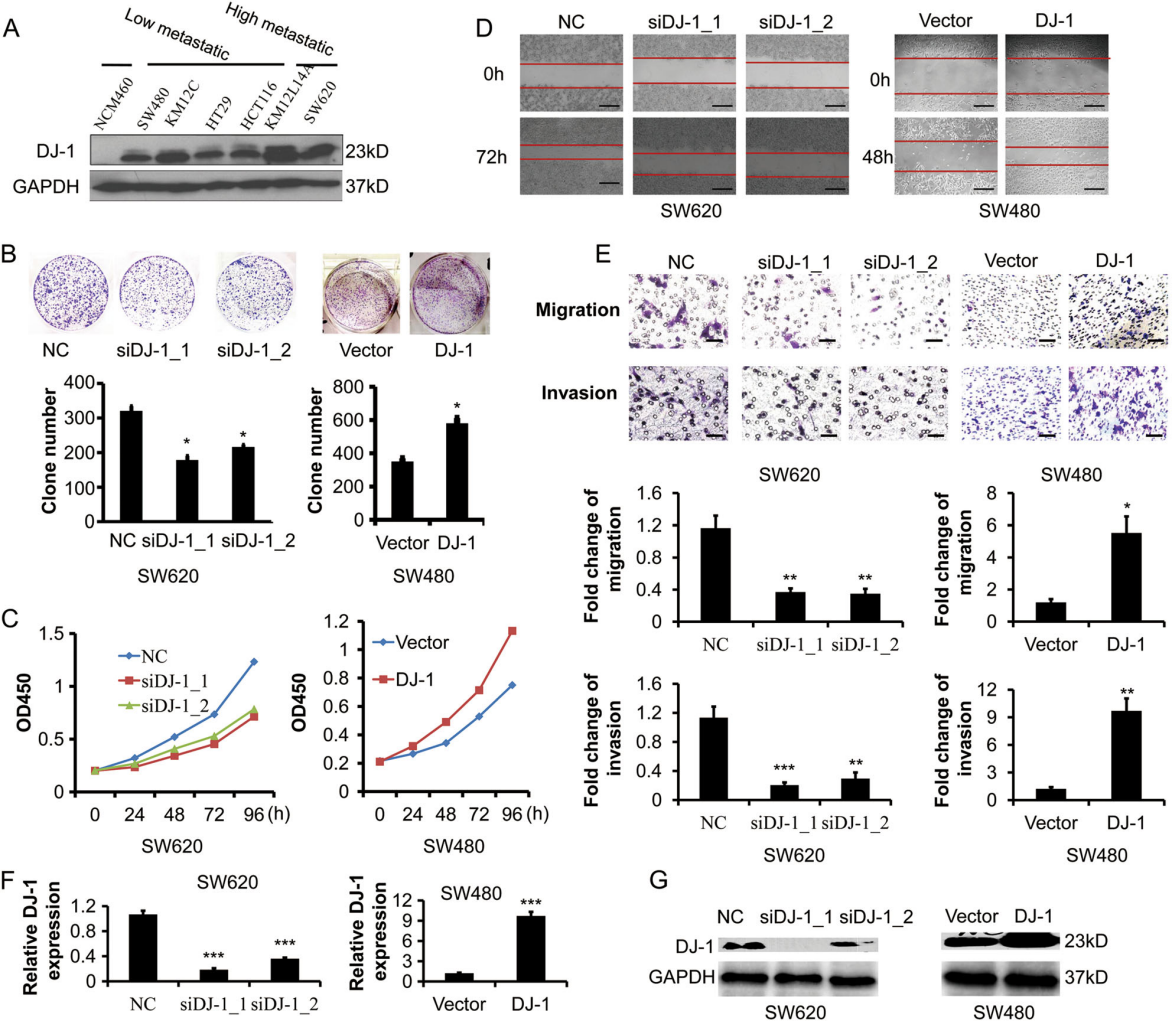
DJ-1 promotes proliferation and invasion of CRC cells. **a** Western blot analysis of DJ-1 from normal colon cell line (NCM460) and six CRC cell lines. **b**–**g** DJ-1 was knocked down by two specific siRNAs in SW620 cells, or stably overexpressed in SW480 cells. **b** Colony formation assay after 14 days culture, with mean colony counts from three independent experiments. **c** Cell proliferation assay was performed by CCK8 kit. **d** Wound-healing migration assay of indicated time. Scale bar, 500 μm. **e** Quantitative analysis of cell migration and matrigel invasion assays. Migration was analyzed at 24 h, invasion at 48 h. Scale bar, 100 μm. **f** Expression of DJ-1 mRNA was examined by qRT-PCR. **g** Expression of DJ-1 protein was examined by Western blot. *P < 0.05, **P < 0.01, ***P < 0.001

To determine the role of DJ-1 in CRC progression, we detected the DJ-1 expression in a series of CRC cell lines. As shown in Fig. 2a, DJ-1 levels in CRC cell lines, including SW480, KM12C, HT29, HCT116, KM12L4A, and SW620, were higher than that in NCM460, a human normal colorectal epithelial cell line. Further, increased DJ-1 protein levels were observed in highly metastatic CRC cell lines (SW620 and KM12L4A) compared with poorly metastatic cell lines (SW480, KM12C, HT29, and HCT116). Based on these observations, two specific siRNAs were introduced to repress the expression of DJ-1 in SW620 cells, while its paired primary CRC cell line SW480 was transfected with DJ-1 cDNA, then the proliferative, migratory, and invasive capacities were evaluated (Fig. 2b–g). As shown in Fig. 2b and c, both DJ-1 siRNAs significantly decreased colonies in the colony formation assay and inhibited proliferation of SW620 cells as examined by CCK8 regent kit, respectively. In the wound-healing migration assay, transwell migration assay, and matrigel invasion assay, the reduced wound closure rate, and migratory and invasive property were observed in SW620 cells treated with DJ-1 siRNAs (Fig. 2d, e). Conversely, enforced expression of DJ-1 significantly enhanced the proliferative, migratory, and invasive capacities of SW480 cells (Fig. 2b–g).

To rule out that DJ-1-induced CRC cell proliferation and invasion were dependent on genetic background (SW480 and SW620 are derived from the same patient), we stably transfected either DJ-1 shRNA or cDNA into HCT116 cells which have a moderate endogenous DJ-1 expression. Similarly, DJ-1 overexpression led to a rise in the proliferative and metastatic potential of HCT116 cells, whereas these biological effects were inhibited by DJ-1 knockdown (Supplementary Figs. 2, 3). These data indicated that DJ-1 could promote proliferation, migration, and invasion of CRC cells in vitro.

### DJ-1 enhances CRC tumor growth and metastasis in vivo

**Fig. 3:**
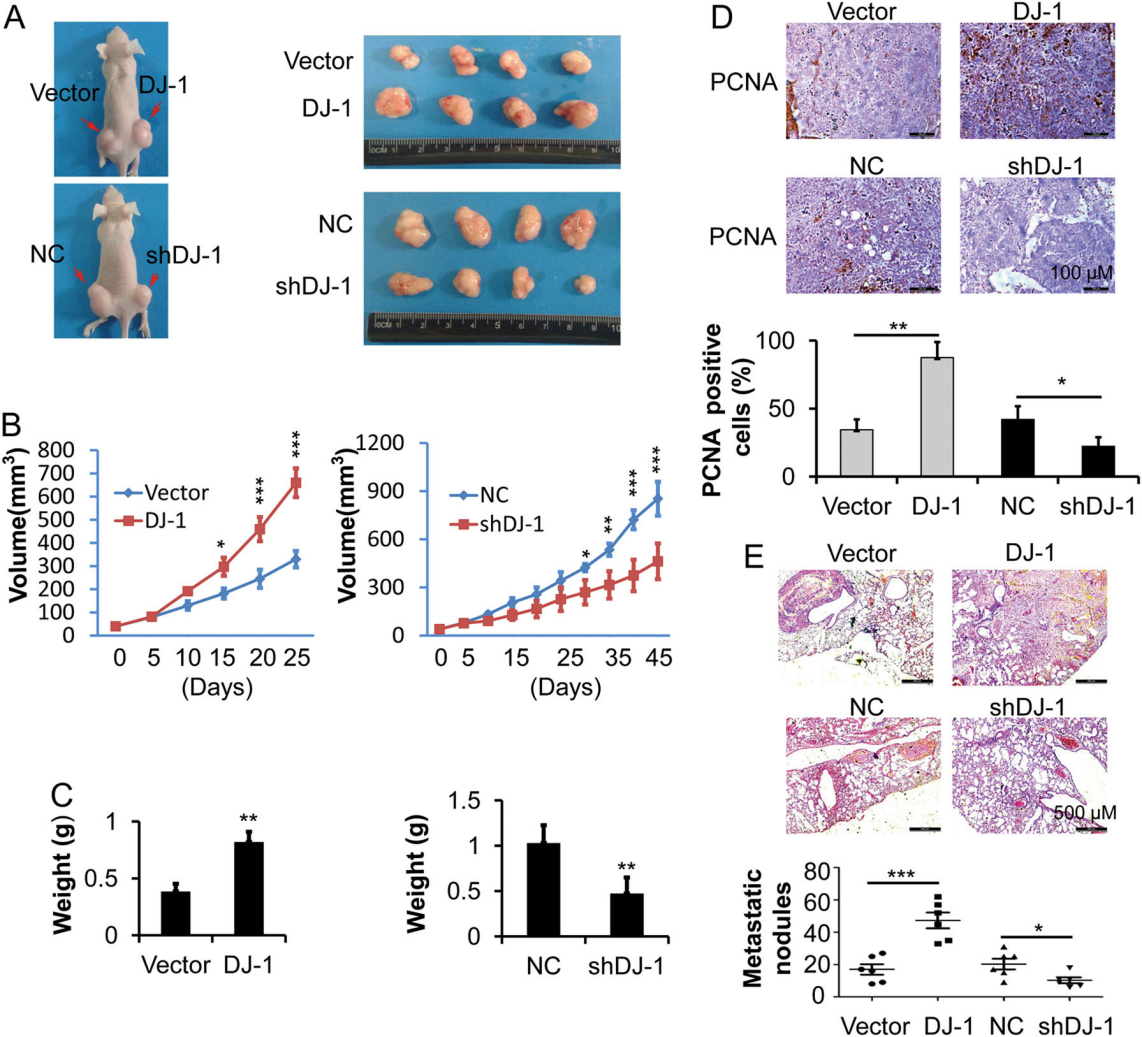
DJ-1 enhances CRC tumor growth and metastasis in vivo. HCT116 cells were stably transfected with DJ-1 shRNA or negative control vector (NC), or stably transfected with DJ-1 cDNA or vector. **a**–**c** Transfected HCT116 cells were subcutaneously injected into nude mice and xenograft tumor growth was determined for 25 days or 45 days. Tumor mass images (**a**). Growth curves of tumor volumes (**b**). Tumor weight (**c**). *n* = 4. **d** Immunohistochemical staining of PCNA in xenografts from **a**. PCNA-positive cells were calculated. Scale bar, 100 μm. **e** Histopathology showing the lung metastases in mice and quantification of number of metastases following tail-vein injection. *n* = 6. *P < 0.05, **P < 0.01, ***P < 0.001

To examine whether DJ-1 could promote CRC growth and metastasis in vivo, HCT116 cells were injected subcutaneously or intravenously into nude mice. In the xenograft tumor model, DJ-1 overexpression enhanced the tumor growth over the course of the 25-day experiment (*n* = 4), while DJ-1 knockdown repressed the tumor growth over the course of the 45-day experiment (*n* = 4), as indicated by tumor volume and weight (Fig. 3a–c). Additionally, immunohistochemistry analysis showed increased PCNA staining in DJ-1-overexpressing HCT116 cells, while reduced PCNA staining was observed in DJ-1-knockdown cells (Fig. 3d). In the lung metastasis model, a higher number of metastatic lesions derived from HCT116-DJ-1 cells was observed compared with the HCT116-Vector group (*n* = 6, Fig. 3e). Conversely, knockdown of DJ-1 markedly decreased the lung metastatic nodules in the tumor-bearing mice (*n* = 6, Fig. 3e). These results suggested that DJ-1 could regulate CRC cell growth and metastasis in vivo.

### Wnt signaling activation is essential for DJ-1-induced CRC malignant progression

**Fig. 4:**
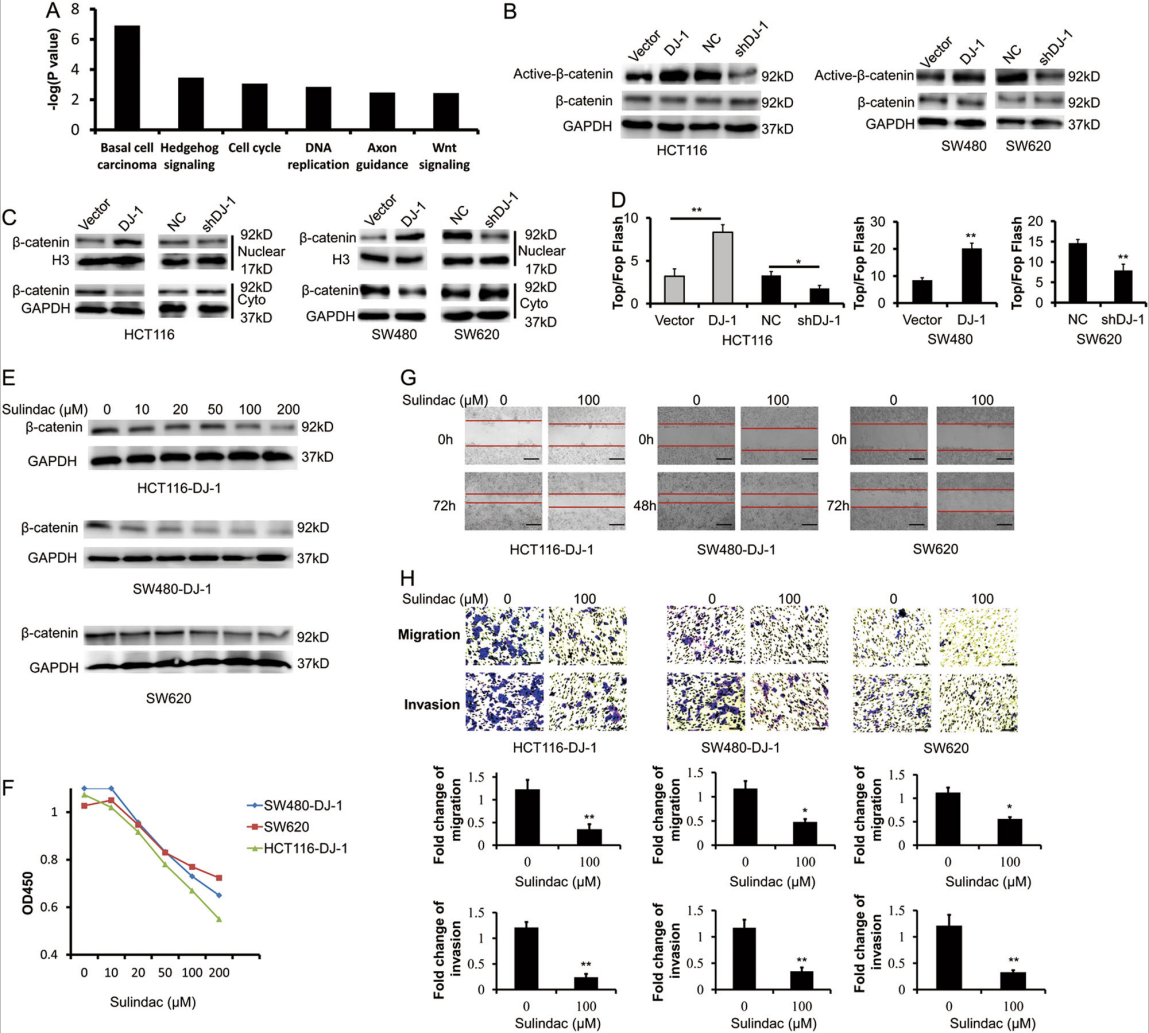
Wnt signaling activation is essential for DJ-1-induced CRC malignant progression. **a** Functional pathway analysis of the upregulated genes by DJ-1 was conducted using the web-based tool DAVID. Statistically significant modulation (indicated by the inverse log of *P*-values) of the top six over-represented canonical pathways following DJ-1 overexpression was depicted based on KEGG pathway database. **b** Western blot analysis of β-catenin and activated-β-catenin (non-phosphorylated β-catenin) expression in HCT116, SW480, and SW620 cells, in which DJ-1 was overexpressed or downregulated. **c** β-catenin expression in cytoplasm and nuclear fractions was detected by Western blot analysis. **d** TOP-Flash/FOP-Flash assay depicting Wnt activity in indicated CRC cells. **e**–**h** SW620, DJ-1-overexpressed HCT116 (HCT116-DJ-1) and SW480 (SW480-DJ-1) cells were treated with indicated concentrations of Wnt inhibitor Sulindac for 36 h. Then, β-catenin was detected by Western blot (**e**); cell proliferation assay was performed by CCK8 kit (**f**); migration and invasion activity were assessed by wound-healing migration assay (**g**, scale bar, 500 μm), transwell migration and matrigel invasion assays (**h**, scale bar, 100 μm). Migration was analyzed at 24 h, invasion at 48 h. *P < 0.05, **P < 0.01

To elucidate the molecular mechanisms underlying DJ-1-induced CRC growth and metastasis, we sought to compare the transcriptome between DJ-1-overexpressing and vector HCT116 cells. As shown in Supplementary Table 2, thousands of differentially expressed genes were defined based on alterations of >2.0-fold. Furthermore, functional analysis of the genes using web-based tool DAVID (https://david.ncifcrf.gov/summary.jsp) showed that basal carcinoma, Hedgehog, cell cycle, DNA replication, Axon guidance, and Wnt signaling were typically activated by DJ-1 overexpression (Fig. 4a, Supplementary Table 3). In fact, basal carcinoma signaling is mainly involved in activation of Hedgehog and Wnt signaling, which have been widely considered to play important roles in tumor progression and metastasis (Supplementary Fig. 4A). Thus, we had a particular interest to test whether DJ-1 could activate Hedgehog and Wnt signaling in CRC cells. As expected, DJ-1 overexpression induced the accumulation of Hedgehog signaling molecules (including GLI1, GLI2, and PTCH1) and the target genes of Wnt signaling (such as CCND1, TCF7, FGF9, and AXIN2) in HCT116 and SW480 cells (Supplementary Fig. 4B–F). In contrast, silencing DJ-1 in HCT116 and SW620 cells inhibited the expression of these genes (Supplementary Fig. 4B–F). These data suggested that DJ-1 could regulate both Hedgehog and Wnt signaling pathways. In CRC, Hedgehog and Wnt signaling can undergo either mutual promotion or reciprocal inhibition, and the balance between these two pathways plays an important role in the development of metastases^19^. However, in most of CRC cell lines, the Hedgehog pathway members are incomplete^20,21^, implying that the activation of Hedgehog pathway in CRC cell lines may depend on other factors such as activation of Wnt signaling. Therefore, we mainly focused on the Wnt signaling in DJ-1-induced CRC growth and metastasis.

To this end, we further investigated whether Wnt signaling was the downstream cascade of DJ-1. As expected, the expression of non-phosphorylated (active) β-catenin, nuclear accumulation of β-catenin, and TCF/LEF transcription activity were enhanced in DJ-1-overexpressing HCT116 and SW480 cells, but inhibited in DJ-1-knockdown HCT116 and SW620 cells, indicating that DJ-1 could positively regulate Wnt signaling (Fig. 4b–d). Next, we used sulindac, an agent that degrades β-catenin, to inactive Wnt signaling in HCT116-DJ-1, SW480-DJ-1, and SW620 cells (Fig. 4e). As shown in Fig. 4f–h, sulindac treatment markedly abrogated DJ-1-induced proliferation, migration, and invasion of CRC cells, suggesting that Wnt signaling was essential for DJ-1-induced aggressive phenotype in CRC.

### BMP4 is required for DJ-1-induced and Wnt signaling-mediated CRC migration and invasion

To investigate the detailed basis of Wnt signaling in DJ-1-induced CRC malignant progression, we analyzed the expression of Wnt signaling target genes in our RNA-Seq data^22^. The results showed that the expression levels of a total of 27 Wnt signaling target genes were significantly increased after DJ-1 overexpression in HCT116 cells (Supplementary Table 4). Of them, bone morphogenetic protein-4 (BMP4) was one of the most significantly altered Wnt signaling target genes. BMP4 belongs to the TGF-β superfamily and has been reported to be involved in CRC progression^23^. The web-based tool GEPIA analysis showed that BMP4 mRNA in CRC was significantly higher than that in normal tissues, and BMP4 expression was negatively correlated with disease-free survival (Supplementary Fig. 5A, B). Moreover, BMP4 expression was positively correlated with other DJ-1-regulated Wnt target genes, including TCF7, CCND1, and AXIN2 (Supplementary Fig. 5C).

**Fig. 5:**
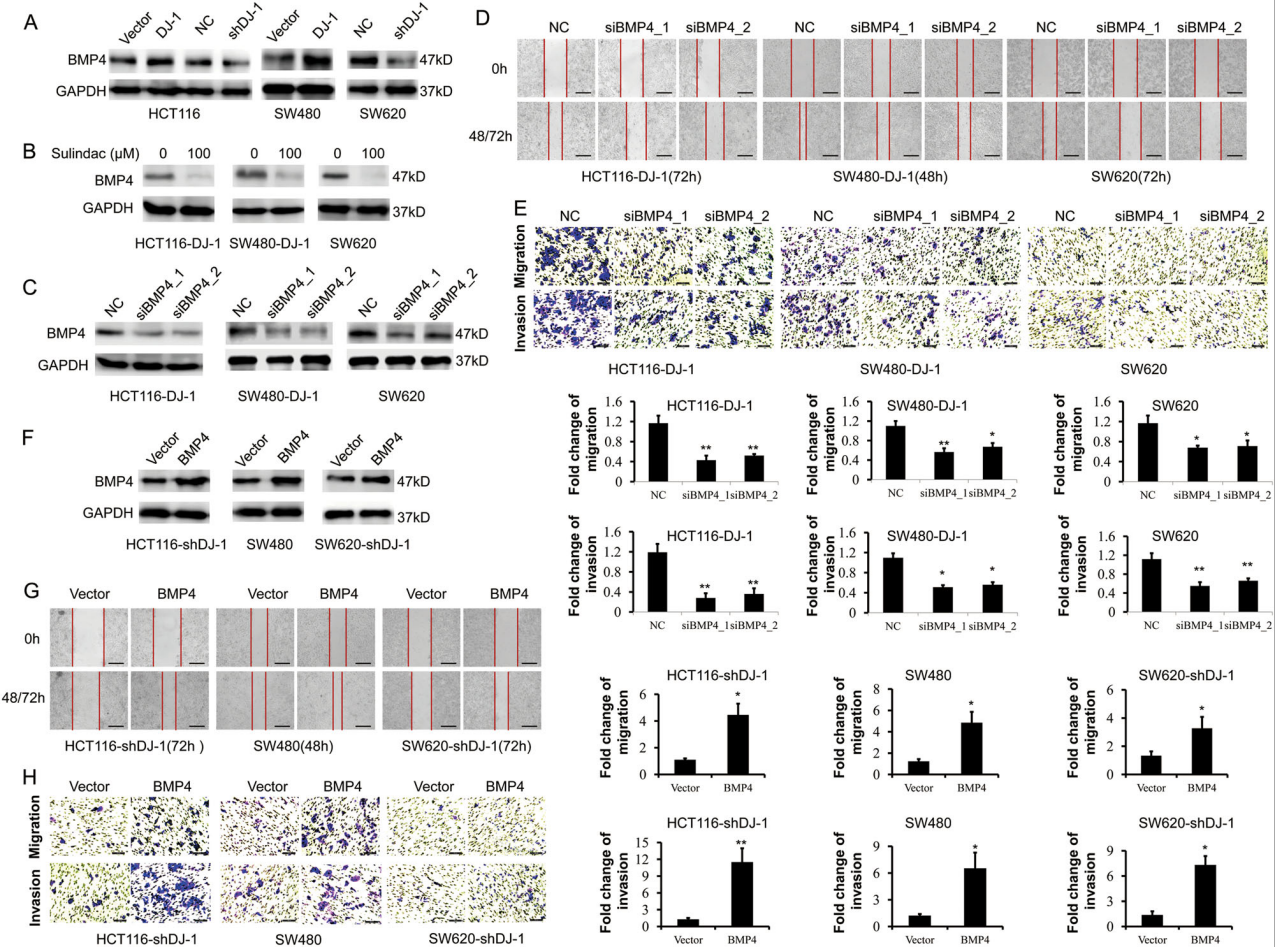
BMP4 is required for DJ-1-induced and Wnt signaling-mediated CRC migration and invasion. **a** Western blot analysis of BMP4 expression in HCT116, SW480, and SW620 cells stable transfected with DJ-1 cDNA, DJ-1 shRNA or vector. **b** Western blot analysis of BMP4 in HCT116-DJ-1, SW480-DJ-1, and SW620 cells treated with or without 100 μM Sulindac for 36 h. **c**–**e** HCT116-DJ-1, SW480-DJ-1, and SW620 cells were transfected with two specific BMP4 siRNAs, respectively. Then, protein level of BMP4 was determined by Western blot (**c**); migration and invasion activity were assessed by wound-healing migration assay (**d**, scale bar, 500 μm), transwell migration and matrigel invasion assays (**e**, scale bar, 100 μm). **f**–**h** SW480, DJ-1-konocdown HCT116 (HCT116-shDJ-1), and SW620 (SW620-shDJ-1) cells were transfected with BMP4 cDNA. Then, BMP4 was examined by Western blot (**f**); migration and invasion activity were assessed by wound-healing migration assay (**g**, scale bar, 500 μm), transwell migration and matrigel invasion assays (**h**, scale bar, 100 μm). Migration was analyzed at 24 h, invasion at 48 h. *P < 0.05, **P < 0.01

As expected, ectopic expression of DJ-1 promoted the levels of both BMP4 mRNA and protein in CRC cells, while DJ-1 silencing decreased BMP4 expression (Fig. 5a and Supplementary Fig. 6A). In addition, inhibition of Wnt signaling by sulindac abolished DJ-1-induced BMP4 expression in CRC cells (Fig. 5b and Supplementary Fig. 6B). Furthermore, knockdown of BMP4 by siRNAs markedly retarded DJ-1-induced CRC cell migration and invasion (Fig. 5c–e and Supplementary Fig. 6C), while BMP4 overexpression restored CRC cell migration and invasion inhibited by DJ-1 shRNA (Fig. 5f–h and Supplementary Fig. 6D). However, neither silencing BMP4 in DJ-1-overexpressing CRC cells nor ectopically expressing BMP4 in DJ-1-knockdown CRC cells affected the proliferative potential (Supplementary Fig. 7). These results showed that BMP4 was required for DJ-1-induced and Wnt signaling-mediated metastatic potential of CRC cells, but not involved in CRC cell proliferation.

CCND1 is another typical DJ-1-upregulated Wnt target gene in our RNA-Seq data (Supplementary Table 4), which has been widely reported to promote cancer cell proliferation by altering cell cycle progression^7^. To determine whether CCND1 was associated with DJ-1-induced and Wnt-mediated CRC cell proliferation, we interfered CCND1 expression by specific siRNA in SW620 and DJ-1-overexpressing HCT116 and SW480 cells. As indicated in Supplementary Fig. 8, silencing CCND1 significantly restrained DJ-1-induced proliferation and colony formation of CRC cells, suggesting that Wnt target gene CCND1 played an important role in DJ-1-induced CRC cell proliferation.

### DJ-1 activates Wnt signaling by enhancing PLAGL2 expression

We further examined the molecular mechanisms through which DJ-1 activated Wnt signaling. Bioinformatics analysis based on our RNA-Seq data showed DJ-1 increased the expression of PLAGL2, a Wnt signaling agonist (Supplementary Table 2). Based on GEPIA analysis, PLAGL2 mRNA was significantly upregulated in CRC cells and predicted poor prognoses of CRC patients (Supplementary Fig. 9A, B). Moreover, there are positive associations between PLAGL2 and Wnt target genes including TCF7, CCND1, and AXIN2 (Supplementary Fig. 9C). These results suggested that PLAGL2 was involved in DJ-1-induced activation of Wnt signaling.

**Fig. 6:**
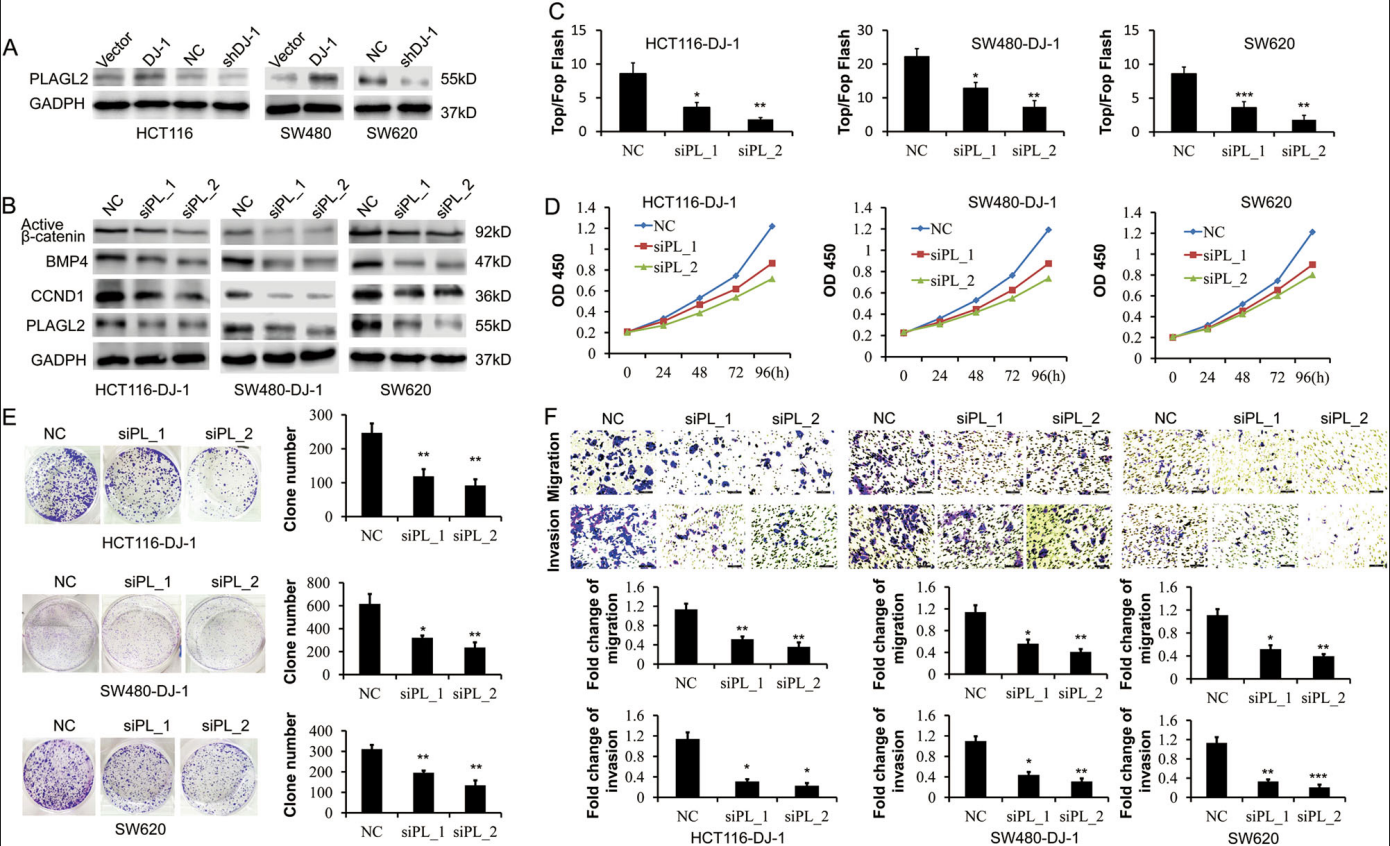
DJ-1 activates Wnt signaling by enhancing PLAGL2 expression. **a** Western blot analysis of PLAGL2 expression in HCT116, SW480, and SW620 cells stably transfected with DJ-1 cDNA, DJ-1 shRNA, or vector. **b**–**f** HCT116-DJ-1, SW480-DJ-1, and SW620 cells were transfected with two specific PLAGL2 (PL) siRNAs, respectively. Then, protein levels of activated-β-catenin, BMP4, CCND1, and PLAGL2 were determined by Western blot (**b**); Wnt activity was analyzed by TOP-Flash/FOP-Flash assay (**c**); proliferation of CRC cells was examined by CCK8 kit (**d**); proliferative activity was detected by colony formation assay (**e**); migration and invasion activity were assessed by transwell migration and matrigel invasion assays (**f**, scale bar, 100 μm). Migration was analyzed at 24 h, invasion at 48 h. *P < 0.05, **P < 0.01, ***P < 0.001

In CRC cells, both mRNA and protein levels of PLAGL2 varied with DJ-1, indicating that DJ-1 positively regulated PLAGL2 expression (Fig. 6a and Supplementary Fig. 10A). Next, PLAGL2 was knocked down in CRC cells with high DJ-1 levels to determine whether PLAGL2 was required for the DJ-1-induced Wnt activation. As shown in Fig. 6b, c and Supplementary Fig. 10B, knockdown of PLAGL2 counteracted DJ-1-induced accumulation of non-phospho (active) β-catenin, as well as upregulation of Wnt target genes BMP4 and CCND1, accompanied with reduced TCF/LEF transcription activity. Consistently, loss of PLAGL2 blocked DJ-1-ehanced proliferation, colony formation, wound closure rate, migration, and invasion (Fig. 6d–f and Supplementary Fig. 11). PLAGL2 overexpression restored proliferation, migration, invasion, and Wnt signaling activity in DJ-1-knockdown CRC cells (Supplementary Figs. 12, 13). These results demonstrated that PLAGL2 contributed to the DJ-1-induced proliferative and metastatic capacity of CRC cells by activating Wnt signaling.

**Fig. 7:**
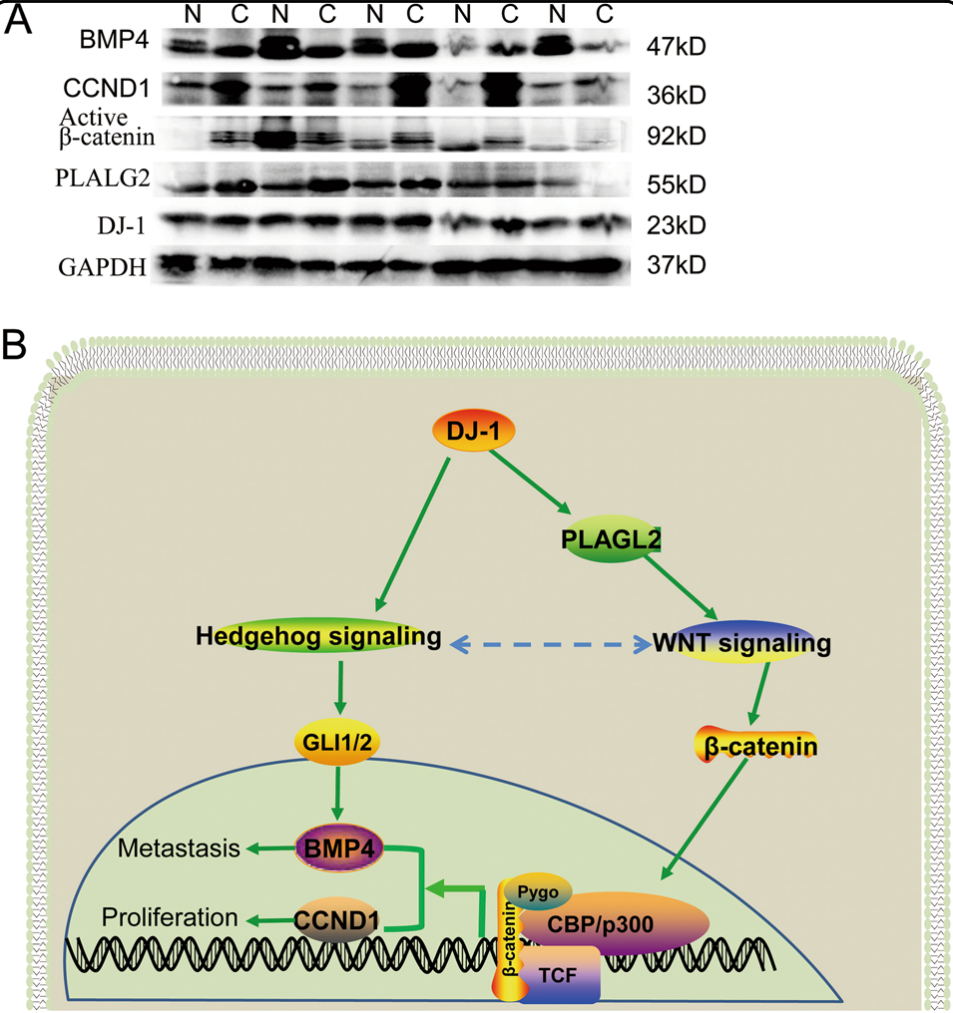
Both Wnt signaling and Hedgehog signaling are involved in DJ-1-induced CRC progression. **a** Western blots analysis of DJ-1, PLAGL2, active β-catenin, BMP4, and CCND1 expression in CRC tissues and patient-matched adjacent normal tissues. **b** Schematic illustrating the potential role of DJ-1 in CRC growth and metastasis. DJ-1 overexpression will enhance PLAGL2 expression, which then induces nuclear β-catenin accumulation and TCF transcription activity to promote BMP4 expression for CRC cell migration and invasion, and CCND1 expression for CRC cell proliferation. DJ-1 can also activate Hedgehog signaling which is involved in DJ-1-induced and Wnt signaling-mediated BMP4 expression and CRC cell proliferation.

To examine the DJ-1/PLAGL2/Wnt signaling in CRC, the protein levels of DJ-1, PLAGL2, active β-catenin, BMP4, and CCND1 in paired non-tumor and tumor tissues (*n* = 5) from frozen tissue samples were analyzed by Western blot analysis. The results showed DJ-1 was overexpressed in 60% CRC (3/5), and the expression of PLAGL2, active β-catenin, BMP4 or CCND1 was consistent with DJ-1, except PLAGL2 in one paired sample (Fig. 7a). More interestingly, in the two DJ-1 low-expressing paired CRC samples, the BMP4 expression was markedly decreased in CRC compared with non-tumor tissues (Fig. 7a). These results suggested that DJ-1/PLAGL2/Wnt signaling was involved in CRC progression.

To test whether Hedgehog signaling was involved in DJ-1-induced and Wnt signaling-mediated CRC progression, a Hedgehog signaling inhibitor, GANT61, was used to treat HCT116-DJ-1, SW480-DJ-1, and SW620 cells. As a result, GANT61 could markedly inhibit the Hedgehog signaling by downregulating transcription factors GLI1 and GLI2, and led to partial repression of the BMP4 and CCND1 expression and DJ-1-induced proliferation of CRC cells (Supplementary Fig. 14). Coincided with this, BMP4 mRNA expression was shown to be positively correlated with key Hedgehog signaling members including GLI1 and PTCH1 in CRC (Supplementary Fig. 15A). Furthermore, although the levels of key Hedgehog signaling members GLI1 and GLI2 were decreased in CRC compared with the normal tissues (Supplementary Fig. 15B), they were increased in high-grade (stage III and IV) tumors compared with that in low-grade (stage I and II) tumors, and CRC patients with high levels of GLI1 or GLI2 showed worse prognoses (Supplementary Fig. 16). These data suggested that Hedgehog signaling is involved in DJ-1-induced and Wnt signaling-mediated CRC progression.

## Discussion

Tumor metastasis remains the leading cause of treatment failure and death in CRC patients^1^, thus special attention should be paid on revealing the molecular mechanisms of CRC metastasis to favor early intervention of individuals with high risk of metastasis. DJ-1 is a potential metastasis regulator identified in our previous proteomics studies^6^. Herein, we confirmed that DJ-1 expression was upregulated in CRC and elevated expression of DJ-1 was associated with poor patient outcome. Moreover, DJ-1 could enhance CRC tumor growth and metastasis both in vitro and in vivo. These results demonstrated that DJ-1 contributed to CRC progression.

DJ-1 could contribute to cancer initiation, progression, and drug sensitivity by multiple mechanisms and exhibit a certain degree of context dependency. For example, DJ-1 can activate PKB/Akt signaling by suppressing PTEN, resulting in progression of breast, lung, ovarian, and clear cell renal cell carcinoma^24–27^. While in pancreatic cancer, DJ-1 promotes cell migration and invasion mainly via activation of ERK pathway^28^. In this study, we found that DJ-1 could activate Wnt signaling and Hedgehog signaling, which play a pivotal role in CRC initiation and progression. Wnt signaling is most relevant to the initiation of CRC. Over 80% CRC have mutations in APC, β-catenin, or axin, which will successively lead to nuclear β-catenin accumulation, TCF/LEF transcription activity augmentation, and increased target genes expression (such as CCND1 and c-Myc), finally resulting in uncontrolled proliferation^7,19^. In addition, mounting evidence has shown that further aggravated activation of Wnt/β-catenin signaling will promote CRC metastasis. For example, our and others’ studies showed that loss of PTEN, claudin-3, or PDLIM1 expression could induce CRC EMT and/or metastasis by enhancing the activation of Wnt signaling^10,29,30^, while surface expression of FGFR4, GPCR48, and FZD8 would response upon autocrine or paracrine growth factors or cytokines in tumor microenvironment to induce aggressive capacity by activating Wnt signaling^11,12,31^.

Considering the Hedgehog signaling is incomplete in the CRC cells in this study, we spent much more effort to reveal the potential function of Wnt signaling on DJ-1-enhanced CRC aggressive capacity. We identified that a series of Wnt target genes were positively regulated by DJ-1. Of them, BMP4 was one of the most significantly altered genes and BMP4 knockdown could ameliorate DJ-1-induced CRC cell migration and invasion, but not proliferation. Interestingly, silencing another classic Wnt target gene CCND1 could markedly inhibit DJ-1-induced CRC proliferation. BMP4, a member of TGF-β family and a direct target of Wnt signaling in CRC, is first identified as a tumor suppressor in the initiation of various cancers including CRC^22,32,33^. For example, Whissell *et al*. showed that the zinc-finger transcription factors GATA6 directly activated Wnt signaling and completely inhibited Wnt signaling-induced expression of BMP4, resulting in development of CRC^33^. After tumor formation, BMP4 will be upregulated to promote CRC progression and metastasis^23,34,35^. Although many studies showed that BMP4 had no effect on the proliferation of cultured CRC cells, BMP4 could promote growth of CRC cells in vivo and enhance the survival in response to various stresses such as starvation and drug treatment, including pinosylvin, 5-fluorouracil, and oxaliplatin^23,34–37^. More interestingly, BMP4 has been reported to activate Wnt signaling, suggesting a positive feedback between BMP4 and Wnt signaling^38^. In this study, we also showed that inhibition of Hedgehog signaling could impair DJ-1-induced and Wnt signaling-mediated BMP4 and CCND1 expression and CRC proliferation.

Finally, we revealed that DJ-1 activated Wnt signaling by augmenting PLAGL2 expression. The roles and mechanisms of PLAGL2 in tumor progression remain largely unknown. PLAGL2 was first identified as a zinc-finger transcriptional factor to regulate oxidative stress, iron depletion, or hypoxia-inducible gene expression^14–17,37,38^. Although PLAGL2 could induce apoptosis in some cell types, more evidence showed that PLAGL2 was upregulated in various cancers including CRC and contributed to tumor survival, progression, and metastasis^15–17,39,40^. Moreover, Zheng *et al*. reported that amplified PLAGL2 impeded differentiation of neural stem cells and contributed to gliomas by activating Wnt signaling, which was associated with upregulated expression of Wnt6 ligand, Fzd9 and Fzd2 receptors^16^. In line with this, our mRNA transcriptome data also showed that the expression of Wnt6, Fzd9 (Supplementary Table 2), and Fzd2 (upregulated about 1.6-fold, data not shown) was significantly enhanced by DJ-1 overexpression. In addition to transcription activity, PLAGL2 was found to promote tumor progression by regulating actin cytoskeletal architecture, or facilitating p53 degradation via binding and stabilizing Pirh2, an E3 ubiquitin ligase for p53^41,42^.

Taken together, this study revealed that DJ-1 induced PLAGL2 expression and then activated Wnt signaling to promote BMP4 and CCND1 expression, resulting in CRC growth and metastasis. Interestingly, Hedgehog signaling was also shown to be activated by DJ-1 and involved in regulation of Wnt signaling-targeted gene expression and CRC cell proliferation (Fig. 7b). These results suggest that DJ-1 is a potential prognostic and therapeutic target in CRC.

## Material and methods

### Clinical specimens

All colorectal carcinomas and corresponding adjacent normal tissues were obtained from Sichuan Provincial People’s Hospital (Chengdu, China). Tumor stage was determined according to the TNM classification system of the International Union against Cancer. Tumor differentiation was graded using Edmondson Steiner grading by two experienced pathologists. The clinicopathologic characteristics of 181 patients are summarized in Supplementary Table 1. Informed consent for tissue procurement was obtained from all patients or their relatives before study initiation, and Ethics approval was obtained from the Institutional Ethics Committee of Chongqing Medical University. Colon Cancer cDNA Array V were purchased from OriGene (MD, USA).

### Cells and cell culture

The SW480 and SW620 cell line were purchased from American Type Culture Collection (Rockville, MD), and HCT116 cell line was purchased from Shanghai cell bank in China. The last time of authentication was between October 2017 and December 2017 using the short tandem repeat analysis. Cells were maintained in Dulbecco’s Modified Eagle’s Medium (Gibco, USA) containing 10% fetal bovine serum (Hyclone, USA), penicillin (10^7^ U/L), and streptomycin (10 mg/L) at 37 °C in a humidified chamber containing 5% CO_2_. In addition, the extracts of KM12C, HT29, KM12L4A, and NCM460 were from our previous study^10^. As previously described, the last time of authentication for these cells was November 2015.

### In vivo tumor proliferation and metastasis

All animals were humanely treated under the guidelines of the Institutional Animal Care and Treatment Committee of Chongqing medical University. For in vivo tumor proliferation assays, CRC cells were transplanted subcutaneously into male athymic nude mice (four mice per group). The tumor volumes were evaluated as follows: tumor volume (mm^3^) = (length × width^2^)/2. Animals were sacrificed 25 or 45 days after injection. Tumors were dissected and fixed in formalin for immunostaining with PCNA. For metastasis assays, 5 × 10^6^ cells were injected into male athymic nude mice (six mice per group) through the tail vein. Animals were sacrificed on day 40. The lungs were excised and fixed in formalin for standard hematoxylin and eosin staining.

### TOP/FOP Flash assay

The TOP/FOP Flash assay was performed in accordance with the protocol provided by the manufacturer (Upstate Biotechnology). The cells were transfected with Top-flash plasmid (Upstate) plus pRL-CMV plasmid (Promega) or Fop-flash plasmid (Upstate) plus pRL-CMV plasmid. Two days later, luciferase activity was measured in cell extracts using a Dual Luciferase Kit (Promega). The transfection efficiency was normalized to the activity of pRL-CMV (Renilla luciferase).

### Data analysis and statistics

Unpaired *t*-test or Pearson’s correlation test was used to compare quantitative variables; Patients’ survival curve was plotted by the Kaplan–Meier method, and the log-rank test was used to determine the significant difference among groups; the Cox regression model was used to perform multivariate analysis. Linear regression was tested using the Spearman rank correlation. *P* < 0.05 was considered statistically significant.

## Electronic supplementary material

Supplementary Materials and Methods,Figure legends and Figures

Supplementary Table 1,3-6

Supplementary Table 2
