# Supplementary material for: DJ-1 promotes colorectal cancer progression through activating PLAGL2/Wnt/BMP4 axis
Source: Cell Death Dis. 2018 Aug 29;9(9):865. doi: 10.1038/s41419-018-0883-4 (PMC6115399; doi:10.1038/s41419-018-0883-4)
Supplement: Supplementary file 1 — Supplementary Materials and Methods,Figure legends and Figures [file 41419_2018_883_MOESM1_ESM.pdf]

Supplementary Materials and Methods

Antibodies

The following primary antibodies were used: DJ-1 (SC-55573) and CCND1 (SC-8396) were purchased from Santa Cruz Biotechnology (CA, USA). GAPDH (D16H11) (#5174), Histone H3 (D1H2) XP (#4499), β-Catenin (D10A8) (#8480) and Non-phospho (Active) β-Catenin (D13A1) (Ser33/37/Thr41; #8814) were purchased from Cell Signaling Technology (Beverly, MA, USA). BMP4 (ab39973), PCNA (ab29) and PLAGL2 (ab121239) were purchased from abcam (CA, USA).

Immunohistochemistry

Slides were stained using the Envision System horseradish peroxidase method (DakoCytomation Inc., Carpinteria, CA) according to the manufacturer's instructions. To estimate the score of each slide, at least eight individual fields were chosen, and 100 cancer cells were counted for each field. The score for each slide was measured as the cross product of the value of immunostaining intensity (A) and the value of proportion of staining-positive cells (B), as described previously[11]. Immunostaining intensity was divided into five grades: 0, negative; 1, weak; 2, moderate; 3, strong; 4, very strong. The proportion of staining-positive cells was divided into five grades: 0, <5%; 1, 6 –25%; 2, 26 –50%; 3, 51–75%; 4, >75%. The results were defined as: 0-4, low; 5-16, high. Results were assessed and confirmed by two independent experienced pathologists.

Transfection

All siRNAs were chemically synthesized by GenePharma company (Shanghai, China). The sequences of the siRNAs used are listed in Supplementary Table 5. The siRNAs

were transfected into the indicated cells using Lipofectamine RNAiMAX reagent (Invitrogen, Carlsbad, CA, USA) according to the manufacturer's instructions. DJ-1 cDNA was a gift from Professor Philipp J. Kahle in University of Tübingen, Germany. DJ-1 shRNA and PLAGL2 cDNA were purchased from Gene Copoeia (Guangzhou, China). Transfection was carried out using Lipofectamine 2000 Reagent (Invitrogen). The transfected cells were selected under zeocin or puromycin for 3 to 5 weeks. Stably transfected clones were validated by qRT-PCR and Western blot analysis.

Cell proliferation assays.

Overall, 1000-2000 cells/well were seeded in 96-well plates and incubated at 37 °C for 24h-96h, and then CCK8 reagents (Dojindo, Kamimashiki-gun, Kumamoto, Japan; CK04) were added and co-incubated for 1 h. The absorbance value was then measured at wavelength 450 nm.

Colony formation assay

The cells were seeded at a density of 1500-2500 cells/well in 6-well plates, the medium was changed every 3 days. After 14 days, the colonies were stained with Crystal Violet for 30 minutes and washed three times.

Wound healing, transwell migration and invasion assays

Cell migration assays were performed in 24-well transwell chambers (Corning, NY, USA). Cells in serum-free medium (3×10^4 cells per well) were added to the upper chamber. After 24 h, the number of cells that migrated through the membrane to the lower chamber was counted. For invasion assays, matrigel (1:5, BD) was added to the transwell membrane chambers, incubated for 4 h, and 8×10^4 cells/well was seeded.

Cells migrated to the lower chamber were counted after 48 h. For the scratch wound healing assay, wounds were created in confluent cells by scraping the cell surface using a sterile pipet tip, and then washed with medium to remove free-floating cells and debris. Wound healing within the scraped line was documented daily over 48 or 72 h, and repeated in duplicate at least three times.

Quantitative RT-PCR (qRT-PCR) and RNA-seq

Total RNA was extracted from cells using Trizol reagent (Invitrogen). cDNA reverse transcription, and qRT-PCR were performed using Reverse Transcription PrimeScript 1st Stand cDNA Synthesis kit (TaKaRa, Otsu, Japan) and quantitative PCR reagents SYBR PremixEx TaqTM (TaKaRa) following the manufacturer's instructions. The relative expression of genes was calculated with the $2^{-\Delta\Delta Ct}$ method. The sequences of the primers used are presented in Supplementary Table 6. RNA-Seq was performed with Illumina HiSeq 2000 by Beijing Genomics Institute (BGI, Wuhan, China).

Western blot

Cells were lysed with RIPA buffer (50 mM Tris base, 1.0 mM EDTA, 150 mM NaCl, 0.1% SDS, 1% Triton X-100, 1% sodium deoxycholate, 1 mM PMSF). Proteins were separated in 12% or 15% SDS-PAGE, and transferred to PVDF membranes (Amersham Biosciences). After blocking with Tris-buffered saline (TBS) containing 0.1% Tween 20 and 5% skimmed milk, blots were incubated with the respective primary antibodies for 2 h at room temperature and washed 3 times in TBS with Tween 20. Subsequently, the blots were incubated with HRP-conjugated secondary antibody (diluted 1:10,000; Santa Cruz Biotechnology) 2 h at room temperature.

Finally, the blots were visualized by enhanced chemiluminescence (Amersham Biosciences). The cytoplasmic and nuclear proteins were prepared using NE-PER nuclear and cytoplasmic extraction reagents.

Supplementary figure legends

Supplementary Figure 1. DJ-1 is upregulated in human CRC. A. The mRNA expression of DJ-1 was analyzed by GEPIA based on TCGA and GTEx data. **B.** Western blots analysis of DJ-1 expression in CRC tissues and patient-matched adjacent normal tissues.

Supplementary Figure 2. DJ-1 overexpression promotes HCT116 cell proliferation, migration and invasion. DJ-1 was consistently overexpressed in HCT116 cells, whereas the empty vector was used as control. **A.** Colony formation. Cells were cultured for 2 weeks. Mean colony counts at the bottom were from three independent experiments. **B.** Cell proliferation assay was performed by CCK8 kit. **C.** Wound healing model. Lines indicated the original wound boundaries. Scale bar, 500 μm. **D.** Quantitative analysis of cell migration and matrigel invasion assays. Migration was proceeded for 24 h, invasion for 48 h. Scale bar, 100 μm. **E.** qRT-PCR and Western blot analysis of DJ-1 expression. * P＜0.05, ** P＜0.01.

Supplementary Figure 3. DJ-1 silencing inhibits aggressive phenotype of HCT116 cells. HCT116 cells were stably transfected with shDJ-1 or negative control vector. **A.** Colony formation. Cells were cultured for 2 weeks. Mean colony counts at the bottom were from three independent experiments. **B.** Cell proliferation assay was performed by CCK8 kit. **C.** Wound healing model. Lines indicated the original wound boundaries. Scale bar, 500 μm. **D.** Quantitative analysis of cell migration and matrigel invasion assays. Migration was proceeded for 24 h, invasion for 48 h. Scale bar, 100 μm. **E.** qRT-PCR and Western blot analysis of DJ-1 expression. * P＜0.05, ** P＜

0.01, *** P＜0.001.

Supplementary Figure 4. DJ-1 activates both Hedgehog signaling and Wnt signaling. A. The signaling pathways of basal cell carcinoma were annotated by KEGG pathway database. **B-D.** qRT-PCR was used to examine the key Hedgehog signaling components GLI1, GLI2 and PTCH1, as well as Wnt signaling target genes TCF7, CCND1, FGF9 and AXIN2 in HCT116, SW480 and SW620 cells stably transfected with DJ-1 cDNA, DJ-1 shRNA or vector. **E-F.** Western blot analysis of CCND1 expression in HCT116, SW480 and SW620 cells which overexpressed or downregulated DJ-1.

Supplementary Figure 5. BMP4 is upregulated in human CRC and associated with Wnt signaling. A. The mRNA expression of BMP4 was analyzed by GEPIA based on TCGA and GTEx data. **B.** GEPIA analysis of disease free survival based on BMP4 mRNA. **C.** GEPIA correlation analysis of BMP4 and Wnt target genes TCF7, CCND1 and AXIN2 expression in CRC patient, respectively.

Supplementary Figure 6. DJ-1 can induce BMP4 transcription by activating Wnt signaling. A. qRT-PCR analysis of BMP4 expression in HCT116, SW480 and SW620 cells stably transfected with DJ-1 cDNA, DJ-1 shRNA or vector. **B.** qRT-PCR analysis of BMP4 expression in indicated cells treated with Sulindac for 36h. **C.** qRT-PCR analysis of BMP4 expression in indicated cells transfected with two special BMP4 siRNAs, respectively. **D.** qRT-PCR analysis of BMP4 mRNA expression in indicated cells transfected with BMP4 cDNA. * P＜0.05, ** P＜0.01, *** P＜0.001.

Supplementary Figure 7. BMP4 doesn’t influence DJ-1-induced proliferation of

CRC cells. A, B. Proliferation of CRC cells transfected with BMP4 siRNAs (A) or BMP4 cDNA (B) examined by CCK8 kit.

Supplementary Figure 8. CCND1 is involved in DJ-1-induced proliferation of CRC cells. SW620, DJ-1-overexpressed HCT116 (HCT116-DJ-1) and SW480 (SW480-DJ-1) cells were transfected with specific CCND1 siRNA, respectively. **A.** qRT-PCR analysis of CCND1 mRNA expression. **B.** Western blot analysis of CCND1 protein expression. **C.** Cell proliferation assay performed by CCK8 kit. **D.** Colony formation. * P＜0.05, ** P＜0.01.

Supplementary Figure 9. PLAGL2 is upregulated in human CRC and associated with Wnt signaling. A. The mRNA expression of PLAGL2 was analyzed by GEPIA based on TCGA and GTEx data. **B.** GEPIA analysis of overall survival based on PLAGL2 mRNA. **C.** GEPIA correlation analysis of PLAGL2 and Wnt target genes TCF7, CCND1 and AXIN2 expression in CRC patients, respectively.

Supplementary Figure 10. DJ-1 can enhance PLAGL2 transcription to activate Wnt signaling. A. qRT-PCR analysis of PLAGL2 (PL) expression in HCT116, SW480 and SW620 cells stably transfected with DJ-1 cDNA, DJ-1 shRNA or vector. **B.** qRT-PCR analysis of of PLAGL2 (PL), BMP4 and CCND1 mRNA expression in indicated cells transfected with two specific PLAGL2 siRNAs, respectively. * P＜0.05, ** P＜0.01, *** P＜0.001.

Supplementary Figure 11. PLAGL2 is required for DJ-1-induced CRC migration. SW620, DJ-1-overexpressed HCT116 (HCT116-DJ-1) and SW480 (SW480-DJ-1) cells were transfected with two specific PLAGL2 (PL) siRNAs, respectively. Then,

scratch wound healing assay was performed to examine the migratory capacity of CRC cells. Scale bar, 500 μm.

Supplementary Figure 12. PLAGL2 overexpression could promote Wnt signaling activity and proliferation in DJ-1-konckdown cells. HCT116-shDJ-1, SW480 and SW620-shDJ-1 cells were transfected with PLAGL2 cDNA or vector. **A.** Protein levels of activated-β-catein, BMP4, CCND1 and PLAGL2 were determined by Western blot. **B.** Wnt activity was analyzed by TOP-Flash/FOP-Flash assay. **C.** Proliferative activity was detected by colony formation assay. **D.** Proliferation of CRC cells was examined by CCK8 kit. ** P＜0.01, *** P＜0.001.

Supplementary Figure 13. PLAGL2 overexpression could restore migration and invasion activity in DJ-1-konckdown cells. HCT116-shDJ-1, SW480 and SW620-shDJ-1 cells were transfected with PLAGL2 cDNA or vector. **A.** scratch wound healing assay was performed to examine the migratory capacity of CRC cells. Scale bar, 500 μm. **B.** Migration and invasion activity were assessed by transwell migration and matrigel invasion assays. Scale bar, 100 μm. Migration was analyzed at 24 h, invasion at 48 h. * P＜0.05, ** P＜0.01, *** P＜0.001.

Supplementary Figure 14. Hedgehog signaling is involved in DJ-1-induced and Wnt signaling-mediated expression of BMP4 and CRC cell proliferation. SW620, DJ-1-overexpressed HCT116 (HCT116-DJ-1) and SW480 (SW480-DJ-1) cells were treated with 20μM GANT61 for 24h. **A.** qRT-PCR analysis of GLI1, GLI2 and BMP4 mRNA expression. **B.** Western blot analysis of BMP4 and CCND1 protein expression. **C.** Cell proliferation assay performed by CCK8 kit. * P＜0.05, ** P＜0.01.

Supplementary Figure 15. BMP4 expression is positively correlated with Hedgehog signaling. A. GEPIA correlation analysis of BMP4 and Hedgehog signaling key members GLI1, GLI2 and PTCH1 expression in CRC patients, respectively. **B.** The mRNA expression of GLI1, GLI2 and PTCH1 in CRC was analyzed by GEPIA based on TCGA and GTEx data, respectively.

Supplementary Figure 16. Hedgehog signaling contributes to CRC progression. A. Comparison of GLI1, GLI2 and PTCH1 mRNA expression in different stage CRC patients by GEPIA. **B.** GEPIA analysis of overall survival based on GLI1, GLI2 and PTCH1 mRNA, respectively.

Fig. S1

A

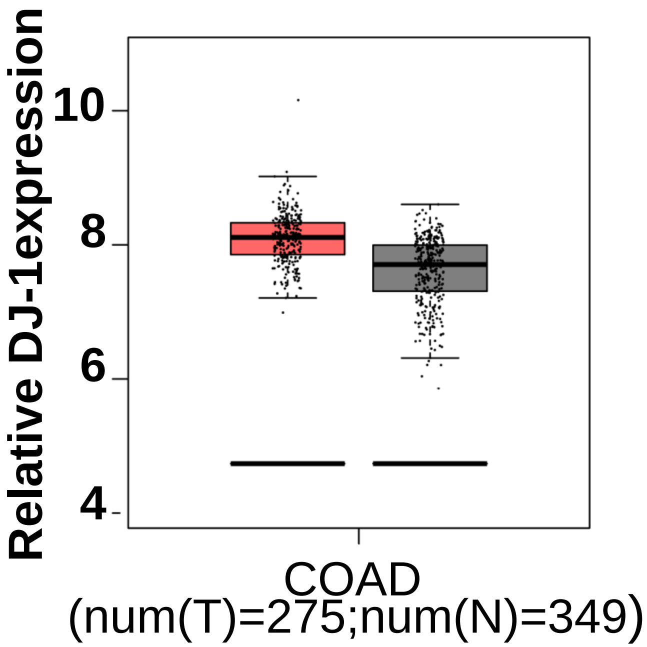

Relative DJ-1expression
10
8
6
4
COAD
(num(T)=275;num(N)=349)

B

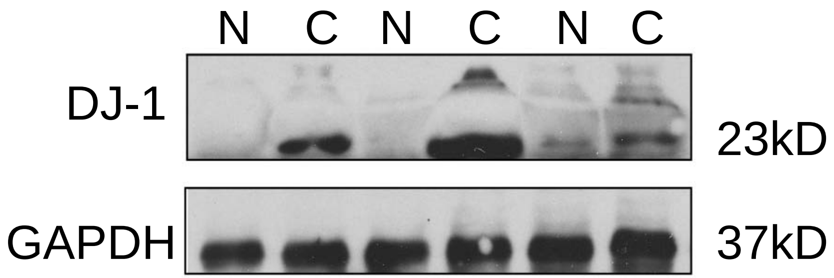

N
C
N
C
N
C
DJ-1
23kD
GAPDH
37kD

Fig. S2

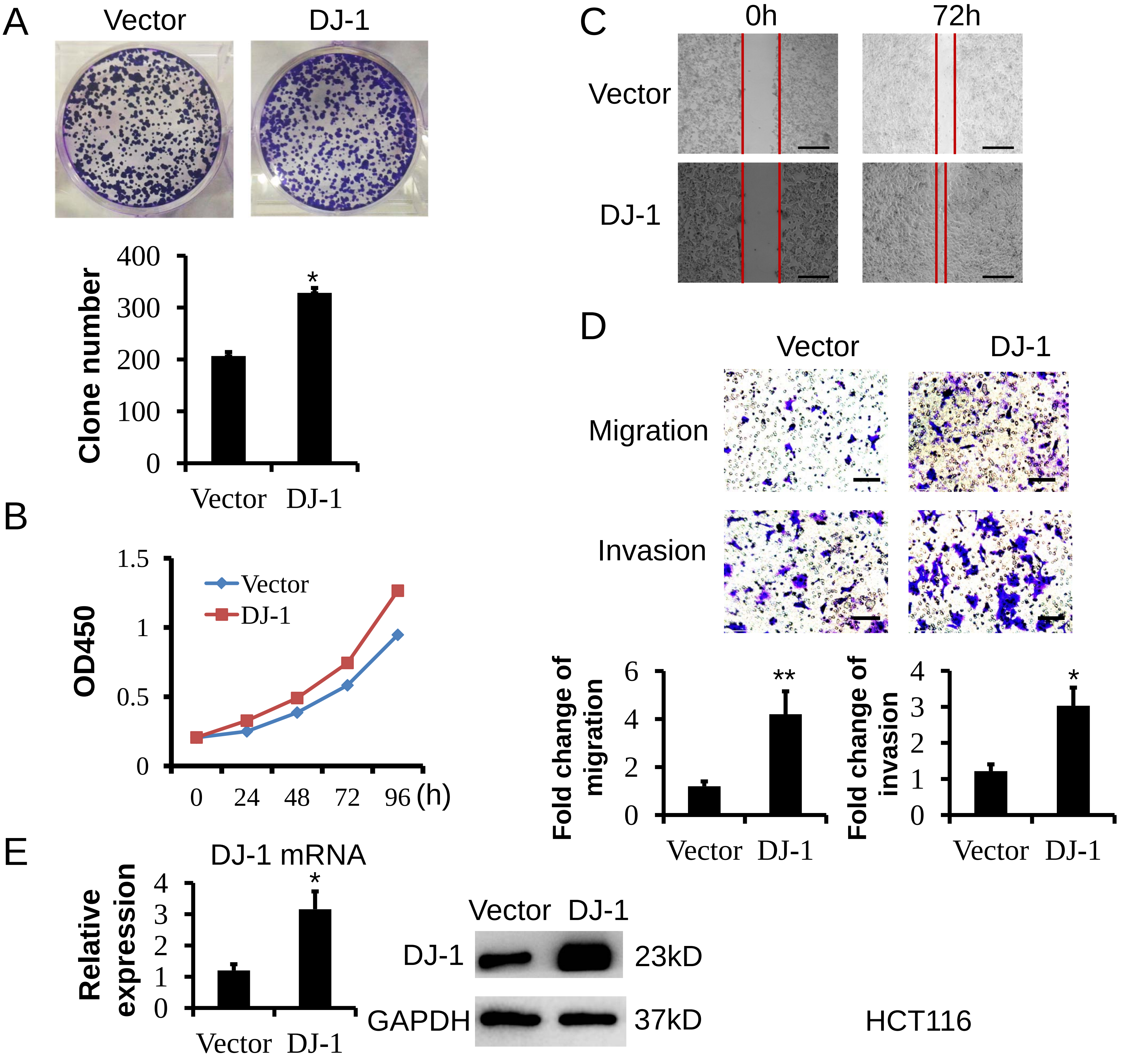

A
Vector
DJ-1
Clone number
400
300
200
100
0
*
Vector DJ-1
B
OD450
1.5
1
0.5
0
Vector
DJ-1
0 24 48 72 96 (h)
C
0h
72h
Vector
DJ-1
D
Vector
DJ-1
Migration
Invasion
Fold change of migration
6
4
2
0
**
Vector DJ-1
Fold change of invasion
4
3
2
1
0
*
Vector DJ-1
E
DJ-1 mRNA
Relative expression
4
3
2
1
0
*
Vector DJ-1
Vector DJ-1
DJ-1
23kD
GAPDH
37kD
HCT116

Fig. S3

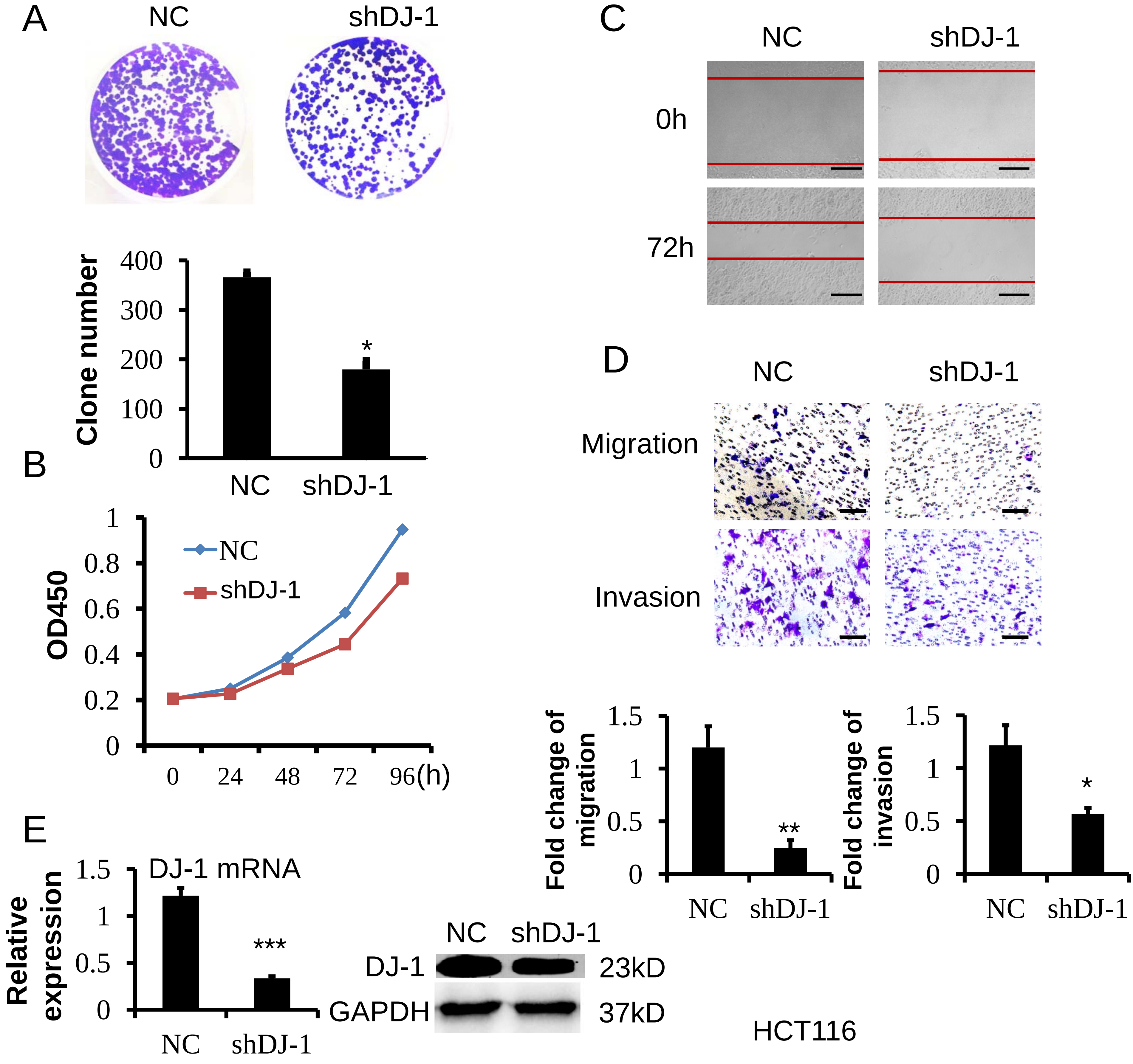
A
NC
shDJ-1
Clone number
400
300
200
100
0
*
NC
shDJ-1
B
OD450
1
0.8
0.6
0.4
0.2
0
NC
shDJ-1
0
24
48
72
96(h)
C
NC
shDJ-1
0h
72h
D
NC
shDJ-1
Migration
Invasion
Fold change of migration
1.5
1
0.5
0
**
NC
shDJ-1
Fold change of invasion
1.5
1
0.5
0
*
NC
shDJ-1
E
Relative expression
1.5
1
0.5
0
DJ-1 mRNA
***
NC
shDJ-1
NC
shDJ-1
DJ-1
GAPDH
23kD
37kD
HCT116

Fig. S4

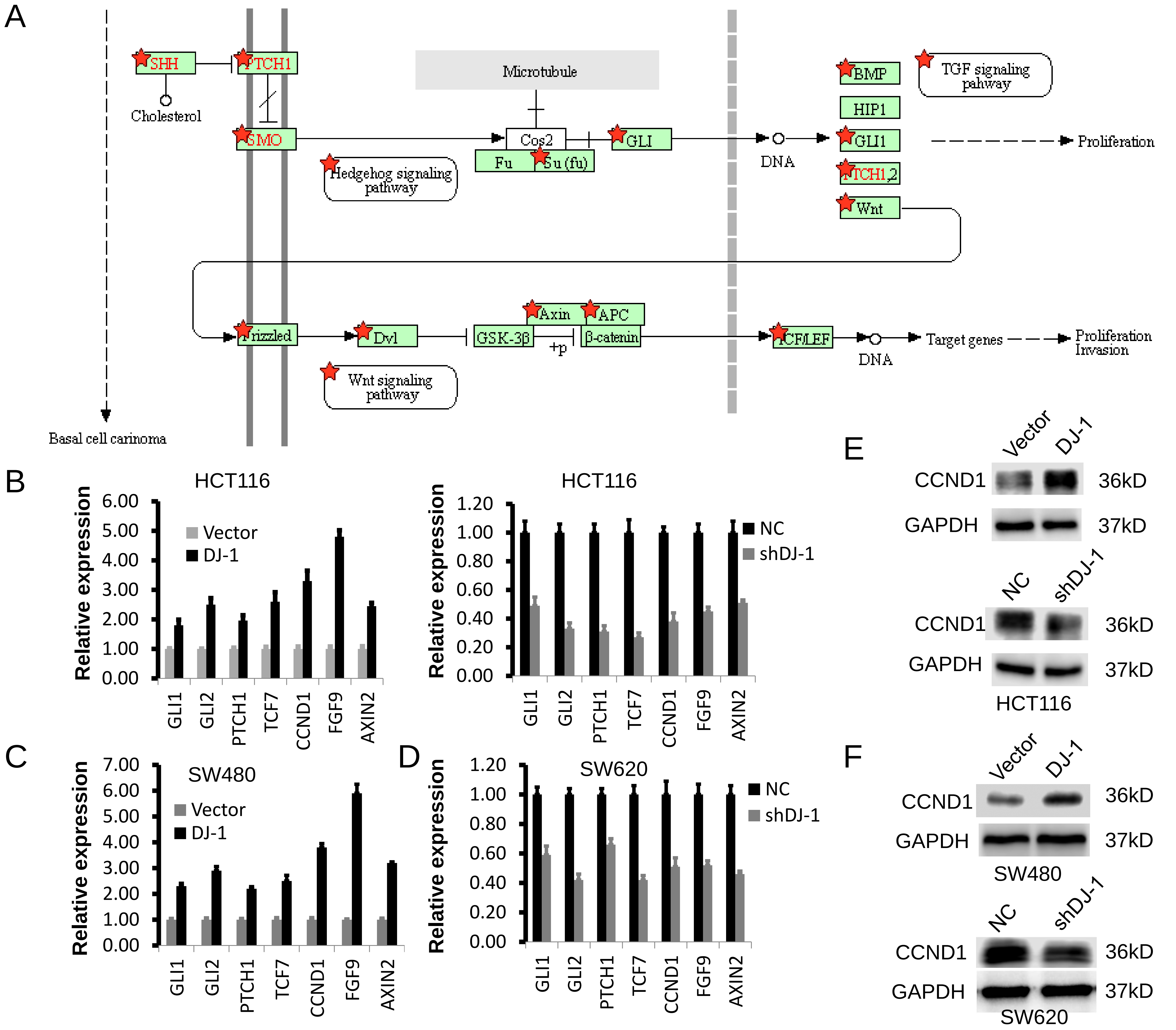
A
SHH
PTCH1
Cholesterol
SMO
Microtubule
Cos2
Fu
Su (fu)
GLI
Hedgehog signaling pathway
DNA
BMP
HIP1
GLI1
PTCH1,2
Wnt
TGF signaling pahway
Proliferation
Frizzled
Dvl
GSK-3β
Axin
APC
β-catenin
+p
TCF/LEF
DNA
Target genes
Proliferation Invasion
Wnt signaling pathway
Basal cell carinoma
B
HCT116
Relative expression
Vector
DJ-1
NC
shDJ-1
GLI1
GLI2
PTCH1
TCF7
CCND1
FGF9
AXIN2
C
SW480
D
SW620
E
Vector
DJ-1
CCND1
GAPDH
36kD
37kD
NC
shDJ-1
HCT116
F
SW480
SW620

Fig. S5

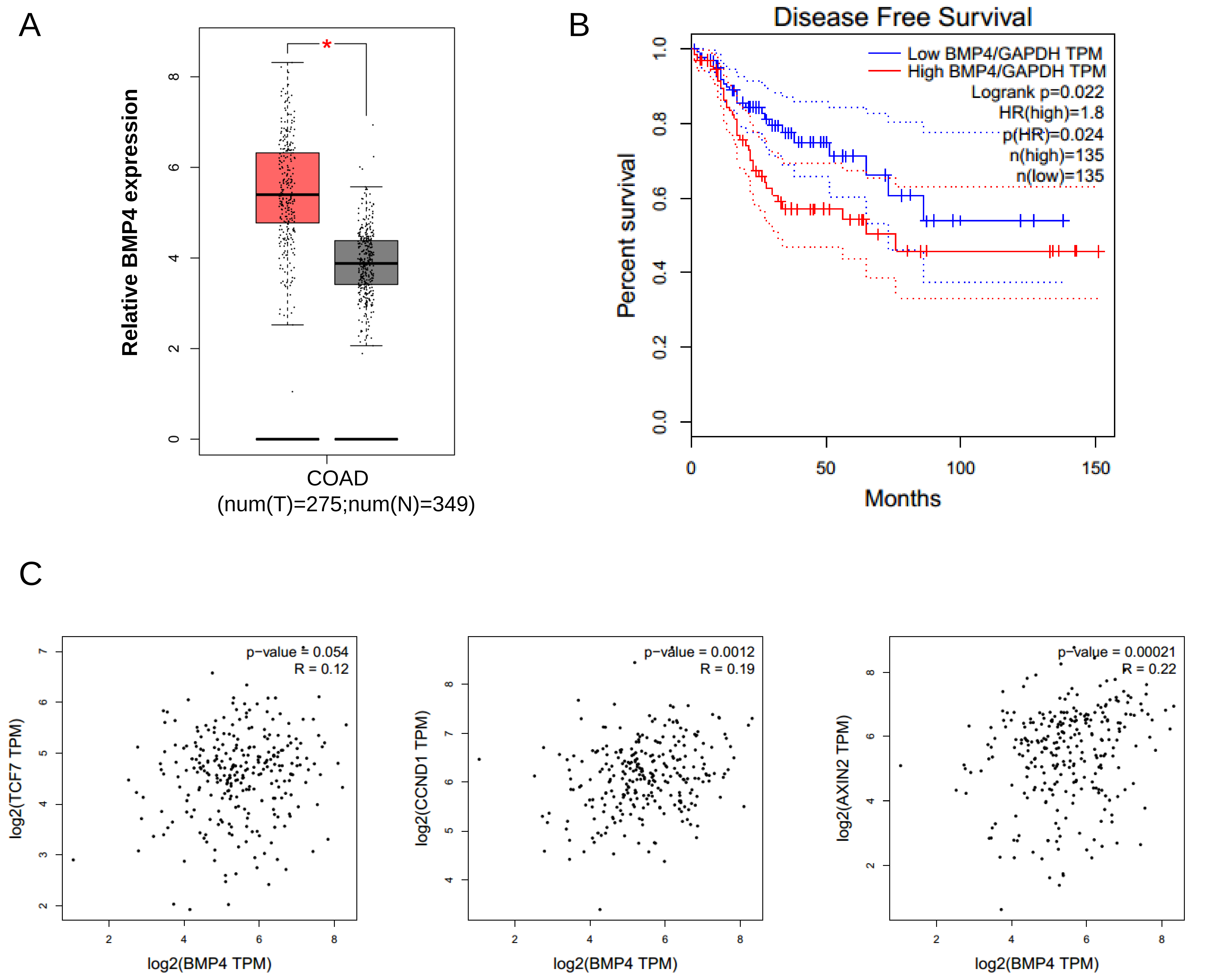
A
Relative BMP4 expression
*
COAD
(num(T)=275;num(N)=349)
B
Disease Free Survival
Low BMP4/GAPDH TPM
High BMP4/GAPDH TPM
Logrank p=0.022
HR(high)=1.8
p(HR)=0.024
n(high)=135
n(low)=135
Percent survival
Months
C
p-value = 0.054
R = 0.12
log2(TCF7 TPM)
log2(BMP4 TPM)
p-value = 0.0012
R = 0.19
log2(CCND1 TPM)
log2(BMP4 TPM)
p-value = 0.00021
R = 0.22
log2(AXIN2 TPM)
log2(BMP4 TPM)

Fig. S6

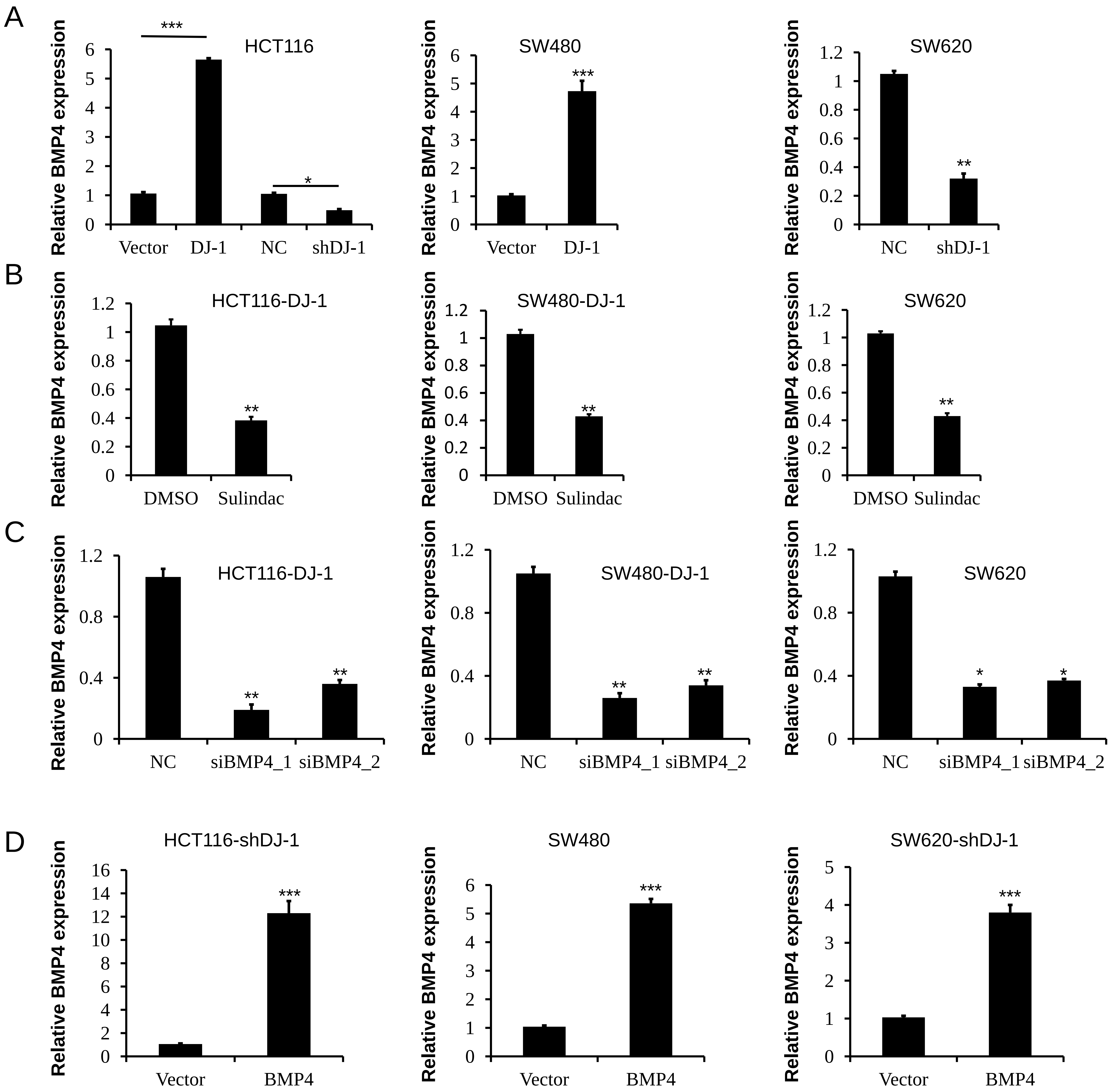
A
HCT116
SW480
SW620
Relative BMP4 expression
Vector
DJ-1
NC
shDJ-1
B
HCT116-DJ-1
SW480-DJ-1
DMSO
Sulindac
C
siBMP4_1
siBMP4_2
D
HCT116-shDJ-1
SW620-shDJ-1
BMP4

Fig. S7

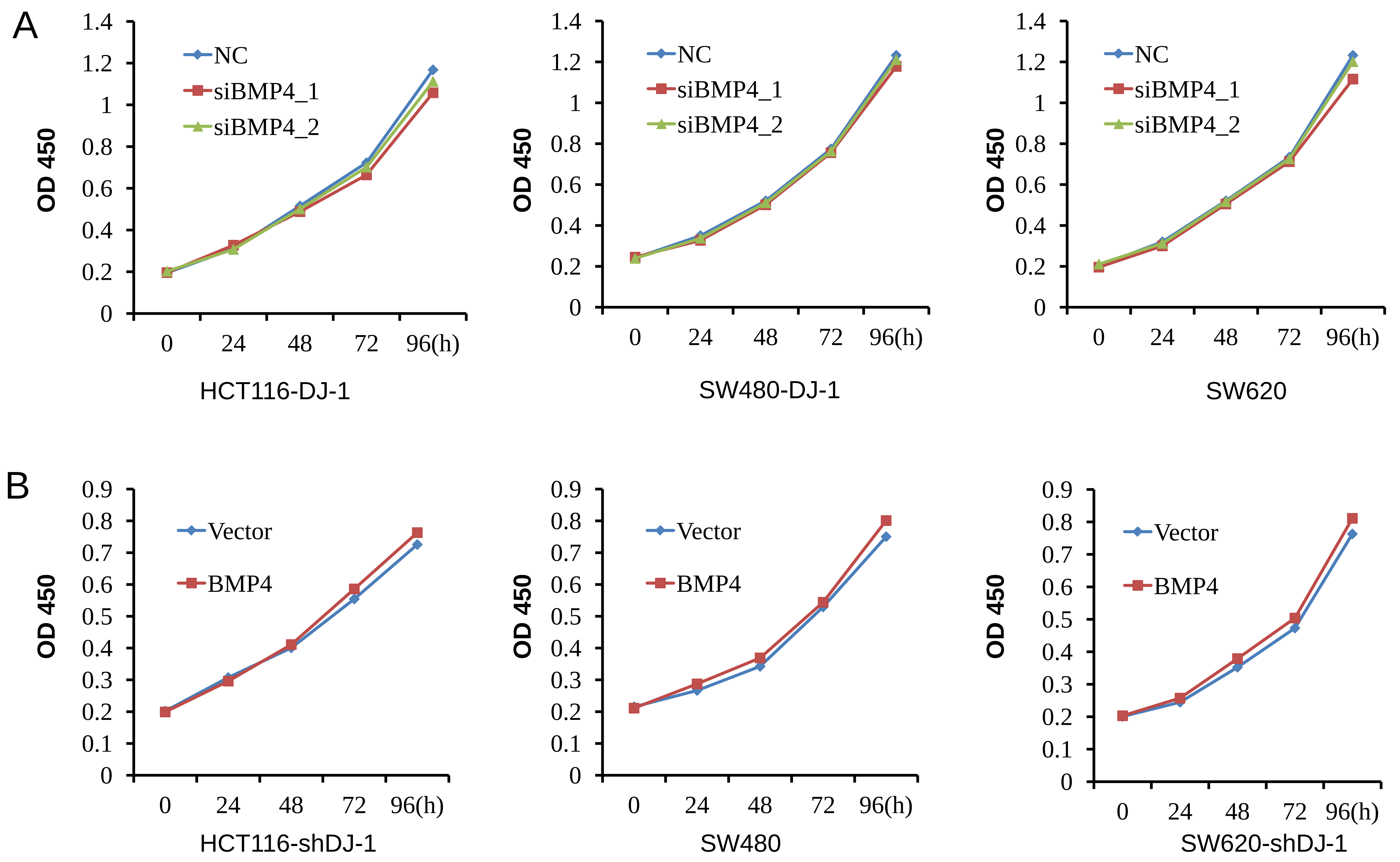
A
OD 450
NC
siBMP4_1
siBMP4_2
0
24
48
72
96(h)
HCT116-DJ-1
SW480-DJ-1
SW620
B
Vector
BMP4
HCT116-shDJ-1
SW480
SW620-shDJ-1

Fig. S8

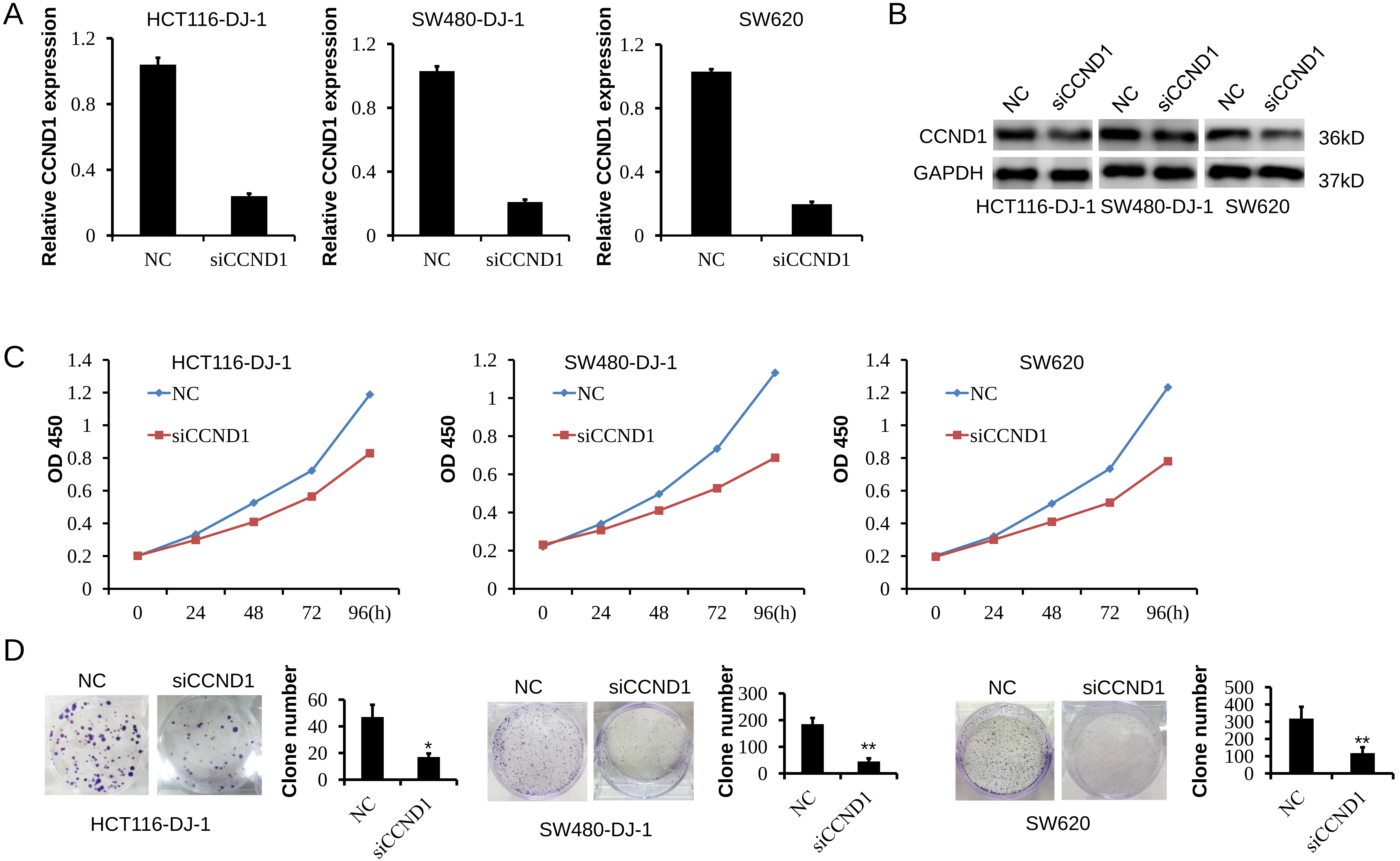

A
HCT116-DJ-1
SW480-DJ-1
SW620
Relative CCND1 expression
1.2
0.8
0.4
0
NC
siCCND1
B
NC
siCCND1
CCND1
GAPDH
36kD
37kD
HCT116-DJ-1 SW480-DJ-1 SW620
C
OD 450
NC
siCCND1
1.4
1.2
1
0.8
0.6
0.4
0.2
0
24
48
72
96(h)
D
Clone number
*
**
HCT116-DJ-1
SW480-DJ-1
SW620
60
40
20
300
200
100
500
400

Fig. S9

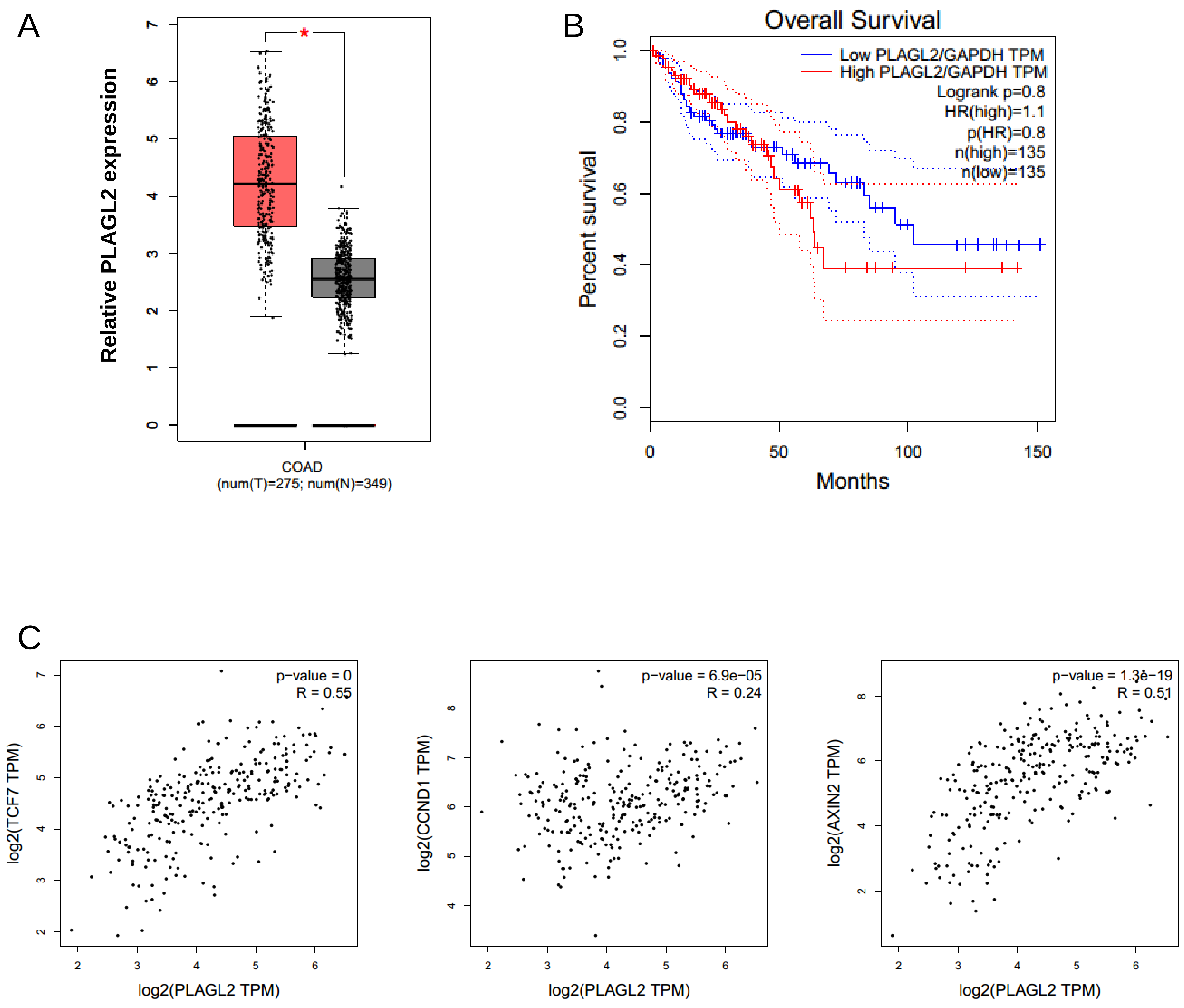
A
Relative PLAGL2 expression
COAD
(num(T)=275; num(N)=349)
B
Overall Survival
Low PLAGL2/GAPDH TPM
High PLAGL2/GAPDH TPM
Logrank p=0.8
HR(high)=1.1
p(HR)=0.8
n(high)=135
n(low)=135
Percent survival
Months
C
p−value = 0
R = 0.55
log2(TCF7 TPM)
log2(PLAGL2 TPM)
p−value = 6.9e−05
R = 0.24
log2(CCND1 TPM)
log2(PLAGL2 TPM)
p−value = 1.3e−19
R = 0.51
log2(AXIN2 TPM)
log2(PLAGL2 TPM)

Fig. S10

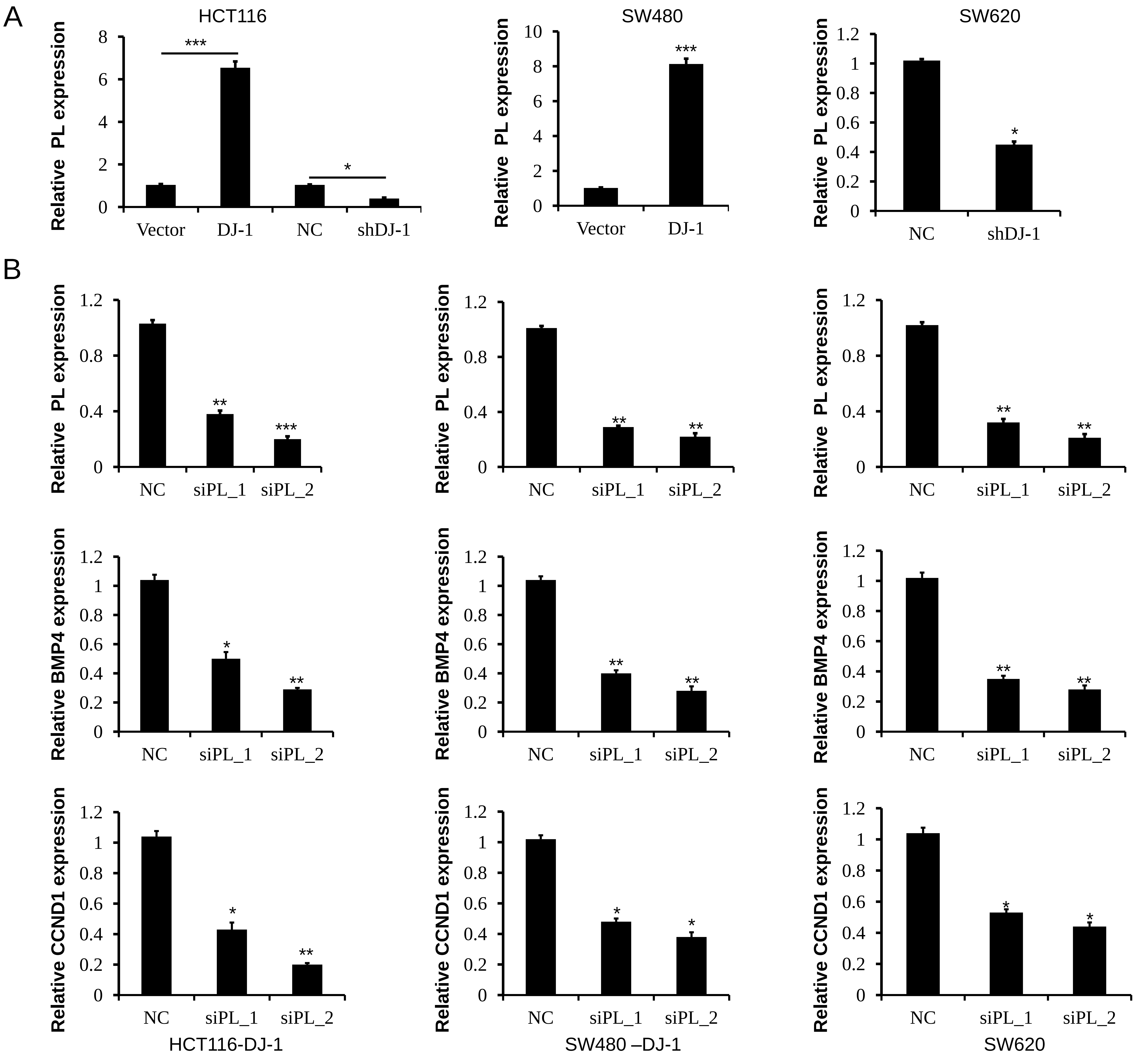
A
HCT116
SW480
SW620
Relative PL expression
Vector
DJ-1
NC
shDJ-1
***
*
B
Relative PL expression
Relative BMP4 expression
Relative CCND1 expression
NC
siPL_1
siPL_2
HCT116-DJ-1
SW480 –DJ-1
SW620

Fig. S11

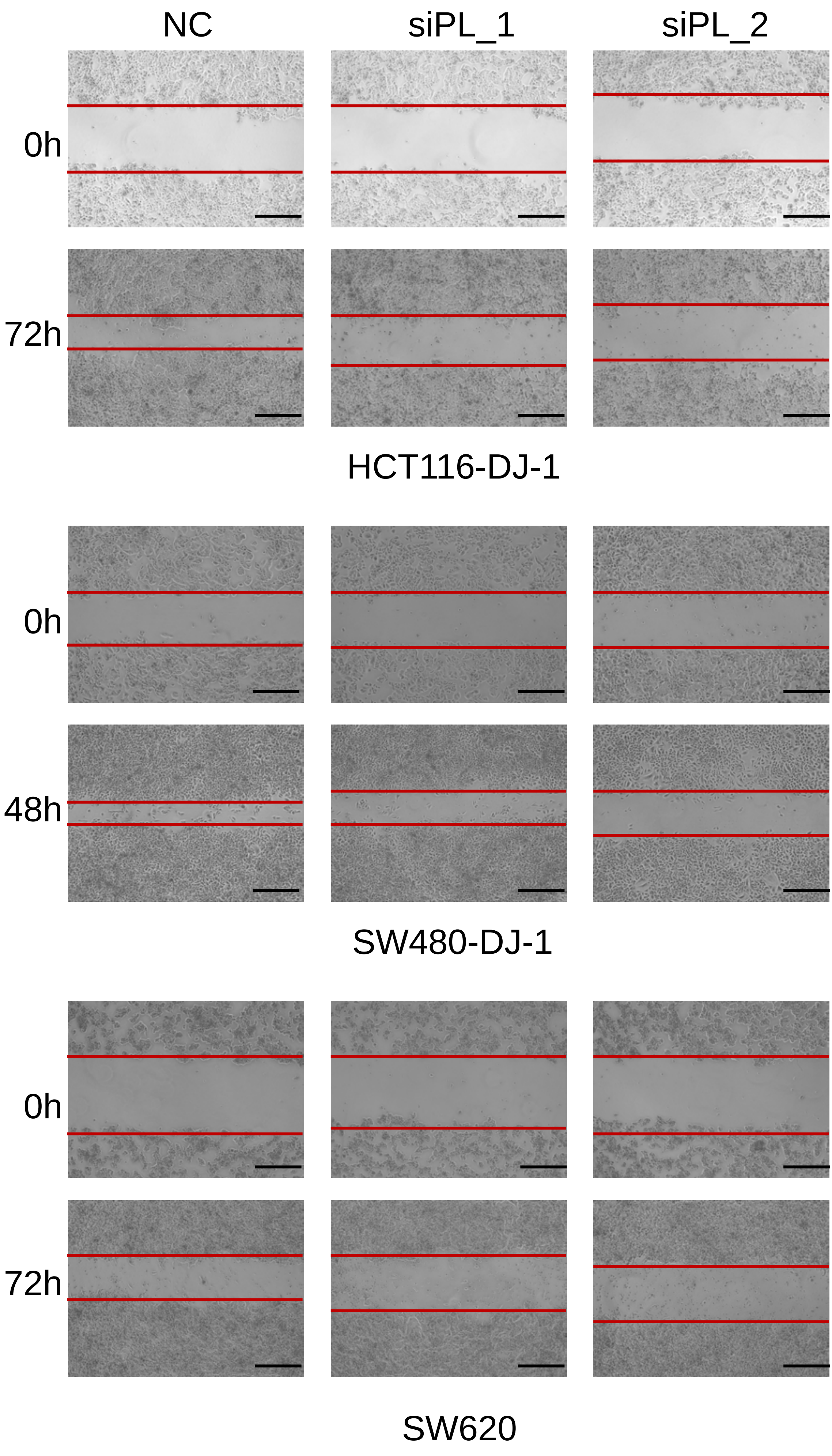

Fig. S12

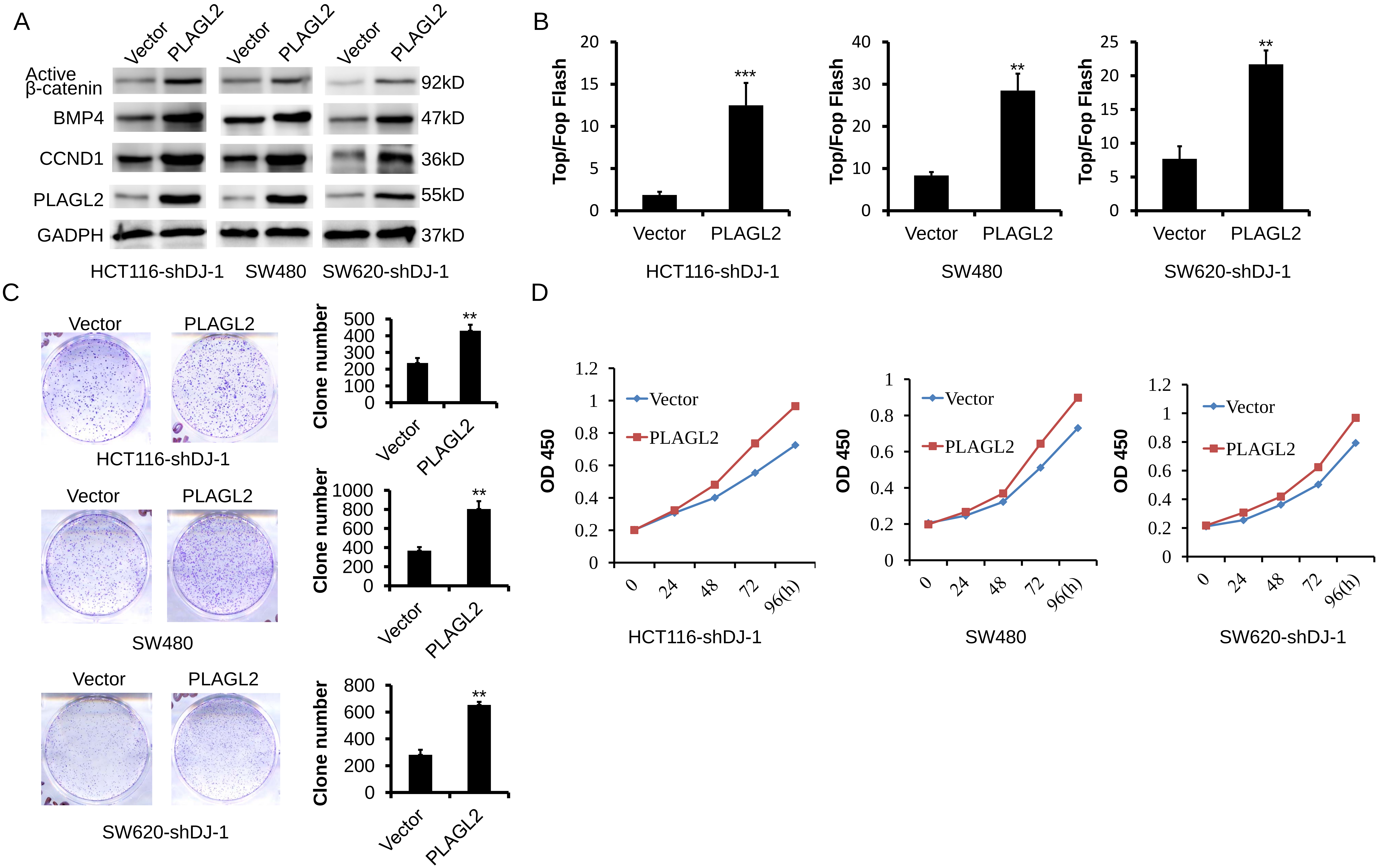
A
Vector
PLAGL2
Active β-catenin
BMP4
CCND1
PLAGL2
GADPH
92kD
47kD
36kD
55kD
37kD
HCT116-shDJ-1
SW480
SW620-shDJ-1
B
Top/Fop Flash
***
**
**
HCT116-shDJ-1
SW480
SW620-shDJ-1
C
Clone number
HCT116-shDJ-1
SW480
SW620-shDJ-1
D
OD 450
Vector
PLAGL2
0
24
48
72
96(h)
HCT116-shDJ-1
SW480
SW620-shDJ-1

Fig. S13

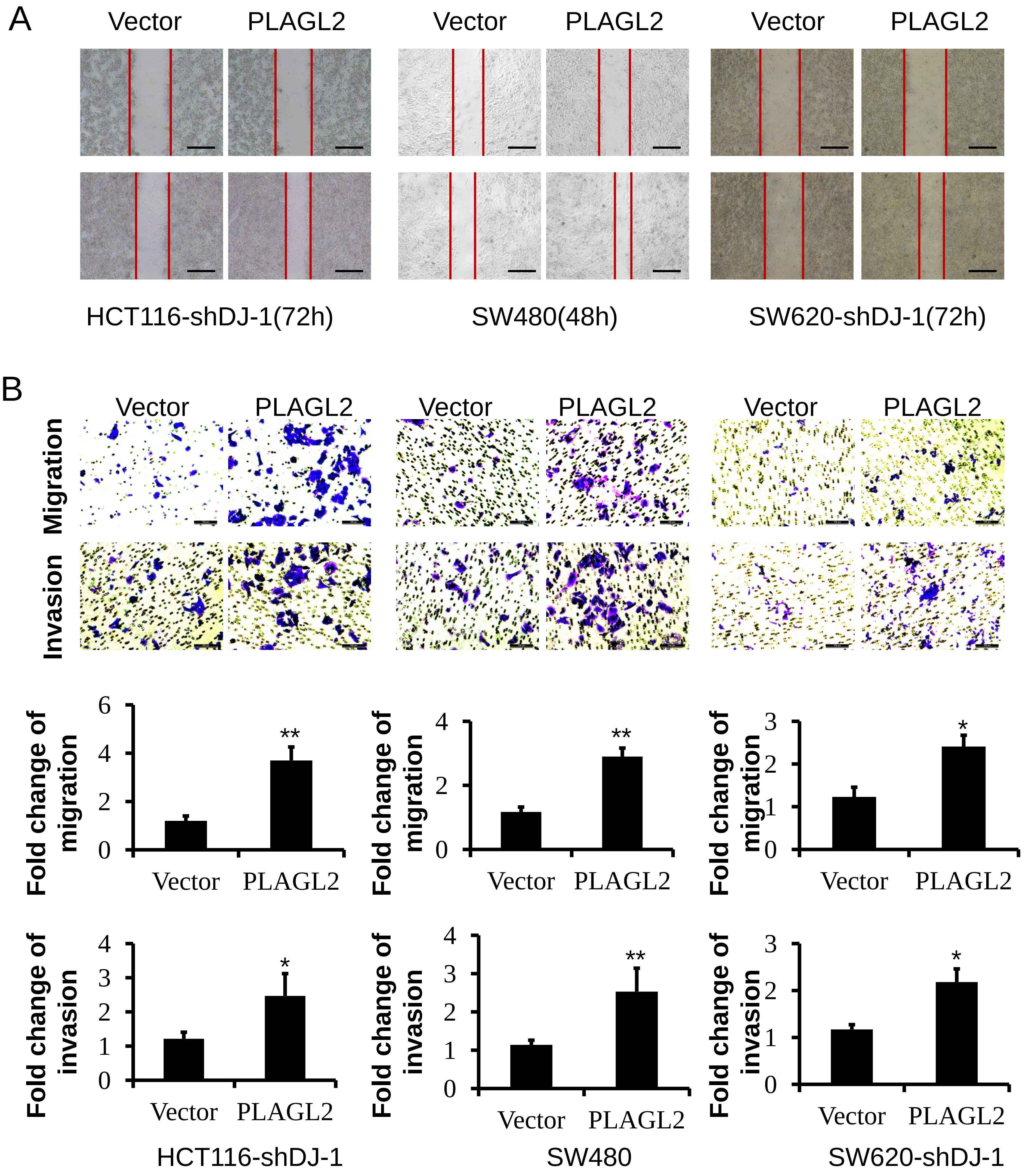
A
Vector
PLAGL2
Vector
PLAGL2
Vector
PLAGL2
HCT116-shDJ-1(72h)
SW480(48h)
SW620-shDJ-1(72h)
B
Vector
PLAGL2
Vector
PLAGL2
Vector
PLAGL2
Migration
Invasion
Fold change of migration
Fold change of invasion
Vector
PLAGL2
**
*
HCT116-shDJ-1
SW480
SW620-shDJ-1

Fig. S14

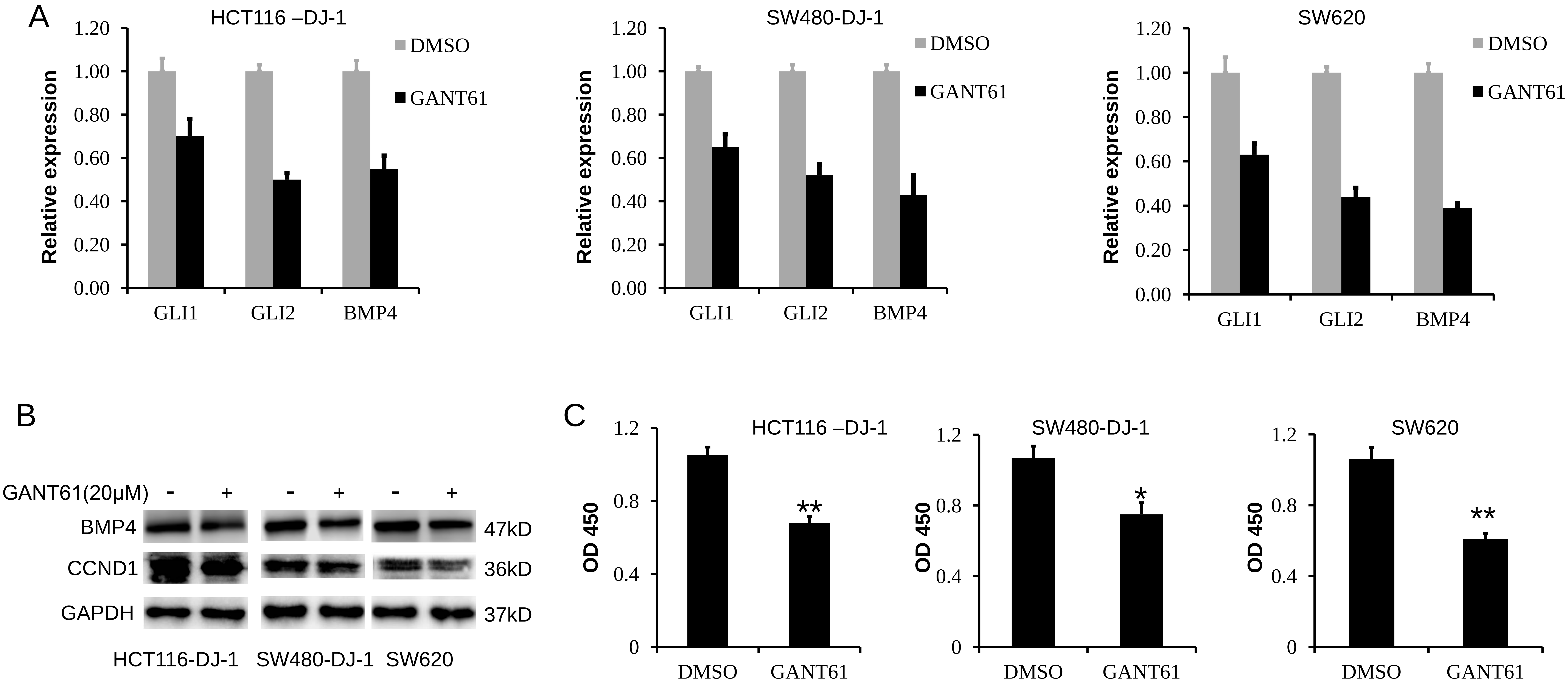
A
HCT116 –DJ-1
SW480-DJ-1
SW620
Relative expression
1.20
1.00
0.80
0.60
0.40
0.20
0.00
DMSO
GANT61
GLI1
GLI2
BMP4
B
GANT61(20μM) - + - + - +
BMP4
CCND1
GAPDH
47kD
36kD
37kD
HCT116-DJ-1
SW480-DJ-1
SW620
C
OD 450
1.2
0.8
0.4
0
DMSO
GANT61
**
*

Fig. S15

A

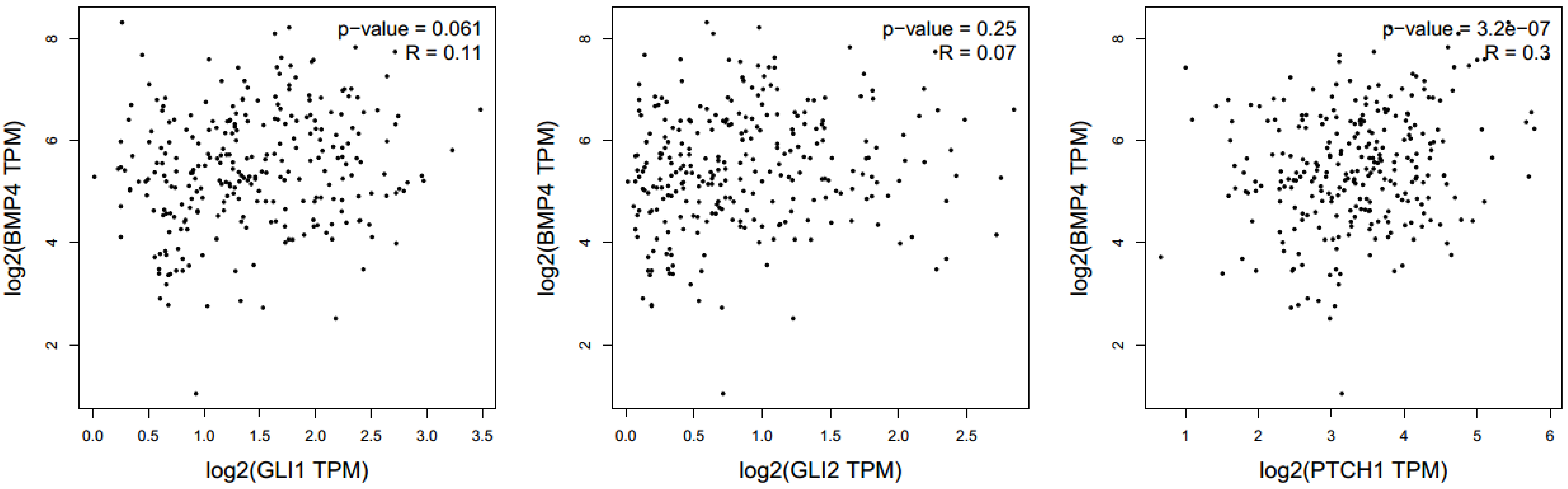
p−value = 0.061
R = 0.11
log2(BMP4 TPM)
log2(GLI1 TPM)
p−value = 0.25
R = 0.07
log2(BMP4 TPM)
log2(GLI2 TPM)
p−value = 3.2e−07
R = 0.3
log2(BMP4 TPM)
log2(PTCH1 TPM)

B

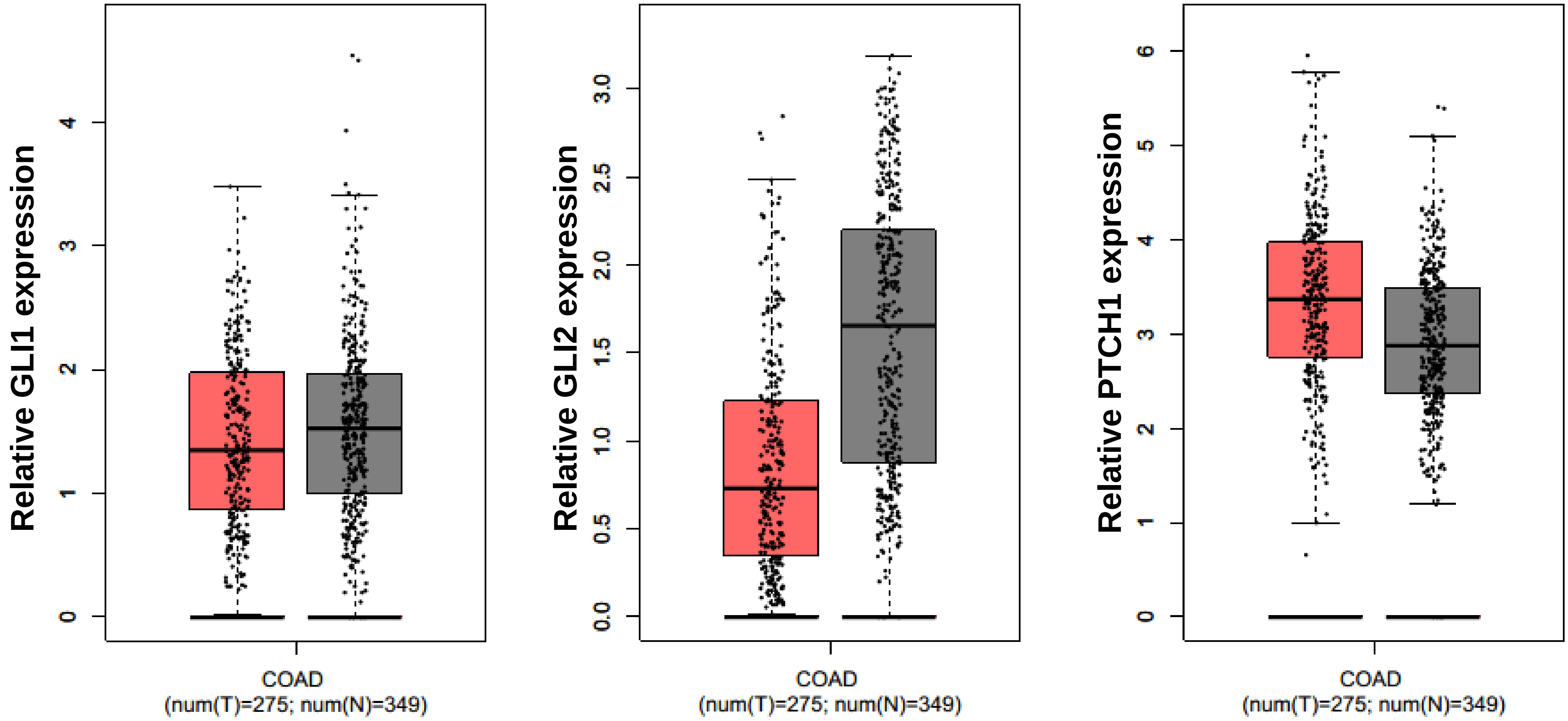
Relative GLI1 expression
COAD
(num(T)=275; num(N)=349)
Relative GLI2 expression
COAD
(num(T)=275; num(N)=349)
Relative PTCH1 expression
COAD
(num(T)=275; num(N)=349)

Fig. S16

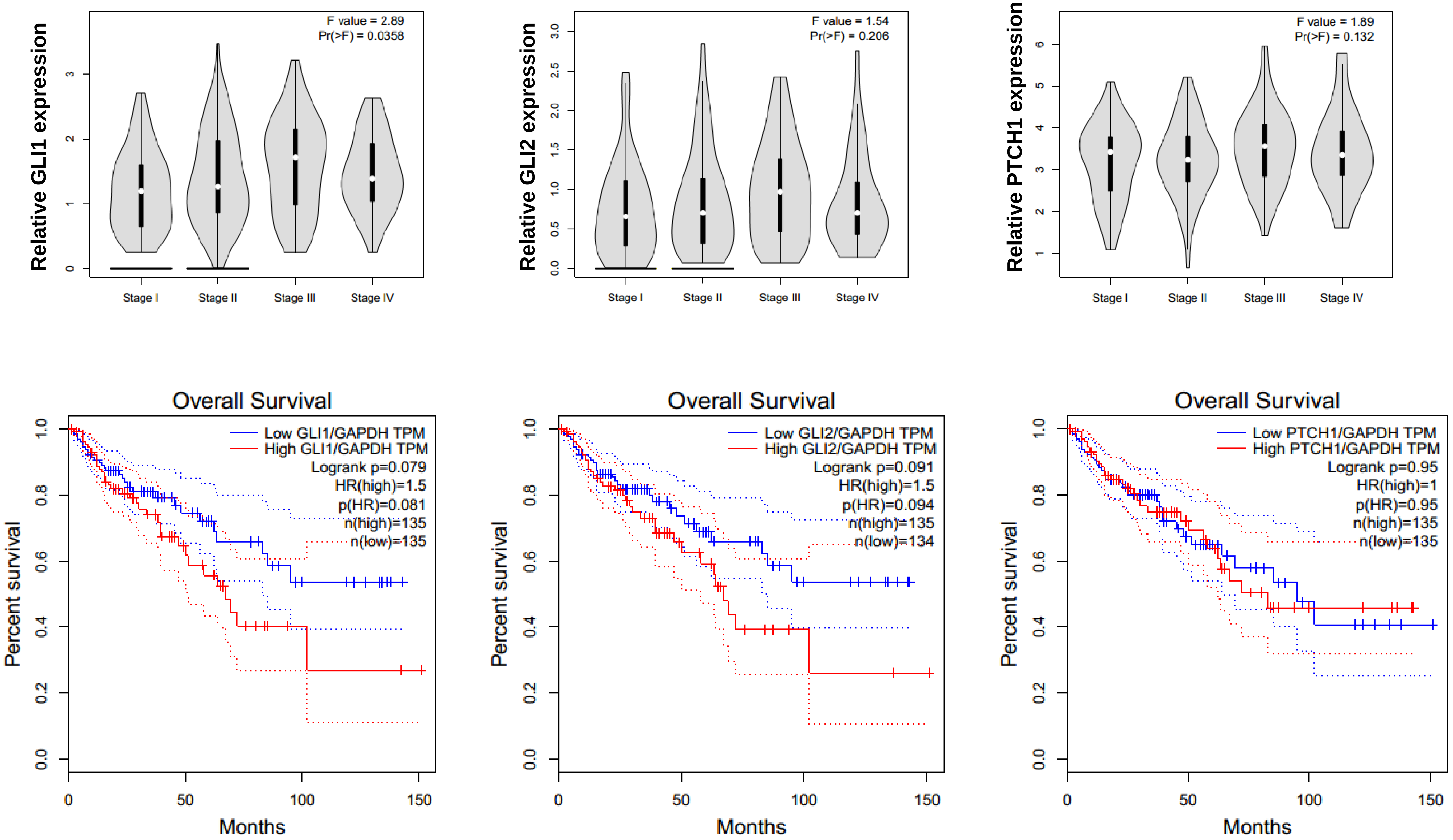
F value = 2.89
Pr(>F) = 0.0358
Relative GLI1 expression
Stage I
Stage II
Stage III
Stage IV
F value = 1.54
Pr(>F) = 0.206
Relative GLI2 expression
Stage I
Stage II
Stage III
Stage IV
F value = 1.89
Pr(>F) = 0.132
Relative PTCH1 expression
Stage I
Stage II
Stage III
Stage IV
Overall Survival
Low GLI1/GAPDH TPM
High GLI1/GAPDH TPM
Logrank p=0.079
HR(high)=1.5
p(HR)=0.081
n(high)=135
n(low)=135
Percent survival
Months
Overall Survival
Low GLI2/GAPDH TPM
High GLI2/GAPDH TPM
Logrank p=0.091
HR(high)=1.5
p(HR)=0.094
n(high)=135
n(low)=134
Percent survival
Months
Overall Survival
Low PTCH1/GAPDH TPM
High PTCH1/GAPDH TPM
Logrank p=0.95
HR(high)=1
p(HR)=0.95
n(high)=135
n(low)=135
Percent survival
Months
