# Supplementary material for: DJ-1 promotes colorectal cancer progression through activating PLAGL2/Wnt/BMP4 axis
Source: Cell Death Dis. 2018 Aug 29;9(9):865. doi: 10.1038/s41419-018-0883-4 (PMC6115399; doi:10.1038/s41419-018-0883-4)
Supplement: Supplementary file 2 — Supplementary Table 1,3-6 [file 41419_2018_883_MOESM2_ESM.pdf]

Supplementary Table 1. Clinico-pathologic parameters of colorectal cancer patients

Characteristics	Number (%)
Gender	n= 181
Male	109 (60.2%)
Female	72 (39.8%)
Age (years)	n= 181
<65	94 (51.9%)
≥65	87 (48.1%)
Tumor location	n= 181
Colon	101 (55.8%)
Rectum	80 (44.2%)
AJCC stages	n= 181
p I	21 (11.6%)
p II	72 (39.8%)
p III/IV	88 (48.6%)
Pathologic grade	n= 181
Well	42 (23.2%)
Moderate	109 (60.2%)
Poor	30 (16.6%)
Tumor size, cm	n= 135
≤3	52 (38.5%)
＞3	83 (61.5%)

Supplementary Table 3. DJ-1 Up-regulated Wnt signaling accocited genes analyzed by DAVID based on TGGA

ID	Gene Name	FPKM.DJ-1	FPKM.Vector	log2_DJ-1_Vector
ENSG00000005339	CREBBP	6.92406568	2.989047681	1.211933
ENSG00000168772	CXXC4	0.51959637	0.037202967	3.803902
ENSG00000134318	ROCK2	30.84483	9.977910286	1.628219
ENSG00000164736	SOX17	0.021732	1.00E-06	14.40753
ENSG00000163904	SENP2	10.7555676	5.243627992	1.036446
ENSG00000134982	APC	4.10750284	1.453119601	1.499108
ENSG00000168646	AXIN2	8.77161157	1.776884799	2.303492
ENSG00000166869	CHP2	0.021367	1.00E-06	14.3831
ENSG00000070808	CAMK2A	0.01655185	0.004399813	1.911479
ENSG00000058404	CAMK2B	0.09434082	0.019236602	2.294028
ENSG00000055130	CUL1	24.441045	7.749850713	1.657066
ENSG00000110092	CCND1	243.029864	116.1983335	1.064544
ENSG00000118971	CCND2	0.01422304	1.00E-06	13.79594
ENSG00000104371	DKK4	0.962132	0.181007	2.410189
ENSG00000146122	DAAM2	0.08933887	0.037180426	1.264745
ENSG00000161202	DVL3	18.8828268	6.940652571	1.443932
ENSG00000157240	FZD1	3.011085	1.426115	1.078193
ENSG00000111432	FZD10	0.015508	1.00E-06	13.92073
ENSG00000188763	FZD9	0.430593	0.092593	2.21735
ENSG00000137673	MMP7	0.17527801	0.046595456	1.911384
ENSG00000137841	PLCB2	0.28891603	0.135825609	1.088895
ENSG00000163637	PRICKLE2	0.11534965	0.03014203	1.936165
ENSG00000165059	PRKACG	0.043653	0.013791	1.662354
ENSG00000137713	PPP2R1B	6.63731763	1.282050827	2.372147
ENSG00000188386	PPP3R2	0.015027	1.00E-06	13.87527
ENSG00000106483	SFRP4	0.06887742	1.00E-06	16.07174
ENSG00000081059	TCF7	3.76387506	0.94755206	1.989942
ENSG00000173218	VANGL1	6.47397394	2.740128994	1.240408
ENSG00000105989	WNT2	0.01693702	1.00E-06	14.04789
ENSG00000085741	WNT11	1.55528489	0.293031233	2.408053
ENSG00000108379	WNT3	1.25987372	0.456280774	1.465285
ENSG00000154342	WNT3A	1.469949	0.295337	2.315332
ENSG00000114251	WNT5A	0.07434077	0.01437267	2.370826
ENSG00000111186	WNT5B	0.24496369	0.075177837	1.704189
ENSG00000115596	WNT6	1.343684	0.62987	1.093068
ENSG00000061492	WNT8A	0.02436383	1.00E-06	14.57245
ENSG00000158955	WNT9B	0.0457805	0.008167879	2.4867

Supplementary Table 4.DJ-1 Up-regulated WNT signaling target genes

gene_id	gene_name	FPKM.DJ-1	FPKM.Vector	log2_DJ-1_Vector
ENSG00000172238	ATOH1	0.252181	1.00E-06	17.9441
ENSG00000104371	DKK4	0.962132	0.181007	2.410189
ENSG00000204335	SP5	0.789138557	0.157632504	2.323714
ENSG00000168646	AXIN2	8.771611573	1.776884799	2.303492
ENSG00000102678	FGF9	1.213276152	0.250392245	2.276646
ENSG00000125378	BMP4	168.7544117	37.64565179	2.16437
ENSG00000112081	SRSF3	40.57174451	9.071450386	2.16107
ENSG00000156427	FGF18	2.126614	0.484523	2.133921
ENSG00000118513	MYB	1.545501518	0.356369927	2.116628
ENSG00000081059	TCF7	3.763875059	0.94755206	1.989942
ENSG00000137673	MMP7	0.17527801	0.046595456	1.911384
ENSG00000171617	ENC1	18.14550806	5.767632692	1.653561
ENSG00000110092	CCND1	243.0298637	116.1983335	1.064544
ENSG00000089685	BIRC5	33.2689195	16.65374151	0.998329
ENSG00000168283	BMI1	28.06997238	14.33501366	0.969484
ENSG00000147889	CDKN2A	7.720489901	4.095436213	0.914675
ENSG00000137693	YAP1	13.31563971	7.082281794	0.910836
ENSG00000182580	EPHB3	5.160764071	2.809231322	0.877409
ENSG00000107984	DKK1	118.4902272	68.40097079	0.792679
ENSG00000115738	ID2	27.31138808	16.06671789	0.765427
ENSG00000130816	DNMT1	22.57308884	13.67770205	0.722778
ENSG00000133216	EPHB2	3.599913788	2.209408532	0.704302
ENSG00000178691	SUZ12	18.24546165	11.47805915	0.668659
ENSG00000196628	TCF4	0.007874903	0.005008902	0.652768
ENSG00000105976	MET	62.99932327	40.22476972	0.647252
ENSG00000157227	MMP14	8.42921086	5.416947883	0.637917
ENSG00000090776	EFNB1	14.896277	9.851439	0.596545

Supplementary Table 5. List of Small Interference RNA Sequences

Gene name	siRNA sense sequences
DJ-1_1	5'-GGUUCUACCAGGAGGUAAUTT-'3
DJ-1_2	5'-GACGGCCUGAUUCUUACAATT-'3
PLAGL2_1	5'-CCACCUACUGGGCUUUCUUTT-'3
PLAGL2_2	5'-GCAUGUAUGGUGCCCACAUTT-'3
BMP4_1	5'-CCGUCAUUCCGGACUACAUTT-'3
BMP4_2	5'-CCGUCAUUCCGGACUACAUTT-'3
CCND1	5'-CAAGCUCAAGUGGAACCUGTT-'3
NC	5'-UUCUCCGAACGUGUCACGUTT-'3

Supplementary Table 6. Primers for qRT-PCR

**Primers sequences for selected genes**	
Gene	Sequences
DJ-1	Forward 5'-GGAGACGGTCATCCCTGTAG-3'
	Reverse 5'-TTCACAGCAGCAGACTCAGA-3'
PLAGL2	Forward 5'-GTGCCAAGGAGAAGAAGCAC-3'
	Reverse 5'-CTAACATGTCCACGGGCTCT-3'
BMP4	Forward 5'-ACACGACTACTGGACACGAG-3'
	Reverse 5'-TCGGCTAATCCTGACATGCT-3'
GLI1	Forward 5'- CCCAATCACAAGTCAGGTTCCT-3'
	Reverse 5'- CCTATGTGAAGCCCTATTTGCC-3'
GLI2	Forward 5'- AGATTCTGAGCCAGCAGAGG-3'
	Reverse 5'- TGGTGTCACTCAGACAGTTGC-3'
PTCH1	Forward 5'- GGCAGCGGTAGTAGTGGTGTTC-3'
	Reverse 5'- TGTAGCGGGTATTGTCGTGTGTG-3'
TCF7	Forward 5'- CTGGCTTCTACTCCCTGACCT-3'
	Reverse 5'- ACCAGAACCTAGCATCAAGGA-3'
CCND1	Forward 5'- GACGGCCGAGAAGCTGTGCA-3'
	Reverse 5'- GCCACCATGGAGGGCGGATT-3′
FGF9	Forward 5'-GTGGACTCTACCTCGGGATG-3'
	Reverse 5'-GTGTGAATTTCTGGTGCCGT-3'
AXIN2	Forward 5'- GACAGGAATCATTCGGCCAC-3'
	Reverse 5'- CCTTCAGCATCCTCCGGTAT-3'
