# Supplementary material for: DJ-1 promotes colorectal cancer progression through activating PLAGL2/Wnt/BMP4 axis
Source: Cell Death Dis. 2018 Aug 29;9(9):865. doi: 10.1038/s41419-018-0883-4 (PMC6115399; doi:10.1038/s41419-018-0883-4)
Supplement: Supplementary file 3 — Supplementary Table 2 [file 41419_2018_883_MOESM3_ESM.pdf]

Supplementary Table 2. Gene features significantly differentially expressed in HCT116 cells transfected with DJ-1 compared to cells transfected with control vector

gene_id	gene_name	FPKM.DJ-1	FPKM.Vector	log2_DJ-1_Vector
ENSG00000198888	MT-ND1	3157.402832	1.00E-06	31.55609119
ENSG00000198763	MT-ND2	204.203659	1.00E-06	27.60543348
ENSG00000210117	MT-TW	60.214703	1.00E-06	25.84361247
ENSG00000210112	MT-TM	56.68663	1.00E-06	25.75650517
ENSG00000240409	MTATP8P1	8.315901	1.00E-06	22.98744115
ENSG00000254911	SCARNA9	5.418355	1.00E-06	22.36942349
ENSG00000266642	RP11-20B24.4	3.979084717	1.00E-06	21.92400518
ENSG00000260371	RP11-343C2.9	3.728373	1.00E-06	21.83011477
ENSG00000224818	RP11-134G8.10	3.694682	1.00E-06	21.81701877
ENSG00000242358	RPS21P4	3.390344	1.00E-06	21.69300023
ENSG00000273154	RP4-583P15.15	3.380023877	1.00E-06	21.68860201
ENSG00000259040	BLOC1S5-TXNDC5	3.375074	1.00E-06	21.6864877
ENSG00000255138	GLTPP1	3.025406	1.00E-06	21.52869733
ENSG00000235288	RP11-70C1.3	3.017801754	1.00E-06	21.5250666
ENSG00000259900	RP11-343C2.7	2.899420552	1.00E-06	21.46733318
ENSG00000232901	CYCSP10	2.731775	1.00E-06	21.38140723
ENSG00000093100	XXbac-B461K10.4	2.590369	1.00E-06	21.30472619
ENSG00000124208	TMEM189-UBE2V1	2.578387	1.00E-06	21.29803739
ENSG00000198211	RP11-566K11.2	2.417639	1.00E-06	21.20516741
ENSG00000274816	MIR6772	2.41757	1.00E-06	21.20512623
ENSG00000232369	RP5-882O7.4	2.394884	1.00E-06	21.19152435
ENSG00000279592	RP11-417N10.4	2.333977	1.00E-06	21.15435891
ENSG00000260272	RP11-20I23.1	2.227654	1.00E-06	21.08709374
ENSG00000252585	Y_RNA	2.161963	1.00E-06	21.0439104
ENSG00000100101	RP1-37E16.12	2.149182	1.00E-06	21.03535623
ENSG00000261915	RP11-542C16.2	2.130641	1.00E-06	21.0228561
ENSG00000259399	TGIF2-C20orf24	2.1257	1.00E-06	21.01950657
ENSG00000207081	RNU6-616P	2.102442	1.00E-06	21.00363457
ENSG00000265806	MIR4292	2.096619	1.00E-06	20.99963329
ENSG00000267952	CTD-2207O23.12	1.982704278	1.00E-06	20.91903808
ENSG00000201405	Y_RNA	1.893558	1.00E-06	20.85266818
ENSG00000275719	CTB-147N14.6	1.857085	1.00E-06	20.82460842
ENSG00000259288	BUB1B-PAK6	1.854450781	1.00E-06	20.82256055
ENSG00000261011	RP11-96C23.11	1.851504	1.00E-06	20.82026624
ENSG00000235681	AC008746.5	1.788372	1.00E-06	20.77021543
ENSG00000248993	XXbac-BPG181M17.5	1.734013	1.00E-06	20.72568328
ENSG00000255959	RP11-804A23.2	1.731245	1.00E-06	20.72337847
ENSG00000228989	AC133528.2	1.720551	1.00E-06	20.71443923
ENSG00000250562	RPL38P4	1.686873	1.00E-06	20.68591993
ENSG00000274396	HOTAIRM1_4	1.642329	1.00E-06	20.64731173
ENSG00000270757	HSPE1-MOB4	1.616644	1.00E-06	20.62457059

ENSG00000272566	RP11-462G22.2	1.603648	1.00E-06	20.61292607
ENSG00000267500	ZNF887P	1.583024	1.00E-06	20.5942517
ENSG00000228952	RP11-567G11.1	1.559989	1.00E-06	20.57310443
ENSG00000199466	Y_RNA	1.556825	1.00E-06	20.57017535
ENSG00000273027	LL21NC02-1C16.1	1.533525	1.00E-06	20.54842026
ENSG00000265575	MIR4784	1.513666	1.00E-06	20.52961547
ENSG00000281415	AC021066.1	1.461831	1.00E-06	20.4793451
ENSG00000213601	KRT18P19	1.451701	1.00E-06	20.46931291
ENSG00000271749	RP11-269G24.7	1.447909	1.00E-06	20.4655395
ENSG00000149300	C11orf52	1.444321014	1.00E-06	20.46196
ENSG00000251868	RNU7-71P	1.44206	1.00E-06	20.45969976
ENSG00000273432	RP5-1165K10.2	1.437645	1.00E-06	20.45527604
ENSG00000274066	MIR6514	1.372392	1.00E-06	20.38826119
ENSG00000272617	RP11-343C2.12	1.327284	1.00E-06	20.34004567
ENSG00000206964	Y_RNA	1.32055	1.00E-06	20.3327075
ENSG00000213997	PGAM1P7	1.30717	1.00E-06	20.31801535
ENSG00000251821	RNU6-583P	1.294534	1.00E-06	20.30400143
ENSG00000233903	Z83851.4	1.281277	1.00E-06	20.28915098
ENSG00000238578	SNORD4A	1.27584	1.00E-06	20.28301598
ENSG00000278571	MIR7161	1.272405	1.00E-06	20.27912652
ENSG00000277971	XXbac-B562F10.12	1.262475	1.00E-06	20.26782339
ENSG00000236654	AC079780.3	1.26134	1.00E-06	20.26652578
ENSG00000273555	MIR6812	1.2565	1.00E-06	20.26097924
ENSG00000281599	AC023283.1	1.232224	1.00E-06	20.23283311
ENSG00000206808	Y_RNA	1.175239	1.00E-06	20.16452275
ENSG00000275010	MIR6834	1.171193	1.00E-06	20.15954741
ENSG00000260274	RP11-817O13.8	1.161988	1.00E-06	20.14816374
ENSG00000279837	RP11-504G3.2	1.153117	1.00E-06	20.13710747
ENSG00000276629	AL160165.1	1.151223	1.00E-06	20.13473589
ENSG00000269403	CTD-2616J11.11	1.135607	1.00E-06	20.11503222
ENSG00000278223	MIR6783	1.130813	1.00E-06	20.10892894
ENSG00000281435	Z98744.1	1.128569	1.00E-06	20.1060632
ENSG00000255439	RP11-196G11.1	1.110786487	1.00E-06	20.0831501
ENSG00000278815	Vault	1.10863	1.00E-06	20.08034652
ENSG00000256206	RP11-140L24.4	1.104988	1.00E-06	20.07559927
ENSG00000273026	RP11-422P24.10	1.096414	1.00E-06	20.06436122
ENSG00000280333	RP11-667K14.14	1.086292	1.00E-06	20.05098053
ENSG00000276131	RP11-481J2.3	1.085538	1.00E-06	20.0499788
ENSG00000200769	Y_RNA	1.08431	1.00E-06	20.04834585
ENSG00000261487	AC135048.13	1.076472	1.00E-06	20.03787936
ENSG00000281867	AC018463.1	1.055625	1.00E-06	20.00966599
ENSG00000259760	RP11-20G13.2	1.034772	1.00E-06	19.98088149
ENSG00000199102	MIR302C	1.017924	1.00E-06	19.95719842
ENSG00000280834	U6	1.017924	1.00E-06	19.95719842
ENSG00000268465	CTC-273B12.7	1.011029	1.00E-06	19.94739295
ENSG00000272711	RP11-259N19.1	1.010288	1.00E-06	19.94633519
ENSG00000180425	C11orf71	1.010205468	1.00E-06	19.94621733
ENSG00000215976	AC078819.1	1.007095	1.00E-06	19.94176835

ENSG00000280502	Metazoa_SRP	0.99949	1.00E-06	19.93083261
ENSG00000251819	RNU6-322P	0.978393	1.00E-06	19.90005456
ENSG00000265982	RP11-927P21.4	0.97551	1.00E-06	19.89579714
ENSG00000251985	RNU6-1161P	0.968026	1.00E-06	19.88468627
ENSG00000276925	RP11-708J19.3	0.964857	1.00E-06	19.87995561
ENSG00000207865	MIR34A	0.962402	1.00E-06	19.87628012
ENSG00000277810	AL357075.1	0.952252	1.00E-06	19.86098389
ENSG00000238490	Y_RNA	0.936851	1.00E-06	19.83746009
ENSG00000234789	RP11-483H20.4	0.935787	1.00E-06	19.83582066
ENSG00000274151	AC018737.1	0.933875	1.00E-06	19.83286993
ENSG00000211868	TRAJ21	0.925386	1.00E-06	19.81969575
ENSG00000221051	AC118344.1	0.890684	1.00E-06	19.76455415
ENSG00000262304	RP11-235E17.2	0.889827	1.00E-06	19.76316535
ENSG00000206728	Y_RNA	0.871108	1.00E-06	19.73249207
ENSG00000252412	Y_RNA	0.867915	1.00E-06	19.72719423
ENSG00000200732	RNU6-194P	0.862812	1.00E-06	19.71868672
ENSG00000263675	MIR5581	0.84827	1.00E-06	19.69416401
ENSG00000199899	Y_RNA	0.843129	1.00E-06	19.68539386
ENSG00000264357	MIR4648	0.834132	1.00E-06	19.66991618
ENSG00000212175	SNORA12	0.832222	1.00E-06	19.6666089
ENSG00000277446	Metazoa_SRP	0.824034	1.00E-06	19.65234434
ENSG00000265917	MIR3685	0.820907	1.00E-06	19.64685926
ENSG00000251787	RNU7-47P	0.820907	1.00E-06	19.64685926
ENSG00000251988	RNU4ATAC18P	0.807877	1.00E-06	19.62377613
ENSG00000266431	MIR5580	0.807319	1.00E-06	19.62277932
ENSG00000207233	SNORA37	0.789089	1.00E-06	19.5898285
ENSG00000275107	MIR6782	0.781884	1.00E-06	19.57659506
ENSG00000279507	RP11-723O4.7	0.781302	1.00E-06	19.57552078
ENSG00000238731	RNU7-90P	0.771653	1.00E-06	19.55759271
ENSG00000274915	HAR1A	0.768483	1.00E-06	19.55165382
ENSG00000272186	RP11-110I1.13	0.764226	1.00E-06	19.54363982
ENSG00000254694	RP11-50B3.4	0.764111	1.00E-06	19.5434227
ENSG00000252489	RNU6-197P	0.763443	1.00E-06	19.54216092
ENSG00000265285	AL591379.1	0.759645	1.00E-06	19.53496585
ENSG00000238391	RNA5SP233	0.756687	1.00E-06	19.52933713
ENSG00000269980	RP11-282O18.7	0.741927	1.00E-06	19.50091772
ENSG00000252145	RNU6-1225P	0.736038	1.00E-06	19.48942073
ENSG00000282883	AKR1C3	0.733119	1.00E-06	19.48368787
ENSG00000250349	RP5-972B16.2	0.729976	1.00E-06	19.47748951
ENSG00000260955	RP11-30L15.6	0.724115	1.00E-06	19.46585931
ENSG00000207875	MIRLET7B	0.723584	1.00E-06	19.46480098
ENSG00000272346	RP11-927P21.11	0.721029	1.00E-06	19.45969776
ENSG00000279413	AC112497.1	0.717132	1.00E-06	19.45187917
ENSG00000240494	RPS12P28	0.715169	1.00E-06	19.44792468
ENSG00000267618	RAD51L3-RFFL	0.714767636	1.00E-06	19.44711479
ENSG00000252556	RNU6-256P	0.713596	1.00E-06	19.444748
ENSG00000280919	AC108667.1	0.712547	1.00E-06	19.44262565
ENSG00000243104	MTND4LP14	0.706891	1.00E-06	19.43112825

ENSG00000221120	MIR1224	0.702914	1.00E-06	19.42298866
ENSG00000273627	MIR4726	0.702016	1.00E-06	19.42114439
ENSG00000267157	CTB-54O9.9	0.701486	1.00E-06	19.42005479
ENSG00000272934	RP11-392E22.10	0.701418	1.00E-06	19.41991493
ENSG00000252139	SCARNA18	0.699056	1.00E-06	19.41504851
ENSG00000260193	RP11-83N9.5	0.698200706	1.00E-06	19.41328229
ENSG00000207980	MIR23A	0.697209	1.00E-06	19.41123167
ENSG00000249141	RP11-514O12.4	0.696201	1.00E-06	19.40914436
ENSG00000236431	AC009237.11	0.684883	1.00E-06	19.38549802
ENSG00000251660	AC007036.5	0.682169	1.00E-06	19.37976967
ENSG00000278939	AC242988.1	0.678616	1.00E-06	19.37223592
ENSG00000254462	TMX2-CTNND1	0.676197	1.00E-06	19.36708409
ENSG00000199325	RNU4-39P	0.673272	1.00E-06	19.36082994
ENSG00000223837	BRD2-IT1	0.673258	1.00E-06	19.36079994
ENSG00000265300	AC007731.2	0.672557	1.00E-06	19.35929702
ENSG00000270100	RP11-130L8.1	0.67162	1.00E-06	19.35728567
ENSG00000264961	MIR4730	0.669687	1.00E-06	19.35312744
ENSG00000212242	RNA5SP219	0.666605	1.00E-06	19.34647261
ENSG00000200403	RNU6-1099P	0.665932	1.00E-06	19.34501534
ENSG00000275833	RP11-479O9.3	0.66585	1.00E-06	19.34483768
ENSG00000212371	SNORA46	0.66531	1.00E-06	19.34366719
ENSG00000273996	AC104946.1	0.662459	1.00E-06	19.33747164
ENSG00000155622	XAGE2	0.657203	1.00E-06	19.32597954
ENSG00000276741	MIR6869	0.654618	1.00E-06	19.32029375
ENSG00000268714	CTD-2287O16.3	0.652424	1.00E-06	19.31545033
ENSG00000263448	AL133410.1	0.651471	1.00E-06	19.31334143
ENSG00000221055	MIR1302-3	0.650164	1.00E-06	19.31044415
ENSG00000258659	TRIM34	0.647683567	1.00E-06	19.30492961
ENSG00000224409	RP11-114B7.6	0.644255	1.00E-06	19.2972723
ENSG00000274427	RP11-972P1.10	0.643123	1.00E-06	19.29473516
ENSG00000234567	RP1-283K11.2	0.639012	1.00E-06	19.2854835
ENSG00000270091	RP11-78O7.2	0.635838383	1.00E-06	19.27830058
ENSG00000207359	RNU6-925P	0.626415	1.00E-06	19.25675923
ENSG00000264203	AC092377.1	0.625297	1.00E-06	19.25418207
ENSG00000221585	MIR1226	0.624326	1.00E-06	19.25194002
ENSG00000254760	CTD-2616J11.3	0.620685	1.00E-06	19.24350176
ENSG00000230758	SNAP23P	0.620685	1.00E-06	19.24350176
ENSG00000223831	RP1-56J10.8	0.614967	1.00E-06	19.23014947
ENSG00000237133	AC020594.5	0.614962	1.00E-06	19.23013774
ENSG00000274292	RP11-347I19.7	0.614576	1.00E-06	19.2292319
ENSG00000273620	AL591856.1	0.613208	1.00E-06	19.22601699
ENSG00000256100	AP000721.4	0.610934	1.00E-06	19.22065701
ENSG00000234617	SNRK-AS1	0.608442	1.00E-06	19.21476022
ENSG00000267277	CTD-2342J14.6	0.606344	1.00E-06	19.20977699
ENSG00000199313	RNU4-82P	0.603483	1.00E-06	19.20295361
ENSG00000277209	RPPH1	0.602196	1.00E-06	19.1998736
ENSG00000280744	LINC01173	0.599684	1.00E-06	19.19384296
ENSG00000277050	RP11-102G14.1	0.593789	1.00E-06	19.17959084

ENSG00000278136	AL390065.1	0.591816	1.00E-06	19.17478918
ENSG00000231858	AC067945.4	0.59180975	1.00E-06	19.17477394
ENSG00000221296	MIR548T	0.591496	1.00E-06	19.17400889
ENSG00000222650	RNU2-70P	0.59142	1.00E-06	19.17382351
ENSG00000198553	KCNRG	0.590709078	1.00E-06	19.17208826
ENSG00000261759	RP11-626G11.3	0.590553	1.00E-06	19.17170702
ENSG00000268108	CTB-60B18.12	0.587264	1.00E-06	19.16364968
ENSG00000221033	MIR1272	0.583925	1.00E-06	19.15542355
ENSG00000206903	SNORA24	0.582781	1.00E-06	19.15259432
ENSG00000100583	SAMD15	0.582115231	1.00E-06	19.15094524
ENSG00000251050	RP11-168A11.4	0.580739	1.00E-06	19.1475304
ENSG00000266583	MIR4478	0.57998	1.00E-06	19.14564363
ENSG00000240925	RPS20P31	0.579481	1.00E-06	19.14440183
ENSG00000275656	RFPL3-AS1_1	0.578366	1.00E-06	19.14162322
ENSG00000216182	AL137024.1	0.578366	1.00E-06	19.14162322
ENSG00000276028	AL592183.6	0.576415	1.00E-06	19.13674835
ENSG00000207737	MIR181B2	0.571868	1.00E-06	19.12532265
ENSG00000274140	MIR6073	0.571867	1.00E-06	19.12532013
ENSG00000269949	RP11-738E22.3	0.569309	1.00E-06	19.11885238
ENSG00000278642	RP11-159D12.11	0.568672	1.00E-06	19.11723725
ENSG00000263388	CTC-297N7.10	0.567265	1.00E-06	19.11366333
ENSG00000266235	MIR3176	0.565514	1.00E-06	19.10920321
ENSG00000200714	Y_RNA	0.565514	1.00E-06	19.10920321
ENSG00000254812	RP11-661A12.12	0.564142	1.00E-06	19.10569882
ENSG00000211643	IGLV5-52	0.563089	1.00E-06	19.10300344
ENSG00000266799	AP003356.1	0.562852	1.00E-06	19.10239609
ENSG00000269921	RP11-646I6.5	0.559955	1.00E-06	19.09495137
ENSG00000252367	Y_RNA	0.559299	1.00E-06	19.09326023
ENSG00000277411	5S_rRNA	0.556978	1.00E-06	19.08726082
ENSG00000270917	RP11-27I1.6	0.556978	1.00E-06	19.08726082
ENSG00000276048	Six3os1_5	0.555914	1.00E-06	19.08450219
ENSG00000212615	SNORD58	0.555232	1.00E-06	19.08273119
ENSG00000276083	MIR7976	0.555232	1.00E-06	19.08273119
ENSG00000253063	RNU6-494P	0.554203	1.00E-06	19.08005499
ENSG00000207449	RNU6-19P	0.553674	1.00E-06	19.07867725
ENSG00000265450	MIR4502	0.552947	1.00E-06	19.07678168
ENSG00000184378	ACTRT3	0.550926	1.00E-06	19.07149902
ENSG00000268400	CTD-3214H19.4	0.549586643	1.00E-06	19.06798742
ENSG00000265530	AL121594.1	0.548113	1.00E-06	19.06411383
ENSG00000280717	AC009121.2	0.548113	1.00E-06	19.06411383
ENSG00000275036	MIR8086	0.547271	1.00E-06	19.06189588
ENSG00000261779	RP11-69H7.3	0.546909	1.00E-06	19.06094128
ENSG00000221540	MIR1180	0.545843	1.00E-06	19.05812653
ENSG00000233313	HMGA1P5	0.544645	1.00E-06	19.05495666
ENSG00000272308	RP11-231G3.1	0.544026	1.00E-06	19.05331608
ENSG00000269038	AP001462.6	0.542743	1.00E-06	19.04990969
ENSG00000229299	RP4-583P15.10	0.542044603	1.00E-06	19.04805205
ENSG00000262050	RP11-74E22.3	0.541524	1.00E-06	19.04666575

ENSG00000229716	RPL23AP19	0.540457	1.00E-06	19.04382031
ENSG00000198976	MIR429	0.539622	1.00E-06	19.04158964
ENSG00000277329	AC091114.1	0.538213	1.00E-06	19.03781771
ENSG00000222197	AL592466.1	0.537556	1.00E-06	19.03605553
ENSG00000201555	Y_RNA	0.537556	1.00E-06	19.03605553
ENSG00000265095	FTLP12	0.537301	1.00E-06	19.035371
ENSG00000278350	AC091849.1	0.535987	1.00E-06	19.03183848
ENSG00000281838	AC017028.12	0.535063	1.00E-06	19.02934924
ENSG00000264653	MIR5194	0.53441	1.00E-06	19.02758748
ENSG00000273366	CTA-989H11.1	0.531359	1.00E-06	19.01932739
ENSG00000264005	MIR4314	0.531091	1.00E-06	19.01859956
ENSG00000206969	RNU6-1316P	0.530169	1.00E-06	19.01609279
ENSG00000201118	Y_RNA	0.530169	1.00E-06	19.01609279
ENSG00000252153	MIR2278	0.530169	1.00E-06	19.01609279
ENSG00000239353	RP11-492E3.51	0.528648	1.00E-06	19.0119479
ENSG00000274902	RP1-197B17.4	0.527303	1.00E-06	19.00827268
ENSG00000272004	RP11-345P4.10	0.526156	1.00E-06	19.00513108
ENSG00000278244	UPF3BP3	0.52512	1.00E-06	19.00228762
ENSG00000279768	AC008914.1	0.524901	1.00E-06	19.00168582
ENSG00000252972	RNA5SP398	0.524703	1.00E-06	19.00114151
ENSG00000256632	RP13-672B3.2	0.524419	1.00E-06	19.00036043
ENSG00000278491	AL360074.1	0.523477	1.00E-06	18.99776663
ENSG00000005102	MEOX1	0.523063	1.00E-06	18.9966252
ENSG00000266555	MIR3145	0.521376	1.00E-06	18.99196465
ENSG00000228153	RP11-23I7.1	0.520482	1.00E-06	18.98948875
ENSG00000252782	RNU6-341P	0.519566	1.00E-06	18.9869475
ENSG00000231366	RP11-399N22.3	0.519349	1.00E-06	18.98634482
ENSG00000266621	AC104841.1	0.519349	1.00E-06	18.98634482
ENSG00000281372	AC108485.1	0.519349	1.00E-06	18.98634482
ENSG00000206717	Y_RNA	0.519349	1.00E-06	18.98634482
ENSG00000277992	AL139383.1	0.514103	1.00E-06	18.97169791
ENSG00000265959	AL354720.1	0.514103	1.00E-06	18.97169791
ENSG00000255054	RP1-317E23.6	0.511881146	1.00E-06	18.96544934
ENSG00000234076	TPRG1-AS1	0.511153	1.00E-06	18.96339566
ENSG00000176125	UFSP1	0.511006	1.00E-06	18.96298071
ENSG00000235493	AC092415.1	0.510192412	1.00E-06	18.96068192
ENSG00000221550	AL645859.1	0.508962	1.00E-06	18.95719842
ENSG00000281172	AL139156.1	0.508962	1.00E-06	18.95719842
ENSG00000253054	RNU7-77P	0.508962	1.00E-06	18.95719842
ENSG00000199740	Y_RNA	0.508962	1.00E-06	18.95719842
ENSG00000221347	AC096649.4	0.508962	1.00E-06	18.95719842
ENSG00000264810	MIR4441	0.508962	1.00E-06	18.95719842
ENSG00000242001	GS1-388B5.1	0.508962	1.00E-06	18.95719842
ENSG00000264031	ABHD15-AS1	0.50624	1.00E-06	18.94946198
ENSG00000280608	AL137229.1	0.503923	1.00E-06	18.94284378
ENSG00000200164	Y_RNA	0.503923	1.00E-06	18.94284378
ENSG00000264687	AL050335.1	0.503923	1.00E-06	18.94284378
ENSG00000266272	Z97632.1	0.503923	1.00E-06	18.94284378

ENSG00000170373	CST1	0.501958211	1.00E-06	18.93720774
ENSG00000222248	RNA5SP201	0.500753	1.00E-06	18.93373963
ENSG00000186919	ZACN	0.500253526	1.00E-06	18.93229991
ENSG00000201134	Y_RNA	0.498982	1.00E-06	18.92862825
ENSG00000199291	Y_RNA	0.498982	1.00E-06	18.92862825
ENSG00000206995	Y_RNA	0.498982	1.00E-06	18.92862825
ENSG00000221044	U3	0.498982	1.00E-06	18.92862825
ENSG00000201501	Y_RNA	0.498982	1.00E-06	18.92862825
ENSG00000274472	Y_RNA	0.498982	1.00E-06	18.92862825
ENSG00000252864	RNA5SP278	0.498982	1.00E-06	18.92862825
ENSG00000225235	DDX26B-AS1	0.498732	1.00E-06	18.92790525
ENSG00000271659	RP11-435O5.4	0.49827	1.00E-06	18.92656819
ENSG00000222651	MIR1469	0.498133	1.00E-06	18.92617146
ENSG00000263363	MIR5702	0.496844	1.00E-06	18.92243342
ENSG00000181585	TMIE	0.49501	1.00E-06	18.91709814
ENSG00000252494	RNU6-126P	0.494138	1.00E-06	18.91455448
ENSG00000251374	RPS23P5	0.494138	1.00E-06	18.91455448
ENSG00000202431	RNU6-438P	0.494138	1.00E-06	18.91455448
ENSG00000199226	RNU6-50P	0.494138	1.00E-06	18.91455448
ENSG00000266877	RP1-41C23.1	0.493567	1.00E-06	18.91288641
ENSG00000236024	PRRX2-AS1	0.493538	1.00E-06	18.91280164
ENSG00000272267	RP11-375N15.2	0.493127	1.00E-06	18.91159972
ENSG00000266688	AC079398.1	0.492544	1.00E-06	18.90989308
ENSG00000228702	RP11-671E7.1	0.491751	1.00E-06	18.90756846
ENSG00000272791	RP11-464F9.22	0.48991	1.00E-06	18.90215721
ENSG00000200105	RNU6-251P	0.489386	1.00E-06	18.9006133
ENSG00000252659	RNU6-1088P	0.489386	1.00E-06	18.9006133
ENSG00000238295	snoU13	0.489386	1.00E-06	18.9006133
ENSG00000264134	AL121893.1	0.489132	1.00E-06	18.89986433
ENSG00000252779	RNU6-182P	0.484725	1.00E-06	18.88680697
ENSG00000222610	RNU6-402P	0.484255	1.00E-06	18.88540742
ENSG00000280607	AC009133.24	0.480426	1.00E-06	18.8739547
ENSG00000276931	RP11-161M6.6	0.480243	1.00E-06	18.87340506
ENSG00000239823	Y_RNA	0.480153	1.00E-06	18.87313467
ENSG00000251986	Y_RNA	0.477801	1.00E-06	18.86605035
ENSG00000207047	SNORD51	0.47744	1.00E-06	18.86495992
ENSG00000273294	C1QTNF3-AMACR	0.476646	1.00E-06	18.86255866
ENSG00000222374	AC022021.1	0.475666	1.00E-06	18.85958938
ENSG00000206924	RNU6-689P	0.475666	1.00E-06	18.85958938
ENSG00000251939	RNU6-1278P	0.475666	1.00E-06	18.85958938
ENSG00000202159	RNU6-742P	0.475666	1.00E-06	18.85958938
ENSG00000206759	RNU6-787P	0.475666	1.00E-06	18.85958938
ENSG00000201744	RNU6-34P	0.475666	1.00E-06	18.85958938
ENSG00000277192	RP11-89G4.1	0.475666	1.00E-06	18.85958938
ENSG00000207459	RNU6-1311P	0.475666	1.00E-06	18.85958938
ENSG00000199601	RNU6-562P	0.475666	1.00E-06	18.85958938
ENSG00000279900	RP11-113K21.6	0.475181	1.00E-06	18.85811763
ENSG00000252186	RNU6-781P	0.473688	1.00E-06	18.8535776

ENSG00000276292	RP11-33N14.5	0.47312	1.00E-06	18.85184662
ENSG00000250327	RPSAP70	0.472204	1.00E-06	18.84905074
ENSG00000281710	U3	0.471261	1.00E-06	18.84616677
ENSG00000231255	AC005009.1	0.471121	1.00E-06	18.84573812
ENSG00000206695	RNU6-442P	0.469811	1.00E-06	18.84172097
ENSG00000221783	MIR1183	0.468931	1.00E-06	18.83901613
ENSG00000273672	AC022395.1	0.466938	1.00E-06	18.83287148
ENSG00000252608	RNU6-1191P	0.466152	1.00E-06	18.83044093
ENSG00000252464	RN7SKP70	0.464514	1.00E-06	18.82536255
ENSG00000271537	RP11-365F18.6	0.463798	1.00E-06	18.82313707
ENSG00000225155	TOMM22P5	0.463747	1.00E-06	18.82297842
ENSG00000269976	RP11-130L8.2	0.463747	1.00E-06	18.82297842
ENSG00000204805	FAM27E4	0.463324	1.00E-06	18.82166189
ENSG00000260247	SUB1P4	0.462834	1.00E-06	18.82013532
ENSG00000252750	RNU7-70P	0.462692	1.00E-06	18.81969263
ENSG00000200953	Y_RNA	0.462692	1.00E-06	18.81969263
ENSG00000222747	RNA5SP25	0.462692	1.00E-06	18.81969263
ENSG00000276751	AC011498.1	0.462692	1.00E-06	18.81969263
ENSG00000273657	MIR6792	0.462544	1.00E-06	18.81923108
ENSG00000230478	RP1-52D1.1	0.461836	1.00E-06	18.81702111
ENSG00000212379	RNU6-269P	0.461739	1.00E-06	18.81671807
ENSG00000260934	CTA-363E6.7	0.46006	1.00E-06	18.8114625
ENSG00000199890	Y_RNA	0.458524	1.00E-06	18.80663772
ENSG00000267291	CTB-186G2.1	0.456968	1.00E-06	18.80173362
ENSG00000270133	CTC-303L1.2	0.456925	1.00E-06	18.80159785
ENSG00000276135	FAM27E2	0.456339	1.00E-06	18.79974643
ENSG00000176043	RP1-146I3.1	0.456311	1.00E-06	18.79965791
ENSG00000278592	HOXA11-AS1_5	0.456311	1.00E-06	18.79965791
ENSG00000221729	AC233287.1	0.455946	1.00E-06	18.79850344
ENSG00000199472	Y_RNA	0.454431	1.00E-06	18.79370173
ENSG00000199668	Y_RNA	0.454431	1.00E-06	18.79370173
ENSG00000266712	AC090810.1	0.454431	1.00E-06	18.79370173
ENSG00000270048	RP11-214K3.22	0.452841	1.00E-06	18.78864506
ENSG00000252845	RNA5SP101	0.45241	1.00E-06	18.78727129
ENSG00000263441	AC012181.1	0.451245	1.00E-06	18.78355142
ENSG00000234022	AC008278.2	0.450475	1.00E-06	18.78108752
ENSG00000207473	Y_RNA	0.450409	1.00E-06	18.78087613
ENSG00000281672	AC124276.1	0.450409	1.00E-06	18.78087613
ENSG00000202522	Y_RNA	0.450409	1.00E-06	18.78087613
ENSG00000199411	SNORD62	0.44978	1.00E-06	18.77885999
ENSG00000260541	LA16c-429E7.1	0.448812	1.00E-06	18.77575172
ENSG00000243650	RN7SL834P	0.448456	1.00E-06	18.77460692
ENSG00000235204	RP11-121A14.2	0.44782	1.00E-06	18.77255944
ENSG00000207004	RNU6-301P	0.447125	1.00E-06	18.77031869
ENSG00000248839	RP11-227H4.5	0.445597	1.00E-06	18.76537999
ENSG00000253084	RNU6-840P	0.445342	1.00E-06	18.76455415
ENSG00000251952	RNU6-1219P	0.445342	1.00E-06	18.76455415
ENSG00000266620	MIR5001	0.444283	1.00E-06	18.76111941

ENSG00000277678	U1	0.443889	1.00E-06	18.75983943
ENSG00000281554	Y_RNA	0.442576	1.00E-06	18.75556569
ENSG00000223191	RNU6-293P	0.44174	1.00E-06	18.75283795
ENSG00000227857	RP4-533D7.5	0.4414	1.00E-06	18.7517271
ENSG00000277202	MIR8063	0.439844	1.00E-06	18.74663241
ENSG00000252019	RNU6ATAC9P	0.43876	1.00E-06	18.74307248
ENSG00000270861	KB-1589B1.4	0.436878	1.00E-06	18.73687093
ENSG00000277311	Six3os1_2	0.436253	1.00E-06	18.73480553
ENSG00000238906	snoU13	0.436253	1.00E-06	18.73480553
ENSG00000200293	RNA5SP393	0.43501	1.00E-06	18.73068904
ENSG00000240027	RP11-415I12.1	0.433117	1.00E-06	18.72439727
ENSG00000223968	AC098614.1	0.433117	1.00E-06	18.72439727
ENSG00000200376	RNU5E-10P	0.431324	1.00E-06	18.71841247
ENSG00000270296	STX8P1	0.430109	1.00E-06	18.71434279
ENSG00000222282	RNU6-584P	0.4286	1.00E-06	18.70927232
ENSG00000201363	Y_RNA	0.4286	1.00E-06	18.70927232
ENSG00000266320	MIR3909	0.427699	1.00E-06	18.70623631
ENSG00000230064	RP11-244N20.7	0.427699	1.00E-06	18.70623631
ENSG00000200571	RNU6-1284P	0.427528	1.00E-06	18.70565938
ENSG00000239093	snoU13	0.426559	1.00E-06	18.70238578
ENSG00000265442	MIR3941	0.424959	1.00E-06	18.69696413
ENSG00000202310	Y_RNA	0.424135	1.00E-06	18.69416401
ENSG00000240723	RN7SL382P	0.422331	1.00E-06	18.68801462
ENSG00000221774	AC016821.1	0.42121	1.00E-06	18.68418016
ENSG00000239096	snoU13	0.420873	1.00E-06	18.68302543
ENSG00000252643	RNU6-1136P	0.420873	1.00E-06	18.68302543
ENSG00000207361	RNU6-178P	0.420873	1.00E-06	18.68302543
ENSG00000277602	AC005363.11	0.42063	1.00E-06	18.68219222
ENSG00000230939	RP11-314C16.1	0.416329	1.00E-06	18.66736453
ENSG00000267030	CTB-50L17.7	0.413945	1.00E-06	18.65907957
ENSG00000265713	RP11-82O19.2	0.413565	1.00E-06	18.65775457
ENSG00000266457	AC026318.1	0.413217	1.00E-06	18.65654008
ENSG00000200241	Y_RNA	0.412672	1.00E-06	18.65463603
ENSG00000207452	RNU6-606P	0.412672	1.00E-06	18.65463603
ENSG00000248774	RP11-798M19.3	0.412375	1.00E-06	18.65359735
ENSG00000251508	RP5-862P8.3	0.409997	1.00E-06	18.64525383
ENSG00000266750	MIR4645	0.409813	1.00E-06	18.64460622
ENSG00000151963	ZNF37CP	0.409704	1.00E-06	18.64422245
ENSG00000212298	RNU6-1009P	0.409072	1.00E-06	18.64199527
ENSG00000276878	MIR6074	0.409072	1.00E-06	18.64199527
ENSG00000229497	AC005189.6	0.408812	1.00E-06	18.64107802
ENSG00000274237	CU459211.1	0.408268	1.00E-06	18.63915697
ENSG00000281678	MIR6516	0.40753	1.00E-06	18.63654674
ENSG00000221105	AL159997.1	0.40717	1.00E-06	18.63527174
ENSG00000259408	RP11-3D4.3	0.406537	1.00E-06	18.63302714
ENSG00000257000	RP13-820C6.2	0.405914	1.00E-06	18.63081457
ENSG00000266083	AC138965.1	0.40498	1.00E-06	18.62749114
ENSG00000274988	MIR6876	0.404381	1.00E-06	18.62535569

ENSG00000225294	OSTCP2	0.401209	1.00E-06	18.61399444
ENSG00000275955	AC108479.1	0.400758	1.00E-06	18.61237179
ENSG00000279063	CTD-2537I9.15	0.399387	1.00E-06	18.60742785
ENSG00000266463	MIR3196	0.397627	1.00E-06	18.6010562
ENSG00000224883	CTA-250D10.19	0.395311	1.00E-06	18.59262857
ENSG00000259369	RP11-291H24.1	0.394544	1.00E-06	18.58982667
ENSG00000144410	CPO	0.394035	1.00E-06	18.58796426
ENSG00000202237	RNU6-53P	0.393725	1.00E-06	18.5868288
ENSG00000265771	AL592205.2	0.393484	1.00E-06	18.58594545
ENSG00000202099	RNU6-234P	0.391509	1.00E-06	18.57868595
ENSG00000261673	RP11-328J14.1	0.390508	1.00E-06	18.57499258
ENSG00000233351	RP1-69D17.3	0.390508	1.00E-06	18.57499258
ENSG00000258172	RP11-1105G2.4	0.390489	1.00E-06	18.57492238
ENSG00000265527	MIR5690	0.390437	1.00E-06	18.57473025
ENSG00000207746	MIR575	0.389844	1.00E-06	18.5725374
ENSG00000176732	PFN4	0.388839063	1.00E-06	18.56881363
ENSG00000252231	RNA5SP67	0.38852	1.00E-06	18.56762934
ENSG00000238151	MLLT10P1	0.38852	1.00E-06	18.56762934
ENSG00000252719	SNORA18	0.38852	1.00E-06	18.56762934
ENSG00000274674	MIR6741	0.38778	1.00E-06	18.56487887
ENSG00000229278	RP11-483F11.7	0.386341	1.00E-06	18.55951526
ENSG00000261550	RP11-467J12.4	0.386287	1.00E-06	18.5593136
ENSG00000280278	FLJ30679	0.385577	1.00E-06	18.55665947
ENSG00000206644	RNU6-1279P	0.385428	1.00E-06	18.55610186
ENSG00000236799	RP11-383C6.2	0.38515	1.00E-06	18.5550609
ENSG00000260022	LA16c-306A4.1	0.384122	1.00E-06	18.55120507
ENSG00000232142	RP11-367H5.8	0.383741	1.00E-06	18.54977339
ENSG00000213304	CTC-398G3.1	0.383399	1.00E-06	18.54848705
ENSG00000272386	RP11-666A8.12	0.382678	1.00E-06	18.54577144
ENSG00000202231	SNORA9	0.382678	1.00E-06	18.54577144
ENSG00000267191	RP11-15A1.2	0.379999	1.00E-06	18.5356361
ENSG00000255647	CTD-2373J6.2	0.379822652	1.00E-06	18.53496642
ENSG00000259405	ISCA1P4	0.379822	1.00E-06	18.53496395
ENSG00000258045	Metazoa_SRP	0.378873	1.00E-06	18.53135481
ENSG00000226856	AC093901.1	0.378472157	1.00E-06	18.52982765
ENSG00000200629	RNY1P11	0.378343	1.00E-06	18.52933523
ENSG00000206612	SNORA2A	0.377009	1.00E-06	18.52423944
ENSG00000212445	SNORA48	0.377009	1.00E-06	18.52423944
ENSG00000207217	SNORA42	0.377009	1.00E-06	18.52423944
ENSG00000252313	RNA5SP281	0.377009	1.00E-06	18.52423944
ENSG00000223807	BOLA3P4	0.376632	1.00E-06	18.52279606
ENSG00000274441	CTD-2282P23.2	0.37658	1.00E-06	18.52259686
ENSG00000235897	TM4SF19-AS1	0.376560788	1.00E-06	18.52252325
ENSG00000228536	RP11-392O17.1	0.376544	1.00E-06	18.52245893
ENSG00000228874	AC090954.5	0.375964	1.00E-06	18.520235
ENSG00000200792	SNORA80A	0.374237	1.00E-06	18.51359268
ENSG00000272021	AC008592.8	0.372185	1.00E-06	18.50566039
ENSG00000273786	RP11-133K1.11	0.372048	1.00E-06	18.50512924

ENSG00000252274	SCARNA24	0.371506	1.00E-06	18.50302599
ENSG00000249020	SNORA58	0.371506	1.00E-06	18.50302599
ENSG00000201077	RNU6-188P	0.371019	1.00E-06	18.50113354
ENSG00000263399	MIR3170	0.370154	1.00E-06	18.49776609
ENSG00000201136	RNU6-353P	0.367584	1.00E-06	18.48771445
ENSG00000243738	RN7SL181P	0.367482	1.00E-06	18.48731406
ENSG00000266258	RP11-41O4.1	0.3653139	1.00E-06	18.47877712
ENSG00000239945	RP11-34P13.8	0.365187	1.00E-06	18.47827588
ENSG00000248101	AC002116.8	0.364659	1.00E-06	18.47618848
ENSG00000276830	MIR6730	0.36463	1.00E-06	18.47607374
ENSG00000256282	RP11-504G3.4	0.364335115	1.00E-06	18.47490652
ENSG00000225704	RP4-798A17.5	0.364172	1.00E-06	18.47426048
ENSG00000234003	MTATP6P27	0.363966	1.00E-06	18.47344416
ENSG00000184270	HIST2H2AB	0.363914	1.00E-06	18.47323803
ENSG00000259533	RP11-630H24.3	0.362942	1.00E-06	18.46937949
ENSG00000201852	RNU6-702P	0.361506	1.00E-06	18.46366007
ENSG00000270035	RP11-338N10.2	0.360738	1.00E-06	18.46059188
ENSG00000260366	CTD-2639E6.4	0.36036	1.00E-06	18.45907936
ENSG00000272468	RP1-86C11.7	0.360327	1.00E-06	18.45894723
ENSG00000260989	LA16c-395F10.2	0.359424	1.00E-06	18.45532722
ENSG00000207170	RNU6-1215P	0.359267	1.00E-06	18.4546969
ENSG00000225675	RP4-784A16.5	0.359056	1.00E-06	18.45384935
ENSG00000278794	Metazoa_SRP	0.35837	1.00E-06	18.45109035
ENSG00000266959	AC005786.3	0.357879	1.00E-06	18.44911236
ENSG00000233509	ZNF197-AS1	0.357826	1.00E-06	18.44889869
ENSG00000248787	RP11-666A20.4	0.357585	1.00E-06	18.44792669
ENSG00000258284	POLR2KP1	0.357485	1.00E-06	18.44752318
ENSG00000223739	AC007389.2	0.355941	1.00E-06	18.4412786
ENSG00000273204	RP4-549L20.3	0.355917	1.00E-06	18.44118132
ENSG00000279219	AL008723.1	0.35443	1.00E-06	18.4351412
ENSG00000212138	RNA5SP372	0.354061	1.00E-06	18.43363841
ENSG00000275322	RP11-262A16.1	0.353914	1.00E-06	18.43303931
ENSG00000253697	RP11-1081K18.1	0.353446	1.00E-06	18.43113029
ENSG00000241959	RN7SL76P	0.352223	1.00E-06	18.42612959
ENSG00000226245	ZNF32-AS1	0.351817	1.00E-06	18.42446567
ENSG00000258122	RP11-61A14.1	0.351817	1.00E-06	18.42446567
ENSG00000266995	RP11-703I16.3	0.351184	1.00E-06	18.42186759
ENSG00000280653	AP002518.1	0.351009	1.00E-06	18.4211485
ENSG00000278084	RP11-87C12.6	0.351009	1.00E-06	18.4211485
ENSG00000227609	TMEM183AP1	0.351009	1.00E-06	18.4211485
ENSG00000241188	RN7SL165P	0.349803	1.00E-06	18.41618314
ENSG00000238963	U8	0.349668	1.00E-06	18.41562625
ENSG00000265178	MIR4728	0.349437	1.00E-06	18.41467285
ENSG00000252341	Y_RNA	0.348734	1.00E-06	18.4117675
ENSG00000253671	RP11-806O11.1	0.348704701	1.00E-06	18.41164629
ENSG00000225483	RP11-38J22.2	0.348008	1.00E-06	18.40876095
ENSG00000274713	MIR7974	0.347898	1.00E-06	18.40830486
ENSG00000277839	AC113133.2	0.347898	1.00E-06	18.40830486

ENSG00000251221	LINC01337	0.347019	1.00E-06	18.40465513
ENSG00000234921	RP11-85O21.5	0.346774	1.00E-06	18.40363621
ENSG00000268750	CTD-2583A14.10	0.346472651	1.00E-06	18.40238195
ENSG00000240571	RP11-775D22.3	0.346173	1.00E-06	18.40113368
ENSG00000277052	MIR6763	0.344529	1.00E-06	18.3942659
ENSG00000259763	RP11-327J17.1	0.343537	1.00E-06	18.39010596
ENSG00000248373	RP11-556I14.1	0.343230622	1.00E-06	18.38881875
ENSG00000267615	RP11-552F3.13	0.343164	1.00E-06	18.38853869
ENSG00000252271	RNU6-1110P	0.342668	1.00E-06	18.38645195
ENSG00000281423	AC010900.1	0.34184	1.00E-06	18.3829617
ENSG00000272940	CTA-384D8.33	0.340822	1.00E-06	18.37865894
ENSG00000275132	RN7SL663P	0.340443	1.00E-06	18.37705374
ENSG00000261829	RP11-223I10.1	0.340443	1.00E-06	18.37705374
ENSG00000273516	U1	0.339308	1.00E-06	18.37223592
ENSG00000266043	MIR3649	0.339308	1.00E-06	18.37223592
ENSG00000201075	Y_RNA	0.339308	1.00E-06	18.37223592
ENSG00000279471	AC008759.1	0.339308	1.00E-06	18.37223592
ENSG00000252060	RNA5SP464	0.339308	1.00E-06	18.37223592
ENSG00000199530	Y_RNA	0.339308	1.00E-06	18.37223592
ENSG00000271400	RP13-266I11.1	0.337671	1.00E-06	18.36525876
ENSG00000202374	SNORA62	0.337061	1.00E-06	18.36265018
ENSG00000236643	RP11-175D17.3	0.336645	1.00E-06	18.36086851
ENSG00000212293	SNORA16	0.336637	1.00E-06	18.36083423
ENSG00000267505	CTC-296K1.3	0.336607	1.00E-06	18.36070565
ENSG00000280207	RP11-423H2.4	0.336318	1.00E-06	18.35946647
ENSG00000263512	MIR4311	0.335915	1.00E-06	18.35773669
ENSG00000235629	AC090952.5	0.335504	1.00E-06	18.35597044
ENSG00000253037	AL109947.2	0.33446	1.00E-06	18.35147416
ENSG00000244256	RN7SL130P	0.333746	1.00E-06	18.34839102
ENSG00000264395	MIR3193	0.333139	1.00E-06	18.34576473
ENSG00000265349	RP11-55L4.2	0.329329	1.00E-06	18.32917003
ENSG00000272375	RP11-51J9.6	0.328261	1.00E-06	18.32448383
ENSG00000243629	LINC00880	0.327874	1.00E-06	18.32278198
ENSG00000234202	RP11-330C7.4	0.326258	1.00E-06	18.31565375
ENSG00000242865	RN7SL244P	0.325598	1.00E-06	18.31273231
ENSG00000226745	RP11-115D7.3	0.325438	1.00E-06	18.31202319
ENSG00000168539	CHRM1	0.325108848	1.00E-06	18.31056329
ENSG00000255503	RP11-113K21.4	0.322935	1.00E-06	18.30088428
ENSG00000223899	SEC13P1	0.322128	1.00E-06	18.29727454
ENSG00000233081	RP11-440G5.2	0.322128	1.00E-06	18.29727454
ENSG00000273084	RP11-1275H24.3	0.320938	1.00E-06	18.29193509
ENSG00000260886	TAT-AS1	0.320235	1.00E-06	18.28877147
ENSG00000274944	RP5-864K19.6	0.319255763	1.00E-06	18.28435314
ENSG00000170152	RP11-374M1.7	0.319007	1.00E-06	18.28322856
ENSG00000270689	BUD13P1	0.318572	1.00E-06	18.28125995
ENSG00000280675	AC092384.1	0.318101	1.00E-06	18.27912538
ENSG00000223460	GAPDHP69	0.317428	1.00E-06	18.27606987
ENSG00000271429	RPL17P51	0.317211	1.00E-06	18.27508328

ENSG00000224251	RP11-499O7.7	0.316781068	1.00E-06	18.27312659
ENSG00000277542	HOXB13-AS1_2	0.314174	1.00E-06	18.26120427
ENSG00000207128	RNU6-729P	0.313939	1.00E-06	18.26012474
ENSG00000277130	RP11-530C5.4	0.311945	1.00E-06	18.25093216
ENSG00000273064	RP11-474G23.3	0.311928	1.00E-06	18.25085354
ENSG00000230778	ANKRD63	0.311701	1.00E-06	18.24980326
ENSG00000267405	CTC-296K1.4	0.311509	1.00E-06	18.24891432
ENSG00000255303	OR5BA1P	0.310975	1.00E-06	18.24643908
ENSG00000280537	RP11-33O4.2	0.310914288	1.00E-06	18.24615739
ENSG00000275263	RP11-1072A3.4	0.310343	1.00E-06	18.24350408
ENSG00000199469	RNU1-62P	0.310343	1.00E-06	18.24350408
ENSG00000274963	RN7SL600P	0.309803	1.00E-06	18.24099159
ENSG00000244268	RP11-529G21.2	0.308844	1.00E-06	18.23651878
ENSG00000259333	RP11-630H24.4	0.307811	1.00E-06	18.23168526
ENSG00000223947	AC016738.4	0.307432	1.00E-06	18.22990781
ENSG00000259556	RP11-56B16.2	0.307132	1.00E-06	18.22849931
ENSG00000252947	SCARNA1	0.306604	1.00E-06	18.22601699
ENSG00000221634	MIR1276	0.306604	1.00E-06	18.22601699
ENSG00000228115	RP11-165F24.5	0.305763	1.00E-06	18.22205431
ENSG00000278328	MIR6802	0.305377	1.00E-06	18.22023188
ENSG00000216206	AF228730.1	0.305377	1.00E-06	18.22023188
ENSG00000279340	RP11-667K14.9	0.304767	1.00E-06	18.21734717
ENSG00000260973	RP11-105C19.2	0.304278	1.00E-06	18.2150305
ENSG00000222328	RNU2-2P	0.303778	1.00E-06	18.21265787
ENSG00000281685	AC008521.1	0.303592	1.00E-06	18.21177425
ENSG00000224328	MDC1-AS1	0.30287	1.00E-06	18.20833916
ENSG00000263567	RP11-805L22.3	0.302354	1.00E-06	18.20587914
ENSG00000177669	MBOAT4	0.302281	1.00E-06	18.20553078
ENSG00000251476	MTCO1P31	0.302067	1.00E-06	18.20450906
ENSG00000258761	RP11-24J19.1	0.301425	1.00E-06	18.20143955
ENSG00000269387	RP11-298J23.8	0.301161	1.00E-06	18.20017543
ENSG00000253917	AC226119.5	0.301003	1.00E-06	18.19941834
ENSG00000267397	RP11-873E20.1	0.300505	1.00E-06	18.19702947
ENSG00000201733	SNORA43	0.298106	1.00E-06	18.18546589
ENSG00000231622	RP4-700A9.1	0.297639	1.00E-06	18.18320405
ENSG00000272370	RP11-307L14.1	0.29706	1.00E-06	18.18039483
ENSG00000237870	AC073130.1	0.297027108	1.00E-06	18.18023508
ENSG00000276863	CTD-2047H16.5	0.294244	1.00E-06	18.16665347
ENSG00000199130	MIR365A	0.293455	1.00E-06	18.16277976
ENSG00000259775	RP11-45P15.4	0.293349	1.00E-06	18.16225855
ENSG00000236750	AC009237.16	0.293349	1.00E-06	18.16225855
ENSG00000235021	RP11-439E19.7	0.292507	1.00E-06	18.15811162
ENSG00000239576	COX6CP14	0.292507	1.00E-06	18.15811162
ENSG00000230637	CTA-246H3.8	0.291882	1.00E-06	18.15502572
ENSG00000279501	bP-2171C21.3	0.291856	1.00E-06	18.1548972
ENSG00000175749	EIF3KP1	0.291722	1.00E-06	18.15423466
ENSG00000226797	AC015923.1	0.291391	1.00E-06	18.15259679
ENSG00000265033	RN7SL262P	0.290835	1.00E-06	18.14984137

ENSG00000273403	RP11-329B9.3	0.290282	1.00E-06	18.14709559
ENSG00000252150	AC092574.2	0.289502	1.00E-06	18.14321379
ENSG00000239670	RP4-803A2.2	0.288982	1.00E-06	18.14062011
ENSG00000231196	RP11-495P10.7	0.288894914	1.00E-06	18.14018528
ENSG00000267757	EML2-AS1	0.288253407	1.00E-06	18.13697814
ENSG00000242399	RPS20P23	0.287549	1.00E-06	18.13344829
ENSG00000271424	RP11-647O20.1	0.287489	1.00E-06	18.13314723
ENSG00000259579	RP11-66B24.5	0.287217	1.00E-06	18.13178162
ENSG00000233275	AC009238.8	0.286739	1.00E-06	18.12937862
ENSG00000259460	RP11-128A17.1	0.28661	1.00E-06	18.12872942
ENSG00000233942	AC004012.1	0.285934	1.00E-06	18.12532265
ENSG00000186146	DEFB131	0.285567	1.00E-06	18.12346975
ENSG00000180592	SKIDA1	0.285087763	1.00E-06	18.12104659
ENSG00000255496	RP11-587D21.4	0.284337	1.00E-06	18.11724232
ENSG00000265280	AC018804.1	0.284226	1.00E-06	18.11667901
ENSG00000274885	RP11-268P4.6	0.284171	1.00E-06	18.11639981
ENSG00000269846	RP4-621N11.2	0.284055	1.00E-06	18.11581077
ENSG00000276272	RP11-121C6.5	0.283256	1.00E-06	18.11174699
ENSG00000275438	LA16c-360A4.1	0.282234	1.00E-06	18.10653227
ENSG00000265200	AC090324.1	0.282197	1.00E-06	18.10634313
ENSG00000221048	AC079781.1	0.281992	1.00E-06	18.10529471
ENSG00000275178	RP11-4B16.3	0.281973	1.00E-06	18.1051975
ENSG00000272699	RP11-145M9.5	0.280595	1.00E-06	18.09812978
ENSG00000275439	AP001619.1	0.280448	1.00E-06	18.09737377
ENSG00000164128	NPY1R	0.280050615	1.00E-06	18.09532807
ENSG00000250808	MTCO2P30	0.279649	1.00E-06	18.09325765
ENSG00000254328	CTC-308K20.4	0.279649	1.00E-06	18.09325765
ENSG00000201047	Y_RNA	0.27943	1.00E-06	18.09212739
ENSG00000206967	Y_RNA	0.27943	1.00E-06	18.09212739
ENSG00000220370	RP3-399J4.2	0.279253	1.00E-06	18.09121325
ENSG00000186603	HPDL	0.27807	1.00E-06	18.08508858
ENSG00000265435	MIR3121	0.277615	1.00E-06	18.082726
ENSG00000131097	HIGD1B	0.277019605	1.00E-06	18.07962856
ENSG00000211519	MIR147B	0.276748	1.00E-06	18.07821337
ENSG00000202008	Y_RNA	0.27661	1.00E-06	18.07749379
ENSG00000207086	Y_RNA	0.275886	1.00E-06	18.07371272
ENSG00000281523	AC104581.1	0.275687	1.00E-06	18.07267171
ENSG00000207839	MIR33B	0.275687	1.00E-06	18.07267171
ENSG00000272109	CTD-2260A17.3	0.273712529	1.00E-06	18.06230195
ENSG00000255462	RP11-483L5.1	0.273635	1.00E-06	18.06189325
ENSG00000261431	RP4-616B8.4	0.272173	1.00E-06	18.05416443
ENSG00000239122	RNU2-11P	0.272173	1.00E-06	18.05416443
ENSG00000281080	Metazoa_SRP	0.271573	1.00E-06	18.05098053
ENSG00000233416	AC012065.5	0.271447	1.00E-06	18.05031101
ENSG00000271748	MIR92B	0.270824	1.00E-06	18.04699607
ENSG00000278405	AL606500.1	0.270724	1.00E-06	18.04646326
ENSG00000272354	RP11-307L14.2	0.270724	1.00E-06	18.04646326
ENSG00000280583	AC138645.1	0.270386	1.00E-06	18.04466093

ENSG00000224969	RP11-54O7.11	0.27015	1.00E-06	18.04340116
ENSG00000135312	HTR1B	0.26983	1.00E-06	18.04169123
ENSG00000273765	RP11-370I10.11	0.268927	1.00E-06	18.03685508
ENSG00000259488	RP11-154J22.1	0.268386206	1.00E-06	18.033951
ENSG00000263433	RP11-260A9.1	0.26837	1.00E-06	18.03386388
ENSG00000222222	RNU2-17P	0.267875	1.00E-06	18.03120042
ENSG00000223336	RNU2-6P	0.267875	1.00E-06	18.03120042
ENSG00000258073	RP11-1079J22.1	0.266766	1.00E-06	18.02521528
ENSG00000222800	RNU2-62P	0.266472	1.00E-06	18.02362442
ENSG00000225798	AC025918.2	0.265777	1.00E-06	18.01985674
ENSG00000225408	RP11-207C16.4	0.265053	1.00E-06	18.01592134
ENSG00000236091	RP3-473B4.3	0.264809	1.00E-06	18.01459263
ENSG00000277245	RP11-48G14.3	0.264168	1.00E-06	18.01109619
ENSG00000272589	ZSWIM8-AS1	0.264099	1.00E-06	18.01071931
ENSG00000276417	RP11-266K4.13	0.26396	1.00E-06	18.0099598
ENSG00000233626	RP11-565J7.1	0.263711	1.00E-06	18.00859823
ENSG00000224620	MEF2AP1	0.263153	1.00E-06	18.00554232
ENSG00000259421	CTA-150C2.20	0.26303	1.00E-06	18.00486783
ENSG00000280903	Metazoa_SRP	0.26256	1.00E-06	18.00228762
ENSG00000271155	RP11-435O5.5	0.262489	1.00E-06	18.00189744
ENSG00000227730	MTND6P5	0.262433	1.00E-06	18.00158962
ENSG00000260869	AC002310.13	0.262304	1.00E-06	18.00088028
ENSG00000199683	RN7SKP185	0.262287	1.00E-06	18.00078678
ENSG00000239804	RP11-379B18.1	0.262014	1.00E-06	17.99928437
ENSG00000150276	PPIAP26	0.261523	1.00E-06	17.99657831
ENSG00000255156	RNY1P9	0.261485	1.00E-06	17.99636866
ENSG00000208036	MIR106B	0.260688	1.00E-06	17.99196465
ENSG00000221468	RNU6ATAC11P	0.260589	1.00E-06	17.99141666
ENSG00000197079	KRT35	0.260493	1.00E-06	17.99088508
ENSG00000270720	RP11-84C13.2	0.259674	1.00E-06	17.98634205
ENSG00000220130	RP1-72A23.3	0.259247	1.00E-06	17.98396777
ENSG00000248172	RP11-1094H24.3	0.258824	1.00E-06	17.98161187
ENSG00000264012	RP11-627G18.2	0.258025	1.00E-06	17.97715133
ENSG00000232709	MARK2P9	0.257576	1.00E-06	17.97463865
ENSG00000273428	RP4-539M6.22	0.257373	1.00E-06	17.97350119
ENSG00000241678	RP11-732A19.1	0.257138	1.00E-06	17.9721833
ENSG00000177807	KCNJ10	0.257126	1.00E-06	17.97211597
ENSG00000252604	RNU2-44P	0.257104	1.00E-06	17.97199253
ENSG00000279903	RP11-349F21.5	0.257051	1.00E-06	17.9716951
ENSG00000276036	RNA5SP440	0.25662	1.00E-06	17.96927409
ENSG00000176933	TOB2P1	0.256533	1.00E-06	17.9687849
ENSG00000199004	MIR21	0.255985	1.00E-06	17.96569975
ENSG00000234735	AL022237.3	0.255903	1.00E-06	17.96523753
ENSG00000262358	CTD-3195I5.4	0.25576	1.00E-06	17.96443112
ENSG00000215263	AC025750.7	0.255153	1.00E-06	17.96100308
ENSG00000262039	RP11-81K2.1	0.255103	1.00E-06	17.96072034
ENSG00000266957	RP11-49K24.4	0.254481	1.00E-06	17.95719842
ENSG00000274034	MIR6813	0.254481	1.00E-06	17.95719842

ENSG00000281592	Z95116.1	0.254481	1.00E-06	17.95719842
ENSG00000266525	MIR4650-1	0.254481	1.00E-06	17.95719842
ENSG00000264494	MIR4650-2	0.254481	1.00E-06	17.95719842
ENSG00000118526	TCF21	0.252971	1.00E-06	17.94861248
ENSG00000172238	ATOH1	0.252181	1.00E-06	17.94410006
ENSG00000278353	AC116533.4	0.252057	1.00E-06	17.9433905
ENSG00000263636	AC138304.1	0.251962	1.00E-06	17.94284664
ENSG00000281871	AC012360.1	0.251962	1.00E-06	17.94284664
ENSG00000265665	AC008391.1	0.251962	1.00E-06	17.94284664
ENSG00000254816	RP11-670N15.2	0.251871	1.00E-06	17.9423255
ENSG00000230779	RP1-140J1.4	0.251461	1.00E-06	17.93997514
ENSG00000224314	RP3-378P9.2	0.25134	1.00E-06	17.93928076
ENSG00000274758	RP1-59D14.10	0.25072	1.00E-06	17.93571756
ENSG00000241532	AGGF1P3	0.250495	1.00E-06	17.93442228
ENSG00000250645	CTD-2228K2.1	0.250104	1.00E-06	17.93216861
ENSG00000272746	RP11-53B2.6	0.249899	1.00E-06	17.9309856
ENSG00000274983	AL138994.1	0.249288	1.00E-06	17.92745391
ENSG00000253543	RP11-89K10.2	0.249067	1.00E-06	17.92617436
ENSG00000275964	RP11-61K9.3	0.248711	1.00E-06	17.92411079
ENSG00000281516	SCARNA4	0.248274	1.00E-06	17.92157366
ENSG00000225839	TRMT2B-AS1	0.248274	1.00E-06	17.92157366
ENSG00000250057	RP11-576N17.5	0.248022	1.00E-06	17.92010857
ENSG00000228573	RP11-279N8.1	0.24767	1.00E-06	17.9180596
ENSG00000260549	MT1L	0.24767	1.00E-06	17.9180596
ENSG00000236519	LINC01424	0.246829	1.00E-06	17.91315238
ENSG00000239315	RPL19P13	0.246187	1.00E-06	17.90939506
ENSG00000267582	CTD-3252C9.2	0.245999	1.00E-06	17.90829293
ENSG00000232906	RP11-746P2.3	0.245678	1.00E-06	17.90640915
ENSG00000259523	RP11-680F8.3	0.245537	1.00E-06	17.90558092
ENSG00000275728	RP4-777L9.3	0.245417	1.00E-06	17.90487566
ENSG00000255421	CTD-2011F17.2	0.244914	1.00E-06	17.90191572
ENSG00000252212	RNU2-58P	0.244747	1.00E-06	17.90093165
ENSG00000223976	EXTL2P1	0.244694	1.00E-06	17.9006192
ENSG00000253398	RP11-775B15.2	0.244411	1.00E-06	17.89894969
ENSG00000267401	RP11-396N11.1	0.242941	1.00E-06	17.89024646
ENSG00000264754	CTD-2653B5.1	0.242838	1.00E-06	17.88963467
ENSG00000232640	RP1-266L20.2	0.242483	1.00E-06	17.88752408
ENSG00000275997	RP11-216P6.2	0.242363	1.00E-06	17.88680994
ENSG00000201308	RNU6-512P	0.242128	1.00E-06	17.8854104
ENSG00000229947	RP13-766D20.1	0.241966	1.00E-06	17.88444482
ENSG00000163823	CCR1	0.241902	1.00E-06	17.88406317
ENSG00000272145	NFYC-AS1	0.241357	1.00E-06	17.88080914
ENSG00000276413	RP3-393E18.3	0.241214	1.00E-06	17.87995412
ENSG00000277463	CTD-3035K23.6	0.240644	1.00E-06	17.87654093
ENSG00000249590	RP4-539M6.19	0.240363854	1.00E-06	17.87486043
ENSG00000207185	RNU6-1157P	0.240076	1.00E-06	17.87313166
ENSG00000201346	U3	0.240076	1.00E-06	17.87313166
ENSG00000180019	AC079741.2	0.240076	1.00E-06	17.87313166

ENSG00000244048	RPS18P6	0.240035	1.00E-06	17.87288526
ENSG00000214754	AC004870.5	0.239863	1.00E-06	17.87185111
ENSG00000251281	CTD-2066L21.2	0.239511	1.00E-06	17.86973239
ENSG00000237481	RP4-803J11.2	0.238576	1.00E-06	17.86408939
ENSG00000212447	SNORD90	0.238432	1.00E-06	17.86321835
ENSG00000258159	IMMP1LP2	0.238275	1.00E-06	17.86226807
ENSG00000200254	RNU6-536P	0.237833	1.00E-06	17.85958938
ENSG00000271063	SNRPGP17	0.237833	1.00E-06	17.85958938
ENSG00000213724	PRDX2P2	0.237573	1.00E-06	17.85801136
ENSG00000221060	SNORA11	0.236726	1.00E-06	17.85285864
ENSG00000271200	RP11-430G17.3	0.236006	1.00E-06	17.84846401
ENSG00000231334	AC104781.1	0.235892	1.00E-06	17.84776697
ENSG00000264569	RP13-650J16.1	0.235458	1.00E-06	17.84511022
ENSG00000263986	RP11-746M1.1	0.235153	1.00E-06	17.84324021
ENSG00000224335	RP11-301M17.2	0.234545	1.00E-06	17.83950522
ENSG00000260922	RP11-538I12.3	0.234185	1.00E-06	17.83728915
ENSG00000251870	RNU2-69P	0.234123	1.00E-06	17.83690714
ENSG00000267021	MIR4321	0.233371	1.00E-06	17.83226577
ENSG00000238669	RNU6-333P	0.233076	1.00E-06	17.83044093
ENSG00000272264	RP11-92K15.3	0.232869	1.00E-06	17.82915907
ENSG00000223660	AC012314.20	0.232855	1.00E-06	17.82907234
ENSG00000221638	U3	0.232402	1.00E-06	17.82626296
ENSG00000235861	AC005237.2	0.232402	1.00E-06	17.82626296
ENSG00000212529	SNORA57	0.231665	1.00E-06	17.82168057
ENSG00000265996	MIR3671	0.231347	1.00E-06	17.81969887
ENSG00000269547	CTC-360G5.8	0.230891	1.00E-06	17.81685241
ENSG00000225480	AC006548.19	0.230862	1.00E-06	17.8166712
ENSG00000249304	RP11-26P13.2	0.230821	1.00E-06	17.81641496
ENSG00000266283	RP11-627G18.1	0.230691	1.00E-06	17.8156022
ENSG00000269970	RP11-498E2.9	0.230299	1.00E-06	17.81314862
ENSG00000213061	PFN1P11	0.230276	1.00E-06	17.81300453
ENSG00000236921	RP11-408N14.1	0.22978	1.00E-06	17.80989371
ENSG00000236723	RP5-1024G6.2	0.229269768	1.00E-06	17.8066866
ENSG00000231564	EIF4A1P11	0.229262	1.00E-06	17.80663772
ENSG00000273973	RP11-973D8.5	0.229033	1.00E-06	17.80519596
ENSG00000276509	CH17-353B19.1	0.228804	1.00E-06	17.80375275
ENSG00000207412	RNU6-455P	0.228319	1.00E-06	17.8006914
ENSG00000255382	RP11-718B12.2	0.228234	1.00E-06	17.8001542
ENSG00000271265	RP11-230C9.4	0.228234	1.00E-06	17.8001542
ENSG00000234488	AC096664.1	0.228155	1.00E-06	17.79965475
ENSG00000266189	MIR3186	0.227536	1.00E-06	17.7957353
ENSG00000226609	RP11-276E15.4	0.227402	1.00E-06	17.79488542
ENSG00000249623	RP11-610J23.1	0.227215	1.00E-06	17.79369855
ENSG00000216676	RP1-15D7.1	0.227215	1.00E-06	17.79369855
ENSG00000232249	LL0XNC01-37G1.2	0.227215	1.00E-06	17.79369855
ENSG00000200473	RNA5SP507	0.22714	1.00E-06	17.79322227
ENSG00000227813	RP1-180M12.1	0.226436	1.00E-06	17.78874382
ENSG00000256093	RP11-158L12.2	0.225952	1.00E-06	17.7856568

ENSG00000228906	RP13-216E22.4	0.225703	1.00E-06	17.78406607
ENSG00000233018	RP4-597N16.1	0.225205	1.00E-06	17.78087933
ENSG00000200391	Y_RNA	0.225205	1.00E-06	17.78087933
ENSG00000238845	Y_RNA	0.225205	1.00E-06	17.78087933
ENSG00000252171	Y_RNA	0.225205	1.00E-06	17.78087933
ENSG00000227625	RP11-520H14.1	0.225118	1.00E-06	17.78032189
ENSG00000270619	RP11-197B12.1	0.225015	1.00E-06	17.77966165
ENSG00000260381	RP11-429P3.5	0.224677	1.00E-06	17.77749292
ENSG00000260685	RP11-326L17.1	0.224212	1.00E-06	17.77450397
ENSG00000221530	AC005037.1	0.223229	1.00E-06	17.76816494
ENSG00000211535	MIR711	0.223229	1.00E-06	17.76816494
ENSG00000261019	RP11-111K18.2	0.223229	1.00E-06	17.76816494
ENSG00000219758	RP1-249H1.3	0.222578	1.00E-06	17.76395148
ENSG00000281522	Y_RNA	0.222403	1.00E-06	17.76281672
ENSG00000224478	RP11-13P5.1	0.221654	1.00E-06	17.75794987
ENSG00000266485	AL365214.1	0.22059	1.00E-06	17.75100787
ENSG00000243055	GK-AS1	0.220472	1.00E-06	17.75023592
ENSG00000271133	CTA-293F17.1	0.220398674	1.00E-06	17.74975602
ENSG00000207696	MIR659	0.220375	1.00E-06	17.74960104
ENSG00000265519	CTD-3157E16.1	0.220012	1.00E-06	17.74722269
ENSG00000270883	RP11-461L13.5	0.219854	1.00E-06	17.74618626
ENSG00000273816	RP11-85B7.5	0.21938	1.00E-06	17.74307248
ENSG00000276768	RP5-1116H23.5	0.218909	1.00E-06	17.73997174
ENSG00000252755	RNU6-703P	0.218806	1.00E-06	17.73929277
ENSG00000240143	RP4-753P9.3	0.218573	1.00E-06	17.73775567
ENSG00000257905	RP3-432I18.1	0.218438	1.00E-06	17.73686433
ENSG00000254002	RP11-213G6.2	0.218291	1.00E-06	17.73589312
ENSG00000224481	RP11-495P10.3	0.2181	1.00E-06	17.73463024
ENSG00000183929	DUSP5P1	0.218063	1.00E-06	17.73438547
ENSG00000253528	RP11-347C18.4	0.21735	1.00E-06	17.72966057
ENSG00000257207	AC108938.5	0.217102	1.00E-06	17.72801349
ENSG00000237772	AC092620.3	0.217057	1.00E-06	17.72771442
ENSG00000257286	RP11-545P7.4	0.217031	1.00E-06	17.7275416
ENSG00000180305	WFDC10A	0.216273	1.00E-06	17.72249404
ENSG00000239207	GAPDHP39	0.21627	1.00E-06	17.72247403
ENSG00000229444	RP11-184I16.4	0.215957	1.00E-06	17.72038456
ENSG00000255010	RP11-113K21.3	0.215843	1.00E-06	17.71962278
ENSG00000277049	5S_rRNA	0.215661	1.00E-06	17.71840578
ENSG00000252636	RNU6-826P	0.21533	1.00E-06	17.71618981
ENSG00000257453	RP11-290L1.3	0.214798	1.00E-06	17.71262103
ENSG00000271052	RP11-479I16.2	0.214751	1.00E-06	17.71230532
ENSG00000205884	DEFB136	0.214751	1.00E-06	17.71230532
ENSG00000255723	RP11-529H2.2	0.214578	1.00E-06	17.71114264
ENSG00000267370	CTD-2623N2.3	0.21445	1.00E-06	17.71028179
ENSG00000279230	AL158839.1	0.214299	1.00E-06	17.70926559
ENSG00000229169	RP11-247I13.6	0.21385	1.00E-06	17.70623968
ENSG00000237611	Metazoa_SRP	0.21385	1.00E-06	17.70623968
ENSG00000270002	RP11-93H12.4	0.213768	1.00E-06	17.70568638

ENSG00000259932	CTD-2651B20.7	0.213225	1.00E-06	17.70201707
ENSG00000237848	RP11-739N20.3	0.213155	1.00E-06	17.70154337
ENSG00000221267	MIR1236	0.212899	1.00E-06	17.69980965
ENSG00000264879	RN7SL690P	0.212067	1.00E-06	17.69416061
ENSG00000266589	MIR4512	0.211516	1.00E-06	17.69040727
ENSG00000228413	AC024937.2	0.211363	1.00E-06	17.68936332
ENSG00000265257	RP11-21J18.1	0.210959006	1.00E-06	17.68660315
ENSG00000268496	CTD-2587H19.2	0.21075	1.00E-06	17.68517311
ENSG00000243220	AC006159.4	0.210351	1.00E-06	17.68243915
ENSG00000250053	RARRES2P4	0.210315	1.00E-06	17.68219222
ENSG00000233073	AC005009.2	0.209972925	1.00E-06	17.67984379
ENSG00000213543	RARRES2P2	0.209882	1.00E-06	17.67921892
ENSG00000228181	RP11-455B2.9	0.209449	1.00E-06	17.67623947
ENSG00000273763	RP11-420C9.1	0.209449	1.00E-06	17.67623947
ENSG00000258752	RP11-356K23.1	0.208830603	1.00E-06	17.67197362
ENSG00000265521	MIR5697	0.208805	1.00E-06	17.67179673
ENSG00000278125	Z99916.1	0.208211	1.00E-06	17.66768676
ENSG00000224421	ATP5J2LP	0.207916	1.00E-06	17.66564126
ENSG00000266587	AC027320.1	0.20774	1.00E-06	17.66441951
ENSG00000263280	LA16c-325D7.2	0.207553	1.00E-06	17.66312026
ENSG00000202273	Y_RNA	0.207537	1.00E-06	17.66300904
ENSG00000251395	FTH1P9	0.206452	1.00E-06	17.65544687
ENSG00000278097	AC131056.5	0.206057	1.00E-06	17.65268395
ENSG00000272081	CTD-2376I4.2	0.206057	1.00E-06	17.65268395
ENSG00000216109	AL713999.1	0.205872	1.00E-06	17.6513881
ENSG00000235077	AC073842.19	0.205641	1.00E-06	17.64976841
ENSG00000226965	AC003088.1	0.205641	1.00E-06	17.64976841
ENSG00000279226	CH507-152C13.5	0.205453	1.00E-06	17.64844887
ENSG00000225956	RP5-1077I2.3	0.205411	1.00E-06	17.64815392
ENSG00000267909	CCDC177	0.20526	1.00E-06	17.64709298
ENSG00000281834	Metazoa_SRP	0.204402	1.00E-06	17.64104979
ENSG00000232665	PHBP10	0.204386	1.00E-06	17.64093685
ENSG00000276696	Y_RNA	0.203585	1.00E-06	17.63527174
ENSG00000267786	AF038458.3	0.203585	1.00E-06	17.63527174
ENSG00000213549	AC005077.8	0.203585	1.00E-06	17.63527174
ENSG00000230424	RP1-43E13.2	0.20249	1.00E-06	17.62749114
ENSG00000260420	LA16c-444G7.2	0.202358	1.00E-06	17.62655036
ENSG00000260136	CTD-2270L9.4	0.202169575	1.00E-06	17.62520637
ENSG00000248936	RP11-36B15.1	0.20206	1.00E-06	17.62442423
ENSG00000265882	RN7SL73P	0.201969	1.00E-06	17.62377435
ENSG00000277010	RP11-616M22.12	0.201569	1.00E-06	17.62091425
ENSG00000239821	RN7SL513P	0.201536	1.00E-06	17.62067804
ENSG00000272567	RP11-73K9.3	0.201252	1.00E-06	17.61864359
ENSG00000280283	CTA-481E9.2	0.201217	1.00E-06	17.61839267
ENSG00000232166	RP4-799P18.5	0.200906	1.00E-06	17.61616112
ENSG00000261000	RP11-534L20.5	0.199985	1.00E-06	17.60953227
ENSG00000179059	ZFP42	0.199292729	1.00E-06	17.60452955
ENSG00000230601	RP11-402G3.3	0.199128	1.00E-06	17.60333657

ENSG00000257241	RP11-611E13.3	0.198918	1.00E-06	17.60181431
ENSG00000231173	AC116609.3	0.198813	1.00E-06	17.60105257
ENSG00000226047	RP11-196I18.4	0.198404	1.00E-06	17.59808159
ENSG00000255703	RP11-46H11.2	0.198297	1.00E-06	17.59730333
ENSG00000266155	RP11-927P21.6	0.19804	1.00E-06	17.59543233
ENSG00000244502	HDAC11-AS1	0.197655	1.00E-06	17.59262493
ENSG00000277087	RP5-1116H23.6	0.197272	1.00E-06	17.58982667
ENSG00000258671	RP11-407N17.2	0.197156	1.00E-06	17.58897809
ENSG00000275963	RP11-180M15.6	0.197017	1.00E-06	17.5879606
ENSG00000277653	CDKN2B-AS	0.196798	1.00E-06	17.58635603
ENSG00000278529	MIR8070	0.196645	1.00E-06	17.58523398
ENSG00000250615	CTC-564N23.2	0.196195	1.00E-06	17.58192875
ENSG00000281342	Metazoa_SRP	0.195276	1.00E-06	17.57515512
ENSG00000270690	RP11-349G13.3	0.19519	1.00E-06	17.57451962
ENSG00000242436	RN7SL789P	0.194552	1.00E-06	17.56979629
ENSG00000267052	CTB-30L5.1	0.194331	1.00E-06	17.56815653
ENSG00000228613	AC144450.1	0.194261	1.00E-06	17.56763677
ENSG00000259341	RP11-20G13.1	0.194247	1.00E-06	17.56753279
ENSG00000239247	RN7SL589P	0.193734	1.00E-06	17.56371764
ENSG00000274092	CTD-3203P2.3	0.193212	1.00E-06	17.55982517
ENSG00000234006	DDX39B-AS1	0.193154368	1.00E-06	17.55939478
ENSG00000230483	AC124057.5	0.193043	1.00E-06	17.55856272
ENSG00000214560	RPL21P41	0.192789	1.00E-06	17.55666321
ENSG00000261773	WI2-89031B12.1	0.192789	1.00E-06	17.55666321
ENSG00000258500	CTD-2062F14.2	0.192239	1.00E-06	17.55254152
ENSG00000278146	RP11-327J17.8	0.192138	1.00E-06	17.55178335
ENSG00000230881	RP11-486B10.3	0.192061	1.00E-06	17.55120507
ENSG00000275355	Metazoa_SRP	0.192061	1.00E-06	17.55120507
ENSG00000227965	AC098592.6	0.192061	1.00E-06	17.55120507
ENSG00000242296	DEFB109P1	0.192061	1.00E-06	17.55120507
ENSG00000270087	RP11-399K21.11	0.191994528	1.00E-06	17.55070567
ENSG00000236794	BCRP8	0.191609	1.00E-06	17.5478058
ENSG00000231274	SBK3	0.191521013	1.00E-06	17.54714316
ENSG00000200428	Y_RNA	0.191404	1.00E-06	17.54626145
ENSG00000239148	U8	0.191339	1.00E-06	17.54577144
ENSG00000278462	RP11-165D6.1	0.191339	1.00E-06	17.54577144
ENSG00000277859	SMAD5-AS1_1	0.191339	1.00E-06	17.54577144
ENSG00000273760	CH17-360D5.3	0.191066	1.00E-06	17.54371155
ENSG00000277504	RP11-278A23.4	0.19098	1.00E-06	17.54306204
ENSG00000251598	RP11-789C2.1	0.190934	1.00E-06	17.5427145
ENSG00000274096	AC104938.1	0.190861	1.00E-06	17.54216281
ENSG00000224794	RP3-333H23.8	0.190784	1.00E-06	17.54158066
ENSG00000251566	HMGB1P35	0.190648	1.00E-06	17.54055187
ENSG00000237180	CYP46A4P	0.190623	1.00E-06	17.54036268
ENSG00000227083	L29074.3	0.190623	1.00E-06	17.54036268
ENSG00000269053	CTD-2521M24.8	0.19059	1.00E-06	17.5401129
ENSG00000226864	ATE1-AS1	0.190458	1.00E-06	17.53911336
ENSG00000273998	RP4-777L9.2	0.190351	1.00E-06	17.53830262

ENSG00000206922	RNU6-80P	0.190266	1.00E-06	17.53765825
ENSG00000273391	RP11-634H22.1	0.190266	1.00E-06	17.53765825
ENSG00000254847	RP11-51B23.3	0.190248	1.00E-06	17.53752176
ENSG00000230648	RP3-406P24.3	0.189948	1.00E-06	17.535245
ENSG00000235892	PKMP2	0.189685	1.00E-06	17.53324607
ENSG00000259115	CTD-2540L5.5	0.189581	1.00E-06	17.53245486
ENSG00000267565	CTC-559E9.8	0.18944	1.00E-06	17.53138146
ENSG00000239799	ITIH4-AS1	0.189411	1.00E-06	17.53116059
ENSG00000225487	RP11-403P14.1	0.189229	1.00E-06	17.52977368
ENSG00000260992	DOCK9-AS2	0.189206	1.00E-06	17.52959831
ENSG00000260651	AF213884.2	0.189134	1.00E-06	17.52904921
ENSG00000259984	RP11-335G20.7	0.1886	1.00E-06	17.52497015
ENSG00000232875	HMGN2P35	0.188504	1.00E-06	17.52423561
ENSG00000257809	RP11-603J24.14	0.188504	1.00E-06	17.52423561
ENSG00000242330	RN7SL683P	0.188504	1.00E-06	17.52423561
ENSG00000263370	RP11-68I3.5	0.188504	1.00E-06	17.52423561
ENSG00000272071	RP11-332J15.4	0.188156	1.00E-06	17.52156977
ENSG00000277658	AL592188.8	0.188153	1.00E-06	17.52154677
ENSG00000274224	AL353644.1	0.188153	1.00E-06	17.52154677
ENSG00000265451	RP11-204L24.2	0.188063	1.00E-06	17.52085651
ENSG00000265139	RP11-227G15.2	0.187809	1.00E-06	17.51890667
ENSG00000228973	AC009955.8	0.187809	1.00E-06	17.51890667
ENSG00000259723	RP5-823G15.5	0.187809	1.00E-06	17.51890667
ENSG00000271378	C8orf59P2	0.187809	1.00E-06	17.51890667
ENSG00000275329	RP11-83N9.6	0.187789	1.00E-06	17.51875303
ENSG00000230289	RP11-334J6.6	0.187535	1.00E-06	17.51680035
ENSG00000256742	RP13-941N14.1	0.187119	1.00E-06	17.51359653
ENSG00000261039	RP11-417E7.2	0.187119	1.00E-06	17.51359653
ENSG00000264745	TTC39C-AS1	0.18689	1.00E-06	17.51182985
ENSG00000180221	TPT1P10	0.186757	1.00E-06	17.51080279
ENSG00000239389	PCDHA13	0.186570807	1.00E-06	17.50936374
ENSG00000213703	RP5-849H19.2	0.186433	1.00E-06	17.50829772
ENSG00000265516	Z84488.1	0.186433	1.00E-06	17.50829772
ENSG00000279538	AC004953.1	0.186433	1.00E-06	17.50829772
ENSG00000243762	AC006547.8	0.186414	1.00E-06	17.50815069
ENSG00000265850	MIR4797	0.186381	1.00E-06	17.50789527
ENSG00000213277	MARCKSL1P1	0.186274	1.00E-06	17.50706679
ENSG00000262759	MRPS21P9	0.186157	1.00E-06	17.50616034
ENSG00000268836	LA16c-OS12.2	0.186092	1.00E-06	17.50565651
ENSG00000257052	RP11-881M11.2	0.185991	1.00E-06	17.50487329
ENSG00000259627	RP11-244F12.2	0.185985	1.00E-06	17.50482674
ENSG00000228729	RP11-211A18.2	0.185979	1.00E-06	17.5047802
ENSG00000258001	RP11-756H6.1	0.185753	1.00E-06	17.50302599
ENSG00000261242	CTD-2302E22.4	0.185753	1.00E-06	17.50302599
ENSG00000239674	RP1-127L4.7	0.185753	1.00E-06	17.50302599
ENSG00000230622	UQCRHP1	0.185753	1.00E-06	17.50302599
ENSG00000276620	RP11-300D11.3	0.185753	1.00E-06	17.50302599
ENSG00000257763	OR5BK1P	0.185527	1.00E-06	17.50126963

ENSG00000158406	HIST1H4H	0.185165	1.00E-06	17.4984519
ENSG00000224810	RP11-538D16.2	0.18474	1.00E-06	17.49513675
ENSG00000273584	RP11-861L17.4	0.184406	1.00E-06	17.49252607
ENSG00000249183	SUMO2P4	0.184406	1.00E-06	17.49252607
ENSG00000160181	TFF2	0.183962304	1.00E-06	17.48905064
ENSG00000230772	VN1R108P	0.183962	1.00E-06	17.48904826
ENSG00000205596	RP5-1132H15.2	0.183962	1.00E-06	17.48904826
ENSG00000118990	GLRXP3	0.183924	1.00E-06	17.48875022
ENSG00000226501	USF1P1	0.183845	1.00E-06	17.48813041
ENSG00000254675	RP11-7I15.4	0.18374	1.00E-06	17.48730621
ENSG00000276032	RP11-12A20.6	0.18341	1.00E-06	17.48471278
ENSG00000273637	RP5-864K19.7	0.183386	1.00E-06	17.48452398
ENSG00000279659	RP11-177G23.1	0.182784	1.00E-06	17.47978026
ENSG00000252985	SNORD116	0.182704	1.00E-06	17.47914869
ENSG00000271741	RP11-244H3.4	0.182425	1.00E-06	17.47694393
ENSG00000279028	CTC-270D5.2	0.182423	1.00E-06	17.47692811
ENSG00000106540	AC004837.3	0.182288	1.00E-06	17.47586007
ENSG00000273901	CTD-2619J13.27	0.181715	1.00E-06	17.47131799
ENSG00000245059	RP11-303E16.7	0.181505	1.00E-06	17.46964977
ENSG00000235574	AC073150.6	0.181235	1.00E-06	17.46750207
ENSG00000273321	RP11-621L6.3	0.181125	1.00E-06	17.46662616
ENSG00000239472	RN7SL221P	0.181125	1.00E-06	17.46662616
ENSG00000264932	CTD-2104P17.2	0.180804	1.00E-06	17.46406707
ENSG00000257979	SNRPGP18	0.180753	1.00E-06	17.46366007
ENSG00000172073	TEX37	0.180537	1.00E-06	17.46193501
ENSG00000223202	RN7SKP297	0.180483	1.00E-06	17.46150343
ENSG00000274510	Metazoa_SRP	0.180483	1.00E-06	17.46150343
ENSG00000203286	Metazoa_SRP	0.180483	1.00E-06	17.46150343
ENSG00000263241	RP11-109M19.4	0.180163	1.00E-06	17.45894323
ENSG00000231083	AC011747.6	0.180163	1.00E-06	17.45894323
ENSG00000278733	RP11-425D17.1	0.179845	1.00E-06	17.45639453
ENSG00000274505	Metazoa_SRP	0.179845	1.00E-06	17.45639453
ENSG00000281353	Metazoa_SRP	0.179845	1.00E-06	17.45639453
ENSG00000267501	RP11-108P20.2	0.179402	1.00E-06	17.45283645
ENSG00000235756	RP11-339A11.1	0.179369	1.00E-06	17.45257105
ENSG00000260391	RP11-71H17.7	0.17928	1.00E-06	17.45185503
ENSG00000260497	CTB-55P19.1	0.179212	1.00E-06	17.45130772
ENSG00000233538	AC017104.2	0.179212	1.00E-06	17.45130772
ENSG00000259258	RP11-325L12.3	0.179185	1.00E-06	17.45109035
ENSG00000240086	RP11-657O9.1	0.17908029	1.00E-06	17.45024703
ENSG00000278464	RP11-96A15.1	0.178785743	1.00E-06	17.44787217
ENSG00000184361	SPATA32	0.178763121	1.00E-06	17.44768961
ENSG00000267543	RP11-666A8.7	0.178584	1.00E-06	17.4462433
ENSG00000276568	Metazoa_SRP	0.178584	1.00E-06	17.4462433
ENSG00000249199	CTD-2139B15.5	0.178584	1.00E-06	17.4462433
ENSG00000225807	RP11-44M6.1	0.178584	1.00E-06	17.4462433
ENSG00000230366	DSCR9	0.178292276	1.00E-06	17.44388468
ENSG00000235151	AC114730.2	0.17827	1.00E-06	17.44370442

ENSG00000256286	RP11-983C2.3	0.177959	1.00E-06	17.44118537
ENSG00000250147	MORF4L2P1	0.177959	1.00E-06	17.44118537
ENSG00000226782	RP11-706D8.3	0.177277	1.00E-06	17.43564585
ENSG00000271427	RP11-188D8.1	0.17703	1.00E-06	17.43363434
ENSG00000237730	RP11-459A10.1	0.176885	1.00E-06	17.43245219
ENSG00000281331	AC016753.2	0.17644	1.00E-06	17.42881814
ENSG00000124102	PI3	0.176416	1.00E-06	17.42862189
ENSG00000264530	RN7SL25P	0.176179	1.00E-06	17.42668244
ENSG00000273473	LL09NC01-139C3.1	0.176179	1.00E-06	17.42668244
ENSG00000262227	RP5-1050D4.5	0.176136	1.00E-06	17.42633028
ENSG00000255533	RP11-326C3.4	0.176111	1.00E-06	17.4261255
ENSG00000227401	RPL37P1	0.175628	1.00E-06	17.42216334
ENSG00000272192	CTD-2532N20.1	0.175504	1.00E-06	17.42114439
ENSG00000231977	RP5-963E22.4	0.175004	1.00E-06	17.41702837
ENSG00000225521	AC005237.4	0.174813	1.00E-06	17.41545295
ENSG00000125695	RP11-51F16.8	0.174509	1.00E-06	17.41294192
ENSG00000259364	RP11-64K12.9	0.174302	1.00E-06	17.4112296
ENSG00000259519	RP11-519G16.2	0.174302	1.00E-06	17.4112296
ENSG00000244044	RN7SL735P	0.174302	1.00E-06	17.4112296
ENSG00000276396	Metazoa_SRP	0.174302	1.00E-06	17.4112296
ENSG00000228700	RP11-155G14.1	0.174302	1.00E-06	17.4112296
ENSG00000236021	RP11-195C7.1	0.174049	1.00E-06	17.409134
ENSG00000260329	RP11-412D9.4	0.173928	1.00E-06	17.40813068
ENSG00000241665	RN7SL418P	0.173792	1.00E-06	17.40700215
ENSG00000239932	RN7SL606P	0.173707	1.00E-06	17.40629637
ENSG00000223658	C1GALT1C1L	0.173707	1.00E-06	17.40629637
ENSG00000253429	KB-1090H4.2	0.173707	1.00E-06	17.40629637
ENSG00000234043	RP11-56M3.1	0.173526	1.00E-06	17.40479232
ENSG00000254197	RP11-10J21.5	0.173116	1.00E-06	17.40137954
ENSG00000230530	LIMD1-AS1	0.1729763	1.00E-06	17.40021486
ENSG00000224797	RP11-57C19.6	0.172855	1.00E-06	17.39920281
ENSG00000249650	RP11-310P5.1	0.17265	1.00E-06	17.39749081
ENSG00000229758	DYNLT3P2	0.17253	1.00E-06	17.39648772
ENSG00000248165	RP11-44F21.2	0.17253	1.00E-06	17.39648772
ENSG00000255395	CTD-2562J17.2	0.172167	1.00E-06	17.39344912
ENSG00000265093	RN7SL246P	0.171947	1.00E-06	17.39160442
ENSG00000241211	IQCJ-SCHIP1-AS1	0.171945986	1.00E-06	17.39159592
ENSG00000278719	MCM8-AS1	0.171813	1.00E-06	17.39047967
ENSG00000213916	RPL13P	0.171801	1.00E-06	17.39037891
ENSG00000263515	MIR548AN	0.171698	1.00E-06	17.38951371
ENSG00000231226	TRIM31-AS1	0.171552	1.00E-06	17.38828642
ENSG00000273481	RP11-126K1.9	0.171417	1.00E-06	17.38715067
ENSG00000226410	AC104058.1	0.171368	1.00E-06	17.38673821
ENSG00000266479	CTD-2515C13.1	0.171045	1.00E-06	17.38401641
ENSG00000232311	RP1-249I4.2	0.170929	1.00E-06	17.38303766
ENSG00000244176	RP11-810P12.1	0.170848	1.00E-06	17.38235383
ENSG00000241587	RN7SL482P	0.170792	1.00E-06	17.38188087
ENSG00000265894	RN7SL357P	0.170792	1.00E-06	17.38188087

ENSG00000265439	RN7SL811P	0.170792	1.00E-06	17.38188087
ENSG00000265170	RN7SL667P	0.170792	1.00E-06	17.38188087
ENSG00000249655	CTC-325J23.2	0.170581	1.00E-06	17.38009744
ENSG00000186743	TPI1P3	0.170572	1.00E-06	17.38002132
ENSG00000270419	CAHM	0.170412	1.00E-06	17.3786674
ENSG00000277452	RN7SL473P	0.170221	1.00E-06	17.37704951
ENSG00000242999	RN7SL239P	0.169654	1.00E-06	17.37223592
ENSG00000264169	RN7SL665P	0.169654	1.00E-06	17.37223592
ENSG00000280116	AC231657.1	0.169654	1.00E-06	17.37223592
ENSG00000225938	RP4-575N6.4	0.169233	1.00E-06	17.36865139
ENSG00000226276	AC093382.1	0.169206	1.00E-06	17.3684212
ENSG00000231888	MTND5P15	0.169168	1.00E-06	17.36809717
ENSG00000254678	RP11-109L13.5	0.16909	1.00E-06	17.36743182
ENSG00000201533	RN7SKP237	0.16909	1.00E-06	17.36743182
ENSG00000264092	RN7SL474P	0.16909	1.00E-06	17.36743182
ENSG00000243424	RN7SL107P	0.169055	1.00E-06	17.36713316
ENSG00000272798	CTA-390C10.9	0.168531	1.00E-06	17.36265446
ENSG00000266420	RN7SL118P	0.168531	1.00E-06	17.36265446
ENSG00000264503	RP11-856M7.7	0.168484	1.00E-06	17.36225207
ENSG00000275287	Metazoa_SRP	0.168484	1.00E-06	17.36225207
ENSG00000249098	RP11-39E3.4	0.168171	1.00E-06	17.35956942
ENSG00000252970	RNA5SP159	0.168007	1.00E-06	17.35816182
ENSG00000230500	MKX-AS1	0.167974257	1.00E-06	17.35788063
ENSG00000247934	RP11-967K21.1	0.167912069	1.00E-06	17.3573464
ENSG00000272735	RP11-467P9.1	0.167698	1.00E-06	17.35550596
ENSG00000221579	AC093162.1	0.167658	1.00E-06	17.3551618
ENSG00000227834	AP003385.2	0.167565	1.00E-06	17.35436131
ENSG00000243905	RN7SL679P	0.167422	1.00E-06	17.35312959
ENSG00000160539	PLPP7	0.167140452	1.00E-06	17.35070142
ENSG00000276682	MIR7114	0.166872	1.00E-06	17.34838237
ENSG00000201358	RN7SKP193	0.166328	1.00E-06	17.34367153
ENSG00000230286	AC013472.4	0.166328	1.00E-06	17.34367153
ENSG00000257449	RP11-603J24.4	0.166261	1.00E-06	17.34309027
ENSG00000252516	RNA5SP82	0.165965	1.00E-06	17.3405195
ENSG00000238195	CTA-503F6.2	0.165248	1.00E-06	17.33427329
ENSG00000264510	BX649553.3	0.165069	1.00E-06	17.33270968
ENSG00000224351	CDKN2AIPNLP3	0.164712	1.00E-06	17.32958614
ENSG00000226429	RP11-111F5.6	0.164712	1.00E-06	17.32958614
ENSG00000270993	RP11-1112G13.3	0.164433	1.00E-06	17.32714034
ENSG00000279304	RP11-573M3.6	0.164181	1.00E-06	17.32492765
ENSG00000273181	RP11-778D9.13	0.164181	1.00E-06	17.32492765
ENSG00000256884	RP11-64B16.3	0.164068	1.00E-06	17.32393436
ENSG00000234770	GULOP	0.163671	1.00E-06	17.3204392
ENSG00000225947	RP11-313E4.1	0.163595	1.00E-06	17.31976913
ENSG00000147573	TRIM55	0.163542457	1.00E-06	17.31930569
ENSG00000211698	TRGV4	0.163489	1.00E-06	17.31883404
ENSG00000280921	Metazoa_SRP	0.163324	1.00E-06	17.31737728
ENSG00000249928	UQCRBP3	0.163128	1.00E-06	17.31564491

ENSG00000279167	bP-2168N6.3	0.163001	1.00E-06	17.31452129
ENSG00000233303	XXYLT1-AS1	0.162963	1.00E-06	17.31418492
ENSG00000279250	RP11-46D1.2	0.162607	1.00E-06	17.31102984
ENSG00000171054	OR13H1	0.162517	1.00E-06	17.31023111
ENSG00000230914	AC004840.8	0.162399	1.00E-06	17.30918322
ENSG00000223751	AC116609.2	0.162243	1.00E-06	17.30779671
ENSG00000205869	KRTAP5-1	0.16209	1.00E-06	17.30643556
ENSG00000257735	RP11-370I10.6	0.161915	1.00E-06	17.30487712
ENSG00000226573	CTA-85E5.7	0.161575	1.00E-06	17.30184447
ENSG00000275773	RP11-343D24.2	0.161575	1.00E-06	17.30184447
ENSG00000263489	CTC-264K15.6	0.161297	1.00E-06	17.29936008
ENSG00000256448	RP11-809N8.4	0.161116163	1.00E-06	17.2977417
ENSG00000232413	RP11-343J18.2	0.161064	1.00E-06	17.29727454
ENSG00000222627	RNU2-37P	0.161027	1.00E-06	17.29694309
ENSG00000239820	RN7SL213P	0.160556	1.00E-06	17.29271705
ENSG00000248757	CTD-2193G5.1	0.160228	1.00E-06	17.28976676
ENSG00000223145	RN7SKP112	0.160051	1.00E-06	17.28817217
ENSG00000238076	MRPL48P1	0.160051	1.00E-06	17.28817217
ENSG00000271803	RP1-63M2.5	0.159206	1.00E-06	17.28053518
ENSG00000261737	RP4-612B15.3	0.158885	1.00E-06	17.2776234
ENSG00000203684	IBA57-AS1	0.158802796	1.00E-06	17.27687678
ENSG00000257999	RP11-61E11.2	0.158802	1.00E-06	17.27686956
ENSG00000233664	NDUFS5P3	0.158555	1.00E-06	17.27462385
ENSG00000243607	RPL35AP26	0.158555	1.00E-06	17.27462385
ENSG00000223458	LMO7DN-IT1	0.158555	1.00E-06	17.27462385
ENSG00000258544	RP11-750I4.2	0.158555	1.00E-06	17.27462385
ENSG00000230047	AC017104.4	0.158555	1.00E-06	17.27462385
ENSG00000235218	AC092638.2	0.158547	1.00E-06	17.27455105
ENSG00000106178	CCL24	0.158380454	1.00E-06	17.27303477
ENSG00000267110	CTD-2587H24.4	0.158259	1.00E-06	17.27192802
ENSG00000255741	RP11-757G1.5	0.158255	1.00E-06	17.27189156
ENSG00000249649	MRPS33P2	0.158063	1.00E-06	17.27014017
ENSG00000240160	RN7SL263P	0.157953	1.00E-06	17.26913581
ENSG00000262380	CTB-193M12.3	0.157849	1.00E-06	17.2681856
ENSG00000243675	RP11-379F4.1	0.157574	1.00E-06	17.26566998
ENSG00000254553	RP1-27O5.3	0.157448	1.00E-06	17.26451591
ENSG00000272002	RP11-557L19.1	0.157345	1.00E-06	17.26357181
ENSG00000257830	RP11-845M18.7	0.15733	1.00E-06	17.26343427
ENSG00000254883	RP11-655M14.14	0.157087	1.00E-06	17.26120427
ENSG00000226509	AC005588.2	0.157087	1.00E-06	17.26120427
ENSG00000232646	RP11-344N10.4	0.15697	1.00E-06	17.26012933
ENSG00000235100	RP11-80H5.9	0.156845	1.00E-06	17.25898001
ENSG00000274275	RP11-326K13.5	0.156604	1.00E-06	17.25676154
ENSG00000179580	RNF151	0.156123428	1.00E-06	17.25232752
ENSG00000222385	RN7SKP158	0.156123	1.00E-06	17.25232356
ENSG00000228568	AC006461.2	0.156123	1.00E-06	17.25232356
ENSG00000272825	LL21NC02-1C16.2	0.156123	1.00E-06	17.25232356
ENSG00000260953	RP11-426C22.6	0.156046	1.00E-06	17.25161185

ENSG00000280417	RP11-5O17.1	0.156044	1.00E-06	17.25159336
ENSG00000177363	LRRN4CL	0.155937	1.00E-06	17.25060376
ENSG00000265070	AC006115.1	0.155682	1.00E-06	17.24824262
ENSG00000258542	AC068831.11	0.155646	1.00E-06	17.24790898
ENSG00000250590	RP11-565A3.2	0.15525637	1.00E-06	17.24429293
ENSG00000217004	RP3-329A5.1	0.155171	1.00E-06	17.24349943
ENSG00000261308	FIGNL2	0.155003	1.00E-06	17.24193661
ENSG00000265222	RP11-466A19.8	0.15483	1.00E-06	17.24032551
ENSG00000250796	RP11-723O4.3	0.1547	1.00E-06	17.23911367
ENSG00000277583	ST7-AS1_2	0.154599	1.00E-06	17.23817146
ENSG00000235659	RP11-374M1.5	0.154582	1.00E-06	17.23801281
ENSG00000236646	LAMTOR5P1	0.154574	1.00E-06	17.23793815
ENSG00000257008	GPR142	0.154461	1.00E-06	17.23688309
ENSG00000254867	RP11-1167A19.6	0.154325	1.00E-06	17.23561227
ENSG00000228355	BX322559.3	0.1542307	1.00E-06	17.23473044
ENSG00000249407	IL20RB-AS1	0.153957	1.00E-06	17.23216794
ENSG00000252408	SNORD38	0.153386	1.00E-06	17.22680728
ENSG00000263895	RP11-110H1.9	0.153301	1.00E-06	17.22600758
ENSG00000270915	RP11-27M5.1	0.153301	1.00E-06	17.22600758
ENSG00000255301	RP11-624G17.3	0.153182	1.00E-06	17.22488725
ENSG00000222179	RN7SKP26	0.152863	1.00E-06	17.22187972
ENSG00000225455	TPI1P4	0.152797	1.00E-06	17.22125669
ENSG00000258451	RP11-903H12.3	0.152005	1.00E-06	17.21375925
ENSG00000240520	UOX	0.151962578	1.00E-06	17.21335657
ENSG00000224312	MCCD1P2	0.151929	1.00E-06	17.21303775
ENSG00000227704	RP1-266L20.4	0.151929	1.00E-06	17.21303775
ENSG00000212330	RNU6-244P	0.151709	1.00E-06	17.21094715
ENSG00000253082	AC007365.2	0.151477	1.00E-06	17.20873923
ENSG00000252949	AL136303.1	0.151477	1.00E-06	17.20873923
ENSG00000259508	RP11-661D19.3	0.151271678	1.00E-06	17.20678238
ENSG00000252578	RNU6-135P	0.151177	1.00E-06	17.20587914
ENSG00000273724	RP11-347C12.12	0.151027	1.00E-06	17.20444697
ENSG00000231548	OR55B1P	0.15058	1.00E-06	17.20017064
ENSG00000268598	VN1R80P	0.15058	1.00E-06	17.20017064
ENSG00000273063	RP11-334G22.1	0.15058	1.00E-06	17.20017064
ENSG00000204780	IGKV1OR9-1	0.150538	1.00E-06	17.19976818
ENSG00000279741	RP11-44F14.9	0.150137	1.00E-06	17.19592004
ENSG00000276831	CTD-2105E13.16	0.150137	1.00E-06	17.19592004
ENSG00000223640	RPL30P3	0.149784	1.00E-06	17.192524
ENSG00000277013	CTC-526N19.1	0.149695	1.00E-06	17.19166651
ENSG00000252629	AL050316.1	0.149695	1.00E-06	17.19166651
ENSG00000252648	AC078993.1	0.149695	1.00E-06	17.19166651
ENSG00000258757	RP11-841O20.2	0.149506	1.00E-06	17.18984386
ENSG00000255464	SEPT14P8	0.149321	1.00E-06	17.18805755
ENSG00000265559	RN7SL652P	0.149296	1.00E-06	17.18781599
ENSG00000226908	HIST1H2BPS3	0.149256	1.00E-06	17.1874294
ENSG00000229635	RP4-713B5.2	0.14911	1.00E-06	17.18601749
ENSG00000230240	RP11-241I20.1	0.14882	1.00E-06	17.1832089

ENSG00000268293	CTD-2623H2.7	0.14882	1.00E-06	17.1832089
ENSG00000281576	AC015923.2	0.148602	1.00E-06	17.18109401
ENSG00000275576	RP5-836N17.4	0.148585	1.00E-06	17.18092895
ENSG00000213657	RP11-346D6.4	0.148504	1.00E-06	17.18014227
ENSG00000207944	MIR574	0.148447	1.00E-06	17.17958841
ENSG00000196301	HLA-DRB9	0.148386	1.00E-06	17.17899546
ENSG00000253454	NDUFA5P2	0.147954	1.00E-06	17.17478918
ENSG00000149634	SPATA25	0.147915	1.00E-06	17.17440884
ENSG00000255478	RP11-867O8.5	0.147788	1.00E-06	17.17316961
ENSG00000255200	AP003068.18	0.147209851	1.00E-06	17.16751469
ENSG00000226398	AC013480.2	0.147099	1.00E-06	17.16642791
ENSG00000131055	COX4I2	0.146886	1.00E-06	17.16433737
ENSG00000275362	Metazoa_SRP	0.146327	1.00E-06	17.15883647
ENSG00000239809	RP11-51L5.2	0.146253	1.00E-06	17.15810669
ENSG00000230176	LINC01433	0.146044	1.00E-06	17.15604356
ENSG00000257070	RP11-667M19.4	0.145852	1.00E-06	17.15414564
ENSG00000230506	RP11-416N4.4	0.145834	1.00E-06	17.15396759
ENSG00000272418	RP11-762H8.4	0.145518	1.00E-06	17.15083809
ENSG00000264468	MIR4520-1	0.145418	1.00E-06	17.14984633
ENSG00000215998	MIR935	0.145418	1.00E-06	17.14984633
ENSG00000224132	AC112715.2	0.145418	1.00E-06	17.14984633
ENSG00000223174	RN7SKP17	0.145003	1.00E-06	17.14572322
ENSG00000277373	RP11-462D18.4	0.145003	1.00E-06	17.14572322
ENSG00000272432	RP3-465N24.6	0.144818	1.00E-06	17.14388141
ENSG00000231655	AC011742.3	0.144591	1.00E-06	17.14161823
ENSG00000242020	RN7SL68P	0.144479	1.00E-06	17.14050029
ENSG00000274373	RP13-714J12.1	0.144386	1.00E-06	17.13957134
ENSG00000235296	AC137723.5	0.144365	1.00E-06	17.13936149
ENSG00000267550	CTC-482H14.5	0.144156	1.00E-06	17.13727136
ENSG00000202046	Y_RNA	0.144131	1.00E-06	17.13702114
ENSG00000180447	GAS1	0.144029	1.00E-06	17.1359998
ENSG00000170890	PLA2G1B	0.143929257	1.00E-06	17.13500036
ENSG00000171433	GLOD5	0.143909359	1.00E-06	17.13480089
ENSG00000248293	CTC-281M20.3	0.143775	1.00E-06	17.13345331
ENSG00000261679	ATP5HP1	0.143769	1.00E-06	17.1333931
ENSG00000224906	RP1-102E24.9	0.14337	1.00E-06	17.12938365
ENSG00000242058	RPS4XP19	0.143284	1.00E-06	17.12851799
ENSG00000260962	LINC00557	0.143258	1.00E-06	17.12825618
ENSG00000281864	AC068152.1	0.143146	1.00E-06	17.12712783
ENSG00000213449	GAPDHP28	0.143098	1.00E-06	17.12664398
ENSG00000242182	RN7SL745P	0.142986	1.00E-06	17.12551437
ENSG00000276031	RN7SL197P	0.142986	1.00E-06	17.12551437
ENSG00000236262	SHANK2-AS2	0.142966	1.00E-06	17.12531256
ENSG00000232097	AC079781.8	0.142966	1.00E-06	17.12531256
ENSG00000216753	HMGA1P7	0.142955	1.00E-06	17.12520156
ENSG00000260588	RP11-930P14.2	0.142624	1.00E-06	17.12185725
ENSG00000250753	RP11-241F15.10	0.142566	1.00E-06	17.12127043
ENSG00000270655	RP1-20N2.7	0.142566	1.00E-06	17.12127043

ENSG00000274215	CTD-2313J17.6	0.142543	1.00E-06	17.12103767
ENSG00000220614	RP11-480N24.4	0.14245	1.00E-06	17.1200961
ENSG00000250473	DUTP7	0.142426	1.00E-06	17.11985301
ENSG00000257820	MRPS6P4	0.142367	1.00E-06	17.11925525
ENSG00000200336	RNA5SP333	0.142337	1.00E-06	17.11895121
ENSG00000201695	RNA5SP334	0.142337	1.00E-06	17.11895121
ENSG00000232915	AC097721.1	0.142168	1.00E-06	17.11723725
ENSG00000256682	TAS2R12	0.142035312	1.00E-06	17.11589012
ENSG00000245667	RP5-940J5.8	0.141783	1.00E-06	17.11332504
ENSG00000273582	RP11-426C22.7	0.141772	1.00E-06	17.1132131
ENSG00000234988	AC068538.4	0.141637446	1.00E-06	17.11184321
ENSG00000271046	RP11-138I18.2	0.141378	1.00E-06	17.10919811
ENSG00000270421	RP11-351O1.4	0.141378	1.00E-06	17.10919811
ENSG00000265948	RP11-296E23.2	0.140987	1.00E-06	17.10520262
ENSG00000260253	RP4-676L2.1	0.140964	1.00E-06	17.10496724
ENSG00000228063	LYPLAL1-AS1	0.140598	1.00E-06	17.10121655
ENSG00000224910	AC002115.5	0.140598	1.00E-06	17.10121655
ENSG00000234685	NUS1P2	0.140504	1.00E-06	17.10025168
ENSG00000258825	AL133168.3	0.140265	1.00E-06	17.09779554
ENSG00000258455	RP11-665C16.5	0.14021	1.00E-06	17.09722972
ENSG00000272425	RP11-363E6.4	0.140128	1.00E-06	17.09638573
ENSG00000265201	MIR4677	0.139965	1.00E-06	17.09470658
ENSG00000260379	RP11-483K5.2	0.13989	1.00E-06	17.09393331
ENSG00000231336	AC017006.3	0.139825	1.00E-06	17.0932628
ENSG00000231398	RP11-375N9.2	0.139441	1.00E-06	17.0892953
ENSG00000262769	RP11-1113L8.1	0.139441	1.00E-06	17.0892953
ENSG00000274159	AL133243.3	0.139061	1.00E-06	17.08535834
ENSG00000261970	RP11-342L8.2	0.138682	1.00E-06	17.08142102
ENSG00000197361	FBXL22	0.138674951	1.00E-06	17.08134769
ENSG00000107018	RLN1	0.138555656	1.00E-06	17.08010608
ENSG00000216906	RP11-350J20.9	0.138467	1.00E-06	17.07918266
ENSG00000196366	C9orf163	0.138466	1.00E-06	17.07917224
ENSG00000244535	RP1-181J22.1	0.138461	1.00E-06	17.07912015
ENSG00000241103	RP11-398J16.1	0.138389	1.00E-06	17.07836975
ENSG00000251072	RP11-434D11.4	0.138285	1.00E-06	17.07728515
ENSG00000274995	RP11-321F6.2	0.138219	1.00E-06	17.07659642
ENSG00000269009	SLC6A21P	0.13793	1.00E-06	17.07357675
ENSG00000234816	U91328.2	0.13793	1.00E-06	17.07357675
ENSG00000226045	AC009274.6	0.137557	1.00E-06	17.06967003
ENSG00000239718	HLTF-AS1	0.137053	1.00E-06	17.06437438
ENSG00000233685	OR6L1P	0.137028	1.00E-06	17.06411119
ENSG00000167807	CTD-2369P2.10	0.136920936	1.00E-06	17.06298353
ENSG00000259120	SMIM6	0.136848356	1.00E-06	17.06221858
ENSG00000229280	EEF1DP6	0.136818	1.00E-06	17.06189852
ENSG00000225936	RP11-501J20.5	0.136818	1.00E-06	17.06189852
ENSG00000236713	RP11-363I22.3	0.136451	1.00E-06	17.05802344
ENSG00000259453	RP11-815J21.1	0.136451	1.00E-06	17.05802344
ENSG00000241679	RP11-80H8.4	0.136450799	1.00E-06	17.05802132

ENSG00000272942	CTA-246H3.12	0.136268	1.00E-06	17.05608729
ENSG00000170369	CST2	0.136086	1.00E-06	17.05415913
ENSG00000272319	AL022345.7	0.135723	1.00E-06	17.0503057
ENSG00000230944	AC026202.5	0.135723	1.00E-06	17.0503057
ENSG00000279448	AC006026.2	0.135723	1.00E-06	17.0503057
ENSG00000234869	RP3-439F8.1	0.135655	1.00E-06	17.0495827
ENSG00000188646	RP11-361A23.2	0.135539	1.00E-06	17.04834851
ENSG00000236099	AC013403.13	0.135465	1.00E-06	17.04756063
ENSG00000232558	KCND3-IT1	0.135363	1.00E-06	17.04647392
ENSG00000204165	CXorf65	0.135361703	1.00E-06	17.0464601
ENSG00000225126	RP4-549F15.1	0.135347	1.00E-06	17.04630338
ENSG00000257169	RP11-117N2.2	0.135003	1.00E-06	17.04263194
ENSG00000235830	SRGAP3-AS4	0.135003	1.00E-06	17.04263194
ENSG00000253701	AL928768.3	0.134974	1.00E-06	17.042322
ENSG00000253291	TRBV7-7	0.134941	1.00E-06	17.04196923
ENSG00000241281	RP11-364M6.1	0.134646	1.00E-06	17.03881185
ENSG00000232891	RP11-136K14.1	0.134646	1.00E-06	17.03881185
ENSG00000249430	CTD-2231H16.1	0.134279	1.00E-06	17.03487417
ENSG00000238055	RP1-128O3.6	0.134266	1.00E-06	17.03473449
ENSG00000227186	RP11-77G23.5	0.133937	1.00E-06	17.03119503
ENSG00000232487	RASA3-IT1	0.133937	1.00E-06	17.03119503
ENSG00000280252	AC009060.3	0.133586	1.00E-06	17.02740929
ENSG00000234325	MRPS10P2	0.133586	1.00E-06	17.02740929
ENSG00000275157	RP11-762N20.1	0.133499	1.00E-06	17.02646941
ENSG00000243709	LEFTY1	0.133498242	1.00E-06	17.02646122
ENSG00000225498	AC002064.5	0.133317	1.00E-06	17.02450123
ENSG00000159723	AGRP	0.133236	1.00E-06	17.02362442
ENSG00000264272	CTD-2514K5.4	0.132831	1.00E-06	17.01923236
ENSG00000200741	RNA5SP161	0.132773	1.00E-06	17.01860227
ENSG00000119608	PROX2	0.132646965	1.00E-06	17.01723214
ENSG00000213231	TCL1B	0.132513492	1.00E-06	17.01577973
ENSG00000255100	RP11-21L23.3	0.132272949	1.00E-06	17.01315852
ENSG00000258753	RP11-794A8.1	0.132198	1.00E-06	17.01234083
ENSG00000226193	RP3-398G3.5	0.132198	1.00E-06	17.01234083
ENSG00000261396	CTD-2012K14.2	0.13206	1.00E-06	17.01083403
ENSG00000233981	RP3-380E11.2	0.131856	1.00E-06	17.0086037
ENSG00000281491	DNAJB5-AS1	0.131761	1.00E-06	17.00756388
ENSG00000259474	RP11-650L12.1	0.131547	1.00E-06	17.00521882
ENSG00000177261	ENSAP3	0.131444	1.00E-06	17.00408876
ENSG00000214326	RPL31P1	0.131345	1.00E-06	17.00300176
ENSG00000223485	LINC01615	0.131344846	1.00E-06	17.00300007
ENSG00000267986	AC130469.2	0.131176	1.00E-06	17.00114426
ENSG00000231221	LINC01593	0.131176	1.00E-06	17.00114426
ENSG00000232305	CLCP1	0.131176	1.00E-06	17.00114426
ENSG00000250603	CTC-228N24.2	0.131176	1.00E-06	17.00114426
ENSG00000255236	CTD-2655K5.1	0.131175901	1.00E-06	17.00114317
ENSG00000260185	RP11-432I5.6	0.131111	1.00E-06	17.0004292
ENSG00000238604	Y_RNA	0.13102	1.00E-06	16.99942753

ENSG00000280346	CH507-152C13.4	0.130993	1.00E-06	16.99913019
ENSG00000259346	RP11-349G13.2	0.130503	1.00E-06	16.99372345
ENSG00000251604	LINC01385	0.130503	1.00E-06	16.99372345
ENSG00000248926	RP11-777B9.1	0.130336	1.00E-06	16.9918761
ENSG00000266897	AC005546.2	0.130169	1.00E-06	16.99002638
ENSG00000231990	RP11-53B5.1	0.130169	1.00E-06	16.99002638
ENSG00000232504	ST3GAL5-AS1	0.130003	1.00E-06	16.98818539
ENSG00000232777	MRPL51P2	0.129948	1.00E-06	16.9875749
ENSG00000229918	DOCK9-AS1	0.129837	1.00E-06	16.98634205
ENSG00000258843	RP11-286O18.1	0.129837	1.00E-06	16.98634205
ENSG00000265845	RP11-20B24.5	0.129794868	1.00E-06	16.98587382
ENSG00000258917	ZMYND19P1	0.129343	1.00E-06	16.98084245
ENSG00000233733	H2AFZP6	0.129179	1.00E-06	16.97901203
ENSG00000275696	Metazoa_SRP	0.129085	1.00E-06	16.97796184
ENSG00000254222	RP11-787D18.2	0.128884	1.00E-06	16.97571365
ENSG00000237073	RP11-168K11.2	0.128851	1.00E-06	16.97534421
ENSG00000249171	RP11-767N15.1	0.128688	1.00E-06	16.973518
ENSG00000230697	SAP18P3	0.128526	1.00E-06	16.97170071
ENSG00000249945	RP11-598D14.1	0.128526	1.00E-06	16.97170071
ENSG00000261537	RP11-319G9.5	0.128363	1.00E-06	16.96986989
ENSG00000147223	RIPPLY1	0.128309378	1.00E-06	16.9692671
ENSG00000241853	RP11-147K6.1	0.128222	1.00E-06	16.96828429
ENSG00000236427	RP11-107M16.2	0.128202	1.00E-06	16.96805924
ENSG00000276662	RP11-631M21.7	0.127979	1.00E-06	16.96554757
ENSG00000274444	RP11-128A17.2	0.12788	1.00E-06	16.96443112
ENSG00000273557	CTD-2024I7.18	0.12762	1.00E-06	16.96149491
ENSG00000274569	RP11-121C6.4	0.127559	1.00E-06	16.96080517
ENSG00000253515	RP11-417F21.2	0.127559	1.00E-06	16.96080517
ENSG00000255557	RP11-770G2.2	0.127241	1.00E-06	16.95720409
ENSG00000255233	RP11-755E23.3	0.127241	1.00E-06	16.95720409
ENSG00000261033	RP11-209D14.2	0.127241	1.00E-06	16.95720409
ENSG00000280624	AL162381.1	0.127241	1.00E-06	16.95720409
ENSG00000267892	CTD-2540F13.2	0.126919	1.00E-06	16.95354853
ENSG00000227589	RP5-1092A11.5	0.126903	1.00E-06	16.95336665
ENSG00000255329	H2AFZP4	0.126903	1.00E-06	16.95336665
ENSG00000214035	AC073310.4	0.126871	1.00E-06	16.95300281
ENSG00000259883	EHD4-AS1	0.126746	1.00E-06	16.95158069
ENSG00000273668	RP11-15E1.4	0.126115	1.00E-06	16.94438035
ENSG00000230570	RP11-69L16.6	0.126077	1.00E-06	16.94394559
ENSG00000235088	RP4-620E11.4	0.126059	1.00E-06	16.9437396
ENSG00000261144	RP11-102D18.1	0.12598	1.00E-06	16.94283519
ENSG00000276746	Metazoa_SRP	0.12572	1.00E-06	16.93985465
ENSG00000269533	AC003002.6	0.125668765	1.00E-06	16.93926659
ENSG00000237705	AC008937.2	0.125412	1.00E-06	16.93631587
ENSG00000276953	TRBV12-4	0.12536	1.00E-06	16.93571756
ENSG00000243023	UBA52P3	0.125242	1.00E-06	16.93435893
ENSG00000168703	WFDC12	0.125216	1.00E-06	16.93405939
ENSG00000229384	HMGB1P16	0.124413	1.00E-06	16.92477772

ENSG00000275493	FAM27D1	0.12438	1.00E-06	16.924395
ENSG00000248884	CTC-537E7.3	0.124137	1.00E-06	16.92157366
ENSG00000211721	TRBV6-5	0.124137	1.00E-06	16.92157366
ENSG00000275714	HIST1H3A	0.123836	1.00E-06	16.91807125
ENSG00000251059	RP11-100N20.1	0.123834742	1.00E-06	16.9180566
ENSG00000164556	FAM183BP	0.12381	1.00E-06	16.91776832
ENSG00000201746	RNU6-828P	0.123673	1.00E-06	16.91617104
ENSG00000253696	KBTBD11-OT1	0.123649	1.00E-06	16.91589105
ENSG00000271904	CTC-498M16.4	0.123534	1.00E-06	16.91454864
ENSG00000270598	RP11-396C23.3	0.123476	1.00E-06	16.91387113
ENSG00000280277	RP1-72A23.4	0.123474	1.00E-06	16.91384776
ENSG00000234281	LANCL1-AS1	0.123269768	1.00E-06	16.9114595
ENSG00000233996	AC013439.4	0.123235	1.00E-06	16.91105253
ENSG00000231098	AC008154.4	0.122938	1.00E-06	16.90757139
ENSG00000254236	KB-1639H6.2	0.122938	1.00E-06	16.90757139
ENSG00000262529	CTA-276F8.2	0.122642	1.00E-06	16.9040936
ENSG00000185631	AC017079.3	0.122642	1.00E-06	16.9040936
ENSG00000254693	RP11-58K22.5	0.122608	1.00E-06	16.90369359
ENSG00000279690	AP000280.66	0.122346	1.00E-06	16.90060741
ENSG00000213438	YBX2P1	0.121883	1.00E-06	16.89513739
ENSG00000257696	RP11-175P13.2	0.121761	1.00E-06	16.89369259
ENSG00000267011	CTB-50L17.16	0.121616075	1.00E-06	16.89197441
ENSG00000276991	MIAT_exon5_3	0.121502	1.00E-06	16.89062054
ENSG00000225945	RP3-441A12.1	0.121471	1.00E-06	16.8902524
ENSG00000250173	RP11-661C8.3	0.121182	1.00E-06	16.8868159
ENSG00000166800	LDHAL6A	0.120975473	1.00E-06	16.88435506
ENSG00000234050	LL0XNC01-237H1.3	0.120942	1.00E-06	16.88395582
ENSG00000226454	RP3-462C17.1	0.120894	1.00E-06	16.88338312
ENSG00000254984	FTLP6	0.120607	1.00E-06	16.87995412
ENSG00000264072	RP11-856M7.4	0.120607	1.00E-06	16.87995412
ENSG00000267643	RP11-64C12.4	0.120607	1.00E-06	16.87995412
ENSG00000235701	PCBP2P1	0.120512	1.00E-06	16.87881728
ENSG00000224471	MTCO3P19	0.120502	1.00E-06	16.87869757
ENSG00000268144	NIFKP6	0.120464	1.00E-06	16.87824254
ENSG00000266590	AC090692.1	0.120249	1.00E-06	16.87566537
ENSG00000270426	RP11-326I11.5	0.120192	1.00E-06	16.87498135
ENSG00000259224	SLC35G6	0.119967	1.00E-06	16.87227808
ENSG00000225203	RP11-182M20.2	0.119756	1.00E-06	16.86973841
ENSG00000225712	ATP5G2P1	0.119475	1.00E-06	16.86634924
ENSG00000255260	CTD-3224I3.3	0.119475	1.00E-06	16.86634924
ENSG00000224418	STK24-AS1	0.119475	1.00E-06	16.86634924
ENSG00000273464	RP11-313P22.1	0.119475	1.00E-06	16.86634924
ENSG00000281735	Clostridiales-1	0.119385	1.00E-06	16.86526206
ENSG00000267394	CTB-175E5.7	0.119076	1.00E-06	16.86152314
ENSG00000228877	RP11-473E2.4	0.118916	1.00E-06	16.85958332
ENSG00000232526	VDAC1P13	0.118501	1.00E-06	16.85453971
ENSG00000236463	LINC00427	0.118363	1.00E-06	16.85285864
ENSG00000250282	RP5-875H18.4	0.118363	1.00E-06	16.85285864

ENSG00000222033	LINC01124	0.118285	1.00E-06	16.85190761
ENSG00000231840	AC073342.12	0.118116105	1.00E-06	16.84984617
ENSG00000188389	PDCD1	0.117971	1.00E-06	16.84807273
ENSG00000236894	RP11-128B16.3	0.117816	1.00E-06	16.84617595
ENSG00000256681	CCDC58P5	0.117816	1.00E-06	16.84617595
ENSG00000228569	AC073133.2	0.117816	1.00E-06	16.84617595
ENSG00000228030	AC019084.7	0.117816	1.00E-06	16.84617595
ENSG00000244211	PDZK1P1	0.117712	1.00E-06	16.84490188
ENSG00000109163	GNRHR	0.117598	1.00E-06	16.843504
ENSG00000269903	RP11-571M6.18	0.117543	1.00E-06	16.8428291
ENSG00000227213	SPATA13-AS1	0.117543	1.00E-06	16.8428291
ENSG00000254061	RP11-359B20.1	0.117543	1.00E-06	16.8428291
ENSG00000235806	RP4-646N3.1	0.117462	1.00E-06	16.84183458
ENSG00000228549	RP11-108M9.3	0.117377979	1.00E-06	16.84080224
ENSG00000266934	RP11-332H18.3	0.11731994	1.00E-06	16.84008871
ENSG00000254271	RP11-131N11.4	0.117272	1.00E-06	16.83949907
ENSG00000233377	MTND4P20	0.117138	1.00E-06	16.83784964
ENSG00000224079	AC091729.7	0.117138	1.00E-06	16.83784964
ENSG00000196390	CTD-2503O16.3	0.117054	1.00E-06	16.83681471
ENSG00000229531	RP1-102G20.5	0.117009605	1.00E-06	16.83626743
ENSG00000278919	AP006285.2	0.116949	1.00E-06	16.83552
ENSG00000272630	RP11-344N10.5	0.116867	1.00E-06	16.83450808
ENSG00000219992	RP11-420L9.2	0.116735	1.00E-06	16.83287766
ENSG00000278075	RP11-248M19.1	0.116667	1.00E-06	16.83203702
ENSG00000189238	LINC00943	0.116438899	1.00E-06	16.82921358
ENSG00000221989	OR2A2	0.116224	1.00E-06	16.82654849
ENSG00000254231	CTD-2284J15.1	0.116212661	1.00E-06	16.82640773
ENSG00000267695	RP11-1030E3.1	0.116201	1.00E-06	16.82626296
ENSG00000235237	RP1-151B14.6	0.116047	1.00E-06	16.8243497
ENSG00000228323	AC008753.6	0.115936	1.00E-06	16.82296909
ENSG00000257438	RP11-114F10.3	0.115712	1.00E-06	16.82017896
ENSG00000212534	SNORD70	0.115673	1.00E-06	16.81969263
ENSG00000227161	AC092755.4	0.115435	1.00E-06	16.81672119
ENSG00000267004	CTD-2659N19.4	0.115435	1.00E-06	16.81672119
ENSG00000276486	RP11-210K20.6	0.115411	1.00E-06	16.81642121
ENSG00000267088	RP11-115N12.1	0.115411	1.00E-06	16.81642121
ENSG00000267117	AC010525.4	0.115411	1.00E-06	16.81642121
ENSG00000272417	CTD-2199O4.6	0.115411	1.00E-06	16.81642121
ENSG00000274447	LLNLR-304G9.1	0.115364	1.00E-06	16.81583357
ENSG00000275793	RIMBP3	0.115242	1.00E-06	16.81430708
ENSG00000259950	RP11-170L3.4	0.11515	1.00E-06	16.81315489
ENSG00000278546	RP1-41C23.4	0.11515	1.00E-06	16.81315489
ENSG00000279098	RP11-15B17.4	0.11515	1.00E-06	16.81315489
ENSG00000229312	RP11-470P21.2	0.11515	1.00E-06	16.81315489
ENSG00000163632	C3orf49	0.114790237	1.00E-06	16.80864041
ENSG00000272707	RP11-534C12.1	0.114516	1.00E-06	16.80518966
ENSG00000256458	RP11-363J17.1	0.114427	1.00E-06	16.80406798
ENSG00000268521	VN1R83P	0.114374	1.00E-06	16.8033996

ENSG00000229276	REV3L-IT1	0.114202	1.00E-06	16.80122839
ENSG00000232448	RP11-416N4.1	0.114117	1.00E-06	16.8001542
ENSG00000204889	KRT40	0.113916119	1.00E-06	16.79761237
ENSG00000269444	CTB-180A7.6	0.113862	1.00E-06	16.79692682
ENSG00000168530	MYL1	0.113777235	1.00E-06	16.79585241
ENSG00000280998	AC091057.1	0.113768	1.00E-06	16.7957353
ENSG00000280902	AC026951.1	0.113768	1.00E-06	16.7957353
ENSG00000236535	RC3H1-IT1	0.113607	1.00E-06	16.79369221
ENSG00000270352	RP11-111N20.4	0.113607	1.00E-06	16.79369221
ENSG00000259033	RP11-718G2.5	0.113607	1.00E-06	16.79369221
ENSG00000253991	KB-1562D12.2	0.113537	1.00E-06	16.792803
ENSG00000267036	AC005559.3	0.113362	1.00E-06	16.79057759
ENSG00000251200	RP11-6E9.5	0.113355	1.00E-06	16.7904885
ENSG00000236703	MYB-AS1	0.113355	1.00E-06	16.7904885
ENSG00000236168	RP5-831C21.1	0.113103	1.00E-06	16.78727767
ENSG00000252373	RNU6-358P	0.113103	1.00E-06	16.78727767
ENSG00000236913	AC025750.5	0.113103	1.00E-06	16.78727767
ENSG00000242135	RPL17P2	0.112852	1.00E-06	16.78407246
ENSG00000249365	RP11-508M8.1	0.112852	1.00E-06	16.78407246
ENSG00000231875	RP11-346E8.1	0.112727	1.00E-06	16.78247358
ENSG00000223525	RABGAP1L-IT1	0.112589	1.00E-06	16.78070636
ENSG00000240179	RPL26P3	0.112353	1.00E-06	16.77767912
ENSG00000270538	RP11-3B7.7	0.112271	1.00E-06	16.7766258
ENSG00000266313	RP11-173M1.4	0.112106	1.00E-06	16.77450397
ENSG00000188487	INSC	0.112045918	1.00E-06	16.77373057
ENSG00000280232	RP11-321N4.4	0.112004	1.00E-06	16.77319073
ENSG00000263360	RN7SL134P	0.111983	1.00E-06	16.77292021
ENSG00000213149	CNN2P9	0.111983	1.00E-06	16.77292021
ENSG00000272555	RP11-459I19.1	0.11186	1.00E-06	16.77133471
ENSG00000278966	RP5-1174N9.2	0.111737	1.00E-06	16.76974747
ENSG00000227067	DPPA3P1	0.111559	1.00E-06	16.76744738
ENSG00000275673	RP11-876N24.7	0.111405	1.00E-06	16.76545446
ENSG00000278472	RP11-74D7.3	0.11137	1.00E-06	16.76500114
ENSG00000271275	AC007326.9	0.111127	1.00E-06	16.76184986
ENSG00000225051	HMGB3P22	0.111046347	1.00E-06	16.7608024
ENSG00000254776	AC084121.17	0.110905	1.00E-06	16.75896488
ENSG00000268307	CTD-2619J13.13	0.110885	1.00E-06	16.75870469
ENSG00000250324	MRPL22P1	0.110716	1.00E-06	16.7565042
ENSG00000258177	RP11-394J1.2	0.110703	1.00E-06	16.75633479
ENSG00000226036	RP11-338C15.2	0.110644	1.00E-06	16.75556569
ENSG00000248211	TRPC7-AS1	0.110644	1.00E-06	16.75556569
ENSG00000230479	AP000695.6	0.11057	1.00E-06	16.75460048
ENSG00000228708	AL109763.1	0.110404	1.00E-06	16.75243292
ENSG00000272128	KB-1836B5.4	0.110404	1.00E-06	16.75243292
ENSG00000230147	RP11-305L7.5	0.110404	1.00E-06	16.75243292
ENSG00000221785	AC120045.2	0.110378	1.00E-06	16.75209312
ENSG00000228247	UBBP2	0.110165	1.00E-06	16.74930642
ENSG00000272966	RP11-686O6.1	0.110165	1.00E-06	16.74930642

ENSG00000272360	RP11-359I18.5	0.110165	1.00E-06	16.74930642
ENSG00000249302	FTH1P24	0.110165	1.00E-06	16.74930642
ENSG00000225613	LINCMD1	0.110165	1.00E-06	16.74930642
ENSG00000266368	RP11-1096G20.5	0.109927	1.00E-06	16.74618626
ENSG00000213148	AC073465.1	0.10969	1.00E-06	16.74307248
ENSG00000229625	RP11-44N12.2	0.10969	1.00E-06	16.74307248
ENSG00000255239	TREHP1	0.109454	1.00E-06	16.73996515
ENSG00000255565	RP11-6B19.1	0.109454	1.00E-06	16.73996515
ENSG00000237382	RPL21P121	0.109454	1.00E-06	16.73996515
ENSG00000243089	RP11-779P15.2	0.109454	1.00E-06	16.73996515
ENSG00000255108	AP006621.8	0.109313596	1.00E-06	16.73811332
ENSG00000254224	KB-1043D8.6	0.109254	1.00E-06	16.73732657
ENSG00000258576	CTD-2644I21.1	0.109219	1.00E-06	16.73686433
ENSG00000187763	OR2B7P	0.108985	1.00E-06	16.73377006
ENSG00000268187	AC118758.1	0.108753	1.00E-06	16.73069567
ENSG00000233451	RP11-301N24.3	0.108521	1.00E-06	16.72761472
ENSG00000230515	AC092580.3	0.108521	1.00E-06	16.72761472
ENSG00000272312	RP11-239L20.6	0.108486	1.00E-06	16.72714935
ENSG00000255085	AF186192.5	0.108457786	1.00E-06	16.7267741
ENSG00000228141	AC105339.1	0.108405051	1.00E-06	16.72607246
ENSG00000269993	AF003625.3	0.10829	1.00E-06	16.7245405
ENSG00000135898	GPR55	0.108107739	1.00E-06	16.72211028
ENSG00000200040	Y_RNA	0.108098	1.00E-06	16.72198031
ENSG00000254695	RP11-396O20.1	0.10806	1.00E-06	16.72147306
ENSG00000249312	ARL2BPP4	0.10806	1.00E-06	16.72147306
ENSG00000271607	RP3-418A9.3	0.108011	1.00E-06	16.72081872
ENSG00000161798	AQP5	0.108002308	1.00E-06	16.72070261
ENSG00000263271	RP11-1055B8.8	0.107917	1.00E-06	16.71956262
ENSG00000236732	AC094019.4	0.107831	1.00E-06	16.71841247
ENSG00000235002	RP11-157D18.2	0.107689	1.00E-06	16.71651137
ENSG00000256189	RP11-167N4.5	0.107687	1.00E-06	16.71648457
ENSG00000105261	OVOL3	0.107651499	1.00E-06	16.71600889
ENSG00000262202	RP11-160E2.6	0.107545	1.00E-06	16.71458093
ENSG00000242318	RP11-810D13.1	0.107376	1.00E-06	16.71231204
ENSG00000267762	RP11-426J5.2	0.107265	1.00E-06	16.71081988
ENSG00000227225	MTND1P14	0.10715	1.00E-06	16.70927232
ENSG00000200095	RNU6-181P	0.107025	1.00E-06	16.70758831
ENSG00000262609	BTF3P14	0.106925	1.00E-06	16.70623968
ENSG00000238267	RP11-241K18.2	0.106925	1.00E-06	16.70623968
ENSG00000250252	RP11-342A1.1	0.106925	1.00E-06	16.70623968
ENSG00000230899	MAGEA8-AS1	0.106925	1.00E-06	16.70623968
ENSG00000243020	RPL7P39	0.106718	1.00E-06	16.70344401
ENSG00000233645	RP11-431K24.4	0.106701	1.00E-06	16.70321417
ENSG00000234896	OR7E62P	0.106701	1.00E-06	16.70321417
ENSG00000273216	AC002059.10	0.106701	1.00E-06	16.70321417
ENSG00000203402	RP11-571E6.3	0.106701	1.00E-06	16.70321417
ENSG00000265759	AC020934.1	0.10664	1.00E-06	16.70238916
ENSG00000237807	RP11-400K9.4	0.106611	1.00E-06	16.70199678

ENSG00000204511	MCCD1	0.106589	1.00E-06	16.70169903
ENSG00000233492	RP5-984P4.4	0.106553	1.00E-06	16.70121169
ENSG00000282915	RP11-205A8.4	0.106255	1.00E-06	16.69717121
ENSG00000275527	CTD-3154N5.2	0.106255	1.00E-06	16.69717121
ENSG00000258737	SUB1P2	0.106248	1.00E-06	16.69707616
ENSG00000228863	RP11-404F10.2	0.106169932	1.00E-06	16.69601571
ENSG00000235787	RP11-219I21.1	0.106034	1.00E-06	16.69416742
ENSG00000240452	MTCO1P29	0.106034	1.00E-06	16.69416742
ENSG00000168148	HIST3H3	0.105813	1.00E-06	16.69115736
ENSG00000242837	RPL21P13	0.105813	1.00E-06	16.69115736
ENSG00000224384	RP3-417O22.3	0.105813	1.00E-06	16.69115736
ENSG00000267304	AC004637.1	0.105593	1.00E-06	16.68815467
ENSG00000236854	AL121656.5	0.105593	1.00E-06	16.68815467
ENSG00000171659	GPR34	0.105593	1.00E-06	16.68815467
ENSG00000242209	AC006445.6	0.105563	1.00E-06	16.68774473
ENSG00000260394	LA16c-313D11.9	0.105467755	1.00E-06	16.68644246
ENSG00000278953	RP11-178H8.2	0.105376	1.00E-06	16.6851868
ENSG00000280186	RP11-483I13.6	0.105242	1.00E-06	16.68335105
ENSG00000228085	AC004920.2	0.105076	1.00E-06	16.68107366
ENSG00000243503	AC090453.1	0.105061	1.00E-06	16.6808677
ENSG00000231561	CEACAMP5	0.104865	1.00E-06	16.67817372
ENSG00000232139	LINC00867	0.104832315	1.00E-06	16.67772398
ENSG00000254607	RP11-115C10.1	0.104725037	1.00E-06	16.67624687
ENSG00000275476	RP11-996F15.4	0.104725	1.00E-06	16.67624636
ENSG00000218073	RP1-13D10.2	0.104725	1.00E-06	16.67624636
ENSG00000237810	CTD-2571E19.3	0.104653	1.00E-06	16.67525414
ENSG00000274737	RP5-1057I20.6	0.104554	1.00E-06	16.67388873
ENSG00000267000	RP11-27G24.3	0.104509	1.00E-06	16.67326766
ENSG00000198483	ANKRD35	0.104425823	1.00E-06	16.67211899
ENSG00000272562	RP11-396C23.4	0.10387	1.00E-06	16.66441951
ENSG00000224256	CTA-292E10.7	0.10387	1.00E-06	16.66441951
ENSG00000229505	RP4-682E18.1	0.103729	1.00E-06	16.66245977
ENSG00000273821	RP5-963E22.6	0.103555	1.00E-06	16.66003769
ENSG00000254526	RP11-466I1.1	0.103448	1.00E-06	16.65854623
ENSG00000260550	RP11-457I16.2	0.103393611	1.00E-06	16.65778751
ENSG00000226133	RP4-683M8.2	0.103377846	1.00E-06	16.65756752
ENSG00000178596	GAPDHP29	0.103343	1.00E-06	16.65708114
ENSG00000227568	SNX18P26	0.10334	1.00E-06	16.65703926
ENSG00000253737	KB-1460A1.3	0.103299	1.00E-06	16.65646676
ENSG00000256209	RP11-897M7.1	0.103238	1.00E-06	16.65561457
ENSG00000262038	RP11-177N22.3	0.103238	1.00E-06	16.65561457
ENSG00000265254	CTD-2350C19.2	0.103238	1.00E-06	16.65561457
ENSG00000250417	CTD-2194D22.2	0.103238	1.00E-06	16.65561457
ENSG00000249085	CTD-2631K10.1	0.103238	1.00E-06	16.65561457
ENSG00000215785	CFL1P6	0.103029	1.00E-06	16.65269095
ENSG00000232622	RP11-484D4.3	0.103029	1.00E-06	16.65269095
ENSG00000281508	CDR1-AS	0.102821	1.00E-06	16.64977542
ENSG00000252234	AC006557.1	0.102726	1.00E-06	16.64844185

ENSG00000255124	RP11-680F20.5	0.102613	1.00E-06	16.64685399
ENSG00000187135	VSTM2B	0.102613	1.00E-06	16.64685399
ENSG00000270972	RP11-326C3.15	0.102407	1.00E-06	16.64395481
ENSG00000255143	RP11-805J14.3	0.102407	1.00E-06	16.64395481
ENSG00000183770	FOXL2	0.102246	1.00E-06	16.64168488
ENSG00000261481	RP11-77H9.6	0.102202	1.00E-06	16.6410639
ENSG00000225867	RP5-1177I5.3	0.102202	1.00E-06	16.6410639
ENSG00000233956	BTF3P6	0.101996	1.00E-06	16.63815305
ENSG00000226230	AC007237.2	0.101996	1.00E-06	16.63815305
ENSG00000213498	TPT1P13	0.101996	1.00E-06	16.63815305
ENSG00000256035	AP000619.6	0.101897	1.00E-06	16.63675205
ENSG00000231992	RP11-57H12.2	0.101792	1.00E-06	16.63526466
ENSG00000264265	RP11-14P20.1	0.101792	1.00E-06	16.63526466
ENSG00000269859	CTD-2537I9.16	0.101792	1.00E-06	16.63526466
ENSG00000244586	WNT5A-AS1	0.101792	1.00E-06	16.63526466
ENSG00000266960	FAM106DP	0.101589	1.00E-06	16.63238467
ENSG00000255038	RP11-1167A19.2	0.101387	1.00E-06	16.62951315
ENSG00000279313	CH507-145C22.4	0.101151	1.00E-06	16.62615106
ENSG00000236711	SMAD9-IT1	0.100984	1.00E-06	16.6237672
ENSG00000248725	RP11-218C23.1	0.100984	1.00E-06	16.6237672
ENSG00000184523	PTGER4P2	0.100984	1.00E-06	16.6237672
ENSG00000116771	AGMAT	0.100978	1.00E-06	16.62368148
ENSG00000216809	RP11-57K17.1	0.100824	1.00E-06	16.62147957
ENSG00000247130	RP11-366H4.3	0.100785	1.00E-06	16.62092141
ENSG00000119440	LCN1P1	0.100785	1.00E-06	16.62092141
ENSG00000235929	TPT1P14	0.100785	1.00E-06	16.62092141
ENSG00000259611	LINC01582	0.100697824	1.00E-06	16.61967298
ENSG00000167850	CD300C	0.100651	1.00E-06	16.61900198
ENSG00000267151	MIR2117HG	0.100585	1.00E-06	16.61805565
ENSG00000231294	AC006994.2	0.100585	1.00E-06	16.61805565
ENSG00000237422	RP11-305L7.3	0.100585	1.00E-06	16.61805565
ENSG00000177202	SPACA4	0.100535	1.00E-06	16.61733832
ENSG00000235071	RP11-759L5.2	0.100535	1.00E-06	16.61733832
ENSG00000274068	RP11-475E11.9	0.100513	1.00E-06	16.61702258
ENSG00000227805	RP11-248J23.5	0.100481	1.00E-06	16.6165632
ENSG00000235781	XXbac-BPG249D20.9	0.100476	1.00E-06	16.61649141
ENSG00000228278	ORM2	0.100453	1.00E-06	16.61616112
ENSG00000254620	RP5-907D15.3	0.100387	1.00E-06	16.61521293
ENSG00000271360	RP11-138I18.1	0.100359	1.00E-06	16.61481048
ENSG00000230234	RP1-276N6.2	0.100267	1.00E-06	16.61348734
ENSG00000268050	RP11-247A12.8	0.100242	1.00E-06	16.61312758
ENSG00000271584	RP11-89C3.4	0.100189	1.00E-06	16.61236459
ENSG00000273866	Metazoa_SRP	0.100189	1.00E-06	16.61236459
ENSG00000274664	RP11-169D4.4	0.099601	1.00E-06	16.60387261
ENSG00000260620	RP11-883G14.4	0.099601	1.00E-06	16.60387261
ENSG00000282317	RP11-173A6.3	0.099442	1.00E-06	16.60156769
ENSG00000257989	RP1-288H2.2	0.099406	1.00E-06	16.60104531
ENSG00000255507	RP11-535A19.2	0.099391057	1.00E-06	16.60082843

ENSG00000259709	CTD-2184D3.7	0.099277451	1.00E-06	16.59917846
ENSG00000183246	RIMBP3C	0.099208153	1.00E-06	16.59817107
ENSG00000273451	RP4-569M23.4	0.099162	1.00E-06	16.59749975
ENSG00000278663	MIR6887	0.099028	1.00E-06	16.59554888
ENSG00000271931	RP11-63L7.5	0.098965	1.00E-06	16.59463077
ENSG00000233033	CASK-AS1	0.098827	1.00E-06	16.59261763
ENSG00000121381	TAS2R9	0.098636	1.00E-06	16.58982667
ENSG00000274139	RP11-14C10.3	0.098636	1.00E-06	16.58982667
ENSG00000251378	AC096582.9	0.098636	1.00E-06	16.58982667
ENSG00000263177	MTND1P8	0.098578	1.00E-06	16.58897809
ENSG00000236366	RP11-440G9.1	0.098573623	1.00E-06	16.58891403
ENSG00000213744	RPS10P14	0.098346	1.00E-06	16.58557875
ENSG00000227432	AC053503.11	0.098066	1.00E-06	16.58146541
ENSG00000236691	NDUFA4P2	0.098037	1.00E-06	16.58103872
ENSG00000255451	RP11-58K22.1	0.097877	1.00E-06	16.57868226
ENSG00000271011	RP11-171I2.5	0.097877	1.00E-06	16.57868226
ENSG00000219993	RP11-288G3.3	0.097783	1.00E-06	16.57729605
ENSG00000237552	RP11-415A20.1	0.097702	1.00E-06	16.57610047
ENSG00000277858	H2AFB2	0.097502	1.00E-06	16.57314419
ENSG00000249192	MTND3P25	0.097434	1.00E-06	16.57213767
ENSG00000232140	AC073257.1	0.097316	1.00E-06	16.5703894
ENSG00000232010	AP001059.5	0.097316	1.00E-06	16.5703894
ENSG00000230370	RPL23AP52	0.097249	1.00E-06	16.56939579
ENSG00000276810	RP11-19G24.2	0.09713	1.00E-06	16.56762934
ENSG00000249840	GAPDHP76	0.096995	1.00E-06	16.56562276
ENSG00000249041	RP11-21G20.3	0.096945	1.00E-06	16.56487887
ENSG00000256902	IQSEC3P1	0.096854	1.00E-06	16.56352401
ENSG00000266835	GAPLINC	0.096853175	1.00E-06	16.56351172
ENSG00000233242	RP11-220I1.2	0.096834	1.00E-06	16.56322607
ENSG00000144227	NXPH2	0.096761	1.00E-06	16.56213806
ENSG00000249198	RP11-772C9.1	0.096761	1.00E-06	16.56213806
ENSG00000259056	DUXAP1	0.096735	1.00E-06	16.56175035
ENSG00000270792	RP11-103J8.1	0.096680797	1.00E-06	16.56094175
ENSG00000277000	DUSP12P1	0.096663	1.00E-06	16.56067615
ENSG00000230825	AC005532.5	0.096628106	1.00E-06	16.56015526
ENSG00000205693	MANSC4	0.096519	1.00E-06	16.55852535
ENSG00000187013	C17orf82	0.09647	1.00E-06	16.55779275
ENSG00000274594	RP11-43P8.2	0.096435	1.00E-06	16.55726923
ENSG00000275631	U1	0.096394	1.00E-06	16.55665573
ENSG00000202215	RNU1-51P	0.096394	1.00E-06	16.55665573
ENSG00000271849	CTC-332L22.1	0.096394	1.00E-06	16.55665573
ENSG00000277374	U1	0.096394	1.00E-06	16.55665573
ENSG00000130487	KLHDC7B	0.096313	1.00E-06	16.55544292
ENSG00000130055	GDPD2	0.096274199	1.00E-06	16.55486159
ENSG00000272395	IFNL4	0.095915182	1.00E-06	16.54947158
ENSG00000237646	HLCS-IT1	0.09585	1.00E-06	16.54849081
ENSG00000236391	AC092573.2	0.095778689	1.00E-06	16.54741707
ENSG00000250614	AC007078.4	0.095501	1.00E-06	16.54322822

ENSG00000225703	AC005522.7	0.095501	1.00E-06	16.54322822
ENSG00000236818	ARL5AP2	0.09549	1.00E-06	16.54306204
ENSG00000187581	COX8C	0.09549	1.00E-06	16.54306204
ENSG00000253263	KB-1507C5.3	0.09549	1.00E-06	16.54306204
ENSG00000227253	RP11-166N17.1	0.095379614	1.00E-06	16.54139332
ENSG00000250413	RP11-448G15.1	0.095361	1.00E-06	16.54111174
ENSG00000205882	DEFB134	0.095311	1.00E-06	16.54035511
ENSG00000234707	RP11-745C15.2	0.095232	1.00E-06	16.53915881
ENSG00000235786	ZNRF3-IT1	0.095147	1.00E-06	16.53787055
ENSG00000232349	YBX1P7	0.095133	1.00E-06	16.53765825
ENSG00000237281	CATIP-AS2	0.095133	1.00E-06	16.53765825
ENSG00000204818	ADGRF5P2	0.095002	1.00E-06	16.53567027
ENSG00000230054	RP11-402G3.5	0.094956082	1.00E-06	16.53497279
ENSG00000276957	RP11-29B2.6	0.094956	1.00E-06	16.53497154
ENSG00000231774	RP11-402P6.11	0.094852	1.00E-06	16.53339057
ENSG00000234451	RP11-211N8.6	0.09469	1.00E-06	16.53092445
ENSG00000254632	RP11-21L23.4	0.094603	1.00E-06	16.52959831
ENSG00000207575	MIR649	0.094447	1.00E-06	16.52721735
ENSG00000224475	AC073900.4	0.094447	1.00E-06	16.52721735
ENSG00000274898	AC001226.7	0.094427	1.00E-06	16.52691182
ENSG00000260052	CTC-527H23.3	0.094427	1.00E-06	16.52691182
ENSG00000227227	AC017101.10	0.094139	1.00E-06	16.52250491
ENSG00000235575	RP4-800F24.1	0.094047	1.00E-06	16.5210943
ENSG00000234928	AP000344.3	0.09399109	1.00E-06	16.52023637
ENSG00000270090	RP11-529E10.7	0.093827953	1.00E-06	16.51773017
ENSG00000225905	RP4-758J18.7	0.093731	1.00E-06	16.51623865
ENSG00000248624	RP11-5N11.2	0.093731	1.00E-06	16.51623865
ENSG00000272255	CTD-3224K15.3	0.093731	1.00E-06	16.51623865
ENSG00000232672	ACTG1P21	0.093649	1.00E-06	16.51497597
ENSG00000241631	RN7SL316P	0.093559	1.00E-06	16.51358882
ENSG00000240803	RN7SL231P	0.093559	1.00E-06	16.51358882
ENSG00000225330	AF064860.5	0.093559	1.00E-06	16.51358882
ENSG00000232363	RP1-102D24.5	0.093387	1.00E-06	16.51093411
ENSG00000260976	GS1-120K12.4	0.093302	1.00E-06	16.50962039
ENSG00000265739	RP11-354P11.3	0.093216	1.00E-06	16.50828999
ENSG00000257359	RP11-780K2.1	0.093195	1.00E-06	16.50796493
ENSG00000218757	RP3-472M2.2	0.093046	1.00E-06	16.50565651
ENSG00000272161	RP4-713A8.1	0.092914	1.00E-06	16.50360837
ENSG00000262429	RP5-1050D4.3	0.092894	1.00E-06	16.5032978
ENSG00000282033	RP11-506F3.1	0.092763	1.00E-06	16.50126186
ENSG00000258428	RP11-1085N6.2	0.09273872	1.00E-06	16.5008842
ENSG00000269489	RP11-98D18.17	0.092707	1.00E-06	16.50039066
ENSG00000257642	RP11-474B16.1	0.092707	1.00E-06	16.50039066
ENSG00000227680	AC108051.2	0.092707	1.00E-06	16.50039066
ENSG00000254864	CTD-2516F10.4	0.092538	1.00E-06	16.4977583
ENSG00000255400	RP13-631K18.5	0.092538	1.00E-06	16.4977583
ENSG00000255669	RP11-885B4.2	0.092538	1.00E-06	16.4977583
ENSG00000280360	AC092159.1	0.092538	1.00E-06	16.4977583

ENSG00000225284	AC018693.6	0.092538	1.00E-06	16.4977583
ENSG00000253604	RP11-489E7.1	0.092538	1.00E-06	16.4977583
ENSG00000132932	ATP8A2	0.092489702	1.00E-06	16.49700512
ENSG00000257373	RP11-547C5.2	0.092204	1.00E-06	16.49254172
ENSG00000249734	RP11-332J15.1	0.092204	1.00E-06	16.49254172
ENSG00000227192	RP1-45I4.3	0.092204	1.00E-06	16.49254172
ENSG00000188425	NANOS2	0.092148	1.00E-06	16.49166523
ENSG00000250060	RP11-332J15.2	0.092037	1.00E-06	16.48992634
ENSG00000225393	RP11-218L14.4	0.092037	1.00E-06	16.48992634
ENSG00000237452	BHMG1	0.091832	1.00E-06	16.48670935
ENSG00000216723	NUDT19P3	0.091705	1.00E-06	16.48471278
ENSG00000237471	AC073115.6	0.091705	1.00E-06	16.48471278
ENSG00000267066	RP11-866E20.1	0.091593	1.00E-06	16.48294972
ENSG00000266579	RP1-71H19.2	0.091466	1.00E-06	16.48094794
ENSG00000253652	RP11-798K23.4	0.091328	1.00E-06	16.47876962
ENSG00000272801	RP1-170O19.23	0.091262	1.00E-06	16.47772665
ENSG00000223643	RP11-108M9.1	0.091212007	1.00E-06	16.47693613
ENSG00000226438	RP11-420K8.1	0.091212	1.00E-06	16.47693602
ENSG00000267298	AC006116.19	0.091048	1.00E-06	16.47433971
ENSG00000229308	AC010084.1	0.091048	1.00E-06	16.47433971
ENSG00000276002	Metazoa_SRP	0.090886	1.00E-06	16.47177046
ENSG00000250192	RP11-300M6.1	0.090886	1.00E-06	16.47177046
ENSG00000230790	AC012456.4	0.090725	1.00E-06	16.46921253
ENSG00000187556	NANOS3	0.090665403	1.00E-06	16.46826451
ENSG00000271369	RP11-350D17.3	0.090563	1.00E-06	16.46663413
ENSG00000264015	RP11-176N18.2	0.090563	1.00E-06	16.46663413
ENSG00000269487	CTB-174O21.2	0.090563	1.00E-06	16.46663413
ENSG00000271219	RP11-369K17.2	0.090563	1.00E-06	16.46663413
ENSG00000258484	SPESP1	0.090495499	1.00E-06	16.46555841
ENSG00000231130	HLA-T	0.090482	1.00E-06	16.4653432
ENSG00000266100	RP11-118E18.2	0.090402	1.00E-06	16.46406707
ENSG00000232306	AC012485.2	0.090402	1.00E-06	16.46406707
ENSG00000226872	AC002472.11	0.090402	1.00E-06	16.46406707
ENSG00000249965	CDC42P4	0.090402	1.00E-06	16.46406707
ENSG00000250284	CTB-1I21.1	0.090241	1.00E-06	16.46149543
ENSG00000253182	RP11-486M23.2	0.090241	1.00E-06	16.46149543
ENSG00000254396	RP11-56F10.3	0.090241	1.00E-06	16.46149543
ENSG00000207815	MIR563	0.090196	1.00E-06	16.46077583
ENSG00000249068	CTC-287O8.1	0.090082	1.00E-06	16.45895124
ENSG00000261873	RP11-515E23.1	0.089922	1.00E-06	16.4563865
ENSG00000268583	CTC-453G23.8	0.089922	1.00E-06	16.4563865
ENSG00000244071	RPL9P33	0.089922	1.00E-06	16.4563865
ENSG00000255092	RP11-58K22.4	0.089764	1.00E-06	16.45384935
ENSG00000254664	CTD-2560E9.3	0.089764	1.00E-06	16.45384935
ENSG00000237951	PPIL1P1	0.089756	1.00E-06	16.45372076
ENSG00000251298	RP11-425A23.1	0.089606	1.00E-06	16.45130772
ENSG00000271173	RP11-272J7.6	0.089466	1.00E-06	16.44905189
ENSG00000234993	CUBNP2	0.08937	1.00E-06	16.447503

ENSG00000265943	RP11-739L10.1	0.089291	1.00E-06	16.44622715
ENSG00000283051	KB-51A8.1	0.089291	1.00E-06	16.44622715
ENSG00000232821	AC003986.6	0.089239	1.00E-06	16.44538673
ENSG00000267705	RP11-108P20.3	0.089212	1.00E-06	16.44495016
ENSG00000274996	CTB-58E17.2	0.089136	1.00E-06	16.4437206
ENSG00000243886	RP11-575C1.1	0.089136	1.00E-06	16.4437206
ENSG00000230215	EIF2B5-AS1	0.089136	1.00E-06	16.4437206
ENSG00000235108	IFNA12P	0.089136	1.00E-06	16.4437206
ENSG00000275077	AC012363.1	0.089068	1.00E-06	16.44261958
ENSG00000272076	RP11-11C20.3	0.08898	1.00E-06	16.44119348
ENSG00000254533	AF186192.1	0.08898	1.00E-06	16.44119348
ENSG00000258553	RP11-16B13.1	0.0889795	1.00E-06	16.44118537
ENSG00000174562	KLK15	0.08894065	1.00E-06	16.44055533
ENSG00000243655	RP11-846F4.12	0.088867	1.00E-06	16.43936017
ENSG00000172967	XKR3	0.088842	1.00E-06	16.43895425
ENSG00000270571	RP11-355F16.1	0.088824	1.00E-06	16.43866192
ENSG00000225336	HMGB3P1	0.088824	1.00E-06	16.43866192
ENSG00000278819	RP5-1099C19.2	0.088824	1.00E-06	16.43866192
ENSG00000240074	RPL9P30	0.088669	1.00E-06	16.43614218
ENSG00000264971	PRR13P4	0.088655	1.00E-06	16.43591438
ENSG00000255850	TMEM5-AS1	0.088594	1.00E-06	16.43492138
ENSG00000232188	RP11-312J18.6	0.088516	1.00E-06	16.43365064
ENSG00000203434	RP11-163F15.1	0.088516	1.00E-06	16.43365064
ENSG00000248468	RP11-517B11.4	0.088516	1.00E-06	16.43365064
ENSG00000225272	RP11-466F5.4	0.088421	1.00E-06	16.43210143
ENSG00000267598	CTC-250I14.6	0.088395714	1.00E-06	16.43168879
ENSG00000260417	CTD-2542L18.1	0.088387	1.00E-06	16.43154657
ENSG00000275614	RP11-12A20.12	0.088362	1.00E-06	16.43113845
ENSG00000250842	CTC-806A22.1	0.088348	1.00E-06	16.43090986
ENSG00000278367	RP11-42O4.2	0.08828	1.00E-06	16.42979901
ENSG00000250310	RP5-1029K10.4	0.088208	1.00E-06	16.42862189
ENSG00000226220	CICP22	0.088135	1.00E-06	16.42742743
ENSG00000232783	FRG2FP	0.088131699	1.00E-06	16.42737339
ENSG00000231210	LINC01510	0.08807777	1.00E-06	16.42649033
ENSG00000249320	RP11-206P5.1	0.088056	1.00E-06	16.42613369
ENSG00000231690	LINC00574	0.087828	1.00E-06	16.42239333
ENSG00000272249	KB-1958F4.2	0.087785	1.00E-06	16.42168682
ENSG00000213731	RAB5CP1	0.087752	1.00E-06	16.42114439
ENSG00000272545	CTC-527H23.4	0.087752	1.00E-06	16.42114439
ENSG00000237077	AC105399.2	0.087668397	1.00E-06	16.41976925
ENSG00000259377	CTD-2308G16.1	0.087602	1.00E-06	16.41867619
ENSG00000268993	RP11-439A17.5	0.087451	1.00E-06	16.41618726
ENSG00000277383	CTD-3001H11.2	0.087451	1.00E-06	16.41618726
ENSG00000225770	AC092933.3	0.087307	1.00E-06	16.41380971
ENSG00000268074	RPL22P5	0.087226	1.00E-06	16.41247061
ENSG00000230107	CTA-126B4.7	0.087199	1.00E-06	16.41202397
ENSG00000177452	RP4-597J3.1	0.087151	1.00E-06	16.4112296
ENSG00000224361	AC011239.1	0.087151	1.00E-06	16.4112296

ENSG00000254019	RP11-10J21.3	0.087151	1.00E-06	16.4112296
ENSG00000250740	RP11-710F7.2	0.087150839	1.00E-06	16.41122693
ENSG00000143512	HHIPL2	0.086967137	1.00E-06	16.40818272
ENSG00000257271	KIRREL3-AS1	0.086853	1.00E-06	16.40628806
ENSG00000254616	RP11-700F16.2	0.086853	1.00E-06	16.40628806
ENSG00000278107	RP11-162A12.4	0.086853	1.00E-06	16.40628806
ENSG00000250928	CTB-161M19.2	0.086853	1.00E-06	16.40628806
ENSG00000174576	NPAS4	0.086808898	1.00E-06	16.40555531
ENSG00000175697	GPR156	0.086683598	1.00E-06	16.40347142
ENSG00000277453	CTC-492K19.7	0.086558	1.00E-06	16.40137954
ENSG00000182916	TCEAL7	0.086485	1.00E-06	16.40016231
ENSG00000234125	EEF1GP8	0.086444	1.00E-06	16.39947821
ENSG00000117601	SERPINC1	0.086362704	1.00E-06	16.39812079
ENSG00000254915	RP11-263C24.3	0.086209	1.00E-06	16.39555087
ENSG00000257105	RP11-259O18.4	0.086195	1.00E-06	16.39531656
ENSG00000164344	KLKB1	0.086176746	1.00E-06	16.39501101
ENSG00000273102	AP000569.9	0.086118	1.00E-06	16.39402719
ENSG00000240175	MAGI1-AS1	0.086118	1.00E-06	16.39402719
ENSG00000237453	RP11-67L3.2	0.086063	1.00E-06	16.39310551
ENSG00000253770	HMGB1P23	0.086022	1.00E-06	16.39241805
ENSG00000254593	OR7E126P	0.085973	1.00E-06	16.39159603
ENSG00000241487	RN7SL586P	0.085973	1.00E-06	16.39159603
ENSG00000261469	RP11-96D1.6	0.085941	1.00E-06	16.39105894
ENSG00000197837	HIST4H4	0.085897808	1.00E-06	16.39033369
ENSG00000268649	RP4-806M20.4	0.085684	1.00E-06	16.38673821
ENSG00000200389	RNU6-509P	0.08562	1.00E-06	16.38566022
ENSG00000277812	AC021087.1	0.085578	1.00E-06	16.38495234
ENSG00000271949	RP11-302M6.4	0.085515	1.00E-06	16.38388988
ENSG00000231713	AF064860.7	0.085491692	1.00E-06	16.38349661
ENSG00000260123	RP11-326A19.4	0.085253	1.00E-06	16.37946298
ENSG00000282012	LL22NC03-N95F10.1	0.085253	1.00E-06	16.37946298
ENSG00000251093	RP11-414H23.3	0.085253	1.00E-06	16.37946298
ENSG00000266547	AL161645.1	0.085222	1.00E-06	16.37893829
ENSG00000261462	CTA-254O6.1	0.085217	1.00E-06	16.37885364
ENSG00000268105	RP11-369G6.2	0.085205	1.00E-06	16.37865047
ENSG00000223685	LINC00571	0.085185614	1.00E-06	16.37832219
ENSG00000213137	ARF1P2	0.085142	1.00E-06	16.37758336
ENSG00000182950	ODF3L1	0.085111	1.00E-06	16.37705798
ENSG00000231944	PHKA1-AS1	0.085111	1.00E-06	16.37705798
ENSG00000272361	GS1-166A23.2	0.084968	1.00E-06	16.37463199
ENSG00000279206	RP5-991G20.6	0.084827	1.00E-06	16.37223592
ENSG00000223564	CYP4F32P	0.084827	1.00E-06	16.37223592
ENSG00000251410	AC007106.1	0.084827	1.00E-06	16.37223592
ENSG00000219186	FTH1P19	0.084827	1.00E-06	16.37223592
ENSG00000229259	LRRC37A12P	0.08478	1.00E-06	16.37143635
ENSG00000261302	RP11-343H19.1	0.084756764	1.00E-06	16.37104089
ENSG00000270124	RP11-118F19.1	0.084686	1.00E-06	16.36983587
ENSG00000161643	SIGLEC16	0.084658	1.00E-06	16.36935879

ENSG00000278077	RP11-157L3.10	0.084425	1.00E-06	16.36538265
ENSG00000279613	RP13-638C3.5	0.084405	1.00E-06	16.36504084
ENSG00000238188	RP5-875H3.2	0.084405	1.00E-06	16.36504084
ENSG00000234698	RP11-112L6.2	0.084276258	1.00E-06	16.36283864
ENSG00000214843	ZFYVE9P2	0.084265	1.00E-06	16.3626459
ENSG00000267626	AC002115.9	0.084229	1.00E-06	16.36202942
ENSG00000233445	RPL17P11	0.084204	1.00E-06	16.36160115
ENSG00000227163	RP5-1198O20.5	0.084174	1.00E-06	16.36108706
ENSG00000271482	CTB-152G17.4	0.084174	1.00E-06	16.36108706
ENSG00000273106	RP11-559M23.1	0.084071	1.00E-06	16.35932061
ENSG00000100122	CRYBB1	0.084057	1.00E-06	16.35908035
ENSG00000266943	RPSAP66	0.083987	1.00E-06	16.35787842
ENSG00000270382	RP1-152L7.9	0.083915	1.00E-06	16.3566411
ENSG00000265881	PDLIM1P2	0.083848	1.00E-06	16.35548875
ENSG00000274244	RP11-697E22.3	0.083848	1.00E-06	16.35548875
ENSG00000260152	RP11-69H7.4	0.083711	1.00E-06	16.35312959
ENSG00000225528	RP3-370M22.8	0.083671963	1.00E-06	16.35245666
ENSG00000241590	RPL17P37	0.083589	1.00E-06	16.35102548
ENSG00000257580	RP11-718L23.1	0.083574	1.00E-06	16.35076657
ENSG00000233902	XXbac-BPG181B23.6	0.083574	1.00E-06	16.35076657
ENSG00000234553	AC022431.3	0.083561	1.00E-06	16.35054214
ENSG00000241899	TPT1P3	0.083501	1.00E-06	16.34950585
ENSG00000269514	RP11-370I10.12	0.0835	1.00E-06	16.34948858
ENSG00000262020	RP11-66H6.3	0.083436	1.00E-06	16.34838237
ENSG00000225311	RP1-91J24.3	0.083303	1.00E-06	16.34608083
ENSG00000225328	LINC01594	0.0833	1.00E-06	16.34602888
ENSG00000235836	AC124944.4	0.0833	1.00E-06	16.34602888
ENSG00000228360	RP11-365F18.1	0.083285	1.00E-06	16.34576906
ENSG00000241168	RP11-10O22.1	0.083254	1.00E-06	16.34523197
ENSG00000216083	MIR936	0.083096	1.00E-06	16.34249141
ENSG00000272973	KB-1125A3.11	0.083028	1.00E-06	16.34131033
ENSG00000235785	AL109767.1	0.083022	1.00E-06	16.34120607
ENSG00000204683	C10orf113	0.082893	1.00E-06	16.33896266
ENSG00000226599	RP11-136K14.3	0.082893	1.00E-06	16.33896266
ENSG00000108442	TVP23BP2	0.082758	1.00E-06	16.33661116
ENSG00000272866	RP11-12D24.10	0.082758	1.00E-06	16.33661116
ENSG00000260035	CTD-2651B20.6	0.082624	1.00E-06	16.33427329
ENSG00000213940	RP11-94D20.1	0.082624	1.00E-06	16.33427329
ENSG00000249958	CCT7P2	0.08261	1.00E-06	16.33402881
ENSG00000257443	RP11-150C16.1	0.082534	1.00E-06	16.33270094
ENSG00000157005	SST	0.082356	1.00E-06	16.32958614
ENSG00000265912	RP11-583F2.2	0.08229	1.00E-06	16.3284295
ENSG00000166558	SLC38A8	0.082261623	1.00E-06	16.32793191
ENSG00000102539	MLNR	0.082157	1.00E-06	16.32609588
ENSG00000257585	LINC00609	0.082062	1.00E-06	16.3244267
ENSG00000250061	RP11-541P9.3	0.081997064	1.00E-06	16.32328464
ENSG00000240015	RP11-90P5.5	0.081958	1.00E-06	16.32259716
ENSG00000207277	RNA5SP284	0.081951	1.00E-06	16.32247393

ENSG00000267091	CTBP2P7	0.081827	1.00E-06	16.32028934
ENSG00000184933	OR6A2	0.081821	1.00E-06	16.32018355
ENSG00000259300	TUBBP8	0.081797	1.00E-06	16.31976031
ENSG00000173366	RP11-330H6.5	0.081767902	1.00E-06	16.31924701
ENSG00000162881	OXER1	0.081731	1.00E-06	16.31859577
ENSG00000254400	RP11-732A19.8	0.081639	1.00E-06	16.31697089
ENSG00000250427	HNCAT21	0.081567	1.00E-06	16.31569797
ENSG00000204361	NXPE2	0.08152	1.00E-06	16.31486643
ENSG00000260091	RP11-33B1.4	0.081497	1.00E-06	16.31445933
ENSG00000123570	RAB9B	0.081478	1.00E-06	16.31412295
ENSG00000253175	RP11-267M23.6	0.081434	1.00E-06	16.31334365
ENSG00000137441	FGFBP2	0.081433894	1.00E-06	16.31334178
ENSG00000224397	LINC01272	0.08140049	1.00E-06	16.31274985
ENSG00000270911	RP11-272L13.4	0.081369	1.00E-06	16.31219164
ENSG00000234233	KCNH1-IT1	0.081304	1.00E-06	16.31103871
ENSG00000279536	RP11-445N20.2	0.080916	1.00E-06	16.30413738
ENSG00000236844	AC091633.2	0.08089	1.00E-06	16.30367374
ENSG00000249604	RP11-286E11.2	0.080865	1.00E-06	16.30322779
ENSG00000270962	RP11-403I13.10	0.080815	1.00E-06	16.30233547
ENSG00000275720	CTB-75G16.3	0.080788	1.00E-06	16.30185339
ENSG00000268352	AC007228.5	0.080725	1.00E-06	16.30072792
ENSG00000236616	BAK1P2	0.080659	1.00E-06	16.2995479
ENSG00000234688	RP1-293L6.1	0.080532	1.00E-06	16.29727454
ENSG00000271278	TCEB1P33	0.080532	1.00E-06	16.29727454
ENSG00000176009	ASCL3	0.080405	1.00E-06	16.2949976
ENSG00000278054	RP11-34H11.3	0.080405	1.00E-06	16.2949976
ENSG00000254333	CTC-367J11.1	0.080216	1.00E-06	16.29160241
ENSG00000105205	CLC	0.080151	1.00E-06	16.2904329
ENSG00000230001	RP11-70J12.1	0.080151	1.00E-06	16.2904329
ENSG00000256381	RP11-500M8.4	0.080081	1.00E-06	16.28917237
ENSG00000249488	NACAP5	0.080025	1.00E-06	16.28816315
ENSG00000257723	CHCHD3P2	0.07965	1.00E-06	16.28138674
ENSG00000236849	LINC01474	0.079649859	1.00E-06	16.28138419
ENSG00000230918	AC008063.2	0.079525	1.00E-06	16.27912085
ENSG00000230682	GRPEL2P3	0.079525	1.00E-06	16.27912085
ENSG00000259368	RP11-64K12.4	0.079519	1.00E-06	16.27901199
ENSG00000116176	TPSG1	0.079487302	1.00E-06	16.27843679
ENSG00000267506	RP11-13K12.1	0.079365783	1.00E-06	16.27622954
ENSG00000273030	RP11-285F16.1	0.079265	1.00E-06	16.27439635
ENSG00000113212	PCDHB7	0.078946	1.00E-06	16.26857855
ENSG00000264978	RN7SL630P	0.078829	1.00E-06	16.26643885
ENSG00000255375	RP1-65P5.5	0.078786	1.00E-06	16.26565167
ENSG00000207307	RNU6-145P	0.078745	1.00E-06	16.2649007
ENSG00000259639	RP11-184D12.1	0.078664757	1.00E-06	16.26342982
ENSG00000251413	RP11-534L6.5	0.078433	1.00E-06	16.25917316
ENSG00000280113	AL357140.1	0.078423	1.00E-06	16.25898921
ENSG00000269376	RP11-120K24.5	0.078423	1.00E-06	16.25898921
ENSG00000275467	RP11-240G22.4	0.078302	1.00E-06	16.25676154

ENSG00000234168	LINC01039	0.078302	1.00E-06	16.25676154
ENSG00000278912	CTD-2196E14.8	0.078302	1.00E-06	16.25676154
ENSG00000262133	RP11-676J12.6	0.078302	1.00E-06	16.25676154
ENSG00000217272	RP1-263J7.2	0.078302	1.00E-06	16.25676154
ENSG00000257864	RP11-46I1.2	0.078165	1.00E-06	16.25423514
ENSG00000272431	RP11-281O15.8	0.078158	1.00E-06	16.25410593
ENSG00000224292	AF196972.9	0.077942	1.00E-06	16.25011333
ENSG00000267968	AC011523.2	0.077903	1.00E-06	16.24939127
ENSG00000265264	TIMM10B	0.077830577	1.00E-06	16.24804943
ENSG00000249736	RP11-83M16.5	0.077813	1.00E-06	16.24772358
ENSG00000228364	SAR1P1	0.07771	1.00E-06	16.24581264
ENSG00000251279	CTC-436P18.1	0.077688	1.00E-06	16.24540415
ENSG00000201595	RNA5SP132	0.077638	1.00E-06	16.24447533
ENSG00000257715	RP11-256L6.2	0.077586	1.00E-06	16.24350873
ENSG00000186051	TAL2	0.077586	1.00E-06	16.24350873
ENSG00000224644	RP11-16L21.7	0.077524	1.00E-06	16.24235539
ENSG00000250698	RP11-392B6.1	0.077468	1.00E-06	16.24131287
ENSG00000271142	YWHAQP7	0.07735	1.00E-06	16.23911367
ENSG00000250732	RPEP1	0.07735	1.00E-06	16.23911367
ENSG00000274443	C8orf89	0.07735	1.00E-06	16.23911367
ENSG00000227868	C1orf234	0.077349483	1.00E-06	16.23910403
ENSG00000259245	RP11-684B21.1	0.07731	1.00E-06	16.23836742
ENSG00000253227	RP11-383J24.1	0.07731	1.00E-06	16.23836742
ENSG00000266494	MIR4641	0.077116	1.00E-06	16.2347426
ENSG00000227527	RP11-223A3.1	0.076999	1.00E-06	16.23255209
ENSG00000171747	LGALS4	0.076970062	1.00E-06	16.2320098
ENSG00000264174	RP11-212E8.1	0.076883	1.00E-06	16.23037701
ENSG00000183396	TMEM89	0.076883	1.00E-06	16.23037701
ENSG00000162040	HS3ST6	0.076764	1.00E-06	16.22814227
ENSG00000224407	RP5-956O18.3	0.076704	1.00E-06	16.22701419
ENSG00000250195	RP11-371F15.3	0.076650913	1.00E-06	16.22601535
ENSG00000230970	HHATL-AS1	0.076478067	1.00E-06	16.22275844
ENSG00000240174	RP11-851M3.1	0.076389	1.00E-06	16.22107728
ENSG00000250815	RP11-89B16.2	0.076344	1.00E-06	16.22022716
ENSG00000237819	AC002454.1	0.076306756	1.00E-06	16.21952317
ENSG00000254010	RP11-103H7.5	0.076299	1.00E-06	16.21937653
ENSG00000237927	RP3-393E18.2	0.076268	1.00E-06	16.21879025
ENSG00000235156	TMEM30C	0.076191802	1.00E-06	16.21734816
ENSG00000260861	RP4-576H24.4	0.076165568	1.00E-06	16.21685133
ENSG00000231615	RP11-296O14.2	0.076135	1.00E-06	16.21627221
ENSG00000221933	OR2A25	0.076126	1.00E-06	16.21610165
ENSG00000143125	PROK1	0.076078	1.00E-06	16.2151917
ENSG00000260339	HEXA-AS1	0.076078	1.00E-06	16.2151917
ENSG00000250564	RP11-215P8.4	0.076078	1.00E-06	16.2151917
ENSG00000213880	RPL7AP7	0.076031	1.00E-06	16.21430015
ENSG00000229983	RP11-15I11.2	0.075846	1.00E-06	16.21078548
ENSG00000255838	RP11-753B7.2	0.075739	1.00E-06	16.20874875
ENSG00000253844	RP11-546K22.1	0.075739	1.00E-06	16.20874875

ENSG00000274184	RP11-158H5.8	0.075551	1.00E-06	16.20516323
ENSG00000224370	RP11-814E24.3	0.075514	1.00E-06	16.20445652
ENSG00000231356	RP13-238N7.2	0.075514	1.00E-06	16.20445652
ENSG00000237528	RP11-234P3.4	0.075402	1.00E-06	16.20231517
ENSG00000248796	MED15P8	0.075332	1.00E-06	16.20097521
ENSG00000189030	VHLL	0.07529	1.00E-06	16.20017064
ENSG00000249876	CTC-563A5.4	0.07529	1.00E-06	16.20017064
ENSG00000141750	STAC2	0.075088	1.00E-06	16.19629475
ENSG00000224637	PDSS1P2	0.075068	1.00E-06	16.19591043
ENSG00000272247	RP11-379F4.9	0.074958	1.00E-06	16.19379484
ENSG00000226669	RP11-509J21.4	0.074958	1.00E-06	16.19379484
ENSG00000235917	MTCO2P11	0.074847	1.00E-06	16.19165687
ENSG00000231217	RP11-1M18.1	0.074818	1.00E-06	16.19109778
ENSG00000277539	RP11-298C2.1	0.074814	1.00E-06	16.19102065
ENSG00000249035	CTB-113P19.1	0.074799006	1.00E-06	16.19073149
ENSG00000231920	NEBL-AS1	0.074737649	1.00E-06	16.18954756
ENSG00000167780	SOAT2	0.074701	1.00E-06	16.18883994
ENSG00000227757	AP000282.2	0.074628	1.00E-06	16.1874294
ENSG00000214125	RP3-412A9.15	0.074628	1.00E-06	16.1874294
ENSG00000251648	CTD-2353N24.1	0.074628	1.00E-06	16.1874294
ENSG00000234139	RP4-550H1.4	0.074627657	1.00E-06	16.18742277
ENSG00000259278	RP11-62C7.2	0.074483	1.00E-06	16.18462356
ENSG00000168454	TXNDC2	0.074424328	1.00E-06	16.18348667
ENSG00000255197	RP11-750H9.5	0.074410889	1.00E-06	16.18322614
ENSG00000259071	RP11-247L20.4	0.074366	1.00E-06	16.18235555
ENSG00000248986	RP11-774O3.1	0.074301	1.00E-06	16.18109401
ENSG00000238268	RP11-229P13.19	0.074192	1.00E-06	16.17897601
ENSG00000263171	RP11-542C16.1	0.07415	1.00E-06	16.17815907
ENSG00000233775	RP4-811H24.9	0.074085	1.00E-06	16.17689385
ENSG00000263787	RP11-456D7.1	0.074085	1.00E-06	16.17689385
ENSG00000223824	AC019178.3	0.073908	1.00E-06	16.17344291
ENSG00000266473	RP11-401F2.3	0.07387	1.00E-06	16.17270096
ENSG00000168955	TM4SF20	0.073842591	1.00E-06	16.17216556
ENSG00000256987	RP11-428G5.7	0.073763	1.00E-06	16.17060971
ENSG00000239570	SETP11	0.073763	1.00E-06	16.17060971
ENSG00000271379	RP11-440G2.2	0.073656	1.00E-06	16.16851543
ENSG00000101443	WFDC2	0.073655514	1.00E-06	16.16850591
ENSG00000251191	LINC00589	0.073620642	1.00E-06	16.16782271
ENSG00000197084	LCE1C	0.073550012	1.00E-06	16.16643795
ENSG00000236611	AP000343.2	0.073549	1.00E-06	16.16641811
ENSG00000255362	RP11-619A14.3	0.073458	1.00E-06	16.164632
ENSG00000236173	RP1-182D15.2	0.073337	1.00E-06	16.16225363
ENSG00000267139	AC005262.3	0.07328	1.00E-06	16.16113188
ENSG00000259151	CAP2P1	0.073231	1.00E-06	16.16016688
ENSG00000279275	RP11-756J15.2	0.073127	1.00E-06	16.15811656
ENSG00000275038	RP11-546B8.6	0.072997	1.00E-06	16.15554955
ENSG00000224685	RP11-84O12.3	0.072935	1.00E-06	16.15432368
ENSG00000170323	FABP4	0.072917389	1.00E-06	16.15397528

ENSG00000259899	CTD-3037G24.3	0.072917	1.00E-06	16.15396759
ENSG00000213605	AC111200.2	0.072819	1.00E-06	16.15202731
ENSG00000224577	LINC01117	0.072813	1.00E-06	16.15190843
ENSG00000267568	RP11-87G24.3	0.072726687	1.00E-06	16.15019724
ENSG00000254939	CTD-2342I9.1	0.072543	1.00E-06	16.14654879
ENSG00000248749	RP11-42A4.1	0.072502	1.00E-06	16.14573317
ENSG00000228961	AP000282.3	0.072457	1.00E-06	16.14483745
ENSG00000174944	P2RY14	0.07232173	1.00E-06	16.14214156
ENSG00000261357	RP11-626G11.1	0.072296	1.00E-06	16.14162821
ENSG00000230245	RP11-475I24.7	0.072296	1.00E-06	16.14162821
ENSG00000264422	RP11-434D2.12	0.072091	1.00E-06	16.13753154
ENSG00000264513	RP11-556O9.2	0.072072	1.00E-06	16.13715126
ENSG00000278626	RP11-467L19.16	0.072037	1.00E-06	16.13645048
ENSG00000215457	RPS8P3	0.072008	1.00E-06	16.13586958
ENSG00000280214	CTD-2012K14.5	0.071989	1.00E-06	16.13548886
ENSG00000204814	RP11-160N1.10	0.071980201	1.00E-06	16.1353125
ENSG00000187185	CTD-2600O9.1	0.071927779	1.00E-06	16.13426143
ENSG00000224516	AC068134.8	0.071853365	1.00E-06	16.1327681
ENSG00000264552	AC105030.1	0.071853	1.00E-06	16.13276077
ENSG00000277527	AC007251.2	0.071786	1.00E-06	16.13141489
ENSG00000242134	RPL5P13	0.071786	1.00E-06	16.13141489
ENSG00000103375	AQP8	0.071634889	1.00E-06	16.12837478
ENSG00000265394	RP11-1148O4.2	0.071387	1.00E-06	16.12337375
ENSG00000274354	RP11-1151B14.5	0.071383	1.00E-06	16.12329291
ENSG00000230105	RP11-431N15.2	0.071383	1.00E-06	16.12329291
ENSG00000251139	RP11-701P16.2	0.07132	1.00E-06	16.12201908
ENSG00000279482	RP11-401L13.7	0.071283	1.00E-06	16.12127043
ENSG00000276412	RP11-157L3.9	0.071283	1.00E-06	16.12127043
ENSG00000226556	NPM1P49	0.071283	1.00E-06	16.12127043
ENSG00000228170	RP1-40E16.11	0.071211	1.00E-06	16.11981249
ENSG00000259663	CTD-2314G24.2	0.071163621	1.00E-06	16.1188523
ENSG00000198033	TUBA3C	0.071099	1.00E-06	16.11754165
ENSG00000163497	FEV	0.071040689	1.00E-06	16.11635796
ENSG00000270196	RP11-550A18.1	0.070981	1.00E-06	16.11514528
ENSG00000224885	TSSC1-IT1	0.070886	1.00E-06	16.1132131
ENSG00000229831	AC108039.3	0.070886	1.00E-06	16.1132131
ENSG00000259755	RP11-505E24.2	0.070689	1.00E-06	16.10919811
ENSG00000277671	pRNA	0.070689	1.00E-06	16.10919811
ENSG00000278775	pRNA	0.070689	1.00E-06	16.10919811
ENSG00000276197	pRNA	0.070689	1.00E-06	16.10919811
ENSG00000273937	pRNA	0.070689	1.00E-06	16.10919811
ENSG00000273739	pRNA	0.070689	1.00E-06	16.10919811
ENSG00000276312	pRNA	0.070689	1.00E-06	16.10919811
ENSG00000226339	RPS26P56	0.070689	1.00E-06	16.10919811
ENSG00000244717	RPS27P14	0.070634	1.00E-06	16.10807518
ENSG00000223513	ATP6V0E1P2	0.070632	1.00E-06	16.10803433
ENSG00000102575	ACP5	0.070538173	1.00E-06	16.10611659
ENSG00000224778	CENPIP1	0.070532	1.00E-06	16.10599033

ENSG00000258717	RP11-566J3.2	0.070493	1.00E-06	16.10519238
ENSG00000267521	RP11-87G24.6	0.070493	1.00E-06	16.10519238
ENSG00000273595	RP11-707E21.1	0.070493	1.00E-06	16.10519238
ENSG00000224594	RPL29P19	0.070425	1.00E-06	16.10380004
ENSG00000249960	RP11-791G16.5	0.070299	1.00E-06	16.10121655
ENSG00000136872	ALDOB	0.070187	1.00E-06	16.09891622
ENSG00000220326	RP11-129H15.1	0.070092	1.00E-06	16.09696217
ENSG00000263342	RP1-4G17.2	0.069897	1.00E-06	16.09294292
ENSG00000207293	Y_RNA	0.069857	1.00E-06	16.09211707
ENSG00000280849	AC005339.1	0.069783	1.00E-06	16.090588
ENSG00000275236	AC009120.11	0.069721	1.00E-06	16.08930564
ENSG00000244217	RPS4XP10	0.069625	1.00E-06	16.0873178
ENSG00000260830	RP11-524O1.4	0.069587	1.00E-06	16.08653019
ENSG00000211510	AL590726.1	0.069548	1.00E-06	16.08572141
ENSG00000269966	RP3-331H24.5	0.06953	1.00E-06	16.08534797
ENSG00000204832	ST8SIA6-AS1	0.069472917	1.00E-06	16.08416305
ENSG00000010282	HHATL	0.069363863	1.00E-06	16.08189663
ENSG00000248886	UGT2A3P7	0.069341	1.00E-06	16.08142102
ENSG00000187758	ADH1A	0.069181705	1.00E-06	16.07810294
ENSG00000259317	RP11-561C5.7	0.069153	1.00E-06	16.07750422
ENSG00000241562	RPL7P5	0.069153	1.00E-06	16.07750422
ENSG00000156150	ALX3	0.069111	1.00E-06	16.07662773
ENSG00000250421	RP11-83M16.6	0.069059251	1.00E-06	16.07554707
ENSG00000213529	RP11-432F4.2	0.069041	1.00E-06	16.07516574
ENSG00000254288	RP11-6I2.3	0.068965	1.00E-06	16.07357675
ENSG00000106483	SFRP4	0.06887742	1.00E-06	16.07174349
ENSG00000250016	SNX18P23	0.068851	1.00E-06	16.07118999
ENSG00000237845	RP5-940F7.2	0.068779	1.00E-06	16.06968052
ENSG00000250046	RP11-148B6.2	0.068779	1.00E-06	16.06968052
ENSG00000273293	RP11-445N20.3	0.068779	1.00E-06	16.06968052
ENSG00000255404	RP11-770G2.5	0.068661	1.00E-06	16.06720325
ENSG00000174326	SLC16A11	0.068658876	1.00E-06	16.06715862
ENSG00000277685	MIR4453	0.068621	1.00E-06	16.06636253
ENSG00000270193	RP11-440D17.2	0.068593	1.00E-06	16.06577373
ENSG00000278652	AP001198.1	0.068514	1.00E-06	16.06411119
ENSG00000124835	AC105760.3	0.068439	1.00E-06	16.06253106
ENSG00000258134	RP11-956A19.1	0.068317	1.00E-06	16.059957
ENSG00000279285	RP11-64C12.5	0.068317	1.00E-06	16.059957
ENSG00000248356	RP11-557J10.4	0.068317	1.00E-06	16.059957
ENSG00000227237	AL672294.1	0.068303	1.00E-06	16.05966133
ENSG00000229688	ISPD-AS1	0.06830224	1.00E-06	16.05964528
ENSG00000253772	RP11-34P1.1	0.068249	1.00E-06	16.05852029
ENSG00000128714	HOXD13	0.068164	1.00E-06	16.05672238
ENSG00000259954	IL21R-AS1	0.068122	1.00E-06	16.05583317
ENSG00000258413	RP11-665C16.6	0.068043142	1.00E-06	16.05416213
ENSG00000216802	RP11-390P2.2	0.068043	1.00E-06	16.05415913
ENSG00000279138	KB-1742H10.3	0.068013	1.00E-06	16.05352291
ENSG00000230327	MTCO1P42	0.068001	1.00E-06	16.05326834

ENSG00000279084	AC022405.1	0.067862	1.00E-06	16.05031633
ENSG00000170484	KRT74	0.067793329	1.00E-06	16.04885569
ENSG00000101441	CST4	0.067771	1.00E-06	16.04838044
ENSG00000183318	SPDYE4	0.067739635	1.00E-06	16.04771259
ENSG00000276846	CTD-3220F14.3	0.067591	1.00E-06	16.04454354
ENSG00000258793	RP11-404P21.3	0.067586	1.00E-06	16.04443681
ENSG00000270773	RP13-685P2.7	0.067502	1.00E-06	16.04264263
ENSG00000229401	MIR5689HG	0.067467463	1.00E-06	16.04190429
ENSG00000235991	RP11-143H17.1	0.067412	1.00E-06	16.04071781
ENSG00000120324	PCDHB10	0.067264852	1.00E-06	16.03756523
ENSG00000272682	AC004471.10	0.067234	1.00E-06	16.03690336
ENSG00000228735	GS1-18A18.2	0.067234	1.00E-06	16.03690336
ENSG00000274928	KRT89P	0.067207724	1.00E-06	16.03633943
ENSG00000224603	RP11-96J15.1	0.067145	1.00E-06	16.03499235
ENSG00000234844	CDC42P2	0.067086	1.00E-06	16.03372411
ENSG00000240386	LCE1F	0.067006	1.00E-06	16.03200267
ENSG00000249892	RP11-525J21.1	0.066968	1.00E-06	16.03118426
ENSG00000277253	RP13-580B18.2	0.066937	1.00E-06	16.03051627
ENSG00000247381	PDX1-AS1	0.066936	1.00E-06	16.03049472
ENSG00000253898	LINC01419	0.066924	1.00E-06	16.03023606
ENSG00000277494	GPIHBP1	0.06691	1.00E-06	16.02993422
ENSG00000251576	LINC01267	0.066851	1.00E-06	16.02866152
ENSG00000251009	RP13-494C23.1	0.066793	1.00E-06	16.02740929
ENSG00000234277	CTD-2090I13.1	0.066726984	1.00E-06	16.02598267
ENSG00000237398	HLA-DPA3	0.066676	1.00E-06	16.02487994
ENSG00000156959	LHFPL4	0.066664335	1.00E-06	16.02462751
ENSG00000255538	OR10V2P	0.066618	1.00E-06	16.02362442
ENSG00000199824	RNU6-199P	0.066594	1.00E-06	16.02310458
ENSG00000238280	RP11-436D10.3	0.066579	1.00E-06	16.02277958
ENSG00000245904	RP11-796E2.4	0.066528747	1.00E-06	16.02169025
ENSG00000233129	RP5-837O21.2	0.066444	1.00E-06	16.01985131
ENSG00000251311	RP11-249M12.2	0.06623	1.00E-06	16.01519724
ENSG00000188293	IGFL1	0.066185	1.00E-06	16.01421666
ENSG00000277981	RP11-428P16.3	0.066099	1.00E-06	16.01234083
ENSG00000234776	C11orf94	0.066098286	1.00E-06	16.01232523
ENSG00000234777	RP11-125D12.2	0.065928	1.00E-06	16.0086037
ENSG00000253952	HIGD1AP18	0.065909	1.00E-06	16.00818786
ENSG00000226726	TMEM256P2	0.065672	1.00E-06	16.00299077
ENSG00000254422	RP11-864G5.3	0.065581	1.00E-06	16.00099028
ENSG00000250185	RCC2P8	0.065567	1.00E-06	16.00068227
ENSG00000266830	CTD-2008P7.8	0.065546	1.00E-06	16.00022012
ENSG00000240156	COX6CP6	0.06548	1.00E-06	15.9987667
ENSG00000259182	RP11-424I19.2	0.065457343	1.00E-06	15.99826741
ENSG00000228872	AC096664.2	0.065419	1.00E-06	15.99742209
ENSG00000223791	UBE2E1-AS1	0.065419	1.00E-06	15.99742209
ENSG00000267452	RP11-1018N14.5	0.065356454	1.00E-06	15.9960421
ENSG00000234995	AC013429.5	0.065168	1.00E-06	15.9918761
ENSG00000266832	AL445464.1	0.065099	1.00E-06	15.99034776

ENSG00000262158	MTCO3P24	0.065085	1.00E-06	15.99003747
ENSG00000230571	CTD-2533K21.3	0.065085	1.00E-06	15.99003747
ENSG00000250230	RP11-855O10.2	0.065002	1.00E-06	15.98819649
ENSG00000255970	RP11-43N5.1	0.064939	1.00E-06	15.98679755
ENSG00000259362	RP11-307C19.1	0.064855	1.00E-06	15.98493018
ENSG00000139988	RDH12	0.064808752	1.00E-06	15.98390103
ENSG00000237328	RAI1-AS1	0.064675	1.00E-06	15.98092053
ENSG00000270555	RP11-1394O16.1	0.064589	1.00E-06	15.97900086
ENSG00000275716	RP3-344J20.3	0.064454	1.00E-06	15.97598227
ENSG00000232876	CTA-212D2.2	0.064425	1.00E-06	15.97533301
ENSG00000272323	CTD-2517O10.6	0.064393	1.00E-06	15.97461624
ENSG00000188573	FBLL1	0.064332	1.00E-06	15.97324892
ENSG00000267496	FAM215A	0.064262702	1.00E-06	15.97169402
ENSG00000105374	NKG7	0.064232092	1.00E-06	15.97100666
ENSG00000201861	RNA5SP298	0.064193	1.00E-06	15.97012837
ENSG00000279604	RP11-542M13.1	0.064182	1.00E-06	15.96988113
ENSG00000215520	RPS4XP4	0.064038	1.00E-06	15.96664063
ENSG00000271989	RP4-736L20.3	0.063859	1.00E-06	15.96260234
ENSG00000223523	AC079613.1	0.063859	1.00E-06	15.96260234
ENSG00000237351	CTD-2522E6.4	0.063833	1.00E-06	15.96201483
ENSG00000122711	SPINK4	0.063814734	1.00E-06	15.96160194
ENSG00000257738	RP11-139E19.2	0.063707	1.00E-06	15.95916428
ENSG00000249911	LINC01265	0.06362	1.00E-06	15.95719275
ENSG00000223956	RP4-710M16.2	0.063503	1.00E-06	15.95453713
ENSG00000232358	RP5-955M13.4	0.063461	1.00E-06	15.95358263
ENSG00000249203	RP11-473L15.3	0.06314663	1.00E-06	15.94641813
ENSG00000255037	RP11-680G24.1	0.063128	1.00E-06	15.94599242
ENSG00000261181	RP11-467M13.2	0.063128	1.00E-06	15.94599242
ENSG00000265345	MIR5188	0.063057	1.00E-06	15.94436891
ENSG00000262881	RP11-669E14.4	0.063034	1.00E-06	15.9438426
ENSG00000206034	DEFB109P1B	0.062978	1.00E-06	15.94256032
ENSG00000205989	DEFB109P1B	0.062978	1.00E-06	15.94256032
ENSG00000279609	RP11-235C23.6	0.062796	1.00E-06	15.93838504
ENSG00000270469	RP11-411B10.8	0.062757	1.00E-06	15.93748877
ENSG00000264672	SEPT4-AS1	0.062573312	1.00E-06	15.93325986
ENSG00000267688	AC005262.2	0.062545	1.00E-06	15.93260694
ENSG00000242888	RP11-436M15.1	0.062526	1.00E-06	15.93216861
ENSG00000273736	CTB-186H2.2	0.062526	1.00E-06	15.93216861
ENSG00000227947	RP11-543D5.1	0.06245	1.00E-06	15.93041395
ENSG00000213917	RPL5P8	0.06245	1.00E-06	15.93041395
ENSG00000250426	FTLP10	0.062391	1.00E-06	15.92905031
ENSG00000258437	RP11-661G16.1	0.062321	1.00E-06	15.92743076
ENSG00000255222	SETP17	0.062297	1.00E-06	15.92687507
ENSG00000177096	FAM109B	0.0622962	1.00E-06	15.92685653
ENSG00000260331	RP11-111J6.2	0.062144	1.00E-06	15.92332749
ENSG00000279561	RP11-4L24.4	0.062093	1.00E-06	15.92214302
ENSG00000260190	RP11-229P13.25	0.062069	1.00E-06	15.92158528
ENSG00000282860	RP11-153F10.2	0.062049	1.00E-06	15.92112034

ENSG00000270941	RP5-966M1.5	0.062024	1.00E-06	15.92053895
ENSG00000277770	RP11-72I8.2	0.061927	1.00E-06	15.91828094
ENSG00000233682	RP11-13P5.2	0.061917451	1.00E-06	15.91805847
ENSG00000184258	CDR1	0.061893	1.00E-06	15.91748863
ENSG00000261229	RP11-38G5.4	0.061774	1.00E-06	15.91471213
ENSG00000234592	RP11-41L14.1	0.061768	1.00E-06	15.914572
ENSG00000171724	VAT1L	0.061759373	1.00E-06	15.91437048
ENSG00000230569	AC114763.1	0.061618	1.00E-06	15.91106424
ENSG00000243799	PEX5L-AS1	0.061543	1.00E-06	15.90930715
ENSG00000236390	RP11-25B7.1	0.061532	1.00E-06	15.90904927
ENSG00000188175	HEPACAM2	0.061493529	1.00E-06	15.90814698
ENSG00000229017	LINC01277	0.06146854	1.00E-06	15.9075606
ENSG00000279480	RP13-977J11.3	0.061457	1.00E-06	15.90728972
ENSG00000236687	RP11-323H21.3	0.061395	1.00E-06	15.90583355
ENSG00000236266	RP3-467L1.4	0.061321	1.00E-06	15.9040936
ENSG00000267694	RP11-691H4.4	0.061321	1.00E-06	15.9040936
ENSG00000135577	NMBR	0.061304584	1.00E-06	15.90370733
ENSG00000171483	SSX6	0.061270991	1.00E-06	15.90291656
ENSG00000227032	RP11-34E5.4	0.061246	1.00E-06	15.902328
ENSG00000197935	ZNF311	0.061200488	1.00E-06	15.90125554
ENSG00000219607	PPP1R3G	0.061196	1.00E-06	15.90114974
ENSG00000280003	RP11-330H6.6	0.061167	1.00E-06	15.9004659
ENSG00000254756	RP11-867G23.12	0.061155	1.00E-06	15.90018284
ENSG00000143107	FNDC7	0.0611	1.00E-06	15.89888476
ENSG00000203356	LINC01562	0.061093	1.00E-06	15.89871947
ENSG00000230651	RGPD4-AS1	0.061075426	1.00E-06	15.89830439
ENSG00000224653	RP11-548K12.10	0.061027	1.00E-06	15.89716005
ENSG00000217684	RPS3AP24	0.06102	1.00E-06	15.89699456
ENSG00000143653	SCCPDH	0.061002	1.00E-06	15.89656892
ENSG00000276484	RP11-1212A22.7	0.060999	1.00E-06	15.89649797
ENSG00000232857	KATNBL1P2	0.060953	1.00E-06	15.89540961
ENSG00000205362	MT1A	0.060880112	1.00E-06	15.8936834
ENSG00000197462	AC005276.1	0.060808	1.00E-06	15.89197352
ENSG00000197734	C14orf178	0.06072854	1.00E-06	15.89008706
ENSG00000238048	ART2P	0.060679	1.00E-06	15.88890969
ENSG00000248682	ARHGAP22-IT1	0.060642	1.00E-06	15.88802971
ENSG00000135248	FAM71F1	0.060566587	1.00E-06	15.8862345
ENSG00000234255	AC012370.3	0.060542902	1.00E-06	15.88567022
ENSG00000255114	RP11-110I1.6	0.060514	1.00E-06	15.88498133
ENSG00000237853	NFIA-AS1	0.060446936	1.00E-06	15.88338159
ENSG00000261394	RP11-165M1.3	0.060433	1.00E-06	15.88304894
ENSG00000234184	RP5-887A10.1	0.060300339	1.00E-06	15.87987848
ENSG00000243101	RPS3P7	0.060291	1.00E-06	15.87965504
ENSG00000241359	SYNPR-AS1	0.0602	1.00E-06	15.87747587
ENSG00000267194	RP1-193H18.2	0.060164	1.00E-06	15.87661287
ENSG00000242412	DBIL5P2	0.060161	1.00E-06	15.87654093
ENSG00000214671	RPL6P12	0.060161	1.00E-06	15.87654093
ENSG00000227375	DLG1-AS1	0.06009	1.00E-06	15.8748373

ENSG00000227255	CDRT15P2	0.05995	1.00E-06	15.87147213
ENSG00000267280	TBX2-AS1	0.059927441	1.00E-06	15.87092915
ENSG00000256115	RP11-319E16.1	0.059920537	1.00E-06	15.87076293
ENSG00000224690	UBE2D3P3	0.059878	1.00E-06	15.86973841
ENSG00000274400	RP11-294N21.3	0.059878	1.00E-06	15.86973841
ENSG00000273171	CTB-96E2.2	0.059807	1.00E-06	15.86802673
ENSG00000225640	RP5-1022P6.4	0.05971	1.00E-06	15.86568495
ENSG00000226348	VN2R10P	0.059668	1.00E-06	15.8646698
ENSG00000234132	RP11-168O16.2	0.059597204	1.00E-06	15.86295702
ENSG00000230005	SNAP47-AS1	0.059597	1.00E-06	15.86295209
ENSG00000234597	AC010096.1	0.059563	1.00E-06	15.8621288
ENSG00000229498	AC105053.3	0.059493	1.00E-06	15.86043231
ENSG00000207260	RNU6-35P	0.059458	1.00E-06	15.85958332
ENSG00000206900	RNU6-98P	0.059458	1.00E-06	15.85958332
ENSG00000275158	TRBV12-5	0.059441	1.00E-06	15.85917077
ENSG00000247877	CTD-2001C12.1	0.059423242	1.00E-06	15.85873969
ENSG00000265907	RP11-737O24.2	0.059388742	1.00E-06	15.85790186
ENSG00000230086	VN1R96P	0.05932	1.00E-06	15.85623098
ENSG00000253530	RP11-103H7.3	0.05932	1.00E-06	15.85623098
ENSG00000228734	RP11-335E6.3	0.05925	1.00E-06	15.85452753
ENSG00000242390	RPL6P9	0.05925	1.00E-06	15.85452753
ENSG00000273956	CTB-31N19.5	0.059045	1.00E-06	15.84952728
ENSG00000250343	CTC-255N20.1	0.058975	1.00E-06	15.84781589
ENSG00000257548	RP11-797M17.1	0.058804	1.00E-06	15.84362667
ENSG00000244345	RP11-654C22.2	0.058772	1.00E-06	15.84284137
ENSG00000234378	AC098828.2	0.058674	1.00E-06	15.84043373
ENSG00000162399	BSND	0.058637	1.00E-06	15.83952367
ENSG00000232374	GPR79	0.058637	1.00E-06	15.83952367
ENSG00000283098	RP11-964E11.3	0.058603	1.00E-06	15.8386869
ENSG00000112077	RHAG	0.058562	1.00E-06	15.8376772
ENSG00000215206	TRBV24OR9-2	0.058501	1.00E-06	15.83617367
ENSG00000275950	MIR6724-1	0.058483	1.00E-06	15.8357297
ENSG00000274060	MIR6724-2	0.058483	1.00E-06	15.8357297
ENSG00000277379	MIR6724-3	0.058483	1.00E-06	15.8357297
ENSG00000275692	MIR6724-4	0.058483	1.00E-06	15.8357297
ENSG00000274908	hsa-mir-6724-1	0.058483	1.00E-06	15.8357297
ENSG00000275755	hsa-mir-6724-1	0.058483	1.00E-06	15.8357297
ENSG00000274411	hsa-mir-6724-1	0.058483	1.00E-06	15.8357297
ENSG00000167912	RP11-25K19.1	0.058400988	1.00E-06	15.83370515
ENSG00000227945	RP11-103C16.2	0.05827	1.00E-06	15.83046569
ENSG00000259690	CTD-3118D7.1	0.058254	1.00E-06	15.8300695
ENSG00000101438	SLC32A1	0.058218	1.00E-06	15.82917766
ENSG00000182508	LHFPL1	0.058166529	1.00E-06	15.82790159
ENSG00000220581	VN1R12P	0.058101	1.00E-06	15.82627537
ENSG00000230965	SNX18P13	0.057968	1.00E-06	15.82296909
ENSG00000244573	RPL30P11	0.057905958	1.00E-06	15.82142418
ENSG00000250325	IGBP1P4	0.057903	1.00E-06	15.82135048
ENSG00000250739	LINC01262	0.057903	1.00E-06	15.82135048

ENSG00000255227	RP11-460B17.2	0.057857	1.00E-06	15.8202039
ENSG00000231646	FSIP2-AS1	0.057836852	1.00E-06	15.81970142
ENSG00000258943	RP11-696D21.2	0.057836	1.00E-06	15.81968016
ENSG00000281937	RP3-510D11.4	0.057705	1.00E-06	15.81640871
ENSG00000273997	RP11-361F15.5	0.057705	1.00E-06	15.81640871
ENSG00000125965	GDF5	0.057704754	1.00E-06	15.81640257
ENSG00000262313	CTD-2561B21.4	0.057696	1.00E-06	15.81618368
ENSG00000256813	RP11-804A23.4	0.057521	1.00E-06	15.81180114
ENSG00000277301	RP5-1184F4.7	0.057477	1.00E-06	15.81069714
ENSG00000162882	HAAO	0.05713228	1.00E-06	15.80201847
ENSG00000258503	RP11-368P15.1	0.057123	1.00E-06	15.80178413
ENSG00000204913	LRRC3C	0.057123	1.00E-06	15.80178413
ENSG00000276231	PIK3R6	0.05712246	1.00E-06	15.80177049
ENSG00000206935	RNU6-514P	0.05708	1.00E-06	15.80069771
ENSG00000156219	ART3	0.057066477	1.00E-06	15.80035589
ENSG00000221986	MYBPHL	0.056899329	1.00E-06	15.79612401
ENSG00000267797	NRBF2P1	0.056881	1.00E-06	15.79565921
ENSG00000215267	AKR1C7P	0.056867	1.00E-06	15.79530408
ENSG00000231947	MTND2P24	0.056832	1.00E-06	15.79441587
ENSG00000236039	AC019117.2	0.056803893	1.00E-06	15.79370218
ENSG00000257341	AL928654.7	0.056742557	1.00E-06	15.79214354
ENSG00000227712	RP11-418J17.3	0.056677	1.00E-06	15.79047578
ENSG00000227454	MTND4P30	0.056614	1.00E-06	15.78887124
ENSG00000213854	CNN2P6	0.056552	1.00E-06	15.78729043
ENSG00000234566	RPL7AP71	0.056474	1.00E-06	15.7852992
ENSG00000248553	OR52H2P	0.056426	1.00E-06	15.78407246
ENSG00000204347	BTBD17	0.056395	1.00E-06	15.78327964
ENSG00000237152	DLEU7-AS1	0.056363	1.00E-06	15.78246078
ENSG00000200057	RN7SKP63	0.056363	1.00E-06	15.78246078
ENSG00000235121	RP11-90L20.2	0.05627	1.00E-06	15.78007834
ENSG00000229648	RPSAP22	0.056219	1.00E-06	15.77877017
ENSG00000227762	GUSBP8	0.056218	1.00E-06	15.77874451
ENSG00000205018	RP11-830F9.6	0.056177	1.00E-06	15.77769196
ENSG00000278213	CNTNAP3P5	0.056169	1.00E-06	15.7774865
ENSG00000213411	RBM22P2	0.056115	1.00E-06	15.77609885
ENSG00000270017	CTD-2576F9.2	0.056101	1.00E-06	15.77573887
ENSG00000164621	SMAD5-AS1	0.056053	1.00E-06	15.77450397
ENSG00000250475	RP11-312A15.3	0.056043	1.00E-06	15.77424657
ENSG00000257242	LINC01619	0.055899353	1.00E-06	15.77054397
ENSG00000225230	AC008937.3	0.055686	1.00E-06	15.76502705
ENSG00000255355	AP000640.2	0.055684676	1.00E-06	15.76499275
ENSG00000125780	TGM3	0.055564409	1.00E-06	15.76187347
ENSG00000124479	NDP	0.055533	1.00E-06	15.76105771
ENSG00000274592	MIR10A	0.055523	1.00E-06	15.7607979
ENSG00000181211	HECW1-IT1	0.055352	1.00E-06	15.75634783
ENSG00000147168	IL2RG	0.055322164	1.00E-06	15.75556996
ENSG00000237215	ABC12-47043100G14.2	0.055322	1.00E-06	15.75556569
ENSG00000245832	MIR4300HG	0.055321959	1.00E-06	15.75556463

ENSG00000259384	GH1	0.055321732	1.00E-06	15.75555869
ENSG00000256657	RP11-144O23.22	0.055202	1.00E-06	15.75243292
ENSG00000223548	AC034228.3	0.055142	1.00E-06	15.75086397
ENSG00000228923	AP000355.2	0.055138	1.00E-06	15.75075932
ENSG00000271009	RP11-346C20.3	0.055112	1.00E-06	15.75007886
ENSG00000279658	RP11-389G6.4	0.055098	1.00E-06	15.74971233
ENSG00000223987	RP11-445P17.5	0.05507	1.00E-06	15.74897899
ENSG00000254919	RP11-698N11.2	0.055053426	1.00E-06	15.74854473
ENSG00000277022	RP3-453C12.15	0.055038	1.00E-06	15.74814043
ENSG00000254843	RP11-648O15.1	0.054964	1.00E-06	15.74619938
ENSG00000257194	RP11-567C2.1	0.054964	1.00E-06	15.74619938
ENSG00000183324	REC114	0.054904	1.00E-06	15.74462364
ENSG00000279164	RP4-737E23.5	0.054904	1.00E-06	15.74462364
ENSG00000244215	RP11-88I21.2	0.054904	1.00E-06	15.74462364
ENSG00000260996	RP13-122B23.8	0.054904	1.00E-06	15.74462364
ENSG00000261364	RP11-59E19.4	0.054845	1.00E-06	15.74307248
ENSG00000150175	FRMPD2B	0.054788	1.00E-06	15.74157232
ENSG00000280778	CH17-270A2.2	0.054698781	1.00E-06	15.73922106
ENSG00000228915	OR7E128P	0.054674	1.00E-06	15.73856731
ENSG00000258597	SERPINA2	0.054669	1.00E-06	15.73843537
ENSG00000158315	RHBDL2	0.054629607	1.00E-06	15.73739542
ENSG00000117148	ACTL8	0.054609967	1.00E-06	15.73687666
ENSG00000203730	TEDDM1	0.054561	1.00E-06	15.73558247
ENSG00000226359	ACTG1P24	0.054553	1.00E-06	15.73537092
ENSG00000214797	RP11-1036E20.9	0.054435	1.00E-06	15.73224694
ENSG00000225548	AC098973.2	0.054434308	1.00E-06	15.7322286
ENSG00000007001	UPP2	0.054390176	1.00E-06	15.73105847
ENSG00000218313	RP11-393I2.2	0.054376	1.00E-06	15.73068241
ENSG00000228965	RPSAP60	0.054366	1.00E-06	15.73041706
ENSG00000230099	TRBV5-4	0.054265	1.00E-06	15.72773436
ENSG00000244411	KRTAP5-7	0.054222	1.00E-06	15.72659071
ENSG00000177462	OR2T8	0.054202	1.00E-06	15.72605847
ENSG00000262395	RP11-515E23.2	0.054186	1.00E-06	15.72563253
ENSG00000183791	TCEB3C	0.054096	1.00E-06	15.7232343
ENSG00000234144	COX6CP13	0.054049	1.00E-06	15.72198031
ENSG00000239590	OR1J4	0.05403	1.00E-06	15.72147306
ENSG00000164761	TNFRSF11B	0.054010589	1.00E-06	15.72095465
ENSG00000232498	RP5-1011O1.2	0.053981	1.00E-06	15.72016408
ENSG00000251018	HMMR-AS1	0.053980355	1.00E-06	15.72014685
ENSG00000237621	OR9A1P	0.053858622	1.00E-06	15.7168897
ENSG00000236311	TLX1NB	0.053830607	1.00E-06	15.71613908
ENSG00000236501	AC079305.11	0.053801467	1.00E-06	15.7153579
ENSG00000227019	OR7E101P	0.05374	1.00E-06	15.7137087
ENSG00000280694	Metazoa_SRP	0.053688	1.00E-06	15.71231204
ENSG00000254444	RP11-290F24.3	0.053641	1.00E-06	15.71104851
ENSG00000236651	DLX2-AS1	0.053575	1.00E-06	15.70927232
ENSG00000261368	RP11-96B5.4	0.053518	1.00E-06	15.70773658
ENSG00000281629	Metazoa_SRP	0.053518	1.00E-06	15.70773658

ENSG00000251688	RP11-752L20.5	0.053471	1.00E-06	15.70646904
ENSG00000172733	PURG	0.053447096	1.00E-06	15.70582395
ENSG00000275840	Metazoa_SRP	0.05335	1.00E-06	15.70320065
ENSG00000265767	MIR4745	0.053321	1.00E-06	15.70241622
ENSG00000229311	RP11-475I24.8	0.053195	1.00E-06	15.69900303
ENSG00000215148	PRSS41	0.053183	1.00E-06	15.69867754
ENSG00000269019	AC005932.1	0.053183	1.00E-06	15.69867754
ENSG00000264735	RP11-498C9.17	0.053127	1.00E-06	15.69715763
ENSG00000241722	RP11-255I10.1	0.053017	1.00E-06	15.69416742
ENSG00000123500	COL10A1	0.052997703	1.00E-06	15.69364221
ENSG00000242575	AC012501.3	0.052976	1.00E-06	15.6930513
ENSG00000257955	RP1-228P16.5	0.052907	1.00E-06	15.69117099
ENSG00000204754	CTC-281M20.1	0.052879	1.00E-06	15.69040727
ENSG00000229543	RP11-90J7.2	0.052854	1.00E-06	15.68972504
ENSG00000220960	RP1-72A23.1	0.052797	1.00E-06	15.68816834
ENSG00000266466	RP11-822E23.2	0.052742	1.00E-06	15.68666466
ENSG00000250304	ZBED1P1	0.052742	1.00E-06	15.68666466
ENSG00000261447	RP11-109D9.4	0.052742	1.00E-06	15.68666466
ENSG00000229697	RP11-374M1.3	0.052662591	1.00E-06	15.68449089
ENSG00000268941	RP5-907D15.4	0.052633	1.00E-06	15.68368001
ENSG00000259920	RP11-2E11.5	0.052614	1.00E-06	15.68315912
ENSG00000225650	EIF2S2P5	0.052579	1.00E-06	15.68219908
ENSG00000215838	RP11-466F5.6	0.052524772	1.00E-06	15.68071038
ENSG00000214525	AC130709.1	0.052492	1.00E-06	15.67980995
ENSG00000163661	PTX3	0.05247	1.00E-06	15.67920517
ENSG00000255413	RP11-436P7.2	0.052308	1.00E-06	15.67474399
ENSG00000232398	TMPRSS11CP	0.052164	1.00E-06	15.67076688
ENSG00000215878	MARCKSL1P2	0.052148	1.00E-06	15.6703243
ENSG00000135697	BCO1	0.052112386	1.00E-06	15.66933868
ENSG00000248455	RP11-321E2.3	0.051988146	1.00E-06	15.66589509
ENSG00000236373	RP11-15K3.1	0.051882	1.00E-06	15.66294647
ENSG00000279912	RP11-624A21.1	0.051868	1.00E-06	15.66255712
ENSG00000279462	RP11-604N13.1	0.051829	1.00E-06	15.66147194
ENSG00000181323	SPEM1	0.051829	1.00E-06	15.66147194
ENSG00000257225	RP11-328C8.4	0.051803	1.00E-06	15.66074803
ENSG00000259527	LINC00052	0.051777	1.00E-06	15.66002376
ENSG00000254488	RP11-65G9.1	0.051777	1.00E-06	15.66002376
ENSG00000213484	EIF4A1P8	0.051751	1.00E-06	15.65929912
ENSG00000229694	RP11-305L7.6	0.051724314	1.00E-06	15.65855499
ENSG00000227015	RP11-548K12.6	0.051724	1.00E-06	15.65854623
ENSG00000253507	CTD-2501M5.1	0.051723882	1.00E-06	15.65854294
ENSG00000267263	RP11-75C10.7	0.051691	1.00E-06	15.65762549
ENSG00000271010	CTC-539A10.7	0.051687	1.00E-06	15.65751385
ENSG00000231680	AP003774.6	0.051671	1.00E-06	15.65706718
ENSG00000227193	RP11-439A17.4	0.051514	1.00E-06	15.65267695
ENSG00000230186	RP5-998N21.7	0.051514	1.00E-06	15.65267695
ENSG00000234892	Z82214.2	0.051514	1.00E-06	15.65267695
ENSG00000275239	RP11-157L3.12	0.051513821	1.00E-06	15.65267193

ENSG00000136634	IL10	0.051462841	1.00E-06	15.65124349
ENSG00000229618	AC011288.2	0.051462791	1.00E-06	15.65124207
ENSG00000131142	CCL25	0.051462	1.00E-06	15.65121991
ENSG00000224604	AC008069.2	0.051432	1.00E-06	15.65037864
ENSG00000253374	RP11-257P3.3	0.051362276	1.00E-06	15.64842151
ENSG00000200227	RNA5SP197	0.051324	1.00E-06	15.64734599
ENSG00000162068	NTN3	0.051255	1.00E-06	15.64540513
ENSG00000232480	TGFB2-AS1	0.05117	1.00E-06	15.64301061
ENSG00000272770	RP11-74E22.5	0.051106	1.00E-06	15.64120506
ENSG00000234703	AF015262.2	0.051042	1.00E-06	15.63939724
ENSG00000231422	LINC01516	0.051033	1.00E-06	15.63914283
ENSG00000261420	RP1-168L15.5	0.05102	1.00E-06	15.63877528
ENSG00000236674	RP4-792G4.3	0.051009	1.00E-06	15.6384642
ENSG00000257114	RP11-25I15.3	0.050744	1.00E-06	15.63094963
ENSG00000214711	CAPN14	0.050718817	1.00E-06	15.63023349
ENSG00000254417	ANO1-AS2	0.050694	1.00E-06	15.62952738
ENSG00000278647	CDK2AP2P1	0.05069	1.00E-06	15.62941354
ENSG00000227869	RP11-807H17.1	0.050673713	1.00E-06	15.62894992
ENSG00000229536	AC079776.1	0.050625	1.00E-06	15.62756238
ENSG00000257407	RP11-1028N23.4	0.050571822	1.00E-06	15.62604613
ENSG00000179412	HNRNPCL4	0.05055	1.00E-06	15.62542347
ENSG00000184139	RPL7AP28	0.05051	1.00E-06	15.62428142
ENSG00000280016	RP11-191F9.1	0.050392	1.00E-06	15.6209071
ENSG00000224984	RP11-524H19.2	0.050375	1.00E-06	15.62042031
ENSG00000259094	RP11-77A13.1	0.050342	1.00E-06	15.61947491
ENSG00000096088	PGC	0.050267292	1.00E-06	15.61733235
ENSG00000258872	FDPSP3	0.050243	1.00E-06	15.61663499
ENSG00000224863	LINC01398	0.050241056	1.00E-06	15.61657916
ENSG00000225929	AC000036.4	0.050144	1.00E-06	15.61378946
ENSG00000242525	OR7E100P	0.050144	1.00E-06	15.61378946
ENSG00000231198	AC004987.10	0.050095	1.00E-06	15.61237899
ENSG00000252909	RNU6-201P	0.050062	1.00E-06	15.61142831
ENSG00000259776	RP11-544D21.2	0.050034	1.00E-06	15.61062117
ENSG00000179058	C9orf50	0.049962	1.00E-06	15.60854361
ENSG00000239873	GAPDHP27	0.049899	1.00E-06	15.60672328
ENSG00000281264	SALRNA3	0.04985	1.00E-06	15.60530588
ENSG00000261210	CLEC19A	0.049800814	1.00E-06	15.60388171
ENSG00000262412	RP11-85G18.6	0.049779	1.00E-06	15.60324963
ENSG00000189051	RNF222	0.049735999	1.00E-06	15.60200283
ENSG00000278873	PRO1804	0.049654	1.00E-06	15.59962232
ENSG00000251253	MTHFD2P4	0.049654	1.00E-06	15.59962232
ENSG00000253775	AC100802.3	0.049606	1.00E-06	15.59822701
ENSG00000259922	RP11-322D14.1	0.049414	1.00E-06	15.59263222
ENSG00000238025	ZDHHC4P1	0.049175	1.00E-06	15.58563743
ENSG00000177243	DEFB103B	0.049128	1.00E-06	15.58425789
ENSG00000176797	DEFB103A	0.049128	1.00E-06	15.58425789
ENSG00000260433	RP11-202D1.2	0.04908	1.00E-06	15.58284763
ENSG00000259467	NDUFAF4P1	0.049033	1.00E-06	15.58146541

ENSG00000258452	RP11-757H14.2	0.048939	1.00E-06	15.578697
ENSG00000276674	IGKV1OR1-1	0.048892	1.00E-06	15.5773108
ENSG00000228668	TRGV5P	0.048884	1.00E-06	15.57707472
ENSG00000251286	RP11-588F10.1	0.048845	1.00E-06	15.57592327
ENSG00000224297	RP11-197P3.1	0.048845	1.00E-06	15.57592327
ENSG00000240057	RP11-572M11.4	0.048811472	1.00E-06	15.57493263
ENSG00000175121	WFDC5	0.048751515	1.00E-06	15.57315944
ENSG00000242983	CABYRP1	0.048705	1.00E-06	15.57178226
ENSG00000269161	CTD-3131K8.3	0.048683	1.00E-06	15.57113045
ENSG00000229962	AP000221.1	0.048658	1.00E-06	15.5703894
ENSG00000234292	RP11-213H15.1	0.048658	1.00E-06	15.5703894
ENSG00000272144	CTD-2035E11.5	0.048611	1.00E-06	15.56899519
ENSG00000276548	CTD-2547E10.5	0.048582	1.00E-06	15.56813426
ENSG00000118473	SGIP1	0.048579472	1.00E-06	15.56805919
ENSG00000278200	RP13-766D20.4	0.048565	1.00E-06	15.56762934
ENSG00000254384	MTND6P19	0.048565	1.00E-06	15.56762934
ENSG00000222335	Y_RNA	0.048473	1.00E-06	15.56489375
ENSG00000147262	GPR119	0.048473	1.00E-06	15.56489375
ENSG00000224479	AC136289.1	0.048456794	1.00E-06	15.56441134
ENSG00000170178	HOXD12	0.04839978	1.00E-06	15.56271287
ENSG00000267032	RP11-807E13.2	0.048334	1.00E-06	15.56075077
ENSG00000227173	MYL6P3	0.048305	1.00E-06	15.55988491
ENSG00000238276	RP11-245J24.1	0.048288653	1.00E-06	15.55939659
ENSG00000234224	TMEM229A	0.048288	1.00E-06	15.55937709
ENSG00000281938	CTB-127M13.1	0.048272	1.00E-06	15.55889898
ENSG00000280181	RP11-629N8.4	0.048197	1.00E-06	15.55665573
ENSG00000272908	RP11-121A8.1	0.048103	1.00E-06	15.55383925
ENSG00000256533	RP11-212D19.5	0.048015	1.00E-06	15.55119756
ENSG00000269421	ZNF92P3	0.048015	1.00E-06	15.55119756
ENSG00000279646	AC016577.1	0.047994	1.00E-06	15.55056644
ENSG00000265817	FSBP	0.0479857	1.00E-06	15.55031691
ENSG00000148204	CRB2	0.047938573	1.00E-06	15.54889934
ENSG00000235235	IGKV1OR2-1	0.047925	1.00E-06	15.54849081
ENSG00000244682	FCGR2C	0.047768222	1.00E-06	15.54376354
ENSG00000112116	IL17F	0.047700716	1.00E-06	15.5417233
ENSG00000230140	AC016738.3	0.047698591	1.00E-06	15.54165903
ENSG00000188124	OR2AG2	0.047611	1.00E-06	15.53900731
ENSG00000178358	OR2D3	0.047567	1.00E-06	15.53767342
ENSG00000206992	RNU6-574P	0.047567	1.00E-06	15.53767342
ENSG00000230257	NFE4	0.047478	1.00E-06	15.53497154
ENSG00000213729	AC098828.3	0.047426	1.00E-06	15.53339057
ENSG00000234174	AC016683.5	0.047389043	1.00E-06	15.5322659
ENSG00000196408	NOXO1	0.047371957	1.00E-06	15.53174566
ENSG00000155886	SLC24A2	0.04735	1.00E-06	15.53107681
ENSG00000253130	CTD-3023L14.2	0.047345137	1.00E-06	15.53092862
ENSG00000258731	RP11-547D23.1	0.047301	1.00E-06	15.52958306
ENSG00000237419	RP11-885N19.6	0.047301	1.00E-06	15.52958306
ENSG00000241505	RP11-161I10.1	0.047275	1.00E-06	15.52878984

ENSG00000167618	LAIR2	0.047213199	1.00E-06	15.52690263
ENSG00000253433	NCRNA00250	0.047072	1.00E-06	15.52258153
ENSG00000279825	CTC-457L16.1	0.047031	1.00E-06	15.52132439
ENSG00000140488	CELF6	0.047000606	1.00E-06	15.52039174
ENSG00000229989	MIR181A1HG	0.046980938	1.00E-06	15.51978789
ENSG00000213886	UBD	0.046822	1.00E-06	15.51489894
ENSG00000170370	EMX2	0.046808486	1.00E-06	15.51448249
ENSG00000203737	GPR52	0.04678	1.00E-06	15.51360424
ENSG00000213264	NIP7P2	0.04678	1.00E-06	15.51360424
ENSG00000261024	GS1-279B7.1	0.046779932	1.00E-06	15.51360214
ENSG00000203386	LINC01317	0.046736423	1.00E-06	15.51225971
ENSG00000004846	ABCB5	0.046734172	1.00E-06	15.51219023
ENSG00000218357	LL22NC03-75H12.2	0.04669396	1.00E-06	15.51094831
ENSG00000240128	KRT18P43	0.046619	1.00E-06	15.50863044
ENSG00000277508	BSNDP3	0.046609	1.00E-06	15.50832094
ENSG00000234256	PTCD2P2	0.04648	1.00E-06	15.50432245
ENSG00000278905	RP11-65F13.3	0.046469	1.00E-06	15.50398098
ENSG00000183876	ARSI	0.046396789	1.00E-06	15.50173733
ENSG00000274973	BSNDP2	0.046353	1.00E-06	15.50037509
ENSG00000270554	RP11-225N10.3	0.046311	1.00E-06	15.49906729
ENSG00000275067	MIR6825	0.04627	1.00E-06	15.49778948
ENSG00000275048	BSNDP1	0.046227	1.00E-06	15.49644812
ENSG00000253197	CTD-3239E11.2	0.046220505	1.00E-06	15.49624539
ENSG00000258660	RP4-693M11.3	0.046213	1.00E-06	15.49601113
ENSG00000253161	LINC01605	0.04614821	1.00E-06	15.49398708
ENSG00000203795	FAM24A	0.046114	1.00E-06	15.49291719
ENSG00000255552	LY6G6E	0.04610153	1.00E-06	15.49252701
ENSG00000162598	C1orf87	0.046039099	1.00E-06	15.49057198
ENSG00000260369	CTD-2526A2.2	0.046037	1.00E-06	15.4905062
ENSG00000251193	RP11-72K17.1	0.045906	1.00E-06	15.48639511
ENSG00000189068	VSTM1	0.045811248	1.00E-06	15.48341423
ENSG00000279026	RP3-414A15.12	0.04575	1.00E-06	15.48148412
ENSG00000175485	OR52W1	0.045687	1.00E-06	15.47949609
ENSG00000124003	MOGAT1	0.045651	1.00E-06	15.47835884
ENSG00000222102	RN7SKP232	0.045632	1.00E-06	15.47775827
ENSG00000048462	TNFRSF17	0.045524844	1.00E-06	15.47436646
ENSG00000235522	AC009505.2	0.045515535	1.00E-06	15.47407141
ENSG00000235584	AC008268.1	0.045484	1.00E-06	15.47307151
ENSG00000054356	PTPRN	0.045469032	1.00E-06	15.47259668
ENSG00000257381	MIR3179-2	0.045443	1.00E-06	15.47177046
ENSG00000266454	MIR3179-3	0.045443	1.00E-06	15.47177046
ENSG00000277014	MIR3179-4	0.045443	1.00E-06	15.47177046
ENSG00000268015	CTD-2525I3.3	0.045402587	1.00E-06	15.47048688
ENSG00000278909	RP11-401P9.7	0.045376	1.00E-06	15.46964182
ENSG00000237451	CDK2AP2P2	0.045303	1.00E-06	15.46731897
ENSG00000176840	MIR7-3HG	0.045281794	1.00E-06	15.46664351
ENSG00000196166	C8orf86	0.045241	1.00E-06	15.4653432
ENSG00000260161	MTCYBP28	0.044962	1.00E-06	15.45641859

ENSG00000257281	RP11-1K3.1	0.044922	1.00E-06	15.45513454
ENSG00000270754	NEK4P3	0.044909	1.00E-06	15.45471698
ENSG00000261187	RP11-1007O24.2	0.044843	1.00E-06	15.45259518
ENSG00000152611	CAPSL	0.044842974	1.00E-06	15.45259436
ENSG00000256310	NDUFA5P6	0.044763	1.00E-06	15.45001911
ENSG00000226020	CDK2AP2P3	0.044694	1.00E-06	15.44779355
ENSG00000196734	LCE1B	0.044685	1.00E-06	15.447503
ENSG00000119660	DPPA5P4	0.044622	1.00E-06	15.44546756
ENSG00000224448	GS1-259H13.7	0.04451	1.00E-06	15.44184188
ENSG00000260392	RP11-1129I3.1	0.044491596	1.00E-06	15.44124523
ENSG00000279170	RP1-199J3.6	0.044489	1.00E-06	15.44116105
ENSG00000251497	RP11-197N18.7	0.04446993	1.00E-06	15.4405425
ENSG00000223561	AC003090.1	0.044452823	1.00E-06	15.43998744
ENSG00000223568	RPL3P11	0.044412	1.00E-06	15.43866192
ENSG00000271466	CICP24	0.044408	1.00E-06	15.43853198
ENSG00000240661	RP11-174O3.3	0.04439291	1.00E-06	15.43804167
ENSG00000250897	HMGB3P15	0.044335	1.00E-06	15.43615846
ENSG00000225508	SSXP5	0.044335	1.00E-06	15.43615846
ENSG00000226867	RP13-77O11.8	0.044335	1.00E-06	15.43615846
ENSG00000277734	TRAC	0.044334348	1.00E-06	15.43613722
ENSG00000205445	KRTAP10-2	0.044296	1.00E-06	15.43488881
ENSG00000232756	RP5-1185I7.1	0.044295924	1.00E-06	15.43488634
ENSG00000265096	C1QTNF1-AS1	0.044257376	1.00E-06	15.43363029
ENSG00000123171	CCDC70	0.044257	1.00E-06	15.43361804
ENSG00000272727	RP11-142A22.4	0.044219	1.00E-06	15.43237878
ENSG00000226298	RAD23BLP	0.044181	1.00E-06	15.43113845
ENSG00000229794	MTCYBP32	0.044135	1.00E-06	15.42963558
ENSG00000223592	FNDC3CP	0.044066	1.00E-06	15.42737832
ENSG00000257062	RP11-125O5.2	0.044050661	1.00E-06	15.42687604
ENSG00000126266	FFAR1	0.044047	1.00E-06	15.42675614
ENSG00000280368	CTD-2199O4.5	0.044018	1.00E-06	15.42580598
ENSG00000128310	GALR3	0.04399	1.00E-06	15.42488798
ENSG00000248547	RP11-468N14.6	0.043939	1.00E-06	15.42321442
ENSG00000151948	GLT1D1	0.04390117	1.00E-06	15.42197178
ENSG00000186442	KRT3	0.043895	1.00E-06	15.42176899
ENSG00000226804	RP4-675G8.3	0.043876	1.00E-06	15.42114439
ENSG00000225922	RP11-57G10.1	0.043839	1.00E-06	15.41992727
ENSG00000283000	RP11-297A16.4	0.043838395	1.00E-06	15.41990737
ENSG00000250400	LINC00977	0.043763	1.00E-06	15.41742402
ENSG00000154620	TMSB4Y	0.043761	1.00E-06	15.41735809
ENSG00000265265	RP11-822E23.7	0.043698	1.00E-06	15.41527963
ENSG00000254934	LINC00678	0.043687931	1.00E-06	15.41494717
ENSG00000178734	LMO7DN	0.043613	1.00E-06	15.41247061
ENSG00000232625	RPL23AP31	0.043595	1.00E-06	15.41187506
ENSG00000255568	BRWD1-AS2	0.043569	1.00E-06	15.41101438
ENSG00000250261	CCDC74BP1	0.043509	1.00E-06	15.40902624
ENSG00000108381	ASPA	0.043471194	1.00E-06	15.40777209
ENSG00000180532	ZSCAN4	0.043450962	1.00E-06	15.4071005

ENSG00000214787	MS4A4E	0.043448251	1.00E-06	15.40701048
ENSG00000266105	RP11-326K13.2	0.043427	1.00E-06	15.40630467
ENSG00000258586	RP5-1021I20.2	0.043352	1.00E-06	15.40381093
ENSG00000228417	RP11-549L6.3	0.043316	1.00E-06	15.4026124
ENSG00000237923	XXbac-BPG27H4.8	0.043316	1.00E-06	15.4026124
ENSG00000261241	RP11-883G14.1	0.043096	1.00E-06	15.39526635
ENSG00000258957	RP11-363J20.1	0.043059458	1.00E-06	15.39404254
ENSG00000241233	KRTAP5-8	0.043023	1.00E-06	15.39282051
ENSG00000261386	CTD-2012K14.6	0.042986406	1.00E-06	15.39159288
ENSG00000172123	SLFN12	0.042889844	1.00E-06	15.38834843
ENSG00000270434	RP11-175I17.6	0.042842	1.00E-06	15.38673821
ENSG00000251538	RP11-166A12.1	0.042824343	1.00E-06	15.3861435
ENSG00000229893	AC004549.6	0.04277	1.00E-06	15.38431159
ENSG00000231929	HMGB3P31	0.04277	1.00E-06	15.38431159
ENSG00000233782	ZNF90P3	0.042698	1.00E-06	15.38188087
ENSG00000250436	RP11-622A1.2	0.042662448	1.00E-06	15.38067912
ENSG00000279785	GS1-124K5.13	0.042627	1.00E-06	15.3794799
ENSG00000283095	ABC11-4932300O16.1	0.042592	1.00E-06	15.37829486
ENSG00000232827	LINC01189	0.042562	1.00E-06	15.37727832
ENSG00000233651	RP11-111F5.8	0.042512	1.00E-06	15.37558251
ENSG00000256705	CTD-2017C7.1	0.042484	1.00E-06	15.37463199
ENSG00000227487	NCAM1-AS1	0.042483841	1.00E-06	15.3746266
ENSG00000228723	SRGAP3-AS2	0.04244302	1.00E-06	15.3732397
ENSG00000255345	CTD-2337I7.1	0.042431438	1.00E-06	15.37284596
ENSG00000231955	AC007383.4	0.042319	1.00E-06	15.36901792
ENSG00000176349	AC110781.3	0.042307074	1.00E-06	15.36861128
ENSG00000227538	HNRNPFP1	0.042227	1.00E-06	15.36587813
ENSG00000064655	EYA2	0.042202133	1.00E-06	15.36502831
ENSG00000225329	LHFPL3-AS2	0.042188678	1.00E-06	15.36456827
ENSG00000268423	AC011551.3	0.042162284	1.00E-06	15.3636654
ENSG00000223601	EBLN1	0.042133	1.00E-06	15.36266302
ENSG00000238210	RP13-210D15.4	0.042132583	1.00E-06	15.36264874
ENSG00000242611	AC093627.8	0.042063	1.00E-06	15.36026413
ENSG00000230090	AC108025.2	0.041994535	1.00E-06	15.35791396
ENSG00000275454	CTD-2026K11.4	0.041821	1.00E-06	15.35193994
ENSG00000111537	IFNG	0.041787	1.00E-06	15.35076657
ENSG00000271998	CTD-2199O4.7	0.041787	1.00E-06	15.35076657
ENSG00000221938	OR2A14	0.041757454	1.00E-06	15.34974614
ENSG00000168903	BTNL3	0.041719	1.00E-06	15.34841696
ENSG00000255299	RP11-655C2.3	0.041651623	1.00E-06	15.34608508
ENSG00000224346	BICD1P1	0.04165	1.00E-06	15.34602888
ENSG00000279106	RP11-426C22.1	0.041616	1.00E-06	15.34485068
ENSG00000258316	KLF17P1	0.041585	1.00E-06	15.34377561
ENSG00000279282	AL354828.1	0.041582	1.00E-06	15.34367153
ENSG00000262619	LINC00621	0.041513	1.00E-06	15.34127557
ENSG00000248640	HNRNPA1P56	0.041461	1.00E-06	15.33946729
ENSG00000242070	NPM1P17	0.041427	1.00E-06	15.33828373
ENSG00000258791	LINC00520	0.041345415	1.00E-06	15.33543973

ENSG00000253653	CTC-248O19.1	0.041343	1.00E-06	15.33535546
ENSG00000231546	RPL3P5	0.041311	1.00E-06	15.33423836
ENSG00000225399	RP11-3B7.1	0.041311	1.00E-06	15.33423836
ENSG00000213487	ASS1P4	0.041245	1.00E-06	15.33193162
ENSG00000266844	RP11-862L9.3	0.041078563	1.00E-06	15.32609811
ENSG00000277769	RBM22P4	0.041046	1.00E-06	15.32495402
ENSG00000163958	ZDHHC19	0.04101838	1.00E-06	15.32398288
ENSG00000180479	ZNF571	0.041005648	1.00E-06	15.32353502
ENSG00000204961	PCDHA9	0.040921052	1.00E-06	15.32055562
ENSG00000206659	Y_RNA	0.040916	1.00E-06	15.32037749
ENSG00000200849	Y_RNA	0.040916	1.00E-06	15.32037749
ENSG00000207142	Y_RNA	0.040916	1.00E-06	15.32037749
ENSG00000200419	Y_RNA	0.040916	1.00E-06	15.32037749
ENSG00000200494	Y_RNA	0.040916	1.00E-06	15.32037749
ENSG00000201955	RNY3P1	0.040916	1.00E-06	15.32037749
ENSG00000207499	Y_RNA	0.040916	1.00E-06	15.32037749
ENSG00000249283	ACTR3BP4	0.040913	1.00E-06	15.32027171
ENSG00000248458	RP4-598P13.1	0.040857	1.00E-06	15.31829565
ENSG00000237088	RP11-73E6.2	0.040852	1.00E-06	15.31811909
ENSG00000234913	XXbac-B476C20.13	0.040831	1.00E-06	15.31737728
ENSG00000255474	RP11-234B24.2	0.04075	1.00E-06	15.31451244
ENSG00000235978	AC018816.3	0.040684602	1.00E-06	15.31219525
ENSG00000229754	CXCR2P1	0.040668256	1.00E-06	15.31161552
ENSG00000126353	CCR7	0.040586927	1.00E-06	15.30872748
ENSG00000236423	LINC01134	0.040576791	1.00E-06	15.30836715
ENSG00000236138	DUX4L26	0.040555	1.00E-06	15.30759217
ENSG00000254017	IGHEP2	0.040523	1.00E-06	15.30645336
ENSG00000267646	CTC-499B15.7	0.040474	1.00E-06	15.30470782
ENSG00000254750	CASP1P2	0.040458	1.00E-06	15.30413738
ENSG00000277041	RP11-368J21.4	0.040432	1.00E-06	15.30320995
ENSG00000277003	RP11-419C5.3	0.040432	1.00E-06	15.30320995
ENSG00000242971	RN7SL233P	0.040423	1.00E-06	15.30288878
ENSG00000211683	KB-1572G7.3	0.040267	1.00E-06	15.29731037
ENSG00000273423	OR13I1P	0.040266	1.00E-06	15.29727454
ENSG00000227681	RP11-307P5.1	0.040170525	1.00E-06	15.29384969
ENSG00000253348	CTC-455F18.1	0.040170361	1.00E-06	15.29384381
ENSG00000278899	AL358852.1	0.040139	1.00E-06	15.29271705
ENSG00000267549	AC006116.17	0.040122822	1.00E-06	15.29213547
ENSG00000204983	PRSS1	0.040110916	1.00E-06	15.29170731
ENSG00000241135	LINC00881	0.040107253	1.00E-06	15.29157554
ENSG00000232719	RP11-13J8.1	0.040107	1.00E-06	15.29156644
ENSG00000155890	TRIM42	0.040092	1.00E-06	15.29102677
ENSG00000276822	RP11-252A24.8	0.040057	1.00E-06	15.28976676
ENSG00000173372	C1QA	0.040012679	1.00E-06	15.28816961
ENSG00000250938	RP11-679C8.2	0.039988512	1.00E-06	15.28729798
ENSG00000214745	RP11-54I5.1	0.039919	1.00E-06	15.28478796
ENSG00000204472	AIF1	0.039886959	1.00E-06	15.28362952
ENSG00000249240	AC069368.3	0.039856435	1.00E-06	15.28252507

ENSG00000274220	RP11-77K12.9	0.039809	1.00E-06	15.28080701
ENSG00000203688	RP11-351J23.1	0.039809	1.00E-06	15.28080701
ENSG00000121764	HCRTR1	0.039793959	1.00E-06	15.2802618
ENSG00000270112	RP11-742D12.2	0.039785419	1.00E-06	15.27995218
ENSG00000224417	RP11-503C24.6	0.039778	1.00E-06	15.27968312
ENSG00000233124	LINC00456	0.039716	1.00E-06	15.27743271
ENSG00000255314	RP11-702F3.3	0.039638526	1.00E-06	15.27461568
ENSG00000279317	AC006994.3	0.039608	1.00E-06	15.27350423
ENSG00000235358	RP11-399E6.1	0.039524429	1.00E-06	15.27045701
ENSG00000234865	CTD-2138O14.1	0.039522	1.00E-06	15.27036834
ENSG00000250391	CTD-2160D9.1	0.039485	1.00E-06	15.26901707
ENSG00000267415	CTC-503J8.2	0.039465	1.00E-06	15.26828613
ENSG00000262332	RP11-517A5.7	0.039439	1.00E-06	15.26733535
ENSG00000057593	F7	0.039429394	1.00E-06	15.26698391
ENSG00000259017	RP11-561B11.6	0.039383	1.00E-06	15.26528539
ENSG00000196289	BECN2	0.039272	1.00E-06	15.26121345
ENSG00000279334	RP11-83B20.6	0.039245	1.00E-06	15.26022124
ENSG00000213109	RP11-55K22.2	0.039241758	1.00E-06	15.26010205
ENSG00000229228	LINC00582	0.039217	1.00E-06	15.25919156
ENSG00000266806	RP11-744K17.3	0.039117	1.00E-06	15.25550811
ENSG00000226913	BSN-AS2	0.039106	1.00E-06	15.25510236
ENSG00000224039	AC091493.1	0.039097	1.00E-06	15.25477029
ENSG00000279852	RP11-91J19.2	0.039085	1.00E-06	15.25432742
ENSG00000266101	RP5-906A24.2	0.039082	1.00E-06	15.25421668
ENSG00000203635	AC144450.2	0.039079	1.00E-06	15.25410593
ENSG00000267934	CTB-176F20.3	0.03906	1.00E-06	15.25340433
ENSG00000182492	BGN	0.039006796	1.00E-06	15.25143787
ENSG00000172640	OR10AD1	0.039001	1.00E-06	15.2512235
ENSG00000073734	ABCB11	0.03893048	1.00E-06	15.24861249
ENSG00000239508	PLCH1-AS1	0.038911	1.00E-06	15.24789044
ENSG00000197919	IFNA1	0.038845479	1.00E-06	15.24545907
ENSG00000277058	HNRNPCL3	0.038837	1.00E-06	15.24514414
ENSG00000205976	RP11-404K5.2	0.038818	1.00E-06	15.24443817
ENSG00000254560	BBOX1-AS1	0.038792881	1.00E-06	15.24350431
ENSG00000259175	RP11-379K22.2	0.038778	1.00E-06	15.24295078
ENSG00000273287	CTA-268H5.14	0.038764	1.00E-06	15.24242983
ENSG00000260509	RP11-271M24.2	0.038705	1.00E-06	15.24023233
ENSG00000229988	HBBP1	0.038697562	1.00E-06	15.23995506
ENSG00000240922	LSAMP-AS1	0.038656	1.00E-06	15.23840474
ENSG00000188399	ANKRD36P1	0.038616	1.00E-06	15.23691111
ENSG00000256070	RP11-56H16.1	0.038585	1.00E-06	15.23575249
ENSG00000106571	GLI3	0.038561342	1.00E-06	15.23486765
ENSG00000261466	RP11-23E10.2	0.038558	1.00E-06	15.2347426
ENSG00000163825	RTP3	0.038528	1.00E-06	15.23361968
ENSG00000271339	RP11-1114A5.6	0.038527	1.00E-06	15.23358223
ENSG00000213232	PPP1R2P10	0.038493	1.00E-06	15.23230849
ENSG00000217576	RP11-248G5.8	0.03834	1.00E-06	15.22656272
ENSG00000269985	RP1-232P20.1	0.038181833	1.00E-06	15.22059876

ENSG00000263327	TAPT1-AS1	0.038174846	1.00E-06	15.22033472
ENSG00000129009	ISLR	0.038109986	1.00E-06	15.21788145
ENSG00000237579	LINC01514	0.038039	1.00E-06	15.2151917
ENSG00000231471	HMGN2P34	0.038031	1.00E-06	15.21488825
ENSG00000249410	AK4P2	0.037982	1.00E-06	15.21302825
ENSG00000259134	LINC00924	0.037954	1.00E-06	15.21196432
ENSG00000144010	TRIM43B	0.037954	1.00E-06	15.21196432
ENSG00000250561	OR7E59P	0.037954	1.00E-06	15.21196432
ENSG00000107295	SH3GL2	0.037953775	1.00E-06	15.21195577
ENSG00000217372	TUBB4BP7	0.037945	1.00E-06	15.21162217
ENSG00000279727	RP11-640L9.1	0.037869357	1.00E-06	15.20874331
ENSG00000105825	TFPI2	0.037842077	1.00E-06	15.20770367
ENSG00000244137	RP11-99J16__A.2	0.037841201	1.00E-06	15.20767028
ENSG00000215124	AMD1P4	0.037841	1.00E-06	15.20766259
ENSG00000179397	C1orf101	0.037822973	1.00E-06	15.20697516
ENSG00000250371	RP11-22A3.2	0.037785	1.00E-06	15.205526
ENSG00000173578	XCR1	0.037680517	1.00E-06	15.20153112
ENSG00000235478	AC006946.15	0.037589586	1.00E-06	15.19804542
ENSG00000224920	TACC1P1	0.037572	1.00E-06	15.19737029
ENSG00000125888	BANF2	0.037478763	1.00E-06	15.19378571
ENSG00000198178	CLEC4C	0.037409605	1.00E-06	15.19112113
ENSG00000066336	SPI1	0.037391408	1.00E-06	15.19041917
ENSG00000165118	C9orf64	0.037300286	1.00E-06	15.18689908
ENSG00000182782	HCAR2	0.037223	1.00E-06	15.18390671
ENSG00000214575	CPEB1	0.037173212	1.00E-06	15.18197573
ENSG00000249853	HS3ST5	0.037145999	1.00E-06	15.1809192
ENSG00000250894	PRAMEF31P	0.037124	1.00E-06	15.18006454
ENSG00000240388	CTD-2061E19.1	0.037124	1.00E-06	15.18006454
ENSG00000244384	RN7SL359P	0.037111	1.00E-06	15.17955926
ENSG00000258203	RP1-228P16.3	0.037050718	1.00E-06	15.1772139
ENSG00000180549	FUT7	0.03703	1.00E-06	15.17640693
ENSG00000270902	RP11-338O1.3	0.037016	1.00E-06	15.17586138
ENSG00000176136	MC5R	0.037015455	1.00E-06	15.17584012
ENSG00000272008	RP11-393I2.4	0.036997	1.00E-06	15.17512067
ENSG00000233708	RP11-342M1.7	0.036837	1.00E-06	15.16886795
ENSG00000250254	PTTG2	0.036827	1.00E-06	15.16847626
ENSG00000146049	KAAG1	0.036827	1.00E-06	15.16847626
ENSG00000255977	RP11-444J21.2	0.036793	1.00E-06	15.16714369
ENSG00000275465	RP11-569G13.3	0.036712	1.00E-06	15.16396409
ENSG00000280424	CITF22-92A6.2	0.036686	1.00E-06	15.16294199
ENSG00000274461	RP11-323P17.2	0.036676441	1.00E-06	15.16256602
ENSG00000144820	ADGRG7	0.03657628	1.00E-06	15.15862072
ENSG00000214604	NPM1P36	0.036572	1.00E-06	15.1584519
ENSG00000187258	NPSR1	0.036544972	1.00E-06	15.15738529
ENSG00000224107	LINC00633	0.036354699	1.00E-06	15.14985421
ENSG00000227183	HDGFP1	0.036354	1.00E-06	15.14982649
ENSG00000205864	KRTAP5-6	0.03629	1.00E-06	15.14728444
ENSG00000224679	MED15P4	0.036262	1.00E-06	15.14617088

ENSG00000279769	CH507-42P11.3	0.036251	1.00E-06	15.14573317
ENSG00000181036	FCRL6	0.036205122	1.00E-06	15.14390618
ENSG00000232864	NUCKS1P1	0.036199	1.00E-06	15.14366222
ENSG00000269107	RP11-15H20.7	0.036177	1.00E-06	15.14278516
ENSG00000223912	EEF1A1P36	0.036166	1.00E-06	15.14234642
ENSG00000234706	PRUNEP1	0.036097	1.00E-06	15.13959132
ENSG00000179071	CCDC89	0.036046	1.00E-06	15.13755155
ENSG00000180869	LINC01555	0.036002	1.00E-06	15.13578943
ENSG00000187862	TTC24	0.03598166	1.00E-06	15.13497412
ENSG00000162888	C1orf147	0.035938	1.00E-06	15.1332225
ENSG00000236212	AC009262.2	0.035927	1.00E-06	15.13278085
ENSG00000232199	RPL3P8	0.035896	1.00E-06	15.13153547
ENSG00000274281	RP11-326N17.2	0.035893	1.00E-06	15.13141489
ENSG00000233220	LINC00167	0.035868	1.00E-06	15.13040968
ENSG00000249481	SPATS1	0.035853816	1.00E-06	15.12983907
ENSG00000275672	GATM-AS1	0.035741	1.00E-06	15.12529238
ENSG00000237301	RP4-680D5.2	0.035585	1.00E-06	15.11898162
ENSG00000237833	RP11-432N13.2	0.035542	1.00E-06	15.11723725
ENSG00000109625	CPZ	0.035519066	1.00E-06	15.11630603
ENSG00000267382	RP11-325K19.2	0.035495115	1.00E-06	15.11533288
ENSG00000182447	OTOL1	0.035493	1.00E-06	15.1152469
ENSG00000188674	C2orf80	0.035468053	1.00E-06	15.11423251
ENSG00000171201	SMR3B	0.035443078	1.00E-06	15.11321628
ENSG00000244503	RP11-278L15.6	0.035429	1.00E-06	15.11264312
ENSG00000164929	BAALC	0.035399092	1.00E-06	15.11142475
ENSG00000162366	PDZK1IP1	0.035393745	1.00E-06	15.11120682
ENSG00000248576	RP11-834C11.8	0.035362	1.00E-06	15.10991225
ENSG00000272768	RP4-673M15.1	0.03532	1.00E-06	15.10819772
ENSG00000223965	ZNF587P1	0.035253	1.00E-06	15.10545841
ENSG00000261166	RP11-775H9.3	0.035247	1.00E-06	15.10521285
ENSG00000188050	RNF133	0.035222	1.00E-06	15.10418921
ENSG00000246777	RP11-61A14.4	0.035173	1.00E-06	15.10218077
ENSG00000260439	LMF1-AS1	0.035149436	1.00E-06	15.10121394
ENSG00000215515	IFIT1P1	0.035126	1.00E-06	15.10025168
ENSG00000280870	MIR325HG	0.035120426	1.00E-06	15.10002273
ENSG00000223349	KLF2P3	0.035117	1.00E-06	15.09988198
ENSG00000242307	RPS26P52	0.035101	1.00E-06	15.09922451
ENSG00000233579	KRT8P15	0.035077	1.00E-06	15.09823774
ENSG00000261043	RP11-24M17.6	0.03504	1.00E-06	15.09671515
ENSG00000232613	AC007386.4	0.035038835	1.00E-06	15.09666719
ENSG00000122133	PAEP	0.035004058	1.00E-06	15.09523455
ENSG00000278434	RP11-709D24.8	0.03498	1.00E-06	15.09424267
ENSG00000283020	DUX4L37	0.034957	1.00E-06	15.09329376
ENSG00000172005	MAL	0.034932217	1.00E-06	15.09227058
ENSG00000218186	KRT8P43	0.034909	1.00E-06	15.09131141
ENSG00000237426	ZIK1P1	0.034884	1.00E-06	15.09027786
ENSG00000224961	RP1-278O22.1	0.034861	1.00E-06	15.08932633
ENSG00000269058	CALR3	0.034860992	1.00E-06	15.08932599

ENSG00000235947	EGOT	0.034765	1.00E-06	15.08534797
ENSG00000254528	RP11-728F11.4	0.03476499	1.00E-06	15.08534757
ENSG00000260834	RP11-256I9.2	0.03476474	1.00E-06	15.08533717
ENSG00000254402	LRRC24	0.034717524	1.00E-06	15.08337643
ENSG00000213694	S1PR3	0.034651511	1.00E-06	15.08063066
ENSG00000257210	NACAP3	0.034566	1.00E-06	15.07706604
ENSG00000241933	RP11-755B10.3	0.034524	1.00E-06	15.07531201
ENSG00000237685	RP11-360O19.4	0.034506	1.00E-06	15.07455962
ENSG00000226097	AC099342.1	0.034458888	1.00E-06	15.07258854
ENSG00000259221	CTD-2050N2.1	0.03436642	1.00E-06	15.06871195
ENSG00000248313	CYP4F27P	0.034343	1.00E-06	15.06772845
ENSG00000249310	APOBEC3B-AS1	0.034259	1.00E-06	15.06419542
ENSG00000167014	C15orf43	0.034227169	1.00E-06	15.06285434
ENSG00000215223	CYP51A1P3	0.034215	1.00E-06	15.06234133
ENSG00000231536	AC123886.2	0.034067	1.00E-06	15.05608729
ENSG00000231017	AC013404.1	0.034067	1.00E-06	15.05608729
ENSG00000260029	RP11-401P9.1	0.03405	1.00E-06	15.05536718
ENSG00000128285	MCHR1	0.034033873	1.00E-06	15.0546837
ENSG00000261618	RP11-79H23.3	0.033999	1.00E-06	15.05320469
ENSG00000137273	FOXF2	0.033977	1.00E-06	15.05227085
ENSG00000167641	PPP1R14A	0.033907941	1.00E-06	15.04933555
ENSG00000145283	SLC10A6	0.033886	1.00E-06	15.04840173
ENSG00000257777	RP11-25J3.2	0.033818	1.00E-06	15.04550372
ENSG00000267310	OR4G1P	0.033677	1.00E-06	15.03947601
ENSG00000261080	RP1-166H4.2	0.033639	1.00E-06	15.0378472
ENSG00000237971	RP11-231N9.1	0.033632	1.00E-06	15.03754695
ENSG00000248231	KRT8P4	0.033604	1.00E-06	15.03634535
ENSG00000271917	RP11-506O24.2	0.033551	1.00E-06	15.03406815
ENSG00000234984	FMO10P	0.033408	1.00E-06	15.027906
ENSG00000214295	FOXO1B	0.033331	1.00E-06	15.02457698
ENSG00000224286	LINC01142	0.033330664	1.00E-06	15.02456244
ENSG00000184530	C6orf58	0.03326538	1.00E-06	15.02173391
ENSG00000227744	FLJ43879	0.03326	1.00E-06	15.02150055
ENSG00000264187	RP11-45M22.4	0.033215	1.00E-06	15.01954729
ENSG00000259814	RP11-300A12.2	0.033114	1.00E-06	15.01515367
ENSG00000249069	LINC01033	0.033096254	1.00E-06	15.0143803
ENSG00000227995	RAB11AP1	0.033091	1.00E-06	15.01415127
ENSG00000223628	AC023449.2	0.03305	1.00E-06	15.01236265
ENSG00000260493	RP11-219B4.7	0.033049496	1.00E-06	15.01234066
ENSG00000177791	MYOZ1	0.033028	1.00E-06	15.01140199
ENSG00000203729	LINC00272	0.033013	1.00E-06	15.01074663
ENSG00000254743	OR10V3P	0.032995	1.00E-06	15.0099598
ENSG00000177340	FLJ13224	0.032945	1.00E-06	15.00777191
ENSG00000029559	IBSP	0.032922	1.00E-06	15.00676436
ENSG00000270074	RP11-351I21.11	0.0329	1.00E-06	15.00579996
ENSG00000225569	CCT4P2	0.032879	1.00E-06	15.0048788
ENSG00000197272	IL27	0.032862161	1.00E-06	15.00413972
ENSG00000279705	RP11-758M2.1	0.032815	1.00E-06	15.00206781

ENSG00000106631	MYL7	0.032813479	1.00E-06	15.00200096
ENSG00000240579	RP11-20L24.1	0.032759	1.00E-06	14.9996037
ENSG00000231672	DIRC3	0.03273039	1.00E-06	14.99834316
ENSG00000242703	CCT4P1	0.032667	1.00E-06	14.99554635
ENSG00000248550	OTX2-AS1	0.032646399	1.00E-06	14.99463625
ENSG00000109205	ODAM	0.032584029	1.00E-06	14.99187737
ENSG00000189167	ZAR1L	0.032542	1.00E-06	14.9900153
ENSG00000157765	SLC34A2	0.032493536	1.00E-06	14.98786511
ENSG00000278891	RP11-246E12.2	0.032478	1.00E-06	14.98717517
ENSG00000226486	LINC01035	0.03246	1.00E-06	14.98637538
ENSG00000258733	CTD-2341M24.1	0.032459372	1.00E-06	14.98634749
ENSG00000280273	AF131216.1	0.032438	1.00E-06	14.98539725
ENSG00000125414	MYH2	0.032430067	1.00E-06	14.98504441
ENSG00000234227	RPL7L1P1	0.032418	1.00E-06	14.98450747
ENSG00000247213	LINC01498	0.032387271	1.00E-06	14.98313928
ENSG00000281228	AL132640.1	0.03235	1.00E-06	14.98147809
ENSG00000257588	RP11-469H8.6	0.032335671	1.00E-06	14.98083892
ENSG00000178248	AP000345.1	0.032254	1.00E-06	14.97719047
ENSG00000174450	GOLGA6L2	0.032251	1.00E-06	14.97705627
ENSG00000250107	CACNA1G-AS1	0.032232971	1.00E-06	14.97624955
ENSG00000183813	CCR4	0.032232	1.00E-06	14.97620609
ENSG00000225196	RP5-1118D24.2	0.032213	1.00E-06	14.9753554
ENSG00000185666	SYN3	0.032160873	1.00E-06	14.97301893
ENSG00000267610	AC007787.2	0.032152	1.00E-06	14.97262086
ENSG00000255106	RP11-58K22.2	0.032132	1.00E-06	14.97172316
ENSG00000146469	VIP	0.032132	1.00E-06	14.97172316
ENSG00000229920	RPS4XP5	0.032131394	1.00E-06	14.97169595
ENSG00000174255	ZNF80	0.032112942	1.00E-06	14.97086721
ENSG00000272347	RP11-43F13.4	0.032111	1.00E-06	14.97077997
ENSG00000036473	OTC	0.032111	1.00E-06	14.97077997
ENSG00000140506	LMAN1L	0.032106357	1.00E-06	14.97057135
ENSG00000189393	FAM90A14P	0.032106	1.00E-06	14.97055531
ENSG00000092067	CEBPE	0.032097	1.00E-06	14.97015084
ENSG00000145908	ZNF300	0.032077617	1.00E-06	14.96927936
ENSG00000255966	RP5-940J5.3	0.032011	1.00E-06	14.96628013
ENSG00000260271	RP1-45N11.1	0.032011	1.00E-06	14.96628013
ENSG00000280188	AC005477.1	0.03193	1.00E-06	14.96262493
ENSG00000170948	MBD3L1	0.031877152	1.00E-06	14.96023514
ENSG00000204478	PRAMEF20	0.03187	1.00E-06	14.9599114
ENSG00000173369	C1QB	0.031790192	1.00E-06	14.95629413
ENSG00000224865	AC009518.4	0.031789724	1.00E-06	14.95627287
ENSG00000114405	C3orf14	0.031709881	1.00E-06	14.95264487
ENSG00000229056	AC020571.3	0.031671599	1.00E-06	14.9509021
ENSG00000261701	HPR	0.031612281	1.00E-06	14.94819751
ENSG00000234986	XX-C2158C12.1	0.031573	1.00E-06	14.94640373
ENSG00000271519	RP11-307A17.2	0.031557	1.00E-06	14.94567244
ENSG00000164303	ENPP6	0.031383386	1.00E-06	14.93771339
ENSG00000257611	RP11-185N2.1	0.031365	1.00E-06	14.93686794

ENSG00000259271	ANKRD62P1	0.031359	1.00E-06	14.93659193
ENSG00000113302	IL12B	0.031338	1.00E-06	14.93562549
ENSG00000136110	LECT1	0.03126331	1.00E-06	14.93218289
ENSG00000258702	RP11-433J8.1	0.031243	1.00E-06	14.93124537
ENSG00000249326	CTD-2194D22.4	0.031129	1.00E-06	14.92597161
ENSG00000156564	LRFN2	0.031129	1.00E-06	14.92597161
ENSG00000180043	FAM71E2	0.031091295	1.00E-06	14.92422309
ENSG00000212128	TAS2R13	0.031091	1.00E-06	14.9242094
ENSG00000196353	CPNE4	0.031043361	1.00E-06	14.92199716
ENSG00000240271	RP11-200A13.2	0.031042	1.00E-06	14.92193389
ENSG00000266495	RP11-17J14.2	0.031007	1.00E-06	14.92030633
ENSG00000120952	PRAMEF2	0.030997	1.00E-06	14.91984097
ENSG00000250267	RP11-122A21.2	0.030977	1.00E-06	14.91890981
ENSG00000279951	RP11-264J4.8	0.030948	1.00E-06	14.91755856
ENSG00000223382	RP5-1125N11.2	0.030921519	1.00E-06	14.91632356
ENSG00000225069	RP5-1025A1.2	0.030884	1.00E-06	14.914572
ENSG00000274744	TCEB3CL2	0.03086	1.00E-06	14.91345044
ENSG00000278674	RP11-49K24.9	0.03086	1.00E-06	14.91345044
ENSG00000211573	AC125238.1	0.030841	1.00E-06	14.91256192
ENSG00000184560	C17orf74	0.030828	1.00E-06	14.91195368
ENSG00000183607	GKN2	0.030790108	1.00E-06	14.91017929
ENSG00000146678	IGFBP1	0.03079	1.00E-06	14.91017425
ENSG00000035720	STAP1	0.030753187	1.00E-06	14.90844829
ENSG00000239828	RP11-446H18.5	0.030753154	1.00E-06	14.90844678
ENSG00000132464	ENAM	0.030746409	1.00E-06	14.90813032
ENSG00000267522	CTD-2621I17.6	0.030642	1.00E-06	14.90322284
ENSG00000147613	PSKH2	0.030623152	1.00E-06	14.90233515
ENSG00000213612	FAM220CP	0.030623	1.00E-06	14.902328
ENSG00000254777	AC022182.1	0.03062	1.00E-06	14.90218666
ENSG00000261665	TUBAP4	0.030587	1.00E-06	14.90063099
ENSG00000224707	E2F3-IT1	0.030538	1.00E-06	14.89831796
ENSG00000267262	CTC-232P5.3	0.030519	1.00E-06	14.89742007
ENSG00000280435	RP4-555D20.1	0.030477	1.00E-06	14.89543328
ENSG00000272874	RP5-1103G7.10	0.03044	1.00E-06	14.89368074
ENSG00000142583	SLC2A5	0.03041763	1.00E-06	14.89262015
ENSG00000278979	RP11-165M1.1	0.030331	1.00E-06	14.88850544
ENSG00000116824	CD2	0.030313215	1.00E-06	14.88765925
ENSG00000270394	MTRNR2L13	0.030291	1.00E-06	14.88660159
ENSG00000261693	RP13-467H17.1	0.030143	1.00E-06	14.87953539
ENSG00000230725	RP4-738P15.1	0.030125533	1.00E-06	14.87869915
ENSG00000238099	LINC01625	0.030116	1.00E-06	14.87824254
ENSG00000188906	LRRK2	0.030079592	1.00E-06	14.87649738
ENSG00000225030	RP4-784A16.3	0.030065	1.00E-06	14.87579734
ENSG00000253944	RP11-156K13.1	0.029939	1.00E-06	14.86973841
ENSG00000271824	AC009014.3	0.029921	1.00E-06	14.86887077
ENSG00000226358	KRT8P38	0.029897	1.00E-06	14.8677131
ENSG00000243658	MTND5P16	0.029886	1.00E-06	14.8671822
ENSG00000235601	BARX1-AS1	0.029868421	1.00E-06	14.86633337

ENSG00000272754	AL133245.2	0.029829	1.00E-06	14.86442799
ENSG00000211898	IGHD	0.029798254	1.00E-06	14.86294018
ENSG00000163581	SLC2A2	0.029789965	1.00E-06	14.8625388
ENSG00000232795	SCAND3P1	0.029769	1.00E-06	14.86152314
ENSG00000184682	PRR33	0.029764	1.00E-06	14.8612808
ENSG00000280043	RP11-1281K21.8	0.029729	1.00E-06	14.85958332
ENSG00000282041	CTA-286B10.8	0.02969	1.00E-06	14.85768947
ENSG00000175336	APOF	0.029678	1.00E-06	14.85710625
ENSG00000260572	RP11-16N11.2	0.029678	1.00E-06	14.85710625
ENSG00000187483	SERPINA13P	0.029626	1.00E-06	14.85457623
ENSG00000118113	MMP8	0.029608889	1.00E-06	14.85374273
ENSG00000243122	RP11-25H11.1	0.029577	1.00E-06	14.85218811
ENSG00000268079	BNIP3P30	0.029556	1.00E-06	14.85116341
ENSG00000226933	NRBF2P2	0.029556	1.00E-06	14.85116341
ENSG00000227236	RP11-365D9.1	0.029522	1.00E-06	14.84950284
ENSG00000180999	C1orf105	0.02950489	1.00E-06	14.84866645
ENSG00000279925	RP11-481J8.3	0.02947	1.00E-06	14.84695944
ENSG00000256263	DDX11L8	0.029322	1.00E-06	14.83969589
ENSG00000232053	AC009784.3	0.029309732	1.00E-06	14.83909217
ENSG00000002933	TMEM176A	0.02925902	1.00E-06	14.83659381
ENSG00000267648	RP11-686D22.3	0.029217	1.00E-06	14.83452043
ENSG00000103313	MEFV	0.02915033	1.00E-06	14.83122459
ENSG00000257477	LINC01154	0.029147	1.00E-06	14.83105978
ENSG00000175097	RAG2	0.029066761	1.00E-06	14.82708268
ENSG00000280997	CCAT2	0.029051	1.00E-06	14.8263002
ENSG00000230469	RPL5P26	0.029017	1.00E-06	14.82461075
ENSG00000238164	RP3-395M20.8	0.028943873	1.00E-06	14.82097036
ENSG00000262079	RP11-515O17.3	0.028869	1.00E-06	14.81723351
ENSG00000273639	RP11-597A11.11	0.028860481	1.00E-06	14.81680773
ENSG00000163565	IFI16	0.028840938	1.00E-06	14.81583045
ENSG00000261382	RP11-461L18.1	0.028837	1.00E-06	14.81563346
ENSG00000246211	RP11-632K5.3	0.028787	1.00E-06	14.81312983
ENSG00000186474	KLK12	0.028771153	1.00E-06	14.81233543
ENSG00000226010	RP11-403E24.1	0.028739	1.00E-06	14.81072224
ENSG00000053328	METTL24	0.028690302	1.00E-06	14.80827554
ENSG00000164082	GRM2	0.02868948	1.00E-06	14.8082342
ENSG00000259697	RP11-299H22.1	0.028645	1.00E-06	14.80599572
ENSG00000165471	MBL2	0.028521	1.00E-06	14.79973695
ENSG00000247193	RP11-431M7.3	0.028513026	1.00E-06	14.79933355
ENSG00000261760	RP11-1223D19.1	0.028465774	1.00E-06	14.7969407
ENSG00000273523	RP11-248G5.9	0.028465	1.00E-06	14.79690148
ENSG00000187546	AGMO	0.028378111	1.00E-06	14.79249094
ENSG00000259711	CTD-3032H12.2	0.028354	1.00E-06	14.79126466
ENSG00000213130	EEF1DP5	0.028349	1.00E-06	14.79101023
ENSG00000196224	KRTAP5-3	0.028307	1.00E-06	14.78887124
ENSG00000133055	MYBPH	0.028198	1.00E-06	14.78330522
ENSG00000267053	CTD-3162L10.1	0.028169445	1.00E-06	14.7818435
ENSG00000227836	AC008850.3	0.028119	1.00E-06	14.77925767

ENSG00000187980	PLA2G2C	0.028087398	1.00E-06	14.77763537
ENSG00000215197	PGAM4P1	0.028016	1.00E-06	14.77396337
ENSG00000163219	ARHGAP25	0.028006129	1.00E-06	14.77345496
ENSG00000179840	PIK3CD-AS1	0.027919	1.00E-06	14.76895965
ENSG00000099960	SLC7A4	0.027908183	1.00E-06	14.7684006
ENSG00000221365	MIR1228	0.027888	1.00E-06	14.76735685
ENSG00000273630	MIR6755	0.027874	1.00E-06	14.76663243
ENSG00000257178	RP11-357H14.16	0.027867	1.00E-06	14.76627008
ENSG00000231881	RP5-1120P11.3	0.027853	1.00E-06	14.76554511
ENSG00000188831	DPPA3P2	0.027834971	1.00E-06	14.76461097
ENSG00000227082	CH17-437K3.1	0.027796	1.00E-06	14.76258967
ENSG00000184735	DDX53	0.027769	1.00E-06	14.7611876
ENSG00000225463	ZNF70P1	0.027732	1.00E-06	14.75926405
ENSG00000246740	PLA2G4E-AS1	0.027699	1.00E-06	14.75754627
ENSG00000177414	UBE2U	0.02769086	1.00E-06	14.75712222
ENSG00000234982	RP11-280O24.3	0.027633	1.00E-06	14.75410458
ENSG00000143416	SELENBP1	0.027598413	1.00E-06	14.7522977
ENSG00000168754	FAM178B	0.027565554	1.00E-06	14.75057899
ENSG00000178055	PRSS42	0.027407302	1.00E-06	14.7422727
ENSG00000248144	ADH1C	0.027363839	1.00E-06	14.73998301
ENSG00000218048	RP3-407E4.4	0.027341	1.00E-06	14.73877839
ENSG00000226806	AC011893.3	0.027334	1.00E-06	14.73840898
ENSG00000280240	AL049794.1	0.027305	1.00E-06	14.73687754
ENSG00000230817	LINC01362	0.027101197	1.00E-06	14.72606895
ENSG00000117560	FASLG	0.026958	1.00E-06	14.71842585
ENSG00000250041	CTD-2003C8.2	0.026872645	1.00E-06	14.71385072
ENSG00000232920	LINC01400	0.026872	1.00E-06	14.71381608
ENSG00000226213	UBQLN4P2	0.026844	1.00E-06	14.71231204
ENSG00000184814	PRR23B	0.026844	1.00E-06	14.71231204
ENSG00000227630	LINC01132	0.026837	1.00E-06	14.71193579
ENSG00000271049	CTD-2553L13.7	0.026788	1.00E-06	14.70929925
ENSG00000268886	CTD-3099C6.7	0.026728	1.00E-06	14.70606427
ENSG00000177354	C10orf71	0.026713498	1.00E-06	14.7052813
ENSG00000224204	PHEX-AS1	0.026703	1.00E-06	14.70471421
ENSG00000170236	USP50	0.026689124	1.00E-06	14.70396435
ENSG00000261257	RP11-673E11.2	0.026661	1.00E-06	14.70244327
ENSG00000172367	PDZD3	0.026598405	1.00E-06	14.69905212
ENSG00000154485	MMP21	0.026522	1.00E-06	14.69490195
ENSG00000231298	LINC00704	0.026508592	1.00E-06	14.69417243
ENSG00000229266	POM121L8P	0.026487	1.00E-06	14.69299683
ENSG00000229418	RP11-35J23.1	0.026454	1.00E-06	14.69119826
ENSG00000229191	RP11-168O16.1	0.02640636	1.00E-06	14.68859784
ENSG00000187690	CXorf67	0.026385	1.00E-06	14.68743036
ENSG00000233694	AC007365.1	0.026384361	1.00E-06	14.68739541
ENSG00000177047	IFNW1	0.026344	1.00E-06	14.6851868
ENSG00000280228	RP11-67L14.1	0.026316	1.00E-06	14.6836526
ENSG00000110887	DAO	0.026215185	1.00E-06	14.67811512
ENSG00000240563	L1TD1	0.026182	1.00E-06	14.67628769

ENSG00000280276	RP11-314C9.1	0.026141	1.00E-06	14.67402671
ENSG00000234944	RP11-124O11.1	0.026131	1.00E-06	14.67347472
ENSG00000258274	RP11-887P2.5	0.026114252	1.00E-06	14.67254976
ENSG00000261519	RP11-403P17.4	0.026114	1.00E-06	14.67253584
ENSG00000275553	TCEB3CL	0.026050414	1.00E-06	14.66901866
ENSG00000258716	RP11-661G16.2	0.026048	1.00E-06	14.66888498
ENSG00000213661	RP11-510I6.2	0.026008	1.00E-06	14.66666784
ENSG00000173610	UGT2A1	0.025973868	1.00E-06	14.66477323
ENSG00000251270	RP11-231L11.1	0.025967	1.00E-06	14.66439173
ENSG00000129988	LBP	0.025954	1.00E-06	14.66366928
ENSG00000259478	RP11-753A21.1	0.025928	1.00E-06	14.66222331
ENSG00000279417	RP11-382B18.1	0.025901	1.00E-06	14.66072018
ENSG00000185052	SLC24A3	0.025881651	1.00E-06	14.65964201
ENSG00000255835	RP4-559A3.7	0.025849	1.00E-06	14.65782085
ENSG00000204930	FAM221B	0.025829209	1.00E-06	14.65671585
ENSG00000267727	CTD-2540B15.7	0.025827	1.00E-06	14.65659245
ENSG00000107447	DNTT	0.025809	1.00E-06	14.65558662
ENSG00000169194	IL13	0.025781733	1.00E-06	14.6540616
ENSG00000162892	IL24	0.025718767	1.00E-06	14.65053383
ENSG00000183785	TUBA8	0.025642242	1.00E-06	14.64623477
ENSG00000231095	GYG1P3	0.025602	1.00E-06	14.6439689
ENSG00000198681	MAGEA1	0.025597	1.00E-06	14.64368711
ENSG00000179577	KB-1552D7.2	0.025589	1.00E-06	14.64323615
ENSG00000233143	DIRC3-AS1	0.025586	1.00E-06	14.643067
ENSG00000046774	MAGEC2	0.025563	1.00E-06	14.64176954
ENSG00000138615	CILP	0.02556	1.00E-06	14.64160022
ENSG00000265746	KYNUP2	0.025532	1.00E-06	14.64001893
ENSG00000149575	SCN2B	0.025509	1.00E-06	14.63871872
ENSG00000243137	PSG4	0.025495758	1.00E-06	14.63796961
ENSG00000180209	MYLPF	0.025471217	1.00E-06	14.63658028
ENSG00000206702	RNU1-11P	0.025448	1.00E-06	14.63526466
ENSG00000264120	AF274855.1	0.025448	1.00E-06	14.63526466
ENSG00000224586	GPX5	0.025422626	1.00E-06	14.63382546
ENSG00000178522	AMBN	0.025334457	1.00E-06	14.62881331
ENSG00000259238	RP11-361D15.2	0.025322	1.00E-06	14.62810374
ENSG00000263206	KYNUP3	0.025303	1.00E-06	14.62702082
ENSG00000004809	SLC22A16	0.025272157	1.00E-06	14.62526119
ENSG00000234564	HSPA8P20	0.025271	1.00E-06	14.62519513
ENSG00000257467	RP11-121G22.3	0.025146587	1.00E-06	14.61807498
ENSG00000233176	OR7E157P	0.025141	1.00E-06	14.61775441
ENSG00000138650	PCDH10	0.025045098	1.00E-06	14.61224064
ENSG00000082074	FYB	0.025030943	1.00E-06	14.611425
ENSG00000250895	CTD-2158P22.4	0.025023	1.00E-06	14.61096714
ENSG00000105131	EPHX3	0.025022492	1.00E-06	14.61093783
ENSG00000226870	RP11-402P6.13	0.025020704	1.00E-06	14.61083475
ENSG00000186334	SLC36A3	0.024919697	1.00E-06	14.60499891
ENSG00000176160	HSF5	0.024864	1.00E-06	14.60177079
ENSG00000234773	CTD-2666L21.1	0.024827307	1.00E-06	14.59964013

ENSG00000253781	ZNF317P1	0.024779	1.00E-06	14.59683035
ENSG00000205856	C22orf42	0.024766836	1.00E-06	14.59612198
ENSG00000258735	LINC00637	0.024755	1.00E-06	14.59543233
ENSG00000253199	RP11-421P23.1	0.024719085	1.00E-06	14.59333772
ENSG00000149633	KIAA1755	0.024683574	1.00E-06	14.59126365
ENSG00000254715	OR7E154P	0.024575	1.00E-06	14.5849038
ENSG00000197134	ZNF257	0.024571135	1.00E-06	14.5846769
ENSG00000205111	CDKL4	0.024540096	1.00E-06	14.5828533
ENSG00000125355	TMEM255A	0.024537843	1.00E-06	14.58272084
ENSG00000276122	RP11-143E21.3	0.024517	1.00E-06	14.58149484
ENSG00000261751	RP11-305A4.3	0.024411	1.00E-06	14.57524378
ENSG00000187140	FOXD3	0.024399	1.00E-06	14.5745344
ENSG00000162878	PKDCC	0.024378276	1.00E-06	14.57330849
ENSG00000061492	WNT8A	0.024363833	1.00E-06	14.57245352
ENSG00000256249	RP11-324E6.6	0.024352437	1.00E-06	14.57177852
ENSG00000166796	LDHC	0.024294232	1.00E-06	14.56832623
ENSG00000233912	AC026202.3	0.024237	1.00E-06	14.56492352
ENSG00000278949	RP11-46H11.11	0.02419	1.00E-06	14.56212315
ENSG00000257108	NHLRC4	0.024178759	1.00E-06	14.56145256
ENSG00000261987	KYNUP1	0.024133	1.00E-06	14.55871965
ENSG00000222028	PSMB11	0.024122	1.00E-06	14.55806191
ENSG00000184108	TRIML1	0.024087284	1.00E-06	14.55598411
ENSG00000250658	RP11-138B4.1	0.024076	1.00E-06	14.5553081
ENSG00000275208	MIR6745	0.024045	1.00E-06	14.55344931
ENSG00000279733	RP11-685B24.1	0.024041	1.00E-06	14.55320929
ENSG00000229791	RP11-385E5.5	0.02403	1.00E-06	14.55254903
ENSG00000186458	DEFB132	0.023997	1.00E-06	14.55056644
ENSG00000112562	SMOC2	0.023984375	1.00E-06	14.54980722
ENSG00000012223	LTF	0.023968079	1.00E-06	14.54882664
ENSG00000269927	RP6-91H8.3	0.023906	1.00E-06	14.54508514
ENSG00000279312	RP3-331H24.7	0.023839	1.00E-06	14.5410361
ENSG00000179520	SLC17A8	0.023828074	1.00E-06	14.54037471
ENSG00000214819	CDRT15L2	0.023797	1.00E-06	14.53849209
ENSG00000230174	LINC01149	0.023773	1.00E-06	14.53703635
ENSG00000251539	SNX18P24	0.023766	1.00E-06	14.53661149
ENSG00000197880	MDS2	0.023760802	1.00E-06	14.53629589
ENSG00000239539	HMGN1P20	0.023751	1.00E-06	14.53570064
ENSG00000230523	RP3-437I16.1	0.023694	1.00E-06	14.53223415
ENSG00000204365	C10orf126	0.023684	1.00E-06	14.53162514
ENSG00000271500	RP11-288K12.1	0.023661	1.00E-06	14.53022343
ENSG00000162947	AC007364.1	0.023639863	1.00E-06	14.52893402
ENSG00000231140	RP11-347J14.7	0.023629	1.00E-06	14.52827095
ENSG00000251504	LINC01099	0.023628016	1.00E-06	14.52821089
ENSG00000172673	THEMIS	0.023622864	1.00E-06	14.52789627
ENSG00000186867	QRFPR	0.023563283	1.00E-06	14.52425296
ENSG00000280243	MTCO1P1	0.023542	1.00E-06	14.52294927
ENSG00000234807	LINC01135	0.023506	1.00E-06	14.52074144
ENSG00000282943	CTC-490G23.6	0.023476435	1.00E-06	14.51892572

ENSG00000227013	OR7E96P	0.023466	1.00E-06	14.51828432
ENSG00000223675	RP11-86H7.6	0.023444	1.00E-06	14.51693112
ENSG00000144852	NR1I2	0.023436942	1.00E-06	14.51649671
ENSG00000236514	RP11-162G10.5	0.023414	1.00E-06	14.5150838
ENSG00000248837	RP11-412P11.1	0.023400707	1.00E-06	14.51426451
ENSG00000255871	RP11-69C13.1	0.023368	1.00E-06	14.51224664
ENSG00000227437	RP11-179H18.5	0.02327	1.00E-06	14.50618359
ENSG00000228347	FTLP17	0.023267	1.00E-06	14.50599758
ENSG00000223414	LINC00473	0.023261384	1.00E-06	14.50564931
ENSG00000213996	TM6SF2	0.023261235	1.00E-06	14.5056401
ENSG00000223893	GNL2P1	0.02324	1.00E-06	14.50432245
ENSG00000156140	ADAMTS3	0.023187209	1.00E-06	14.50104156
ENSG00000198075	SULT1C4	0.023182594	1.00E-06	14.50075435
ENSG00000240654	C1QTNF9	0.023121997	1.00E-06	14.49697837
ENSG00000237540	RP11-730A19.7	0.023109	1.00E-06	14.49616721
ENSG00000280200	RP11-243M5.2	0.023101	1.00E-06	14.49566768
ENSG00000062096	ARSF	0.023092158	1.00E-06	14.49511537
ENSG00000231386	AC007395.4	0.023055	1.00E-06	14.49279205
ENSG00000250456	AC006552.1	0.022967	1.00E-06	14.4872748
ENSG00000261842	RP11-143I21.1	0.022967	1.00E-06	14.4872748
ENSG00000184459	BPIFC	0.022844269	1.00E-06	14.47954465
ENSG00000267413	RP11-636O21.1	0.022824	1.00E-06	14.47826403
ENSG00000259293	RP11-355N15.1	0.022813	1.00E-06	14.47756856
ENSG00000169562	GJB1	0.022773033	1.00E-06	14.47503881
ENSG00000220008	LINGO3	0.022711	1.00E-06	14.47110361
ENSG00000249557	HSPD1P15	0.022681	1.00E-06	14.46919663
ENSG00000145934	TENM2	0.022676746	1.00E-06	14.46892604
ENSG00000160185	UBASH3A	0.022619817	1.00E-06	14.46529964
ENSG00000103426	CORO7-PAM16	0.022589064	1.00E-06	14.46333689
ENSG00000215088	RPS5P3	0.022584	1.00E-06	14.46301341
ENSG00000253558	CTD-2179L22.1	0.022521	1.00E-06	14.45898327
ENSG00000250986	AC141928.1	0.022495	1.00E-06	14.45731675
ENSG00000131183	SLC34A1	0.022476185	1.00E-06	14.45610959
ENSG00000105967	TFEC	0.022473794	1.00E-06	14.45595605
ENSG00000232520	AC012507.3	0.022470759	1.00E-06	14.45576123
ENSG00000278897	AC020951.1	0.022412	1.00E-06	14.45198378
ENSG00000204173	LRRC37A5P	0.022411096	1.00E-06	14.45192561
ENSG00000204583	LRCOL1	0.022408938	1.00E-06	14.45178665
ENSG00000223653	RP11-131L23.1	0.022352635	1.00E-06	14.44815729
ENSG00000259793	RP11-400N9.1	0.022274	1.00E-06	14.44307304
ENSG00000224333	GAPDHP20	0.022239	1.00E-06	14.4408043
ENSG00000187848	P2RX2	0.02223	1.00E-06	14.44022033
ENSG00000240103	RP11-846F4.6	0.022198	1.00E-06	14.43814208
ENSG00000186075	ZPBP2	0.022176942	1.00E-06	14.43677286
ENSG00000265069	RP11-781P6.1	0.022176	1.00E-06	14.43671154
ENSG00000218754	RPL18AP8	0.02217	1.00E-06	14.43632115
ENSG00000249504	PCDHA14	0.022148	1.00E-06	14.43488881
ENSG00000251483	LYPLA1P2	0.022097	1.00E-06	14.43156289

ENSG00000269138	ZNF209P	0.02209	1.00E-06	14.4311058
ENSG00000233757	AC092835.2	0.022089996	1.00E-06	14.43110551
ENSG00000253810	PSAT1P1	0.022089	1.00E-06	14.43104049
ENSG00000247033	RP11-252E2.1	0.022043	1.00E-06	14.42803296
ENSG00000260784	AC026150.6	0.022014	1.00E-06	14.42613369
ENSG00000149305	HTR3B	0.021994987	1.00E-06	14.4248871
ENSG00000134216	CHIA	0.021975766	1.00E-06	14.42362586
ENSG00000267551	AC005264.2	0.021947	1.00E-06	14.42173613
ENSG00000260278	RP11-109G23.3	0.021938	1.00E-06	14.42114439
ENSG00000278499	RP11-457D20.2	0.021926	1.00E-06	14.42035502
ENSG00000184274	LINC00315	0.02191	1.00E-06	14.41930186
ENSG00000227964	LINC01429	0.021881	1.00E-06	14.41739105
ENSG00000260008	RP11-732A21.2	0.021846	1.00E-06	14.41508153
ENSG00000214146	RP11-699L21.1	0.02179718	1.00E-06	14.4118539
ENSG00000233393	AP000688.29	0.021788	1.00E-06	14.41124615
ENSG00000205420	KRT6A	0.021785635	1.00E-06	14.41108956
ENSG00000164100	NDST3	0.021784993	1.00E-06	14.41104702
ENSG00000204044	RP11-465L10.10	0.021773805	1.00E-06	14.41030594
ENSG00000101251	SEL1L2	0.021752854	1.00E-06	14.40891706
ENSG00000164736	SOX17	0.021732	1.00E-06	14.40753333
ENSG00000132911	NMUR2	0.021704177	1.00E-06	14.40568512
ENSG00000278989	RP11-762L8.6	0.021575	1.00E-06	14.39707294
ENSG00000224675	AC009227.2	0.021548	1.00E-06	14.39526635
ENSG00000175311	ANKS4B	0.021519	1.00E-06	14.39332342
ENSG00000214248	CTD-3193O13.12	0.021479	1.00E-06	14.39063921
ENSG00000112303	VNN2	0.021456182	1.00E-06	14.38910578
ENSG00000267136	RP11-53B2.3	0.021453	1.00E-06	14.38889179
ENSG00000102802	MEDAG	0.021438	1.00E-06	14.3878827
ENSG00000176083	ZNF683	0.021393883	1.00E-06	14.38491075
ENSG00000215354	FAM90A24P	0.021393693	1.00E-06	14.38489793
ENSG00000244668	SNRPCP3	0.021385	1.00E-06	14.38431159
ENSG00000166869	CHP2	0.021367	1.00E-06	14.38309674
ENSG00000064218	DMRT3	0.021322432	1.00E-06	14.38008437
ENSG00000143768	LEFTY2	0.021278087	1.00E-06	14.37708086
ENSG00000250790	RP11-46H11.3	0.021214	1.00E-06	14.37272905
ENSG00000238125	SLC9A3P2	0.021154	1.00E-06	14.36864287
ENSG00000134812	GIF	0.021127296	1.00E-06	14.36682054
ENSG00000261741	FRG2KP	0.021118646	1.00E-06	14.3662297
ENSG00000120903	CHRNA2	0.021075143	1.00E-06	14.36325483
ENSG00000279985	CTC-436P18.4	0.021043	1.00E-06	14.36105278
ENSG00000248115	RP11-752D24.2	0.021031511	1.00E-06	14.36026489
ENSG00000251032	CUL1P1	0.020989	1.00E-06	14.35734581
ENSG00000259433	CTD-2651B20.4	0.020979	1.00E-06	14.35665829
ENSG00000181291	TMEM132E	0.020974625	1.00E-06	14.3563574
ENSG00000262209	PCDHGB3	0.020970359	1.00E-06	14.35606397
ENSG00000280159	CTD-2591A1.1	0.020937	1.00E-06	14.35376712
ENSG00000233642	GPR158-AS1	0.020919	1.00E-06	14.35252627
ENSG00000145888	GLRA1	0.0209108	1.00E-06	14.35196063

ENSG00000094963	FMO2	0.020843356	1.00E-06	14.3473
ENSG00000203857	HSD3B1	0.020833462	1.00E-06	14.34661499
ENSG00000262576	PCDHGA4	0.020829232	1.00E-06	14.34632203
ENSG00000269987	RP3-430N8.11	0.020817	1.00E-06	14.34547455
ENSG00000170837	GPR27	0.020799	1.00E-06	14.34422655
ENSG00000259246	HMGN2P47	0.020791	1.00E-06	14.34367153
ENSG00000143278	F13B	0.020773742	1.00E-06	14.34247352
ENSG00000228983	SLC47A1P1	0.020660176	1.00E-06	14.33456495
ENSG00000197646	PDCD1LG2	0.02066	1.00E-06	14.33455263
ENSG00000137752	CASP1	0.020644714	1.00E-06	14.33348482
ENSG00000270024	C8orf44-SGK3	0.020642007	1.00E-06	14.33329563
ENSG00000204480	PRAMEF19	0.020639	1.00E-06	14.33308545
ENSG00000269920	RP11-690P14.4	0.020631	1.00E-06	14.33252613
ENSG00000175699	LINC00521	0.020622614	1.00E-06	14.33193961
ENSG00000214695	NPAP1P2	0.020622	1.00E-06	14.33189664
ENSG00000216306	KRT19P2	0.020619886	1.00E-06	14.33174876
ENSG00000254443	RP11-304C12.3	0.020613887	1.00E-06	14.33132898
ENSG00000258393	RP11-907D1.2	0.020601	1.00E-06	14.33042675
ENSG00000145384	FABP2	0.02054	1.00E-06	14.32614856
ENSG00000212122	TSSK1B	0.02054	1.00E-06	14.32614856
ENSG00000242419	PCDHGC4	0.020465154	1.00E-06	14.32088193
ENSG00000276633	AJ011931.1	0.020424	1.00E-06	14.31797782
ENSG00000277475	AC213203.1	0.020415	1.00E-06	14.31734195
ENSG00000116748	AMPD1	0.020391126	1.00E-06	14.31565384
ENSG00000279149	RP11-374F3.5	0.020391	1.00E-06	14.31564491
ENSG00000229102	RP11-360P21.2	0.0203	1.00E-06	14.30919211
ENSG00000267153	CTBP2P3	0.020277	1.00E-06	14.3075566
ENSG00000215834	FMO9P	0.020269277	1.00E-06	14.30700702
ENSG00000256162	SMLR1	0.020261	1.00E-06	14.30641776
ENSG00000251557	HNRNPKP3	0.02025288	1.00E-06	14.30583947
ENSG00000278090	RP11-6O2.2	0.020229	1.00E-06	14.30413738
ENSG00000261375	RP11-632K20.6	0.020165	1.00E-06	14.29956579
ENSG00000249825	CTD-2201I18.1	0.020150702	1.00E-06	14.29854249
ENSG00000182077	PTCHD3	0.020125	1.00E-06	14.29670116
ENSG00000197506	SLC28A3	0.020104942	1.00E-06	14.29526258
ENSG00000261786	RP4-555D20.2	0.02009	1.00E-06	14.29418994
ENSG00000185640	KRT79	0.020021852	1.00E-06	14.28928781
ENSG00000204368	RP11-552E4.3	0.019989	1.00E-06	14.28691868
ENSG00000180210	F2	0.019959473	1.00E-06	14.28478603
ENSG00000278702	Metazoa_SRP	0.019959	1.00E-06	14.28475182
ENSG00000211699	TRGV3	0.019903	1.00E-06	14.28069829
ENSG00000174807	CD248	0.019897	1.00E-06	14.2802633
ENSG00000124575	HIST1H1D	0.01987	1.00E-06	14.27830425
ENSG00000056291	NPFFR2	0.019849684	1.00E-06	14.2768284
ENSG00000279341	RP11-720D4.2	0.019827	1.00E-06	14.27517878
ENSG00000270490	RP11-622C24.1	0.01982	1.00E-06	14.27466934
ENSG00000135333	EPHA7	0.019796693	1.00E-06	14.2729718
ENSG00000142698	C1orf94	0.019758224	1.00E-06	14.27016565

ENSG00000182901	RGS7	0.019742071	1.00E-06	14.26898569
ENSG00000142224	IL19	0.019673404	1.00E-06	14.26395898
ENSG00000229433	RP11-329B9.1	0.019644	1.00E-06	14.26180111
ENSG00000228963	OR7E93P	0.01964	1.00E-06	14.26150731
ENSG00000249726	TUBB1P1	0.019576	1.00E-06	14.25679839
ENSG00000186148	AC013271.3	0.019550334	1.00E-06	14.25490566
ENSG00000171044	XKR6	0.019541454	1.00E-06	14.25425023
ENSG00000237756	RP11-77M5.1	0.019534	1.00E-06	14.25369978
ENSG00000267072	RP11-165E7.1	0.019528982	1.00E-06	14.25332911
ENSG00000264242	RP11-271K11.1	0.019482	1.00E-06	14.24985417
ENSG00000109991	P2RX3	0.01946996	1.00E-06	14.24896232
ENSG00000143627	PKLR	0.019440928	1.00E-06	14.24680945
ENSG00000232497	ADIPOR1P1	0.01943	1.00E-06	14.24599828
ENSG00000205622	AF064858.6	0.019426018	1.00E-06	14.24570261
ENSG00000237243	AC022173.2	0.019406	1.00E-06	14.24421516
ENSG00000198488	B3GNT6	0.01938157	1.00E-06	14.2423978
ENSG00000176769	TCERG1L	0.019359339	1.00E-06	14.24074207
ENSG00000151790	TDO2	0.019338059	1.00E-06	14.23915535
ENSG00000174358	SLC6A19	0.019292665	1.00E-06	14.23576484
ENSG00000227066	RP3-340N1.2	0.019285561	1.00E-06	14.23523348
ENSG00000279096	AL356289.1	0.019278	1.00E-06	14.23466777
ENSG00000225931	RP3-395M20.7	0.019229	1.00E-06	14.23099612
ENSG00000207709	MIR197	0.019229	1.00E-06	14.23099612
ENSG00000137691	C11orf70	0.01922831	1.00E-06	14.23094436
ENSG00000132975	GPR12	0.019214166	1.00E-06	14.22988272
ENSG00000261617	RP11-243A14.1	0.019213	1.00E-06	14.22979518
ENSG00000204003	RP11-216L13.16	0.019198423	1.00E-06	14.22870021
ENSG00000173627	APOBEC4	0.019184282	1.00E-06	14.22763715
ENSG00000226017	PRICKLE2-AS3	0.019168	1.00E-06	14.22641219
ENSG00000256343	RP11-662M24.1	0.019134255	1.00E-06	14.22387007
ENSG00000260314	MRC1	0.018995	1.00E-06	14.21333209
ENSG00000254081	LINC01299	0.018936	1.00E-06	14.20884399
ENSG00000223486	AC092198.1	0.018913	1.00E-06	14.2070906
ENSG00000242550	SERPINB10	0.018850433	1.00E-06	14.20231007
ENSG00000171564	FGB	0.01879459	1.00E-06	14.19802983
ENSG00000237613	FAM138A	0.018788486	1.00E-06	14.19756123
ENSG00000248893	FAM138E	0.018788486	1.00E-06	14.19756123
ENSG00000185863	TMEM210	0.01876777	1.00E-06	14.19596962
ENSG00000259849	VENTXP1	0.018739	1.00E-06	14.19375635
ENSG00000253418	SNX18P27	0.01873	1.00E-06	14.19306328
ENSG00000109819	PPARGC1A	0.018729063	1.00E-06	14.1929911
ENSG00000214992	AKAP17BP	0.018719	1.00E-06	14.19221575
ENSG00000227555	MIR4290HG	0.018711	1.00E-06	14.19159904
ENSG00000261115	TMEM178B	0.018621859	1.00E-06	14.18470952
ENSG00000218839	FAM138C	0.018589968	1.00E-06	14.18223663
ENSG00000233288	RP11-760D2.5	0.018569	1.00E-06	14.1806085
ENSG00000206145	P2RX6P	0.018474534	1.00E-06	14.17325032
ENSG00000279490	CTD-2576D5.1	0.018474	1.00E-06	14.17320865

ENSG00000227502	LINC01268	0.018474	1.00E-06	14.17320865
ENSG00000167165	UGT1A6	0.018472379	1.00E-06	14.17308202
ENSG00000225239	RPL21P107	0.018441	1.00E-06	14.17062927
ENSG00000181631	P2RY13	0.018414	1.00E-06	14.16851543
ENSG00000180770	OR7E129P	0.018403	1.00E-06	14.16765335
ENSG00000234962	LINC00700	0.018394032	1.00E-06	14.16695015
ENSG00000253490	AC145110.1	0.018371246	1.00E-06	14.16516188
ENSG00000271382	RP11-1060G2.2	0.018354	1.00E-06	14.16380689
ENSG00000250733	C8orf17	0.018341	1.00E-06	14.16278468
ENSG00000258551	CRAT37	0.018338053	1.00E-06	14.16255284
ENSG00000233760	AC004947.2	0.018301	1.00E-06	14.15963486
ENSG00000154277	UCHL1	0.018288757	1.00E-06	14.15866942
ENSG00000273305	RP11-440D17.4	0.018286	1.00E-06	14.1584519
ENSG00000274052	OR7E156P	0.018282	1.00E-06	14.15813629
ENSG00000233701	PRR23C	0.018236	1.00E-06	14.15450169
ENSG00000237440	ZNF737	0.018216265	1.00E-06	14.15293957
ENSG00000276070	CCL4L2	0.018216236	1.00E-06	14.15293728
ENSG00000198155	ZNF876P	0.018151718	1.00E-06	14.1478185
ENSG00000173585	CCR9	0.018119546	1.00E-06	14.1452592
ENSG00000272506	RP4-535B20.4	0.018118	1.00E-06	14.14513609
ENSG00000130988	RGN	0.018106119	1.00E-06	14.14418971
ENSG00000250500	RP11-119H12.3	0.018069	1.00E-06	14.14122904
ENSG00000245750	DRAIC	0.0180548	1.00E-06	14.14009484
ENSG00000248364	OR7E86P	0.017994	1.00E-06	14.13522831
ENSG00000260102	LINC01070	0.017991	1.00E-06	14.13498776
ENSG00000183671	GPR1	0.01795862	1.00E-06	14.1323889
ENSG00000257947	RP11-46I1.1	0.017953	1.00E-06	14.13193732
ENSG00000268758	ADGRE4P	0.017940486	1.00E-06	14.13093139
ENSG00000282591	FAM138F	0.017823401	1.00E-06	14.12148501
ENSG00000236114	RP11-517P14.7	0.01764	1.00E-06	14.10656294
ENSG00000255261	OR7E4P	0.017585	1.00E-06	14.10205771
ENSG00000253485	PCDHGA5	0.017578466	1.00E-06	14.10152153
ENSG00000186235	AC016757.3	0.017514351	1.00E-06	14.09624991
ENSG00000143340	FAM163A	0.017496	1.00E-06	14.09473751
ENSG00000174004	NRROS	0.017490709	1.00E-06	14.09430117
ENSG00000237432	RPS7P12	0.01749	1.00E-06	14.09424267
ENSG00000232040	ZBED9	0.01747815	1.00E-06	14.09326485
ENSG00000116962	NID1	0.017359	1.00E-06	14.08339622
ENSG00000279905	RP11-117L5.1	0.017271	1.00E-06	14.076064
ENSG00000133063	CHIT1	0.017251033	1.00E-06	14.07439513
ENSG00000112041	TULP1	0.01720683	1.00E-06	14.07069374
ENSG00000206549	PRSS50	0.017200185	1.00E-06	14.07013648
ENSG00000281880	PAUPAR	0.017165	1.00E-06	14.06718224
ENSG00000254004	ZNF260	0.017102053	1.00E-06	14.06188188
ENSG00000255099	RP11-25D10.2	0.017091	1.00E-06	14.06094919
ENSG00000234148	RP11-395L14.3	0.01708	1.00E-06	14.06002035
ENSG00000272108	AC005754.8	0.01708	1.00E-06	14.06002035
ENSG00000112818	MEP1A	0.017079262	1.00E-06	14.05995802

ENSG00000138435	CHRNA1	0.017044589	1.00E-06	14.05702615
ENSG00000183347	GBP6	0.017039	1.00E-06	14.05655305
ENSG00000119614	VSX2	0.016993	1.00E-06	14.05265295
ENSG00000105989	WNT2	0.016937024	1.00E-06	14.04789275
ENSG00000166509	CLEC3A	0.016926216	1.00E-06	14.0469719
ENSG00000249747	RP11-308K2.1	0.016811161	1.00E-06	14.03713174
ENSG00000204001	LCN8	0.016753168	1.00E-06	14.03214633
ENSG00000163352	LENEP	0.01673363	1.00E-06	14.03046283
ENSG00000259129	LINC00648	0.016725984	1.00E-06	14.02980349
ENSG00000204571	KRTAP5-11	0.016709406	1.00E-06	14.02837281
ENSG00000278073	MIR6726	0.016687	1.00E-06	14.02643699
ENSG00000206181	TCEB3B	0.016687	1.00E-06	14.02643699
ENSG00000090402	SI	0.016583622	1.00E-06	14.01747155
ENSG00000253379	RP11-1102P16.1	0.016579316	1.00E-06	14.01709684
ENSG00000162747	FCGR3B	0.016562284	1.00E-06	14.01561406
ENSG00000254254	RP11-17A4.2	0.016535494	1.00E-06	14.01327854
ENSG00000175746	C15orf54	0.016535	1.00E-06	14.01323543
ENSG00000282807	AC008993.3	0.016535	1.00E-06	14.01323543
ENSG00000144057	ST6GAL2	0.016507492	1.00E-06	14.01083335
ENSG00000006116	CACNG3	0.016482	1.00E-06	14.0086037
ENSG00000156103	MMP16	0.016468361	1.00E-06	14.00740938
ENSG00000112246	SIM1	0.016464588	1.00E-06	14.0070788
ENSG00000164002	EXO5	0.016460514	1.00E-06	14.00672176
ENSG00000222035	AC079354.3	0.016456	1.00E-06	14.00632608
ENSG00000197376	OR8S1	0.016433994	1.00E-06	14.00439554
ENSG00000268565	AC005339.2	0.016397	1.00E-06	14.00114426
ENSG00000112164	GLP1R	0.016392	1.00E-06	14.00070427
ENSG00000232117	LINC00384	0.016342	1.00E-06	13.99629694
ENSG00000117215	PLA2G2D	0.01632601	1.00E-06	13.9948846
ENSG00000127920	GNG11	0.016326	1.00E-06	13.99488374
ENSG00000241890	RPL13P4	0.016301	1.00E-06	13.99267285
ENSG00000214338	SOGA3	0.016280663	1.00E-06	13.99087182
ENSG00000280414	RP11-572N21.1	0.016266	1.00E-06	13.9895719
ENSG00000135902	CHRND	0.016250487	1.00E-06	13.9881953
ENSG00000257431	RP11-263K4.3	0.016203	1.00E-06	13.98397333
ENSG00000261758	RP11-102M11.2	0.016152	1.00E-06	13.9794252
ENSG00000233619	AC006328.9	0.016115	1.00E-06	13.97611657
ENSG00000283039	KLF18	0.016112	1.00E-06	13.97584797
ENSG00000224367	OACYLP	0.016075997	1.00E-06	13.97262064
ENSG00000205695	RP11-15H7.2	0.016046	1.00E-06	13.96992608
ENSG00000180730	SHISA2	0.016016	1.00E-06	13.96722626
ENSG00000174899	PQLC2L	0.016005134	1.00E-06	13.96624713
ENSG00000234568	BIN2P1	0.015967	1.00E-06	13.96280565
ENSG00000280380	RP11-326K13.3	0.01596	1.00E-06	13.96217303
ENSG00000215174	NLRP2P	0.015935	1.00E-06	13.9599114
ENSG00000248118	LINC01019	0.01591	1.00E-06	13.95764622
ENSG00000221686	AC244205.1	0.015905	1.00E-06	13.95719275
ENSG00000232111	RP11-126O22.1	0.015905	1.00E-06	13.95719275

ENSG00000230773	AC079807.4	0.015899276	1.00E-06	13.95667348
ENSG00000161911	TREML1	0.015786625	1.00E-06	13.94641516
ENSG00000166862	CACNG2	0.015786605	1.00E-06	13.94641337
ENSG00000168334	XIRP1	0.015739944	1.00E-06	13.94214282
ENSG00000153012	LGI2	0.015725	1.00E-06	13.9407724
ENSG00000234715	CTB-107G13.1	0.015689	1.00E-06	13.93746578
ENSG00000204442	FAM155A	0.015682	1.00E-06	13.93682194
ENSG00000219240	RP11-63K6.5	0.015677	1.00E-06	13.93636189
ENSG00000277795	AC005752.10	0.015661	1.00E-06	13.93488872
ENSG00000186471	AKAP14	0.015660076	1.00E-06	13.9348036
ENSG00000259450	RP11-265N7.1	0.015572	1.00E-06	13.92666663
ENSG00000228824	MIR4500HG	0.015550394	1.00E-06	13.92466353
ENSG00000075035	WSCD2	0.015524252	1.00E-06	13.92223613
ENSG00000111432	FZD10	0.015508	1.00E-06	13.92072502
ENSG00000204661	C5orf60	0.015497741	1.00E-06	13.91977028
ENSG00000227640	SOX21-AS1	0.015484	1.00E-06	13.91849059
ENSG00000087258	GNAO1	0.015456173	1.00E-06	13.91589552
ENSG00000015592	STMN4	0.015451293	1.00E-06	13.91543998
ENSG00000158014	SLC30A2	0.015449585	1.00E-06	13.91528051
ENSG00000131482	G6PC	0.015441488	1.00E-06	13.91452415
ENSG00000239510	RPL9P5	0.015437	1.00E-06	13.91410479
ENSG00000104044	OCA2	0.015427811	1.00E-06	13.9132458
ENSG00000275226	LINC00547	0.015358	1.00E-06	13.90670273
ENSG00000228303	RP11-10F11.2	0.015355	1.00E-06	13.90642089
ENSG00000279444	RP11-715H19.2	0.015284	1.00E-06	13.89973454
ENSG00000255622	PCDHB17P	0.015272	1.00E-06	13.89860139
ENSG00000123407	HOXC12	0.015262	1.00E-06	13.89765641
ENSG00000274391	TPTE	0.015255595	1.00E-06	13.89705084
ENSG00000146477	SLC22A3	0.015202	1.00E-06	13.89197352
ENSG00000280294	RP11-177H2.1	0.015183	1.00E-06	13.89016926
ENSG00000081853	PCDHGA2	0.015139054	1.00E-06	13.88598746
ENSG00000138944	KIAA1644	0.015079	1.00E-06	13.88025314
ENSG00000204397	CARD16	0.015075748	1.00E-06	13.87994199
ENSG00000075461	CACNG4	0.015058	1.00E-06	13.87824254
ENSG00000189275	LINC01164	0.01504	1.00E-06	13.87651695
ENSG00000177306	OR7E125P	0.01504	1.00E-06	13.87651695
ENSG00000188386	PPP3R2	0.015027	1.00E-06	13.8752694
ENSG00000142025	DMRTC2	0.015018012	1.00E-06	13.87440627
ENSG00000253305	PCDHGB6	0.015009219	1.00E-06	13.8735613
ENSG00000236963	LINC01141	0.014995865	1.00E-06	13.8722771
ENSG00000219159	AC011298.2	0.014979	1.00E-06	13.87065369
ENSG00000244753	RPL15P21	0.01496	1.00E-06	13.86882255
ENSG00000228843	RP11-112J3.15	0.014926	1.00E-06	13.86553997
ENSG00000226367	ST7-AS2	0.014917353	1.00E-06	13.86470392
ENSG00000137959	IFI44L	0.014911011	1.00E-06	13.86409049
ENSG00000170374	SP7	0.014907719	1.00E-06	13.86377189
ENSG00000271231	KRT18P9	0.014877	1.00E-06	13.86079601
ENSG00000224341	RP4-562J12.1	0.014861	1.00E-06	13.85924358

ENSG00000115165	CYTIP	0.014847567	1.00E-06	13.85793893
ENSG00000251342	CTC-235G5.2	0.014833	1.00E-06	13.85652279
ENSG00000198535	C2CD4A	0.014774	1.00E-06	13.85077286
ENSG00000280305	CTD-2351A16.1	0.01477	1.00E-06	13.85038221
ENSG00000219438	FAM19A5	0.01475717	1.00E-06	13.84912845
ENSG00000278955	CH507-210P18.4	0.014726	1.00E-06	13.84607799
ENSG00000142973	CYP4B1	0.014710094	1.00E-06	13.84451881
ENSG00000272235	RP11-22L13.1	0.014706	1.00E-06	13.84411727
ENSG00000007933	FMO3	0.014697273	1.00E-06	13.84326086
ENSG00000227769	AC072062.3	0.014682901	1.00E-06	13.84184946
ENSG00000260855	RP11-439E19.10	0.014659	1.00E-06	13.83949907
ENSG00000179136	LINC00670	0.014626443	1.00E-06	13.83629132
ENSG00000217770	FEM1AP3	0.014617	1.00E-06	13.83535962
ENSG00000112238	PRDM13	0.014604181	1.00E-06	13.83409381
ENSG00000184945	AQP12A	0.014600201	1.00E-06	13.83370059
ENSG00000186019	AC084219.4	0.014591769	1.00E-06	13.83286717
ENSG00000238154	USP9YP4	0.014584	1.00E-06	13.83209885
ENSG00000275166	MIR6814	0.014542	1.00E-06	13.82793808
ENSG00000155961	RAB39B	0.014521	1.00E-06	13.82585319
ENSG00000222961	AC008949.1	0.014489	1.00E-06	13.82267041
ENSG00000224559	LINC01087	0.014476	1.00E-06	13.82137539
ENSG00000177103	DSCAML1	0.014469758	1.00E-06	13.82075314
ENSG00000232416	BPESC1	0.01445	1.00E-06	13.81878187
ENSG00000260947	RP11-384P7.7	0.014426	1.00E-06	13.81638371
ENSG00000128652	HOXD3	0.014344789	1.00E-06	13.80823909
ENSG00000240505	TNFRSF13B	0.014336878	1.00E-06	13.80744331
ENSG00000227811	FAM212B-AS1	0.01433	1.00E-06	13.80675099
ENSG00000260779	RP11-856M7.1	0.014272	1.00E-06	13.8008999
ENSG00000236389	RP1-287H17.1	0.014245	1.00E-06	13.798168
ENSG00000118971	CCND2	0.014223043	1.00E-06	13.79594256
ENSG00000231009	CUBNP1	0.014216	1.00E-06	13.79522797
ENSG00000266687	AL356261.1	0.014203	1.00E-06	13.79390807
ENSG00000278727	AC000403.4	0.014197	1.00E-06	13.79329848
ENSG00000115353	TACR1	0.014190326	1.00E-06	13.79262012
ENSG00000205126	ACCSL	0.014175919	1.00E-06	13.7911546
ENSG00000124564	SLC17A3	0.01416167	1.00E-06	13.78970378
ENSG00000113600	C9	0.014149346	1.00E-06	13.78844775
ENSG00000164007	CLDN19	0.01413049	1.00E-06	13.78652385
ENSG00000104055	TGM5	0.014110305	1.00E-06	13.78446155
ENSG00000227508	LINC01624	0.014094384	1.00E-06	13.78283279
ENSG00000106013	ANKRD7	0.014049368	1.00E-06	13.77821759
ENSG00000224674	EIF3EP2	0.014029	1.00E-06	13.77612456
ENSG00000166770	ZNF667-AS1	0.014024718	1.00E-06	13.77568415
ENSG00000243349	RP11-445N18.7	0.013994	1.00E-06	13.77252078
ENSG00000177398	UMODL1	0.013986296	1.00E-06	13.77172635
ENSG00000174171	RP11-23P13.6	0.013932384	1.00E-06	13.76615456
ENSG00000148702	HABP2	0.013917756	1.00E-06	13.76463904
ENSG00000231898	AC012594.1	0.013875775	1.00E-06	13.76028072

ENSG00000253408	RP11-231D20.2	0.013865176	1.00E-06	13.7591783
ENSG00000257060	RP11-266O8.1	0.01384158	1.00E-06	13.75672097
ENSG00000234795	RFTN1P1	0.013815309	1.00E-06	13.75398026
ENSG00000225128	LINC00972	0.013772069	1.00E-06	13.74945768
ENSG00000229766	RP5-971N18.3	0.013756108	1.00E-06	13.74778469
ENSG00000232628	RP11-365O16.3	0.013756019	1.00E-06	13.74777536
ENSG00000203335	AC006019.3	0.013737	1.00E-06	13.74577935
ENSG00000162493	PDPN	0.013729778	1.00E-06	13.7450207
ENSG00000101958	GLRA2	0.013707626	1.00E-06	13.74269113
ENSG00000170549	IRX1	0.013696	1.00E-06	13.74146699
ENSG00000138075	ABCG5	0.013681586	1.00E-06	13.73994786
ENSG00000186094	AGBL4	0.013641427	1.00E-06	13.73570693
ENSG00000254226	CTB-12O2.1	0.013591144	1.00E-06	13.73037927
ENSG00000279942	RP1-153P14.7	0.01359	1.00E-06	13.73025784
ENSG00000273249	LL09NC01-251B2.3	0.013586224	1.00E-06	13.72985691
ENSG00000239516	FLYWCH1P1	0.013579	1.00E-06	13.72908962
ENSG00000183837	PNMA3	0.013578993	1.00E-06	13.72908882
ENSG00000151490	PTPRO	0.013537678	1.00E-06	13.7246927
ENSG00000185823	NPAP1	0.013525	1.00E-06	13.72334097
ENSG00000114487	MORC1	0.013522	1.00E-06	13.72302093
ENSG00000067842	ATP2B3	0.013510846	1.00E-06	13.72183043
ENSG00000260093	RP11-1E4.1	0.013501	1.00E-06	13.72077865
ENSG00000205221	VIT	0.013444827	1.00E-06	13.71476362
ENSG00000204544	MUC21	0.013418123	1.00E-06	13.71189524
ENSG00000242111	TOPORSLP1	0.013414793	1.00E-06	13.71153721
ENSG00000099958	DERL3	0.013407258	1.00E-06	13.71072655
ENSG00000245768	RP11-410D17.2	0.013368702	1.00E-06	13.70657182
ENSG00000152213	ARL11	0.013302499	1.00E-06	13.69940964
ENSG00000255554	OR7E1P	0.013282	1.00E-06	13.69718478
ENSG00000104415	WISP1	0.01324	1.00E-06	13.6926155
ENSG00000197863	ZNF790	0.013216589	1.00E-06	13.69006223
ENSG00000225217	HSPA7	0.013207	1.00E-06	13.68901517
ENSG00000129744	ART1	0.013183731	1.00E-06	13.68647106
ENSG00000184730	APOBR	0.013154	1.00E-06	13.68321395
ENSG00000120328	PCDHB12	0.013135001	1.00E-06	13.68112872
ENSG00000233427	RP1-212P9.3	0.013134	1.00E-06	13.68101874
ENSG00000185306	C12orf56	0.013130907	1.00E-06	13.68067899
ENSG00000260337	RP11-386M24.6	0.013087147	1.00E-06	13.67586301
ENSG00000233821	ENOX1-AS1	0.013027	1.00E-06	13.66921726
ENSG00000282137	OR4G3P	0.013007	1.00E-06	13.66700063
ENSG00000278631	RP11-71B7.1	0.012991	1.00E-06	13.66522487
ENSG00000089558	KCNH4	0.012970765	1.00E-06	13.6629759
ENSG00000230563	RP5-828H9.1	0.012957635	1.00E-06	13.66151485
ENSG00000265052	RN7SL622P	0.012955	1.00E-06	13.6612214
ENSG00000257488	RP5-1057I20.2	0.012939238	1.00E-06	13.65946508
ENSG00000231918	AC007682.1	0.012922	1.00E-06	13.65754176
ENSG00000164871	SPAG11B	0.012898043	1.00E-06	13.65486452
ENSG00000178287	SPAG11A	0.012898043	1.00E-06	13.65486452

ENSG00000254307	KB-1608C10.2	0.012885894	1.00E-06	13.65350498
ENSG00000187950	OVCH1	0.01285865	1.00E-06	13.65045152
ENSG00000255693	RP11-766N7.3	0.012820085	1.00E-06	13.64611822
ENSG00000173714	WFIKKN2	0.012816867	1.00E-06	13.64575606
ENSG00000178175	ZNF366	0.012804244	1.00E-06	13.64433446
ENSG00000167194	C16orf92	0.012781033	1.00E-06	13.64171684
ENSG00000129221	AIPL1	0.012771605	1.00E-06	13.64065216
ENSG00000185013	NT5C1B	0.012765785	1.00E-06	13.63999461
ENSG00000167390	POM121L3P	0.012764	1.00E-06	13.63979289
ENSG00000197322	C17orf102	0.012733	1.00E-06	13.63628475
ENSG00000164729	SLC35G3	0.012699	1.00E-06	13.63242727
ENSG00000253105	KB-1448A5.1	0.012651178	1.00E-06	13.62698409
ENSG00000204241	RP11-713P17.3	0.012622675	1.00E-06	13.62373007
ENSG00000268505	RP11-805I24.3	0.012601	1.00E-06	13.62125061
ENSG00000178722	C5orf64	0.012588892	1.00E-06	13.61986368
ENSG00000221792	MIR1282	0.012575	1.00E-06	13.61827078
ENSG00000188984	AADACL3	0.012570171	1.00E-06	13.6177167
ENSG00000143473	KCNH1	0.012558086	1.00E-06	13.61632902
ENSG00000164122	ASB5	0.012523758	1.00E-06	13.6123799
ENSG00000275302	CCL4	0.012523637	1.00E-06	13.61236596
ENSG00000269939	PCF11-AS1	0.012515	1.00E-06	13.61137067
ENSG00000228123	RP11-15B24.3	0.012511	1.00E-06	13.61090949
ENSG00000197410	DCHS2	0.012504416	1.00E-06	13.6101501
ENSG00000113520	IL4	0.01244407	1.00E-06	13.60317076
ENSG00000165443	PHYHIPL	0.012434271	1.00E-06	13.60203433
ENSG00000145721	LIX1	0.012368576	1.00E-06	13.59439184
ENSG00000226440	RP11-506B6.6	0.012317648	1.00E-06	13.58843914
ENSG00000241449	RP11-545G3.1	0.012293757	1.00E-06	13.5856382
ENSG00000236841	AC007750.5	0.012243724	1.00E-06	13.57975479
ENSG00000269834	ZNF528-AS1	0.012234894	1.00E-06	13.57871401
ENSG00000152954	NRSN1	0.012161407	1.00E-06	13.57002252
ENSG00000232633	CTD-2201G3.1	0.012136143	1.00E-06	13.56702238
ENSG00000140955	ADAD2	0.012132922	1.00E-06	13.56663941
ENSG00000279804	PRAMEF18	0.012095	1.00E-06	13.56212315
ENSG00000254447	OR7E11P	0.012029	1.00E-06	13.55422909
ENSG00000154646	TMPRSS15	0.012023757	1.00E-06	13.55360018
ENSG00000177990	DPY19L2	0.01201958	1.00E-06	13.55309889
ENSG00000279910	RP11-113E21.2	0.012016	1.00E-06	13.5526691
ENSG00000106536	POU6F2	0.011995408	1.00E-06	13.55019456
ENSG00000196267	ZNF836	0.011992163	1.00E-06	13.54980428
ENSG00000242109	NPM1P23	0.01199	1.00E-06	13.54954404
ENSG00000232913	PLCE1-AS2	0.011981331	1.00E-06	13.54850051
ENSG00000234571	RP5-998N21.4	0.011981	1.00E-06	13.54846071
ENSG00000203395	AC015969.3	0.011981	1.00E-06	13.54846071
ENSG00000233030	RP11-196G18.3	0.011971	1.00E-06	13.54725605
ENSG00000249125	RP11-381N20.2	0.011961	1.00E-06	13.54605039
ENSG00000256870	SLC5A8	0.011938	1.00E-06	13.54327354
ENSG00000169618	PROKR1	0.011911	1.00E-06	13.54000692

ENSG00000282419	TEX13D	0.011857905	1.00E-06	13.53356149
ENSG00000233424	RP11-175I6.5	0.01184	1.00E-06	13.53138146
ENSG00000253171	CTB-118P15.2	0.0118	1.00E-06	13.52649924
ENSG00000277586	NEFL	0.011757556	1.00E-06	13.52130053
ENSG00000234754	C1orf140	0.011728	1.00E-06	13.51766939
ENSG00000230400	RP11-359G22.2	0.011711035	1.00E-06	13.51558102
ENSG00000261411	RP11-268G13.1	0.011674	1.00E-06	13.51101135
ENSG00000226237	GAS1RR	0.011601	1.00E-06	13.50196155
ENSG00000145428	RNF175	0.011593741	1.00E-06	13.50105857
ENSG00000011083	SLC6A7	0.011591666	1.00E-06	13.50080036
ENSG00000149599	DUSP15	0.011546112	1.00E-06	13.49511955
ENSG00000272299	RP11-394A14.4	0.01151	1.00E-06	13.49060021
ENSG00000267634	RPL7L1P5	0.011494	1.00E-06	13.48859333
ENSG00000066813	ACSM2B	0.011479384	1.00E-06	13.48675763
ENSG00000197629	MPEG1	0.011458	1.00E-06	13.48406762
ENSG00000019991	HGF	0.011440828	1.00E-06	13.48190381
ENSG00000154975	CA10	0.011437402	1.00E-06	13.48147179
ENSG00000171540	OTP	0.011434816	1.00E-06	13.48114559
ENSG00000235617	XXbac-B476C20.10	0.011392	1.00E-06	13.47573343
ENSG00000267881	AC011513.3	0.011373522	1.00E-06	13.4733915
ENSG00000167555	ZNF528	0.01133695	1.00E-06	13.4687449
ENSG00000269793	ZIM2-AS1	0.011299368	1.00E-06	13.46395446
ENSG00000232006	AC005537.2	0.011260236	1.00E-06	13.4589494
ENSG00000267391	RP11-1151B14.3	0.011258	1.00E-06	13.45866293
ENSG00000086548	CEACAM6	0.011228178	1.00E-06	13.45483625
ENSG00000188877	POTEA	0.011221	1.00E-06	13.45391363
ENSG00000152192	POU4F1	0.011193	1.00E-06	13.45030915
ENSG00000101938	CHRDL1	0.011166429	1.00E-06	13.44688021
ENSG00000115935	WIPF1	0.011160476	1.00E-06	13.44611097
ENSG00000236491	RP13-565O16.2	0.011155	1.00E-06	13.44540289
ENSG00000273499	SNORA40	0.011097	1.00E-06	13.43788209
ENSG00000171804	WDR87	0.011065626	1.00E-06	13.43379741
ENSG00000221028	MIR1231	0.011065	1.00E-06	13.43371583
ENSG00000115593	SMYD1	0.01106419	1.00E-06	13.43361019
ENSG00000235931	LINC01553	0.011059551	1.00E-06	13.43300518
ENSG00000187889	C1orf168	0.010985695	1.00E-06	13.42333852
ENSG00000248587	GDNF-AS1	0.010978416	1.00E-06	13.42238228
ENSG00000186369	LINC00643	0.010976218	1.00E-06	13.42209348
ENSG00000259043	BRD7P1	0.010897	1.00E-06	13.41164339
ENSG00000254786	RP11-142C4.5	0.010887	1.00E-06	13.41031884
ENSG00000250754	RP11-386B13.3	0.010879807	1.00E-06	13.40936532
ENSG00000105122	RASAL3	0.010872755	1.00E-06	13.40842992
ENSG00000257109	OR4F28P	0.010852	1.00E-06	13.40567333
ENSG00000175065	DSG4	0.01083792	1.00E-06	13.40380022
ENSG00000116783	TNNI3K	0.010785308	1.00E-06	13.3967798
ENSG00000179299	NSUN7	0.010764715	1.00E-06	13.39402255
ENSG00000278060	RP11-466M21.1	0.01076	1.00E-06	13.39339046
ENSG00000173110	HSPA6	0.010733	1.00E-06	13.38976576

ENSG00000186160	CYP4Z1	0.010709314	1.00E-06	13.3865784
ENSG00000246100	LINC00900	0.010679052	1.00E-06	13.38249597
ENSG00000169594	BNC1	0.010609631	1.00E-06	13.37308686
ENSG00000241636	LINC01323	0.010604	1.00E-06	13.37232095
ENSG00000120156	TEK	0.010591867	1.00E-06	13.37066926
ENSG00000205045	SLFN12L	0.010579057	1.00E-06	13.36892338
ENSG00000223611	SUPT20HL2	0.010564	1.00E-06	13.36686859
ENSG00000205002	AARD	0.010550427	1.00E-06	13.36501383
ENSG00000276547	PCDHGB5	0.010492313	1.00E-06	13.35704512
ENSG00000270433	RP11-124D2.7	0.010483436	1.00E-06	13.35582403
ENSG00000139155	SLCO1C1	0.010472351	1.00E-06	13.35429771
ENSG00000259070	LINC00639	0.010472165	1.00E-06	13.35427206
ENSG00000256452	RP11-667M19.10	0.010469	1.00E-06	13.35383602
ENSG00000272568	CTB-113D17.1	0.010353667	1.00E-06	13.33785415
ENSG00000164440	TXLNB	0.01030988	1.00E-06	13.3317399
ENSG00000181234	TMEM132C	0.010288	1.00E-06	13.32867493
ENSG00000230977	AC023274.6	0.010265	1.00E-06	13.32544601
ENSG00000197496	SLC2A10	0.010178969	1.00E-06	13.31330379
ENSG00000090376	IRAK3	0.010174955	1.00E-06	13.3127348
ENSG00000235535	RP11-532N4.2	0.010126361	1.00E-06	13.30582814
ENSG00000231290	APCDD1L-AS1	0.010096751	1.00E-06	13.30160351
ENSG00000186354	C9orf47	0.010078201	1.00E-06	13.29895048
ENSG00000226741	CTA-929C8.6	0.010074	1.00E-06	13.29834902
ENSG00000117400	MPL	0.010045066	1.00E-06	13.29419939
ENSG00000134207	SYT6	0.009972202	1.00E-06	13.28369642
ENSG00000279200	RP11-524F11.3	0.009962	1.00E-06	13.2822197
ENSG00000248599	FLJ42969	0.009929	1.00E-06	13.27743271
ENSG00000134183	GNAT2	0.009923726	1.00E-06	13.27666612
ENSG00000238152	OR7E140P	0.009922	1.00E-06	13.27641524
ENSG00000274214	RP11-757O6.6	0.00988	1.00E-06	13.27029533
ENSG00000233725	LINC00284	0.009863357	1.00E-06	13.26786298
ENSG00000187553	CYP26C1	0.00979681	1.00E-06	13.25809638
ENSG00000235414	RPSAP24	0.009717	1.00E-06	13.24629525
ENSG00000234381	MED15P7	0.009659	1.00E-06	13.23765812
ENSG00000275771	AC140725.8	0.00964	1.00E-06	13.23481743
ENSG00000278128	RP11-440J4.2	0.009637	1.00E-06	13.23436839
ENSG00000243144	RP11-115N4.1	0.009636077	1.00E-06	13.23423027
ENSG00000112280	COL9A1	0.009613408	1.00E-06	13.23083218
ENSG00000173769	TOPAZ1	0.009542	1.00E-06	13.22007597
ENSG00000133619	KRBA1	0.009536672	1.00E-06	13.21927023
ENSG00000229060	RP11-20P5.2	0.009525	1.00E-06	13.21750338
ENSG00000156486	KCNS2	0.00951677	1.00E-06	13.21625635
ENSG00000239998	LILRA2	0.009514608	1.00E-06	13.21592853
ENSG00000154493	C10orf90	0.009491992	1.00E-06	13.21249511
ENSG00000152207	CYSLTR2	0.009474454	1.00E-06	13.20982712
ENSG00000259345	RP11-624L4.1	0.00941945	1.00E-06	13.20142716
ENSG00000230894	RP11-67K19.3	0.009372	1.00E-06	13.19414124
ENSG00000227906	SNAP25-AS1	0.009355715	1.00E-06	13.19163221

ENSG00000253537	PCDHGA7	0.009350907	1.00E-06	13.19089057
ENSG00000249359	RP11-374A4.1	0.009332	1.00E-06	13.18797059
ENSG00000185974	GRK1	0.009270189	1.00E-06	13.17838311
ENSG00000187997	C17orf99	0.009209678	1.00E-06	13.16893498
ENSG00000050730	TNIP3	0.009185032	1.00E-06	13.16506901
ENSG00000162543	UBXN10	0.009136	1.00E-06	13.15734694
ENSG00000258368	ZNF970P	0.00913	1.00E-06	13.15639914
ENSG00000186487	MYT1L	0.00910454	1.00E-06	13.15237042
ENSG00000241111	PRICKLE2-AS1	0.009092	1.00E-06	13.15038197
ENSG00000180926	OR7E18P	0.009063	1.00E-06	13.14577297
ENSG00000102468	HTR2A	0.009049942	1.00E-06	13.14369286
ENSG00000259876	CTD-3037G24.4	0.00904	1.00E-06	13.14210706
ENSG00000107186	MPDZ	0.009029664	1.00E-06	13.14045661
ENSG00000257398	RP11-554D14.6	0.009022146	1.00E-06	13.13925486
ENSG00000188868	ZNF563	0.008939134	1.00E-06	13.12591933
ENSG00000197893	NRAP	0.008892516	1.00E-06	13.1183759
ENSG00000237136	C4orf51	0.008843059	1.00E-06	13.11032978
ENSG00000224228	RP1-15D23.2	0.008818	1.00E-06	13.10623576
ENSG00000110077	MS4A6A	0.008788716	1.00E-06	13.10143674
ENSG00000267097	SLC14A2-AS1	0.008781196	1.00E-06	13.10020178
ENSG00000188089	PLA2G4E	0.008740493	1.00E-06	13.09349892
ENSG00000187268	FAM9C	0.008688	1.00E-06	13.08480839
ENSG00000163749	CCDC158	0.008685561	1.00E-06	13.08440325
ENSG00000171815	PCDHB1	0.008679	1.00E-06	13.08331311
ENSG00000273295	AP000350.5	0.008668	1.00E-06	13.08148344
ENSG00000187068	C3orf70	0.008625	1.00E-06	13.07430874
ENSG00000250208	FZD10-AS1	0.008587545	1.00E-06	13.06803002
ENSG00000236977	ANKRD44-IT1	0.00857	1.00E-06	13.06507949
ENSG00000196277	GRM7	0.008551075	1.00E-06	13.06189006
ENSG00000213316	LTC4S	0.008539414	1.00E-06	13.05992143
ENSG00000132854	KANK4	0.008510895	1.00E-06	13.05509508
ENSG00000261272	MUC22	0.008456	1.00E-06	13.04575966
ENSG00000274342	TTC28-AS1_3	0.008436	1.00E-06	13.04234338
ENSG00000229292	RFPL4AL1	0.008408	1.00E-06	13.03754695
ENSG00000267098	RP11-325K19.1	0.008392	1.00E-06	13.03479896
ENSG00000221836	OR2A5	0.008373	1.00E-06	13.03152891
ENSG00000087495	PHACTR3	0.008350441	1.00E-06	13.02763659
ENSG00000184012	TMPRSS2	0.008320608	1.00E-06	13.02247326
ENSG00000237642	HMGB3P5	0.008316	1.00E-06	13.02167404
ENSG00000166183	ASPG	0.008309664	1.00E-06	13.02057443
ENSG00000226516	FAM138B	0.008262	1.00E-06	13.01227534
ENSG00000112499	SLC22A2	0.008247924	1.00E-06	13.00981535
ENSG00000245526	LINC00461	0.008243795	1.00E-06	13.0090929
ENSG00000257162	MED15P6	0.008242397	1.00E-06	13.0088482
ENSG00000164330	EBF1	0.00823871	1.00E-06	13.00820273
ENSG00000277321	MIR6820	0.008229	1.00E-06	13.00650141
ENSG00000231532	LINC01249	0.008211697	1.00E-06	13.00346468
ENSG00000187527	ATP13A5	0.008172327	1.00E-06	12.99653123

ENSG00000235531	MSC-AS1	0.008098854	1.00E-06	12.98350211
ENSG00000080224	EPHA6	0.008090337	1.00E-06	12.981984
ENSG00000018625	ATP1A2	0.008080716	1.00E-06	12.98026738
ENSG00000273758	MIR6790	0.008079	1.00E-06	12.97996102
ENSG00000215512	AP005901.1	0.008054261	1.00E-06	12.9755365
ENSG00000263278	CH17-351M24.1	0.007990004	1.00E-06	12.96398043
ENSG00000215288	RP11-613H2.1	0.007977	1.00E-06	12.96163056
ENSG00000110680	CALCA	0.007972385	1.00E-06	12.96079573
ENSG00000198358	RP11-544M22.1	0.007943	1.00E-06	12.95546829
ENSG00000260409	RP11-403B2.7	0.007943	1.00E-06	12.95546829
ENSG00000101292	PROKR2	0.007932	1.00E-06	12.95346896
ENSG00000009694	TENM1	0.007892939	1.00E-06	12.94634681
ENSG00000245384	AC004053.1	0.007888	1.00E-06	12.94544384
ENSG00000249267	LINC00939	0.007865406	1.00E-06	12.9413056
ENSG00000278517	MIR6865	0.00783	1.00E-06	12.93479659
ENSG00000103546	SLC6A2	0.007825671	1.00E-06	12.93399872
ENSG00000277784	MIR6786	0.007794	1.00E-06	12.92814822
ENSG00000178171	AMER3	0.007778437	1.00E-06	12.92526454
ENSG00000187754	SSX7	0.007765	1.00E-06	12.92277021
ENSG00000261231	RP11-523L20.2	0.007755355	1.00E-06	12.92097706
ENSG00000268447	SSX2B	0.007738129	1.00E-06	12.91776916
ENSG00000115850	LCT	0.007728147	1.00E-06	12.91590676
ENSG00000241884	RP11-85I21.1	0.007719689	1.00E-06	12.91432703
ENSG00000185176	AQP12B	0.00770445	1.00E-06	12.91147624
ENSG00000263753	LINC00667	0.007651141	1.00E-06	12.90145917
ENSG00000189184	PCDH18	0.00761261	1.00E-06	12.89417538
ENSG00000113262	GRM6	0.007601905	1.00E-06	12.89214531
ENSG00000276124	MIR6855	0.007597	1.00E-06	12.89121411
ENSG00000170516	COX7B2	0.007568007	1.00E-06	12.88569779
ENSG00000258053	CTD-2021H9.3	0.007567	1.00E-06	12.88550573
ENSG00000254325	RP11-318K15.2	0.007562	1.00E-06	12.88455213
ENSG00000128815	WDFY4	0.007436566	1.00E-06	12.86042087
ENSG00000183090	FREM3	0.007409835	1.00E-06	12.85522568
ENSG00000223638	RFPL4A	0.007376	1.00E-06	12.84862294
ENSG00000162374	ELAVL4	0.007309447	1.00E-06	12.83554651
ENSG00000232517	AC112198.1	0.007275	1.00E-06	12.82873153
ENSG00000177599	ZNF491	0.007218221	1.00E-06	12.81742766
ENSG00000154734	ADAMTS1	0.007206256	1.00E-06	12.81503413
ENSG00000254506	RP11-748H22.1	0.007155215	1.00E-06	12.80477942
ENSG00000137727	ARHGAP20	0.007143626	1.00E-06	12.80244086
ENSG00000278034	MIR6731	0.007068	1.00E-06	12.78708632
ENSG00000274805	MIR6768	0.007068	1.00E-06	12.78708632
ENSG00000111218	PRMT8	0.007047902	1.00E-06	12.78297818
ENSG00000275572	GRIFIN	0.00702039	1.00E-06	12.77733539
ENSG00000164304	CAGE1	0.007004682	1.00E-06	12.77410389
ENSG00000134215	VAV3	0.007000138	1.00E-06	12.77316755
ENSG00000179270	C2orf71	0.006997511	1.00E-06	12.77262621
ENSG00000261623	RP11-189E14.4	0.006928	1.00E-06	12.75822321

ENSG00000196091	MYBPC1	0.006891727	1.00E-06	12.7506499
ENSG00000250579	CTD-2297D10.2	0.00687	1.00E-06	12.74609438
ENSG00000257853	MED15P1	0.006854485	1.00E-06	12.74283253
ENSG00000154856	APCDD1	0.006851235	1.00E-06	12.7421483
ENSG00000169876	MUC17	0.006842547	1.00E-06	12.74031774
ENSG00000217874	OR4F7P	0.006833	1.00E-06	12.73830341
ENSG00000274764	PRAMEF27	0.00683	1.00E-06	12.73766986
ENSG00000204505	PRAMEF9	0.00683	1.00E-06	12.73766986
ENSG00000225087	RP4-660H19.1	0.0068	1.00E-06	12.73131903
ENSG00000266971	OR4F8P	0.00679	1.00E-06	12.72919586
ENSG00000146216	TTBK1	0.006783253	1.00E-06	12.7277615
ENSG00000224777	OR4F2P	0.006782	1.00E-06	12.72749507
ENSG00000220212	OR4F1P	0.006782	1.00E-06	12.72749507
ENSG00000152208	GRID2	0.006779376	1.00E-06	12.72693683
ENSG00000232762	RP4-784A16.4	0.006768	1.00E-06	12.72451385
ENSG00000157554	ERG	0.006707135	1.00E-06	12.71148091
ENSG00000249158	PCDHA11	0.006657993	1.00E-06	12.70087171
ENSG00000120645	IQSEC3	0.006621436	1.00E-06	12.69292838
ENSG00000118492	ADGB	0.006547888	1.00E-06	12.67681389
ENSG00000203685	C1orf95	0.006530415	1.00E-06	12.67295899
ENSG00000278973	RP11-83B20.9	0.006525	1.00E-06	12.67176219
ENSG00000185662	SMIM23	0.006525	1.00E-06	12.67176219
ENSG00000185985	SLITRK2	0.006469562	1.00E-06	12.65945243
ENSG00000232225	LINC01047	0.00645592	1.00E-06	12.65640691
ENSG00000163295	ALPI	0.006445726	1.00E-06	12.65412708
ENSG00000236445	LINC00608	0.006325965	1.00E-06	12.6270699
ENSG00000229571	PRAMEF25	0.006321058	1.00E-06	12.62595037
ENSG00000248772	RP11-639F1.1	0.006284	1.00E-06	12.61746747
ENSG00000149256	TENM4	0.006232795	1.00E-06	12.60566343
ENSG00000243981	RP11-368M16.3	0.006225085	1.00E-06	12.60387785
ENSG00000232044	LINC01105	0.006206961	1.00E-06	12.59967139
ENSG00000139656	SMIM2	0.006176971	1.00E-06	12.59268381
ENSG00000189212	DPY19L2P1	0.006153026	1.00E-06	12.58708031
ENSG00000060718	COL11A1	0.006145797	1.00E-06	12.58538432
ENSG00000234718	AC007161.5	0.006124272	1.00E-06	12.58032266
ENSG00000234377	RNF219-AS1	0.006112121	1.00E-06	12.57745735
ENSG00000081237	PTPRC	0.006080084	1.00E-06	12.56987556
ENSG00000107562	CXCL12	0.00603023	1.00E-06	12.55799733
ENSG00000227028	SLC8A1-AS1	0.006005513	1.00E-06	12.55207184
ENSG00000145626	UGT3A1	0.005998362	1.00E-06	12.55035277
ENSG00000277736	MIR8085	0.005933	1.00E-06	12.53454607
ENSG00000160593	JAML	0.00592751	1.00E-06	12.53321052
ENSG00000139797	RNF113B	0.005918	1.00E-06	12.53089398
ENSG00000247765	RP11-32B5.7	0.005877	1.00E-06	12.52086418
ENSG00000266509	MIR3934	0.005826	1.00E-06	12.50828999
ENSG00000112992	NNT	0.005802855	1.00E-06	12.50254718
ENSG00000176593	CTD-2368P22.1	0.005791782	1.00E-06	12.49979163
ENSG00000203910	C1orf146	0.005756317	1.00E-06	12.49093025

ENSG00000131951	LRRC9	0.005744337	1.00E-06	12.48792465
ENSG00000198626	RYR2	0.005724975	1.00E-06	12.48305363
ENSG00000121853	GHSR	0.005718665	1.00E-06	12.48146256
ENSG00000120251	GRIA2	0.00569204	1.00E-06	12.47473021
ENSG00000167210	LOXHD1	0.005642388	1.00E-06	12.46209005
ENSG00000144619	CNTN4	0.005580441	1.00E-06	12.44616331
ENSG00000119283	TRIM67	0.005457487	1.00E-06	12.41402097
ENSG00000280267	PRAMEF26	0.005319519	1.00E-06	12.37708015
ENSG00000154545	MAGED4	0.00529208	1.00E-06	12.36961928
ENSG00000151892	GFRA1	0.005258396	1.00E-06	12.36040695
ENSG00000112038	OPRM1	0.005255311	1.00E-06	12.3595605
ENSG00000235855	OR7E145P	0.005231	1.00E-06	12.35287105
ENSG00000187243	MAGED4B	0.005144364	1.00E-06	12.32877709
ENSG00000198914	POU3F3	0.005129	1.00E-06	12.32446186
ENSG00000258423	OR7E105P	0.005125	1.00E-06	12.32333629
ENSG00000273898	MIR6798	0.005064	1.00E-06	12.30606169
ENSG00000196209	SIRPB2	0.005055698	1.00E-06	12.3036947
ENSG00000237164	SLC25A15P2	0.005019	1.00E-06	12.29318423
ENSG00000179170	OR7E97P	0.005014	1.00E-06	12.29174628
ENSG00000118729	CASQ2	0.004962831	1.00E-06	12.27694771
ENSG00000230461	PROX1-AS1	0.004946151	1.00E-06	12.27209046
ENSG00000158321	AUTS2	0.00488449	1.00E-06	12.2539922
ENSG00000214140	PRCD	0.004797075	1.00E-06	12.22793934
ENSG00000233029	RP11-439A17.9	0.004788	1.00E-06	12.22520744
ENSG00000182168	UNC5C	0.00476329	1.00E-06	12.21774257
ENSG00000263189	RP11-618P13.1	0.004734	1.00E-06	12.20884399
ENSG00000237425	RPSAP2	0.0047	1.00E-06	12.19844504
ENSG00000170153	RNF150	0.004623235	1.00E-06	12.17468705
ENSG00000177822	AC108142.1	0.004584388	1.00E-06	12.16251339
ENSG00000135517	MIP	0.004559894	1.00E-06	12.15478466
ENSG00000231205	ZNF826P	0.004518336	1.00E-06	12.1415757
ENSG00000188730	VWC2	0.004504	1.00E-06	12.13699111
ENSG00000173157	ADAMTS20	0.004497851	1.00E-06	12.13502011
ENSG00000162594	IL23R	0.004393137	1.00E-06	12.10103571
ENSG00000165583	SSX5	0.004359434	1.00E-06	12.08992514
ENSG00000276783	AC009065.3	0.004242	1.00E-06	12.05052891
ENSG00000277652	AC139256.1	0.004242	1.00E-06	12.05052891
ENSG00000277330	AC138969.1	0.004242	1.00E-06	12.05052891
ENSG00000275085	AC126755.1	0.004242	1.00E-06	12.05052891
ENSG00000274969	MIR6771	0.004242	1.00E-06	12.05052891
ENSG00000085265	FCN1	0.004189188	1.00E-06	12.03245482
ENSG00000180251	SLC9A4	0.004091796	1.00E-06	11.99851862
ENSG00000182048	TRPC2	0.004056226	1.00E-06	11.98592241
ENSG00000248257	PSG10P	0.003996976	1.00E-06	11.96469332
ENSG00000163092	XIRP2	0.003906285	1.00E-06	11.9315815
ENSG00000231248	RP11-160N1.9	0.003899	1.00E-06	11.92888844
ENSG00000178031	ADAMTSL1	0.003864422	1.00E-06	11.91603703
ENSG00000156475	PPP2R2B	0.003854121	1.00E-06	11.9121861

ENSG00000234456	MAGI2-AS3	0.00385281	1.00E-06	11.91169532
ENSG00000137766	UNC13C	0.003781729	1.00E-06	11.88483023
ENSG00000196724	ZNF418	0.003769505	1.00E-06	11.88015937
ENSG00000161640	SIGLEC11	0.003769	1.00E-06	11.87996608
ENSG00000275711	MIR6795	0.003742	1.00E-06	11.86959384
ENSG00000241476	SSX2	0.003728908	1.00E-06	11.86453737
ENSG00000139287	TPH2	0.003672695	1.00E-06	11.8426234
ENSG00000104177	MYEF2	0.003618304	1.00E-06	11.82109784
ENSG00000227518	XXyac-YRM2039.3	0.00357	1.00E-06	11.80170836
ENSG00000236172	MIR7515HG	0.00356975	1.00E-06	11.80160734
ENSG00000242512	LINC01206	0.003526248	1.00E-06	11.78391834
ENSG00000230102	RP11-407B7.1	0.003464058	1.00E-06	11.75824737
ENSG00000165584	SSX3	0.003451003	1.00E-06	11.75280015
ENSG00000180660	MAB21L1	0.003446	1.00E-06	11.75070699
ENSG00000048740	CELF2	0.003416791	1.00E-06	11.73842614
ENSG00000163618	CADPS	0.003407508	1.00E-06	11.73450147
ENSG00000259660	DNM1P47	0.003331295	1.00E-06	11.70186721
ENSG00000173068	BNC2	0.003321854	1.00E-06	11.69777304
ENSG00000264668	RP13-582O9.6	1.213449	0.000366	11.69498221
ENSG00000144290	SLC4A10	0.003235415	1.00E-06	11.65973487
ENSG00000258367	RP11-536C10.3	0.003134	1.00E-06	11.61378946
ENSG00000223379	RP11-374M1.4	0.003104	1.00E-06	11.59991284
ENSG00000264302	MIR4723	0.002905	1.00E-06	11.50432245
ENSG00000258867	LINC01146	0.00290089	1.00E-06	11.50227983
ENSG00000249388	RP11-834C11.6	0.002892	1.00E-06	11.49785184
ENSG00000144810	COL8A1	0.002705145	1.00E-06	11.40149026
ENSG00000151812	SLC35F4	0.002400708	1.00E-06	11.22924395
ENSG00000261427	CTD-2349B8.1	0.002063485	1.00E-06	11.01086694
ENSG00000250007	RP11-814P5.1	0.001973417	1.00E-06	10.94648032
ENSG00000266885	RP11-744K17.8	0.001955382	1.00E-06	10.93323476
ENSG00000227914	RP11-130C19.3	0.001928219	1.00E-06	10.913053
ENSG00000081138	CDH7	0.001751092	1.00E-06	10.77403877
ENSG00000279072	RP13-580B18.4	0.001055	1.00E-06	10.04302728
ENSG00000198670	LPA	0.001030197	1.00E-06	10.00870441
ENSG00000221062	MIR1292	0.137455	0.000141	9.929048509
ENSG00000258611	RP11-368J22.2	0.193416	0.000265	9.511499069
ENSG00000278050	NEAT1_2	122.573997	0.183529	9.383429195
ENSG00000264594	MIR4640	0.000525	1.00E-06	9.036173613
ENSG00000273778	MIR6765	3.164926	0.006221	8.990809546
ENSG00000220988	SNORD88C	0.65192	0.001501	8.762627149
ENSG00000263365	AC123788.1	0.593789	0.001455	8.672787404
ENSG00000147724	FAM135B	0.00031751	1.00E-06	8.310658637
ENSG00000254905	RP11-712L6.7	0.102956	0.000397	8.018673186
ENSG00000185085	INTS5	7.185505	0.03295	7.768667278
ENSG00000177374	HIC1	1.266995075	0.006140714	7.688788792
ENSG00000277830	ZNFX1-AS1_1	0.000203	1.00E-06	7.665335917
ENSG00000276587	MIR6789	0.110509	0.000616	7.487017803
ENSG00000282034	RP11-2C24.9	9.724463	0.057373	7.405102817

ENSG00000221410	MIR1238	1.030188	0.00652	7.30381996
ENSG00000277599	NEAT1_3	4.906949	0.031593	7.279077528
ENSG00000275770	MIR6505	0.716848	0.005082	7.140127058
ENSG00000269837	IPO5P1	0.120702671	0.001056045	6.836642908
ENSG00000170396	ZNF804A	1.168856108	0.011530166	6.663540223
ENSG00000223396	RP11-134G8.5	2.284938	0.023237	6.619587387
ENSG00000141086	CTRL	0.555064302	0.005894905	6.557042545
ENSG00000265480	KRT18P55	2.37352953	0.025944535	6.51545951
ENSG00000275259	MIR6511A1	0.143573	0.001616	6.473213457
ENSG00000276311	MIR6511A3	0.143573	0.001616	6.473213457
ENSG00000273613	MIR6511A4	0.143573	0.001616	6.473213457
ENSG00000278221	MIR6511A2	0.142053	0.001616	6.457858292
ENSG00000233984	RPSAP14	18.876209	0.216247	6.447745122
ENSG00000224631	RP11-51O6.1	147.7734749	1.712606523	6.431049795
ENSG00000276511	MIR7703	0.664852	0.00914	6.184695248
ENSG00000256713	PGA5	0.069888593	0.000991044	6.139963672
ENSG00000275967	MIR6880	0.607471	0.00873	6.120690074
ENSG00000215867	KRT18P57	0.29799	0.004316	6.109425242
ENSG00000204282	TNRC6C-AS1	0.83381647	0.012168473	6.098509883
ENSG00000275666	KCNQ1OT1_1	0.000513	8.00E-06	6.002815016
ENSG00000169136	ATF5	1.154530386	0.018321226	5.977646275
ENSG00000106328	FSCN3	0.230566146	0.003704468	5.959770536
ENSG00000279803	RP11-407G23.5	0.649957	0.010896	5.898473762
ENSG00000239344	RP11-771F20.1	37.770103	0.646567	5.868301032
ENSG00000263608	RN7SL353P	0.201969	0.003681	5.777892312
ENSG00000265371	MIR3198-2	11.389616	0.207893	5.775734116
ENSG00000257390	RP11-762I7.5	2.213347236	0.040839931	5.760105566
ENSG00000198865	CCDC152	1.687747	0.031548	5.741406297
ENSG00000089505	CMTM1	1.278457813	0.024381941	5.712447746
ENSG00000266044	MIR4469	0.068721	0.00137	5.648503232
ENSG00000276598	MIR6893	0.745002	0.015687	5.569602917
ENSG00000204947	ZNF425	0.652162799	0.014679078	5.473398904
ENSG00000186594	MIR22HG	0.778057164	0.018124325	5.423876994
ENSG00000133665	DYDC2	0.148662501	0.003512217	5.403515145
ENSG00000176490	DIRAS1	11.28247007	0.274624623	5.360478156
ENSG00000270797	RP5-1173I20.1	0.24026	0.005948	5.336048022
ENSG00000279589	RP11-454F8.4	0.085324	0.002117	5.332858427
ENSG00000182118	FAM89A	10.84669071	0.271280788	5.321324259
ENSG00000213658	LAT	0.550648116	0.013856734	5.312471556
ENSG00000213762	ZNF134	3.496762422	0.088790263	5.299474492
ENSG00000234005	GAPDHP22	0.598924	0.015725	5.251241023
ENSG00000252503	RNU6-531P	1.957546	0.052039	5.23330916
ENSG00000187624	C17orf97	2.209409501	0.059912041	5.204671039
ENSG00000276753	MIR6821	0.268237	0.007314	5.196703816
ENSG00000171303	KCNK3	0.266299796	0.007305678	5.187889289
ENSG00000270617	URGCP-MRPS24	2.359365	0.066943	5.139321609
ENSG00000103740	ACSBG1	2.519510783	0.072336007	5.12228585
ENSG00000233963	ATP8A2P3	0.049675	0.001434	5.114403038

ENSG00000207406	SNORA41	1.29554	0.038414	5.075777552
ENSG00000136630	HLX	0.320615835	0.009561218	5.067507453
ENSG00000226147	TUBBP10	4.117988	0.124361	5.049333598
ENSG00000259471	LINC01169	0.053768	0.00167	5.008827801
ENSG00000274021	RP11-823E8.3	27.106703	0.848378	4.997798628
ENSG00000259319	RP11-293M10.6	0.424323	0.013333	4.992089544
ENSG00000230561	LINC01183	0.447993681	0.014242453	4.975208868
ENSG00000258466	RP11-1012A1.4	1.030306249	0.033016037	4.963762444
ENSG00000104043	ATP8B4	0.046111718	0.001494891	4.947021667
ENSG00000272913	RP11-440D17.3	1.033889	0.033953	4.928398441
ENSG00000184809	B3GALT5-AS1	0.651116758	0.021510823	4.919781634
ENSG00000083812	ZNF324	0.856120459	0.02866589	4.900406843
ENSG00000206936	RNU4-52P	1.608025	0.054905	4.87220849
ENSG00000172780	RAB43	7.685381508	0.264475756	4.860909492
ENSG00000249148	AC006445.7	0.344028	0.012122	4.826826336
ENSG00000258365	RP11-1105G2.3	1.361976982	0.048495129	4.811718664
ENSG00000165917	RAPSN	0.110659753	0.003950112	4.808093399
ENSG00000114786	ABHD14A-ACY1	3.550778049	0.129214022	4.78030064
ENSG00000226668	RP11-526D8.7	0.076850933	0.002798527	4.779322994
ENSG00000273489	RP11-180C16.1	0.754175	0.02756	4.774251535
ENSG00000257921	RP11-571M6.15	0.566985102	0.020724	4.773936433
ENSG00000258701	LINC00638	0.500068	0.018484	4.757775388
ENSG00000240163	RP11-745A24.1	1.778105	0.065916	4.753568012
ENSG00000159958	TNFRSF13C	0.606134	0.022779	4.733860451
ENSG00000170961	HAS2	0.686067	0.025833	4.731062375
ENSG00000245025	RP11-875O11.1	1.248041667	0.047725672	4.708756766
ENSG00000277449	CEBPB-AS1	0.266398	0.010219	4.704257321
ENSG00000166246	C16orf71	0.150991482	0.005811949	4.699301286
ENSG00000164850	GPER1	0.892502119	0.034373593	4.698483046
ENSG00000233652	CICP1	0.057989	0.002246	4.690349427
ENSG00000122194	PLG	0.053183058	0.002078218	4.677547479
ENSG00000206262	FOXL2NB	0.302818601	0.011850402	4.67544592
ENSG00000203883	SOX18	1.482107	0.058069	4.673737606
ENSG00000279010	RP5-1039K5.19	0.120064	0.004764	4.655486415
ENSG00000244480	AC005154.7	0.242277	0.00962	4.654476748
ENSG00000233198	RNF224	0.302886	0.01204	4.652867598
ENSG00000235946	LL0XNC01-240C2.1	0.402244	0.016367	4.61920909
ENSG00000140835	CHST4	0.164251171	0.006758894	4.602972586
ENSG00000163453	IGFBP7	0.971770734	0.040828466	4.572968704
ENSG00000231887	PRH1	0.255674662	0.010829155	4.56131667
ENSG00000277589	RP11-333J10.3	0.358424	0.015246	4.555164557
ENSG00000261334	RP11-65J3.14	0.184216	0.007836	4.555137164
ENSG00000266680	RP5-1148A21.3	1.732791	0.074011	4.549214135
ENSG00000203326	ZNF525	0.386908218	0.016721568	4.532209347
ENSG00000233203	RP11-67L3.4	0.713646608	0.031127542	4.518946254
ENSG00000272902	RP11-299H21.1	0.349167	0.015288	4.513445627
ENSG00000248690	HAS2-AS1	0.68182839	0.030133483	4.499969314
ENSG00000092969	TGFB2	2.211449967	0.098677335	4.486130038

ENSG00000213424	KRT222	1.904251836	0.085390941	4.478997452
ENSG00000111834	RSPH4A	0.415565458	0.018674842	4.475907834
ENSG00000205097	FRG2	0.056471327	0.002573324	4.455813605
ENSG00000157445	CACNA2D3	0.076289172	0.003476692	4.455691288
ENSG00000225899	FRG2B	0.056632864	0.002581302	4.455468727
ENSG00000231133	HAR1B	1.304147	0.060403	4.43234248
ENSG00000279880	RP11-855A2.2	0.523754	0.024289	4.430514358
ENSG00000236833	AC024560.2	0.789566	0.036791	4.423635071
ENSG00000249678	RP11-619J20.1	0.976723	0.048107	4.34363073
ENSG00000259939	RP11-77H9.5	0.138786	0.006849	4.340824873
ENSG00000201229	SNORA63	1.156732	0.0574	4.332860102
ENSG00000188368	PRR19	0.411914579	0.02059687	4.321848177
ENSG00000224738	AC099850.1	0.484405	0.02427	4.318967743
ENSG00000248710	RP11-432B6.3	0.729607	0.036896	4.305583248
ENSG00000164379	FOXQ1	1.851257	0.094672	4.28942358
ENSG00000240963	RP11-518L10.5	4.834659	0.247986	4.285083556
ENSG00000227542	AC092614.2	0.162375	0.008336	4.283830438
ENSG00000277578	Metazoa_SRP	0.075282	0.00388	4.278176399
ENSG00000145358	DDIT4L	10.64491784	0.560004425	4.248582778
ENSG00000244255	XXbac-BPG116M5.17	0.298636411	0.015824466	4.238161315
ENSG00000180626	ZNF594	1.712120171	0.090845941	4.236218092
ENSG00000242731	FAM86LP	0.238868	0.012689	4.234563314
ENSG00000253731	PCDHGA6	0.025737143	0.001368571	4.233109266
ENSG00000198673	FAM19A2	0.109289929	0.005847442	4.224210888
ENSG00000251485	AC068134.10	3.800423	0.206042	4.20514965
ENSG00000212123	PRR22	1.078737138	0.059335127	4.184313092
ENSG00000230284	RP11-402G3.4	0.354379	0.019623	4.174675589
ENSG00000225764	P3H2-AS1	0.126892	0.007043	4.171267223
ENSG00000184588	PDE4B	10.85164769	0.606130134	4.162142736
ENSG00000271532	RP11-426K3.1	0.897524	0.050435	4.153453355
ENSG00000259529	RP11-468E2.4	1.072322	0.060278	4.152962828
ENSG00000264455	MIR4721	1.297558	0.073528	4.141361474
ENSG00000253335	RP11-420B22.1	0.278294	0.015889	4.13050957
ENSG00000260910	LINC00565	0.406121	0.023218	4.12859402
ENSG00000223284	RNU6-195P	1.699653	0.099304	4.097244595
ENSG00000197385	ZNF860	0.573500716	0.033640331	4.091531482
ENSG00000265039	AC107016.1	0.733724	0.043082	4.090080345
ENSG00000235959	AC009237.17	1.071941	0.063821	4.070050477
ENSG00000183169	POM121L1P	0.054672183	0.003255869	4.069692217
ENSG00000152433	ZNF547	0.1491625	0.008912438	4.064920882
ENSG00000227120	AC009238.7	1.06714	0.063821	4.063574434
ENSG00000180438	TPRXL	0.026875361	0.001616133	4.055666203
ENSG00000206567	AC022007.5	0.699548543	0.042732322	4.033024549
ENSG00000253620	AC144568.4	0.446175	0.027652	4.012153943
ENSG00000230519	RP11-1060J15.5	0.192613	0.011957	4.009777709
ENSG00000267702	RP11-53B2.2	0.10288	0.00642	4.00224544
ENSG00000266610	RN7SL176P	0.096394	0.00605	3.993936302
ENSG00000250942	ENPP7P11	0.340931	0.021579	3.981779871

ENSG00000278922	AC002310.14	0.620956	0.039303	3.981779702
ENSG00000257528	KRT8P19	0.081853	0.005207	3.974510982
ENSG00000224490	TTC21B-AS1	0.079587694	0.00507783	3.970261492
ENSG00000158486	DNAH3	0.056686684	0.003617126	3.970094144
ENSG00000277999	RP11-426C22.8	1.513081	0.097867	3.950522935
ENSG00000233740	CICP2	0.035926	0.002325	3.949725693
ENSG00000213976	CTD-2561J22.2	0.971638	0.063094	3.944844192
ENSG00000275101	MIR6766	0.346377	0.02255	3.941143797
ENSG00000257231	DYNLL1P4	0.619939	0.040692	3.929309171
ENSG00000275466	MIR6716	0.20833	0.013741	3.922311701
ENSG00000272593	RP11-339B21.11	0.336946	0.022295	3.917725294
ENSG00000269749	AC005614.5	0.273288	0.018086	3.917476839
ENSG00000146094	DOK3	1.243604053	0.08240808	3.915597621
ENSG00000129749	CHRNA10	0.5960048	0.039659761	3.909576048
ENSG00000081148	IMPG2	0.194867	0.012984	3.907682986
ENSG00000180066	C10orf91	0.789888922	0.053058926	3.895982397
ENSG00000253395	KB-1460A1.1	0.63568	0.042793	3.892853971
ENSG00000249753	RP11-415I12.3	0.522312	0.035424	3.882112817
ENSG00000259500	KRT8P24	2.312239	0.15749	3.875958396
ENSG00000253552	HOXA-AS2	0.735991607	0.05013795	3.8757144
ENSG00000276771	HOTAIRM1_2	0.888773	0.060802	3.869624305
ENSG00000265458	RP13-20L14.6	1.733515	0.118685276	3.868487445
ENSG00000180834	MAP6D1	4.907630494	0.337102767	3.863766255
ENSG00000266852	MIR4482	2.006765	0.139167	3.849982624
ENSG00000267680	ZNF224	2.405562777	0.168384615	3.836542216
ENSG00000173208	ABCD2	0.121734	0.008676	3.810558302
ENSG00000225901	MTND2P9	0.633879	0.045246	3.808345313
ENSG00000174236	REP15	1.882066	0.134548	3.80612437
ENSG00000135960	EDAR	0.513101812	0.036682989	3.806062004
ENSG00000271550	BNIP3P11	1.342916	0.096129	3.80425353
ENSG00000168772	CXXC4	0.519596371	0.037202967	3.803901765
ENSG00000180938	ZNF572	1.150643	0.082627	3.799683193
ENSG00000162461	SLC25A34	0.235445398	0.016939358	3.796941388
ENSG00000260425	LA16c-316G12.2	0.103458	0.007465	3.792759137
ENSG00000259146	RP1-261D10.2	0.767413941	0.055622244	3.786271131
ENSG00000224903	AC005534.8	1.004157	0.072938	3.783170401
ENSG00000196214	ZNF766	3.110201893	0.227187935	3.775050109
ENSG00000261428	RP11-16P6.1	1.312368	0.095924	3.774136691
ENSG00000223768	LINC00205	2.646969	0.19376	3.771998624
ENSG00000185065	AC000068.5	0.350429	0.025809	3.763176022
ENSG00000262560	RP11-296A16.1	1.24163	0.091952	3.755210557
ENSG00000259080	RP11-158I13.2	1.220951	0.090881	3.747882783
ENSG00000267908	ZSCAN5D	0.162642	0.012124	3.745762199
ENSG00000075886	TUBA3D	0.225721805	0.016934408	3.73651633
ENSG00000064489	BORCS8-MEF2B	5.041439636	0.378287952	3.736279034
ENSG00000082126	MPP4	0.145535975	0.0109915	3.726915631
ENSG00000277639	RP11-295M3.4	0.619068	0.04702	3.718751443
ENSG00000258303	RP11-887P2.6	0.256115	0.019453	3.718727184

ENSG00000251365	RP11-332J15.3	0.167377	0.012713	3.718724873
ENSG00000273456	RP11-686O6.2	1.054224	0.080417	3.712537114
ENSG00000136931	NR5A1	0.206738011	0.015814798	3.708456598
ENSG00000136488	CSH1	0.141639354	0.010867922	3.704074191
ENSG00000197479	PCDHB11	0.083897	0.006446	3.70214313
ENSG00000279718	SNX18P12	0.364789	0.028046	3.701195297
ENSG00000197180	CH17-340M24.3	1.17623	0.090503	3.700060767
ENSG00000224914	LINC00863	2.314806	0.178372	3.697930218
ENSG00000148677	ANKRD1	41.879684	3.227831	3.697613606
ENSG00000163126	ANKRD23	0.169504324	0.01314898	3.688299325
ENSG00000145087	STXBP5L	0.062627341	0.004882066	3.681228853
ENSG00000270587	RP11-51F16.9	0.635894	0.049796	3.674684533
ENSG00000267149	CTC-550B14.6	0.431488	0.034065	3.662958326
ENSG00000230203	CTB-1048E9.7	0.121091	0.00958	3.659922176
ENSG00000242396	RP11-67L3.5	1.186148	0.094123	3.655592917
ENSG00000224715	CITF22-49D8.1	0.056215	0.004465	3.65422306
ENSG00000259287	RP11-3D4.2	0.628505	0.050076	3.64973299
ENSG00000171161	ZNF672	4.649873653	0.373148903	3.639368167
ENSG00000264175	MIR3189	12.549749	1.008273	3.637700289
ENSG00000223718	AC093107.7	0.183862	0.014812	3.633784979
ENSG00000119686	FLVCR2	0.984359731	0.079313629	3.633544933
ENSG00000240875	LINC00886	0.6015763	0.048586609	3.630117077
ENSG00000224950	RP5-1086K13.1	0.110548	0.008934	3.62922286
ENSG00000257218	GATC	4.597116218	0.373583368	3.621227006
ENSG00000229689	AC009237.8	0.293619884	0.023918246	3.617766152
ENSG00000242583	RP11-379K17.9	0.938374	0.076441	3.617744484
ENSG00000224358	RP11-466F5.8	0.160417	0.01309	3.615290035
ENSG00000258056	RP11-644F5.11	1.37728	0.112517	3.613606991
ENSG00000259985	RP11-549B18.1	0.565997	0.046297	3.61180379
ENSG00000138449	SLC40A1	0.305142068	0.024997823	3.609606718
ENSG00000255856	RP11-87C12.5	0.577981795	0.047349606	3.609599718
ENSG00000182791	CCDC87	0.208275	0.017122	3.604566542
ENSG00000196247	ZNF107	4.470484289	0.368575975	3.600397189
ENSG00000256243	RPL7AP3	0.250312	0.020639	3.60028248
ENSG00000273218	LLNLR-246C6.1	0.185287	0.015297	3.598441014
ENSG00000212901	KRTAP3-1	11.62820381	0.970437054	3.582849816
ENSG00000183153	GJD3	0.732675	0.061153	3.582678209
ENSG00000264769	RP11-498C9.12	0.564593	0.047221	3.579710743
ENSG00000269901	RP11-178L8.9	1.234193	0.103431	3.576827462
ENSG00000282827	ATRIP	1.39120556	0.117214934	3.569107311
ENSG00000279213	SNX18P10	0.309398	0.02607	3.569001383
ENSG00000131095	GFAP	0.04737521	0.00400176	3.565425759
ENSG00000198585	NUDT16	5.094879709	0.431485808	3.561663071
ENSG00000187536	TPM3P7	0.217862	0.018484	3.55906568
ENSG00000196227	FAM217B	3.031634861	0.258452786	3.552123341
ENSG00000229619	MBNL1-AS1	0.191836854	0.016437	3.544860993
ENSG00000240032	RP11-274H2.3	0.142136	0.012213	3.540782472
ENSG00000132016	C19orf57	0.163746476	0.014093314	3.538381019

ENSG00000243335	KCTD7	0.887235762	0.076524718	3.535319786
ENSG00000221886	ZBED8	1.71447973	0.148021971	3.533887614
ENSG00000254539	RP4-791M13.3	0.718857	0.062279	3.528887124
ENSG00000223839	FAM95B1	0.642253264	0.055785233	3.525187141
ENSG00000259431	THTPA	1.720308651	0.14960745	3.523413508
ENSG00000241170	RP11-147I3.1	0.404645159	0.035326478	3.517835491
ENSG00000169218	RSPO1	0.800102831	0.070082265	3.513064127
ENSG00000006555	TTC22	0.568518856	0.049995339	3.507342697
ENSG00000226200	SGMS1-AS1	0.346766699	0.03050263	3.506959812
ENSG00000236662	RP11-108L7.4	0.72209	0.063699	3.502836034
ENSG00000231528	FAM225A	0.050061578	0.004436	3.496372493
ENSG00000270277	RP11-65L3.2	0.339685	0.030101	3.496314191
ENSG00000206014	OR7E161P	0.06316	0.005597	3.496285518
ENSG00000272582	RP5-1039K5.17	0.067772	0.006044	3.487113781
ENSG00000186340	THBS2	0.189359388	0.016920665	3.484268734
ENSG00000261335	RP11-318A15.2	0.703713	0.063041	3.48062484
ENSG00000232654	FAM136BP	0.723776	0.064893	3.479408501
ENSG00000224324	THAP5P1	1.151963	0.103284	3.479405695
ENSG00000235890	TSPEAR-AS1	0.158819053	0.014248469	3.478505205
ENSG00000267247	RP11-64C12.6	1.242498	0.111589	3.476976805
ENSG00000270959	LPP-AS2	0.823762	0.074008	3.476474442
ENSG00000124160	NCOA5	15.29478553	1.377472	3.472944978
ENSG00000207584	MIR196B	0.013539	0.00122	3.472168131
ENSG00000135482	ZC3H10	1.163405566	0.104844192	3.472035256
ENSG00000227621	PHBP11	0.311626	0.028097	3.471327604
ENSG00000281831	HCP5B	0.107774	0.009741	3.467795481
ENSG00000198081	ZBTB14	0.955285573	0.08669871	3.461849637
ENSG00000253986	CTC-756D1.3	0.832135	0.075693	3.458585808
ENSG00000274258	MIR6728	1.265783	0.115539	3.453578279
ENSG00000268509	RP11-572B2.1	0.525671	0.048022	3.452392752
ENSG00000261423	RP11-1007O24.3	0.435788813	0.039897788	3.449248479
ENSG00000250056	LINC01018	0.334585512	0.030767331	3.442903752
ENSG00000241790	ENO1P4	0.693157	0.06377	3.442232373
ENSG00000261159	RP11-723O4.9	2.349634	0.216215	3.441897528
ENSG00000106624	AEBP1	0.030815462	0.002838992	3.440203571
ENSG00000225806	RP1-309F20.3	0.100386715	0.009251347	3.439761083
ENSG00000249337	SNX18P25	0.192595	0.01785	3.43157427
ENSG00000269908	RP11-1406H17.1	0.399387	0.037024	3.431254699
ENSG00000267838	AC008746.12	0.661651	0.0615	3.427412126
ENSG00000254605	RP11-626H12.2	2.562050691	0.239119374	3.421496089
ENSG00000177839	PCDHB9	0.1182	0.011033	3.421333001
ENSG00000125841	NRSN2	0.243287975	0.022815333	3.41458939
ENSG00000258948	KRT8P1	0.057107	0.005381	3.407721386
ENSG00000198538	ZNF28	1.858759155	0.175967382	3.400959914
ENSG00000240935	PLGLA	0.141064178	0.013379818	3.398221234
ENSG00000249152	RP11-562F9.2	0.049385	0.004686	3.397644051
ENSG00000282021	CTD-2336O2.3	1.015937	0.096644	3.393986963
ENSG00000128000	ZNF780B	1.738773091	0.165965788	3.389111888

ENSG00000213971	RP11-15H20.6	0.102155951	0.009780136	3.384774924
ENSG00000136367	ZFHX2	0.876498994	0.084109419	3.381413158
ENSG00000213385	RP11-577H5.1	9.505298	0.914085	3.378331626
ENSG00000256116	RP11-783K16.14	0.384479145	0.037075947	3.374349907
ENSG00000171658	RP11-443P15.2	0.42684641	0.041187591	3.373435382
ENSG00000279048	RP11-511H23.2	0.231347	0.022364	3.370808236
ENSG00000267607	CTD-2369P2.8	0.173924	0.016813	3.370807948
ENSG00000196096	AC079610.2	0.324179	0.031338	3.370805623
ENSG00000101306	MYLK2	0.188124171	0.018185956	3.370788578
ENSG00000164778	EN2	0.164907	0.015942	3.370748101
ENSG00000229186	ADAM1A	0.110818	0.010722	3.36954629
ENSG00000240399	RP1-228P16.1	0.544896338	0.0528	3.36737196
ENSG00000175093	SPSB4	0.596547978	0.057882988	3.365426863
ENSG00000139438	FAM222A	5.064050636	0.491767119	3.364244651
ENSG00000186710	CFAP73	0.505711964	0.04919272	3.361799189
ENSG00000261485	PAN3-AS1	0.938811	0.091336	3.361579232
ENSG00000269897	COMMD3-BMI1	3.219260072	0.313332864	3.360961129
ENSG00000162039	MEIOB	0.24091354	0.023458944	3.360305495
ENSG00000172404	DNAJB7	0.64557	0.06298	3.357607875
ENSG00000204179	PTPN20	0.061220024	0.006005188	3.349722406
ENSG00000259354	RP11-519G16.3	0.07098783	0.006963644	3.349657387
ENSG00000113140	SPARC	13.64968964	1.340714928	3.34779373
ENSG00000229956	ZRANB2-AS2	0.040481187	0.004073153	3.313033844
ENSG00000177614	PGBD5	0.151227258	0.015227832	3.311935728
ENSG00000248618	ENPP7P3	0.37169	0.037428	3.311910011
ENSG00000231010	RP6-109B7.2	0.455071	0.045826	3.311853383
ENSG00000166192	SENP8	0.965064966	0.097231793	3.311126027
ENSG00000171766	GATM	0.242522905	0.024437302	3.31096408
ENSG00000172081	MOB3A	7.958088398	0.803250897	3.308499332
ENSG00000265452	MIR3682	1.781367	0.180402	3.303697535
ENSG00000172086	KRCC1	2.728013	0.277858	3.295430836
ENSG00000274455	CTD-2012B7.1	0.034158	0.003486	3.292579031
ENSG00000207181	SNORA14B	0.942522	0.096214	3.292207554
ENSG00000187944	C2orf66	0.634907	0.064854	3.291277821
ENSG00000220685	RP11-530A18.1	0.445556	0.045557	3.289862118
ENSG00000280160	RP11-196G11.3	0.476461	0.048802	3.287345951
ENSG00000180257	ZNF816	1.702701327	0.175101578	3.2815614
ENSG00000247081	BAALC-AS1	0.64839034	0.067005072	3.274520381
ENSG00000204682	CASC10	1.754922	0.181398	3.274176455
ENSG00000266208	CTD-2267D19.3	0.740109	0.076503	3.274149532
ENSG00000242349	NPPA-AS1	0.886082287	0.091908025	3.269177938
ENSG00000255121	RP11-110I1.12	1.036546759	0.107809488	3.265229142
ENSG00000010704	HFE	1.705997573	0.177945364	3.26110934
ENSG00000260920	RP1-228H13.5	0.847092	0.088396	3.260465672
ENSG00000261305	RP4-584D14.7	0.617808	0.06466	3.25621314
ENSG00000198003	CCDC151	0.40747832	0.042795715	3.251185045
ENSG00000272173	U47924.31	2.06493	0.216982	3.250445604
ENSG00000179409	GEMIN4	5.496947417	0.578917321	3.247201451

ENSG00000200831	SNORD36B	2.05788	0.218154	3.237740026
ENSG00000278978	RP11-164P12.5	3.554228	0.377261	3.23590136
ENSG00000187116	LILRA5	0.028918098	0.003074839	3.233390075
ENSG00000224245	RP11-533K9.3	0.101589	0.010802	3.233373838
ENSG00000258355	RP11-651L5.2	0.221856	0.023591	3.233315091
ENSG00000222987	RN7SKP68	0.165785	0.017629	3.233290934
ENSG00000225684	FAM225B	0.034356306	0.003653633	3.233171284
ENSG00000213218	CSH2	0.126196344	0.013428638	3.232285257
ENSG00000236009	RP11-293F5.1	0.16686757	0.017779277	3.230435018
ENSG00000267774	RP11-2N1.2	0.469884	0.050065	3.230430358
ENSG00000250825	PGAM1P12	0.16285	0.017365	3.229289404
ENSG00000236137	RP11-27K13.3	0.520993664	0.055621643	3.227547552
ENSG00000272606	RP11-554J4.1	1.090164	0.116388	3.22753096
ENSG00000279255	LA16c-380A1.2	0.356776	0.038167	3.224620872
ENSG00000272601	RP11-155G14.5	3.006774	0.32196	3.223263068
ENSG00000214960	ISPD	0.320430525	0.034313768	3.223152143
ENSG00000225720	RP4-742C19.12	2.992863513	0.320535625	3.222969872
ENSG00000107242	PIP5K1B	0.102340828	0.011005229	3.217120767
ENSG00000223134	AL122015.1	6.252851	0.673293	3.215207769
ENSG00000225891	RP3-476K8.3	0.264761	0.028646	3.208285018
ENSG00000248878	RP11-5N11.1	0.68654	0.074341	3.207113779
ENSG00000263823	RP11-326K13.4	0.631859	0.06842	3.207112646
ENSG00000273472	RP11-102N12.3	0.358537	0.038824	3.207101338
ENSG00000221381	SNORD88B	4.049945	0.439296	3.204637048
ENSG00000271857	RP1-244F24.1	0.538061	0.058382	3.204174201
ENSG00000273320	RP11-22N19.2	0.538407	0.058785	3.195177188
ENSG00000279246	RP11-286N22.14	0.289808	0.031768	3.189451249
ENSG00000154027	AK5	0.035633538	0.003910829	3.187689504
ENSG00000205861	C1QTNF9B-AS1	0.270598433	0.029766217	3.184405713
ENSG00000256678	RP11-611O2.2	0.250683	0.027695	3.17816672
ENSG00000224376	AC017104.6	0.612539576	0.067776945	3.175936547
ENSG00000173728	C1orf100	0.042450407	0.004701855	3.174476447
ENSG00000265784	RP1-56K13.3	9.075144	1.007237	3.17151735
ENSG00000239462	CTD-2021J15.1	1.414786	0.157065	3.171150213
ENSG00000196350	ZNF729	0.012472	0.001396	3.159321986
ENSG00000205364	MT1M	0.102657395	0.011490714	3.15929715
ENSG00000215583	ASS1P6	0.122497	0.013742	3.156082524
ENSG00000168874	ATOH8	0.238182285	0.026721663	3.155984439
ENSG00000279365	KB-176G8.1	0.337874	0.0381	3.148622434
ENSG00000234927	HMGN1P18	0.207627	0.023446	3.146580347
ENSG00000158458	NRG2	0.783132115	0.088605277	3.14379118
ENSG00000207088	SNORA7B	1.713627	0.194678	3.137891353
ENSG00000136881	BAAT	0.140189842	0.015969254	3.134012992
ENSG00000279923	CTD-2008E3.1	0.349204	0.039874	3.130549841
ENSG00000278112	RP11-972P1.11	1.013564	0.115889	3.128621649
ENSG00000273270	RP11-212P7.2	0.395971	0.04542	3.123995163
ENSG00000134508	CABLES1	2.28383699	0.262153235	3.122977424
ENSG00000183150	GPR19	0.536321832	0.061898576	3.115120846

ENSG00000259305	ZHX1-C8orf76	2.0963525	0.242266116	3.113216779
ENSG00000227848	SUCLA2-AS1	0.47454	0.054905	3.111520259
ENSG00000162194	LBHD1	1.2255808	0.142092686	3.108561401
ENSG00000184619	KRBA2	0.813903569	0.094465727	3.106994975
ENSG00000259495	RP11-210M15.2	0.279925379	0.032542145	3.104661045
ENSG00000227331	AC005042.2	0.604392	0.070357	3.102718691
ENSG00000215190	LINC00680	1.633925389	0.190670662	3.099187328
ENSG00000267140	RP11-322E11.6	0.345875	0.040442	3.096324488
ENSG00000184343	SRPK3	0.099772935	0.011709224	3.091003065
ENSG00000143851	PTPN7	0.152705378	0.017960237	3.087872607
ENSG00000215014	RP5-832C2.5	0.218508	0.025759	3.084537608
ENSG00000173928	SWSAP1	0.817697	0.096415	3.084236828
ENSG00000267150	RP11-411B10.2	0.422181	0.049881	3.081299359
ENSG00000178636	RP11-455G16.1	0.186054631	0.021982727	3.081284054
ENSG00000268678	CTC-444N24.13	0.438146	0.05179	3.080666224
ENSG00000180884	ZNF792	0.750676577	0.08896486	3.076883966
ENSG00000251034	RP11-582J16.4	0.559809	0.06639	3.075896824
ENSG00000254590	RP11-334E6.2	0.175802	0.020863	3.074932953
ENSG00000213160	KLHL23	13.01495522	1.546981278	3.072642706
ENSG00000235377	RP11-388B24.4	0.116184	0.013813	3.072312812
ENSG00000279794	CTD-2621I17.3	0.675092	0.080298	3.071648163
ENSG00000242849	ALDOAP1	0.202547	0.024098	3.071271396
ENSG00000259205	PRKXP1	0.770764	0.091859	3.068796206
ENSG00000242622	RP11-18H7.1	0.206958197	0.024667267	3.068669598
ENSG00000229214	LINC00242	0.187756	0.022382	3.068448149
ENSG00000213638	ADAT3	2.018031528	0.240965767	3.066048608
ENSG00000276513	MIR378D2	3.323834	0.39762	3.063386102
ENSG00000277726	RP4-635E18.9	0.079535574	0.009541111	3.059371085
ENSG00000220586	TUBBP9	0.20516	0.024623	3.058671025
ENSG00000176945	MUC20	0.761741148	0.091453663	3.058187962
ENSG00000106038	EVX1	0.134185025	0.016140134	3.0554992
ENSG00000238198	RP11-31F15.2	0.18297	0.022009	3.05544162
ENSG00000173432	SAA1	0.588134118	0.070745294	3.055439094
ENSG00000185313	SCN10A	0.034972	0.004216	3.052253532
ENSG00000188660	LINC00319	0.048868741	0.005895946	3.051116602
ENSG00000271717	CTD-3020H12.4	0.360111	0.043545	3.047862691
ENSG00000276648	MIR7706	0.546944	0.066227	3.045901715
ENSG00000215873	FEN1P1	0.195139	0.023633	3.045627457
ENSG00000268593	CTD-2611O12.6	0.257426	0.031208	3.044169964
ENSG00000137266	SLC22A23	4.661379765	0.565195809	3.043934382
ENSG00000225411	RP11-764K9.1	0.13403	0.01627	3.0422698
ENSG00000237857	RP11-435O5.2	1.587063	0.192737	3.041653939
ENSG00000147434	CHRNA6	0.283889676	0.034553159	3.03844088
ENSG00000128268	MGAT3	0.054654202	0.006661273	3.036462664
ENSG00000204291	COL15A1	0.666248568	0.08134809	3.033880155
ENSG00000148735	PLEKHS1	0.451988753	0.055302789	3.030862726
ENSG00000119737	GPR75	1.647293	0.201641	3.030236267
ENSG00000101222	SPEF1	0.229554644	0.028115	3.029425668

ENSG00000231784	DBIL5P	0.00494322	0.000606462	3.026960877
ENSG00000214273	AGGF1P1	0.460841	0.056566	3.026262015
ENSG00000249459	ZNF286B	3.801351241	0.466715415	3.02589731
ENSG00000259642	ST20-AS1	0.208743	0.025631	3.025766147
ENSG00000234353	AP000347.4	1.934131876	0.238943	3.016947758
ENSG00000183840	GPR39	1.256852294	0.15531465	3.016549289
ENSG00000270332	SMC2-AS1	0.057316604	0.007103408	3.012369911
ENSG00000164404	GDF9	0.225687338	0.028059201	3.007779633
ENSG00000263853	AC139530.1	0.970578	0.120827	3.005901284
ENSG00000198393	ZNF26	0.15848218	0.019732078	3.00570581
ENSG00000150773	PIH1D2	0.74072462	0.092315891	3.004286376
ENSG00000215068	AC025171.1	0.155932763	0.019517689	2.998069952
ENSG00000234996	RP11-480I12.9	0.775727467	0.097157188	2.997157241
ENSG00000257831	RP11-596D21.1	0.885357	0.111016	2.995491689
ENSG00000261732	LA16c-431H6.6	2.599647	0.326427	2.993483438
ENSG00000260212	RP11-325O24.5	0.463356	0.058194	2.993178734
ENSG00000225471	RP11-262D11.2	4.098058	0.514741	2.993021798
ENSG00000169955	ZNF747	0.814494829	0.102606	2.988790443
ENSG00000170122	FOXD4	0.669686	0.084535	2.98586412
ENSG00000260027	HOXB7	19.60143297	2.480760905	2.982104526
ENSG00000176927	EFCAB5	0.061532665	0.007812077	2.977574338
ENSG00000241155	ARHGAP31-AS1	0.244694	0.031223	2.970297659
ENSG00000268322	BNIP3P25	0.091048	0.011618	2.970265592
ENSG00000271788	CTD-2201E18.5	0.062757	0.008008	2.970262509
ENSG00000101470	TNNC2	0.116201301	0.014828014	2.970228968
ENSG00000276017	AC007325.1	0.018021	0.002301	2.969346181
ENSG00000137502	RAB30	1.417282179	0.181080416	2.968424591
ENSG00000227155	RP11-165F24.3	0.105470103	0.013476162	2.968352566
ENSG00000254503	CTD-2521M24.4	0.336611	0.043021	2.967969358
ENSG00000267724	RP11-49K24.8	0.634723	0.081395	2.963115042
ENSG00000213186	TRIM59	7.309857371	0.938648624	2.961186154
ENSG00000268970	CTD-3099C6.11	0.664856	0.085474	2.95948436
ENSG00000253669	KB-1732A1.1	0.591540403	0.076139261	2.957764233
ENSG00000253584	CTD-2374C24.1	0.699367375	0.090051025	2.957235895
ENSG00000268362	CTD-2017D11.1	0.49386261	0.063729723	2.954071463
ENSG00000197557	TTC30A	0.420799	0.054347	2.952858972
ENSG00000263235	RP11-461A8.4	1.533558	0.198645	2.948618346
ENSG00000283033	RP11-219A15.5	1.22131	0.158247	2.948179385
ENSG00000260669	AL136419.6	2.925775739	0.37970887	2.945853586
ENSG00000256518	RP11-807H22.5	0.281169794	0.036518866	2.944727724
ENSG00000217929	CICP18	0.080519	0.01046	2.944446403
ENSG00000225335	XXbac-B476C20.9	0.560684769	0.073129316	2.9386681
ENSG00000214178	PPIAP23	0.378905	0.049531	2.935432524
ENSG00000145703	IQGAP2	10.82732818	1.415647022	2.935143785
ENSG00000205740	RP11-363N22.3	0.161588049	0.021150846	2.933533217
ENSG00000168685	IL7R	0.096585167	0.012681729	2.929050227
ENSG00000254389	RHPN1-AS1	0.644296	0.084651	2.928124622
ENSG00000143494	VASH2	1.966953311	0.258726029	2.926465605

ENSG00000152443	ZNF776	3.505017821	0.462543959	2.921759392
ENSG00000225805	CTD-2313N18.7	0.307979	0.040654	2.921362769
ENSG00000187634	SAMD11	0.509117487	0.067355895	2.918122488
ENSG00000214447	FAM187A	0.827522556	0.109524033	2.917551157
ENSG00000279602	CTD-3014M21.1	0.618769	0.081938	2.916796336
ENSG00000232656	IDI2-AS1	0.320231392	0.042413588	2.916516309
ENSG00000158258	CLSTN2	0.135533146	0.017968373	2.915114053
ENSG00000187391	MAGI2	0.03994817	0.005296348	2.915059691
ENSG00000185518	SV2B	0.032694066	0.004345322	2.911493774
ENSG00000120327	PCDHB14	0.126503	0.016814	2.911436715
ENSG00000238961	SNORA47	0.649111	0.086279	2.911383844
ENSG00000228393	LINC01004	0.360008504	0.047852032	2.911378897
ENSG00000275411	MIR6882	0.123385	0.016401	2.911311329
ENSG00000154451	GBP5	0.050002139	0.006646578	2.91130622
ENSG00000270344	RP11-734K2.4	0.220396	0.029345	2.908911427
ENSG00000276261	RP11-1060J15.9	0.781942	0.1042	2.907706323
ENSG00000116745	RPE65	2.226139	0.296664	2.907641905
ENSG00000257167	TMPO-AS1	0.594945	0.079319	2.90701791
ENSG00000267546	RP11-666A8.8	0.54319827	0.072450289	2.906415533
ENSG00000155974	GRIP1	0.127200722	0.016971219	2.905944803
ENSG00000213240	RP11-458D21.5	0.531544	0.070956	2.905192532
ENSG00000198881	ASB12	0.3143	0.041987	2.904127657
ENSG00000136040	PLXNC1	0.365381385	0.048823937	2.903742609
ENSG00000198846	TOX	0.19602	0.026202	2.903252019
ENSG00000228775	WEE2-AS1	0.341751791	0.045718093	2.902111766
ENSG00000272031	ANKRD34A	0.761039097	0.101852799	2.901484948
ENSG00000177311	ZBTB38	3.374587126	0.451699993	2.901274205
ENSG00000263843	RP11-649A18.12	1.54705895	0.207138748	2.900858813
ENSG00000259459	RP11-321G12.1	0.181217343	0.024269647	2.900496028
ENSG00000234390	USP27X-AS1	0.185332	0.024884	2.896821687
ENSG00000272301	RP11-111M22.4	1.172411	0.157523	2.895844011
ENSG00000244349	HCG16	0.889479	0.119509	2.895841276
ENSG00000196705	ZNF431	1.26970435	0.170767532	2.894386996
ENSG00000180574	EIF2S3L	15.71646289	2.115251577	2.893375399
ENSG00000105810	CDK6	7.800091462	1.050517232	2.892391213
ENSG00000175040	CHST2	2.593574	0.349644	2.89098288
ENSG00000134138	MEIS2	2.097066012	0.282967335	2.88966485
ENSG00000256196	RP11-881M11.4	0.464193	0.062639	2.889591681
ENSG00000269936	RP11-394O4.5	0.157341	0.021233	2.88951453
ENSG00000257731	RP11-536C10.11	0.062628	0.008454	2.88910179
ENSG00000281593	GS1-114I9.3	0.601649	0.081262	2.888269292
ENSG00000272502	RP11-713M15.2	0.702212	0.094865	2.887958836
ENSG00000054219	LY75	0.045528372	0.006151065	2.887857647
ENSG00000269392	CTB-191K22.6	1.317609	0.178028	2.887746245
ENSG00000265148	BZRAP1-AS1	0.266068845	0.035953132	2.887610225
ENSG00000099957	P2RX6	0.165990752	0.022471757	2.884918024
ENSG00000181449	SOX2	0.477164	0.064609	2.884678151
ENSG00000136490	LIMD2	1.22360126	0.165762246	2.883946136

ENSG00000174327	SLC16A13	1.482935417	0.201098633	2.882480586
ENSG00000230432	AC114803.3	0.692865	0.093972	2.882271422
ENSG00000235257	ITGA9-AS1	0.119975314	0.016274739	2.882031294
ENSG00000225886	RP11-288L9.4	0.130377339	0.017701435	2.880754872
ENSG00000184659	FOXD4L4	0.148702	0.020225	2.878212444
ENSG00000164512	ANKRD55	0.017084822	0.002325348	2.87719638
ENSG00000244615	PSPC1P2	7.204971	0.981074	2.876558759
ENSG00000239445	ST3GAL6-AS1	1.413706612	0.192600292	2.875800953
ENSG00000217644	RP4-803A2.1	0.188683	0.025722	2.874889716
ENSG00000280046	RP11-1099M24.6	2.940328	0.400974	2.874396502
ENSG00000158234	FAIM	3.451290655	0.470838183	2.873832751
ENSG00000267811	RP11-727F15.11	0.53718	0.073346	2.872615398
ENSG00000260757	RP11-120K18.2	0.671513	0.091886	2.869498359
ENSG00000207603	MIR7-1	0.610754	0.083784	2.865844739
ENSG00000270369	RP11-144G6.13	0.441214	0.060537	2.865589482
ENSG00000261079	RP11-252A24.3	0.083347	0.011442	2.864791022
ENSG00000265055	AC145343.2	0.447887	0.061614	2.861804688
ENSG00000234225	RP4-704D21.2	0.55626905	0.076528133	2.86172072
ENSG00000154721	JAM2	0.128333679	0.017696685	2.858348783
ENSG00000264247	LINC00909	2.716731036	0.376038119	2.85292092
ENSG00000224618	AC096921.2	1.042354	0.144321	2.852492178
ENSG00000179935	LINC00652	0.329723542	0.045719415	2.850378063
ENSG00000255154	RP11-80H18.3	1.366111751	0.189941349	2.846449596
ENSG00000245281	CTD-2547L16.1	0.423896613	0.059019265	2.844454579
ENSG00000243053	RPL31P58	0.225609	0.031416	2.844253213
ENSG00000198796	ALPK2	0.164599121	0.022954355	2.84211684
ENSG00000167716	WDR81	1.726332584	0.2410927	2.840050558
ENSG00000152782	PANK1	1.551281563	0.216760301	2.839288101
ENSG00000168806	LCMT2	0.834735	0.116689	2.838649696
ENSG00000198890	PRMT6	4.143293	0.579272	2.83846501
ENSG00000197409	HIST1H3D	0.941146	0.131681	2.837371349
ENSG00000154537	FAM27C	1.562498	0.219645	2.83060878
ENSG00000228748	RP13-39P12.3	1.615157695	0.227110879	2.830206309
ENSG00000231870	KRT17P3	0.152446	0.021443	2.82971963
ENSG00000067840	PDZD4	0.219483413	0.03091884	2.827553818
ENSG00000261067	RP11-264B17.3	1.137791282	0.160861274	2.822346976
ENSG00000233435	AGGF1P2	0.302897	0.04283	2.822133707
ENSG00000237719	RP1-179N16.3	0.583445	0.082501	2.822113151
ENSG00000140527	WDR93	0.265888762	0.037638568	2.820539172
ENSG00000204532	ZSCAN5CP	0.251119	0.035566	2.819800556
ENSG00000205181	LINC00654	0.060649235	0.008598409	2.818347733
ENSG00000199366	Y_RNA	1.440458	0.204228	2.818275015
ENSG00000277159	RP11-88E10.4	0.212659	0.030151	2.818264156
ENSG00000274301	MIR3179-1	0.045443	0.006443	2.81825358
ENSG00000162086	ZNF75A	0.813585594	0.115479349	2.816659248
ENSG00000177946	CENPBD1	2.49844	0.355018	2.815063494
ENSG00000273514	FOXD4L6	0.202692	0.028826	2.813846586
ENSG00000100413	POLR3H	10.03717399	1.430846105	2.810412713

ENSG00000259890	DNM1P50	0.175331	0.025036	2.808005113
ENSG00000274966	RP11-382B18.6	0.186335	0.026608	2.807966708
ENSG00000272269	RP11-500C11.3	0.69627	0.099487	2.807066938
ENSG00000108375	RNF43	14.86939896	2.127320584	2.805236966
ENSG00000232725	U52111.14	0.3474394	0.049788795	2.802868367
ENSG00000230583	GTF2IRD1P1	0.046456949	0.006666814	2.800824922
ENSG00000112981	NME5	1.447436106	0.207796693	2.800255064
ENSG00000261857	MIA	0.709435445	0.101934233	2.799032771
ENSG00000268635	RP11-739B23.1	0.197518	0.028392	2.798427749
ENSG00000182141	ZNF708	0.918994051	0.132412795	2.795012986
ENSG00000141905	NFIC	12.21583238	1.764619614	2.79132304
ENSG00000261342	AC006538.1	1.63767	0.236969	2.78887443
ENSG00000273795	AC145212.1	0.539622	0.078246	2.78586026
ENSG00000170468	C14orf169	1.834694	0.266035	2.785851496
ENSG00000204802	RP11-111F5.4	0.460436847	0.066766674	2.785803203
ENSG00000130287	NCAN	0.107910238	0.015675668	2.783232901
ENSG00000205363	C15orf59	0.33982	0.049402	2.78212941
ENSG00000261118	RP11-104N10.1	0.711945565	0.103580654	2.78101237
ENSG00000180767	CHST13	0.830157	0.121007	2.778293699
ENSG00000277713	MIR6856	0.94196	0.137321	2.778113529
ENSG00000254721	RP11-805J14.5	0.849170488	0.123994016	2.775783737
ENSG00000207110	RNU1-106P	2.259295	0.330003	2.775321613
ENSG00000230061	TRPM2-AS	1.097961068	0.160644517	2.772883258
ENSG00000204103	MAFB	0.642315	0.094427	2.766009647
ENSG00000158296	SLC13A3	0.015802217	0.002323892	2.765511909
ENSG00000275506	CTD-2062A1.2	0.182776	0.026911	2.763808738
ENSG00000273143	RP11-525A16.4	1.697080072	0.24991972	2.76351799
ENSG00000281193	AL121895.1	0.355466	0.052379	2.762651152
ENSG00000004139	SARM1	0.282950392	0.041812293	2.75855007
ENSG00000261527	RP11-343C2.10	0.486404	0.071961	2.756867953
ENSG00000159917	ZNF235	1.098000946	0.162660321	2.754945025
ENSG00000166189	HPS6	8.254445	1.223382	2.754296262
ENSG00000187686	KRT18P59	0.108989229	0.016213207	2.748944202
ENSG00000239373	RN7SL425P	0.173116	0.025772	2.747862665
ENSG00000246627	CACNA1C-AS1	0.038572061	0.005742761	2.74773974
ENSG00000259349	RP11-15E18.1	0.506191981	0.075377305	2.747482531
ENSG00000279452	AC006277.3	0.898429	0.133788	2.747455776
ENSG00000157600	TMEM164	6.261366705	0.934377258	2.744400531
ENSG00000165495	PKNOX2	0.110672597	0.016555684	2.740899562
ENSG00000234393	RP11-211N11.5	0.146363	0.021898	2.740679876
ENSG00000231890	DARS-AS1	0.141902198	0.021248491	2.73946467
ENSG00000081041	CXCL2	0.954587449	0.142986704	2.738996368
ENSG00000196810	CTBP1-AS2	0.18352848	0.027491087	2.738968078
ENSG00000184995	IFNE	0.978587	0.146767	2.737172497
ENSG00000261533	RP11-15N24.4	0.656058	0.098446	2.736418871
ENSG00000227233	CICP17	0.096059	0.01442	2.735849625
ENSG00000226659	RP11-137H2.4	0.602178	0.090503	2.734152481
ENSG00000163009	C2orf48	1.254563	0.188865451	2.731754202

ENSG00000139679	LPAR6	0.243009744	0.036595187	2.73128835
ENSG00000213455	AC091654.7	0.1681	0.025372	2.728010572
ENSG00000162931	TRIM17	0.308076468	0.046676874	2.722508635
ENSG00000144476	ACKR3	0.157782802	0.023967625	2.718781078
ENSG00000263369	RP11-640N20.9	0.398516	0.060537	2.718748561
ENSG00000219392	ZNF602P	0.385577	0.058572	2.718735933
ENSG00000183346	C10orf107	0.211438	0.032119	2.718735802
ENSG00000271751	RP11-110I1.14	0.85849	0.130411	2.718735765
ENSG00000279368	RP1-80N2.4	0.387886	0.058923	2.718729915
ENSG00000101445	PPP1R16B	0.055668085	0.008456431	2.718729656
ENSG00000280132	RP11-452L6.6	0.18702	0.02841	2.718721824
ENSG00000173013	CCDC96	0.170384	0.025883	2.718713115
ENSG00000255176	AP002954.3	0.430746	0.065506	2.717138438
ENSG00000227684	CROCCP4	0.099522339	0.015151748	2.715536142
ENSG00000198739	LRRTM3	0.07639902	0.011638203	2.714685819
ENSG00000177721	ANXA2R	0.148891454	0.022682521	2.714608056
ENSG00000259002	RP11-671J11.7	0.134160009	0.02043852	2.714592081
ENSG00000253741	CTD-2292P10.4	0.808752	0.123399	2.712366672
ENSG00000244414	CFHR1	0.391973045	0.059860224	2.711084865
ENSG00000174007	CEP19	4.993656	0.762753	2.710808587
ENSG00000132744	ACY3	0.27249	0.041632	2.710438514
ENSG00000227470	AC073415.2	2.175563	0.332506	2.709936516
ENSG00000227582	ADGRF5P1	0.237788979	0.036346679	2.709786397
ENSG00000271798	SNORA51	0.363636	0.05559	2.709597746
ENSG00000274177	LLNLR-284B4.1	0.326874	0.049981	2.709282956
ENSG00000264091	AP002495.1	0.673395	0.103246	2.70536712
ENSG00000106004	HOXA5	3.531717021	0.541522718	2.705275984
ENSG00000267077	RP11-127I20.5	0.490193	0.075167	2.705178552
ENSG00000274877	RP11-65I12.1	3.324919	0.509883	2.705081052
ENSG00000254757	RP13-726E6.1	0.490671	0.075381	2.702483168
ENSG00000197619	ZNF615	0.035946679	0.005523766	2.702134341
ENSG00000261360	CTD-2165H16.4	0.251478	0.038699	2.700064002
ENSG00000221160	MIR1281	0.367584	0.056596	2.699301974
ENSG00000178597	PSAPL1	0.130649	0.020134	2.697990358
ENSG00000273056	RP11-77E14.2	0.601342	0.092672	2.697980313
ENSG00000264379	SNORD39	4.607446	0.712113	2.693789162
ENSG00000265411	RN7SL656P	0.248745	0.03852	2.690987918
ENSG00000267390	RP11-635N19.1	0.444775	0.068943	2.689599716
ENSG00000263528	IKBKE	1.439965282	0.223616077	2.686938208
ENSG00000152315	KCNK13	1.652411	0.256654	2.686675919
ENSG00000060709	RIMBP2	0.07578172	0.011777577	2.685807081
ENSG00000171786	NHLH1	0.406296	0.063348	2.681160184
ENSG00000275709	RP11-96O20.5	0.633417	0.09876	2.681156846
ENSG00000275580	RP11-295H24.4	0.536495	0.083649	2.681144527
ENSG00000247796	CTD-2366F13.1	1.5038124	0.234511236	2.68089565
ENSG00000277350	RP11-395E19.6	0.06924649	0.010798876	2.680859758
ENSG00000228175	GEMIN8P4	0.9509	0.148479	2.679034731
ENSG00000170161	RP11-262H14.4	0.435176	0.06809	2.676084156

ENSG00000221909	FAM200A	4.310555259	0.675043862	2.67482057
ENSG00000255870	RP11-667M19.5	0.351435	0.055049	2.674469617
ENSG00000260852	FBXL19-AS1	1.632545	0.255878	2.673594742
ENSG00000172421	EFCAB3	0.147150294	0.023086093	2.672194501
ENSG00000228554	AC004837.5	0.278502	0.043694	2.672180598
ENSG00000128422	KRT17	0.339441809	0.053331747	2.670097782
ENSG00000216101	MIR877	2.542063	0.399824	2.668562806
ENSG00000224769	MUC20P1	1.329967	0.209395	2.66709155
ENSG00000271643	RP11-10C24.3	0.538379	0.084845	2.665720589
ENSG00000227449	RP11-111F5.5	0.335702938	0.052963352	2.664118825
ENSG00000259081	RP11-488C13.6	0.718464469	0.113460153	2.662731095
ENSG00000279508	AL589743.1	0.847468	0.133922	2.661765915
ENSG00000164056	SPRY1	3.61860125	0.57183424	2.661763227
ENSG00000273496	AC011841.1	0.135424	0.021447	2.658635674
ENSG00000225614	ZNF469	3.074534731	0.487398364	2.657194789
ENSG00000145861	C1QTNF2	0.420832	0.06681	2.655108447
ENSG00000233555	RP11-452D2.1	0.806193	0.128082	2.654057516
ENSG00000268191	CTD-2396E7.10	3.558975	0.565892	2.652863151
ENSG00000281189	GHET1	0.356754	0.05679	2.651220789
ENSG00000176022	B3GALT6	4.02771	0.641573	2.650274478
ENSG00000254604	AP000487.6	0.188854	0.030122	2.648381873
ENSG00000256651	RP11-144O23.8	0.359458	0.057392	2.646901657
ENSG00000189398	OR7E12P	0.139054	0.022249	2.643832846
ENSG00000213959	RP5-1100H13.3	0.897001	0.143938	2.639662076
ENSG00000199645	RNU6-1330P	1.485188	0.238351	2.63948598
ENSG00000260405	CTD-2576D5.2	1.02178	0.164001	2.639308085
ENSG00000250326	RP11-284M14.1	0.331626971	0.053268204	2.638214809
ENSG00000261071	RP1-223E5.4	0.625366	0.100473	2.637892925
ENSG00000237945	LINC00649	0.023431037	0.00377536	2.633734509
ENSG00000104361	NIPAL2	0.964085302	0.155604867	2.631273614
ENSG00000264624	MIR3615	0.37912	0.06125	2.629872817
ENSG00000170439	METTL7B	12.41583608	2.007011661	2.629060512
ENSG00000238061	RP4-635A23.4	0.096248	0.015574	2.627617031
ENSG00000159184	HOXB13	4.222316	0.684038	2.625886177
ENSG00000139445	FOXN4	0.192382267	0.03118463	2.625068789
ENSG00000254690	GS1-393G12.12	0.411782	0.066791	2.624155148
ENSG00000179886	TIGD5	1.732171	0.281145	2.623195065
ENSG00000234429	AC105342.1	0.243333	0.039516	2.622423164
ENSG00000226957	RP4-533D7.4	0.078423	0.012787	2.616599003
ENSG00000230438	SERPINB9P1	1.135054966	0.185344349	2.614482126
ENSG00000229693	RP11-25C15.1	0.26466	0.043296	2.611834517
ENSG00000261594	TPBGL	0.46869	0.076734	2.610696147
ENSG00000260328	RP11-416I2.1	1.825779	0.299008	2.610256156
ENSG00000106538	RARRES2	0.285241932	0.046767661	2.608602897
ENSG00000251359	WWC2-AS2	0.30216	0.049584	2.607366124
ENSG00000205978	NYNRIN	0.08291777	0.013607277	2.607302926
ENSG00000198464	ZNF480	4.100054732	0.673484754	2.605925977
ENSG00000182257	PRR34	0.233715	0.038424	2.604670712

ENSG00000253923	KB-1980E6.2	0.33722	0.055508	2.602922485
ENSG00000234859	AC003958.2	0.061428906	0.010113814	2.602590589
ENSG00000273192	CITF22-1A6.3	0.951765	0.156819	2.601505037
ENSG00000231023	LINC00326	2.336714	0.385192	2.600831522
ENSG00000144043	TEX261	16.34508914	2.696421317	2.599739404
ENSG00000223244	MIR1909	0.86172	0.142281	2.598476149
ENSG00000075407	ZNF37A	6.387903271	1.057377669	2.594851703
ENSG00000197852	FAM212B	0.683048741	0.113078365	2.594665596
ENSG00000266868	MIR4517	0.400901	0.066399	2.594012597
ENSG00000170324	FRMPD2	0.041498206	0.006875318	2.593550524
ENSG00000215271	HOMEZ	4.166572444	0.691102567	2.591889321
ENSG00000157087	ATP2B2	0.064190301	0.010649021	2.59163459
ENSG00000127774	EMC6	22.10665797	3.669185969	2.590949006
ENSG00000279645	RP11-759A24.1	0.077544	0.012892	2.588539063
ENSG00000182040	USH1G	1.271117	0.211432	2.587831181
ENSG00000253559	OSGEPL1-AS1	0.369805952	0.061672381	2.584071986
ENSG00000228150	RP11-84A14.4	0.619461	0.103322	2.583865981
ENSG00000177045	SIX5	1.766782748	0.295022256	2.58222895
ENSG00000273382	RP5-1065J22.8	0.224169	0.03748	2.580393926
ENSG00000248885	RP11-950K24.2	0.053392	0.008942	2.577954141
ENSG00000160216	AGPAT3	3.302703343	0.553236677	2.577678681
ENSG00000205634	LINC00898	0.074799357	0.012545299	2.575879005
ENSG00000117586	TNFSF4	1.71874812	0.288313691	2.575646884
ENSG00000282785	RP11-1223D19.2	39.187805	6.589716	2.572116571
ENSG00000277534	RP11-9E17.1	1.369019	0.230219	2.57206366
ENSG00000265087	MIR4761	0.538058	0.090509	2.571628534
ENSG00000138161	CUZD1	0.514511445	0.086556163	2.571494717
ENSG00000128276	RFPL3	0.055608457	0.009373924	2.568579296
ENSG00000207561	MIR635	4.975364	0.83942	2.567337335
ENSG00000268798	CTB-25B13.5	0.291497	0.049201	2.566721492
ENSG00000232485	AC098820.3	0.446759647	0.075429196	2.566303933
ENSG00000248559	RP11-215P8.3	0.616111	0.104078	2.56552515
ENSG00000164011	ZNF691	4.558640524	0.770340037	2.565036335
ENSG00000120337	TNFSF18	3.172902	0.536354	2.564545546
ENSG00000166086	JAM3	0.23472933	0.039714228	2.563270261
ENSG00000126231	PROZ	0.576000065	0.097456052	2.563245295
ENSG00000227199	ST7-AS1	0.169196	0.02865	2.562088418
ENSG00000042832	TG	0.028620845	0.004861862	2.557485374
ENSG00000254837	AP001372.2	2.192231716	0.372526312	2.556986065
ENSG00000198838	RYR3	0.078472811	0.013339148	2.556526338
ENSG00000105855	ITGB8	3.839951093	0.653045729	2.555832014
ENSG00000105549	THEG	0.037285881	0.006343297	2.555324532
ENSG00000226842	RP11-166N17.3	0.109219	0.018582	2.555246159
ENSG00000266668	MIR5692C2	0.66099	0.112458	2.555242151
ENSG00000266947	RP11-799D4.4	0.339031962	0.05769768	2.554836068
ENSG00000184371	CSF1	1.402458305	0.238737178	2.554462722
ENSG00000132692	BCAN	0.621041046	0.105729725	2.554307593
ENSG00000119922	IFIT2	1.847084	0.314644524	2.553454738

ENSG00000182010	RTKN2		2.222925963	0.378725276	2.553236283
ENSG00000279691	RP11-485M7.2		0.656887	0.111973	2.552494317
ENSG00000083817	ZNF416		1.73377	0.295856	2.550945466
ENSG00000248206	RP11-739P1.2		0.489323	0.08353	2.550420753
ENSG00000267767	CTC-523E23.4		0.27981663	0.047767334	2.550385453
ENSG00000228492	RAB11FIP1P1		2.496023	0.42633	2.549588745
ENSG00000251602	RP11-521B24.3		0.972915	0.166232	2.549115637
ENSG00000228146	CASP16P		0.092125535	0.015750898	2.548167049
ENSG00000246465	RP11-57A19.2		0.366798	0.062824	2.545598065
ENSG00000280179	CH507-42P11.6		0.036558937	0.00626664	2.54446002
ENSG00000278883	AL591806.1		0.357404	0.061337	2.54272627
ENSG00000254008	LINC00051		0.096342	0.016557	2.540723582
ENSG00000185614	FAM212A		0.888726	0.152994	2.53826362
ENSG00000259898	CYP4F33P		0.76526	0.131903	2.536472615
ENSG00000232457	SLC16A6P1		3.017945	0.520246	2.536300641
ENSG00000184845	DRD1		2.167672	0.373798	2.535815718
ENSG00000249306	LINC01411		0.120543565	0.020793572	2.535345147
ENSG00000144712	CAND2		0.234784287	0.040506673	2.535104358
ENSG00000154997		14-Sep	0.05604417	0.009701425	2.530295718
ENSG00000213269	AC004386.4		0.838082	0.14508	2.530242757
ENSG00000164855	TMEM184A		2.157290335	0.37361379	2.529600741
ENSG00000280594	AP000432.1		0.959092087	0.166265484	2.528180639
ENSG00000203506	RBMS3-AS2		0.066299786	0.011514662	2.52553219
ENSG00000205930	C21orf62-AS1		0.088893457	0.015445359	2.524903804
ENSG00000139200	PIANP		0.190693117	0.033142064	2.524517427
ENSG00000184492	FOXD4L1		0.343578	0.059748	2.523675334
ENSG00000271122	RP11-379H18.1		2.512576	0.437088	2.52317156
ENSG00000255422	AP002954.4		0.800606	0.139492	2.520910043
ENSG00000238795	SCARNA12		0.896943	0.15633	2.520421645
ENSG00000227267	RP11-305O4.2		0.267183	0.046575	2.520200542
ENSG00000187559	FOXD4L3		0.215508	0.037602	2.518860121
ENSG00000137834	SMAD6		2.60317276	0.454469532	2.518015576
ENSG00000280303	ERICD		0.927464	0.162118	2.516247001
ENSG00000280212	RP11-49K24.3		0.647382	0.113223	2.515450198
ENSG00000163817	SLC6A20		0.941901176	0.164787122	2.514972202
ENSG00000154928	EPHB1		0.177350624	0.03104366	2.514233826
ENSG00000074047	GLI2		2.725681749	0.479037065	2.508407932
ENSG00000114450	GNB4		0.172442472	0.030310679	2.508217062
ENSG00000185674	LYG2		0.249943071	0.043978627	2.506725069
ENSG00000175564	UCP3		0.44570401	0.078448377	2.50627043
ENSG00000196418	ZNF124		1.388707927	0.244437048	2.506208336
ENSG00000275897	RP11-94C24.13		0.238558	0.042068	2.503544932
ENSG00000261792	DNM1P28		0.152116	0.026832	2.503145409
ENSG00000280399	RP11-80P20.3		0.533369	0.094135	2.502330845
ENSG00000198133	TMEM229B		0.542562667	0.095761895	2.502266177
ENSG00000213727	LA16c-60G3.7		0.242857	0.04287	2.502066748
ENSG00000242748	RPL23AP81		0.656093	0.115889	2.501156694
ENSG00000216895	AC009403.2		1.069344887	0.189083939	2.499628496

ENSG00000270781	RP11-501C14.9	1.391393	0.246309	2.497988719
ENSG00000135426	TESPA1	0.030386128	0.00538481	2.496445611
ENSG00000236740	RP11-411K7.1	0.09463112	0.016770449	2.496393355
ENSG00000163530	DPPA2	0.073603	0.013044	2.496378224
ENSG00000274769	RP11-493E12.3	0.220808	0.039132	2.496371691
ENSG00000216588	IGSF23	0.072547225	0.012857	2.49636638
ENSG00000177875	CCDC184	0.260672	0.046197	2.496364552
ENSG00000238161	OR7E117P	0.09154	0.016223	2.496361661
ENSG00000278916	CEP83-AS1	0.246074	0.04361	2.496361336
ENSG00000163016	ALMS1P1	0.184629953	0.0327208	2.496356675
ENSG00000260986	RP11-854K16.3	0.598779	0.106118	2.496354234
ENSG00000138823	MTTP	0.056572393	0.010025993	2.496353012
ENSG00000257660	RP11-579D7.4	0.551221735	0.097689868	2.496351927
ENSG00000272668	RP11-190A12.8	0.417182	0.073935	2.496347525
ENSG00000275966	RP11-1055B8.9	0.62195	0.110225	2.496347126
ENSG00000177464	GPR4	0.09709891	0.017208359	2.496345601
ENSG00000213652	HMGB3P30	0.251962	0.044654	2.496344849
ENSG00000273606	MIR6776	0.103518	0.018346	2.496344199
ENSG00000272010	CTD-3025N20.3	0.490172	0.086871	2.496341526
ENSG00000275202	RP11-156K23.3	0.277364	0.049156	2.496341119
ENSG00000196196	HRCT1	0.321449	0.056969	2.496340875
ENSG00000161544	CYGB	0.103552599	0.018352197	2.496339098
ENSG00000261333	CD24P2	0.464806	0.082376	2.496332713
ENSG00000254510	RP11-867G23.10	0.229607311	0.040692722	2.496325896
ENSG00000261051	RP11-274H2.5	0.291529	0.051667	2.496324381
ENSG00000154548	SRSF12	0.067145471	0.011900124	2.496313533
ENSG00000148848	ADAM12	0.030971081	0.005488974	2.496313325
ENSG00000226777	KIAA0125	0.030895714	0.005476039	2.496202034
ENSG00000150893	FREM2	0.009411396	0.001668118	2.49618723
ENSG00000198756	COLGALT2	0.042656828	0.007572458	2.493943111
ENSG00000258920	FOXN3-AS1	1.130764	0.201005	2.491994564
ENSG00000136546	SCN7A	0.037121587	0.006600699	2.491567694
ENSG00000122641	INHBA	0.040408686	0.007185391	2.491526848
ENSG00000268287	CTB-60B18.18	0.478323	0.085072	2.491228888
ENSG00000179023	KLHDC7A	0.325063	0.057855	2.4902058
ENSG00000232931	LINC00342	3.051181474	0.543334998	2.489454105
ENSG00000182986	ZNF320	2.876787342	0.512682426	2.488321229
ENSG00000165025	SYK	2.154629168	0.384037204	2.488121602
ENSG00000270574	RP11-171I2.2	0.573634	0.102259	2.487902714
ENSG00000147465	STAR	0.080525949	0.014366819	2.486713061
ENSG00000158955	WNT9B	0.045780499	0.008167879	2.486699731
ENSG00000170356	OR2A20P	1.510436718	0.269506643	2.486573004
ENSG00000177710	SLC35G5	0.019264	0.003441	2.4850075
ENSG00000239377	RP4-584D14.6	0.302954	0.054121	2.484838353
ENSG00000185634	SHC4	0.249822518	0.044632014	2.484752702
ENSG00000245522	RP11-540A21.2	0.460232313	0.082249736	2.484279335
ENSG00000108932	SLC16A6	7.709735394	1.378001819	2.484103554
ENSG00000162520	SYNC	0.520940588	0.09312391	2.483895307

ENSG00000174417	TRHR	0.068548701	0.012256103	2.483629055
ENSG00000260589	STAM-AS1	0.349899	0.062567	2.483464706
ENSG00000205913	SRRM2-AS1	0.258284363	0.046193412	2.483201274
ENSG00000121577	POPDC2	0.373613628	0.066937173	2.480667552
ENSG00000235029	MNX1-AS2	0.80563	0.144707	2.476982695
ENSG00000273551	RP11-616M22.11	0.567431	0.101922	2.476979478
ENSG00000272189	RP3-325F22.5	0.159084	0.028575	2.476963339
ENSG00000157388	CACNA1D	0.867887486	0.155906936	2.47682291
ENSG00000008735	MAPK8IP2	3.015026408	0.541997619	2.475812219
ENSG00000233003	CICP26	0.110817	0.019924	2.475599995
ENSG00000264914	RP11-343K8.3	0.655805991	0.117909887	2.475584389
ENSG00000197816	CCDC180	0.361191718	0.064963503	2.475063492
ENSG00000223309	RNU6-722P	1.732429	0.312234	2.472096678
ENSG00000258256	RP11-219B4.5	0.239082328	0.043089852	2.472087451
ENSG00000232491	SAPCD2P3	0.08832	0.015918	2.472081091
ENSG00000229809	ZNF688	0.496398829	0.089514685	2.471303433
ENSG00000276851	RP5-875H18.9	0.689674	0.124394	2.470997679
ENSG00000267254	ZNF790-AS1	0.067591368	0.012218384	2.467785469
ENSG00000262381	MTATP6P24	0.150358	0.02718	2.467784269
ENSG00000235990	RPL23AP20	0.241788	0.043708	2.467773375
ENSG00000231686	RP1-203P18.1	8.596891	1.554596	2.467275305
ENSG00000274767	AC131056.3	0.213614889	0.03862969	2.467230217
ENSG00000170049	KCNAB3	0.632920042	0.114522462	2.466392659
ENSG00000276097	AC006538.8	0.496634	0.089889	2.465966549
ENSG00000270147	RP11-646I6.6	1.067217	0.193287	2.465037039
ENSG00000177133	LINC00982	0.053339583	0.009664646	2.464417737
ENSG00000231119	RP4-569M23.2	0.164985	0.029918	2.463249224
ENSG00000267190	AC002314.4	0.351712	0.063821	2.46229144
ENSG00000260063	RP5-968P14.2	0.447378	0.081181	2.462280296
ENSG00000250379	RP11-23P13.4	0.052470248	0.009543162	2.458960284
ENSG00000129028	THAP10	1.599452	0.29147	2.456158393
ENSG00000176046	NUPR1	0.147484384	0.026897577	2.455014063
ENSG00000248375	RP11-177B4.1	0.790212	0.144129	2.454879108
ENSG00000245975	RP11-30K9.6	0.212513	0.038769	2.454575669
ENSG00000196152	ZNF79	3.053809098	0.557661723	2.453147723
ENSG00000273314	RP5-1136G13.2	0.808596	0.147736	2.452397645
ENSG00000231079	AC105402.4	0.142548541	0.026044602	2.452396993
ENSG00000125798	FOXA2	0.442677026	0.08108722	2.448708042
ENSG00000188958	UTS2B	0.536683196	0.098400908	2.447327186
ENSG00000237714	P4HA2-AS1	0.182936	0.033543	2.44725538
ENSG00000099998	GGT5	0.940155539	0.172445892	2.446755697
ENSG00000196872	KIAA1211L	0.605669621	0.111116575	2.446457018
ENSG00000249572	CTD-2203K17.1	1.09793	0.201567	2.445454707
ENSG00000261662	RP5-1042I8.7	0.632926	0.116286	2.444359416
ENSG00000229590	MSX2P1	0.190285	0.035007	2.442446502
ENSG00000189057	FAM111B	5.156518114	0.95015629	2.440160484
ENSG00000229996	AC093585.6	0.356749	0.065754	2.43975882
ENSG00000046653	GPM6B	0.594998227	0.109763345	2.438489017

ENSG00000134551	PRH2	0.301696989	0.055764762	2.435674625
ENSG00000272742	CTB-43P18.1	0.790456	0.146256	2.434189348
ENSG00000007376	RPUSD1	14.72246421	2.725805445	2.433264668
ENSG00000118308	LRMP	0.10861939	0.020141931	2.431007728
ENSG00000101935	AMMECR1	4.135355629	0.766984592	2.430741902
ENSG00000136279	DBNL	3.180881941	0.590712417	2.428898985
ENSG00000075213	SEMA3A	19.50289925	3.621993084	2.42883291
ENSG00000273696	CT45A7	0.149135531	0.027716345	2.427815108
ENSG00000270504	RP11-420L9.5	0.842736	0.156814	2.42602639
ENSG00000278289	CT45A6	0.162268969	0.030198983	2.425815293
ENSG00000228836	CT45A5	0.127913	0.023826	2.424554223
ENSG00000269483	AC006272.1	0.659364	0.122827	2.42444739
ENSG00000217258	AC007249.3	0.587632	0.109556	2.423244463
ENSG00000176024	ZNF613	0.977255285	0.182303629	2.422392202
ENSG00000273486	RP11-731C17.2	2.229278	0.415867	2.422382424
ENSG00000177692	DNAJC28	1.165039719	0.217876956	2.418793621
ENSG00000259590	RP11-20G13.3	0.465208	0.087129	2.416651016
ENSG00000269959	SPACA6P-AS	0.218383	0.04098	2.413868665
ENSG00000150051	MKX	6.609605436	1.241635714	2.412322192
ENSG00000207331	RNU6-1263P	2.717665	0.510571	2.412184128
ENSG00000224046	AC005076.5	0.972071423	0.182840142	2.410479477
ENSG00000104371	DKK4	0.962132	0.181007	2.410189348
ENSG00000138459	SLC35A5	7.144770749	1.345684518	2.408547497
ENSG00000085741	WNT11	1.555284888	0.293031233	2.408052521
ENSG00000172061	LRRC15	0.721286503	0.135938897	2.407614105
ENSG00000244578	LINC01391	0.073812103	0.013916376	2.40707383
ENSG00000071246	VASH1	6.525928183	1.230604294	2.406816179
ENSG00000274223	AL139340.1	4.244202	0.801007	2.405606566
ENSG00000214654	RP11-27I1.4	1.132515577	0.213780308	2.40533002
ENSG00000280164	CH507-254M2.3	2.062197	0.389452	2.404664727
ENSG00000229376	CICP3	0.110008	0.020779	2.404410313
ENSG00000248008	NRAV	4.38441	0.828463	2.403873544
ENSG00000186230	ZNF749	2.954405915	0.558451887	2.403363159
ENSG00000158865	SLC5A11	0.419088755	0.079240072	2.402953722
ENSG00000275969	SPATA31A3	0.013454	0.002544	2.402864588
ENSG00000213594	GAPDHP25	0.16584	0.031359	2.402840562
ENSG00000233077	LINC01271	0.508797	0.096214	2.402771425
ENSG00000187010	RHD	0.323832026	0.061260024	2.402227844
ENSG00000175189	INHBC	0.143592	0.027169	2.401942003
ENSG00000167333	TRIM68	1.724031862	0.326479867	2.400720503
ENSG00000235823	OLMALINC	15.102847	2.862384	2.399531401
ENSG00000138829	FBN2	0.064887677	0.012303745	2.398846991
ENSG00000266278	RP11-41O4.2	0.664477	0.126014	2.398635239
ENSG00000226491	FTOP1	0.331146	0.062858	2.397299157
ENSG00000222894	AL662800.1	1.110463	0.210859	2.396810818
ENSG00000270000	RP3-449M8.9	0.198389	0.037671	2.396805799
ENSG00000132623	ANKEF1	7.512487632	1.427765725	2.395531437
ENSG00000184961	AL772307.1	1.039795	0.197945	2.393127592

ENSG00000251636	LINC01218	0.590273	0.112517	2.39123936
ENSG00000214106	PAXIP1-AS2	1.601450813	0.30552898	2.389998355
ENSG00000029534	ANK1	0.523173047	0.099922802	2.388402385
ENSG00000271270	TMCC1-AS1	0.475883565	0.091000814	2.386657283
ENSG00000280924	LINC00628	0.039485	0.007557	2.385419162
ENSG00000227544	AC018647.3	0.053593528	0.010257757	2.385343482
ENSG00000235078	AC142528.1	0.261275	0.050008	2.385338275
ENSG00000166535	A2ML1	0.027877167	0.005336107	2.385224563
ENSG00000241418	FAM195CP	1.479675	0.28325	2.385132472
ENSG00000167157	PRRX2	15.819616	3.033392	2.382710729
ENSG00000116014	KISS1R	1.653046308	0.316991923	2.382609157
ENSG00000180385	EMC3-AS1	1.614171838	0.309606391	2.38228701
ENSG00000229129	ACTG1P2	0.188052	0.03608	2.381860406
ENSG00000269343	ZNF587B	2.567421321	0.492797095	2.38125441
ENSG00000280211	RP11-2C24.3	0.067563	0.012978	2.380165318
ENSG00000160298	C21orf58	1.930193765	0.371251428	2.378277202
ENSG00000152475	ZNF837	0.673712145	0.129827276	2.375538794
ENSG00000267152	CTD-2528L19.6	0.412811	0.079589	2.374840459
ENSG00000273466	RP11-548H3.1	4.992681	0.963335	2.373705243
ENSG00000137713	PPP2R1B	6.637317625	1.282050827	2.372146857
ENSG00000251484	RP5-1065J22.4	0.920143	0.177736	2.372122163
ENSG00000242288	RP11-464F9.1	0.324456	0.062674	2.372083868
ENSG00000114251	WNT5A	0.074340769	0.01437267	2.370825537
ENSG00000270019	RP11-141B14.1	0.361665	0.069923	2.370814999
ENSG00000182585	EPGN	0.154315647	0.029835038	2.370804833
ENSG00000233190	RPS24P13	0.176064	0.034081	2.369060377
ENSG00000174792	C4orf26	0.184093585	0.035639761	2.368879771
ENSG00000166682	TMPRSS5	0.209931753	0.040661473	2.368186026
ENSG00000262503	RP11-530N7.2	3.103255	0.60134	2.367529418
ENSG00000136932	TRMO	1.860645898	0.360897439	2.36614271
ENSG00000235192	AC009495.2	0.318154	0.061755	2.365097406
ENSG00000167637	ZNF283	0.974353768	0.18937671	2.363186764
ENSG00000102870	ZNF629	7.583007	1.474867	2.362185193
ENSG00000233454	RP11-196D18.2	0.832847	0.162034	2.361754917
ENSG00000267221	CTD-2132N18.2	0.365354	0.071118	2.361008344
ENSG00000172031	EPHX4	1.892616909	0.368658488	2.360025541
ENSG00000114349	GNAT1	0.048418987	0.009432794	2.359815754
ENSG00000134326	CMPK2	0.516060773	0.100642349	2.358303477
ENSG00000171320	ESCO2	5.535486256	1.080886043	2.356495625
ENSG00000124459	ZNF45	3.341213317	0.652504556	2.356312213
ENSG00000242852	ZNF709	0.142666409	0.027876068	2.355546688
ENSG00000197757	HOXC6	1.846660918	0.360858356	2.355414417
ENSG00000257557	RP11-84G21.1	0.171228	0.033469	2.355021286
ENSG00000260772	RP11-311C24.1	45.691425	8.931577	2.354936605
ENSG00000150076	CCDC7	0.246465803	0.048187264	2.3546637
ENSG00000275203	AL583842.2	0.539997	0.105601	2.354327896
ENSG00000278024	AC138775.3	0.539997	0.105601	2.354327896
ENSG00000229465	ACTG1P11	0.184433	0.03608	2.353825572

ENSG00000213307	RPL18P11	0.480153	0.093945	2.353605907
ENSG00000229267	AC072062.1	0.607450052	0.119072517	2.350925326
ENSG00000215417	MIR17HG	0.702359532	0.137936235	2.348208232
ENSG00000100228	RAB36	2.802016338	0.550297664	2.348181259
ENSG00000267594	CYP4F24P	0.232255	0.045671	2.346359371
ENSG00000197782	ZNF780A	1.577883898	0.310284127	2.346329252
ENSG00000272281	TPTE2P2	0.044165238	0.008697125	2.344300824
ENSG00000184389	A3GALT2	0.150251	0.029626	2.342438836
ENSG00000276089	DLEU1_1	3.572069	0.704516	2.342055572
ENSG00000270012	LL0XNC01-7P3.1	0.599882	0.118407	2.34092437
ENSG00000280106	CTC-523E23.3	0.578921	0.114332	2.340137239
ENSG00000267737	AC061992.2	0.535720307	0.105878592	2.339069066
ENSG00000281267	AC110602.1	0.405284	0.080178	2.337654885
ENSG00000273329	RP11-448A19.1	0.57931	0.114613	2.337564878
ENSG00000261468	RP11-1024P17.1	2.139978	0.4237	2.336480931
ENSG00000154118	JPH3	0.091952061	0.018218956	2.335441651
ENSG00000213188	YBX1P4	0.074329	0.014736	2.334580232
ENSG00000166596	CFAP52	0.127628405	0.025319863	2.333607958
ENSG00000244617	ASPRV1	0.250623	0.049721	2.333591601
ENSG00000166963	MAP1A	2.091441971	0.41503006	2.333210232
ENSG00000234883	MIR155HG	0.09543	0.018942	2.332854206
ENSG00000281527	AC087350.1	0.681415	0.135301	2.332361204
ENSG00000230882	AC005077.14	0.15582	0.03097	2.330937132
ENSG00000237357	RP11-475I24.3	0.533005	0.105956	2.330683781
ENSG00000248240	RP11-159F24.5	0.531521	0.105704	2.330096721
ENSG00000163389	POGLUT1	2.275251156	0.452826359	2.328995962
ENSG00000167077	MEI1	0.151231369	0.030105648	2.32865134
ENSG00000277701	RP11-734K23.9	1.034775559	0.206018612	2.3284713
ENSG00000271013	LRRC37A9P	0.505365	0.100671	2.327677601
ENSG00000251867	RP11-48B3.5	0.518094	0.103233	2.327309652
ENSG00000224439	RPSAP10	0.038645	0.007705	2.326414904
ENSG00000230287	RP11-305E17.4	0.293455	0.058509	2.326408824
ENSG00000268049	CTD-2619J13.9	0.216286	0.043146	2.325641563
ENSG00000204335	SP5	0.789138557	0.157632504	2.323713581
ENSG00000271924	RNA5SP108	2.314255	0.462568	2.322810469
ENSG00000249357	RP11-432M8.8	0.453547	0.090654	2.322809478
ENSG00000263368	RP11-720N19.1	0.323112	0.064583	2.322807968
ENSG00000279670	RP11-70L8.5	0.641231	0.128334	2.320940734
ENSG00000122862	SRGN	0.101954477	0.020405641	2.320885194
ENSG00000249348	UGDH-AS1	0.629308	0.126223	2.317791465
ENSG00000264333	AC020915.1	0.00952	0.00191	2.317388935
ENSG00000059804	SLC2A3	7.959294324	1.596934091	2.317335755
ENSG00000154342	WNT3A	1.469949	0.295337	2.315332087
ENSG00000250802	ZBED3-AS1	0.174092234	0.035012663	2.313903129
ENSG00000227629	SLC25A15P1	0.776326	0.156138	2.313840911
ENSG00000281447	AL009031.1	2.729887	0.549406	2.312896663
ENSG00000236499	LINC00896	0.252089268	0.050865345	2.309179727
ENSG00000162267	ITIH3	0.017191666	0.003473099	2.307413716

ENSG00000215720	RP1-21O18.3	0.14337	0.028966	2.307310796
ENSG00000242610	OR5BH1P	0.110764	0.022379	2.307271583
ENSG00000233327	USP32P2	0.126321481	0.025536879	2.306445876
ENSG00000196793	ZNF239	1.172094436	0.237276546	2.304447404
ENSG00000275198	RP11-471B22.3	0.11595	0.023485	2.303691321
ENSG00000186918	ZNF395	11.66993986	2.363831187	2.303598212
ENSG00000168646	AXIN2	8.771611573	1.776884799	2.303491778
ENSG00000248830	ZNF807	0.677626	0.137388	2.302233232
ENSG00000270179	RP11-159N11.4	0.322384	0.06539	2.301638216
ENSG00000227268	KLLN	0.712095	0.144507	2.300930343
ENSG00000215304	RP13-395E19.3	0.570661	0.115842	2.300475554
ENSG00000226747	AC007966.1	0.076067126	0.015441959	2.300417282
ENSG00000258457	RP11-298I3.4	0.182319252	0.037018583	2.30014534
ENSG00000227879	PSPC1P1	8.401089	1.707033	2.299085402
ENSG00000153253	SCN3A	0.358992564	0.072955162	2.298871995
ENSG00000235016	SEMA3F-AS1	0.664674346	0.135121407	2.298391413
ENSG00000256982	CTD-2555A7.2	0.86481	0.175879	2.29779997
ENSG00000236358	RP5-827C21.2	0.32429	0.066001	2.296724748
ENSG00000058404	CAMK2B	0.094340824	0.019236602	2.294028256
ENSG00000183250	LINC01547	1.089236722	0.222105893	2.29399795
ENSG00000120075	HOXB5	8.95883	1.827844	2.293167386
ENSG00000264383	AC068014.1	0.591816	0.120827	2.292205826
ENSG00000145075	CCDC39	0.755733072	0.15447954	2.290460988
ENSG00000265038	PMM2P1	0.193359	0.03954	2.289897141
ENSG00000207340	RNVU1-1	1.613782	0.330003	2.289894658
ENSG00000254935	RP11-266E8.2	0.624199	0.127643	2.289891624
ENSG00000267116	RP11-64C12.7	0.19283	0.039432	2.289890729
ENSG00000078900	TP73	3.619407739	0.740542316	2.289099561
ENSG00000245556	SCAMP1-AS1	1.071320088	0.219517604	2.286981047
ENSG00000226003	RP11-312J18.3	0.267828	0.054889	2.286717834
ENSG00000280145	CH507-154B10.1	0.473044917	0.096999078	2.285934236
ENSG00000273782	UCA1	16.44978	3.375609	2.284848579
ENSG00000274191	RP11-324E6.10	0.484637	0.099487	2.284324627
ENSG00000240050	RP1-93H18.1	0.191339	0.039336	2.282208798
ENSG00000274026	FAM27E3	0.660990472	0.135896	2.282126484
ENSG00000261168	RP11-68I18.10	0.565293	0.116275	2.281457892
ENSG00000232450	RP4-730K3.3	1.054667	0.217061	2.280615114
ENSG00000144362	PHOSPHO2	1.589901023	0.327826481	2.277932653
ENSG00000204569	PPP1R10	22.52820613	4.648412633	2.276922394
ENSG00000173065	FAM222B	3.234420414	0.667402152	2.27687897
ENSG00000102678	FGF9	1.213276152	0.250392245	2.276646171
ENSG00000128408	RIBC2	2.152722782	0.444295674	2.276570547
ENSG00000250990	AC073635.5	0.349886	0.072342	2.27397955
ENSG00000227896	RP11-77P6.2	0.509425	0.105524	2.271298604
ENSG00000250940	RP11-489M13.3	0.295217	0.061153	2.271280621
ENSG00000104972	LILRB1	0.018932901	0.003923419	2.270712304
ENSG00000214946	TBC1D26	0.039776745	0.008253479	2.268850943
ENSG00000124217	MOCS3	1.369792	0.284276	2.26859263

ENSG00000145040	UCN2	0.704313	0.146173	2.26853986
ENSG00000183935	HTR7P1	0.354692705	0.073616599	2.268466661
ENSG00000172954	LCLAT1	7.12059407	1.479438155	2.266948221
ENSG00000271182	RP11-678G14.5	2.6561	0.55225	2.265916045
ENSG00000235903	CPB2-AS1	0.375062881	0.078018631	2.265241908
ENSG00000206739	Y_RNA	1.254228	0.260938	2.26502067
ENSG00000254996	ANKHD1-EIF4EBP3	3.917965523	0.816121904	2.263248133
ENSG00000178163	ZNF518B	5.079054772	1.058332179	2.262767515
ENSG00000107593	PKD2L1	0.083028727	0.017304891	2.262430742
ENSG00000260400	RP11-119F7.5	0.904822	0.188656	2.261876023
ENSG00000248898	CTD-2288O8.1	0.437725	0.091266	2.261875376
ENSG00000253966	CTC-455F18.3	0.194069	0.040479	2.26132413
ENSG00000263465	SRSF8	8.442826	1.765645	2.257530677
ENSG00000205730	ITPRIPL2	8.785455133	1.840243446	2.255220394
ENSG00000173540	GMPPB	2.131794726	0.446633374	2.254905563
ENSG00000125551	PLGLB2	0.238056435	0.049902367	2.254123476
ENSG00000144285	SCN1A	0.252659088	0.053007315	2.252928706
ENSG00000258944	RP4-647C14.3	0.995034	0.208959	2.251525925
ENSG00000185379	RAD51D	0.355913636	0.074838051	2.249683324
ENSG00000231588	RP11-50E11.2	1.000124	0.210313	2.249568948
ENSG00000281808	SNORA17	1.127328	0.237063	2.249564919
ENSG00000266962	RP11-400F19.6	5.078672	1.068578	2.248759082
ENSG00000010030	ETV7	0.627940413	0.13222291	2.247655494
ENSG00000089101	CFAP61	0.104267149	0.022026662	2.242961905
ENSG00000145022	TCTA	3.704949644	0.784521262	2.239569481
ENSG00000174684	B4GAT1	5.054249	1.070396	2.239352111
ENSG00000226752	PSMD5-AS1	1.210844402	0.256530541	2.238810983
ENSG00000197406	DIO3	0.961749	0.203918	2.237671296
ENSG00000061455	PRDM6	0.268035338	0.056840904	2.237421822
ENSG00000106006	HOXA6	1.377894041	0.292292985	2.236977839
ENSG00000247137	RP11-727A23.5	0.884418126	0.187638554	2.236772302
ENSG00000089335	ZNF302	2.049723821	0.434888829	2.236710979
ENSG00000232578	CTC-303L1.1	0.06296	0.013389	2.233387328
ENSG00000236695	HNRNPA1P47	0.054669	0.011626	2.233368172
ENSG00000266909	SLC25A6P4	0.184073	0.039146	2.233341223
ENSG00000214313	AZGP1P1	0.07333761	0.015596542	2.233327082
ENSG00000266821	RP11-6N17.2	0.086943	0.01849	2.233324652
ENSG00000228697	RP5-968D22.1	0.13853092	0.029461105	2.23332457
ENSG00000237025	RP1-315G1.1	0.359945	0.076549	2.233321042
ENSG00000273365	RP11-466F5.10	0.349083	0.074239	2.233320918
ENSG00000226979	LTA	0.175807123	0.037388696	2.233319464
ENSG00000273367	RP5-827C21.6	0.406519	0.086454	2.233318163
ENSG00000259974	LINC00261	0.015278691	0.003249305	2.233318023
ENSG00000171772	SYCE1	0.062525919	0.01329733	2.233317792
ENSG00000264372	AC108519.1	0.771155	0.164001	2.233316255
ENSG00000236318	AC019117.1	0.30404	0.06466	2.233315727
ENSG00000253633	KB-1980E6.3	0.103364114	0.021982388	2.23331535
ENSG00000272438	RP11-54O7.16	0.725017	0.154189	2.233314978

ENSG00000271551	RP11-230C9.2	0.203748	0.043331	2.233314461
ENSG00000104804	TULP2	0.11360752	0.024160875	2.233313747
ENSG00000265683	RP11-173M1.5	0.49006	0.104221	2.233312392
ENSG00000188707	ZBED6CL	1.388236	0.295236	2.233312293
ENSG00000272420	RP4-740C4.7	0.89606	0.190565	2.233312166
ENSG00000237768	RP11-344N10.2	0.139921	0.029757	2.233311514
ENSG00000200579	Y_RNA	2.494911	0.530593	2.233310804
ENSG00000250474	WBP1LP2	0.269008	0.05721	2.233309828
ENSG00000256984	RP11-551L14.6	0.731268	0.155519	2.233309384
ENSG00000240288	GHRLOS	0.297290769	0.063224932	2.233309175
ENSG00000221216	RNU6ATAC27P	0.427699	0.090959	2.233307536
ENSG00000255992	RP11-417L19.4	0.597374	0.127044	2.233306206
ENSG00000249400	HMGB3P17	0.392113	0.083391	2.233305879
ENSG00000182366	FAM87A	0.028082077	0.00597224	2.233305685
ENSG00000273355	RP11-672L10.6	0.526875	0.112051	2.233305202
ENSG00000280426	RP11-837J7.3	0.148906	0.031668	2.233304225
ENSG00000273474	RP11-295P9.12	0.737627	0.156872	2.233303596
ENSG00000162896	PIGR	0.058153722	0.01236764	2.233301321
ENSG00000279791	RP11-499E14.1	0.101833	0.021657	2.233299842
ENSG00000279345	RP3-522J7.7	0.144838	0.030803	2.233297389
ENSG00000127324	TSPAN8	0.10102494	0.021485193	2.233296885
ENSG00000235267	AC074117.13	0.128202	0.027265	2.233296708
ENSG00000231728	LL0XNC01-116E7.2	0.194111802	0.041282258	2.233294052
ENSG00000278888	AC090154.1	0.208347	0.04431	2.233284095
ENSG00000244476	ERVFRD-1	0.078495126	0.016693903	2.233281831
ENSG00000103522	IL21R	0.185251806	0.039399566	2.233235957
ENSG00000165805	C12orf50	0.045369844	0.009649352	2.233229801
ENSG00000254245	PCDHGA3	0.010438427	0.002220225	2.233126809
ENSG00000229676	ZNF492	0.064846	0.013812	2.231095346
ENSG00000232335	RP11-435D7.3	0.476296	0.101472	2.230776745
ENSG00000260031	RPL10P14	1.029197	0.219388	2.229962636
ENSG00000273888	FRMD6-AS1	0.341592	0.072841	2.229451554
ENSG00000267121	CTD-2020K17.1	0.221339672	0.047240133	2.228177112
ENSG00000237813	AC002066.1	3.516093969	0.750615651	2.22782735
ENSG00000250565	ATP6V1E2	1.938322729	0.413865268	2.227575713
ENSG00000259609	RP11-342A23.1	0.127241	0.027169	2.227530244
ENSG00000254207	RP11-43A14.1	0.802099	0.171268	2.227524693
ENSG00000159650	UROC1	0.076137882	0.016257694	2.227491812
ENSG00000156218	ADAMTSL3	0.025985653	0.005549006	2.227413979
ENSG00000269656	GLTSCR2-AS1	0.495099	0.105853	2.225654878
ENSG00000246695	RASSF8-AS1	1.411853072	0.30189585	2.225467127
ENSG00000272989	RP13-616I3.1	0.29328	0.062748	2.22463731
ENSG00000279253	RP4-614O4.13	1.385102	0.296348	2.224627995
ENSG00000168329	CX3CR1	0.138415424	0.029614711	2.224618786
ENSG00000236914	RP11-1008C21.2	0.169249467	0.036221033	2.224251678
ENSG00000279147	RP11-271K21.12	0.137124	0.029397	2.221740261
ENSG00000260347	MOCS1P1	0.078302	0.016787	2.221704728
ENSG00000168314	MOBP	0.027330119	0.005859313	2.221688288

ENSG00000254872	RP13-870H17.3	0.073321253	0.015719616	2.221665461
ENSG00000140948	ZCCHC14	13.21119649	2.836027277	2.219817818
ENSG00000168447	SCNN1B	0.128604645	0.027626433	2.218821566
ENSG00000197889	MEIG1	0.357991392	0.076930526	2.218296823
ENSG00000175536	LIPT2	0.835321229	0.179587738	2.217642255
ENSG00000188763	FZD9	0.430593	0.092593	2.217349831
ENSG00000176714	CCDC121	0.796372084	0.17126679	2.217197224
ENSG00000142686	C1orf216	7.174401003	1.54349008	2.216662174
ENSG00000276071	CTD-3234P18.6	0.192961	0.041535	2.215909829
ENSG00000216425	AC004066.2	0.15675	0.033741	2.215890806
ENSG00000280259	RP11-479O17.9	0.111696	0.024043	2.215888695
ENSG00000273372	SFTPD-AS1	0.100551	0.021644	2.215888378
ENSG00000109061	MYH1	0.010223	0.002202	2.214932252
ENSG00000087589	CASS4	0.198203106	0.042706982	2.21443571
ENSG00000135436	FAM186B	0.338808107	0.073055667	2.213400307
ENSG00000142185	TRPM2	2.137313843	0.46096543	2.213068303
ENSG00000214883	RP11-574M7.2	0.240804	0.051939	2.212969214
ENSG00000211517	MIR671	0.987792	0.213416	2.210538944
ENSG00000235686	PPIAP20	0.309148	0.066815	2.210053742
ENSG00000075073	TACR2	0.08722	0.018850751	2.210036993
ENSG00000273328	RP11-141M3.6	0.063668646	0.013760962	2.210001723
ENSG00000131944	FAAP24	1.472768808	0.31858214	2.208793681
ENSG00000249896	RP11-586D19.1	1.168594	0.25286	2.208363047
ENSG00000223842	RP11-135J2.3	0.222031	0.048064	2.207732497
ENSG00000086570	FAT2	0.443732408	0.096094165	2.207169189
ENSG00000219146	RPS4XP8	0.298192	0.06458	2.207082207
ENSG00000185090	MANEAL	2.708786524	0.587053038	2.206083945
ENSG00000236743	RP5-857K21.15	0.118584	0.0257	2.206069102
ENSG00000131848	ZSCAN5A	0.709551628	0.153820747	2.205657553
ENSG00000275224	MIR6501	0.22161	0.048117	2.203404382
ENSG00000188981	MSANTD1	0.039598044	0.008598373	2.203293638
ENSG00000115461	IGFBP5	0.618306553	0.134419394	2.201580993
ENSG00000124818	OPN5	0.049011451	0.010657838	2.201204096
ENSG00000270921	CICP23	0.098546	0.021432	2.201030827
ENSG00000277714	MIR6791	0.774838	0.168525	2.200932084
ENSG00000101144	BMP7	3.301605267	0.718165977	2.200778433
ENSG00000274099	ABCB10P1	0.080977	0.017618	2.200462037
ENSG00000271781	CTD-2589H19.6	0.313999	0.06842	2.198269955
ENSG00000008853	RHOBTB2	4.182160937	0.911617485	2.19774808
ENSG00000234500	GS1-124K5.10	0.418407	0.091215	2.197563993
ENSG00000168631	DPCR1	0.181211	0.039516	2.197161711
ENSG00000235209	CTA-150C2.13	0.226706	0.049491	2.195584476
ENSG00000225361	PPP1R26-AS1	0.657081145	0.143455438	2.195468891
ENSG00000101188	NTSR1	40.0742644	8.753553374	2.194735357
ENSG00000180155	LYNX1	1.28547985	0.280863234	2.194367306
ENSG00000159339	PADI4	0.083257018	0.018216883	2.192295787
ENSG00000119714	GPR68	0.370888986	0.081195426	2.19151707
ENSG00000183779	ZNF703	3.558631	0.779243	2.19117715

ENSG00000121931	LRIF1	8.72376424	1.911894807	2.189947634
ENSG00000274642	CH17-472G23.1	0.168858	0.037011	2.18978451
ENSG00000178033	FAM26E	0.149266	0.032746	2.18849499
ENSG00000241127	YAE1D1	4.700116843	1.032240624	2.186917308
ENSG00000279632	RP11-286N22.6	0.280566	0.061641	2.186378017
ENSG00000280384	RP4-695O20.1	0.120458	0.026465	2.186372652
ENSG00000261762	RP11-650L12.2	5.932037	1.303399	2.186248803
ENSG00000279692	RP11-1055B8.1	0.621623	0.136711	2.184910555
ENSG00000174516	PELI3	2.613027622	0.574978462	2.184142556
ENSG00000250071	CTD-2060C23.1	0.29609	0.065186	2.183401711
ENSG00000229661	BUD31P2	0.67183	0.14793	2.183181561
ENSG00000122783	C7orf49	3.867357089	0.852236833	2.182021671
ENSG00000206530	CFAP44	1.197814343	0.264009671	2.181741628
ENSG00000189269	DRICH1	0.689698925	0.152135945	2.180605663
ENSG00000176410	DNAJC30	3.145881	0.694746	2.17890657
ENSG00000253848	RP11-10N23.5	1.635703	0.361271	2.178757461
ENSG00000259891	CTA-204B4.2	0.767891	0.169809	2.176988614
ENSG00000204539	CDSN	0.049859	0.011028	2.176682775
ENSG00000273706	LHX1	0.150344641	0.033257297	2.176530607
ENSG00000134258	VTCN1	0.242459334	0.053637148	2.176438363
ENSG00000127083	OMD	0.061932	0.013701	2.176403841
ENSG00000128262	POM121L9P	0.401509596	0.088846049	2.176054942
ENSG00000272221	XXbac-BPG181B23.7	1.215268	0.269033	2.175419451
ENSG00000271699	SNX29P2	0.051088153	0.011312442	2.175078376
ENSG00000222585	RNA5SP494	0.378762	0.083907	2.174428518
ENSG00000273619	RP5-908M14.9	0.731443	0.162038	2.174413259
ENSG00000147124	ZNF41	4.38108913	0.971092682	2.173608667
ENSG00000249717	RP11-44F21.3	0.0730786	0.016198839	2.173558564
ENSG00000263721	MIR4444-1	0.800971	0.17759	2.173199663
ENSG00000128713	HOXD11	0.56220995	0.124656876	2.173146525
ENSG00000259546	RP11-351M8.2	0.674636	0.149702	2.17201581
ENSG00000213783	RPL35P4	0.37856	0.084038	2.171408246
ENSG00000130812	ANGPTL6	0.111689699	0.024814386	2.170247471
ENSG00000160207	HSF2BP	0.315299689	0.070061953	2.17002064
ENSG00000236473	KRT43P	0.353274	0.078504	2.169949504
ENSG00000249685	RP11-360F5.3	1.069359	0.237892	2.168367607
ENSG00000248213	CICP16	0.354362	0.078904	2.167053561
ENSG00000279667	RP11-656D10.7	0.705963	0.157246	2.166569254
ENSG00000155749	ALS2CR12	0.335598037	0.074762572	2.166346165
ENSG00000150667	FSIP1	0.429453009	0.095733101	2.165410538
ENSG00000125378	BMP4	168.7544117	37.64565179	2.164370075
ENSG00000215381	RP5-1057J7.1	0.713285	0.159224	2.163420824
ENSG00000233250	RP3-339A18.6	0.667054	0.148956	2.16291732
ENSG00000251555	RP11-745L13.2	0.533625	0.119208	2.1623452
ENSG00000112081	SRSF3	40.57174451	9.071450386	2.161070198
ENSG00000266542	MIR5572	0.004953	0.001108	2.16034474
ENSG00000279806	RP11-58O9.2	5.303349	1.186536	2.160147819
ENSG00000136535	TBR1	0.067648884	0.015137897	2.159901361

ENSG00000126882	FAM78A	2.196339283	0.49150505	2.15982279
ENSG00000270808	RP11-574K11.27	0.281769	0.063077	2.159326945
ENSG00000204934	ATP6V0E2-AS1	0.293840985	0.065779996	2.159314806
ENSG00000225302	RP11-539I5.1	1.023002059	0.229137	2.158526705
ENSG00000104899	AMH	1.739766816	0.389868568	2.1578342
ENSG00000230613	HM13-AS1	0.306001	0.068619	2.156856361
ENSG00000224356	RP11-151A6.4	0.124811252	0.027991159	2.156704889
ENSG00000108733	PEX12	1.625578515	0.365436631	2.153260081
ENSG00000166349	RAG1	0.338136292	0.076039089	2.152791706
ENSG00000188659	SAXO2	0.502573841	0.113097424	2.151769516
ENSG00000221923	ZNF880	0.032297752	0.007276432	2.150130686
ENSG00000047621	C12orf4	4.439902331	1.000298887	2.150096802
ENSG00000171889	MIR31HG	1.60661	0.363225	2.145084353
ENSG00000185112	FAM43A	8.112373	1.834977	2.144362008
ENSG00000231770	TMEM44-AS1	2.951986818	0.668406	2.14288969
ENSG00000176692	FOXC2	0.326573	0.074039	2.141048204
ENSG00000189229	AC069277.2	0.050509071	0.011457972	2.140190798
ENSG00000109846	CRYAB	0.050325475	0.01141648	2.140170988
ENSG00000215251	FASTKD5	10.950153	2.484187	2.140105345
ENSG00000232706	NUTM2HP	0.332246	0.075465	2.138372235
ENSG00000186628	FSD2	0.036443161	0.008280752	2.137814445
ENSG00000180035	ZNF48	5.42240392	1.232377242	2.137488639
ENSG00000115902	SLC1A4	2.180930276	0.495734396	2.13730438
ENSG00000101542	CDH20	0.057746517	0.013138859	2.135893893
ENSG00000245164	LINC00861	0.604500685	0.137544067	2.135850059
ENSG00000156427	FGF18	2.126614	0.484523	2.133921138
ENSG00000110934	BIN2	0.056862088	0.01295627	2.133816646
ENSG00000277200	RP11-74E22.8	0.11321	0.025796	2.133782119
ENSG00000273045	C2orf15	0.666000956	0.151811418	2.133243949
ENSG00000257496	RP11-474P2.4	0.422022	0.0963	2.131710505
ENSG00000271895	RP4-635E18.8	2.648941897	0.604657	2.131227308
ENSG00000219547	RPL17P25	0.142372	0.032505	2.130931883
ENSG00000118762	PKD2	5.552040772	1.269232004	2.129062358
ENSG00000144460	NYAP2	0.021084	0.00482	2.129043546
ENSG00000225385	RP11-350E12.4	0.488213	0.111616	2.128966861
ENSG00000203668	CHML	4.879795	1.116513	2.127820492
ENSG00000280667	Y_RNA	0.80416	0.18401	2.127698405
ENSG00000060749	QSER1	13.13928857	3.007332015	2.127331105
ENSG00000179873	NLRP11	0.328975649	0.075307003	2.127124857
ENSG00000234857	HNRNPUL2-BSCL2	11.610435	2.661822	2.124936021
ENSG00000171084	FAM86JP	4.043588337	0.927323227	2.124491933
ENSG00000283064	RP1-221C16.8	0.553881	0.127044	2.124247808
ENSG00000269549	RP3-461P17.10	0.074754494	0.017181	2.121346302
ENSG00000173966	RP11-18M17.1	1.024711	0.235725	2.120040405
ENSG00000223802	CERS1	0.152769788	0.035179486	2.118552938
ENSG00000162782	TDRD5	0.2207575	0.050865016	2.117716823
ENSG00000118513	MYB	1.545501518	0.356369927	2.11662757
ENSG00000261708	DNM1P32	0.101356	0.023379	2.116146368

ENSG00000258168	RP11-588H23.3	1.631024786	0.376444339	2.115270238
ENSG00000271324	RP11-10C24.2	0.261578	0.060374	2.115241915
ENSG00000267141	CTB-31O20.8	0.512084	0.118256	2.114467099
ENSG00000173376	NDNF	0.263605111	0.060877881	2.114388294
ENSG00000162227	TAF6L	3.172488567	0.7328907	2.113945003
ENSG00000188993	LRRC66	0.274517	0.063441	2.113408077
ENSG00000279573	RP11-855A2.1	0.118363	0.027361	2.113025304
ENSG00000179431	FJX1	2.064101	0.477475409	2.112015227
ENSG00000279382	RP11-449J21.3	2.301355	0.533258	2.109577937
ENSG00000121410	A1BG	0.054786372	0.012699213	2.109078019
ENSG00000199273	Y_RNA	0.096866	0.022475	2.107669252
ENSG00000272507	RNU6-88P	0.885594	0.205658	2.106398255
ENSG00000138622	HCN4	1.248928	0.290071	2.106212335
ENSG00000237054	PRMT5-AS1	0.890826427	0.20736695	2.102958379
ENSG00000281255	AL139099.2	51.16872	11.91216	2.102827107
ENSG00000280187	CTC-351M12.1	0.808352	0.188215	2.102602047
ENSG00000105679	GAPDHS	0.082426154	0.019207022	2.101468352
ENSG00000267419	CTC-559E9.6	0.310530153	0.072436397	2.099946679
ENSG00000187189	TSPYL4	4.431136	1.034246	2.099097229
ENSG00000226952	RP4-714D9.4	0.165614	0.038657	2.099023049
ENSG00000225506	CYP4A22-AS1	0.503252	0.117526	2.098301045
ENSG00000062725	APPBP2	10.4103953	2.43247538	2.097527743
ENSG00000229750	AC096649.2	0.360336	0.084203	2.097399254
ENSG00000200719	RNA5SP260	0.458524	0.107266	2.09580439
ENSG00000164318	EGFLAM	0.017783323	0.00416055	2.09567871
ENSG00000277327	SPDYE20P	0.220444	0.051624	2.094298375
ENSG00000212127	TAS2R14	0.786535666	0.184240071	2.093925317
ENSG00000196653	ZNF502	1.128848359	0.264567278	2.093145157
ENSG00000126752	SSX1	0.355646	0.083391	2.092478344
ENSG00000268751	SCGB1B2P	0.659337604	0.154630344	2.092193908
ENSG00000271449	CT45A2	0.053143195	0.012472966	2.091080391
ENSG00000261572	RP11-384L8.1	1.211174	0.284484	2.089986721
ENSG00000110446	SLC15A3	0.074526836	0.017512299	2.089391516
ENSG00000180530	NRIP1	4.072593995	0.957109127	2.089192665
ENSG00000226180	RP11-278A23.1	0.687713	0.161678	2.088683237
ENSG00000162490	DRAXIN	2.384764	0.56095	2.087902414
ENSG00000126500	FLRT1	0.115222	0.027136	2.086136225
ENSG00000203804	ADAMTSL4-AS1	0.175145889	0.041272326	2.085310487
ENSG00000263325	LA16c-325D7.1	0.865537	0.204228	2.083414818
ENSG00000230818	MTND2P16	0.243971	0.057575	2.083195259
ENSG00000260495	RP11-55K13.1	0.377989	0.08923	2.082743506
ENSG00000165828	PRAP1	0.065140514	0.015379575	2.082539462
ENSG00000220201	ZGLP1	0.282743223	0.066787947	2.081832762
ENSG00000260597	AC012531.25	0.853082	0.201538	2.081632538
ENSG00000225333	RP1-144F13.4	0.403227	0.095282	2.081316643
ENSG00000188582	PAQR9	0.109874773	0.025963322	2.081313309
ENSG00000278937	RP11-139H14.5	0.28416	0.067147	2.081308637
ENSG00000262155	RP11-266L9.5	0.239416	0.056574	2.081308486

ENSG00000244968	LIFR-AS1	0.089851881	0.021232049	2.081305091
ENSG00000262185	RP11-462G12.1	0.328598	0.077648	2.081303029
ENSG00000135625	EGR4	0.096354	0.022769	2.081273629
ENSG00000253767	PCDHGA8	0.026443727	0.006248812	2.081271707
ENSG00000170364	SETMAR	1.799345487	0.425215282	2.081206869
ENSG00000118407	FILIP1	0.102753116	0.024307243	2.079723992
ENSG00000162396	PARS2	4.865801	1.151128	2.079629055
ENSG00000144355	DLX1	2.400389485	0.567874117	2.079625453
ENSG00000168546	GFRA2	0.079959363	0.018936724	2.078080181
ENSG00000223779	RP11-403I13.4	0.105919	0.02509	2.077777031
ENSG00000275763	C18orf65	0.439826	0.104278	2.076498073
ENSG00000144026	ZNF514	0.992476361	0.235350229	2.076223481
ENSG00000136161	RCBTB2	0.996818543	0.236481201	2.075605407
ENSG00000260806	RP11-872J21.3	0.301607	0.071588	2.074880233
ENSG00000130749	ZC3H4	5.978865042	1.419314667	2.074677171
ENSG00000250541	RP11-302F12.10	0.471993	0.112051	2.074609939
ENSG00000272578	AP000347.2	0.682505949	0.162083985	2.074100075
ENSG00000279118	RP11-517I3.2	0.19562	0.046535	2.071665765
ENSG00000115468	EFHD1	2.678554735	0.637317044	2.071371628
ENSG00000166268	MYRFL	0.089545275	0.021326316	2.069982534
ENSG00000111254	AKAP3	0.92296587	0.219846704	2.069779396
ENSG00000256542	RP13-895J2.3	0.224161	0.053426	2.068921385
ENSG00000067646	ZFY	0.123211551	0.029375958	2.06842971
ENSG00000205559	CHKB-AS1	0.785457	0.187268	2.068427901
ENSG00000273532	H19_1	1.598572	0.381131	2.068424863
ENSG00000278685	IQCA1L	0.073841277	0.017619548	2.067250615
ENSG00000204128	C2orf72	1.931703612	0.461239719	2.066285096
ENSG00000278763	FAM27B	0.531264	0.127044	2.064100713
ENSG00000213606	AKR1B10P1	0.080036	0.019151	2.06322934
ENSG00000151640	DPYSL4	3.181349349	0.76170985	2.062325348
ENSG00000272172	RP13-582O9.7	1.01336	0.242693	2.061942386
ENSG00000234569	RAD1P1	0.277957	0.066569	2.061939314
ENSG00000269972	RP3-430N8.10	0.128033	0.030681	2.061098296
ENSG00000224843	LINC00240	0.066908796	0.01603373	2.061085779
ENSG00000214041	PGAM1P4	1.718369	0.411918	2.060610796
ENSG00000270946	CT45A9	0.069370632	0.016638053	2.059838388
ENSG00000278085	CT45A8	0.069507089	0.016670923	2.059826165
ENSG00000175305	CCNE2	3.465249175	0.831139378	2.059796759
ENSG00000066294	CD84	0.032666543	0.007836258	2.059577038
ENSG00000171462	DLK2	1.020426418	0.244807914	2.059450051
ENSG00000198570	RD3	0.046529	0.011164	2.059276152
ENSG00000229953	RP11-284F21.7	10.92908	2.623964	2.05835213
ENSG00000115594	IL1R1	4.261701621	1.023560377	2.057833382
ENSG00000276624	AL078621.1	0.303742	0.073008	2.056719947
ENSG00000226435	ANKRD18DP	0.399829056	0.09614277	2.056133043
ENSG00000215198	RP11-182N22.7	0.097177	0.023376	2.055586812
ENSG00000273327	OR6L2P	0.243201	0.058509	2.055418695
ENSG00000235368	SAPCD2P2	0.558312	0.134456	2.053937429

ENSG00000255142	AP006621.6	0.917892	0.221055	2.053919048
ENSG00000171574	ZNF584	3.105770873	0.74834614	2.053173766
ENSG00000272681	FAM223B	0.214148651	0.051616477	2.052709011
ENSG00000273259	SERPINA3	0.269057	0.064984	2.049755387
ENSG00000271974	RP11-927P21.12	0.713544	0.172443	2.048882828
ENSG00000262312	LA16c-390H2.4	0.075784264	0.018315	2.048872614
ENSG00000257267	ZNF271P	3.754731701	0.907537868	2.048680075
ENSG00000213211	KRT18P64	0.128547	0.031078	2.048330373
ENSG00000163885	CFAP100	0.089487813	0.021635405	2.048297119
ENSG00000265218	RP11-927P21.1	0.329864784	0.079764627	2.048053758
ENSG00000242411	CTD-2086L14.1	0.535383	0.129534	2.047240505
ENSG00000270223	RP13-36G14.4	0.740975	0.179405	2.046204769
ENSG00000228703	RP5-1160K1.6	0.329324	0.079737	2.046186422
ENSG00000237943	PRKCQ-AS1	0.688348225	0.166867176	2.044438391
ENSG00000276232	SCARNA10	0.820907	0.199151	2.043356065
ENSG00000232828	AF196970.3	0.371562	0.09018	2.042723548
ENSG00000155754	ALS2CR11	0.032710465	0.007941648	2.04224201
ENSG00000271430	RP3-368A4.5	4.5671	1.109918	2.040825284
ENSG00000254027	RP11-363E6.3	0.369761455	0.089866391	2.040741274
ENSG00000207169	RNU6-24P	0.297291	0.072257	2.040666526
ENSG00000272897	RP1-309K20.6	1.7598375	0.4278105	2.040398422
ENSG00000243761	RP11-216N21.2	2.149509	0.522544	2.040382724
ENSG00000278455	RP11-261C10.8	0.066521	0.016195	2.038261388
ENSG00000170577	SIX2	0.501807	0.122231	2.037522371
ENSG00000180535	BHLHA15	2.220433393	0.541205622	2.036592565
ENSG00000256967	RP11-273B20.1	1.602179682	0.390605088	2.036253305
ENSG00000254685	FPGT	3.156235093	0.769500212	2.03621104
ENSG00000196597	ZNF782	1.135422006	0.276859525	2.036002548
ENSG00000136928	GABBR2	0.061709158	0.015050249	2.03569729
ENSG00000230487	PSMG3-AS1	0.212063679	0.051720375	2.035692916
ENSG00000089127	OAS1	0.551246693	0.134473583	2.035375308
ENSG00000137561	TTPA	0.998111438	0.243628604	2.034517375
ENSG00000169783	LINGO1	5.780695603	1.411927045	2.03357756
ENSG00000137726	FXYD6	2.009625685	0.491018669	2.033077024
ENSG00000125787	GNRH2	0.247368649	0.060499241	2.031673729
ENSG00000172458	IL17D	0.061191492	0.014965751	2.031666366
ENSG00000169856	ONECUT1	0.287133792	0.070286468	2.030404274
ENSG00000231995	RP11-111F5.2	1.057275	0.258896	2.029906094
ENSG00000266936	CTC-215O4.4	0.603523	0.14787	2.029079366
ENSG00000241520	AC098820.4	0.226321	0.055452	2.029059056
ENSG00000115616	SLC9A2	1.218015617	0.298459301	2.028926512
ENSG00000272374	RP3-329A5.8	0.308889	0.075701	2.028704232
ENSG00000182866	LCK	0.264470822	0.064834043	2.028285114
ENSG00000188807	TMEM201	4.821767269	1.182133179	2.02816944
ENSG00000232496	RPL3P12	0.515843	0.126491	2.0278973
ENSG00000215034	DSTNP4	0.209383	0.05138	2.026865518
ENSG00000135736	CCDC102A	2.000760148	0.492242283	2.023107732
ENSG00000186088	GSAP	0.050815712	0.012506581	2.022587236

ENSG00000130701	RBBP8NL	0.324366	0.079834	2.022547402
ENSG00000251155	SEPT14P4	0.705816	0.173775	2.02207159
ENSG00000152778	IFIT5	7.359478	1.813004	2.021221333
ENSG00000206846	Y_RNA	2.663929	0.656356	2.021005198
ENSG00000251144	CTD-2532K18.2	2.294762	0.56572	2.020184452
ENSG00000196670	ZFP62	1.728475923	0.426351321	2.019385876
ENSG00000164741	DLC1	0.039780973	0.009813491	2.019240187
ENSG00000234546	RP3-510D11.2	0.37220969	0.091829325	2.01908877
ENSG00000176095	IP6K1	4.879877993	1.2040222	2.018983085
ENSG00000213057	C1orf220	0.236966062	0.058508579	2.017960354
ENSG00000274653	RP11-347C12.11	0.856199	0.211408	2.017916179
ENSG00000278000	AC139100.4	1.323916	0.327013	2.017391694
ENSG00000240280	TCAM1P	0.926495411	0.228936609	2.016835651
ENSG00000129646	QRICH2	2.143941645	0.530010968	2.016171519
ENSG00000227632	AC018804.6	0.531228	0.13136	2.015805158
ENSG00000177098	SCN4B	0.487472476	0.120715446	2.013710473
ENSG00000234419	CICP13	0.259822	0.06436	2.013287363
ENSG00000165480	SKA3	20.20555089	5.005557899	2.013148906
ENSG00000130193	THEM6	7.844428038	1.943568916	2.012959995
ENSG00000178977	LINC00324	0.216101	0.053549	2.012774209
ENSG00000123444	KBTBD4	5.621424652	1.393806979	2.011905019
ENSG00000271941	RP11-188P20.3	0.633368	0.157056	2.01176492
ENSG00000224891	AC007899.3	0.140799	0.034916	2.01167689
ENSG00000144015	TRIM43	0.03026	0.007508	2.010911432
ENSG00000278655	KB-1572G7.5	0.206071	0.051137	2.01070206
ENSG00000197360	ZNF98	0.06734733	0.01671908	2.010125289
ENSG00000254473	RP11-522I20.3	1.345562746	0.334077325	2.009955696
ENSG00000159871	LYPD5	0.760355407	0.188800038	2.009814871
ENSG00000071655	MBD3	4.632723258	1.150371372	2.009760825
ENSG00000273826	MIR6861	0.095238	0.023678	2.007990098
ENSG00000245848	CEBPA	2.183096	0.543056	2.007202691
ENSG00000139515	PDX1	1.024436	0.254914	2.006747343
ENSG00000224904	RP5-934G17.6	0.082286	0.020524	2.003335063
ENSG00000149926	FAM57B	0.351931698	0.087780476	2.003323458
ENSG00000164220	F2RL2	0.308798	0.077022	2.003320918
ENSG00000112149	CD83	7.339983178	1.831300839	2.002907946
ENSG00000196553	LINC00238	0.153011881	0.038193735	2.002235782
ENSG00000231531	HINT1P1	0.573592	0.143176	2.002235223
ENSG00000136870	ZNF189	4.639112851	1.158622826	2.001437948
ENSG00000278422	RN7SL331P	0.274744	0.068655	2.000651277
ENSG00000278785	Metazoa_SRP	0.274744	0.068655	2.000651277
ENSG00000103241	FOXF1	1.832901	0.458085	2.000441636
ENSG00000163430	FSTL1	6.100497868	1.524811042	2.000296515
ENSG00000255122	RP11-303G3.6	1.163298	0.29097	1.999278398
ENSG00000260743	RP11-255C15.3	0.552513	0.138239	1.998843723
ENSG00000139194	RBP5	1.379725312	0.345653007	1.996984691
ENSG00000149798	CDC42EP2	7.764807602	1.945320984	1.996941953
ENSG00000253293	HOXA10	6.951726444	1.74233944	1.996345597

ENSG00000246859	STARD4-AS1	0.83608014	0.209610497	1.995930269
ENSG00000272669	RP3-508I15.21	1.318533	0.330675	1.995447796
ENSG00000230872	MFSD13B	0.167287	0.041954	1.995445064
ENSG00000160602	NEK8	0.827177783	0.207648301	1.99405537
ENSG00000231025	RP11-175O19.4	7.312813	1.836397	1.99354849
ENSG00000232470	RP11-313D6.3	0.35456	0.089076	1.992921107
ENSG00000259869	AL022344.7	0.444816	0.11182	1.992030433
ENSG00000206712	RNU6-26P	8.490629	2.134474	1.991990846
ENSG00000116406	EDEM3	5.395493407	1.3574189	1.990888892
ENSG00000273174	RP11-434H6.6	1.043147	0.262461	1.990767508
ENSG00000186280	KDM4D	1.33555548	0.336208439	1.990012068
ENSG00000116885	OSCP1	1.418107335	0.356996439	1.989985144
ENSG00000081059	TCF7	3.763875059	0.94755206	1.989941624
ENSG00000114854	TNNC1	11.42553099	2.876753822	1.989747542
ENSG00000173706	HEG1	4.994602921	1.257961481	1.989282239
ENSG00000228672	PROB1	0.585475	0.147506	1.988833929
ENSG00000184113	CLDN5	0.062637498	0.015782758	1.988677266
ENSG00000237188	RP11-337C18.8	0.53400189	0.13462836	1.987862492
ENSG00000251455	RP11-164P12.3	0.334546	0.084399	1.986906784
ENSG00000273682	RP11-427P5.3	1.096833	0.276896	1.985927765
ENSG00000276073	RP5-1125A11.7	0.946401	0.239074	1.984994357
ENSG00000031081	ARHGAP31	1.381531194	0.349174035	1.984249949
ENSG00000137434	C6orf52	2.084883635	0.527735581	1.982079701
ENSG00000272525	RP11-79P5.9	0.545903	0.138211	1.981772183
ENSG00000213077	FAM106A	0.177835	0.04508	1.979979871
ENSG00000225813	AC009299.4	0.887312	0.224936	1.979926903
ENSG00000164506	STXBP5	2.223110302	0.563572116	1.979907394
ENSG00000235884	LINC00941	1.351871728	0.342826186	1.979409054
ENSG00000240549	THOC7-AS1	0.266319	0.06755	1.979127684
ENSG00000279879	RP11-1072C15.6	0.057574	0.014606	1.978856312
ENSG00000278175	GLIDR	0.492647924	0.125040314	1.978163668
ENSG00000161267	BDH1	1.134646156	0.288104397	1.977578873
ENSG00000241286	RPL29P25	0.313361	0.079589	1.977184683
ENSG00000243018	RP5-1070G24.2	0.985802	0.250558	1.976153306
ENSG00000272006	RP11-583F2.6	1.708658	0.434897	1.974117998
ENSG00000242073	AC006014.7	1.242136	0.316971	1.970400383
ENSG00000254369	HOXA-AS3	0.462042507	0.117909768	1.970342349
ENSG00000196972	SMIM10L2B	1.620964	0.413666	1.970313761
ENSG00000226792	LINC00371	0.016188411	0.004131298	1.970294473
ENSG00000236375	POU5F1P5	0.1547	0.03948	1.970279302
ENSG00000271057	RP11-747H12.7	0.139061	0.035489	1.970274041
ENSG00000261330	RP11-281J9.1	0.64393	0.164334	1.970272867
ENSG00000277118	Metazoa_SRP	0.177959	0.045416	1.970272345
ENSG00000232768	RP11-201O14.2	0.312246	0.079687	1.970266801
ENSG00000140798	ABCC12	0.013652642	0.00348438	1.970206319
ENSG00000265688	MAFG-AS1	2.116584542	0.540348	1.969777365
ENSG00000181830	SLC35C1	4.921436061	1.256605179	1.96954792
ENSG00000247982	LINC00926	0.365024666	0.093224672	1.969210233

ENSG00000092200	RPGRIP1	0.101467002	0.025916845	1.969048639
ENSG00000180891	CUEDC1	13.56658735	3.465501452	1.968921831
ENSG00000172955	ADH6	0.178193615	0.045537847	1.968307666
ENSG00000204175	GPRIN2	0.537927526	0.137501842	1.967960865
ENSG00000139970	RTN1	0.204574747	0.052298414	1.967788957
ENSG00000179909	ZNF154	0.184017702	0.047085732	1.966482695
ENSG00000226137	BAIAP2-AS1	5.496355146	1.408147725	1.964676535
ENSG00000223813	AC007255.8	0.880080347	0.225501556	1.964497856
ENSG00000010319	SEMA3G	3.558976364	0.912413576	1.963702534
ENSG00000145431	PDGFC	8.50470454	2.180638565	1.96351045
ENSG00000220418	TUBB3P1	0.186849	0.047944	1.962450658
ENSG00000196453	ZNF777	4.810345	1.235079	1.961537044
ENSG00000224540	RP11-385M4.3	0.96858	0.24879	1.960942715
ENSG00000264219	MIR4442	1.823148	0.468506	1.960292252
ENSG00000268129	RP11-91G21.1	1.057843	0.27205	1.959181792
ENSG00000137747	TMPRSS13	0.159696855	0.041078509	1.958880194
ENSG00000134986	NREP	3.095880096	0.797074946	1.957562311
ENSG00000126895	AVPR2	0.222278799	0.057250022	1.957022186
ENSG00000260277	RP11-101E7.2	1.015936885	0.261687556	1.956893549
ENSG00000128253	RFPL2	0.347767221	0.08960363	1.956492869
ENSG00000140839	CLEC18B	0.158773503	0.040981503	1.953925377
ENSG00000163840	DTX3L	4.123911	1.064826	1.953395494
ENSG00000241634	RP11-381E24.1	3.417745	0.882515	1.953352056
ENSG00000251794	RNU6-237P	2.007174	0.518337	1.953203403
ENSG00000251432	RP11-420A23.1	0.533504288	0.137999444	1.950837412
ENSG00000279245	FAM223A	0.21415	0.055411	1.950377369
ENSG00000182810	DDX28	2.92766	0.757538	1.950357853
ENSG00000167100	SAMD14	0.235958237	0.061055976	1.950327114
ENSG00000177494	ZBED2	7.24748	1.87724	1.94886634
ENSG00000234373	SNX18P7	0.274118	0.071025	1.948398235
ENSG00000275632	RP5-967N21.11	3.979423	1.031801	1.947394511
ENSG00000173451	THAP2	0.92187596	0.239107514	1.946913179
ENSG00000278291	RP11-172H24.4	0.850654	0.2208	1.945832269
ENSG00000247763	RP11-447H19.1	1.045627	0.271609	1.944765109
ENSG00000128710	HOXD10	0.309538327	0.080417073	1.944544328
ENSG00000204923	FBXO48	1.122843	0.291712	1.944539579
ENSG00000233101	HOXB-AS3	3.108386599	0.807876496	1.943959284
ENSG00000187792	ZNF70	2.12493	0.552461	1.943470788
ENSG00000198513	ATL1	1.25892055	0.327363415	1.943222232
ENSG00000240040	AC096579.13	0.575773575	0.149788799	1.94257183
ENSG00000225756	DBH-AS1	1.808887	0.470658	1.942351264
ENSG00000137460	FHDC1	1.728602142	0.449841662	1.942116668
ENSG00000271698	GS1-393G12.13	0.199403	0.051924	1.941213683
ENSG00000259162	RP11-203M5.6	0.050280514	0.013093718	1.941124617
ENSG00000259553	AC140725.4	0.382871146	0.099726997	1.940802932
ENSG00000234817	RP3-400B16.1	0.089409	0.023293	1.9405236
ENSG00000228436	RP5-864K19.4	0.208590676	0.054342664	1.940517478
ENSG00000005421	PON1	0.126162068	0.032869856	1.940441173

ENSG00000272763	RP11-357H14.17	6.228088608	1.622711505	1.940382938
ENSG00000139531	SUOX	3.412946084	0.889479483	1.939984391
ENSG00000272625	RP11-737O24.5	0.288388	0.075167	1.939839804
ENSG00000272872	LL22NC03-N14H11.1	2.393002	0.623868	1.939508886
ENSG00000103042	SLC38A7	2.617920452	0.682917029	1.938639047
ENSG00000248817	PMPCAP1	0.383013	0.09993	1.9384036
ENSG00000139083	ETV6	5.509369486	1.438573014	1.937248774
ENSG00000248592	TMEM110-MUSTN1	2.184870064	0.570551529	1.93711839
ENSG00000100302	RASD2	3.021552	0.789284	1.936673362
ENSG00000163637	PRICKLE2	0.115349654	0.03014203	1.936165216
ENSG00000283122	HYMAI	0.160885	0.042042	1.936126616
ENSG00000157335	CLEC18C	0.121855415	0.031848132	1.935889681
ENSG00000167528	ZNF641	0.762134179	0.199360584	1.934664819
ENSG00000230163	RP5-997D16.2	1.724068	0.451005	1.934601345
ENSG00000152683	SLC30A6	8.056960318	2.108620825	1.933935959
ENSG00000100365	NCF4	0.225543856	0.059074134	1.9328095
ENSG00000197980	LEKR1	0.279058119	0.073116885	1.932289115
ENSG00000204104	TRAF3IP1	7.099471142	1.862125721	1.930761079
ENSG00000181938	GINS3	3.746974895	0.983392681	1.929886789
ENSG00000176697	BDNF	16.15044528	4.23913726	1.929731356
ENSG00000183111	ARHGEF37	2.511145129	0.659730272	1.928397201
ENSG00000111671	SPSB2	2.183168307	0.574992462	1.924808409
ENSG00000229562	ZFYVE9P1	0.874053	0.230211	1.924763993
ENSG00000124813	RUNX2	3.987406584	1.051170429	1.923454124
ENSG00000135437	RDH5	0.137631958	0.036285987	1.923331104
ENSG00000131781	FMO5	0.266631861	0.070321332	1.922814888
ENSG00000177706	FAM20C	4.554380669	1.202133003	1.921658357
ENSG00000237934	RP11-467D18.2	0.529478	0.139788	1.921330222
ENSG00000258592	RP11-108M12.3	0.38727868	0.102269065	1.92100227
ENSG00000230659	RP11-466F5.9	0.555919	0.14681	1.92092445
ENSG00000108511	HOXB6	8.578989068	2.26757801	1.91965547
ENSG00000089225	TBX5	0.139751455	0.036964772	1.91864039
ENSG00000182612	TSPAN10	0.470307691	0.124551468	1.916862897
ENSG00000266265	KLF14	0.229726	0.060878	1.915921279
ENSG00000281490	CICP14	1.514135	0.401278	1.915819874
ENSG00000157423	HYDIN	0.008719049	0.002312052	1.914996819
ENSG00000267530	AC006273.5	0.67393608	0.178722871	1.914887497
ENSG00000233839	RP11-389O22.4	0.414618	0.110001	1.914266111
ENSG00000136197	C7orf25	3.154791631	0.837068466	1.914127186
ENSG00000166548	TK2	0.891449555	0.23661349	1.913620833
ENSG00000033100	CHPF2	6.355205013	1.687723768	1.91285987
ENSG00000244036	RP11-306G20.1	0.132911818	0.035303978	1.912566738
ENSG00000070808	CAMK2A	0.016551853	0.004399813	1.911478594
ENSG00000238755	RP11-23D24.2	0.036147906	0.009609219	1.911421039
ENSG00000235500	SNX19P2	0.057104	0.01518	1.911420016
ENSG00000270540	RP11-785G17.1	0.131941	0.035074	1.911419068
ENSG00000091831	ESR1	0.01493791	0.003970975	1.911413072
ENSG00000240184	PCDHGC3	0.037478927	0.009963108	1.911411836

ENSG00000270863	DDX55P1	0.071989	0.019137	1.911411794
ENSG00000230731	RP11-478K15.6	0.061027	0.016223	1.91140704
ENSG00000282968	AGGF1P10	0.077432	0.020584	1.911406544
ENSG00000164175	SLC45A2	0.062315254	0.016565542	1.911399974
ENSG00000168497	SDPR	0.126097	0.033521	1.911396859
ENSG00000004948	CALCR	0.05036783	0.013389542	1.911395978
ENSG00000279199	RP11-749I16.3	0.091376	0.024291	1.911393406
ENSG00000279044	AC007787.3	0.339875	0.090351	1.911391772
ENSG00000245869	RP11-158I9.5	0.123685	0.03288	1.911388343
ENSG00000279873	LINC01126	0.123685	0.03288	1.911388343
ENSG00000238741	SCARNA7	0.616924	0.164001	1.91138816
ENSG00000272931	RP5-943J3.2	0.292928	0.077871	1.911388044
ENSG00000274918	RP11-54G14.1	0.449415	0.119471	1.911387812
ENSG00000272444	RP11-1017G21.6	0.314174	0.083519	1.91138745
ENSG00000276772	CTD-2515H24.2	0.117952	0.031356	1.911386446
ENSG00000249279	CTC-436P18.3	0.19651027	0.052239691	1.911386443
ENSG00000261379	RP11-395N3.1	0.198426	0.052749	1.911385427
ENSG00000237458	TUBB4BP3	0.154004	0.04094	1.911384816
ENSG00000245685	AF146191.4	0.088361479	0.023489781	1.911384318
ENSG00000271916	RP11-884K10.6	0.795253	0.211408	1.911383938
ENSG00000137673	MMP7	0.17527801	0.046595456	1.911383828
ENSG00000218631	RP3-395C13.1	0.330495	0.087858	1.911382882
ENSG00000244459	RP11-1398P2.1	1.054843	0.280417	1.911382569
ENSG00000267475	CTD-2538C1.2	1.79212	0.476413	1.911382557
ENSG00000226144	RPS27AP3	0.224212	0.059604	1.911382437
ENSG00000236797	SPA17P1	0.225205	0.059868	1.911381878
ENSG00000162894	FCMR	0.086705239	0.023049532	1.911381732
ENSG00000278881	FP325331.1	0.147526	0.039218	1.911381368
ENSG00000179219	LINC00311	0.065002	0.01728	1.911380891
ENSG00000229912	AC128709.4	0.339308	0.090201	1.911380113
ENSG00000101883	RHOXF1	0.26474	0.070378	1.911379769
ENSG00000234044	AC108059.1	0.466152	0.123921	1.911379765
ENSG00000171931	FBXW10	0.092749908	0.024656474	1.91137936
ENSG00000181781	ODF3L2	0.118500868	0.031502075	1.91137889
ENSG00000137674	MMP20	0.07980572	0.021215431	1.911378181
ENSG00000263680	RP11-57A1.1	0.2444	0.064971	1.911376469
ENSG00000229162	RP11-84D1.1	0.248274	0.066001	1.911373398
ENSG00000277559	RP11-434E6.4	0.139633	0.03712	1.911371324
ENSG00000271553	RP11-274B21.10	0.365502	0.097165	1.911370664
ENSG00000233533	MKNK2P1	0.16209	0.04309	1.911370084
ENSG00000261250	RP11-545A16.1	0.061804	0.01643	1.911367733
ENSG00000276255	RP5-881P19.7	0.077057158	0.020484939	1.911365407
ENSG00000253125	RP11-459E5.1	0.092918183	0.024701456	1.91136487
ENSG00000069188	SDK2	0.017539085	0.004662616	1.911362007
ENSG00000229165	GDI2P1	0.076192	0.020255	1.911361441
ENSG00000064195	DLX3	0.074985519	0.019934291	1.91135972
ENSG00000149488	TMC2	0.041253615	0.010966967	1.911355944
ENSG00000139767	SRRM4	0.046085804	0.012251592	1.911353232

ENSG00000281912	LINC01144	0.119055	0.03165	1.911350807
ENSG00000260325	HSPB9	0.105923	0.028159	1.911347882
ENSG00000175262	C1orf127	0.056978756	0.015147471	1.91134721
ENSG00000005379	BZRAP1	0.017930884	0.004767002	1.911292554
ENSG00000143622	RIT1	3.095965224	0.823226664	1.911027649
ENSG00000127325	BEST3	0.088918087	0.023657286	1.910192318
ENSG00000153404	PLEKHG4B	0.122240852	0.032553024	1.908863029
ENSG00000173262	SLC2A14	2.228843677	0.593578251	1.908785298
ENSG00000260936	FTO-IT1	0.332095	0.088504	1.907781436
ENSG00000255521	RP4-607I7.1	0.167277972	0.044580166	1.90777357
ENSG00000203952	CCDC160	0.680701	0.181410067	1.907766711
ENSG00000279518	AC083843.4	0.185797	0.049516	1.907760527
ENSG00000240694	PNMA2	0.655069434	0.174614218	1.9074768
ENSG00000160326	SLC2A6	3.906805953	1.04241573	1.906058841
ENSG00000273058	RP11-385F5.5	1.471584	0.392698	1.90587774
ENSG00000224209	LINC00466	0.039739637	0.010607385	1.905509615
ENSG00000182308	DCAF4L1	0.46822	0.124981	1.905477772
ENSG00000148483	TMEM236	0.111219	0.029714	1.904188536
ENSG00000211890	IGHA2	0.189138	0.050532	1.90417006
ENSG00000266074	BAHCC1	3.974813927	1.062300953	1.903694781
ENSG00000163485	ADORA1	2.173602469	0.581367033	1.902566939
ENSG00000264759	AC005000.1	0.407727	0.109059	1.902494664
ENSG00000206195	DUXAP8	1.216144884	0.325450659	1.901804372
ENSG00000233706	RP5-1087E8.3	1.061113	0.284272	1.90023439
ENSG00000138080	EMILIN1	0.102032846	0.027346966	1.89958094
ENSG00000148488	ST8SIA6	0.494495251	0.132616848	1.898692594
ENSG00000182230	FAM153B	0.028473571	0.00763875	1.89821506
ENSG00000236156	CHCHD4P3	1.601807	0.429988	1.897332026
ENSG00000198053	SIRPA	6.930512917	1.860556784	1.897227706
ENSG00000124508	BTN2A2	2.888282671	0.775449236	1.897107697
ENSG00000207399	RNU6-1011P	2.373571	0.637307	1.896998793
ENSG00000274922	RP11-88E10.5	0.066147	0.017762	1.896881688
ENSG00000184564	SLITRK6	0.186706	0.050135	1.896878263
ENSG00000165140	FBP1	0.120833014	0.032446554	1.896877512
ENSG00000261441	RP11-217B1.2	0.181739	0.048802	1.896855867
ENSG00000241717	VWFP1	0.034666	0.009309	1.896823275
ENSG00000265722	AC099326.1	0.44978	0.120827	1.896276636
ENSG00000265294	AL671883.1	0.44978	0.120827	1.896276636
ENSG00000223742	RP11-149B9.2	0.266362	0.071588	1.895598598
ENSG00000281571	AC241585.2	0.775192	0.208355	1.895509961
ENSG00000125817	CENPB	20.328733	5.465412	1.895118141
ENSG00000124713	GNMT	0.375506	0.100972	1.894880677
ENSG00000259744	RP11-138H8.6	0.532649	0.143365	1.893492294
ENSG00000235109	ZSCAN31	1.776843688	0.478296713	1.893338989
ENSG00000174564	IL20RB	1.868924934	0.503345132	1.892588756
ENSG00000227117	HORMAD2-AS1	0.139627044	0.037609451	1.892411246
ENSG00000197847	SLC22A20	0.183581933	0.049459765	1.892096796
ENSG00000227407	AC008746.3	0.577273	0.155556	1.891819707

ENSG00000255467	RP11-144G7.2	0.126608	0.03412	1.891679016
ENSG00000145192	AHSG	0.170663706	0.046000668	1.891429575
ENSG00000099994	SUSD2	29.89779467	8.06356868	1.890548696
ENSG00000213859	KCTD11	2.220158285	0.598938755	1.890182144
ENSG00000280122	RP11-87G24.2	0.063032	0.017007	1.889955765
ENSG00000240889	NDUFB2-AS1	0.121682	0.03284	1.889589739
ENSG00000276384	RP11-186B7.7	0.47073	0.127044	1.889571558
ENSG00000248196	RP11-476C8.2	0.225063	0.060742	1.889562583
ENSG00000004864	SLC25A13	8.335217158	2.251175487	1.888541262
ENSG00000175785	PRIMA1	1.491577905	0.403261364	1.887052236
ENSG00000183128	CALHM3	0.242157	0.065522	1.885891408
ENSG00000006606	CCL26	2.825965547	0.765046884	1.88512381
ENSG00000242207	HOXB-AS4	1.194945659	0.323591067	1.884701325
ENSG00000242474	AC093627.9	0.339426101	0.091937108	1.884378327
ENSG00000241668	RPL19P11	0.106286	0.028791	1.884261773
ENSG00000249846	RP11-77P16.4	0.252884474	0.068542008	1.883418111
ENSG00000187952	HS6ST1P1	3.19153	0.865315	1.882950892
ENSG00000143578	CREB3L4	2.173830615	0.589636205	1.882342512
ENSG00000125872	LRRN4	0.148227	0.040208	1.882253781
ENSG00000224207	RP11-10L12.1	0.391203	0.106118	1.882248043
ENSG00000181333	HEPHL1	0.074655	0.020251	1.882245737
ENSG00000196273	LINC00523	0.120515126	0.032691276	1.882236639
ENSG00000198429	ZNF69	0.068069859	0.018465	1.882222895
ENSG00000280053	RP11-61K12.2	0.059344	0.016098	1.882220713
ENSG00000176383	B3GNT4	1.045963312	0.283734929	1.882216578
ENSG00000131435	PDLIM4	0.062504781	0.016955575	1.882206866
ENSG00000181896	ZNF101	1.619408519	0.439310289	1.882154776
ENSG00000259515	RP11-365N19.2	0.481774	0.130726	1.881810431
ENSG00000198093	ZNF649	0.823400718	0.223493162	1.881364012
ENSG00000170476	MZB1	0.087707809	0.023810403	1.881113226
ENSG00000226251	RP11-15I11.3	0.27290689	0.074137742	1.880128746
ENSG00000138867	GUCD1	6.413011957	1.743573585	1.878954845
ENSG00000187808	SOWAHD	0.256449	0.069743	1.878551617
ENSG00000250405	RP11-114H7.2	0.104082	0.028306	1.878540792
ENSG00000233611	AC079135.1	0.206965324	0.056304969	1.878054927
ENSG00000263590	RP11-403I13.7	0.436318	0.118708	1.877962828
ENSG00000231074	HCG18	2.186320329	0.594941302	1.877685552
ENSG00000272760	RP11-5C23.1	1.858266	0.5058	1.877318088
ENSG00000278512	MIR6721	0.787881	0.214481	1.877127893
ENSG00000280129	RP11-298I3.3	0.48923	0.13322	1.876702187
ENSG00000164627	KIF6	0.031793703	0.008664173	1.875607098
ENSG00000272265	CTD-2287O16.4	0.430575	0.117398	1.874856726
ENSG00000117069	ST6GALNAC5	0.465622901	0.126954148	1.874854485
ENSG00000135973	GPR45	0.151871	0.041409	1.874830143
ENSG00000033011	ALG1	2.791087761	0.761374915	1.874148545
ENSG00000114805	PLCH1	1.915839067	0.522719848	1.873866532
ENSG00000270711	RP11-46A10.8	0.279867	0.076375	1.873569004
ENSG00000180423	HARBI1	0.893647648	0.24396933	1.873006315

ENSG00000270558	CTD-2124B8.2	1.131454	0.308968	1.872648602
ENSG00000166866	MYO1A	0.08922289	0.024389414	1.871158768
ENSG00000152086	TUBA3E	0.064197	0.017549	1.871117057
ENSG00000259538	UBE2Q2P11	0.041379	0.011314	1.870789704
ENSG00000186666	BCDIN3D	3.872701014	1.059070046	1.870542113
ENSG00000259422	RP11-593F23.1	0.084057	0.02299	1.870361501
ENSG00000229180	GS1-124K5.11	1.015807283	0.277885816	1.870062623
ENSG00000177406	RP11-218M22.1	0.336233297	0.091990131	1.869911596
ENSG00000224717	RP11-576D8.4	0.193353	0.052901	1.86987025
ENSG00000116793	PHTF1	3.137771276	0.858540424	1.869782222
ENSG00000268278	RP11-420K14.1	0.289348	0.079182	1.869561259
ENSG00000202025	RNU6-1240P	0.065663	0.017973	1.869249426
ENSG00000105011	ASF1B	26.72011426	7.315929221	1.868813155
ENSG00000177732	SOX12	10.455035	2.862644	1.868777719
ENSG00000250015	CTC-339F2.2	0.441049	0.120851	1.867709535
ENSG00000260360	RP11-533E19.5	0.157102	0.043055	1.867448854
ENSG00000261996	CTC-281F24.1	0.521121	0.142844	1.867177955
ENSG00000103343	ZNF174	3.161602734	0.866708138	1.867037944
ENSG00000214980	RP11-274J2.1	1.390912	0.38132	1.86695704
ENSG00000186451	SPATA12	0.482597	0.132329	1.866689683
ENSG00000271141	RP11-171I2.4	1.088813	0.299008	1.864500208
ENSG00000259153	RP6-65G23.3	2.82800963	0.776650999	1.864448683
ENSG00000244259	AP000797.1	0.39012	0.107169	1.864030314
ENSG00000224438	RPL23AP14	0.125428	0.034462	1.863781109
ENSG00000253508	RP1-170O19.14	0.587889	0.161583	1.863268362
ENSG00000176268	CYCSP34	0.647364	0.178028	1.862472975
ENSG00000137135	ARHGEF39	3.183337663	0.875630731	1.862145701
ENSG00000114670	NEK11	0.43643817	0.120066051	1.861949001
ENSG00000258646	RP11-950C14.3	0.402216	0.110675	1.861641099
ENSG00000267113	AF038458.4	1.251037	0.344278	1.86147856
ENSG00000240766	PLCXD2-AS1	0.400459	0.110225	1.861203066
ENSG00000206888	RNU6-48P	0.16218	0.044666	1.860346951
ENSG00000180353	HCLS1	0.077148843	0.021252445	1.860015728
ENSG00000262165	RP11-81A22.5	0.349571	0.0963	1.859977802
ENSG00000147485	PXDNL	0.037506153	0.01033286	1.859887687
ENSG00000197362	ZNF786	3.472649035	0.956994769	1.859453668
ENSG00000268756	AC104534.2	4.973748	1.371181	1.85891439
ENSG00000219435	TEX40	0.568999491	0.156871204	1.858846814
ENSG00000084636	COL16A1	0.492822768	0.135929152	1.858214009
ENSG00000235070	AC068138.1	1.806346	0.498252	1.858126762
ENSG00000231852	CYP21A2	0.229293823	0.063255339	1.857938332
ENSG00000186272	ZNF17	1.487381075	0.410764163	1.856392098
ENSG00000198736	MSRB1	8.152808444	2.251573205	1.856363734
ENSG00000119917	IFIT3	1.038745382	0.287003127	1.855703701
ENSG00000252690	SCARNA15	0.4716	0.130338	1.855305956
ENSG00000228084	RP5-884G6.2	0.298938	0.082627	1.855161106
ENSG00000169570	DTWD2	1.883344168	0.520603857	1.85503876
ENSG00000219902	RPL35P3	0.137187	0.037928	1.854808573

ENSG00000276151	MIR6773	0.268923	0.074349	1.854807906
ENSG00000156398	SFXN2	1.260339408	0.348630262	1.854042594
ENSG00000255062	RP11-712L6.5	1.403525825	0.388313141	1.853763172
ENSG00000272102	RP3-355L5.5	0.361259	0.100037	1.852499831
ENSG00000171189	GRIK1	0.046061057	0.012755067	1.852477016
ENSG00000238062	SPATA3-AS1	0.05292207	0.014655138	1.852462952
ENSG00000258498	DIO3OS	0.034869522	0.009656148	1.852446844
ENSG00000280183	RP11-400F19.12	1.09403	0.30348	1.849978952
ENSG00000278002	RP11-596C23.2	0.298061	0.082754	1.848706664
ENSG00000083828	ZNF586	1.296446357	0.360017121	1.848425093
ENSG00000239415	AP001469.9	0.238695707	0.066303994	1.848004938
ENSG00000185621	LMLN	3.027398785	0.841213815	1.847534279
ENSG00000230658	KLHL7-AS1	0.455763	0.126647	1.847470905
ENSG00000061337	LZTS1	3.432835487	0.954365	1.84678768
ENSG00000215151	ABCD1P2	0.121545	0.033804	1.846224672
ENSG00000134802	SLC43A3	5.965093087	1.660948754	1.84453709
ENSG00000279208	bP-21264C1.1	1.32018	0.36784	1.843584371
ENSG00000129514	FOXA1	0.485699191	0.135369294	1.843162557
ENSG00000174365	SNHG11	1.726442582	0.481214805	1.84304942
ENSG00000224557	HLA-DPB2	0.18195408	0.050738508	1.842421414
ENSG00000274911	RP11-12A20.7	0.882719	0.246228	1.841959423
ENSG00000267655	CTD-2286N8.2	0.543178	0.151598	1.841174328
ENSG00000270127	RP11-526I2.5	2.120142	0.593007	1.838039855
ENSG00000262664	OVCA2	3.619108	1.012539	1.837656683
ENSG00000181004	BBS12	1.447398141	0.404999033	1.837471457
ENSG00000243537	CTC-458A3.1	0.237016	0.066325	1.837359777
ENSG00000189337	KAZN	2.808583668	0.786924287	1.835546041
ENSG00000132773	TOE1	2.847251179	0.7978424	1.835394071
ENSG00000278952	RP11-399J13.2	0.698078	0.195698	1.834759234
ENSG00000257594	GALNT4	3.000793	0.841429	1.834430356
ENSG00000281453	TGFB2-OT1	0.562606	0.157901	1.833104631
ENSG00000157870	FAM213B	12.0273309	3.375773296	1.833026589
ENSG00000141642	ELAC1	2.094165299	0.58796418	1.832575152
ENSG00000137878	GCOM1	1.001605116	0.281246398	1.832407313
ENSG00000178409	BEND3	1.732445757	0.486993559	1.830835586
ENSG00000187866	FAM122A	6.072005	1.707281	1.830472449
ENSG00000243870	RN7SL236P	1.354843	0.381131	1.829766819
ENSG00000163283	ALPP	0.156901628	0.044229925	1.826765624
ENSG00000242082	RP1-90G24.10	1.721295	0.485602	1.825648103
ENSG00000217289	AC018865.5	0.803627	0.226755	1.825391673
ENSG00000258297	RP11-658F2.8	2.384998008	0.673413345	1.824423844
ENSG00000105750	ZNF85	0.548594523	0.154912147	1.824289945
ENSG00000276728	AC142472.6	0.394073	0.111287	1.824177832
ENSG00000278148	AP001453.1	1.085885	0.306994	1.822588959
ENSG00000273047	RP4-583P15.14	0.82862614	0.234308826	1.822310034
ENSG00000236581	STARD13-AS	0.047782769	0.013513438	1.822095662
ENSG00000180044	C3orf80	0.667692237	0.188938357	1.821267651
ENSG00000268858	RP4-591C20.9	1.115357	0.315733	1.820728595

ENSG00000268119	CTD-2561J22.5	0.039561508	0.011203632	1.820130873
ENSG00000282059	CICP19	0.093501	0.026497	1.819152768
ENSG00000164690	SHH	0.151536785	0.042968988	1.81830034
ENSG00000196632	WNK3	0.033263046	0.009432716	1.818175212
ENSG00000245849	RAD51-AS1	2.066634396	0.586061666	1.818158808
ENSG00000133111	RFXAP	2.474624888	0.702206446	1.817242709
ENSG00000276026	RP4-545L17.11	1.350201	0.383246	1.816831553
ENSG00000269825	CTD-3099C6.9	1.44611	0.410655	1.816178528
ENSG00000165501	LRR1	5.78657886	1.64403409	1.815470436
ENSG00000171847	FAM90A1	0.284435361	0.080906124	1.813780014
ENSG00000107099	DOCK8	0.029539142	0.008402456	1.813744891
ENSG00000253878	RP11-347C18.3	0.516659	0.14697	1.813690704
ENSG00000167670	CHAF1A	16.78355252	4.775350422	1.813369609
ENSG00000125740	FOSB	8.019159317	2.282570884	1.812791338
ENSG00000164900	GBX1	0.510166162	0.145309693	1.811836268
ENSG00000236875	DDX11L5	0.096841	0.027584	1.811786297
ENSG00000256001	RP11-575F12.2	1.23880599	0.352997881	1.811218834
ENSG00000229320	KRT8P12	0.379200294	0.108082584	1.810826013
ENSG00000279968	GVQW2	0.751102858	0.214094885	1.810760162
ENSG00000276570	CTD-2587H24.14	1.102322	0.314286	1.810395799
ENSG00000075891	PAX2	2.498122603	0.71235898	1.810167934
ENSG00000270521	RP11-24C14.1	0.74781	0.213342	1.809503751
ENSG00000103355	PRSS33	0.904661752	0.258243458	1.808646673
ENSG00000273032	DGCR9	0.153848	0.043924	1.808424343
ENSG00000262554	LA16c-360H6.2	0.911259	0.260195	1.808267919
ENSG00000179292	TMEM151A	3.96601	1.133532	1.806863196
ENSG00000252581	RNU6-1098P	1.972228	0.563755	1.806686116
ENSG00000276603	RP11-425M5.7	0.451871	0.129166	1.806684608
ENSG00000184774	MGAT4EP	0.033689085	0.00963	1.80667356
ENSG00000125912	NCLN	20.93736216	5.990282469	1.805383754
ENSG00000248866	USP46-AS1	1.08271	0.309851	1.805000344
ENSG00000259802	CTD-2256P15.2	0.885741	0.253483	1.804995906
ENSG00000274553	AC138035.3	0.145941	0.041778	1.80456991
ENSG00000143633	C1orf131	3.978715425	1.139149824	1.804345209
ENSG00000160818	GPATCH4	5.65438408	1.619203727	1.804085367
ENSG00000205835	GMNC	0.505750774	0.144833181	1.804034459
ENSG00000196860	TOMM20L	0.194893	0.055866	1.802639842
ENSG00000059377	TBXAS1	0.176982414	0.050745971	1.802240818
ENSG00000246877	DNM1P35	0.348124	0.099821	1.802186016
ENSG00000237438	CECR7	1.650645351	0.473578003	1.801356206
ENSG00000042286	AIFM2	6.471829908	1.856919617	1.801262327
ENSG00000162415	ZSWIM5	0.405571596	0.116369413	1.801244712
ENSG00000123405	NFE2	0.305501112	0.087680356	1.800852071
ENSG00000178772	CPN2	0.033617	0.009652	1.800291163
ENSG00000250510	GPR162	1.421695626	0.408327031	1.799815646
ENSG00000160094	ZNF362	3.239909617	0.930821177	1.799377629
ENSG00000135747	ZNF670-ZNF695	0.363967934	0.10457686	1.799247698
ENSG00000162913	C1orf145	0.297255577	0.085416684	1.799114084

ENSG00000065675	PRKCQ	1.642888816	0.472566442	1.79764576
ENSG00000254363	CTB-131B5.5	0.183855854	0.052886363	1.797607458
ENSG00000264944	MIR3620	0.640091	0.184166	1.797270282
ENSG00000259635	AC100830.3	0.250371	0.072078	1.796436582
ENSG00000180346	TIGD2	5.101116844	1.469234416	1.795748551
ENSG00000237499	RP11-356I2.4	0.091868267	0.026477789	1.794783975
ENSG00000269313	MAGIX	0.128760559	0.037125097	1.79422405
ENSG00000177352	CCDC71	11.426355	3.294637	1.794173834
ENSG00000162066	AMDHD2	1.920692714	0.554026583	1.793599618
ENSG00000278385	RP11-89H19.2	0.318101	0.091856	1.792039041
ENSG00000197841	ZNF181	0.638749353	0.184489902	1.791708075
ENSG00000253704	RP11-267M23.4	0.196871	0.056873	1.791434799
ENSG00000277969	CTB-58E17.1	0.812655	0.234774	1.791370362
ENSG00000217327	RPS7P5	0.080593	0.023288	1.791067793
ENSG00000081665	ZNF506	0.178635946	0.05162939	1.790757953
ENSG00000254610	RP5-903G2.2	0.602964	0.174357	1.790027583
ENSG00000228315	GUSBP11	1.910852413	0.552780076	1.789438833
ENSG00000186638	KIF24	2.037008	0.589285	1.789414198
ENSG00000280143	AP000892.6	0.806305	0.233277	1.789281597
ENSG00000223181	RNU6-1199P	0.575519	0.166525	1.789124776
ENSG00000153790	C7orf31	1.023323313	0.296203597	1.788600961
ENSG00000168496	FEN1	36.0055951	10.42634837	1.787987142
ENSG00000172936	MYD88	5.487109444	1.589668024	1.787320838
ENSG00000162728	KCNJ9	0.134258	0.038935	1.785868525
ENSG00000280184	AL023806.1	0.270072	0.078322	1.785854564
ENSG00000155962	CLIC2	0.197132815	0.057169442	1.785851839
ENSG00000207773	MIR642A	0.923477	0.267813	1.785850037
ENSG00000185730	ZNF696	1.97792175	0.573707788	1.785597344
ENSG00000228992	RPL5P32	0.282949	0.082094	1.78519335
ENSG00000260927	RP11-459F6.3	0.232717	0.067534	1.784890688
ENSG00000087303	NID2	0.058339518	0.01693378	1.784569435
ENSG00000262700	RP11-266L9.3	0.177551	0.051543	1.784385071
ENSG00000243251	PGBD3	1.689964	0.490796	1.783797118
ENSG00000250081	CTD-2116N20.1	0.744619	0.216253	1.783782282
ENSG00000269096	CT45A3	0.112556429	0.032697111	1.783413364
ENSG00000276085	CCL3L3	0.126807292	0.036842042	1.78321277
ENSG00000207304	SNORA8	0.771436	0.224169	1.782959689
ENSG00000255670	RP11-253I19.3	0.370604	0.107716	1.782645891
ENSG00000137310	TCF19	7.579092737	2.203157573	1.782452477
ENSG00000233901	LINC01503	1.254502384	0.364844471	1.781761716
ENSG00000133488	SEC14L4	6.017615005	1.751358467	1.780717405
ENSG00000229728	RP11-314N13.3	1.16213	0.338253	1.780596828
ENSG00000235313	HM13-IT1	1.566917	0.456072	1.780595256
ENSG00000236882	LINC01554	0.180265362	0.052518655	1.779220338
ENSG00000267281	RP11-793H13.10	1.37968428	0.40238361	1.777694717
ENSG00000278834	RP11-458J1.1	1.568269	0.457679	1.776765039
ENSG00000135414	GDF11	3.236096	0.945078	1.775749097
ENSG00000260896	PRCAT47	0.218985989	0.063971559	1.775336008

ENSG00000261266	CTD-2196E14.5	0.28893	0.084431	1.774875302
ENSG00000277161	PIGW	4.357543717	1.274221099	1.773899507
ENSG00000166793	YPEL4	0.433847166	0.12690583	1.773428563
ENSG00000279332	RP11-799B12.1	3.593504	1.05139	1.773093374
ENSG00000269858	EGLN2	3.773036139	1.106130189	1.770204721
ENSG00000109705	NKX3-2	0.395395	0.115932	1.770015788
ENSG00000228649	AC005682.5	3.433237842	1.006875281	1.769684814
ENSG00000184524	CEND1	0.54816	0.160819	1.769159192
ENSG00000266644	RP11-927P21.2	0.653609	0.191787	1.768922916
ENSG00000198298	ZNF485	1.307971166	0.383833871	1.768776808
ENSG00000227832	ST13P21	0.038628	0.011337	1.768608058
ENSG00000258640	RPL21P5	0.20303	0.059625	1.767703652
ENSG00000138083	SIX3	1.091024	0.320476	1.767394613
ENSG00000270228	RP11-453E17.3	0.155939	0.045825	1.766775002
ENSG00000274425	AC114271.2	1.752066	0.514915	1.766650919
ENSG00000261586	RP11-923I11.6	0.137163	0.040319	1.766359602
ENSG00000272405	RP11-284F21.10	20.467367	6.01776	1.766027045
ENSG00000283073	RP11-834C11.15	0.063688	0.018727	1.765901764
ENSG00000254726	MEX3A	4.89579	1.43962	1.765853626
ENSG00000141314	RHBDL3	3.519475429	1.035568229	1.764937805
ENSG00000178654	PPIAP33	0.375072	0.11047	1.763512932
ENSG00000232300	FAM215B	2.024773	0.596365	1.76349268
ENSG00000251453	HAUS1P1	0.878653	0.258894	1.762931994
ENSG00000234667	ACTBP13	0.118839	0.035037	1.762057214
ENSG00000166148	AVPR1A	0.092352	0.027228	1.76205219
ENSG00000105997	HOXA3	1.774863509	0.523530478	1.76136265
ENSG00000263002	ZNF234	0.796955911	0.23522207	1.760476487
ENSG00000260804	PKI55	7.736935	2.28379	1.760332156
ENSG00000116560	SFPQ	51.97574403	15.34619975	1.759957067
ENSG00000253415	RP11-44N12.4	0.142833	0.042189	1.75939054
ENSG00000227002	SNRPEP10	0.167178	0.04938	1.759386265
ENSG00000278903	CH507-145C22.1	0.322371824	0.095250776	1.758922906
ENSG00000174276	ZNHIT2	3.496492	1.033512	1.758353064
ENSG00000279970	RP11-299G20.5	0.459164	0.135777	1.75777042
ENSG00000237783	RP11-306I1.1	0.095312	0.028188	1.757576745
ENSG00000213069	KRT8P40	0.37283	0.110264	1.757556109
ENSG00000121390	PSPC1	13.48000124	3.987242068	1.757357529
ENSG00000071909	MYO3B	0.071847462	0.021254695	1.757155627
ENSG00000068976	PYGM	0.330384833	0.097757438	1.756869077
ENSG00000175877	WBSCR28	0.561577121	0.166183163	1.756709941
ENSG00000125384	PTGER2	0.665170172	0.196961262	1.755811568
ENSG00000275160	RP11-187C18.3	1.512456	0.447942	1.755509325
ENSG00000005801	ZNF195	7.622799804	2.258155511	1.755176144
ENSG00000214717	ZBED1	3.749728766	1.11086564	1.75510191
ENSG00000142513	ACPT	0.119304	0.035357	1.75457464
ENSG00000225630	MTND2P28	2406.317139	713.616333	1.753606253
ENSG00000131849	ZNF132	0.762844976	0.226403018	1.752496719
ENSG00000261226	RP11-830F9.7	0.159084	0.047218826	1.752354677

ENSG00000230606	AC159540.1	3.623313865	1.075719216	1.752008228
ENSG00000231177	LINC00852	0.686026	0.203767	1.751342828
ENSG00000270001	RP11-218C14.8	0.371096	0.110225	1.751340974
ENSG00000166603	MC4R	0.218738	0.064971	1.751336057
ENSG00000140092	FBLN5	2.629931621	0.78161547	1.750494363
ENSG00000272787	KB-226F1.2	0.790695	0.235094	1.749883582
ENSG00000227252	AC105760.2	0.388733	0.115593	1.74972555
ENSG00000136531	SCN2A	0.261206518	0.077682178	1.749535341
ENSG00000080573	COL5A3	0.157438829	0.046824531	1.749454959
ENSG00000120149	MSX2	21.85800687	6.501140512	1.749397113
ENSG00000131620	ANO1	28.15469656	8.379421393	1.748453069
ENSG00000260918	RP11-731J8.2	0.021813	0.006494	1.748008912
ENSG00000231735	RP11-1O7.1	0.041125	0.012244	1.747940729
ENSG00000207780	MIR648	0.541449	0.16121	1.747884216
ENSG00000243978	RGAG1	0.164223597	0.048941951	1.746517925
ENSG00000244694	PTCHD4	0.964638758	0.287486196	1.746496092
ENSG00000263335	AF001548.5	2.235259	0.667868	1.74280711
ENSG00000079999	KEAP1	10.70990549	3.200847041	1.742420108
ENSG00000198416	ZNF658B	1.817764602	0.543639854	1.741442257
ENSG00000185432	METTL7A	1.011691551	0.303011617	1.739324492
ENSG00000206706	Y_RNA	3.547976	1.063469	1.738218271
ENSG00000171466	ZNF562	2.713346479	0.813542101	1.73778437
ENSG00000251520	RP11-46A10.6	0.668555	0.200559	1.737019543
ENSG00000230757	NFU1P1	0.920604	0.276197	1.736883062
ENSG00000247402	CTD-2340E1.2	0.461285	0.138415	1.736658084
ENSG00000149639	SOGA1	4.498045458	1.34976251	1.736592654
ENSG00000155846	PPARGC1B	2.218385774	0.665745392	1.736467827
ENSG00000122952	ZWINT	21.40511849	6.432470751	1.734510925
ENSG00000131379	C3orf20	0.216025224	0.06493034	1.734235116
ENSG00000224061	AC092106.1	2.530875	0.760725	1.734189332
ENSG00000169105	CHST14	3.244883	0.975502	1.733949724
ENSG00000261524	ABCB10P3	0.168166	0.050556	1.733931822
ENSG00000248428	CTC-551A13.1	0.072292	0.021741	1.733417704
ENSG00000251595	ABCA11P	1.094946944	0.329379319	1.733039086
ENSG00000226472	RP11-551L14.4	0.116464147	0.035075371	1.73135564
ENSG00000204876	AC021218.2	0.354253	0.106704	1.731165811
ENSG00000244306	LINC01296	1.078045116	0.324723937	1.731131912
ENSG00000226007	RP11-211N8.2	0.432708448	0.130359074	1.730904274
ENSG00000281852	LINC00891	0.074246	0.022369	1.73081254
ENSG00000090530	P3H2	0.813966051	0.245312899	1.73034553
ENSG00000266163	AC007216.1	10.589989	3.192225	1.730066844
ENSG00000126003	PLAGL2	9.012421	2.717122	1.729835364
ENSG00000144792	ZNF660	0.052357373	0.015788141	1.729551388
ENSG00000229043	AC091729.9	1.982887388	0.598176772	1.728958952
ENSG00000127580	WDR24	2.183079624	0.65887077	1.72829732
ENSG00000101977	MCF2	0.035859545	0.010832168	1.727035184
ENSG00000239686	RP11-665C16.1	0.514812	0.155519	1.726954835
ENSG00000224331	AC019181.3	0.577761	0.174582	1.726568003

ENSG00000230071	RPL4P6	0.549294	0.166014	1.726273624
ENSG00000183578	TNFAIP8L3	0.712367	0.215334	1.726044555
ENSG00000225189	REREP1Y	0.599787	0.181312	1.725975841
ENSG00000235014	REREP2Y	0.593789	0.179499	1.725974561
ENSG00000232775	BMS1P22	0.310198029	0.093792395	1.725646666
ENSG00000233223	AC113189.5	1.483803098	0.448688174	1.725514594
ENSG00000254114	HMGN1P28	0.152409	0.046091	1.725391125
ENSG00000010539	ZNF200	1.224253585	0.37061146	1.723923022
ENSG00000278852	NPPA-AS1_2	0.599482	0.181612	1.722856904
ENSG00000198105	ZNF248	1.273760465	0.385903034	1.722783708
ENSG00000267356	RP11-411B10.3	0.378293	0.114687	1.721802212
ENSG00000257228	RP11-413B19.2	0.028270316	0.008571439	1.72167873
ENSG00000271625	PSMA6P4	0.248574	0.075377	1.721479121
ENSG00000009724	MASP2	0.834291764	0.25309717	1.720860627
ENSG00000178935	ZNF552	1.964715923	0.596179375	1.720502357
ENSG00000253219	KRT18P41	0.423942	0.12865	1.720415444
ENSG00000179859	AC025335.1	0.349773	0.106143	1.720409697
ENSG00000243667	WDR92	2.851341538	0.865348439	1.720287791
ENSG00000256433	RP1-102E24.8	0.538037	0.163292	1.720251276
ENSG00000253680	RP11-59O2.1	5.964995	1.810423	1.72019411
ENSG00000166188	ZNF319	2.156360168	0.654663866	1.719771908
ENSG00000271533	RP3-368A4.6	2.130321	0.646803	1.719672559
ENSG00000258388	PPT2-EGFL8	1.234316415	0.374885181	1.719191573
ENSG00000230513	THAP7-AS1	0.881514792	0.267762188	1.719032535
ENSG00000261654	RP11-96K19.4	0.059528	0.018085	1.718774826
ENSG00000272702	RP11-44N22.3	0.123277761	0.037453226	1.718750654
ENSG00000263412	RP5-890E16.2	0.121470921	0.036904316	1.718749511
ENSG00000203527	Z99756.1	0.091306	0.02774	1.71874188
ENSG00000232208	RP3-477M7.5	1.01214	0.307503	1.718736468
ENSG00000273004	GS1-279B7.2	0.473768	0.143938	1.718733239
ENSG00000236483	MTND2P40	0.211816	0.064353	1.71873226
ENSG00000278134	RP11-12A20.4	0.2964	0.090051	1.718731245
ENSG00000252105	RNU1-143P	0.203353	0.061782	1.718727795
ENSG00000275229	RNU1-68P	0.203353	0.061782	1.718727795
ENSG00000086159	AQP6	0.069462	0.021104	1.718707481
ENSG00000271318	AP000654.5	1.31699	0.400208	1.71842248
ENSG00000261770	CTC-459F4.1	0.848686	0.257907	1.718379948
ENSG00000164296	TIGD6	1.070960295	0.325491064	1.718215155
ENSG00000184058	TBX1	0.558751911	0.16997846	1.716855921
ENSG00000197081	IGF2R	17.36398346	5.282594853	1.716779281
ENSG00000222750	RNU4-46P	1.295272	0.394138	1.716482332
ENSG00000180921	FAM83H	21.718565	6.610308177	1.716139346
ENSG00000245146	LINC01024	0.317378687	0.096627345	1.715701816
ENSG00000254470	AP5B1	4.169729	1.269549	1.715637544
ENSG00000138767	CNOT6L	3.215190988	0.979094635	1.715384222
ENSG00000261326	LINC01355	1.370643	0.417536	1.714880356
ENSG00000162946	DISC1	0.246102341	0.074978818	1.714703397
ENSG00000124749	COL21A1	0.050191581	0.015292554	1.714616066

ENSG00000143167	GPA33	0.111017867	0.033825394	1.714613245
ENSG00000172935	MRGPRF	0.129372476	0.039417837	1.714610208
ENSG00000225787	LSM3P3	2.874138	0.877248	1.712072675
ENSG00000141933	TPGS1	0.670790127	0.204772171	1.711841792
ENSG00000188321	ZNF559	0.938162531	0.286470869	1.711449443
ENSG00000253846	PCDHGA10	2.004128717	0.612124286	1.711078656
ENSG00000269473	CTD-2619J13.19	0.492003	0.150279	1.711023691
ENSG00000113319	RASGRF2	0.115892493	0.035399236	1.710996963
ENSG00000067221	STOML1	0.223564924	0.068311917	1.710484667
ENSG00000217178	RP11-9G10.1	0.149467	0.045671	1.710476711
ENSG00000272070	AC005618.6	0.112527	0.034384	1.710461914
ENSG00000116133	DHCR24	61.77808188	18.88056487	1.71019315
ENSG00000102387	TAF7L	0.26085675	0.07976565	1.709418252
ENSG00000183597	TANGO2	2.226388008	0.68136223	1.708211161
ENSG00000249828	RP11-1281K21.2	0.079488	0.024335	1.707704304
ENSG00000244300	GATA2-AS1	1.798013532	0.55072299	1.707005137
ENSG00000268707	RP11-247A12.7	0.564175	0.172929	1.70596291
ENSG00000229127	AC007038.7	0.321148	0.098445	1.705848473
ENSG00000207650	MIR570	10.515052	3.224915	1.705122944
ENSG00000231982	RP11-573D15.1	0.826337	0.253457	1.704989258
ENSG00000168350	DEGS2	0.189635525	0.058167858	1.70493516
ENSG00000259738	ZNF444P1	0.297179	0.091169	1.704716916
ENSG00000265091	RP11-835E18.5	0.079444	0.024374	1.704595234
ENSG00000227214	HCG15	0.341845	0.104885	1.704533956
ENSG00000111186	WNT5B	0.244963692	0.075177837	1.704188611
ENSG00000110442	COMMD9	2.650908649	0.813641778	1.704021292
ENSG00000262712	RP11-295D4.1	0.26114	0.080161	1.703851048
ENSG00000272821	CTA-384D8.36	3.213073	0.986968	1.702878544
ENSG00000204779	FOXD4L5	0.330435	0.101545	1.702247302
ENSG00000279926	RP3-406P24.5	0.241565	0.074239	1.702162257
ENSG00000280231	LA16c-380F5.3	0.73992	0.227397	1.702156074
ENSG00000254298	CTB-17P3.4	0.710085	0.218228	1.702155511
ENSG00000229388	RP11-442N24__B.1	0.360124319	0.110675779	1.702155497
ENSG00000253116	RP11-648L3.2	0.281761	0.086593	1.702149624
ENSG00000154479	CCDC173	0.157514824	0.048409226	1.70213367
ENSG00000168016	TRANK1	0.456276555	0.140228071	1.70213335
ENSG00000166133	RPUSD2	5.153599036	1.584279188	1.7017537
ENSG00000198574	SH2D1B	0.073769796	0.022686253	1.701211898
ENSG00000235568	NFAM1	0.120831586	0.037215693	1.699014618
ENSG00000040633	PHF23	19.02618515	5.861971083	1.698528566
ENSG00000265660	MIR4664	4.501805	1.387315	1.698208167
ENSG00000255237	RP13-317D12.3	0.528891	0.163013	1.697983404
ENSG00000233542	RP11-547D24.1	0.179726	0.055395	1.697971463
ENSG00000198482	ZNF808	1.222255007	0.376731735	1.697935844
ENSG00000212125	TAS2R15P	0.556129	0.171459	1.697555936
ENSG00000126803	HSPA2	0.696177583	0.21472102	1.69699193
ENSG00000260025	RP11-490M8.1	1.879308	0.579981	1.696123986
ENSG00000199672	RNU4-21P	0.250442	0.077316	1.695637622

ENSG00000275393	RP11-568J23.8	1.50973	0.466207	1.69524799
ENSG00000164342	TLR3	0.596767124	0.184318064	1.694970595
ENSG00000199436	SNORD9	1.68349	0.52039	1.693790007
ENSG00000250765	AC138035.1	0.199852	0.061837	1.692389776
ENSG00000151364	KCTD14	10.74646466	3.325697995	1.692133055
ENSG00000149050	ZNF214	0.6877186	0.212884838	1.691745164
ENSG00000124104	SNX21	1.319656692	0.408536014	1.691627495
ENSG00000165556	CDX2	2.761630874	0.855034528	1.691465914
ENSG00000203499	FAM83H-AS1	4.559081471	1.411595536	1.691416418
ENSG00000221297	AL590708.1	0.631762	0.195616	1.691356785
ENSG00000230638	RP11-486B10.4	0.696929	0.215807	1.691270027
ENSG00000154165	GPR15	0.557744	0.172909	1.689590125
ENSG00000183317	EPHA10	0.867168542	0.268836314	1.689584392
ENSG00000167595	PROSER3	2.806275403	0.870813917	1.68822023
ENSG00000005073	HOXA11	3.452646944	1.071619759	1.687909728
ENSG00000261713	SSTR5-AS1	0.253922702	0.078828239	1.687604925
ENSG00000278156	TSC22D1-AS1	0.073612899	0.02285711	1.687315566
ENSG00000260796	RP11-1348G14.5	0.322773	0.100224	1.687291879
ENSG00000127152	BCL11B	0.354555398	0.1101017	1.687174317
ENSG00000242779	ZNF702P	0.226418709	0.070359888	1.686168074
ENSG00000225077	LINC00337	0.516654701	0.1605951	1.685772521
ENSG00000180806	HOXC9	3.667410309	1.140269468	1.685386882
ENSG00000206557	TRIM71	0.040084	0.012463	1.685375098
ENSG00000127863	TNFRSF19	1.762504208	0.548932202	1.682926823
ENSG00000135315	CEP162	2.115447271	0.659254034	1.682056326
ENSG00000189409	MMP23B	0.07633733	0.02379227	1.681895771
ENSG00000158006	PAFAH2	5.195395958	1.619663107	1.681539945
ENSG00000277170	RP11-295D4.4	0.72466	0.225973	1.681153857
ENSG00000205269	TMEM170B	0.80818	0.252027	1.68109835
ENSG00000278967	RP5-849H19.3	0.083941	0.026182	1.680800344
ENSG00000142961	MOB3C	2.104845276	0.656655691	1.680505172
ENSG00000204611	ZNF616	1.714406465	0.534958145	1.680211271
ENSG00000281280	AC137055.1	2.907159	0.907336	1.679901172
ENSG00000228192	RP11-342M1.3	0.357978524	0.111788648	1.679099348
ENSG00000185220	PGBD2	1.412906266	0.44124434	1.679016082
ENSG00000176678	FOXL1	0.96386124	0.301119451	1.678489566
ENSG00000234025	RBPMS2P1	0.408477	0.127701	1.677485025
ENSG00000163263	C1orf189	0.08943	0.027969	1.676930203
ENSG00000217455	AC091801.1	0.100317147	0.031374445	1.6769064
ENSG00000270069	MIR222HG	1.419217641	0.444115779	1.676088113
ENSG00000244265	SIAH2-AS1	0.392154	0.122723	1.676014661
ENSG00000259826	RP11-467D6.1	0.13624	0.042637	1.675972503
ENSG00000136842	TMOD1	7.636540758	2.391231311	1.675165573
ENSG00000137275	RIPK1	9.006734557	2.821823783	1.674376246
ENSG00000100294	MCAT	6.988910189	2.190239043	1.673979173
ENSG00000266575	BOLA2P1	0.972586	0.304817	1.673882459
ENSG00000271672	DUXAP8	1.342364	0.420839	1.673435617
ENSG00000163131	CTSS	0.203199711	0.063736211	1.672713202

ENSG00000159792	PSKH1	4.028185337	1.263941298	1.672200602
ENSG00000228463	AP006222.2	0.442725351	0.138965433	1.671685919
ENSG00000207857	AC097639.1	22.932198	7.199382	1.671429666
ENSG00000267800	RP11-49K24.5	0.745732	0.234294	1.670337241
ENSG00000179546	HTR1D	2.954334	0.928544	1.669790758
ENSG00000109667	SLC2A9	0.142040986	0.044646148	1.669699649
ENSG00000166801	FAM111A	8.795403966	2.764698327	1.669627773
ENSG00000239213	NCK1-AS1	2.524941938	0.793818861	1.669368468
ENSG00000262730	RP11-1099M24.7	1.978488	0.622108	1.669161351
ENSG00000233543	CHTF8P1	1.562556	0.491334	1.669131914
ENSG00000232662	LDHBP1	0.064587	0.020314	1.668769464
ENSG00000188070	C11orf95	2.36430381	0.743746931	1.668531717
ENSG00000121454	LHX4	1.498976001	0.471556354	1.668475188
ENSG00000141219	C17orf80	7.088115074	2.230117513	1.668282293
ENSG00000163032	VSNL1	3.801348312	1.196214902	1.668034628
ENSG00000188603	CLN3	5.525388488	1.738768279	1.668010222
ENSG00000136720	HS6ST1	17.60542187	5.541751887	1.667605773
ENSG00000196544	BORCS6	2.577424	0.811703	1.666906037
ENSG00000165506	DNAAF2	5.560620675	1.751291763	1.66682647
ENSG00000109956	B3GAT1	0.103163492	0.032496559	1.66657364
ENSG00000230564	CALM1P2	1.533702	0.48322	1.66626612
ENSG00000230069	LRRC37A15P	0.672983	0.212208	1.665091017
ENSG00000168517	HEXIM2	1.700811409	0.536483749	1.664616805
ENSG00000175206	NPPA	0.292901175	0.092442295	1.663789004
ENSG00000165059	PRKACG	0.043653	0.013791	1.662353733
ENSG00000257740	RP11-977G19.12	1.050514	0.331926	1.662161842
ENSG00000277203	F8A1	8.43338	2.665797	1.661544039
ENSG00000089775	ZBTB25	2.244087428	0.709369856	1.661518952
ENSG00000215035	FDPSP5	1.133728	0.358414	1.661375659
ENSG00000073670	ADAM11	3.984546908	1.259993222	1.660999709
ENSG00000159307	SCUBE1	1.984863845	0.628074368	1.660032749
ENSG00000259299	RP11-37J13.1	1.808756	0.572606	1.65938311
ENSG00000264058	RP5-1028K7.3	1.471338	0.46581	1.659315188
ENSG00000198108	CHSY3	1.073733861	0.340005447	1.659006683
ENSG00000234771	SLC25A25-AS1	1.7273	0.547051	1.658771431
ENSG00000083814	ZNF671	0.160594585	0.050872329	1.658470196
ENSG00000260213	RP11-303E16.3	0.484587241	0.153517278	1.658355385
ENSG00000177989	ODF3B	0.345204307	0.109364206	1.658309827
ENSG00000055130	CUL1	24.441045	7.749850713	1.657065546
ENSG00000174498	IGDCC3	0.334969017	0.106252981	1.65652434
ENSG00000279809	AC005538.3	6.884822	2.184217	1.656303162
ENSG00000175611	LINC00476	0.326134993	0.103490185	1.6559753
ENSG00000063176	SPHK2	1.226103202	0.389196847	1.655508488
ENSG00000203943	SAMD13	1.008287741	0.320171468	1.654990755
ENSG00000196639	HRH1	1.563915298	0.496698252	1.654720805
ENSG00000114745	GORASP1	4.133166238	1.312859293	1.654535088
ENSG00000183891	TTC32	1.00642595	0.319719667	1.654361629
ENSG00000171617	ENC1	18.14550806	5.767632692	1.653561256

ENSG00000197446	CYP2F1	0.152830257	0.048597943	1.652963053
ENSG00000257150	PGAM1P5	0.487653594	0.155098558	1.652671414
ENSG00000200418	SNORA63	0.844771	0.268894	1.651522746
ENSG00000258875	CTD-2547L24.3	0.177470883	0.056492787	1.65144376
ENSG00000158186	MRAS	2.809260869	0.894254799	1.65143274
ENSG00000228626	RP11-495P10.9	0.596122	0.189897	1.650390503
ENSG00000182902	SLC25A18	0.173697864	0.055334415	1.650331077
ENSG00000254662	RP11-872D17.4	2.042383	0.650737	1.650106944
ENSG00000259306	RP11-108K3.2	0.520768	0.165989	1.649553163
ENSG00000277805	MIR1199	0.106925	0.034084	1.649432646
ENSG00000231305	RP11-723O4.2	0.931563777	0.296994854	1.649216608
ENSG00000255046	RP11-297N6.4	0.184737772	0.058921625	1.648609745
ENSG00000272733	KB-208E9.1	0.470826	0.150195	1.648357205
ENSG00000248459	RP11-93K22.1	0.37608	0.119971	1.648353873
ENSG00000180105	AC017081.2	0.309212	0.09864	1.648351604
ENSG00000273108	RP11-416N2.4	0.35542	0.113381	1.648345968
ENSG00000111886	GABRR2	0.319528403	0.101931385	1.648345844
ENSG00000248696	AC011406.2	0.275114	0.087763	1.64834481
ENSG00000163762	TM4SF18	0.113556666	0.036225301	1.64834281
ENSG00000011590	ZBTB32	0.095544856	0.030479483	1.648339774
ENSG00000166707	ZCCHC18	0.075090525	0.023954961	1.648306392
ENSG00000090554	FLT3LG	0.338536569	0.107998144	1.648305169
ENSG00000204439	C6orf47	6.8787	2.194561	1.648203565
ENSG00000258034	RP11-493P1.2	0.338594	0.108025	1.648191177
ENSG00000230896	RP11-767N6.7	3.419078	1.092237	1.646321401
ENSG00000264895	RP11-421E14.2	0.234076	0.074821	1.645461868
ENSG00000260306	RP11-645C24.5	0.610143	0.195029	1.645458746
ENSG00000272058	FAM231D	0.488467	0.156164	1.645199188
ENSG00000155629	PIK3AP1	6.304781602	2.016920872	1.644291911
ENSG00000170608	FOXA3	1.453729198	0.465160135	1.643959183
ENSG00000224533	TMLHE-AS1	0.276454615	0.08845972	1.643950068
ENSG00000221916	C19orf73	0.812535	0.260103	1.643346919
ENSG00000277152	RP11-622C24.2	0.075424	0.024157	1.642582363
ENSG00000264177	RP1-37N7.1	0.029834	0.009557	1.642327697
ENSG00000136205	TNS3	2.840577548	0.910325773	1.641729458
ENSG00000258344	RP11-968A15.8	0.482465	0.15463	1.641604041
ENSG00000170145	SIK2	10.39773015	3.332665734	1.641520092
ENSG00000223723	BX842568.2	4.096525	1.313707	1.640757076
ENSG00000142552	RCN3	0.240255099	0.077056571	1.640577151
ENSG00000183479	TREX2	0.227036965	0.07285385	1.639850083
ENSG00000261754	CTC-523E23.1	0.386568	0.124063	1.6396493
ENSG00000240761	AC098831.4	0.456099304	0.146521913	1.638231527
ENSG00000148411	NACC2	13.41050049	4.309682639	1.637709541
ENSG00000235299	MRPL53P1	0.137212	0.04411	1.637228994
ENSG00000168077	SCARA3	9.341912424	3.00380706	1.636925772
ENSG00000092964	DPYSL2	18.01573569	5.793462646	1.636759784
ENSG00000274925	CTD-2547G23.4	0.699965	0.22517	1.636268159
ENSG00000175279	APITD1	4.164244788	1.339642564	1.636206758

ENSG00000278131	AC116050.1	0.070881	0.022816	1.635353071
ENSG00000262703	RP11-485G7.6	0.307005	0.09885	1.634949281
ENSG00000186831	KRT17P2	0.067387066	0.021699071	1.634838407
ENSG00000197343	ZNF655	6.034109226	1.943859403	1.634216937
ENSG00000269054	CTD-2619J13.3	0.42524	0.137014	1.633953995
ENSG00000184709	LRRC26	0.416768	0.134294	1.63384966
ENSG00000270326	RP5-874C20.6	0.522147	0.16825	1.633849521
ENSG00000246582	RP11-1149O23.3	2.064895775	0.665565799	1.633415758
ENSG00000108798	ABI3	0.114806541	0.037019991	1.63282837
ENSG00000224050	RP1-90G24.6	1.295395	0.41788	1.632231464
ENSG00000229168	RPL19P20	0.283762	0.091574	1.631671457
ENSG00000179403	VWA1	3.185691343	1.028344392	1.631282989
ENSG00000230082	PRRT3-AS1	1.894931	0.611721	1.631199608
ENSG00000262621	LA16c-306E5.2	0.745855092	0.240776902	1.631198367
ENSG00000269113	TRABD2B	0.071489	0.023082	1.630953036
ENSG00000196660	SLC30A10	0.087132367	0.028138722	1.63065193
ENSG00000221266	AC044860.1	0.023052	0.007445	1.630548171
ENSG00000188766	SPRED3	0.246112268	0.079493798	1.630402371
ENSG00000230735	RP11-413E1.4	0.184018	0.059446	1.63019525
ENSG00000252106	RNY3P15	8.607407	2.782275	1.629313665
ENSG00000172426	RSPH9	0.971398317	0.314130689	1.628698094
ENSG00000134318	ROCK2	30.84483003	9.977910286	1.628219093
ENSG00000101162	TUBB1	0.143546	0.046442	1.628011122
ENSG00000259065	RP5-1021I20.1	0.486177619	0.157326811	1.627718931
ENSG00000247595	SPTY2D1-AS1	0.308634769	0.099936042	1.626823612
ENSG00000257616	RP11-641A6.5	0.650468	0.210628	1.62678085
ENSG00000128394	APOBEC3F	11.65936371	3.776300154	1.626443713
ENSG00000080839	RBL1	6.657723928	2.156456575	1.626366383
ENSG00000221990	EXOC3-AS1	0.642706835	0.208280731	1.625631438
ENSG00000172824	CES4A	0.038465326	0.012469897	1.625109024
ENSG00000118307	CASC1	0.221649216	0.071867429	1.624868284
ENSG00000178718	RPP25	2.889516	0.937214	1.624377448
ENSG00000254671	STT3A-AS1	0.365408	0.118586	1.623574521
ENSG00000100154	TTC28	0.044275324	0.014380665	1.62237242
ENSG00000223776	LGALS8-AS1	0.114358778	0.037144667	1.622340111
ENSG00000268230	CTD-2619J13.8	0.140453936	0.045632484	1.621963968
ENSG00000143164	DCAF6	15.46131589	5.023538624	1.621887236
ENSG00000275437	RP5-908M14.10	0.44376	0.144224	1.62146837
ENSG00000256294	ZNF225	0.930021537	0.302384597	1.620879471
ENSG00000234402	ELK2BP	0.090648	0.029477	1.620685487
ENSG00000117899	MESDC2	9.108960263	2.964480191	1.619507231
ENSG00000231999	FLJ27354	0.238807522	0.077759809	1.618751701
ENSG00000206561	COLQ	1.266776539	0.412543002	1.618545639
ENSG00000241346	SNRPCP11	2.5055	0.81602	1.618422121
ENSG00000100056	DGCR14	3.26333962	1.063269451	1.617841891
ENSG00000100297	MCM5	12.86772839	4.19290377	1.617735765
ENSG00000149289	ZC3H12C	2.48526834	0.810332677	1.616815407
ENSG00000004776	HSPB6	0.088668697	0.028914778	1.616617839

ENSG00000170619	COMMD5	3.036335059	0.990157508	1.616601057
ENSG00000092054	MYH7	0.012542	0.00409	1.616594676
ENSG00000234638	AC053503.6	0.637047	0.207784	1.616315249
ENSG00000188315	C3orf62	2.199039405	0.717634027	1.615553254
ENSG00000280153	RP11-876N24.3	0.557754	0.182071	1.615127805
ENSG00000122592	HOXA7	4.107663691	1.341004105	1.615004414
ENSG00000106003	LFNG	3.846300006	1.256343736	1.614240055
ENSG00000267296	CEBPA-AS1	0.526097	0.171866	1.614044657
ENSG00000125637	PSD4	0.694771441	0.226969066	1.614042768
ENSG00000248015	AC005329.7	0.708119	0.231386	1.61369025
ENSG00000166225	FRS2	4.915953023	1.606932281	1.613161997
ENSG00000161682	FAM171A2	7.406003788	2.420959033	1.613116621
ENSG00000221838	AP4M1	3.38777307	1.107774414	1.612673116
ENSG00000138092	CENPO	7.758852204	2.537758314	1.612288566
ENSG00000151576	QTRTD1	4.163849756	1.362158943	1.612022959
ENSG00000154258	ABCA9	0.057918933	0.018950164	1.611824674
ENSG00000124507	PACSIN1	3.011421964	0.98536056	1.611721242
ENSG00000280401	RP11-104F15.9	0.675081	0.221117	1.610250668
ENSG00000276418	RP11-26J3.4	0.228159	0.074733	1.610222221
ENSG00000275294	RP11-428O18.6	0.601924517	0.197160182	1.610214362
ENSG00000271780	RP11-1017G21.5	0.612264	0.200632	1.609602129
ENSG00000157851	DPYSL5	3.888754547	1.274400009	1.609489994
ENSG00000226246	KRT18P36	0.272601	0.089372	1.60889604
ENSG00000196456	ZNF775	1.256028982	0.412071718	1.607902398
ENSG00000100014	SPECC1L	9.216020145	3.024842032	1.607284069
ENSG00000204390	HSPA1L	0.993916	0.32634	1.606748097
ENSG00000143867	OSR1	0.649483474	0.213260541	1.606675768
ENSG00000278228	MIR6732	1.153647	0.378844	1.606526043
ENSG00000180818	HOXC10	2.504416783	0.822592364	1.60622509
ENSG00000174151	CYB561D1	2.020204319	0.663904406	1.60545378
ENSG00000146834	MEPCE	9.641036313	3.168655123	1.605317585
ENSG00000174307	PHLDA3	9.513354862	3.127006251	1.60517209
ENSG00000224051	CPTP	5.121481002	1.683750141	1.604882995
ENSG00000160392	C19orf47	2.464445695	0.810284785	1.604762236
ENSG00000239224	RN7SL546P	0.170239	0.055987	1.604397799
ENSG00000260458	KCNJ18	0.521471	0.17153	1.604126103
ENSG00000166997	CNPY4	1.317170993	0.433286922	1.60404805
ENSG00000177602	GSG2	5.674874	1.868001	1.603093133
ENSG00000159176	CSRP1	6.118731299	2.014149685	1.603061642
ENSG00000115944	COX7A2L	12.42326438	4.090681574	1.602631166
ENSG00000135638	EMX1	0.441514729	0.145406987	1.602364972
ENSG00000246067	RAB30-AS1	1.353024746	0.445619564	1.602303747
ENSG00000138061	CYP1B1	0.573788161	0.188980795	1.602278573
ENSG00000119681	LTBP2	0.99257049	0.32693127	1.60218219
ENSG00000220981	AL391384.1	0.881374	0.290403	1.601697981
ENSG00000226746	SMCR5	0.035792	0.011798	1.601094847
ENSG00000241313	WWTR1-AS1	0.117317509	0.038672224	1.601048686
ENSG00000276581	SPATA31A5	0.007714	0.002544	1.600380475

ENSG00000276040	SPATA31A7	0.006580634	0.002170227	1.600380475
ENSG00000153531	ADPRHL1	1.067628474	0.352113878	1.600295692
ENSG00000253958	CLDN23	1.583438	0.522492	1.599579526
ENSG00000138231	DBR1	9.387269497	3.097724196	1.599496878
ENSG00000006042	TMEM98	6.266563957	2.068873089	1.598829461
ENSG00000272994	RP11-332H14.2	1.607391	0.530692	1.598774203
ENSG00000181027	FKRP	0.385782396	0.127385679	1.598584215
ENSG00000099783	HNRNPM	50.91689413	16.81420714	1.598463668
ENSG00000271002	RP11-599B13.8	0.520272	0.171811	1.59844366
ENSG00000012048	BRCA1	4.499062466	1.486220733	1.597975998
ENSG00000085276	MECOM	1.378346352	0.455424869	1.597653477
ENSG00000163576	EFHB	0.195613937	0.064655355	1.597167398
ENSG00000184185	KCNJ12	2.823559418	0.93325936	1.597165013
ENSG00000281881	SPRY4-IT1	3.558632	1.176333	1.59702623
ENSG00000244607	CCDC13	0.406551171	0.134401491	1.596887806
ENSG00000169129	AFAP1L2	1.557481836	0.515021374	1.596511127
ENSG00000183778	B3GALT5	0.365704567	0.120948041	1.596291242
ENSG00000260011	RP13-20L14.1	0.390908	0.129287	1.596251893
ENSG00000260293	RP11-715J22.6	0.116546	0.038548	1.596171575
ENSG00000225101	OR52K3P	0.161918	0.053565	1.595900835
ENSG00000207475	SNORA80E	0.334354	0.110612	1.595868469
ENSG00000167695	FAM57A	6.985741266	2.311091791	1.595838649
ENSG00000215695	RSC1A1	7.609484	2.517451	1.59583493
ENSG00000273485	RP11-225H22.7	0.254293	0.084184	1.594873783
ENSG00000238227	C9orf69	7.483393393	2.478134307	1.594438239
ENSG00000140986	RPL3L	0.235842349	0.078100252	1.594423696
ENSG00000229539	RP11-119B16.2	2.020662	0.669184	1.594353163
ENSG00000108179	PPIF	27.99977846	9.273487203	1.594231555
ENSG00000231769	RP1-8B1.4	0.968336371	0.320768663	1.593975076
ENSG00000280489	U7	1.293231	0.428434	1.593835116
ENSG00000188613	NANOS1	0.283812393	0.094040707	1.593580299
ENSG00000244641	RPS26P43	0.388592	0.128772	1.59343727
ENSG00000198624	CCDC69	4.620623015	1.532630807	1.592077178
ENSG00000087116	ADAMTS2	0.999778167	0.331629856	1.592034126
ENSG00000276845	RP11-374M1.9	0.248284	0.082376	1.591695315
ENSG00000198182	ZNF607	1.268826131	0.420978174	1.591677045
ENSG00000101412	E2F1	11.582903	3.847467	1.590016021
ENSG00000166170	BAG5	4.115199686	1.367233946	1.589702315
ENSG00000156239	N6AMT1	1.818035699	0.604072108	1.589587849
ENSG00000273424	CTA-223H9.9	0.368479	0.122444	1.58946032
ENSG00000197162	ZNF785	0.03288885	0.010929617	1.589355734
ENSG00000115993	TRAK2	7.886997758	2.621429136	1.589122686
ENSG00000057019	DCBLD2	65.31095854	21.71064905	1.588922225
ENSG00000169548	ZNF280A	1.818568	0.604706	1.588497073
ENSG00000207928	MIR632	0.069257	0.02303	1.588445485
ENSG00000185585	OLFML2A	9.172544759	3.050533331	1.588260545
ENSG00000276600	RAB7B	0.44141467	0.14682708	1.5880165
ENSG00000174514	MFSD4	0.687319535	0.228633063	1.587946914

ENSG00000225920	RIMKLBP2	1.989154	0.662025	1.58719737
ENSG00000150455	TIRAP	2.532080608	0.842723621	1.587191865
ENSG00000111875	ASF1A	18.99029434	6.321234991	1.586983914
ENSG00000169314	C22orf15	0.233758781	0.077834659	1.586535944
ENSG00000151835	SACS	10.24659086	3.412375201	1.586295801
ENSG00000185972	CCIN	0.234422	0.078072	1.586230837
ENSG00000207556	MIR636	0.047971	0.01598	1.585895106
ENSG00000157978	LDLRAP1	6.693354809	2.229688781	1.585887139
ENSG00000106689	LHX2	4.839942549	1.612487525	1.585701924
ENSG00000264391	RN7SL208P	0.51584	0.17187	1.585605905
ENSG00000279742	RP11-700A24.1	0.063966	0.021313	1.58557159
ENSG00000225117	ARSDP1	0.062834	0.020936	1.585559595
ENSG00000049283	EPN3	2.051213758	0.683652684	1.58514236
ENSG00000263731	RP11-498C9.15	2.275717	0.758736	1.584651264
ENSG00000196372	ASB13	5.347453723	1.783320548	1.584286045
ENSG00000135766	EGLN1	9.549617697	3.185569286	1.583891762
ENSG00000186654	PRR5	2.488431154	0.83017404	1.583750749
ENSG00000261211	RP1-80N2.3	0.287774	0.096034	1.583319077
ENSG00000121075	TBX4	0.162266405	0.05417497	1.582665973
ENSG00000101638	ST8SIA5	0.051318969	0.017139121	1.582199096
ENSG00000186193	SAPCD2	23.359108	7.801814	1.582103675
ENSG00000227775	RP1-283E3.4	1.59992667	0.534525997	1.581673762
ENSG00000223756	TSSC2	0.589216328	0.196935421	1.581074787
ENSG00000244491	RP3-508I15.18	1.116904	0.373372	1.580819543
ENSG00000204685	STARD7-AS1	0.665709249	0.222564804	1.580666747
ENSG00000173614	NMNAT1	2.996383519	1.001875043	1.58051971
ENSG00000198924	DCLRE1A	7.0062104	2.342713858	1.580453562
ENSG00000235795	RP11-421L21.2	0.711485	0.237929	1.580302216
ENSG00000108423	TUBD1	3.836034474	1.283702397	1.579304907
ENSG00000134287	ARF3	42.58734569	14.26278906	1.578168689
ENSG00000234898	CHEK2P3	0.444736	0.149019	1.577452904
ENSG00000261655	CTD-3064M3.3	2.705822	0.907614	1.575916168
ENSG00000159216	RUNX1	6.630415787	2.224345837	1.575718231
ENSG00000233653	CICP7	0.104338	0.035015	1.575219692
ENSG00000245614	DDX11-AS1	0.65969716	0.22143112	1.5749459
ENSG00000159720	ATP6V0D1	4.989781491	1.675143723	1.574691759
ENSG00000276965	TUG1_3	2.014457	0.676381	1.574482971
ENSG00000171004	HS6ST2	5.297797222	1.778932023	1.574381241
ENSG00000239272	RPL21P10	0.375852	0.126229	1.574121286
ENSG00000251087	ALG1L3P	0.194489	0.065322	1.574047692
ENSG00000261916	RP11-235E17.4	0.912553	0.306542	1.573823597
ENSG00000229670	PKP4P1	0.892688	0.299945	1.57345805
ENSG00000144230	GPR17	0.180886965	0.060786975	1.573254321
ENSG00000271646	RP11-326I11.3	0.321685	0.108111	1.573135345
ENSG00000277287	RP4-794I6.4	0.505377	0.169853	1.573073308
ENSG00000187908	DMBT1	0.218976629	0.073609504	1.57281294
ENSG00000154133	ROBO4	0.047144254	0.015851799	1.572435385
ENSG00000084090	STARD7	47.3150516	15.91229026	1.572157701

ENSG00000277916	uc_338	1.233031	0.414748	1.571902142
ENSG00000235586	AC011247.3	0.690132	0.232277	1.571026023
ENSG00000254294	IMPDH1P6	0.37158	0.125114	1.570429619
ENSG00000120925	RNF170	1.740971665	0.586289394	1.570207859
ENSG00000271851	RP11-565F19.2	0.744362	0.250769	1.569645394
ENSG00000188493	C19orf54	3.166913562	1.066995112	1.569523924
ENSG00000115318	LOXL3	0.983659832	0.331414667	1.569522038
ENSG00000248996	RP11-1334A24.6	0.739655	0.249404	1.568367902
ENSG00000119242	CCDC92	1.586405572	0.535095931	1.567892188
ENSG00000180185	FAHD1	6.184919417	2.087175227	1.567203072
ENSG00000022567	SLC45A4	4.467296594	1.508282222	1.566495638
ENSG00000247092	SNHG10	0.634423544	0.214201127	1.566480241
ENSG00000164889	SLC4A2	21.72094374	7.334301678	1.566355273
ENSG00000171385	KCND3	1.585983454	0.535630247	1.566068383
ENSG00000144730	IL17RD	17.51988229	5.918447672	1.56570235
ENSG00000234705	HMGA1P4	0.479561	0.162038	1.565382154
ENSG00000281147	SNORD50A	0.275686	0.093166	1.565150547
ENSG00000146147	MLIP	0.062343902	0.021074364	1.564759347
ENSG00000237027	RP11-16C18.3	0.136625761	0.046217304	1.56372452
ENSG00000278505	C17orf78	0.011669762	0.003948139	1.563530638
ENSG00000260686	CTB-36H16.2	0.514103	0.173941	1.563459397
ENSG00000103742	IGDCC4	0.436327726	0.147639678	1.563331651
ENSG00000270049	RP11-297D21.4	0.288708569	0.09769036	1.563325822
ENSG00000088256	GNA11	14.37823575	4.867916196	1.562510427
ENSG00000205413	SAMD9	5.936331905	2.009920916	1.562433019
ENSG00000236701	RP11-549A6.1	0.157919	0.053479	1.562140365
ENSG00000163807	KIAA1143	11.44443544	3.87628622	1.561899289
ENSG00000250508	RP11-757G1.6	0.289852	0.098189	1.561683125
ENSG00000277150	F8A3	6.213453	2.106262	1.560710334
ENSG00000010379	SLC6A13	0.037620178	0.012755285	1.560411518
ENSG00000256746	RP11-17G12.3	0.36652166	0.124272224	1.560394585
ENSG00000213801	ZNF321P	0.26785188	0.09084706	1.559923691
ENSG00000175267	VWA3A	0.08524743	0.028927327	1.559223331
ENSG00000159164	SV2A	0.029752159	0.010096711	1.559108957
ENSG00000168792	ABHD15	2.970456	1.00813	1.55900273
ENSG00000235955	CICP20	0.036038	0.012237	1.558269035
ENSG00000168286	THAP11	7.953354	2.701077	1.558028618
ENSG00000166359	WDR88	0.707158764	0.240185652	1.557884181
ENSG00000276150	TTC28-AS1_2	0.964061	0.32746	1.557805746
ENSG00000259439	RP11-89K21.1	0.82298253	0.279548497	1.557763219
ENSG00000214321	CBX1P4	1.742202	0.592128	1.556930926
ENSG00000105137	SYDE1	1.542429795	0.524548326	1.556057229
ENSG00000230333	AC004538.3	0.008992306	0.003059791	1.555257997
ENSG00000234607	RP11-5P18.1	0.090402	0.030761	1.555252286
ENSG00000266891	RP11-692N5.2	0.069153	0.023531	1.555229208
ENSG00000278831	RP11-12J10.4	0.655931	0.223331	1.554360538
ENSG00000274349	ZNF658	1.784325798	0.607696209	1.553956862
ENSG00000249799	SNRPCP13	2.606421	0.887766	1.553818771

ENSG00000159714	ZDHHC1	2.770908507	0.943792323	1.553817734
ENSG00000272220	RP11-927P21.9	1.457874	0.496716	1.553372913
ENSG00000197256	KANK2	9.21184132	3.139434871	1.552984677
ENSG00000111206	FOXM1	18.6680272	6.363674867	1.552637442
ENSG00000213024	NUP62	13.5882004	4.632202173	1.552584274
ENSG00000280388	RP11-229D13.3	0.269978	0.092062	1.55216416
ENSG00000226537	OR7E33P	0.067177	0.022921	1.551297381
ENSG00000280832	ST3GAL4-AS1	0.840961723	0.287141876	1.550276392
ENSG00000196542	SPTSSB	4.140815822	1.414122592	1.55000784
ENSG00000160472	TMEM190	0.165687	0.056585	1.549968844
ENSG00000128191	DGCR8	5.754037715	1.965340337	1.549795512
ENSG00000223972	DDX11L1	0.2768445	0.094619953	1.548859514
ENSG00000176055	MBLAC2	1.722903315	0.589070915	1.548328516
ENSG00000198022	SAGE2P	0.150342	0.051422	1.547790473
ENSG00000278198	AC011043.2	0.169999	0.058214	1.546088203
ENSG00000104059	FAM189A1	0.601024931	0.205818507	1.546052123
ENSG00000090534	THPO	0.316805909	0.108523796	1.545587821
ENSG00000274888	MIR6890	0.638661	0.218848	1.545121155
ENSG00000207383	Y_RNA	2.126554	0.728751	1.545019627
ENSG00000203644	RP11-332M2.1	6.906224	2.36703	1.544819137
ENSG00000082497	SERTAD4	0.378904937	0.129881578	1.544639115
ENSG00000227201	CNN2P1	0.171871	0.058955	1.543640059
ENSG00000080166	DCT	0.020917347	0.007175399	1.543569023
ENSG00000198841	KTI12	3.489093	1.197345	1.543013146
ENSG00000090674	MCOLN1	4.126094305	1.416086324	1.542867581
ENSG00000239985	RP11-460N20.3	0.644597	0.221317	1.542283201
ENSG00000227051	C14orf132	1.935155103	0.664423905	1.542273319
ENSG00000266154	MIR4658	0.928233	0.318776	1.541943972
ENSG00000076706	MCAM	11.17777304	3.841309954	1.540962503
ENSG00000145700	ANKRD31	0.100378658	0.034510049	1.540364126
ENSG00000183476	SH2D7	0.319086862	0.109706959	1.540294162
ENSG00000213888	LINC01521	0.157167976	0.05403679	1.540293406
ENSG00000253295	RP11-779O18.1	0.443159	0.152392	1.540037244
ENSG00000170681	MURC	0.932675	0.320791	1.53974078
ENSG00000262001	DLGAP1-AS2	6.263912002	2.155443096	1.53907947
ENSG00000160336	ZNF761	3.632916163	1.25013451	1.539044741
ENSG00000073111	MCM2	29.28452345	10.07768817	1.538973697
ENSG00000228570	NUTM2E	0.577047453	0.198628351	1.538618401
ENSG00000154319	FAM167A	1.659392639	0.571207049	1.538569603
ENSG00000277702	CH17-13I23.3	1.258069	0.433219	1.538042628
ENSG00000232748	RP11-196G11.6	1.953428513	0.672850638	1.537650269
ENSG00000263531	RP13-753N3.1	0.367482	0.126606	1.537327808
ENSG00000279673	RP11-185E8.2	1.785234	0.61523	1.536915429
ENSG00000272447	RP11-182L21.6	1.116922	0.384984	1.536658046
ENSG00000155026	RSPH10B	0.067292614	0.023196077	1.53656733
ENSG00000184887	BTBD6	7.347262319	2.533175227	1.536259908
ENSG00000228882	CICP9	0.152485	0.052574	1.536245922
ENSG00000165985	C1QL3	1.741302	0.600386	1.536204195

ENSG00000257366	MIR3180-2		0.10756	0.037094	1.535883907
ENSG00000257563	MIR3180-3		0.10756	0.037094	1.535883907
ENSG00000224625	TUBB8P6		0.146209	0.050423	1.535878259
ENSG00000169851	PCDH7		18.87196606	6.508960532	1.535745658
ENSG00000261546	CTD-2555A7.3		0.065592	0.022626	1.535538309
ENSG00000165409	TSHR		0.082997965	0.028633587	1.535367562
ENSG00000135905	DOCK10		0.014167491	0.004891155	1.534337325
ENSG00000276791	CTD-2270P14.5		0.313521	0.108241	1.534315007
ENSG00000184923	NUTM2A		0.583593055	0.201491683	1.534242423
ENSG00000171135	JAGN1		9.851881815	3.404268745	1.533054388
ENSG00000132581	SDF2		6.527262685	2.256228122	1.532565158
ENSG00000259493	RP11-621H8.2		1.704788	0.589703	1.531531904
ENSG00000272831	RP11-792A8.4		3.262109	1.128413	1.531509797
ENSG00000240024	LINC00888		3.761308574	1.301175265	1.531419366
ENSG00000165695	AK8		0.343656012	0.118902968	1.531180473
ENSG00000249244	RP11-548H18.2		0.296616	0.102631	1.531129854
ENSG00000143502	SUSD4		1.115442363	0.386030227	1.530830245
ENSG00000267466	RP11-13K12.5		0.028804	0.009969	1.530748473
ENSG00000178202	KDELC2		8.375164595	2.899873036	1.530127807
ENSG00000239713	APOBEC3G		2.519622579	0.87244895	1.530065024
ENSG00000200463	SNORD118		0.025708	0.008903	1.529853917
ENSG00000173838		10-Mar	0.06351742	0.02200027	1.529631114
ENSG00000182165	TP53TG1		0.065672598	0.022748718	1.52950626
ENSG00000273588	RP11-433A19.2		0.434083	0.150399	1.529175949
ENSG00000279094	CH507-24F1.1		0.353619202	0.122577534	1.528502027
ENSG00000263842	AC104986.1		1.95821	0.6788	1.528477019
ENSG00000005889	ZFX		3.389157	1.174859581	1.528438134
ENSG00000232579	MTCO1P27		0.207188	0.071842	1.528041028
ENSG00000129933	MAU2		2.555867639	0.886562518	1.527518851
ENSG00000167080	B4GALNT2		0.113139967	0.039257432	1.527070927
ENSG00000281199	AL139042.1		0.575612	0.199731	1.5270384
ENSG00000065320	NTN1		6.922217597	2.403077069	1.526351375
ENSG00000090612	ZNF268		0.138417992	0.048053422	1.526320406
ENSG00000186564	FOXD2		1.135176	0.394298	1.525557695
ENSG00000256271	CACNA1C-AS2		0.25072	0.087091	1.525481542
ENSG00000256235	SMIM3		22.516117	7.825199	1.524758704
ENSG00000146267	FAXC		0.759706324	0.264058372	1.524584947
ENSG00000104833	TUBB4A		22.89051338	7.95755297	1.524353059
ENSG00000130717	UCK1		5.607474895	1.951339626	1.522886359
ENSG00000189114	BLOC1S3		0.557474686	0.194008006	1.522790109
ENSG00000266921	RP11-15A1.7		0.053423771	0.018604744	1.521811285
ENSG00000266456	RP11-806L2.2		0.258268	0.089944	1.521769951
ENSG00000213155	RP11-252O18.3		1.576051	0.548937	1.521601731
ENSG00000237276	ANO7P1		0.555894345	0.193637869	1.521449581
ENSG00000273604	C17orf96		14.594443	5.085206	1.521041028
ENSG00000173894	CBX2		11.15433031	3.88673348	1.520973811
ENSG00000262251	RP11-199F11.2		1.385478	0.482802	1.520880245
ENSG00000055955	ITIH4		0.454558351	0.158425318	1.520662591

ENSG00000151575	TEX9	0.874426245	0.304762431	1.520651634
ENSG00000136295	TTYH3	18.68472301	6.512434362	1.520590345
ENSG00000281358	RASSF1-AS1	0.18574	0.064739	1.520577554
ENSG00000275496	CH507-24F1.2	1.337577798	0.466213883	1.520558937
ENSG00000223749	MIR503HG	0.155773467	0.054302645	1.520355144
ENSG00000034053	APBA2	0.104546337	0.036497304	1.518280695
ENSG00000169715	MT1E	33.69903285	11.76859537	1.517765048
ENSG00000147274	RBMX	28.96302246	10.1159923	1.51757432
ENSG00000119414	PPP6C	20.5076711	7.163517029	1.517423688
ENSG00000272808	RP11-66B24.7	0.580636	0.202899	1.516872269
ENSG00000237206	IMPDH1P4	0.201893	0.070561	1.516647978
ENSG00000182742	HOXB4	2.948054	1.030551	1.516347048
ENSG00000122482	ZNF644	7.53960022	2.635708743	1.516297072
ENSG00000227799	AC012358.4	0.971526	0.339669	1.51612305
ENSG00000233695	GAS6-AS1	0.776292082	0.271495178	1.515672996
ENSG00000144120	TMEM177	5.34708162	1.870231675	1.515534704
ENSG00000175606	TMEM70	8.424751439	2.949047606	1.51438501
ENSG00000273253	RP3-402G11.26	0.646911	0.226545	1.513769594
ENSG00000142449	FBN3	0.991213241	0.347148831	1.513641144
ENSG00000163946	FAM208A	5.28595216	1.852115378	1.512989394
ENSG00000267815	CTB-191K22.5	1.184523	0.415443	1.511583758
ENSG00000181577	C6orf223	4.102495993	1.439369372	1.511065059
ENSG00000242170	RN7SL329P	0.340443	0.119464	1.510837336
ENSG00000177994	C2orf73	0.037478597	0.013151544	1.510834738
ENSG00000179922	ZNF784	1.348389988	0.473356892	1.510237589
ENSG00000207425	Y_RNA	4.077295	1.431488	1.510096766
ENSG00000206927	Y_RNA	4.077295	1.431488	1.510096766
ENSG00000259212	CTD-3065B20.2	0.893154	0.313846	1.508852128
ENSG00000156509	FBXO43	0.090196486	0.031695764	1.508781175
ENSG00000204054	LINC00963	0.957447804	0.336547722	1.508382748
ENSG00000277105	FP236383.10	0.432171	0.151917	1.508318945
ENSG00000276736	AL592188.6	0.432171	0.151917	1.508318945
ENSG00000253943	KRT18P37	0.167114	0.058778	1.507484425
ENSG00000217379	RP11-254A17.1	0.029708	0.010453	1.50693443
ENSG00000198324	FAM109A	3.553916419	1.250909787	1.506432004
ENSG00000086061	DNAJA1	73.60519708	25.90859723	1.506376729
ENSG00000275106	RP11-309L24.10	0.93417	0.328833	1.506330027
ENSG00000259764	PCSK6-AS1	0.481084	0.169392	1.505923078
ENSG00000006194	ZNF263	2.17291263	0.76534578	1.505446563
ENSG00000279868	RN7SL3	67.834984	23.895826	1.505270857
ENSG00000198074	AKR1B10	0.529721628	0.186608928	1.505216406
ENSG00000278677	HIST1H2AM	0.781359	0.275423	1.504336514
ENSG00000135083	CCNJL	2.890053926	1.018856965	1.504144884
ENSG00000261136	RP11-37C7.3	0.751667	0.265008	1.504057755
ENSG00000258653	RP5-1021I20.4	0.427044	0.150608	1.503586319
ENSG00000260219	RP11-347C12.10	1.470448	0.518649	1.503425348
ENSG00000235207	TUBBP6	0.560164	0.19767	1.502755336
ENSG00000198718	FAM179B	0.253748892	0.089551029	1.502619606

ENSG00000277991	CH507-338C24.1	0.731825442	0.25828496	1.502535936
ENSG00000125931	CITED1	1.129548931	0.399138948	1.500783798
ENSG00000187955	COL14A1	0.104299347	0.0368645	1.500426025
ENSG00000144649	FAM198A	0.242943951	0.08588658	1.500118876
ENSG00000127366	TAS2R5	1.183303	0.418482	1.499582067
ENSG00000251013	GAPDHP62	2.294908	0.811808	1.499225857
ENSG00000238123	MID1IP1-AS1	2.912549	1.030358	1.49913663
ENSG00000134982	APC	4.107502837	1.453119601	1.499108121
ENSG00000245888	FLJ21408	0.230343388	0.081528324	1.498412922
ENSG00000267040	RP11-35G9.3	2.911309673	1.031278824	1.497233864
ENSG00000272533	SNORA28	0.65438	0.231835	1.49703027
ENSG00000186889	TMEM17	1.110914186	0.393693022	1.496604331
ENSG00000163638	ADAMTS9	0.014297142	0.005066962	1.496533881
ENSG00000081277	PKP1	0.024583852	0.00871292	1.496482807
ENSG00000116183	PAPPA2	0.013969049	0.00495101	1.496438908
ENSG00000164188	RANBP3L	0.045402576	0.016092251	1.49640802
ENSG00000121351	IAPP	0.03523042	0.012487049	1.496389109
ENSG00000224189	HAGLR	0.031475714	0.01115627	1.496384383
ENSG00000154864	PIEZO2	0.010319628	0.003657714	1.496376637
ENSG00000222004	C7orf71	0.075253816	0.026673194	1.496374159
ENSG00000248318	RP11-713M15.1	0.077389	0.02743	1.496373895
ENSG00000261056	RP11-454F8.2	0.097316	0.034493	1.496373411
ENSG00000226057	PHF2P2	0.079942	0.028335	1.496368464
ENSG00000226673	LINC01108	0.041300834	0.014638853	1.496368391
ENSG00000279891	FLJ42393	0.067263	0.023841	1.496368376
ENSG00000230528	NOS2P3	0.020956442	0.007427935	1.496360692
ENSG00000226148	SLC25A39P1	0.14337	0.050817	1.49636006
ENSG00000248531	NDUFA5P12	0.148386	0.052595	1.496357422
ENSG00000154556	SORBS2	0.009126607	0.003234901	1.496357034
ENSG00000233895	RP1-122P22.2	0.140209871	0.049697022	1.496356606
ENSG00000174837	ADGRE1	0.041189693	0.014599583	1.496356163
ENSG00000176788	BASP1	0.068871859	0.024411459	1.496356117
ENSG00000229336	AP000568.2	0.34545	0.122444	1.496354831
ENSG00000261186	RP11-341N2.1	0.13793	0.048889	1.496354478
ENSG00000229156	RP11-250H24.2	0.269292	0.09545	1.496354269
ENSG00000274340	RP11-435J9.2	0.098573	0.034939	1.496354217
ENSG00000264840	RN7SL404P	0.108676	0.03852	1.496353763
ENSG00000250072	CTC-529P8.1	0.070804182	0.025096417	1.496353157
ENSG00000238173	RPL39P6	0.101792	0.03608	1.496352938
ENSG00000276188	RP11-173P15.10	0.370604	0.13136	1.496352421
ENSG00000235726	AC010148.1	0.051358449	0.018203923	1.496352222
ENSG00000231378	NDUFAF4P4	0.456241	0.161714	1.496351518
ENSG00000270557	RP11-546J1.1	0.345841	0.122583	1.496349995
ENSG00000272970	RP11-329B9.4	0.435631	0.154409	1.496349776
ENSG00000233936	RP11-399H11.2	0.172335	0.061084	1.496349289
ENSG00000269794	AC010642.2	0.125877	0.044617	1.496349284
ENSG00000244671	RN7SL280P	0.530169	0.187918	1.496349051
ENSG00000265802	RN7SL49P	0.512378	0.181612	1.496349

ENSG00000134339	SAA2	0.137125176	0.048603939	1.496348331
ENSG00000225364	ATP6V0E1P1	0.675614	0.239471	1.496348268
ENSG00000234380	LINC01426	0.094867288	0.03362566	1.496348114
ENSG00000123843	C4BPB	0.047433767	0.016812876	1.496347919
ENSG00000272088	RP11-168F9.2	0.799417	0.283353	1.496347776
ENSG00000229373	LINC00452	0.085348633	0.030251788	1.496347648
ENSG00000272156	RP11-477N3.1	0.208591	0.073935	1.496347525
ENSG00000151376	ME3	0.039783627	0.014101299	1.496346743
ENSG00000270207	RP11-157E16.1	0.302954	0.107382	1.496346573
ENSG00000257252	RP11-486A14.2	0.176518488	0.062566957	1.496346444
ENSG00000134594	RAB33A	0.121374	0.043021	1.496346444
ENSG00000230782	RP11-508N12.2	0.058681318	0.020799588	1.496346318
ENSG00000250961	CTD-2023N9.1	0.340443	0.12067	1.49634622
ENSG00000259999	RP11-252K23.1	0.357585	0.126746	1.496346002
ENSG00000234175	RP11-730A19.9	0.451742	0.16012	1.496345531
ENSG00000197238	HIST1H4J	0.601749	0.21329	1.496345512
ENSG00000233595	MTND2P29	0.075974	0.026929	1.496345124
ENSG00000230502	CTD-2230M5.1	0.908861	0.322146	1.496344986
ENSG00000224460	RP11-439L18.2	0.379822	0.134628	1.496344978
ENSG00000228844	RP11-467I20.3	0.112106	0.039736	1.496344937
ENSG00000277825	CTD-2013N17.7	0.211773	0.075063	1.496344809
ENSG00000270083	RP1-257I20.14	1.316413	0.466603	1.496344691
ENSG00000242791	RP11-651P23.5	0.211773315	0.075063135	1.496344368
ENSG00000243797	CTB-111H14.1	0.073337223	0.025994409	1.496344268
ENSG00000278957	RP11-89K21.2	0.316126	0.112051	1.496344171
ENSG00000231802	AC009502.1	0.398665	0.141307	1.496344017
ENSG00000259650	RP11-272D12.1	0.148161283	0.052515838	1.496344
ENSG00000244468	RP11-206M11.7	0.554224	0.196445	1.496343738
ENSG00000273450	RP11-76P2.4	0.725362	0.257105	1.496343501
ENSG00000272248	RP3-406P24.4	0.311609	0.11045	1.496343484
ENSG00000270880	RP11-255E6.5	0.246669	0.087432	1.496343111
ENSG00000240244	GAPDHP33	0.150729	0.053426	1.496343104
ENSG00000269404	SPIB	0.089165985	0.03160495	1.496342931
ENSG00000188916	FAM196A	0.034647524	0.012280843	1.496342626
ENSG00000246526	RP11-539L10.2	0.083848	0.02972	1.496342256
ENSG00000264630	PRKCA-AS1	0.018459015	0.006542818	1.496341442
ENSG00000234405	LL0XNC01-250H12.3	0.241596	0.085634	1.496340948
ENSG00000197540	GZMM	0.161575177	0.057270554	1.496340093
ENSG00000242154	RP4-778K6.3	0.279649	0.099122	1.496339969
ENSG00000237470	DCLRE1CP1	0.162434	0.057575	1.496339232
ENSG00000270177	CTD-2410N18.3	0.107679	0.038167	1.49633922
ENSG00000235374	SSR4P1	0.112373085	0.039830835	1.49633892
ENSG00000112530	PACRG	0.116913158	0.041440078	1.49633869
ENSG00000253596	CTD-2320G14.2	0.258794	0.09173	1.496338625
ENSG00000121594	CD80	0.047700695	0.016907599	1.496338472
ENSG00000259005	RP3-449M8.6	0.259234	0.091886	1.496337978
ENSG00000163331	DAPL1	0.101454308	0.035960696	1.496337295
ENSG00000248964	RP11-94H18.1	0.073620053	0.026094791	1.496336956

ENSG00000259656	RP11-325E5.1	0.205503	0.072841	1.496336821
ENSG00000273669	RP11-405M12.4	0.230299	0.08163	1.496336784
ENSG00000234772	LINC00412	0.363544	0.128859	1.496336681
ENSG00000234123	RHBDF1P1	0.15733	0.055766	1.496336095
ENSG00000279091	RP11-461F11.2	0.019729	0.006993	1.496334422
ENSG00000228069	MTCO3P29	0.196006	0.069475	1.496331983
ENSG00000251332	RP11-177C12.4	0.215055	0.076227	1.496331671
ENSG00000223928	RP11-183E9.2	0.080617	0.028575	1.496330599
ENSG00000134193	REG4	0.087425757	0.030988406	1.496329846
ENSG00000124134	KCNS1	0.033646	0.011926	1.496324759
ENSG00000232743	XXyac-YM21GA2.1	0.067591	0.023958	1.496323681
ENSG00000279286	RP11-248B24.1	0.053991369	0.019137539	1.496323493
ENSG00000233765	RP11-154P18.2	0.080151	0.02841	1.496321689
ENSG00000185527	PDE6G	0.096455003	0.034189075	1.496320702
ENSG00000235278	ZNF652P1	0.058323	0.020673	1.496317167
ENSG00000275552	CH17-302M23.1	0.057166	0.020263	1.496309561
ENSG00000237090	RP11-342M1.6	0.057575	0.020408	1.496307702
ENSG00000187905	LRRC74B	0.040775234	0.014453199	1.496304272
ENSG00000104903	LYL1	0.048954072	0.017352291	1.496302727
ENSG00000249628	LINC00942	0.063172	0.022392	1.496301856
ENSG00000122176	FMOD	0.046129	0.016351	1.496295146
ENSG00000274390	MIR6885	0.04627	0.016401	1.496293318
ENSG00000169418	NPR1	0.030519859	0.010818183	1.496290038
ENSG00000275167	MIR6815	0.025031	0.008873	1.496222055
ENSG00000197937	ZNF347	0.005574005	0.00197627	1.495934353
ENSG00000111664	GNB3	1.080534172	0.383367628	1.494944274
ENSG00000253228	NRBF2P4	1.914096	0.67916	1.494839792
ENSG00000129159	KCNC1	0.061952691	0.021996031	1.493923739
ENSG00000256897	RP11-17G12.2	0.481127	0.170895	1.493307576
ENSG00000139437	TCHP	2.229045777	0.791822198	1.493177828
ENSG00000078399	HOXA9	27.1082787	9.637965178	1.491933015
ENSG00000239494	RN7SL333P	1.024779	0.364448	1.491527929
ENSG00000227477	STK4-AS1	0.147389	0.052417	1.491522166
ENSG00000145949	MYLK4	0.052421	0.018643	1.491510841
ENSG00000256603	RP11-667M19.2	0.246664	0.087738	1.491273448
ENSG00000196584	XRCC2	4.651702489	1.654953886	1.49096781
ENSG00000143368	SF3B4	62.45582312	22.22294225	1.490786251
ENSG00000178093	TSSK6	0.449083103	0.159827273	1.490468835
ENSG00000118495	PLAGL1	3.19687484	1.137817256	1.490393397
ENSG00000198131	ZNF544	3.698621184	1.31661383	1.490155288
ENSG00000280602	AL627171.1	73.346741	26.110374	1.490109742
ENSG00000142303	ADAMTS10	1.144942181	0.407633975	1.489928538
ENSG00000186790	FOXE3	0.230202	0.081959	1.489926081
ENSG00000144843	ADPRH	0.494073859	0.175906066	1.489921489
ENSG00000261888	AC144831.1	0.171698	0.06113	1.489920762
ENSG00000229525	AC053503.4	0.38047	0.13547	1.489809302
ENSG00000279342	AP000866.1	1.464037	0.521549	1.489077308
ENSG00000257846	RP11-597A11.3	0.935025	0.333122	1.488954304

ENSG00000233922	AL133493.2	0.441249934	0.157355289	1.487570391
ENSG00000255561	FDXACB1	1.734834185	0.618665397	1.487566529
ENSG00000261613	RP11-20I23.13	1.531012	0.546038	1.487412331
ENSG00000159173	TNNI1	0.407384516	0.145299886	1.487357573
ENSG00000140682	TGFB1I1	1.322200691	0.471699583	1.487000944
ENSG00000259956	RBM15B	9.487462	3.384841	1.486934136
ENSG00000100628	ASB2	0.060186681	0.021476272	1.486700654
ENSG00000225660	RP1-73A14.1	0.561743	0.200446	1.486696614
ENSG00000254644	RP11-5A11.2	0.806759	0.287875	1.486695261
ENSG00000270480	RP11-57B24.1	0.2072	0.073935	1.486694616
ENSG00000272498	RP11-415F23.3	0.53784	0.191917	1.486694544
ENSG00000250768	DPP3P1	0.036759	0.013117	1.486659722
ENSG00000181035	SLC25A42	1.805031399	0.644151064	1.486552965
ENSG00000274897	PANO1	0.35385	0.126282	1.486488905
ENSG00000141028	CDRT15P1	0.381809	0.136261	1.486478411
ENSG00000267295	CTD-2319I12.3	0.266417	0.095115	1.485941361
ENSG00000188191	PRKAR1B	1.204336202	0.430470142	1.484253112
ENSG00000267698	AC002116.7	1.176628	0.420584	1.484192401
ENSG00000223735	OR51B3P	2.895454	1.034988	1.484175536
ENSG00000234942	GRID1-AS1	0.14419	0.051543	1.484122698
ENSG00000174282	ZBTB4	11.42954724	4.085810563	1.484074035
ENSG00000158882	TOMM40L	3.417444839	1.222134743	1.483514697
ENSG00000175766	EIF4E1B	0.136041956	0.048652544	1.483464517
ENSG00000250989	RP11-392E22.5	0.69289	0.247813	1.483374463
ENSG00000206066	IGLL3P	0.339344	0.121376	1.483265323
ENSG00000188878	FBF1	1.707018888	0.610714543	1.482908917
ENSG00000248126	CTD-2012J19.2	1.787676	0.639606	1.482829907
ENSG00000076356	PLXNA2	0.600787995	0.214974106	1.48269309
ENSG00000261437	RP11-22C11.2	0.41547	0.148751	1.48184494
ENSG00000053438	NNAT	0.465884	0.16691	1.480900392
ENSG00000171103	TRMT61B	8.210080361	2.941766815	1.48071345
ENSG00000267696	ERVK-28	0.24572517	0.088047793	1.48068689
ENSG00000225210	DUXAP9	1.456784951	0.522008781	1.480641944
ENSG00000171861	RNMTL1	6.331421176	2.269124614	1.480393529
ENSG00000078549	ADCYAP1R1	0.066302408	0.023765083	1.480217797
ENSG00000231616	RP11-575L7.4	0.296606802	0.106335623	1.479926693
ENSG00000213020	ZNF611	1.07297258	0.384678152	1.479889409
ENSG00000215007	DNAJA1P3	2.282799	0.818456	1.479827071
ENSG00000235079	ZRANB2-AS1	0.298316961	0.106960054	1.479773908
ENSG00000280816	Metazoa_SRP	2.196987	0.787777	1.479667131
ENSG00000258988	RP11-125H8.1	4.960704	1.778796	1.47964381
ENSG00000259751	RP11-83J16.3	0.506415	0.18162	1.479397057
ENSG00000224216	RP13-228J13.1	0.264375715	0.094833146	1.47912635
ENSG00000158301	GPRASP2	1.515125869	0.543520019	1.479032572
ENSG00000148291	SURF2	12.69434039	4.55387776	1.479017963
ENSG00000198719	DLL1	2.003340054	0.718699466	1.478946812
ENSG00000114554	PLXNA1	12.20518451	4.379675361	1.478598265
ENSG00000102879	CORO1A	3.271204834	1.17428706	1.478036975

ENSG00000227427	RP1-49C23.1	0.23225	0.083433	1.47698857
ENSG00000257017	HP	0.027872832	0.010013049	1.476978288
ENSG00000224080	UBE2FP1	0.674061	0.24215	1.476978153
ENSG00000142166	IFNAR1	9.329407505	3.351596271	1.476937086
ENSG00000142512	SIGLEC10	0.116657446	0.041915119	1.47673576
ENSG00000155085	AK9	0.650942378	0.233989027	1.476088967
ENSG00000106128	GHRHR	0.122067367	0.043882586	1.475957116
ENSG00000104356	POP1	11.04462802	3.97106518	1.475746884
ENSG00000183647	ZNF530	0.38979507	0.140180753	1.475427565
ENSG00000221411	MIR1227	1.319085	0.474495	1.475072743
ENSG00000254991	RP13-631K18.3	0.167348	0.0602	1.475015917
ENSG00000182912	TSPEAR-AS2	0.343019382	0.123407543	1.474859514
ENSG00000139998	RAB15	2.093152642	0.753186247	1.474598962
ENSG00000244528	SEPT14P2	0.687191	0.247279	1.474571417
ENSG00000213287	RP11-680L20.1	0.73171	0.263325	1.474427479
ENSG00000117010	ZNF684	1.832265037	0.659467107	1.474255597
ENSG00000207707	MIR639	0.033387	0.01202	1.473849569
ENSG00000185875	THNSL1	3.451751	1.242715	1.473832925
ENSG00000154040	CABYR	0.128180843	0.046151661	1.473726176
ENSG00000263321	RP11-388C12.5	0.639461	0.230243	1.473699078
ENSG00000197471	SPN	0.061052638	0.021993548	1.472973288
ENSG00000165494	PCF11	5.150014153	1.855518548	1.472753975
ENSG00000179673	RPRML	0.39022	0.140625	1.472434626
ENSG00000267106	ZNF561-AS1	0.866238604	0.31222244	1.472190235
ENSG00000151065	DCP1B	4.775996993	1.721574532	1.472073288
ENSG00000026559	KCNG1	4.108948602	1.48176983	1.47144792
ENSG00000167656	LY6D	0.193266773	0.069697947	1.471405559
ENSG00000272115	GS1-393G12.14	0.600261	0.216482	1.471342864
ENSG00000135454	B4GALNT1	5.206333376	1.878092109	1.470999874
ENSG00000114646	CSPG5	1.365547598	0.49261298	1.470953054
ENSG00000214425	LRRC37A4P	3.488040551	1.259262121	1.469838194
ENSG00000225462	FDPSP1	1.04121	0.375963	1.46959848
ENSG00000280054	RP1-197B17.7	0.306043	0.11054	1.469165853
ENSG00000177335	C8orf31	0.102311334	0.036955386	1.469109402
ENSG00000240137	ERICH6-AS1	0.898574597	0.32459227	1.469009636
ENSG00000008438	PGLYRP1	0.224689	0.081181	1.46871548
ENSG00000143549	TPM3	49.14646207	17.76168537	1.468319082
ENSG00000148300	REXO4	11.94055079	4.31563234	1.468225518
ENSG00000136816	TOR1B	12.61001061	4.557846453	1.468145261
ENSG00000157578	LCA5L	0.245352295	0.088692589	1.467969293
ENSG00000269400	CTD-2529P6.4	0.106701	0.038576	1.467798236
ENSG00000267795	SMIM22	0.028885679	0.01044326	1.467782189
ENSG00000207948	MIR328	0.458524	0.165774	1.467779497
ENSG00000145491	ROPN1L	0.224089037	0.081017533	1.467766017
ENSG00000225605	RP11-550H2.1	0.090805068	0.032830169	1.467750618
ENSG00000109794	FAM149A	1.421391294	0.51393567	1.467644077
ENSG00000237517	DGCR5	0.874727494	0.316280188	1.467630452
ENSG00000203441	LINC00449	0.192612	0.069659	1.467315921

ENSG00000178762	HIST1H2BPS1	0.048269	0.017458	1.467208646
ENSG00000269896	RP4-740C4.5	0.062166511	0.02248467	1.467195921
ENSG00000268621	AC006262.5	0.085456981	0.030908641	1.467188141
ENSG00000232362	ATP5LP2	0.24057	0.087011	1.46718704
ENSG00000186376	ZNF75D	1.184868628	0.428597531	1.467031665
ENSG00000117394	SLC2A1	70.58902997	25.53894193	1.46674524
ENSG00000242498	ARPIN	2.513403973	0.909494872	1.466505162
ENSG00000114473	IQCG	2.335825812	0.845505395	1.466046828
ENSG00000262339	RP11-1197K16.2	0.076182373	0.027577347	1.465973539
ENSG00000236199	RP11-264I13.2	0.916965	0.331974	1.465796413
ENSG00000265735	RN7SL5P	24.585032	8.901557	1.465650623
ENSG00000108379	WNT3	1.259873718	0.456280774	1.465285364
ENSG00000225762	LINC01389	0.30939	0.112051	1.465271043
ENSG00000225154	RP11-184J23.2	0.216287	0.078344	1.465052259
ENSG00000255251	PRR23D1	0.234464	0.084929	1.465037258
ENSG00000255378	PRR23D2	0.234464	0.084929	1.465037258
ENSG00000215914	MMP23A	0.2202	0.079823	1.463938063
ENSG00000050165	DKK3	0.088532734	0.032093862	1.463913545
ENSG00000205885	C1RL-AS1	1.036961973	0.376113812	1.463121798
ENSG00000138400	MDH1B	1.15993632	0.420724279	1.463098623
ENSG00000239829	KRT8P25	0.152133	0.055207	1.462410019
ENSG00000082684	SEMA5B	0.143759223	0.052172343	1.462297398
ENSG00000260083	MIR762HG	0.567939494	0.206242255	1.461397294
ENSG00000168129	AC098817.5	0.244011	0.088749	1.459143417
ENSG00000164093	PITX2	1.773090081	0.644922439	1.459068261
ENSG00000144857	BOC	0.351745535	0.127982389	1.458586808
ENSG00000261215	RP11-195F19.30	0.049122	0.017876	1.458345351
ENSG00000267472	LOC440461	0.219847	0.080059	1.457364345
ENSG00000228022	HCG20	0.10840006	0.039474872	1.457359047
ENSG00000271757	RP11-111M22.5	0.41864	0.152452	1.457355087
ENSG00000130427	EPO	0.111742	0.040692	1.457354452
ENSG00000276351	H19_2	1.076934	0.392178	1.457349325
ENSG00000243339	RN7SL738P	0.500393	0.182224	1.457348624
ENSG00000227598	RP1-167A14.2	0.386018	0.140573	1.457348601
ENSG00000235023	AP001626.2	0.052073	0.018963	1.457348304
ENSG00000270750	RP11-461L13.3	0.29664	0.108025	1.457347918
ENSG00000262873	CTD-2561B21.11	0.158948	0.057882835	1.457347367
ENSG00000237008	LAPTM4BP1	0.215387	0.078436	1.457343307
ENSG00000272862	RP11-814H16.2	0.299028	0.108895	1.457342867
ENSG00000173080	RXFP4	0.119852	0.043646	1.457332637
ENSG00000134243	SORT1	10.56031349	3.848200355	1.456396843
ENSG00000214211	CTAGE14P	0.163258	0.059496	1.456289106
ENSG00000127743	IL17B	0.122626464	0.044690557	1.456228444
ENSG00000266553	RN7SL356P	0.035914	0.013094	1.455640461
ENSG00000179965	ZNF771	0.996238173	0.363304322	1.455312161
ENSG00000204604	ZNF468	3.672092618	1.339210808	1.455219371
ENSG00000225972	MTND1P23	2090.905029	762.629639	1.455073028
ENSG00000147799	ARHGAP39	6.920056218	2.52400039	1.455071625

ENSG00000126804	ZBTB1	4.445640252	1.621608682	1.45496549
ENSG00000132386	SERPINF1	1.026372048	0.374409891	1.454863331
ENSG00000204632	HLA-G	3.499506505	1.277044231	1.454342995
ENSG00000135407	AVIL	0.331806907	0.121087768	1.454290787
ENSG00000230295	GTF2IP23	1.696431	0.619265	1.453873937
ENSG00000159885	ZNF222	2.71383939	0.990872449	1.45356408
ENSG00000272977	CTA-390C10.10	0.86689	0.316592	1.453224141
ENSG00000261777	RP11-529K1.2	0.786124641	0.287111302	1.45314795
ENSG00000224616	RTCA-AS1	0.700555	0.255889	1.452980189
ENSG00000135540	NHSL1	2.282464341	0.834144088	1.452223804
ENSG00000256061	DYX1C1	2.611538212	0.954576626	1.451966897
ENSG00000164576	SAP30L	2.616309985	0.956375328	1.451884667
ENSG00000058056	USP13	8.471003391	3.0973569	1.451495237
ENSG00000224049	RP11-30O15.1	0.327096649	0.119601068	1.451486706
ENSG00000258583	LINC01500	0.195858297	0.071651566	1.450740106
ENSG00000167522	ANKRD11	17.71901378	6.483977523	1.450347312
ENSG00000176422	SPRYD4	0.493049591	0.180451992	1.450117691
ENSG00000178623	GPR35	0.296634458	0.108585515	1.449854533
ENSG00000239608	RUVBL1-AS1	0.251432	0.092042	1.449804037
ENSG00000144834	TAGLN3	0.326305948	0.119481389	1.449439367
ENSG00000132825	PPP1R3D	1.296381	0.47471	1.449371435
ENSG00000100311	PDGFB	10.79300203	3.954208827	1.448635231
ENSG00000111371	SLC38A1	20.51747187	7.518478824	1.448340272
ENSG00000271821	XXbac-BPG299F13.14	0.434153	0.159117	1.448115572
ENSG00000272087	RP11-379F4.7	1.378667	0.505347	1.447927764
ENSG00000269934	RP5-1139B12.3	0.56973	0.208835	1.447914853
ENSG00000043143	JADE2	1.159929296	0.425188836	1.447861247
ENSG00000122687	FTSJ2	9.957986879	3.650946215	1.447583699
ENSG00000013016	EHD3	0.031838	0.011674	1.447450738
ENSG00000183625	CCR3	0.041367275	0.015168161	1.447443709
ENSG00000133574	GIMAP4	0.067304606	0.024678668	1.447440672
ENSG00000214602	CTBP2P5	0.116771	0.042817	1.447426405
ENSG00000229082	OR5AW1P	0.146718	0.053798	1.447421433
ENSG00000116703	PDC	0.110230478	0.040419262	1.447408284
ENSG00000267062	CTD-2659N19.10	2.009067	0.736688	1.447400029
ENSG00000279390	AF127577.13	0.067366	0.024702	1.447392784
ENSG00000113946	CLDN16	0.041194309	0.015105311	1.447389179
ENSG00000164483	SAMD3	0.599973786	0.220021449	1.447255296
ENSG00000261192	RNF126P1	0.321956761	0.118081155	1.447088206
ENSG00000274791	F8A2	1.17116	0.429666	1.446650661
ENSG00000272941	RP11-134L10.1	0.298819	0.109659	1.44624766
ENSG00000183186	C2CD4C	3.085003	1.132224	1.446112482
ENSG00000198176	TFDP1	53.95324595	19.80406724	1.445913006
ENSG00000184209	SNRNP35	1.984993933	0.728837275	1.445465948
ENSG00000225075	RP11-426L16.3	0.899315	0.330357	1.444800618
ENSG00000161057	PSMC2	36.23650336	13.31322791	1.444583341
ENSG00000182759	MAFA	1.870395413	0.687313889	1.444302279
ENSG00000143942	CHAC2	5.086153	1.869022	1.44429131

ENSG00000161202	DVL3	18.88282675	6.940652571	1.443931533
ENSG00000087494	PTHLH	0.294194131	0.108160569	1.443593825
ENSG00000178184	PARD6G	8.807799156	3.238600786	1.44341093
ENSG00000166106	ADAMTS15	0.589349	0.21675	1.443090227
ENSG00000007314	SCN4A	1.241161794	0.456615088	1.442640756
ENSG00000187372	PCDHB13	0.043578	0.01604	1.441925844
ENSG00000081692	JMJD4	1.78801257	0.658426655	1.441262233
ENSG00000166002	SMCO4	8.701999795	3.204560958	1.441220269
ENSG00000229589	ACVR2B-AS1	0.631678	0.232651	1.441021939
ENSG00000221817	PPP3CB-AS1	0.595235148	0.219263325	1.440795196
ENSG00000132763	MMACHC	4.297436	1.584092	1.439820029
ENSG00000176318	FOXN3P1	0.154408	0.056924	1.439638554
ENSG00000232852	CICP4	0.139443	0.051417	1.439358172
ENSG00000136827	TOR1A	8.662983791	3.19477532	1.439149544
ENSG00000161681	SHANK1	1.144665591	0.422258492	1.43872784
ENSG00000228956	SATB1-AS1	0.007386671	0.002725072	1.43863001
ENSG00000227471	AKR1B15	0.036757455	0.013563748	1.438280988
ENSG00000270497	RP1-290F12.3	0.886579	0.327342	1.437450469
ENSG00000237390	RP11-139I14.2	0.026241	0.009689	1.437403022
ENSG00000136492	BRIP1	5.326895171	1.966924694	1.437353167
ENSG00000170500	LONRF2	0.496358752	0.183341249	1.436851828
ENSG00000248643	RBM14-RBM4	2.932703517	1.084103281	1.435729022
ENSG00000130520	LSM4	38.55079822	14.25377518	1.435416657
ENSG00000255463	RP11-16F15.4	1.662802	0.615006	1.434943998
ENSG00000267796	LIN37	2.414330329	0.892975351	1.434930822
ENSG00000205662	RP11-706O15.7	2.688688	0.994627	1.434674852
ENSG00000183921	SDR42E2	0.363619	0.134582	1.43394212
ENSG00000265043	RP11-728E14.3	0.116829	0.043262	1.433226165
ENSG00000215319	EIF5P1	3.083955	1.142203	1.432962635
ENSG00000148297	MED22	2.223837548	0.823971493	1.432385072
ENSG00000253641	LINCR-0001	0.712851081	0.264176763	1.432097147
ENSG00000167685	ZNF444	2.107372595	0.781195098	1.431690611
ENSG00000267216	AC010642.1	1.285882	0.47671	1.431574464
ENSG00000268288	RP11-98D18.16	1.133615	0.420354	1.431254047
ENSG00000269425	AC007292.7	1.780455	0.660487	1.430643902
ENSG00000157601	MX1	0.276713422	0.102681437	1.430217238
ENSG00000198346	ZNF813	0.035450014	0.013158057	1.429839736
ENSG00000197702	PARVA	7.741090191	2.873356988	1.429799512
ENSG00000136874	STX17	4.366376155	1.621417222	1.429181049
ENSG00000007968	E2F2	4.190871898	1.557213365	1.428283792
ENSG00000268006	PTOV1-AS1	3.609067456	1.34125385	1.428043796
ENSG00000238058	RP11-432J22.2	1.588793	0.590525	1.427861129
ENSG00000205808	PLPP6	2.188675	0.813646	1.427584592
ENSG00000279631	RP11-573G6.4	0.057809	0.021493	1.427427248
ENSG00000161914	ZNF653	1.165037659	0.43316108	1.427401063
ENSG00000009709	PAX7	0.023058186	0.008573242	1.427366338
ENSG00000171873	ADRA1D	0.052165422	0.019397156	1.427248727
ENSG00000132801	ZSWIM3	0.786874	0.292754	1.426443756

ENSG00000198169	ZNF251	5.235739474	1.948028906	1.426378227
ENSG00000168484	SFTPC	0.063859809	0.023766939	1.425952166
ENSG00000226510	UPK1A-AS1	2.709342	1.008889	1.425175061
ENSG00000279672	CMB9-55F22.1	0.647634635	0.241380427	1.423871447
ENSG00000223496	EXOSC6	4.352532	1.622259	1.423850735
ENSG00000227456	LINC00310	0.088503868	0.032999882	1.423279634
ENSG00000162188	GNG3	2.07154	0.773151	1.421881566
ENSG00000230303	RP11-213G2.2	0.24375346	0.090988712	1.421663222
ENSG00000173868	PHOSPHO1	3.031075438	1.13169883	1.421339683
ENSG00000241015	TPM3P9	0.604459433	0.225687545	1.42131872
ENSG00000135299	ANKRD6	2.427499894	0.906622282	1.420897708
ENSG00000248508	SRP14-AS1	1.654518446	0.618140914	1.420403713
ENSG00000142556	ZNF614	0.823549783	0.307826387	1.419738954
ENSG00000101346	POFUT1	12.29997547	4.598433907	1.419440928
ENSG00000235254	TMEM185AP1	4.228866	1.580994	1.419438954
ENSG00000231551	RP11-495P10.1	0.605718874	0.22650639	1.419096618
ENSG00000238113	LINC01410	0.327037592	0.122299761	1.419034893
ENSG00000119004	CYP20A1	0.954907116	0.357211109	1.418583459
ENSG00000249693	THEGL	0.080617178	0.030184049	1.41730093
ENSG00000267264	CTC-459F4.6	0.276378	0.103481	1.417276879
ENSG00000150627	WDR17	0.018512019	0.006931253	1.417274253
ENSG00000243396	RP11-454H13.1	0.359565	0.134628	1.417274102
ENSG00000274765	RP11-139H15.7	0.332288	0.124416	1.417262164
ENSG00000245067	IGFBP7-AS1	0.083071779	0.031104021	1.417257372
ENSG00000053770	AP5M1	3.765686126	1.410410125	1.416798018
ENSG00000274333	RP11-717F1.2	0.630589582	0.236248124	1.416398463
ENSG00000143344	RGL1	2.16747806	0.812063242	1.416353398
ENSG00000272068	RP11-284F21.9	5.281074	1.978709	1.4162719
ENSG00000151466	SCLT1	1.983824353	0.743509394	1.415861421
ENSG00000125871	MGME1	18.52859969	6.944851	1.415738206
ENSG00000139269	INHBE	0.330413324	0.123858088	1.415583782
ENSG00000206785	SNORA15	0.872049	0.326936	1.415400956
ENSG00000171823	FBXL14	2.391955552	0.897483271	1.414233629
ENSG00000106823	ECM2	0.041000637	0.015383824	1.414232149
ENSG00000256101	RP11-90D4.3	0.424763972	0.159384733	1.414147956
ENSG00000152926	ZNF117	1.48785382	0.558383636	1.413904224
ENSG00000201643	SNORA14A	0.256366	0.096214	1.413886201
ENSG00000214888	XXyac-YM21GA2.7	0.107402	0.040308	1.413882752
ENSG00000233421	RP5-875O13.1	0.069631059	0.026132871	1.413865329
ENSG00000227001	NBPF2P	1.95472	0.734025	1.413060861
ENSG00000257758	KRT18P20	0.75947	0.285267	1.412680114
ENSG00000275891	MIR7110	1.641535	0.616722	1.412353294
ENSG00000186141	POLR3C	7.010804393	2.635120219	1.411711201
ENSG00000198746	GPATCH3	4.456806253	1.6754258	1.41148245
ENSG00000158423	RIBC1	0.288468306	0.108493905	1.410798818
ENSG00000123643	SLC36A1	6.641310499	2.497984429	1.410703466
ENSG00000206120	EGFEM1P	0.110576947	0.04159543	1.410553721
ENSG00000214652	ZNF727	0.032118	0.012087	1.409925847

ENSG00000238142	RP11-108M9.4	0.334966283	0.126067575	1.409818631
ENSG00000176894	PXMP2	5.690970358	2.141918894	1.409770813
ENSG00000164815	ORC5	9.937796319	3.741683643	1.409238389
ENSG00000172831	CES2	6.336404253	2.385876574	1.409144969
ENSG00000232830	RP11-144C15.1	1.061801	0.399905	1.408784181
ENSG00000170689	HOXB9	13.013826	4.901395	1.408780847
ENSG00000272141	RP11-465B22.8	1.446958	0.545044	1.408578441
ENSG00000228434	AC004951.6	3.10004	1.167791	1.408504733
ENSG00000027869	SH2D2A	1.058446646	0.398779397	1.408285768
ENSG00000101265	RASSF2	7.33810275	2.76485625	1.408202631
ENSG00000240912	RP11-274J15.2	0.083994	0.03165	1.408080775
ENSG00000278878	CH507-145C22.3	0.333352528	0.125706222	1.406992603
ENSG00000104812	GYS1	14.63155325	5.518905364	1.406628878
ENSG00000186226	LCE1E	0.110405754	0.041663356	1.405964395
ENSG00000140090	SLC24A4	0.028252422	0.010664561	1.40554998
ENSG00000040275	SPDL1	7.618011975	2.875843554	1.405429361
ENSG00000133878	DUSP26	0.34228446	0.129235151	1.405197266
ENSG00000101888	NXT2	4.618309617	1.743727139	1.405190594
ENSG00000237788	RP5-1041C10.3	0.130038	0.049111	1.404815168
ENSG00000276256	AC011043.1	3.162707	1.195157	1.40395976
ENSG00000250829	RP11-11N5.1	1.412279	0.533795	1.403667428
ENSG00000170270	C14orf142	8.561502	3.235977	1.403662566
ENSG00000139178	C1RL	5.002400598	1.891501999	1.403088293
ENSG00000112186	CAP2	2.826535348	1.069023316	1.40274142
ENSG00000213937	CLDN9	0.488604	0.184802	1.402685304
ENSG00000214827	MTCP1	0.49003371	0.185349253	1.402634697
ENSG00000231087	FDPSP7	0.271462	0.102696	1.40237027
ENSG00000272610	MAGI1-IT1	0.334285	0.126499	1.40195264
ENSG00000121380	BCL2L14	0.055903338	0.021174169	1.400629057
ENSG00000131153	GINS2	11.41640667	4.325034492	1.400325088
ENSG00000009954	BAZ1B	25.56371452	9.685174636	1.400247513
ENSG00000272277	RP1-40E16.12	0.504079	0.191014	1.399971471
ENSG00000281220	AC011404.1	1.037651	0.393251	1.399798955
ENSG00000188152	NUTM2G	0.443825825	0.168215102	1.399686383
ENSG00000253187	HOXA10-AS	2.335002839	0.885069971	1.399560884
ENSG00000168491	CCDC110	1.172862473	0.444720682	1.399062452
ENSG00000259165	DDX18P1	0.274665	0.104147	1.3990518
ENSG00000196199	MPHOSPH8	10.65910935	4.041819517	1.399010088
ENSG00000257365	FNTB	6.319411286	2.396529428	1.398843508
ENSG00000258734	RP11-112J1.3	1.825314	0.692264	1.398750435
ENSG00000271662	RP11-141C7.3	1.24021	0.470442	1.398495656
ENSG00000188282	RUFY4	0.144733318	0.054902324	1.398457961
ENSG00000259881	RP11-830F9.5	0.140007742	0.053111248	1.398417283
ENSG00000254433	RP11-677I18.3	0.379811706	0.14415919	1.397621562
ENSG00000183044	ABAT	2.332110479	0.885588258	1.396928137
ENSG00000156097	GPR61	0.028181167	0.010702226	1.396820411
ENSG00000255374	TAS2R43	0.063963478	0.024291224	1.396813219
ENSG00000240823	RN7SL23P	0.518216	0.196802	1.396808678

ENSG00000272473	AC006273.4	0.165516	0.062858	1.396802411
ENSG00000177076	ACER2	2.736677	1.039466	1.396582599
ENSG00000130021	PUDP	5.184616466	1.969319001	1.396540443
ENSG00000169683	LRRC45	5.491819803	2.086378183	1.3962836
ENSG00000136026	CKAP4	36.11230119	13.72157807	1.396043946
ENSG00000198753	PLXNB3	0.541996673	0.20594479	1.396026365
ENSG00000229563	LINC01204	0.080143504	0.030455831	1.395867094
ENSG00000157637	SLC38A10	13.31000462	5.059096874	1.395559302
ENSG00000135999	EPC2	5.069388875	1.926927695	1.3955094
ENSG00000229798	KRT18P26	0.13583	0.051649	1.394989833
ENSG00000225774	SIRPAP1	0.319179	0.121389	1.394728041
ENSG00000237476	XXbac-B135H6.15	0.832165	0.316495	1.394686903
ENSG00000248593	DSTNP2	2.821191	1.073066	1.394565529
ENSG00000225828	FAM229A	4.462487775	1.69765385	1.394305892
ENSG00000167395	ZNF646	2.84954	1.084553033	1.393628445
ENSG00000146205	ANO7	0.120040355	0.045709536	1.392952413
ENSG00000188295	ZNF669	3.627917598	1.382175984	1.392200371
ENSG00000228838	RP4-784A16.2	0.206125	0.078575	1.391377222
ENSG00000236287	ZBED5	2.95257361	1.125914812	1.390875352
ENSG00000164292	RHOBTB3	55.99431913	21.35269194	1.390862505
ENSG00000166272	WBP1L	12.02114071	4.590035201	1.38899668
ENSG00000268516	CTD-3138B18.5	1.449292	0.553646	1.388312575
ENSG00000140465	CYP1A1	0.880491289	0.336436919	1.387972704
ENSG00000117461	PIK3R3	0.716731874	0.273889843	1.387837749
ENSG00000254016	ALG1L10P	0.95063	0.363363	1.387472409
ENSG00000222051	RNU6-1165P	0.846685	0.323711	1.38711894
ENSG00000266229	AC020606.1	0.444094	0.16979	1.387113588
ENSG00000239480	RP11-514P8.2	0.37443	0.143191	1.386755213
ENSG00000267360	CTC-454I21.3	0.085555655	0.032721115	1.386641291
ENSG00000198908	BHLHB9	0.952581083	0.364562251	1.385676715
ENSG00000185247	MAGEA11	0.029377841	0.011245004	1.385444172
ENSG00000258952	SALRNA1	0.048798	0.01868	1.385327565
ENSG00000259786	CTD-2118P12.1	0.036148055	0.013837652	1.38531881
ENSG00000281079	AC055866.1	1.028206	0.393604	1.385312549
ENSG00000225152	RP11-338O1.2	0.122346	0.046835	1.385307964
ENSG00000235211	TMSB10P2	0.38852	0.148729	1.385302887
ENSG00000223695	RP4-633O19__A.1	0.099796	0.038203	1.385296056
ENSG00000272717	RP11-342I1.2	0.120464	0.046115	1.385294066
ENSG00000167985	SDHAF2	9.800284095	3.751886034	1.385207566
ENSG00000175283	DOLK	5.712064	2.187091	1.384998895
ENSG00000254912	RP11-632K20.2	0.34581	0.132432	1.384727821
ENSG00000224425	AC073869.2	0.618899	0.237019	1.384701277
ENSG00000114166	KAT2B	2.940107596	1.126431784	1.384109005
ENSG00000229178	AC069513.4	0.335479	0.128553	1.383859183
ENSG00000243364	EFNA4	5.952598231	2.281376	1.383615283
ENSG00000030419	IKZF2	0.219052463	0.084010208	1.382639885
ENSG00000167771	RCOR2	3.177148974	1.218799442	1.382271994
ENSG00000201772	SNORA5C	0.72072	0.276528	1.382013312

ENSG00000258787	RP11-164C12.1	1.135377	0.435675	1.381847186
ENSG00000183723	CMTM4	8.185300594	3.14159134	1.381539872
ENSG00000205266	KRT17P5	0.058333586	0.022391687	1.381363545
ENSG00000170382	LRRN2	1.014341093	0.389637542	1.380338277
ENSG00000206824	Y_RNA	0.82831	0.318356	1.379529851
ENSG00000156574	NODAL	0.388184965	0.149200916	1.379487851
ENSG00000267469	AC005944.2	3.483131	1.338813	1.379430269
ENSG00000168404	MLKL	1.139414473	0.437991495	1.379317877
ENSG00000276102	MIR6747	3.3458	1.286472	1.378931152
ENSG00000198668	CALM1	39.16803322	15.06054932	1.378902295
ENSG00000136867	SLC31A2	0.633645067	0.243703791	1.378546254
ENSG00000100276	RASL10A	0.314011802	0.120805018	1.378138401
ENSG00000105939	ZC3HAV1	10.68687815	4.112916055	1.377606943
ENSG00000114993	RTKN	7.548898538	2.905687175	1.377386764
ENSG00000177663	IL17RA	4.639759886	1.786356918	1.377029783
ENSG00000168679	SLC16A4	0.621732144	0.239502858	1.376250295
ENSG00000181392	SYNE4	3.832596058	1.476547417	1.376094265
ENSG00000232378	RPL29P28	0.049602	0.01911	1.376070514
ENSG00000173698	ADGRG2	0.013832924	0.005329414	1.37605718
ENSG00000272343	RP11-140I16.3	0.289041	0.111359	1.37605599
ENSG00000274210	U1	0.851355	0.328003	1.376051826
ENSG00000274104	CTD-2553L13.10	0.260137	0.100224	1.376043589
ENSG00000277034	SNORA71	4.740246	1.826298	1.37603974
ENSG00000177971	IMP3	17.52109897	6.751515554	1.375809973
ENSG00000282221	RP11-27G14.4	2.204145	0.849438	1.37563858
ENSG00000203593	RP5-1096D14.6	0.323664	0.124761	1.375329887
ENSG00000231970	RP11-452K12.7	1.716745	0.661903	1.374984047
ENSG00000175322	ZNF519	0.497300419	0.19179673	1.374539525
ENSG00000068305	MEF2A	13.11737707	5.059397625	1.374441738
ENSG00000150347	ARID5B	5.696460126	2.197638278	1.374111739
ENSG00000047932	GOPC	17.74494466	6.847160506	1.373830339
ENSG00000227354	RBM26-AS1	0.387041238	0.14936394	1.373655399
ENSG00000276943	AL023284.1	0.856807	0.330724	1.373342524
ENSG00000183615	FAM167B	0.449046	0.173463	1.372235275
ENSG00000203965	EFCAB7	1.867148025	0.721538782	1.371687462
ENSG00000232940	HCG25	1.344035493	0.519397306	1.371660801
ENSG00000233496	SYNJ2-IT1	0.056079	0.021684	1.370829711
ENSG00000278619	MRM1	2.134242	0.82536	1.370628345
ENSG00000266970	RP11-806H10.4	1.340178872	0.518933752	1.368803291
ENSG00000272509	RP11-347C18.5	1.119276	0.433433	1.368684927
ENSG00000198959	TGM2	15.69010431	6.076229409	1.368606698
ENSG00000080644	CHRNA3	0.299418954	0.115988708	1.368181197
ENSG00000137869	CYP19A1	0.088691174	0.03435777	1.368154124
ENSG00000229197	RP11-227H15.7	0.048598	0.018829	1.367940561
ENSG00000205663	RP11-706O15.5	2.195608519	0.850697559	1.367902622
ENSG00000246898	LINC00920	1.554706	0.60246	1.367704425
ENSG00000135363	LMO2	1.437011791	0.557046349	1.367202624
ENSG00000196562	SULF2	15.02828988	5.82693321	1.36687217

ENSG00000258429	PDF	9.721024	3.769743	1.366642121
ENSG00000168481	LGI3	1.879460687	0.729084336	1.366161127
ENSG00000054598	FOXC1	44.620392	17.314022	1.36576229
ENSG00000168268	NT5DC2	15.03173055	5.833086894	1.365679641
ENSG00000163083	INHBB	0.739933	0.287156	1.365559936
ENSG00000259774	RP11-182J1.13	2.385281	0.925825	1.365347809
ENSG00000248256	OCIAD1-AS1	0.543436	0.210961	1.365133825
ENSG00000109084	TMEM97	23.32096846	9.054506622	1.364919765
ENSG00000092529	CAPN3	0.335995567	0.130463748	1.364793215
ENSG00000104221	BRF2	2.770886279	1.076189025	1.364416003
ENSG00000249744	CTD-2057J6.2	0.78136	0.30348	1.364385959
ENSG00000198824	CHAMP1	16.126189	6.26401918	1.364245001
ENSG00000101417	PXMP4	1.729919391	0.671991223	1.36419052
ENSG00000173638	SLC19A1	3.361644887	1.306405661	1.363564383
ENSG00000170633	RNF34	9.900700187	3.849409923	1.362893246
ENSG00000088367	EPB41L1	12.2184103	4.75094164	1.362771203
ENSG00000269102	CTD-2525I3.5	0.311377	0.121075	1.36276138
ENSG00000261512	RP11-46D6.1	7.882354	3.065923	1.362305079
ENSG00000130881	LRP3	4.700010379	1.829454465	1.361250435
ENSG00000235165	CDK5PS	0.237747	0.092567	1.360857265
ENSG00000180422	LINC00304	0.315064695	0.122697439	1.360542967
ENSG00000169508	GPR183	0.238913	0.093051	1.360391798
ENSG00000123342	MMP19	0.118961918	0.04633574	1.360302502
ENSG00000182405	PGBD4	0.721414	0.281079	1.35985175
ENSG00000140265	ZSCAN29	3.67876444	1.433697087	1.359481058
ENSG00000112242	E2F3	5.99572986	2.33752756	1.358952009
ENSG00000204620	RP11-1148L6.9	0.448191	0.17478	1.358573571
ENSG00000277363	SRCIN1	2.026172509	0.790382941	1.358133296
ENSG00000100162	CENPM	5.378861826	2.098233422	1.358125746
ENSG00000114853	ZBTB47	1.380296	0.538705	1.357410321
ENSG00000204882	GPR20	1.032621	0.403053	1.357269378
ENSG00000221971	TTC4P1	0.458857	0.179155	1.356836309
ENSG00000229544	NKX1-2	4.305298	1.681362	1.356482728
ENSG00000163888	CAMK2N2	2.333231	0.911546	1.35594178
ENSG00000184571	PIWIL3	0.036419303	0.014230681	1.355698591
ENSG00000111554	MDM1	2.288363851	0.894273931	1.355527734
ENSG00000104883	PEX11G	0.566618604	0.221502967	1.355051947
ENSG00000125735	TNFSF14	0.028973519	0.011326416	1.355043467
ENSG00000272524	RP11-254F7.4	0.416981	0.163013	1.354994626
ENSG00000135048	TMEM2	7.508833779	2.935612064	1.354927524
ENSG00000168427	KLHL30	0.597204	0.233564	1.354405905
ENSG00000258430	RP11-982M15.2	5.050496	1.97579	1.353995463
ENSG00000244642	RN7SL396P	13.49396	5.279771	1.353766528
ENSG00000225082	DAP3P1	0.457699	0.179104	1.353601582
ENSG00000078687	TNRC6C	4.162642602	1.629020124	1.35349527
ENSG00000168040	FADD	11.878979	4.649615	1.353227674
ENSG00000228020	HNRNPA1P46	0.280282	0.109742	1.352763322
ENSG00000184436	THAP7	4.770309977	1.868174028	1.352454162

ENSG00000254088	SLC2A3P4	0.547829	0.214618	1.351954559
ENSG00000196652	ZKSCAN5	5.217878234	2.044476087	1.351732089
ENSG00000131378	RFTN1	1.365011994	0.534990247	1.351329133
ENSG00000112079	STK38	16.007017	6.274108	1.35122221
ENSG00000248180	GAPDHP60	1.551354	0.60838	1.350483298
ENSG00000124116	WFDC3	0.229403486	0.090013482	1.349674313
ENSG00000204315	FKBPL	4.508004	1.768877	1.349655063
ENSG00000114770	ABCC5	5.443955851	2.136183997	1.34961945
ENSG00000235750	KIAA0040	33.87940545	13.29459031	1.349569238
ENSG00000179041	RRS1	19.568424	7.679026	1.349532331
ENSG00000113790	EHHADH	4.128121776	1.620622277	1.348937653
ENSG00000201558	RNVU1-6	0.403445	0.158401	1.348790567
ENSG00000244705	RP11-133K1.1	0.141077	0.05539	1.348785359
ENSG00000233691	RP11-312J18.7	0.333845	0.131173	1.34770764
ENSG00000243479	MNX1-AS1	2.632326	1.034581	1.347291569
ENSG00000186017	ZNF566	1.013726545	0.398492825	1.347042879
ENSG00000240053	LY6G5B	1.444020688	0.567703776	1.346881169
ENSG00000254829	RP11-7I15.3	0.899843	0.353783	1.346808584
ENSG00000272692	RP11-399K21.14	0.267676	0.105254	1.34661273
ENSG00000096070	BRPF3	6.754769143	2.656274926	1.34650199
ENSG00000170265	ZNF282	6.914685069	2.722080898	1.344953602
ENSG00000266195	RN7SL163P	0.497897	0.196088	1.344346073
ENSG00000142623	PADI1	0.028027245	0.011038326	1.344308526
ENSG00000253540	FAM86HP	1.358116729	0.534938832	1.344161644
ENSG00000016391	CHDH	3.460282146	1.36307768	1.344021896
ENSG00000173546	CSPG4	7.140168	2.814244	1.343210601
ENSG00000281814	AC090950.1	49.933586	19.68395	1.342990761
ENSG00000182557	SPNS3	0.400193131	0.157847682	1.342163327
ENSG00000023902	PLEKHO1	1.822404383	0.718846084	1.342088317
ENSG00000265597	AL590764.1	0.501662	0.197961	1.341499426
ENSG00000186063	AIDA	13.53964937	5.343957473	1.341209946
ENSG00000257243	RP11-133N21.7	0.76015	0.300114	1.340773512
ENSG00000205579	DYNLL1P1	8.74931	3.45446	1.340709039
ENSG00000168079	SCARA5	0.18292746	0.072241963	1.340362661
ENSG00000101307	SIRPB1	0.527261402	0.208242913	1.340250988
ENSG00000038427	VCAN	20.5242431	8.107310109	1.340033786
ENSG00000256537	SMIM10L1	3.284307	1.297349	1.340022358
ENSG00000084731	KIF3C	3.589465421	1.417893561	1.340019764
ENSG00000140391	TSPAN3	20.80877071	8.220665052	1.339864721
ENSG00000183309	ZNF623	6.972200346	2.75449812	1.339824548
ENSG00000263316	RP11-530N7.3	1.455861	0.575167	1.33981981
ENSG00000150401	DCUN1D2	4.862211775	1.921004586	1.339751769
ENSG00000198153	ZNF849P	0.133615	0.0528	1.339472143
ENSG00000204388	HSPA1B	45.941055	18.156895	1.339266478
ENSG00000235325	AC009963.6	1.925538	0.761102	1.339099878
ENSG00000140932	CMTM2	0.274681696	0.108590742	1.338859666
ENSG00000272604	RP11-251G23.5	1.380397	0.545824	1.338575507
ENSG00000185275	CD24P4	2.980465	1.178922	1.338069162

ENSG00000108244	KRT23	21.52553151	8.514690559	1.338022856
ENSG00000159217	IGF2BP1	0.165221062	0.065355426	1.338018691
ENSG00000260423	RP13-735L24.1	0.259817	0.102781	1.337922236
ENSG00000139428	MMAB	1.785886236	0.706951818	1.336956384
ENSG00000269791	SSX4B	0.055476214	0.021964125	1.336720314
ENSG00000247271	ZBED5-AS1	2.192491054	0.868098916	1.336639611
ENSG00000203739	RP11-296O14.3	0.238811965	0.094565318	1.33649205
ENSG00000197245	FAM110D	0.139617	0.055295	1.33625368
ENSG00000213779	RP11-452G18.1	0.252799	0.100135	1.336044432
ENSG00000107816	LZTS2	9.957075478	3.944174902	1.335998538
ENSG00000116132	PRRX1	0.016567503	0.006563154	1.335894841
ENSG00000274460	CTD-2649C14.2	0.661548	0.262072	1.335882615
ENSG00000264920	RP11-6N17.4	0.126825203	0.050255795	1.335479606
ENSG00000123297	TSFM	7.274658635	2.883606404	1.335005292
ENSG00000265728	RP11-883A18.3	1.085816	0.430433	1.334919054
ENSG00000204070	SYS1	2.978731559	1.181492284	1.334087907
ENSG00000082458	DLG3	7.088146098	2.811976983	1.333823555
ENSG00000189233	NUGGC	0.034453802	0.013669791	1.33367207
ENSG00000243847	RN7SL610P	0.468735	0.185981	1.333617282
ENSG00000142700	DMRTA2	0.042295514	0.01678222	1.333571057
ENSG00000147883	CDKN2B	4.446508267	1.764541268	1.333379697
ENSG00000176396	EID2	6.571033	2.608495	1.332902519
ENSG00000163866	SMIM12	2.108532441	0.837156199	1.332670483
ENSG00000228604	RP5-872K7.7	0.083451411	0.033154365	1.331737622
ENSG00000184986	TMEM121	0.778412901	0.309259626	1.33171712
ENSG00000172594	SMPDL3A	1.048474483	0.41662248	1.331479158
ENSG00000278266	RP11-575F12.3	2.856643	1.135185	1.33139332
ENSG00000260103	RP11-10O17.1	0.562687	0.223837	1.329884101
ENSG00000264230	ANXA8L1	0.133592323	0.053148096	1.329747192
ENSG00000111879	FAM184A	0.451884236	0.179777128	1.32974374
ENSG00000280018	CH507-154B10.2	0.834725749	0.332213055	1.329193508
ENSG00000178878	APOLD1	0.49007677	0.195056926	1.329112534
ENSG00000119574	ZBTB45	2.871865613	1.14313234	1.328995807
ENSG00000276470	NPPA-AS1_1	0.298611	0.118878	1.328785562
ENSG00000257539	HSPA8P14	0.587063	0.233872	1.327796185
ENSG00000064012	CASP8	4.705112239	1.874472119	1.327744772
ENSG00000113761	ZNF346	2.348885302	0.936013219	1.327375458
ENSG00000175518	UBQLNL	0.176036	0.070195	1.326430319
ENSG00000280027	RP11-44F14.10	0.293986	0.117228	1.326430254
ENSG00000266569	RN7SL377P	0.351514	0.140168	1.326425126
ENSG00000134242	PTPN22	0.180844158	0.072112741	1.32642091
ENSG00000258593	CTD-3051D23.4	0.389636	0.15537	1.326419016
ENSG00000124785	NRN1	0.174900698	0.069742844	1.326418953
ENSG00000269274	AC078899.3	0.060972	0.024313	1.32641895
ENSG00000229184	ATP5HP2	0.979446	0.390561	1.32641806
ENSG00000282965	RP11-683L23.7	0.274743495	0.109555972	1.3264172
ENSG00000257894	RP1-78O14.1	0.180723577	0.072064891	1.326416251
ENSG00000224598	RPS5P2	0.318101	0.126846	1.326406881

ENSG00000147113	CXorf36	0.030295678	0.012080833	1.326392024
ENSG00000185958	FAM186A	0.049079412	0.019571866	1.32633665
ENSG00000213117	RP3-352A20.1	0.584024	0.232934	1.32610642
ENSG00000213625	LEPROT	8.067300476	3.21784109	1.32599291
ENSG00000037897	METTL1	5.107329992	2.037509169	1.325762725
ENSG00000227159	DDX11L16	0.245828	0.098074	1.325706624
ENSG00000178503	NECAP1P1	0.960896	0.383408	1.325499853
ENSG00000200059	Y_RNA	1.66736	0.665683	1.3246584
ENSG00000111321	LTBR	11.11715101	4.438553888	1.324625498
ENSG00000006377	DLX6	0.012296907	0.004911949	1.323927999
ENSG00000185551	NR2F2	15.55641887	6.214196691	1.323870175
ENSG00000273784	RP11-78J21.7	0.800753625	0.319901497	1.323730679
ENSG00000274641	HIST1H2BO	0.484263	0.193501	1.323449756
ENSG00000231625	CTD-2104P17.1	0.107764181	0.043062576	1.323371202
ENSG00000260144	RP11-361M10.3	0.113387	0.045327	1.322812659
ENSG00000162669	HFM1	0.069701678	0.027863916	1.32279537
ENSG00000131591	C1orf159	1.798925103	0.719164775	1.322740859
ENSG00000188033	ZNF490	1.101215454	0.440355117	1.322357425
ENSG00000260097	SPDYE6	1.542672	0.616894	1.322336832
ENSG00000185015	CA13	1.986248322	0.794511912	1.321905246
ENSG00000215210	RBMXP2	8.906372	3.562936	1.321771302
ENSG00000259540	RP11-526I2.1	0.642162	0.256901544	1.321721733
ENSG00000273306	RP11-527J8.1	2.583899	1.033997	1.321317679
ENSG00000186765	FSCN2	0.760384303	0.304296686	1.321250126
ENSG00000177459	ERICH5	6.09528623	2.439778562	1.320943758
ENSG00000188199	NUTM2B	0.48053413	0.192397693	1.320547404
ENSG00000157014	TATDN2	7.764088426	3.109364558	1.320196773
ENSG00000237757	EEF1A1P30	0.381498	0.152854	1.319521188
ENSG00000165156	ZHX1	5.305033349	2.125672805	1.319442274
ENSG00000258811	CTD-3051D23.1	0.124503	0.049896	1.319184436
ENSG00000101846	STS	0.414131	0.165989	1.318999562
ENSG00000227954	TARID	0.248016468	0.099417487	1.318864376
ENSG00000254741	RP11-661A12.7	3.675827	1.474211	1.31812584
ENSG00000130349	C6orf203	5.955319379	2.388797906	1.317894075
ENSG00000198331	HYLS1	4.839897987	1.94147692	1.317822083
ENSG00000221184	MIR1254-1	3.337113	1.339064	1.317375624
ENSG00000111846	GCNT2	0.134051932	0.053809716	1.316853425
ENSG00000101181	MTG2	4.18201203	1.679020557	1.316577318
ENSG00000272097	RP11-421M1.8	0.878980897	0.352985534	1.316222753
ENSG00000085552	IGSF9	3.099065466	1.244733347	1.315996518
ENSG00000174292	TNK1	3.339947497	1.341486036	1.315993387
ENSG00000238160	AC116366.5	0.045852627	0.018419189	1.315794824
ENSG00000007129	CEACAM21	0.205762048	0.082669885	1.315543114
ENSG00000236618	PITPNA-AS1	6.224469	2.501009	1.315440519
ENSG00000235560	AC002310.12	1.905473454	0.765843349	1.315028281
ENSG00000169258	GPRIN1	9.259086	3.721951	1.314810724
ENSG00000275180	RP11-631N16.4	1.096704	0.440901	1.314647541
ENSG00000279179	RP11-131M11.3	0.351355	0.141265	1.314525363

ENSG00000254635	WAC-AS1	6.734357495	2.708905316	1.313830341
ENSG00000271198	VDAC3P1	1.820288	0.732219	1.313819612
ENSG00000267731	RP11-147L13.8	0.702329	0.282561	1.313584653
ENSG00000265252	MIR3132	1.506528	0.60615	1.313480729
ENSG00000260978	MKRN3-AS1	0.712881	0.286858	1.313324517
ENSG00000114904	NEK4	7.617859162	3.066079321	1.312990595
ENSG00000233615	HNRNPA1P42	0.210711	0.084829	1.312636171
ENSG00000116141	MARK1	2.041241953	0.821859897	1.312482815
ENSG00000250220	RP11-692D12.1	0.196155	0.079008	1.311923464
ENSG00000228701	TNKS2-AS1	1.059390159	0.426705733	1.311920612
ENSG00000173662	TAS1R1	0.046461	0.018714	1.311902248
ENSG00000007541	PIGQ	1.419150849	0.571707079	1.311679891
ENSG00000167925	GHDC	3.019383248	1.216489079	1.31153052
ENSG00000272949	RP11-514P8.8	0.769957	0.310286	1.311179273
ENSG00000011114	BTBD7	5.165408333	2.082104499	1.310839922
ENSG00000204815	TTC25	0.747563806	0.301343077	1.310789799
ENSG00000178951	ZBTB7A	15.63766525	6.305255064	1.310398493
ENSG00000140836	ZFHX3	1.787306477	0.720816443	1.310083214
ENSG00000236869	RP11-944L7.4	0.588513	0.237371	1.309930595
ENSG00000279789	CTD-2574D22.3	1.879958	0.75828	1.309897854
ENSG00000131730	CKMT2	0.216412217	0.087346965	1.30895247
ENSG00000267308	AC004510.3	0.210319502	0.084892842	1.308867821
ENSG00000122863	CHST3	5.069544	2.04666	1.308584529
ENSG00000280693	SH3PXD2A-AS1	0.362725113	0.146469722	1.308274171
ENSG00000138794	CASP6	8.120072344	3.279061762	1.308209505
ENSG00000086827	ZW10	17.57345357	7.098117024	1.307889476
ENSG00000198246	SLC29A3	3.145362432	1.270474955	1.307858329
ENSG00000136999	NOV	6.265105589	2.531091724	1.307579034
ENSG00000251898	SCARNA11	0.374237	0.151219	1.307312783
ENSG00000274509	AP000944.1	0.727089	0.293798	1.307307394
ENSG00000236549	AC079807.3	0.335579	0.135599	1.307305898
ENSG00000271826	PLS3-AS1	0.12264104	0.049556802	1.307286823
ENSG00000233193	AC005324.7	0.061321	0.024779	1.307263258
ENSG00000196812	ZSCAN16	4.173258	1.686518	1.307126398
ENSG00000129194	SOX15	0.3474555	0.140482063	1.306442284
ENSG00000202523	Y_RNA	0.708555	0.286521	1.306238961
ENSG00000230445	LRRC37A6P	0.224350791	0.090761888	1.305597749
ENSG00000101191	DIDO1	6.34993982	2.568978135	1.305548308
ENSG00000168916	ZNF608	2.146998972	0.86867688	1.305429955
ENSG00000229512	AC068580.5	0.243645	0.098596	1.305179593
ENSG00000086289	EPDR1	7.526374082	3.046121187	1.304981658
ENSG00000142677	IL22RA1	1.828975	0.74071	1.304054636
ENSG00000183722	LHFP	2.442452398	0.989380541	1.303733014
ENSG00000275445	CTD-2649C14.3	0.33698	0.136516	1.303592921
ENSG00000266184	RP11-552O4.2	0.658362	0.266749	1.303398205
ENSG00000020633	RUNX3	9.009468148	3.650474092	1.303358102
ENSG00000140365	COMMD4	2.279310305	0.923868009	1.30283869
ENSG00000276390	RP1-197B17.5	3.099057	1.256183	1.30278264

ENSG00000166130	IKBIP	7.813907262	3.167606388	1.302651058
ENSG00000224611	AC007919.18	3.013899	1.222076	1.302297063
ENSG00000131650	KREMEN2	3.643603695	1.477410327	1.302295482
ENSG00000273820	USP27X	2.435163	0.988145	1.301223681
ENSG00000188505	NCCRP1	0.067416	0.027361	1.300970071
ENSG00000237614	AC073257.2	0.252553	0.102501	1.300948194
ENSG00000198780	FAM169A	6.162043869	2.501022222	1.300891077
ENSG00000256103	RP1-96H9.5	6.459346	2.62212	1.30065439
ENSG00000160401	CFAP157	0.43897989	0.178211186	1.300566955
ENSG00000185070	FLRT2	0.003208765	0.001302807	1.300394933
ENSG00000150510	FAM124A	1.271394264	0.516248491	1.300273919
ENSG00000186814	ZSCAN30	2.724892298	1.106806781	1.299795821
ENSG00000277609	MIR6800	0.095578	0.038824	1.299729814
ENSG00000259585	RBM17P4	1.993008	0.80969	1.299505936
ENSG00000185339	TCN2	1.222990508	0.497153093	1.29865112
ENSG00000179943	FIZ1	1.127791624	0.458485361	1.298552957
ENSG00000260036	RP11-178D12.2	2.117273	0.860838	1.298393632
ENSG00000188542	DUSP28	0.821647789	0.334148717	1.298029761
ENSG00000248238	RP11-3J1.1	0.618666	0.251605	1.298000158
ENSG00000262500	RP11-259G18.2	0.782955	0.31843	1.29795313
ENSG00000232034	AC092168.2	0.122484	0.049818	1.297854294
ENSG00000250900	CTC-338M12.6	0.283095804	0.115169394	1.297532997
ENSG00000070718	AP3M2	5.238185797	2.131156966	1.297430377
ENSG00000183067	IGSF5	0.150576317	0.061268205	1.297284398
ENSG00000177383	MAGEF1	34.027039	13.847051	1.297102856
ENSG00000203666	EFCAB2	0.339754124	0.138271919	1.29698287
ENSG00000112144	ICK	6.2800852	2.556523254	1.296600983
ENSG00000160345	C9orf116	3.247331209	1.322585704	1.295893326
ENSG00000125510	OPRL1	0.929009652	0.378493718	1.295424231
ENSG00000118160	SLC8A2	0.501641056	0.204432717	1.29502933
ENSG00000196233	LCOR	6.601205524	2.690460179	1.294876562
ENSG00000244425	RN7SL268P	0.819805	0.334167	1.294711519
ENSG00000173338	KCNK7	0.254759	0.103878	1.294242971
ENSG00000279092	RP11-1H8.5	0.220086	0.089752	1.294051383
ENSG00000198373	WWP2	5.194065229	2.118391252	1.293895063
ENSG00000087903	RFX2	1.584983994	0.646584274	1.293557946
ENSG00000114988	LMAN2L	9.082145367	3.705007571	1.293556638
ENSG00000217835	RP6-159A1.2	6.764579	2.759572	1.293555622
ENSG00000116990	MYCL	2.085836173	0.851035505	1.293334623
ENSG00000049089	COL9A2	1.812234892	0.739540704	1.293068502
ENSG00000167384	ZNF180	1.626674394	0.664201018	1.292233662
ENSG00000199133	MIRLET7D	2.42363	0.989633	1.292203955
ENSG00000108852	MPP2	5.08266178	2.075409587	1.292188148
ENSG00000177225	PDDC1	6.531538788	2.667087788	1.292157609
ENSG00000267056	AC005336.4	7.161774	2.925179	1.291792083
ENSG00000161103	AC008132.13	0.157277242	0.064272238	1.291042304
ENSG00000157193	LRP8	9.532596512	3.896156359	1.290817656
ENSG00000259343	TMC3-AS1	0.518232059	0.211828134	1.290704052

ENSG00000103647	CORO2B	1.848328917	0.755900452	1.289953354
ENSG00000272398	CD24	16.0955166	6.582655702	1.289917236
ENSG00000235937	AC008280.1	0.064238	0.026272	1.289900944
ENSG00000275542	RP11-455O6.9	0.779942	0.318981	1.28989635
ENSG00000115919	KYNU	0.037593818	0.015375175	1.289892575
ENSG00000279361	RP11-360N9.3	0.165413	0.067651	1.289889457
ENSG00000171604	CXXC5	14.89574746	6.092980528	1.289680484
ENSG00000155636	RBM45	2.088519642	0.854374617	1.289540021
ENSG00000237818	RPS3AP29	0.1125	0.046025	1.289435375
ENSG00000187764	SEMA4D	2.49669343	1.021523809	1.289295852
ENSG00000265037	MIR4707	1.335634	0.5467	1.288703444
ENSG00000259673	IQCH-AS1	0.838226242	0.343269809	1.287996711
ENSG00000261408	TEN1-CDK3	1.466488688	0.600641871	1.287788989
ENSG00000172687	ZNF738	2.033241875	0.832878441	1.287603995
ENSG00000253797	UTP14C	1.106782	0.453436	1.287400244
ENSG00000279519	RP11-288C18.1	2.414168	0.989385	1.286922143
ENSG00000138771	SHROOM3	4.740967894	1.943338332	1.28664453
ENSG00000072163	LIMS2	0.722522545	0.296199659	1.286472627
ENSG00000040531	CTNS	3.874678461	1.588868973	1.286076437
ENSG00000170160	CCDC144A	0.007629238	0.003129121	1.285781772
ENSG00000167447	SMG8	9.17411081	3.763010715	1.285680936
ENSG00000106459	NRF1	4.621290997	1.895833701	1.285463518
ENSG00000230634	RP5-996D20.3	0.254481	0.1044	1.285436234
ENSG00000152457	DCLRE1C	2.0637795	0.846682371	1.285396082
ENSG00000154822	PLCL2	1.733398177	0.711370062	1.284930928
ENSG00000173264	GPR137	1.650118104	0.677275595	1.284754371
ENSG00000173093	CCDC63	0.295825676	0.121424255	1.284690642
ENSG00000221740	SNORD93	10.082951	4.139489	1.284393347
ENSG00000161021	MAML1	6.152736817	2.527637432	1.283438746
ENSG00000116774	OLFML3	0.23346198	0.095924747	1.28321267
ENSG00000215790	SLC35E2	1.777510745	0.730478798	1.282943976
ENSG00000280128	XXbac-BPG283O16.9	1.46948	0.604	1.282685269
ENSG00000264635	RP11-769O8.3	0.32155	0.132182	1.28251736
ENSG00000177683	THAP5	7.832463498	3.220019397	1.282396762
ENSG00000268520	CTD-2616J11.10	0.408941	0.168197	1.28174074
ENSG00000188599	NPIPP1	4.73267146	1.946582832	1.281711038
ENSG00000186951	PPARA	2.658933628	1.093660892	1.281682289
ENSG00000272667	RP11-395A13.2	1.218277	0.501116	1.281625689
ENSG00000273243	CTA-217C2.2	1.190089	0.489716	1.281052231
ENSG00000233184	RP11-421L21.3	1.398219791	0.575367098	1.281036533
ENSG00000176014	TUBB6	20.02376575	8.239793655	1.281033204
ENSG00000170209	ANKK1	0.151838189	0.062494372	1.280736517
ENSG00000145604	SKP2	13.73996993	5.656858361	1.280305893
ENSG00000170185	USP38	7.250104966	2.985073829	1.280235269
ENSG00000099864	PALM	8.707089744	3.585205894	1.28013462
ENSG00000144579	CTDSP1	9.860252825	4.060124052	1.280100831
ENSG00000231416	RP11-422P24.9	0.208947	0.086088	1.279252989
ENSG00000217612	RP1-28C20.1	0.104826	0.043204	1.278759799

ENSG00000232815	DUX4L50	0.183654	0.075693	1.278758526
ENSG00000262714	RP11-44F14.8	0.245214	0.101066	1.278743613
ENSG00000225649	AC064875.2	0.092278197	0.038033155	1.27873219
ENSG00000171443	ZNF524	2.108450518	0.869112306	1.278568646
ENSG00000159259	CHAF1B	11.5144112	4.747154048	1.278305867
ENSG00000280985	AC217773.1	0.239947	0.098952	1.277915003
ENSG00000207294	Y_RNA	2.275569	0.938991	1.277044097
ENSG00000185745	IFIT1	3.091064346	1.275522	1.277015904
ENSG00000225793	RP1-234P15.4	0.518009153	0.21377448	1.276887953
ENSG00000184557	SOCS3	13.597888	5.611987	1.27679902
ENSG00000250268	ALG1L14P	0.749096	0.309167	1.276764282
ENSG00000252699	SNORA21	0.414895	0.171241	1.276718105
ENSG00000260750	RP11-482M8.1	0.334819581	0.138218102	1.276437332
ENSG00000180855	ZNF443	1.23663244	0.51053763	1.27632755
ENSG00000130684	ZNF337	2.135579867	0.881793389	1.276115287
ENSG00000099364	FBXL19	9.345087175	3.862045003	1.274843146
ENSG00000187801	ZFP69B	0.3292521	0.13609546	1.274573698
ENSG00000279833	RP4-742C19.13	1.019614	0.421468	1.274528084
ENSG00000145194	ECE2	1.539437144	0.636362459	1.274482329
ENSG00000257433	RP1-197B17.3	0.147224621	0.060880821	1.273959237
ENSG00000275367	RP11-266K4.14	0.13641	0.056409	1.273952143
ENSG00000240476	LINC00973	2.72046	1.125167	1.27371147
ENSG00000131845	ZNF304	2.33571324	0.966178629	1.273501316
ENSG00000262823	RP13-580F15.2	0.493995662	0.204421311	1.272952769
ENSG00000280172	CH507-210P18.3	0.153916	0.063693	1.272936481
ENSG00000236804	RPS3AP12	0.201422	0.083373	1.272569114
ENSG00000228716	DHFR	21.63411442	8.955698226	1.272430246
ENSG00000111087	GLI1	1.179418158	0.488262246	1.272347176
ENSG00000243415	RP11-274H2.2	0.113380706	0.04694461	1.272143744
ENSG00000149679	CABLES2	7.200636	2.982049781	1.271819998
ENSG00000171227	TMEM37	2.275232517	0.942622799	1.271261507
ENSG00000141564	RPTOR	6.559295028	2.719341076	1.270283653
ENSG00000233622	CYP2T1P	0.079216	0.032851	1.269854567
ENSG00000223394	TRBV29OR9-2	0.152841	0.063385	1.269818229
ENSG00000070748	CHAT	0.130701146	0.054228724	1.269142662
ENSG00000134363	FST	0.392563539	0.162962925	1.268382401
ENSG00000006756	ARSD	1.321860207	0.548765736	1.268307302
ENSG00000150873	C2orf50	0.221801346	0.092104685	1.267921679
ENSG00000258605	DYNLL1P2	10.113578	4.200021	1.26782504
ENSG00000214694	ARHGEF33	0.037082435	0.01540321	1.267504958
ENSG00000274482	AL592188.3	0.762206	0.316624	1.267410349
ENSG00000223908	AC068657.2	3.073732	1.276962	1.267275788
ENSG00000213690	RP11-513D4.1	0.465717	0.19349	1.267194539
ENSG00000100461	RBM23	2.360861784	0.980940333	1.267076291
ENSG00000228594	C1orf233	2.969311	1.23452	1.266177997
ENSG00000169688	MT1B	2.97341	1.236227	1.266174729
ENSG00000182578	CSF1R	0.137856336	0.057315835	1.266159888
ENSG00000160051	IQCC	2.089455669	0.869094785	1.265541718

ENSG00000069011	PITX1	3.401944622	1.415384114	1.265166025
ENSG00000140543	DET1	1.785356299	0.742884643	1.26500191
ENSG00000214049	UCA1	48.85299099	20.32861022	1.264935305
ENSG00000146122	DAAM2	0.089338868	0.037180426	1.26474469
ENSG00000255063	RP11-718B12.1	0.665115	0.276845	1.264525342
ENSG00000180739	S1PR5	2.40705122	1.002174579	1.264132993
ENSG00000258759	RP11-1012A1.7	4.095773	1.70578	1.263704169
ENSG00000132677	RHBG	1.75720924	0.731926313	1.263515674
ENSG00000151422	FER	5.299580474	2.208175892	1.263023063
ENSG00000239899	RN7SL674P	2.259148	0.941369	1.262946537
ENSG00000229852	RP11-398K22.12	0.330336674	0.13765756	1.262853305
ENSG00000100403	ZC3H7B	9.043766203	3.769076605	1.262712579
ENSG00000174775	HRAS	13.05591584	5.441307824	1.262678311
ENSG00000180611	MB21D2	2.715572	1.132224	1.262096705
ENSG00000234311	RP11-432J24.3	0.256172	0.106852	1.261498884
ENSG00000263272	CTC-524C5.2	0.782857	0.326542	1.261480239
ENSG00000187605	TET3	2.574134384	1.073766428	1.261407169
ENSG00000174721	FGFBP3	0.560758	0.23392	1.261363081
ENSG00000228507	DAP3P2	1.677579	0.700384	1.260162675
ENSG00000235072	AC012074.2	0.710831404	0.296780321	1.26011199
ENSG00000114631	PODXL2	0.4149	0.173305	1.259450379
ENSG00000240219	RP11-430C7.5	0.408729	0.170728	1.259444924
ENSG00000114648	KLHL18	4.204200333	1.756284472	1.259304874
ENSG00000110925	CSRNP2	9.265575564	3.871563345	1.25896435
ENSG00000100599	RIN3	1.86177888	0.778093019	1.258667196
ENSG00000273055	CTB-13F3.1	3.333395	1.393754	1.258016338
ENSG00000266920	ACTBP9	1.340251	0.560411	1.257946033
ENSG00000128973	CLN6	3.951219228	1.65263728	1.257527777
ENSG00000204414	CSHL1	0.028010754	0.01171603	1.257496993
ENSG00000223764	RP11-54O7.3	0.401335	0.167888	1.257307862
ENSG00000126778	SIX1	1.512076097	0.632707008	1.256921267
ENSG00000164306	PRIMPOL	3.310129123	1.385541496	1.256437576
ENSG00000173327	MAP3K11	4.281040861	1.792179262	1.256246655
ENSG00000276043	UHRF1	17.02193219	7.126670715	1.256094638
ENSG00000167419	LPO	0.039967887	0.016735665	1.255915424
ENSG00000125850	OVOL2	0.222462382	0.093165954	1.25568665
ENSG00000106477	CEP41	1.206076148	0.505139021	1.255568602
ENSG00000255262	TCEB2P2	0.662367	0.277463	1.255335398
ENSG00000070601	FRMPD1	0.572288988	0.239838702	1.254679365
ENSG00000228451	SDAD1P1	0.873927	0.36626	1.254644625
ENSG00000256128	LINC00944	0.416659067	0.174658051	1.254334233
ENSG00000174243	DDX23	15.85333352	6.646588107	1.254100374
ENSG00000156042	CFAP70	0.450491287	0.18889838	1.25388887
ENSG00000280325	AC074183.3	3.693563	1.549068	1.253612709
ENSG00000275183	LENG9	1.122054	0.470589	1.253602606
ENSG00000170604	IRF2BP1	7.488729	3.140848	1.253566757
ENSG00000173175	ADCY5	0.244471502	0.10259265	1.252738919
ENSG00000260912	RP11-363E7.4	2.40572	1.009699	1.252543461

ENSG00000259768	RP5-991G20.1	1.53488	0.644415	1.252063886
ENSG00000244230	RN7SL151P	0.455368	0.191238	1.25166369
ENSG00000059378	PARP12	3.357109348	1.410054185	1.251468926
ENSG00000161999	JMJD8	31.48611851	13.22608292	1.251330067
ENSG00000197044	ZNF441	0.568133503	0.238686094	1.251115468
ENSG00000236811	GAPDHP2	0.615038	0.258492	1.250555918
ENSG00000149483	TMEM138	2.691969073	1.131484453	1.250445074
ENSG00000101057	MYBL2	35.99235335	15.13079155	1.250202973
ENSG00000132938	MTUS2	0.137169501	0.05767686	1.249895224
ENSG00000250273	PSMC1P5	15.038342	6.323745	1.249794418
ENSG00000129038	LOXL1	3.156849187	1.327483078	1.24979187
ENSG00000135537	LACE1	0.394485036	0.165888114	1.249760056
ENSG00000164438	TLX3	2.515913	1.058026	1.249706954
ENSG00000237799	CICP11	0.036418	0.015315	1.249706328
ENSG00000228049	POLR2J2	3.768086698	1.585183	1.249182759
ENSG00000196172	ZNF681	0.029833	0.012551	1.249106747
ENSG00000257069	KCNK4-TEX40	0.094106	0.039605	1.248604132
ENSG00000249626	RP11-496H1.1	1.747757	0.735982	1.247762226
ENSG00000128602	SMO	5.062521246	2.132138104	1.247555169
ENSG00000213730	POLD2P1	1.54118	0.649229	1.24723602
ENSG00000197332	CTC-499B15.5	0.921373	0.388162	1.247126433
ENSG00000152784	PRDM8	0.722397986	0.30433859	1.247116592
ENSG00000131115	ZNF227	2.216906098	0.93447401	1.246321218
ENSG00000167874	TMEM88	0.730578	0.308023	1.24600023
ENSG00000162104	ADCY9	3.390586258	1.429713439	1.245808735
ENSG00000112118	MCM3	48.14353494	20.30525339	1.245489045
ENSG00000060762	MPC1	8.491316924	3.58207394	1.245193203
ENSG00000037965	HOXC8	0.597824	0.252237	1.2449409
ENSG00000171388	APLN	0.397007	0.167512	1.244899996
ENSG00000232160	RAP2C-AS1	0.901757182	0.380496812	1.244854642
ENSG00000258118	RP11-77I22.4	0.165069	0.069653	1.24481181
ENSG00000108592	FTSJ3	20.5883539	8.688359594	1.244672767
ENSG00000226261	AC064836.3	1.249271	0.527368	1.244204532
ENSG00000235969	CHEK2P4	0.507937	0.214471	1.243866984
ENSG00000215022	RP1-257A7.4	0.470422011	0.198633776	1.243844605
ENSG00000267261	CTD-2132N18.3	13.64299123	5.761077638	1.243749385
ENSG00000089091	DZANK1	0.947083572	0.399949652	1.243673341
ENSG00000271853	RP1-178F15.5	0.506863598	0.214103077	1.243292024
ENSG00000052723	SIKE1	7.371754021	3.114989196	1.242780773
ENSG00000007923	DNAJC11	15.5687308	6.57883264	1.24274782
ENSG00000234805	AC090505.5	0.349113	0.147548	1.242509715
ENSG00000157933	SKI	15.70926237	6.640303925	1.24229426
ENSG00000119703	ZC2HC1C	0.139503795	0.058978401	1.242045744
ENSG00000159905	ZNF221	0.937090694	0.396393014	1.241257144
ENSG00000231969	AC144449.1	0.117672	0.049778	1.241190901
ENSG00000110104	CCDC86	29.14237349	12.32862049	1.241107004
ENSG00000236502	SIX3-AS1	0.524574839	0.221937019	1.240998283
ENSG00000267249	RP11-973H7.3	1.420852	0.60134	1.240503455

ENSG00000173218	VANGL1	6.473973942	2.740128994	1.240407747
ENSG00000271079	CTAGE15	1.738157	0.735782	1.240208112
ENSG00000166863	TAC3	0.160814296	0.068079796	1.240097044
ENSG00000111237	VPS29	16.81323606	7.119187601	1.239812904
ENSG00000006638	TBXA2R	1.356867792	0.57454903	1.23977824
ENSG00000235027	AC068580.6	0.778727	0.329755	1.239723113
ENSG00000165804	ZNF219	1.465085197	0.620545813	1.239374934
ENSG00000244045	TMEM199	4.005117512	1.696574085	1.239220143
ENSG00000049769	PPP1R3F	0.271251512	0.114904826	1.239191782
ENSG00000167178	ISLR2	0.020522062	0.008693774	1.239121289
ENSG00000147649	MTDH	39.74037314	16.83528173	1.239117555
ENSG00000270175	RP11-793H13.11	0.989028	0.418998	1.239068008
ENSG00000093072	CECR1	0.044051646	0.018662351	1.239065185
ENSG00000239626	RPSAP41	0.297091	0.125862	1.239062128
ENSG00000245017	RP11-181C3.1	0.133210727	0.056435128	1.239044921
ENSG00000171962	DRC3	0.749814778	0.317665959	1.239023759
ENSG00000271321	CTAGE6	1.474787	0.624888	1.238837064
ENSG00000110921	MVK	2.623875008	1.112142038	1.238357942
ENSG00000233980	FDPSP2	0.140873	0.059732	1.237819195
ENSG00000147459	DOCK5	11.04046459	4.681649217	1.237712137
ENSG00000137507	LRRC32	0.54362217	0.230609655	1.237151378
ENSG00000178803	ADORA2A-AS1	0.252900107	0.107300335	1.236913074
ENSG00000170647	TMEM133	2.050987	0.870279	1.236768458
ENSG00000198727	MT-CYB	4911.836426	2085.072754	1.236164793
ENSG00000167566	NCKAP5L	3.515811892	1.492686351	1.235946831
ENSG00000230799	AC007279.2	2.659657	1.129504	1.235550822
ENSG00000112146	FBXO9	4.25069416	1.805503008	1.235297636
ENSG00000268940	CT45A1	0.2606686	0.110736997	1.235079503
ENSG00000135637	CCDC142	0.950245725	0.403728937	1.234913634
ENSG00000260240	APOOP5	0.729758914	0.310147647	1.23446475
ENSG00000109618	SEPSECS	2.191849979	0.931652272	1.234285564
ENSG00000211526	AL121869.1	1.844691	0.784224	1.234041475
ENSG00000280670	CCDC163P	1.704735566	0.72474803	1.233996557
ENSG00000254900	RP11-152H18.4	0.711031	0.30235	1.233692882
ENSG00000177613	CSTF2T	12.01076	5.107766	1.233563105
ENSG00000073282	TP63	0.034140472	0.01452062	1.23337999
ENSG00000250423	KIAA1210	0.032526	0.013834	1.233375051
ENSG00000273892	MIR6886	0.041719	0.017744	1.233373306
ENSG00000184611	KCNH7	0.030697831	0.013056749	1.233340994
ENSG00000136014	USP44	0.047248755	0.020096558	1.233327867
ENSG00000164746	C7orf57	0.111860238	0.047578516	1.233315109
ENSG00000228352	RP11-537H15.3	0.400758	0.170458	1.23331501
ENSG00000249849	RP11-1101H11.1	0.295221	0.125569	1.233315007
ENSG00000267686	RP11-795H16.3	0.668515	0.284346	1.233312422
ENSG00000232908	HSD17B7P1	0.125114	0.053216	1.233311254
ENSG00000251703	RNU6-998P	0.503923	0.214339	1.233308926
ENSG00000266126	RP11-209D14.4	0.695303	0.295741	1.233307549
ENSG00000171403	KRT9	0.111127	0.047267	1.233304178

ENSG00000170989	S1PR1	0.082865295	0.035246168	1.233301602
ENSG00000278376	RP11-158I9.8	0.135074	0.057453	1.233295869
ENSG00000254855	RP11-867G23.1	0.106255	0.045195	1.233295652
ENSG00000232926	AC000078.5	0.171644	0.073008	1.233292963
ENSG00000116701	NCF2	0.086912417	0.036967899	1.233289263
ENSG00000134874	DZIP1	0.030045089	0.012779629	1.233283286
ENSG00000280789	PAGR1	9.564721	4.068759	1.233134057
ENSG00000179627	ZBTB42	1.808803023	0.769467401	1.233103196
ENSG00000236680	RP11-296P7.4	3.009235	1.28059	1.232588126
ENSG00000128581	IFT22	4.448167047	1.89370648	1.231998234
ENSG00000146828	SLC12A9	2.917251662	1.242033729	1.231905496
ENSG00000273137	RP3-402G11.28	1.469534	0.625685	1.231850316
ENSG00000141298	SSH2	2.181861996	0.929077173	1.231689509
ENSG00000228432	DHFRP2	0.290304	0.123676	1.230998886
ENSG00000167525	PROCA1	0.154674369	0.065899271	1.230899736
ENSG00000163848	ZNF148	6.551621925	2.79136055	1.230883621
ENSG00000279148	RP11-135F9.4	0.392841	0.167426	1.230421924
ENSG00000108312	UBTF	14.00195279	5.96806763	1.230292258
ENSG00000165916	PSMC3	69.22113618	29.5137317	1.229826276
ENSG00000187860	CCDC157	0.237924979	0.101447409	1.229774727
ENSG00000100364	KIAA0930	7.079533169	3.019198214	1.229488756
ENSG00000273247	RP11-83A24.2	0.357694385	0.152567276	1.229281916
ENSG00000103196	CRISPLD2	3.738395608	1.595063644	1.228805258
ENSG00000269486	CTC-360G5.9	0.430924	0.183863	1.228802265
ENSG00000090924	PLEKHG2	3.147942033	1.343188909	1.228746752
ENSG00000161217	PCYT1A	4.195110488	1.790336751	1.228477833
ENSG00000280057	RP1-168L15.6	0.141996	0.060639	1.227532423
ENSG00000203462	RP11-814E24.1	0.372739	0.159178	1.227524823
ENSG00000277496	RP11-93B14.9	0.483895	0.206658	1.227448819
ENSG00000196659	TTC30B	0.283813	0.121256	1.226884537
ENSG00000171792	RHNO1	9.499404937	4.058937637	1.226734969
ENSG00000127824	TUBA4A	10.501549	4.487549003	1.226602546
ENSG00000188869	TMC3	0.081489364	0.034830137	1.226275639
ENSG00000124164	VAPB	8.193191388	3.502864035	1.225890525
ENSG00000186908	ZDHHC17	6.060528109	2.591236093	1.225803045
ENSG00000185115	NSMCE3	13.989947	5.983845	1.225245786
ENSG00000076554	TPD52	15.89902768	6.802052398	1.224896515
ENSG00000197381	ADARB1	2.066659465	0.884217088	1.224828167
ENSG00000127507	ADGRE2	2.977682553	1.274136084	1.224670585
ENSG00000103485	QPRT	0.138909538	0.059440396	1.224630024
ENSG00000261458	RP11-787D11.1	0.790482	0.338253	1.224629882
ENSG00000235651	AC064850.4	1.221072	0.522624	1.224302988
ENSG00000258401	RP11-326E7.1	2.395653	1.025441	1.224174468
ENSG00000188761	BCL2L15	0.357506101	0.153029338	1.224159595
ENSG00000272486	RP11-532M24.1	1.154914	0.494421	1.2239735
ENSG00000276987	MIR6848	0.632853	0.270987	1.223646784
ENSG00000274183	H2AFB1	0.101156	0.043333	1.22304387
ENSG00000277745	H2AFB3	0.100472	0.04304	1.223043518

ENSG00000089280	FUS	29.11349854	12.47433841	1.222724914
ENSG00000226008	SEPT14P3	0.387207	0.165943	1.222417261
ENSG00000269721	RPL23AP51	0.18681	0.080066	1.222310047
ENSG00000070404	FSTL3	4.696816681	2.013050136	1.222300182
ENSG00000145476	CYP4V2	0.970877728	0.416129242	1.222257941
ENSG00000173727	CMB9-22P13.1	4.05400587	1.738029659	1.221895476
ENSG00000182256	GABRG3	0.010021635	0.004296941	1.221736166
ENSG00000229742	RP11-365O16.5	0.266292	0.114178	1.221724391
ENSG00000162078	ZG16B	0.094725936	0.040615952	1.221713034
ENSG00000148719	DNAJB12	3.426344366	1.469492873	1.221351795
ENSG00000113621	TXNDC15	3.46798701	1.488240181	1.220491119
ENSG00000162517	PEF1	10.68831807	4.587237478	1.220337344
ENSG00000227329	RP11-258C19.4	3.352444	1.438893	1.22025392
ENSG00000106278	PTPRZ1	0.049576652	0.021283687	1.219912751
ENSG00000258841	EEF1A1P2	0.16725	0.071811	1.219729453
ENSG00000123364	HOXC13	2.585351	1.110107	1.219661421
ENSG00000242732	RGAG4	2.297730357	0.986716999	1.219501236
ENSG00000091317	CMTM6	78.86742802	33.87157904	1.219354351
ENSG00000131355	ADGRE3	0.107461553	0.046166252	1.218910069
ENSG00000172987	HPSE2	0.226793229	0.097443672	1.218737168
ENSG00000254973	RP11-429J17.7	0.628079	0.26987	1.218681424
ENSG00000255120	OVOL1-AS1	0.364075349	0.156435942	1.218665047
ENSG00000232110	RP11-149I23.3	0.248797681	0.106957663	1.217933189
ENSG00000077984	CST7	0.303197	0.130355	1.217809558
ENSG00000107742	SPOCK2	0.249031387	0.107104617	1.217306911
ENSG00000261167	RP11-517B11.7	2.013866	0.866428	1.21681592
ENSG00000130382	MLLT1	17.35981046	7.469319088	1.21670256
ENSG00000164683	HEY1	0.406602754	0.1749871	1.216371418
ENSG00000114859	CLCN2	5.540920078	2.38474083	1.216293073
ENSG00000152292	SH2D6	0.087992981	0.037876008	1.216104164
ENSG00000126856	PRDM7	0.158969095	0.068427346	1.216101431
ENSG00000136286	MYO1G	0.029056401	0.012508414	1.215957135
ENSG00000276075	CTD-2012K14.8	0.28474235	0.122583058	1.215897485
ENSG00000176402	GJC3	0.225293	0.09699	1.215894575
ENSG00000258708	SLC25A21-AS1	0.392038	0.168775	1.215892282
ENSG00000004939	SLC4A1	0.047555816	0.02047323	1.215883093
ENSG00000122694	GLIPR2	8.875407804	3.821775352	1.215570432
ENSG00000237412	PRSS56	0.085182137	0.036684194	1.215392335
ENSG00000132746	ALDH3B2	0.06293156	0.027102504	1.215357535
ENSG00000151208	DLG5	11.51654179	4.959829475	1.215345142
ENSG00000147689	FAM83A	0.234326149	0.100924072	1.215247632
ENSG00000228643	AC079779.4	0.202039028	0.087024197	1.215145499
ENSG00000196081	ZNF724P	1.955406043	0.842327345	1.215015309
ENSG00000186326	RGS9BP	0.347163	0.149608	1.214425874
ENSG00000160131	VMA21	15.71415207	6.773700634	1.214048294
ENSG00000154309	DISP1	2.287013046	0.985901451	1.213949246
ENSG00000249709	ZNF564	1.766612645	0.761751618	1.213593179
ENSG00000198040	ZNF84	0.750937627	0.323810583	1.213542944

ENSG00000113430	IRX4	1.63386077	0.704560353	1.213489851
ENSG00000204438	GPANK1	4.932768356	2.127171881	1.213460928
ENSG00000162144	CYB561A3	1.678837783	0.724048637	1.213304321
ENSG00000272419	RP11-403I13.8	0.380356377	0.1640875	1.212886457
ENSG00000036448	MYOM2	0.261512271	0.11285783	1.212372124
ENSG00000137133	HINT2	4.915249527	2.121824404	1.21195939
ENSG00000005339	CREBBP	6.924065676	2.989047681	1.211933498
ENSG00000245532	NEAT1	7.080963434	3.057317612	1.211679231
ENSG00000105784	RUNDC3B	2.346880444	1.013489009	1.211413906
ENSG00000146858	ZC3HAV1L	3.70661	1.600747	1.21135502
ENSG00000240710	RP11-430C7.4	0.143075	0.061807	1.210929461
ENSG00000207765	AL132780.1	1.922746	0.830769	1.210648903
ENSG00000213644	SAPCD2P1	0.84347	0.364461	1.210572313
ENSG00000184828	ZBTB7C	0.30918394	0.133603587	1.210506641
ENSG00000276853	RP11-305O6.4	0.813098	0.351453	1.210097476
ENSG00000233370	AC092664.1	0.111194	0.048064	1.21005032
ENSG00000172568	FNDC9	0.109588621	0.047370295	1.210043438
ENSG00000230614	DYNLL1P7	4.974217	2.150137	1.21004086
ENSG00000236829	Z97634.3	0.156842734	0.067798326	1.209997134
ENSG00000133454	MYO18B	0.063974532	0.027656673	1.209870083
ENSG00000279379	RP11-84N19.1	0.80224	0.346818	1.209855124
ENSG00000101986	ABCD1	3.597522948	1.555282625	1.20982712
ENSG00000277582	RN7SL428P	0.053807	0.023263	1.209756713
ENSG00000213519	AC132008.1	0.533877	0.230864	1.209464175
ENSG00000280916	FOXCUT	0.375795	0.162524	1.209293094
ENSG00000276290	RBBP4P3	0.289985	0.125423	1.209176343
ENSG00000267198	RP11-798G7.6	0.392277014	0.169692814	1.208947332
ENSG00000266411	RP11-180P8.3	0.27436	0.118685	1.208932549
ENSG00000136243	NUPL2	7.474507174	3.234075156	1.208625254
ENSG00000241357	RP11-758P17.2	0.822658	0.356021	1.208330449
ENSG00000198944	SOWAHA	0.388657	0.16821	1.208234025
ENSG00000239322	ATP6V1B1-AS1	0.603546153	0.261336241	1.207556889
ENSG00000235426	RP11-342M3.5	0.24456	0.105911	1.207336021
ENSG00000183853	KIRREL	4.350389401	1.884162021	1.207221513
ENSG00000188985	DHFRP1	18.5193	8.022555	1.206895888
ENSG00000260566	RP11-20G6.3	3.014791	1.306479	1.206374056
ENSG00000250682	LINC00491	1.528960373	0.662812636	1.205878005
ENSG00000165338	HECTD2	0.982115184	0.425787564	1.205758421
ENSG00000271127	LL22NC03-N64E9.1	0.562987	0.244088	1.205700239
ENSG00000274290	HIST1H2BE	0.196600284	0.085257221	1.205371471
ENSG00000151773	CCDC122	0.405363368	0.175793282	1.205335787
ENSG00000140025	EFCAB11	1.040177058	0.451330532	1.204572841
ENSG00000126264	HCST	0.406838	0.176576	1.204165171
ENSG00000279425	CTD-2017D11.2	0.113929	0.049448	1.204150949
ENSG00000165182	CXorf58	0.129553646	0.056229644	1.204146806
ENSG00000196814	MVB12B	1.550320593	0.673075081	1.203727233
ENSG00000225963	AC009950.2	0.405696017	0.176198412	1.203198217
ENSG00000126259	KIRREL2	0.390375215	0.169558529	1.203078107

ENSG00000047230	CTPS2	2.656694512	1.154014695	1.20297075
ENSG00000166961	MS4A15	1.609665649	0.699252077	1.202876511
ENSG00000119431	HDHD3	4.483165233	1.947582878	1.202832951
ENSG00000121858	TNFSF10	0.102583587	0.044568101	1.202716523
ENSG00000227304	RP11-791G16.2	0.65520761	0.284693286	1.202543642
ENSG00000126460	PRRG2	2.062777286	0.896360622	1.202436888
ENSG00000120500	ARR3	0.436094087	0.189529198	1.202219305
ENSG00000134940	ACRV1	0.168887	0.0734	1.202206313
ENSG00000158292	GPR153	4.529394	1.968629	1.202126789
ENSG00000267080	ASB16-AS1	3.300373116	1.434664472	1.201915764
ENSG00000151116	UEVLD	3.678122876	1.599235568	1.201587213
ENSG00000233614	DDX11L10	0.237180411	0.103132548	1.201485152
ENSG00000123609	NMI	1.593139224	0.692836298	1.201285928
ENSG00000103202	NME4	6.897898757	3.000326496	1.201037451
ENSG00000107882	SUFU	8.095270998	3.521311487	1.200966527
ENSG00000137486	ARRB1	4.274433712	1.86039235	1.200126388
ENSG00000235876	FEM1AP4	0.333322	0.145093	1.199938623
ENSG00000240207	RP11-379F4.4	0.19567019	0.085180252	1.199833081
ENSG00000244921	MTCYBP18	40.887302	17.800367	1.199745882
ENSG00000135447	PPP1R1A	1.416653029	0.616773	1.199674936
ENSG00000138496	PARP9	2.507310001	1.09163493	1.199649916
ENSG00000175745	NR2F1	44.54488106	19.39722941	1.199409051
ENSG00000122986	HVCN1	0.301533002	0.131331769	1.199099964
ENSG00000120784	ZFP30	0.525938182	0.229125416	1.198755734
ENSG00000239696	RP11-397J20.1	0.344724	0.15023	1.198268805
ENSG00000185838	GNB1L	0.747666674	0.32594577	1.197763276
ENSG00000255874	LINC00346	1.204774	0.525224	1.197757794
ENSG00000257964	RP11-133N21.10	0.357166	0.155716	1.197677561
ENSG00000188937	NYX	0.017033427	0.007426202	1.197672236
ENSG00000101197	BIRC7	0.065167421	0.028411836	1.197658839
ENSG00000279840	RP1-228P16.9	0.034239	0.014928	1.197619675
ENSG00000029153	ARNTL2	2.666792746	1.163057336	1.197183488
ENSG00000164045	CDC25A	7.360010893	3.210160055	1.197062671
ENSG00000170345	FOS	17.92995765	7.820469547	1.197044945
ENSG00000075218	GTSE1	11.39388042	4.969886512	1.196974358
ENSG00000128011	LRFN1	3.686852	1.609339	1.195921249
ENSG00000157322	CLEC18A	0.101366023	0.044248427	1.195876087
ENSG00000247572	CKMT2-AS1	1.738854296	0.759136709	1.195705428
ENSG00000115705	TPO	0.036603972	0.015980558	1.195682391
ENSG00000226338	AC079150.2	4.750313	2.074621	1.195174773
ENSG00000205485	AC004980.7	1.893702208	0.827083394	1.195104772
ENSG00000177575	CD163	0.006181271	0.00269997	1.194959907
ENSG00000248472	DDX11L9	0.287174719	0.125451361	1.194800628
ENSG00000176595	KBTBD11	3.408908	1.489242	1.194731455
ENSG00000163959	SLC51A	0.318424007	0.139112636	1.194695639
ENSG00000245498	RP11-677M14.7	0.073397849	0.032067736	1.194615272
ENSG00000164694	FNDC1	0.366684487	0.16021332	1.194545133
ENSG00000272142	RP11-428J1.5	1.317685138	0.575735351	1.194527974

ENSG00000281357	ARRDC3-AS1	0.393582643	0.171993291	1.194314308
ENSG00000179240	RP11-111M22.2	0.272991465	0.119309619	1.194145482
ENSG00000169710	FASN	68.04177739	29.742884	1.193876284
ENSG00000143409	FAM63A	0.301932409	0.131985651	1.193844526
ENSG00000164651	SP8	0.395425997	0.172930211	1.193217793
ENSG00000272953	RP11-1275H24.2	0.1958	0.085634	1.193125144
ENSG00000264207	RP11-196G18.23	0.493289	0.215781	1.192865279
ENSG00000116833	NR5A2	0.537712595	0.235269437	1.192521348
ENSG00000123388	HOXC11	0.075760071	0.033151336	1.192370678
ENSG00000185798	WDR53	6.823350755	2.986691009	1.19193239
ENSG00000153896	ZNF599	0.206568414	0.090440702	1.191575576
ENSG00000132109	TRIM21	10.55343512	4.620726545	1.191521051
ENSG00000223821	RP11-505P4.6	0.04677	0.020478	1.191508608
ENSG00000074370	ATP2A3	6.4681637	2.83205948	1.191504626
ENSG00000196757	ZNF700	4.777506683	2.092103708	1.191303522
ENSG00000260874	RP11-715J22.4	0.313061556	0.137108273	1.191130732
ENSG00000065328	MCM10	12.47233971	5.464012305	1.190699491
ENSG00000105705	SUGP1	7.311893708	3.204530758	1.190131981
ENSG00000267801	RP11-552F3.9	1.267419	0.555464	1.190128231
ENSG00000274527	RP11-146B14.1	0.796733	0.349257	1.1898073
ENSG00000175985	PLEKHD1	0.714130624	0.313079386	1.189659468
ENSG00000170579	DLGAP1	0.069133112	0.030310633	1.189552884
ENSG00000145248	SLC10A4	3.433691	1.505699	1.189326822
ENSG00000173933	RBM4	13.42419525	5.886994385	1.189232448
ENSG00000265474	AC010761.9	0.264838	0.116158	1.189021622
ENSG00000276529	AP001505.10	3.269766	1.434255	1.188885846
ENSG00000025434	NR1H3	2.410550052	1.057547304	1.188640189
ENSG00000176953	NFATC2IP	7.345040185	3.22293599	1.188394848
ENSG00000276007	RP11-358L22.3	1.920982	0.843089	1.188087159
ENSG00000166831	RBPMS2	12.61479897	5.536471745	1.188078435
ENSG00000179918	SEPHS2	14.573102	6.396501	1.187953153
ENSG00000164631	ZNF12	5.806517616	2.548802015	1.187853873
ENSG00000260053	ABCB10P4	0.170664	0.07492	1.187735963
ENSG00000108557	RAI1	4.850617887	2.129866462	1.187405554
ENSG00000231908	IDH1-AS1	0.383859362	0.168558673	1.18732697
ENSG00000125503	PPP1R12C	6.758668069	2.967915287	1.187289049
ENSG00000114923	SLC4A3	3.899740065	1.712771317	1.187045424
ENSG00000085644	ZNF213	1.756527883	0.771516096	1.186958317
ENSG00000108417	KRT37	0.083504	0.036692	1.186379762
ENSG00000250727	RP11-959I15.1	0.084594	0.037171	1.18637784
ENSG00000267147	CTC-548K16.1	0.961707	0.422817	1.185564036
ENSG00000163781	TOPBP1	24.23516667	10.66103162	1.184754956
ENSG00000164764	SBSPON	0.300321493	0.132120956	1.184648414
ENSG00000250090	RP13-401N8.3	0.498726	0.219408	1.184631287
ENSG00000106031	HOXA13	2.513587723	1.105820676	1.184630587
ENSG00000213965	NUDT19	6.689413	2.943523	1.184335718
ENSG00000163633	C4orf36	0.320241229	0.140915351	1.184330271
ENSG00000127124	HIVEP3	3.391724519	1.492977338	1.18382673

ENSG00000279400	CTD-2353F22.2	2.895771	1.275043	1.183401616
ENSG00000199961	SNORD1B	1.066397	0.469589	1.183274108
ENSG00000188994	ZNF292	6.221260854	2.73962381	1.183229195
ENSG00000204178	TMEM57	14.0670023	6.194686771	1.18321168
ENSG00000225371	CICP8	0.03058	0.013467	1.183159905
ENSG00000109113	RAB34	0.295360174	0.130096989	1.182887732
ENSG00000107282	APBA1	0.323064246	0.142337198	1.182508354
ENSG00000226051	ZNF503-AS1	0.675881675	0.29783211	1.182271395
ENSG00000177628	GBA	6.652644803	2.932017599	1.182034245
ENSG00000234420	ZNF37BP	1.231804593	0.542919228	1.181963927
ENSG00000083838	ZNF446	0.759900762	0.334972104	1.181770067
ENSG00000270503	YTHDF2P1	7.37174	3.249787	1.181660025
ENSG00000119401	TRIM32	1.54173844	0.67981282	1.181348555
ENSG00000237424	FOXD2-AS1	1.644339	0.725202	1.181052949
ENSG00000196371	FUT4	2.524902	1.113853	1.180668547
ENSG00000147536	GINS4	7.121258717	3.141709389	1.180582531
ENSG00000259417	LINC01314	0.035605	0.01571	1.180396672
ENSG00000218891	ZNF579	4.623723654	2.040167104	1.180367851
ENSG00000261924	CTD-2561B21.5	0.084548895	0.037307052	1.180337537
ENSG00000141098	GFOD2	1.880967568	0.83029914	1.179771865
ENSG00000143315	PIGM	2.485065	1.097198	1.17945969
ENSG00000110944	IL23A	0.800157534	0.353286723	1.179444533
ENSG00000099866	MADCAM1	0.129434282	0.057162524	1.179078264
ENSG00000259940	CTD-3203P2.1	0.508675	0.2247	1.178744067
ENSG00000156853	ZNF689	2.228590381	0.985074136	1.177827261
ENSG00000236397	DDX11L2	0.979044	0.43281	1.177639866
ENSG00000233396	RP11-458D21.1	0.405959072	0.179532266	1.177091134
ENSG00000259813	RP5-1142A6.5	1.366322	0.604289	1.176986937
ENSG00000187870	RNFT1P3	0.730173	0.322962	1.176873896
ENSG00000228106	RP11-452F19.3	4.452449573	1.969979251	1.176418838
ENSG00000188636	LDOC1L	8.06559	3.569099	1.176220151
ENSG00000257043	RP11-137N23.1	0.857789	0.379603	1.176131421
ENSG00000015171	ZMYND11	8.193265997	3.626283915	1.17594677
ENSG00000261061	RP11-303E16.2	15.160814	6.712157	1.175498849
ENSG00000021488	SLC7A9	0.114844357	0.050848995	1.175388809
ENSG00000129993	CBFA2T3	3.736509358	1.654716319	1.17510723
ENSG00000183873	SCN5A	3.255780375	1.442362169	1.17456992
ENSG00000168453	HR	3.423757591	1.516843786	1.174508045
ENSG00000250462	LRRC37BP1	4.484970807	1.987003303	1.174504324
ENSG00000203872	C6orf163	0.423563084	0.187657991	1.17447113
ENSG00000271344	RP11-274B21.9	0.09641	0.042715	1.174440013
ENSG00000232027	RP11-275F13.3	0.426355	0.188902	1.174417199
ENSG00000113721	PDGFRB	0.085575568	0.037915477	1.174412077
ENSG00000175899	A2M	0.937015567	0.415163894	1.174392035
ENSG00000139880	CDH24	6.677179739	2.958803673	1.174224904
ENSG00000051128	HOMER3	8.31857536	3.686576818	1.174054654
ENSG00000248424	OR51K1P	2.429089	1.076714	1.173780262
ENSG00000281538	RP4-669P10.20	0.785689	0.348283	1.173698307

ENSG00000170412	GPRC5C	2.250054319	0.99749727	1.173575033
ENSG00000213918	DNASE1	0.912011467	0.404533694	1.172792093
ENSG00000085491	SLC25A24	8.392127748	3.723084325	1.172538347
ENSG00000259877	RP11-46C24.7	0.665838	0.295525	1.171891028
ENSG00000249004	PRMT5P1	2.432464	1.079696	1.17179329
ENSG00000232412	RP1-315G1.3	0.747964	0.332027	1.17166827
ENSG00000238145	RP11-346M5.1	0.177966	0.079002	1.171640562
ENSG00000240210	RP11-204K16.1	0.258717	0.114862	1.171473267
ENSG00000099849	RASSF7	4.579162335	2.033711388	1.170968755
ENSG00000185215	TNFAIP2	9.095581505	4.039760298	1.170896184
ENSG00000104142	VPS18	8.307157704	3.690060857	1.170710333
ENSG00000163900	TMEM41A	3.493115357	1.552053828	1.170335694
ENSG00000073536	NLE1	4.138202999	1.838716692	1.170305211
ENSG00000216105	MIR943	0.017831	0.007923	1.170268907
ENSG00000276057	TUG1_2	2.387708	1.06126	1.169848268
ENSG00000198468	FLVCR1-AS1	2.371262176	1.054073747	1.169679374
ENSG00000279078	SND1-IT1	0.245352	0.10907	1.169598693
ENSG00000259726	CSPG4P11	0.368547	0.16392	1.168856725
ENSG00000072954	TMEM38A	6.029353328	2.681999729	1.168694184
ENSG00000169247	SH3TC2	0.587327604	0.261302461	1.168444731
ENSG00000114491	UMPS	6.51050951	2.896550443	1.168434663
ENSG00000013297	CLDN11	0.024975443	0.011111942	1.168399233
ENSG00000214870	AC004540.5	0.142939749	0.063596834	1.168380297
ENSG00000177556	ATOX1	7.605393544	3.385384233	1.167703317
ENSG00000166888	STAT6	5.463035612	2.431762202	1.167700671
ENSG00000274225	KB-68A7.1	0.943635	0.420191	1.167183669
ENSG00000249930	AC007016.3	0.488103	0.21737	1.167032776
ENSG00000043093	DCUN1D1	5.132925644	2.286605413	1.166573933
ENSG00000179921	GPBAR1	0.045092055	0.020104247	1.165372956
ENSG00000124766	SOX4	93.884201	41.861233	1.165267593
ENSG00000155893	PXYLP1	5.139785418	2.291898835	1.165164764
ENSG00000221667	MIR1825	1.125264	0.501775	1.165151016
ENSG00000280433	CH507-9B2.9	2.75737432	1.229740019	1.164941784
ENSG00000103150	MLYCD	0.530820007	0.236760025	1.16479723
ENSG00000275264	MIR1204	0.470022	0.209655	1.164711049
ENSG00000172315	TP53RK	6.00893326	2.680811612	1.164439057
ENSG00000104524	PYCRL	3.121394039	1.392904752	1.164093884
ENSG00000197020	ZNF100	1.830570616	0.816933028	1.164003713
ENSG00000107249	GLIS3	1.954471011	0.872633339	1.163330689
ENSG00000246863	RP11-325N19.3	0.021036567	0.009394325	1.163037885
ENSG00000232724	TRIM80P	0.035741	0.015962	1.162938571
ENSG00000221269	MIR1302-8	0.397627	0.177582	1.162930368
ENSG00000212296	SNORD72	0.636203	0.284133	1.162920775
ENSG00000237032	RPSAP7	0.177993	0.079499	1.162811887
ENSG00000132026	RTBDN	5.338381534	2.38493387	1.162453155
ENSG00000118655	DCLRE1B	6.902280015	3.083783525	1.162371508
ENSG00000233478	RP1-187B23.1	0.108932	0.04867	1.162323144
ENSG00000077713	SLC25A43	4.766484044	2.131131628	1.161305764

ENSG00000158792	SPATA2L	8.902735115	3.980614297	1.161257544
ENSG00000102931	ARL2BP	14.23374131	6.367787708	1.160450776
ENSG00000139291	TMEM19	2.829721929	1.266242139	1.160106977
ENSG00000237298	TTN-AS1	0.281859002	0.126128974	1.160073917
ENSG00000139318	DUSP6	106.413142	47.61895591	1.160068441
ENSG00000229835	KHSRPP1	4.477887	2.004011	1.159927695
ENSG00000166275	BORCS7	5.399679176	2.41656853	1.159913783
ENSG00000144671	SLC22A14	0.132143239	0.059163975	1.159311728
ENSG00000185633	NDUFA4L2	0.257126819	0.115158035	1.158865016
ENSG00000256966	RP11-613M10.8	0.598575171	0.26808289	1.158853292
ENSG00000188322	SBK1	13.407052	6.007178	1.158232728
ENSG00000105711	SCN1B	2.687694818	1.20438616	1.158071297
ENSG00000246596	RP11-1277A3.2	1.663160204	0.74544858	1.157746397
ENSG00000158856	DMTN	10.85270494	4.865018637	1.15753743
ENSG00000196159	FAT4	1.107813564	0.496661975	1.157378905
ENSG00000120334	CENPL	2.574949924	1.15465406	1.157083698
ENSG00000104611	SH2D4A	5.628811616	2.524473234	1.156847984
ENSG00000184992	BRI3BP	18.448189	8.273978	1.156826172
ENSG00000251571	DDX3P3	0.210579	0.094453	1.156693046
ENSG00000139351	SYCP3	0.126010272	0.05653824	1.156242466
ENSG00000148824	MTG1	2.691366403	1.208136064	1.155555868
ENSG00000280353	CTC-453G23.7	0.186682	0.083829	1.155061504
ENSG00000261611	AC010547.9	0.09776	0.0439	1.155023345
ENSG00000102921	N4BP1	5.699299399	2.559402759	1.154977389
ENSG00000185133	INPP5J	2.572531738	1.155744854	1.154365937
ENSG00000260139	CSPG4P13	0.607319525	0.272848862	1.154353727
ENSG00000163072	NOSTRIN	0.381192162	0.171264832	1.15428952
ENSG00000171791	BCL2	0.253316518	0.11382201	1.154161594
ENSG00000166313	APBB1	3.937793005	1.769502445	1.154043521
ENSG00000214300	SPDYE3	1.151766469	0.517657718	1.153777837
ENSG00000246174	KCTD21-AS1	0.235224929	0.105730013	1.153655993
ENSG00000184990	SIVA1	3.237932612	1.45596621	1.153096086
ENSG00000225639	PGAM1P2	4.892578	2.200579	1.152711686
ENSG00000211454	AKR7L	1.063210267	0.478279861	1.152499992
ENSG00000257122	RRN3P3	1.382910465	0.622281717	1.152067988
ENSG00000236608	EIF4A1P6	0.167972	0.07559	1.15195347
ENSG00000140398	NEIL1	1.114496529	0.501541456	1.151951264
ENSG00000224728	AC090945.1	0.393709	0.177208	1.151685957
ENSG00000176182	MYPOP	6.086899	2.740389	1.151326728
ENSG00000162391	FAM151A	0.287318	0.129361263	1.15124271
ENSG00000134575	ACP2	4.399611794	1.981696461	1.150640231
ENSG00000242798	RP11-506M12.1	1.461993	0.65853	1.150615333
ENSG00000132164	SLC6A11	0.008963893	0.00403879	1.150202379
ENSG00000268584	RP11-464F9.20	0.453264	0.204228	1.150170906
ENSG00000272148	RP11-195B17.1	1.751677	0.789258	1.15016789
ENSG00000189166	TNRC18P3	1.422169	0.640959	1.149788934
ENSG00000151617	EDNRA	3.504608615	1.57994174	1.149381976
ENSG00000260844	RP11-578F21.9	0.223028	0.10059	1.148737955

ENSG00000178826	TMEM139	1.911078832	0.862044512	1.148553019
ENSG00000188549	C15orf52	2.917996614	1.316244872	1.148550299
ENSG00000179342	GS1-124K5.9	0.888898	0.400968	1.148530777
ENSG00000258760	CTD-2509G16.5	0.10731168	0.048408701	1.148468813
ENSG00000232396	RP1-76B20.11	0.04345621	0.019604334	1.148389728
ENSG00000040487	PQLC2	2.693302408	1.21507203	1.148334387
ENSG00000138600	SPPL2A	8.140533148	3.673454732	1.147985788
ENSG00000249465	RBMXP4	1.675147	0.756049	1.147736058
ENSG00000257038	RP11-800A3.7	0.141483	0.063859	1.147666849
ENSG00000068654	POLR1A	6.809038594	3.073712856	1.147468715
ENSG00000226534	KB-1183D5.16	1.394147	0.629345	1.14745968
ENSG00000087085	ACHE	0.864252927	0.390201661	1.147233667
ENSG00000115129	TP53I3	2.781084006	1.255949404	1.146868977
ENSG00000110321	EIF4G2	102.9141895	46.48481551	1.146610475
ENSG00000184788	SATL1	0.100125127	0.045242486	1.146053961
ENSG00000283031	RP11-557N21.1	0.176135	0.0796	1.145841281
ENSG00000067064	IDI1	16.04661017	7.254269501	1.145366313
ENSG00000263421	MIR4687	0.922301	0.416974	1.145280233
ENSG00000233230	AC079807.2	1.501392522	0.678793053	1.145257499
ENSG00000272356	RP5-1112D6.8	0.911434	0.412076	1.145227749
ENSG00000166105	GLB1L3	0.100654583	0.045508675	1.14519939
ENSG00000196678	ERI2	4.628600944	2.092959047	1.145032103
ENSG00000244693	CTAGE8	2.556523	1.156015	1.145022888
ENSG00000014257	ACPP	0.399595269	0.180702947	1.144919469
ENSG00000220541	RP5-1170D6.1	0.348659	0.157674	1.144871939
ENSG00000078902	TOLLIP	4.121694173	1.864183778	1.144693368
ENSG00000243307	POM121L6P	0.053516	0.024205	1.144665193
ENSG00000182318	ZSCAN22	1.724169	0.779959	1.144430999
ENSG00000168734	PKIG	7.181159589	3.25015849	1.143706753
ENSG00000157315	TMED6	0.552710683	0.250238281	1.143221993
ENSG00000163125	RPRD2	6.842084579	3.098294615	1.142961603
ENSG00000167900	TK1	59.84569341	27.10321354	1.142783515
ENSG00000169221	TBC1D10B	7.36199793	3.334364744	1.142685416
ENSG00000225392	AC016700.4	2.086605	0.945268	1.142362199
ENSG00000207166	SNORA68	0.530179	0.240191	1.142297433
ENSG00000248909	HMGB1P21	0.31236	0.141522	1.142183376
ENSG00000180139	ACTA2-AS1	0.031562135	0.014300408	1.142138438
ENSG00000230712	GGTLC4P	0.610123	0.276444	1.142112858
ENSG00000107263	RAPGEF1	12.42668246	5.631124442	1.141946254
ENSG00000184743	ATL3	9.638299729	4.36820228	1.141739001
ENSG00000120093	HOXB3	3.06582634	1.38985158	1.141345152
ENSG00000101680	LAMA1	0.074351094	0.033722519	1.140641661
ENSG00000144063	MALL	48.54829656	22.0277952	1.140095575
ENSG00000169402	RSPH10B2	0.051134271	0.023201158	1.140093723
ENSG00000280202	RP5-1180D12.1	3.126352	1.418551	1.140062203
ENSG00000010165	METTL13	9.776641813	4.436300704	1.139981839
ENSG00000255875	CTD-2102P23.1	0.404294	0.18346	1.139939248
ENSG00000260675	CTD-2576D5.3	0.235136	0.106717	1.139705421

ENSG00000167613	LAIR1	0.294877556	0.133842661	1.139577989
ENSG00000273541	DLEU1_2	4.985829	2.263665	1.139172934
ENSG00000183628	DGCR6	1.454522418	0.66039373	1.139147205
ENSG00000140987	ZSCAN32	2.522547645	1.14595391	1.138332498
ENSG00000186687	LYRM7	4.812109595	2.186331812	1.13815713
ENSG00000154153	FAM134B	2.96190382	1.345904661	1.137948575
ENSG00000235033	RP11-61I13.3	0.034752838	0.015792363	1.137903729
ENSG00000278261	SNORD1A	1.486289	0.675454	1.137785239
ENSG00000174527	MYO1H	0.094942831	0.04315551	1.137514307
ENSG00000102543	CDADC1	2.627782795	1.194521964	1.13741265
ENSG00000126062	TMEM115	9.094898	4.135036	1.137157568
ENSG00000129173	E2F8	5.097581638	2.318240138	1.136782958
ENSG00000280219	RP11-752L20.3	0.101767	0.046282	1.1367467
ENSG00000152804	HHEX	4.036213329	1.83567424	1.136692368
ENSG00000216829	RP11-175I6.1	0.712547	0.324112	1.136492742
ENSG00000197584	KCNMB2	0.068246379	0.031048167	1.136244415
ENSG00000146243	IRAK1BP1	1.356266614	0.61710085	1.136062622
ENSG00000204428	LY6G5C	0.272269486	0.123892833	1.13594258
ENSG00000149557	FEZ1	0.960464377	0.437108866	1.135739464
ENSG00000223901	AP001469.5	0.227825604	0.103687143	1.135692877
ENSG00000122386	ZNF205	5.530540097	2.517408556	1.135481003
ENSG00000232627	AC021876.4	0.847797	0.385929	1.135383404
ENSG00000197106	SLC6A17	5.420377908	2.467681668	1.135237142
ENSG00000228008	CTD-2330K9.3	0.258118393	0.117525733	1.135056272
ENSG00000144668	ITGA9	0.556598163	0.253437854	1.135004123
ENSG00000280422	RP11-155D18.13	0.221029	0.100652	1.134859831
ENSG00000095587	TLL2	0.408656358	0.186204013	1.134004012
ENSG00000164619	BMPER	0.060508712	0.027574802	1.133792367
ENSG00000269954	RP11-148O21.6	0.706891	0.322146	1.133773093
ENSG00000271151	RP11-394I13.2	0.333589	0.152024	1.133772617
ENSG00000259601	RP11-568G20.2	0.089741	0.040897	1.133772242
ENSG00000203397	RP13-36G14.3	0.349287	0.159178	1.133771994
ENSG00000138152	BTBD16	0.148077542	0.067482352	1.133770685
ENSG00000269097	AC003682.17	0.23439	0.106817	1.133769749
ENSG00000204816	RP11-475I24.1	0.720942	0.328567	1.13369561
ENSG00000243485	RP11-34P13.3	0.194744901	0.088787307	1.133160213
ENSG00000231443	AC024937.6	1.85629	0.846419	1.132978196
ENSG00000163739	CXCL1	3.541948779	1.615187757	1.132841469
ENSG00000233995	KB-67B5.12	1.645672	0.750846	1.132087876
ENSG00000108590	MED31	8.088729421	3.690535505	1.132082932
ENSG00000067191	CACNB1	2.485171639	1.133919552	1.132027206
ENSG00000173825	TIGD3	0.524646	0.2394	1.131921154
ENSG00000234841	RP11-119H12.4	0.364606	0.166397	1.13170888
ENSG00000180694	TMEM64	10.54278031	4.811613587	1.131662689
ENSG00000196922	ZNF252P	2.205826789	1.006728259	1.131645192
ENSG00000155016	CYP2U1	2.538632583	1.158761577	1.131467855
ENSG00000268895	A1BG-AS1	0.072162366	0.032940943	1.131364781
ENSG00000130675	MNX1	1.782457425	0.813740522	1.131226878

ENSG00000084110	HAL	0.371016252	0.169398138	1.131064369
ENSG00000225246	RPS2P1	0.688905	0.31455	1.131015687
ENSG00000178567	EPM2AIP1	4.286975858	1.958622	1.130121298
ENSG00000243701	DUBR	0.982831561	0.449047701	1.130075479
ENSG00000258057	BCDIN3D-AS1	0.508958267	0.232642943	1.129429943
ENSG00000175604	RP11-276H1.3	0.051567	0.023578	1.12900677
ENSG00000228195	RPL5P27	0.057771	0.026415	1.128988058
ENSG00000151748	SAV1	16.8981776	7.72649596	1.128981474
ENSG00000280367	RP11-121L10.2	0.240502	0.109968	1.128965122
ENSG00000277511	CTD-2095E4.5	0.118363	0.054121	1.128957767
ENSG00000268087	CTC-429P9.2	6.445729	2.948664	1.128282097
ENSG00000247373	RP11-486O12.2	0.339539	0.155339	1.128157211
ENSG00000035141	FAM136A	21.37488036	9.779783065	1.128041976
ENSG00000235314	LINC00957	0.504924477	0.231061892	1.127788275
ENSG00000250999	RP11-1379J22.5	0.525793614	0.240618867	1.127746849
ENSG00000173041	ZNF680	0.828728815	0.379251648	1.127744636
ENSG00000178338	ZNF354B	1.545945641	0.707633435	1.127415471
ENSG00000260807	RP11-161M6.2	1.678818768	0.768602174	1.127137534
ENSG00000130204	TOMM40	27.09688734	12.41049907	1.126566005
ENSG00000273679	RP11-566K19.8	1.312762	0.601372	1.126275781
ENSG00000255458	RP11-539G18.3	0.154013249	0.070561342	1.126104564
ENSG00000100478	AP4S1	0.543982686	0.249254302	1.125942331
ENSG00000254468	RP11-304M2.6	0.057563	0.026377	1.125861294
ENSG00000174370	C11orf45	1.132474842	0.518979994	1.125728172
ENSG00000261678	SCRT1	0.031246	0.014321	1.125539277
ENSG00000166035	LIPC	0.02277709	0.010439528	1.125526954
ENSG00000232254	CSF2RBP1	0.603985	0.276831	1.125507213
ENSG00000237922	RP11-478H16.1	0.179999	0.082501	1.12550538
ENSG00000259051	HNRNPUP1	18.286461	8.382995	1.125238222
ENSG00000278770	Metazoa_SRP	0.294026	0.13482	1.124909204
ENSG00000115226	FNDC4	1.963781453	0.90055193	1.124753006
ENSG00000228801	RP11-110G21.1	0.26532206	0.12167266	1.124739604
ENSG00000143858	SYT2	0.078352507	0.035936826	1.124516421
ENSG00000205238	SPDYE2	1.410600474	0.647039837	1.124382986
ENSG00000230423	XX-C2158C6.1	0.104442	0.047909	1.124333384
ENSG00000173678	SPDYE2B	1.330996455	0.610563769	1.12429284
ENSG00000274528	CTD-2650P22.2	0.411401	0.188735	1.124183312
ENSG00000104228	TRIM35	7.335757357	3.366432065	1.123725569
ENSG00000236200	KDM4A-AS1	0.227544068	0.10442375	1.123696099
ENSG00000167747	C19orf48	15.01209008	6.890961719	1.123347604
ENSG00000015520	NPC1L1	0.179590385	0.082437531	1.123336916
ENSG00000233221	AC133785.1	0.220769003	0.101348393	1.123214407
ENSG00000254859	RP11-661A12.5	0.868711	0.39884	1.1230662
ENSG00000269609	RPARP-AS1	1.838510903	0.844572177	1.122245107
ENSG00000151023	ENKUR	0.08670891	0.039833496	1.122198138
ENSG00000100263	RHBDD3	4.245281088	1.950754511	1.121827844
ENSG00000159337	PLA2G4D	0.06280316	0.02886172	1.121679885
ENSG00000104866	PPP1R37	2.917544388	1.340997798	1.121447738

ENSG00000110768	GTF2H1		6.264694201	2.879547314	1.121402062
ENSG00000262514	RP11-96D1.9		0.297574	0.136783	1.121359542
ENSG00000276128	RP11-459O16.8		0.157196	0.072257	1.121355245
ENSG00000279621	RP11-67A1.4		0.30957	0.142298	1.121350284
ENSG00000169598	DFFB		2.385472745	1.096558468	1.121292465
ENSG00000010610	CD4		0.207412644	0.095349009	1.121214003
ENSG00000123080	CDKN2C		1.626551298	0.748031225	1.120645924
ENSG00000100167		3-Sep	19.08727186	8.77891397	1.120496532
ENSG00000120662	MTRF1		3.784294998	1.741188349	1.119952284
ENSG00000256576	RP13-977J11.2		1.172259	0.539422	1.119805088
ENSG00000123144	C19orf43		67.90084198	31.2461662	1.119750278
ENSG00000267649	CTD-2587H24.10		0.298175202	0.137274838	1.119093066
ENSG00000166257	SCN3B		0.136631232	0.062904202	1.119059008
ENSG00000265743	RP11-848P1.3		0.805131	0.370836	1.118442233
ENSG00000198855	FICD		1.842053455	0.848527068	1.11828234
ENSG00000233870	AC007881.4		6.134762	2.82758	1.117439533
ENSG00000277156	RP11-496I2.5		2.616549	1.206028	1.117401874
ENSG00000236905	RP11-348H3.2		1.582139	0.729328	1.117236667
ENSG00000100206	DMC1		0.099943974	0.046072882	1.117201738
ENSG00000261882	RP11-502F1.2		0.767325	0.353729	1.117193258
ENSG00000219249	AMZ2P2		0.333209	0.153606	1.117192798
ENSG00000236081	ELFN1-AS1		9.569151774	4.41185168	1.117006757
ENSG00000161180	CCDC116		0.172243568	0.079417274	1.116925373
ENSG00000272056	RP11-503P10.1		1.767679	0.815173	1.116678141
ENSG00000236337	FMR1-IT1		0.342145	0.157786	1.116638661
ENSG00000121274	PAPD5		4.254006089	1.961822265	1.116627754
ENSG00000225932	CTAGE4		2.527021	1.166111	1.115732531
ENSG00000184939	ZFP90		5.032080439	2.322354387	1.115566842
ENSG00000200332	Y_RNA		0.689594	0.318356	1.115106267
ENSG00000164465	DCBLD1		6.307316822	2.911940454	1.115045549
ENSG00000241478	HSPA8P9		9.541629	4.405165	1.115039534
ENSG00000250210	SEPT14P9		0.359394	0.165943	1.11487855
ENSG00000186998	EMID1		3.204504977	1.479956601	1.114546641
ENSG00000161132	XXbac-B444P24.10		0.283487	0.130937	1.114409749
ENSG00000128045	RASL11B		0.235103325	0.108591917	1.114378217
ENSG00000124659	TBCC		7.485775	3.458891	1.113842135
ENSG00000104450	SPAG1		5.969233961	2.758507767	1.113657758
ENSG00000134780	DAGLA		1.49721	0.691901	1.113639058
ENSG00000170456	DENND5B		7.381746908	3.411438234	1.113582178
ENSG00000236761	CTAGE9		1.822833	0.842532	1.113379007
ENSG00000187695	RP11-723O4.6		0.482198839	0.222882381	1.113345603
ENSG00000172461	FUT9		0.008532699	0.003944197	1.11327054
ENSG00000136982	DSCC1		17.34232027	8.016591149	1.11323613
ENSG00000093010	COMT		6.780576207	3.134459321	1.113191271
ENSG00000232832	LMLN-AS1		0.216001	0.099853	1.113160314
ENSG00000225251	RPL5P25		0.075524	0.034916	1.113046884
ENSG00000214526	AP000343.1		0.202703	0.093715	1.113015552
ENSG00000213332	SLC25A5P6		0.330308	0.152711	1.113007927

ENSG00000118194	TNNT2	0.29511442	0.136450362	1.112898194
ENSG00000087586	AURKA	23.91141261	11.05772948	1.11264418
ENSG00000273542	HIST1H4K	0.551376	0.254991	1.112590144
ENSG00000103942	HOMER2	0.237756395	0.109972731	1.112338316
ENSG00000249784	SCARNA22	0.40717	0.188336	1.112322474
ENSG00000065413	ANKRD44	0.594341798	0.274954266	1.112101173
ENSG00000278872	RP13-895J2.4	0.853324	0.394801	1.111967981
ENSG00000157184	CPT2	4.94170979	2.28710057	1.111490482
ENSG00000132622	HSPA12B	0.84654	0.391803	1.111449795
ENSG00000211697	TRGV5	0.208438211	0.09647177	1.11144104
ENSG00000158220	ESYT3	0.044068653	0.020400079	1.111178032
ENSG00000241250	RPL17P20	0.347847	0.161029	1.111132349
ENSG00000152952	PLOD2	15.34120992	7.102540261	1.111005259
ENSG00000189366	ALG1L	0.451202977	0.208903707	1.110938495
ENSG00000101353	MROH8	0.150693818	0.069778855	1.110758399
ENSG00000264517	AC003001.1	0.409813	0.189774	1.110683401
ENSG00000223566	TNRC18P2	0.818827	0.379282	1.110287769
ENSG00000272990	RP11-305K5.1	6.111552	2.83174	1.109849984
ENSG00000223799	IL10RB-AS1	0.099388	0.046073	1.109150132
ENSG00000165655	ZNF503	10.83471079	5.024085049	1.108727852
ENSG00000065371	ROPN1	0.149940121	0.06953202	1.108637068
ENSG00000186312	CA5BP1	2.714207784	1.258739705	1.108551191
ENSG00000250580	SNRPCP8	1.029697	0.47757	1.108435753
ENSG00000281433	Metazoa_SRP	0.355705	0.164977	1.108416346
ENSG00000250616	RP11-455F5.3	0.212114	0.098401	1.108094963
ENSG00000163508	EOMES	0.756849581	0.35113653	1.107974511
ENSG00000279792	RP11-893F2.18	0.462243	0.214476	1.107835254
ENSG00000281100	RP11-640L9.2	0.058044	0.026952	1.10675461
ENSG00000260647	RP1-178F10.1	0.311221	0.144514	1.106730149
ENSG00000213140	ELK2AP	0.077162	0.03583	1.106722494
ENSG00000234449	RP11-706O15.3	2.208274289	1.025608956	1.106438615
ENSG00000164961	KIAA0196	17.49478211	8.125481754	1.10639944
ENSG00000141540	TTYH2	6.955751118	3.230619337	1.106395544
ENSG00000248538	RP11-10A14.5	1.988642208	0.924070011	1.105709668
ENSG00000255627	RP11-547L9.1	0.437026	0.203079	1.105678051
ENSG00000273240	RP11-455J20.3	0.568539	0.264267	1.105263035
ENSG00000214894	LINC00243	0.58988327	0.274217522	1.105108735
ENSG00000242017	ALG1L15P	1.214821	0.564743	1.105077364
ENSG00000251574	RP11-6N13.1	0.168174968	0.078181508	1.105063673
ENSG00000281406	BLACAT1	2.171163	1.009383	1.104994346
ENSG00000272899	RP11-309L24.4	0.377902	0.1757	1.104897963
ENSG00000100084	HIRA	9.85642446	4.58270698	1.104864344
ENSG00000157613	CREB3L1	0.37481843	0.174279605	1.104788141
ENSG00000163449	TMEM169	0.512639494	0.238381411	1.104672888
ENSG00000176049	JAKMIP2	0.043899796	0.020422392	1.104062366
ENSG00000282885	RP11-596C23.6	0.175334755	0.081568299	1.104031522
ENSG00000276855	CTD-3157E16.2	2.095596	0.974954	1.103954557
ENSG00000175130	MARCKSL1	117.214661	54.534958	1.103899803

ENSG00000070388	FGF22	0.314024602	0.14611376	1.103785543
ENSG00000197798	FAM118B	6.626268129	3.083289854	1.103726062
ENSG00000132694	ARHGEF11	6.831618152	3.179118013	1.103600767
ENSG00000232075	MRPL35P2	0.713808	0.332208	1.103449255
ENSG00000147121	KRBOX4	2.876686615	1.338849019	1.103414785
ENSG00000170852	KBTBD2	5.528271557	2.573607101	1.103036663
ENSG00000175264	CHST1	1.497938074	0.69743583	1.102845594
ENSG00000233382	NKAPP1	0.368689171	0.171666836	1.102793694
ENSG00000224536	RP11-134G8.7	1.060897	0.494082	1.102462192
ENSG00000167617	CDC42EP5	0.496712292	0.231346286	1.102356514
ENSG00000124593	PRICKLE4	5.264678587	2.452710758	1.101968346
ENSG00000114547	ROPN1B	0.4074969	0.189846473	1.101955887
ENSG00000128573	FOXP2	0.282402684	0.13167703	1.100750093
ENSG00000142039	CCDC97	6.849640424	3.194141509	1.100600026
ENSG00000203825	RP11-744H18.1	0.014912	0.006954	1.100558793
ENSG00000144152	FBLN7	0.052923303	0.024682271	1.100427972
ENSG00000224687	RASAL2-AS1	0.14776299	0.068914082	1.100414244
ENSG00000226101	AC007461.2	0.168749	0.078702	1.100406749
ENSG00000162670	BRINP3	0.027068315	0.012624424	1.100387531
ENSG00000279569	RP11-394B2.1	0.576439	0.268864	1.100291351
ENSG00000235194	PPP1R3E	0.301924542	0.140864422	1.099880753
ENSG00000144677	CTDSPL	11.30898709	5.276580574	1.099794502
ENSG00000170365	SMAD1	3.384281211	1.579168374	1.09968445
ENSG00000273311	DGCR11	1.239072	0.578223	1.099562121
ENSG00000134504	KCTD1	1.042051673	0.486352587	1.099352322
ENSG00000256013	RP11-27M24.1	0.583309	0.272302	1.099052755
ENSG00000173141	MRPL57	8.910008	4.159523	1.099008633
ENSG00000168282	MGAT2	7.288367	3.402726	1.098904622
ENSG00000135835	KIAA1614	0.531237069	0.248043889	1.098760406
ENSG00000256806	C17orf100	0.2444005	0.114141772	1.098420378
ENSG00000099381	SETD1A	9.138761	4.268301	1.098336665
ENSG00000158483	FAM86C1	3.94730122	1.844062848	1.09797879
ENSG00000231490	RPL7L1P2	0.529082	0.247202	1.097800921
ENSG00000257702	LBX2-AS1	0.773700564	0.361495454	1.09779782
ENSG00000179588	ZFPM1	2.142097939	1.001197019	1.097298542
ENSG00000267544	AC007229.3	4.74743	2.22043	1.096307638
ENSG00000265100	RP11-147L13.2	0.264971	0.12394	1.096192598
ENSG00000065883	CDK13	4.419802041	2.0675003	1.096094215
ENSG00000241186	TDGF1	0.010153262	0.004749786	1.096009008
ENSG00000078328	RBFOX1	0.006197093	0.002899419	1.095827986
ENSG00000228427	RP5-1091N2.9	0.070105	0.0328	1.095821528
ENSG00000275939	uc_338	0.306604	0.143452	1.095808437
ENSG00000242683	RPL12P21	0.098255	0.045971	1.095806776
ENSG00000274991	AC004846.1	0.591816	0.276896	1.095804488
ENSG00000265386	RN7SL219P	0.343893	0.160899	1.09580439
ENSG00000241787	MTND4P16	0.074464	0.03484	1.095798492
ENSG00000240375	VPS26AP1	0.051988	0.024324	1.095798161
ENSG00000261072	RP11-5N19.3	0.11186	0.052337	1.0957911

ENSG00000233681	CEACAMP1	0.049175	0.023008	1.095789472
ENSG00000198795	ZNF521	0.016357731	0.007653847	1.095715645
ENSG00000175213	ZNF408	2.447182843	1.145442192	1.095217247
ENSG00000162757	C1orf74	3.053772	1.429592	1.094988886
ENSG00000261879	RP11-333E1.1	0.335758396	0.15719768	1.094843551
ENSG00000173762	CD7	0.158846166	0.074373539	1.094768945
ENSG00000110011	DNAJC4	4.279471703	2.003924388	1.094604634
ENSG00000116977	LGALS8	8.182246822	3.832566845	1.094186103
ENSG00000278463	HIST1H2AB	0.587961	0.275423	1.094071422
ENSG00000230177	RP5-1112D6.4	2.121412	0.993832	1.093950934
ENSG00000179674	ARL14	0.981738	0.460067	1.093494081
ENSG00000272636	DOC2B	6.04363069	2.832356174	1.093412806
ENSG00000228274	RP3-508I15.9	0.991665126	0.464747071	1.093407246
ENSG00000115596	WNT6	1.343684	0.62987	1.093067889
ENSG00000185904	LINC00839	5.219866921	2.446893816	1.09306153
ENSG00000183397	C19orf71	3.81586	1.789069	1.092799212
ENSG00000161653	NAGS	2.605995308	1.222087787	1.092486564
ENSG00000247844	CCAT1	4.68933	2.199119	1.092456134
ENSG00000255031	RP11-802E16.3	0.577579132	0.270911285	1.092198128
ENSG00000166828	SCNN1G	0.098687	0.046297	1.09194134
ENSG00000134283	PPHLN1	6.128926748	2.875783766	1.09167926
ENSG00000037749	MFAP3	0.97210495	0.456162335	1.091564748
ENSG00000121152	NCAPH	17.78692534	8.347072924	1.091474867
ENSG00000261744	RP11-21B21.4	0.270602	0.126995	1.091400805
ENSG00000069849	ATP1B3	75.35010094	35.37000025	1.091083215
ENSG00000261150	EPPK1	4.171457497	1.958255166	1.090982782
ENSG00000165671	NSD1	7.49803187	3.521718213	1.09023248
ENSG00000236559	RP11-243J16.7	1.201429	0.564358	1.090068863
ENSG00000100354	TNRC6B	5.097829948	2.394664717	1.090059574
ENSG00000155115	GTF3C6	8.448279318	3.968670575	1.090001719
ENSG00000229145	ACTBP1	0.380921	0.178943	1.089991717
ENSG00000137841	PLCB2	0.288916029	0.135825609	1.088894734
ENSG00000218730	RP3-453I5.2	0.56412	0.265271	1.088535121
ENSG00000198466	ZNF587	3.922485218	1.844700342	1.08838153
ENSG00000004848	ARX	0.348846	0.164092	1.088085387
ENSG00000148120	C9orf3	0.529202	0.248984588	1.087762073
ENSG00000249091	RP11-733C7.1	0.284013	0.133631	1.087702241
ENSG00000233871	DLG5-AS1	0.141134954	0.06640544	1.087702002
ENSG00000077935	SMC1B	0.047868484	0.022523208	1.087663804
ENSG00000163975	MFI2	9.450659224	4.4474715	1.087429607
ENSG00000197249	SERPINA1	5.080167574	2.390790918	1.087388119
ENSG00000132330	SCLY	1.712767556	0.806083832	1.087327583
ENSG00000154743	TSEN2	3.309074741	1.557626125	1.087078891
ENSG00000264570	SNX19P3	0.096603	0.04548	1.086835738
ENSG00000173137	ADCK5	4.591847401	2.162353932	1.086472017
ENSG00000159445	THEM4	7.278400603	3.427900882	1.086296065
ENSG00000278997	RP11-131M11.2	1.267166	0.596918	1.086000867
ENSG00000207445	SNORD15B	1.680272	0.791716	1.085639881

ENSG00000224773	HSPA8P7	4.303203	2.027613	1.085628581
ENSG00000169490	TM2D2	9.416744495	4.437426382	1.0855052
ENSG00000233771	CICP5	0.146621	0.069127	1.084770529
ENSG00000065457	ADAT1	4.304933625	2.029715247	1.084713651
ENSG00000126106	TMEM53	0.944786778	0.44563151	1.084137528
ENSG00000185684	EP400NL	1.474681588	0.695597061	1.08407974
ENSG00000206168	Z69890.1	1.46533	0.691274	1.083896034
ENSG00000149929	HIRIP3	3.577654104	1.687977568	1.08371818
ENSG00000198932	GPRASP1	0.162803142	0.076826965	1.08344387
ENSG00000216316	RP3-354N19.3	0.591166	0.278972	1.083442969
ENSG00000105708	ZNF14	2.021852	0.954313	1.083142965
ENSG00000275105	MIR6511B2	0.015842	0.007478	1.083030106
ENSG00000181744	C3orf58	6.633712738	3.132273323	1.082606432
ENSG00000113966	ARL6	1.557819714	0.735622635	1.082490503
ENSG00000102384	CENPI	4.863530038	2.296759992	1.082403723
ENSG00000251211	RP11-889L3.4	0.465809	0.220002	1.082221875
ENSG00000258101	RP11-977B10.2	0.315255	0.148989	1.081313433
ENSG00000237472	AP000361.2	0.717971	0.339314	1.081304616
ENSG00000271743	CTD-2541M15.3	0.287189	0.135726	1.081303378
ENSG00000160285	LSS	7.369153013	3.483178887	1.081094242
ENSG00000269890	RP5-1139B12.2	0.822892	0.388974	1.081029372
ENSG00000093009	CDC45	7.967329008	3.766128214	1.081014036
ENSG00000196141	SPATS2L	14.8662618	7.027349078	1.08098945
ENSG00000225792	AC004540.4	0.261510344	0.123640907	1.080711868
ENSG00000153363	LINC00467	3.660515184	1.73141867	1.080092088
ENSG00000111077	TNS2	2.13638425	1.010647983	1.079890571
ENSG00000274238	RP11-115D19.3	3.129028	1.480417	1.079710961
ENSG00000140104	C14orf79	2.653405651	1.255536474	1.07954131
ENSG00000161664	ASB16	0.934159811	0.442122667	1.079222677
ENSG00000258289	CHURC1	5.12439565	2.425322944	1.079205007
ENSG00000063587	ZNF275	3.794151633	1.795806475	1.079145451
ENSG00000187626	ZKSCAN4	1.912197	0.905124	1.079043805
ENSG00000078070	MCCC1	4.414659785	2.090246434	1.078629219
ENSG00000234559	AC079776.4	0.171947	0.081424	1.078437944
ENSG00000184677	ZBTB40	8.590620682	4.068189372	1.078375534
ENSG00000255986	MT1JP	1.028076193	0.486868633	1.078342727
ENSG00000173914	RBM4B	6.001674826	2.842332252	1.078289948
ENSG00000157240	FZD1	3.011085	1.426115	1.078193111
ENSG00000272769	RP11-725P16.2	0.543457	0.25741	1.078097788
ENSG00000228157	AC007952.5	0.06025828	0.028543231	1.078010839
ENSG00000267896	AC018766.4	3.294948	1.560785	1.077983879
ENSG00000234171	RNASEH1-AS1	3.484853	1.650808	1.077925466
ENSG00000129474	AJUBA	6.043065943	2.864121556	1.077187962
ENSG00000196275	GTF2IRD2	0.42654016	0.202218475	1.076766772
ENSG00000242689	CNTF	1.036654	0.491505	1.076656454
ENSG00000226401	RP5-965K10.3	0.391098	0.185466	1.076375425
ENSG00000121039	RDH10	3.250523473	1.541667573	1.07618036
ENSG00000092470	WDR76	8.790187211	4.169483057	1.076025366

ENSG00000119596	YLPM1	6.190213855	2.937238501	1.075528836
ENSG00000186532	SMYD4	2.461729344	1.168385994	1.075155184
ENSG00000137210	TMEM14B	11.23208959	5.331584395	1.074990118
ENSG00000237004	ZNRF2P1	0.257354	0.122168	1.074887763
ENSG00000254258	RP11-398H6.1	0.109937	0.05219	1.074831709
ENSG00000236830	CBR3-AS1	0.314045098	0.149088673	1.074801099
ENSG00000234949	AC104667.3	0.885766354	0.42050645	1.074798269
ENSG00000228878	SEPT7-AS1	0.126057297	0.059852081	1.074606319
ENSG00000124098	FAM210B	10.03723945	4.766904338	1.074237959
ENSG00000109758	HGFAC	0.090458081	0.042961597	1.074201748
ENSG00000176076	KCNE5	0.207317	0.098468	1.074111562
ENSG00000270084	GAS5-AS1	1.017924	0.483498	1.074048024
ENSG00000280254	RP11-81A22.4	0.668466	0.31763	1.073507002
ENSG00000063127	SLC6A16	1.624922321	0.772195684	1.073332357
ENSG00000227165	WDR11-AS1	0.027694598	0.013161936	1.073232933
ENSG00000104472	CHRAC1	15.58712259	7.40785678	1.073226517
ENSG00000137880	GCHFR	0.271731246	0.1291484	1.073150695
ENSG00000113838	TBCCD1	7.429892824	3.531475868	1.073070162
ENSG00000143147	GPR161	0.936867447	0.445445894	1.072594735
ENSG00000109062	SLC9A3R1	48.59423784	23.10864285	1.07235272
ENSG00000146535	GNA12	9.642334406	4.58591981	1.072171337
ENSG00000090565	RAB11FIP3	3.107735587	1.478068611	1.072150522
ENSG00000186566	GPATCH8	8.945929435	4.255254261	1.07198604
ENSG00000198730	CTR9	29.74560597	14.15052225	1.071821269
ENSG00000247372	CTD-2235C13.2	0.21155	0.100641	1.071780523
ENSG00000266401	RP11-874J12.4	0.998557	0.475103	1.071604466
ENSG00000182973	CNOT10	12.85632218	6.117041291	1.071572069
ENSG00000122483	CCDC18	2.965062348	1.410834043	1.071514148
ENSG00000278970	HEIH	3.288907	1.565302	1.071167185
ENSG00000213423	RBMX2P2	0.448346	0.213393	1.071099675
ENSG00000166348	USP54	4.755842396	2.264084342	1.070773204
ENSG00000239831	RNF7P1	0.811066	0.386123	1.070758824
ENSG00000116198	CEP104	5.810044392	2.766202897	1.070642207
ENSG00000183527	PSMG1	20.05706476	9.549813697	1.070565998
ENSG00000174871	CNIH2	1.030570944	0.490720362	1.070470784
ENSG00000058262	SEC61A1	91.72464179	43.68651264	1.070121421
ENSG00000112541	PDE10A	0.073615608	0.035063592	1.070037876
ENSG00000106609	TMEM248	17.06430607	8.131831868	1.069329456
ENSG00000081386	ZNF510	1.562969333	0.745127769	1.068729737
ENSG00000164077	MON1A	2.376579396	1.133044182	1.068682481
ENSG00000122035	RASL11A	2.746700527	1.309690533	1.068473664
ENSG00000187498	COL4A1	0.055446213	0.026438326	1.068458116
ENSG00000133818	RRAS2	12.60454395	6.010324094	1.068429228
ENSG00000273680	RP11-996F15.6	1.031997	0.492156	1.068251189
ENSG00000249274	PDLIM1P4	0.428351	0.204316	1.06799127
ENSG00000153283	CD96	0.237345425	0.113212578	1.06795399
ENSG00000273695	MIR6084	0.548291	0.26154	1.067910183
ENSG00000277103	RP11-520B13.8	0.165206	0.078828	1.067486007

ENSG00000117751	PPP1R8	18.07072924	8.623385778	1.0673284
ENSG00000137261	KIAA0319	0.624653373	0.298220886	1.066674543
ENSG00000264349	MIR4258	0.548113	0.261681	1.066664175
ENSG00000183978	COA3	5.008083994	2.391215182	1.066514797
ENSG00000184470	TXNRD2	1.077712833	0.514578135	1.066510747
ENSG00000198954	KIF1BP	15.11219151	7.218074501	1.06602695
ENSG00000247400	DNAJC3-AS1	0.388907805	0.185829518	1.065448506
ENSG00000163273	NPPC	0.422946541	0.202130557	1.065187882
ENSG00000276259	RP11-481J2.4	0.826448	0.394985	1.065126182
ENSG00000130508	PXDN	7.660879811	3.661439852	1.065098991
ENSG00000162734	PEA15	32.86660874	15.71071058	1.064874169
ENSG00000148153	INIP	8.041575026	3.844111601	1.064827875
ENSG00000110195	FOLR1	7.185821676	3.435298618	1.064717621
ENSG00000110092	CCND1	243.0298637	116.1983335	1.064544227
ENSG00000174744	BRMS1	10.92111098	5.222126019	1.064410448
ENSG00000272690	RP11-803B1.8	0.15963158	0.076333328	1.0643611
ENSG00000157110	RBPMS	1.558263368	0.74531345	1.064019888
ENSG00000264324	RP11-287D1.3	0.742923	0.355379	1.063854259
ENSG00000149346	SLX4IP	5.460149934	2.611907757	1.06383662
ENSG00000165891	E2F7	6.850047937	3.277252891	1.063627081
ENSG00000196689	TRPV1	1.623725213	0.776861996	1.063577262
ENSG00000205683	DPF3	0.426880765	0.204258203	1.063439139
ENSG00000271585	CTB-88F18.4	0.14337	0.068603	1.063399602
ENSG00000261104	RP11-734K21.5	0.031101	0.014882	1.063392544
ENSG00000093217	XYLB	1.447367406	0.692577647	1.063383458
ENSG00000161850	KRT82	0.019105	0.009142	1.063368534
ENSG00000247735	CTD-2574D22.2	0.57251	0.273981	1.063225044
ENSG00000126243	LRFN3	3.949132273	1.890186755	1.063006907
ENSG00000234494	SP2-AS1	0.393836866	0.188608015	1.062207182
ENSG00000157657	ZNF618	3.550800831	1.700481444	1.062201177
ENSG00000177576	C18orf32	5.681662451	2.721265062	1.062035636
ENSG00000200013	RNU6-623P	1.076091	0.515433	1.061943273
ENSG00000228889	UBAC2-AS1	0.574199	0.275034	1.061940841
ENSG00000224086	LL22NC03-86G7.1	0.321644	0.154081	1.061775805
ENSG00000181638	ZFP41	1.990435846	0.953944315	1.061107414
ENSG00000213222	AC093724.2	8.703896	4.172605	1.060712964
ENSG00000002746	HECW1	0.75034594	0.359762466	1.060511212
ENSG00000171606	ZNF274	1.364156691	0.654167568	1.060277225
ENSG00000162069	CCDC64B	2.555196819	1.225347242	1.06024378
ENSG00000160972	PPP1R16A	7.008567633	3.361661524	1.059945154
ENSG00000132819	RBM38	10.77753052	5.169677775	1.059880383
ENSG00000239437	RN7SL752P	2.010514	0.964418	1.059833899
ENSG00000125454	SLC25A19	5.00764538	2.402340919	1.0596915
ENSG00000249210	GAPDHP38	1.368868	0.656972	1.059079545
ENSG00000139719	VPS33A	4.143882329	1.9891263	1.058848153
ENSG00000026751	SLAMF7	0.093304913	0.044792186	1.058705984
ENSG00000268996	MAN1B1-AS1	0.481774	0.231284	1.058691072
ENSG00000248907	MTND2P33	0.189071	0.090813	1.057957356

ENSG00000236861	AC006378.2	0.324166725	0.155724384	1.057741146
ENSG00000169084	DHRSX	1.399067625	0.672189285	1.057526247
ENSG00000214544	GTF2IRD2P1	0.419204043	0.201444081	1.057273216
ENSG00000168917	SLC35G2	1.277212693	0.61379569	1.057168375
ENSG00000136213	CHST12	0.831358353	0.399626446	1.056818421
ENSG00000223819	RP3-347M6.1	0.745052	0.358144	1.056801348
ENSG00000157350	ST3GAL2	2.136674777	1.027135033	1.056741474
ENSG00000143776	CDC42BPA	18.35015633	8.82253512	1.05652718
ENSG00000120948	TARDBP	28.1612338	13.54050424	1.056429077
ENSG00000226784	PGAM4	25.220472	12.127177	1.056351521
ENSG00000172113	NME6	1.006764837	0.484309098	1.055726724
ENSG00000263126	CTC-479C5.10	0.259064	0.12467	1.055194206
ENSG00000177842	ZNF620	2.387429681	1.149101629	1.054951841
ENSG00000242220	TCP10L	0.14562218	0.070109666	1.054544845
ENSG00000228624	RP3-399L15.3	0.068001443	0.032740312	1.054497281
ENSG00000185792	NLRP9	0.062466116	0.030075603	1.054480186
ENSG00000120278	PLEKHG1	2.699191657	1.299729201	1.054316351
ENSG00000105929	ATP6V0A4	0.516532827	0.248734351	1.05425428
ENSG00000176108	CHMP6	5.127877542	2.469993233	1.05385472
ENSG00000167202	TBC1D2B	4.090976365	1.970566595	1.053834697
ENSG00000235920	AC073109.2	0.689198	0.332027	1.053617951
ENSG00000188078	RP5-1119A7.11	0.379429	0.18284	1.053248225
ENSG00000196517	SLC6A9	1.805144017	0.869927847	1.05314629
ENSG00000178896	EXOSC4	16.54114767	7.971560694	1.053125224
ENSG00000116863	ADPRHL2	10.460574	5.041516	1.053032491
ENSG00000236005	RP11-438P9.1	0.309301	0.149108	1.052653834
ENSG00000187051	RPS19BP1	5.993678015	2.889754457	1.052494671
ENSG00000248159	HSPA8P11	3.568821	1.720937	1.052253258
ENSG00000135097	MSI1	10.91445924	5.263534802	1.052136761
ENSG00000226466	RPA2P2	0.371704	0.179269	1.052028181
ENSG00000072310	SREBF1	18.18649308	8.771180839	1.052024387
ENSG00000129910	CDH15	1.688483836	0.814363362	1.051983807
ENSG00000262580	RP11-334C17.5	0.503169	0.242711811	1.051798724
ENSG00000076321	KLHL20	4.901611722	2.364452052	1.05175032
ENSG00000096080	MRPS18A	22.28489101	10.75108677	1.051583403
ENSG00000186076	RP11-887P2.3	67.552979	32.598164	1.051228681
ENSG00000172379	ARNT2	1.597996048	0.771153822	1.051173271
ENSG00000125352	RNF113A	4.55696	2.199222	1.051078463
ENSG00000136866	ZFP37	0.441196811	0.212938482	1.050985669
ENSG00000203667	COX20	6.52693512	3.150397364	1.050871889
ENSG00000115325	DOK1	3.899097477	1.882126597	1.050776551
ENSG00000123454	DBH	0.495368713	0.239147446	1.050602367
ENSG00000105438	KDELR1	89.01469813	42.97659476	1.050492398
ENSG00000118785	SPP1	2.819546973	1.361290154	1.050488773
ENSG00000113368	LMNB1	36.85080559	17.7950957	1.050216466
ENSG00000104827	CGB	0.257093	0.12415	1.050206068
ENSG00000060069	CTDP1	5.149520885	2.487136692	1.04995241
ENSG00000115998	C2orf42	2.556143096	1.234727336	1.049776115

ENSG00000160766	GBAP1		2.362050976	1.141034222	1.049698039
ENSG00000259207	ITGB3		0.405076998	0.195685901	1.049656349
ENSG00000186205		1-Mar	2.32723399	1.12438578	1.049479159
ENSG00000063761	ADCK1		1.53305923	0.74083846	1.049182536
ENSG00000161547	SRSF2		39.68861627	19.18796128	1.048523831
ENSG00000278594	RP11-146E13.5		2.541981	1.229191	1.048244138
ENSG00000234176	HSPA8P1		41.268192	19.956295	1.048186333
ENSG00000150456	N6AMT2		4.578885157	2.214940335	1.047728545
ENSG00000179152	TCAIM		2.885781978	1.395983346	1.047680577
ENSG00000259699	HMGB1P8		1.950738	0.943719	1.047590771
ENSG00000107957	SH3PXD2A		3.296971016	1.595257027	1.047352315
ENSG00000144567	FAM134A		10.03620178	4.856274991	1.047291358
ENSG00000156603	MED19		8.141360795	3.939587643	1.047225324
ENSG00000227881	ASS1P5		0.343005	0.165987	1.047159352
ENSG00000264943	SH3GL1P2		0.497515	0.240758	1.047156286
ENSG00000224066	RP4-622L5.7		0.134551963	0.065116364	1.047071391
ENSG00000152076	CCDC74B		1.184323813	0.573355682	1.046561291
ENSG00000053372	MRTO4		18.22481545	8.823900904	1.046415711
ENSG00000162174	ASRGL1		4.776443268	2.312707166	1.046354125
ENSG00000171314	PGAM1		79.29957874	38.39841625	1.046266394
ENSG00000261635	RP11-618N24.1		1.951419	0.945056	1.046051855
ENSG00000158806	NPM2		2.032074956	0.984290514	1.045797523
ENSG00000174173	TRMT10C		21.32725067	10.33048831	1.045789547
ENSG00000254862	RP11-159H22.2		0.05813052	0.028159143	1.045692374
ENSG00000214668	SLC29A4P1		1.274065	0.617194	1.045642941
ENSG00000263443	RP11-973F15.2		0.891954	0.432101	1.045600739
ENSG00000125952	MAX		11.92042258	5.775098536	1.045517913
ENSG00000136731	UGGT1		19.28693437	9.344818938	1.045385231
ENSG00000167842	MIS12		5.79547073	2.808706373	1.045020037
ENSG00000034693	PEX3		9.6152394	4.660986986	1.044687293
ENSG00000128335	APOL2		5.696476225	2.761478534	1.044628848
ENSG00000143450	OAZ3		0.36829793	0.178543831	1.044595004
ENSG00000251633	GYG1P1		0.788235	0.382256	1.044086664
ENSG00000234152	TCEB2P1		6.150315	2.983452	1.043677737
ENSG00000280033	CTD-2095E4.3		0.525752	0.255045	1.043630615
ENSG00000227080	CTD-2021A8.3		0.988451	0.479579	1.043400963
ENSG00000240230	COX19		3.369620545	1.634913075	1.043372205
ENSG00000230733	AC092171.4		6.009808	2.916	1.043328181
ENSG00000216285	RP11-490H24.5		58.23983	28.26009	1.043240084
ENSG00000280356	RP1-101G11.3		0.008765	0.004255	1.042594959
ENSG00000227627	RP1-101K10.6		0.157345	0.076388	1.04251341
ENSG00000278629	MIR6717		0.270151	0.131154	1.042504214
ENSG00000088986	DYNLL1		36.90273174	17.91646412	1.042441672
ENSG00000257663	RP11-1100L3.7		3.780569	1.836388	1.041732476
ENSG00000278932	bP-21264C1.2		0.148627378	0.072199881	1.041631523
ENSG00000118600	TMEM5		2.424233938	1.177842361	1.041382459
ENSG00000203387	AC074019.2		0.301848	0.146659	1.041356635
ENSG00000107021	TBC1D13		5.308491149	2.57935486	1.041291589

ENSG00000176476	SGF29	3.804209864	1.848532045	1.041216783
ENSG00000167840	ZNF232	1.693532564	0.822934052	1.041187002
ENSG00000119915	ELOVL3	5.743363	2.79116	1.04103092
ENSG00000246922	UBAP1L	0.160444384	0.077983139	1.040839156
ENSG00000124615	MOCS1	3.173790409	1.54326061	1.040225148
ENSG00000273576	RP11-390P24.1	2.24259	1.090801	1.039777957
ENSG00000269929	RP11-2B6.2	1.317982	0.64115	1.039596841
ENSG00000157111	TMEM171	4.758049476	2.314665807	1.039566362
ENSG00000053108	FSTL4	0.238339058	0.115979587	1.039144492
ENSG00000013275	PSMC4	57.74446154	28.10120191	1.039050743
ENSG00000105968	H2AFV	19.45745292	9.469898509	1.038901997
ENSG00000172602	RND1	0.58302492	0.283787752	1.038745224
ENSG00000183691	NOG	1.337506	0.651048	1.038709545
ENSG00000139505	MTMR6	8.000278669	3.894449191	1.038630957
ENSG00000260537	RP11-529K1.3	1.898375543	0.924234425	1.038434687
ENSG00000282682	C11orf71	1.428378	0.69542	1.038421355
ENSG00000258545	RHOXF1-AS1	0.273554201	0.133187167	1.038371632
ENSG00000070731	ST6GALNAC2	1.554462857	0.756890905	1.038258868
ENSG00000198569	SLC34A3	0.141881837	0.069090122	1.038138538
ENSG00000216835	RBMXP1	0.365447	0.177979	1.037955165
ENSG00000267258	CTC-448F2.3	0.786857	0.383354	1.037424237
ENSG00000123201	GUCY1B2	0.572980516	0.279158486	1.037401665
ENSG00000232917	HSPE1P6	2.094732	1.020727	1.037168618
ENSG00000125449	ARMC7	9.254845779	4.51002926	1.037072156
ENSG00000214748	RP11-332E4.1	1.879245	0.915886	1.036913222
ENSG00000073711	PPP2R3A	4.031725729	1.965198932	1.036722138
ENSG00000163904	SENP2	10.75556764	5.243627992	1.036446424
ENSG00000225187	AC073283.7	1.592906	0.776629	1.036363649
ENSG00000237523	LINC00857	2.847937653	1.388537421	1.036351505
ENSG00000235106	LINC00094	2.630325643	1.282980256	1.035742452
ENSG00000113732	ATP6V0E1	41.09988359	20.04795004	1.035679583
ENSG00000109572	CLCN3	16.80263964	8.199764561	1.035033502
ENSG00000154529	CNTNAP3B	3.037072285	1.482362585	1.034782873
ENSG00000161958	FGF11	0.212617528	0.103802848	1.034414513
ENSG00000091879	ANGPT2	0.378426112	0.184781129	1.034194211
ENSG00000214013	GANC	1.136105429	0.55477404	1.034124536
ENSG00000225244	RP11-220H4.2	0.589423	0.287847	1.034001181
ENSG00000162999	DUSP19	0.535848866	0.261743272	1.033673696
ENSG00000204444	APOM	0.500046836	0.244258907	1.033652056
ENSG00000163171	CDC42EP3	9.771132836	4.773951594	1.033341896
ENSG00000168612	ZSWIM1	4.37939	2.139804	1.033251277
ENSG00000112697	TMEM30A	34.45699332	16.83832906	1.033047839
ENSG00000159231	CBR3	1.118833	0.546761	1.033012466
ENSG00000163472	TMEM79	1.553269523	0.759134708	1.032880368
ENSG00000172171	TEFM	2.722033445	1.330405152	1.032819132
ENSG00000169057	MECP2	3.020756005	1.476541896	1.032687365
ENSG00000144785	RP11-977G19.10	2.157311707	1.054739486	1.032347938
ENSG00000163002	NUP35	6.726465451	3.289530042	1.031967124

ENSG00000207171	SNORA51	0.38852	0.190041	1.031678163
ENSG00000266274	RN7SL138P	1.064032	0.520462	1.031676801
ENSG00000271967	RP11-134K13.4	0.187463	0.091696	1.031675169
ENSG00000265619	AC093824.1	1.007846	0.49298	1.031674187
ENSG00000263727	RP11-806L2.5	0.189911	0.092894	1.03166615
ENSG00000100285	NEFH	0.026908	0.013162	1.031656436
ENSG00000254221	PCDHGB1	0.021326843	0.01043208	1.031643553
ENSG00000131401	NAPSB	0.086643592	0.042391817	1.031307245
ENSG00000100664	EIF5	24.96828504	12.21623265	1.03129729
ENSG00000215586	RP5-836E8.1	4.504072	2.203995	1.031108939
ENSG00000260641	RP11-1299A16.3	0.251462	0.123058	1.031001951
ENSG00000109063	MYH3	0.811520862	0.397147712	1.030952491
ENSG00000181847	TIGIT	0.033423959	0.016360252	1.03068769
ENSG00000275328	SPDYE19P	0.198445	0.097159	1.030319666
ENSG00000226942	IL9RP3	0.152526	0.074682	1.030222721
ENSG00000218510	LINC00339	1.790483334	0.876706941	1.030182515
ENSG00000165449	SLC16A9	3.594680762	1.760186023	1.030135746
ENSG00000213380	COG8	2.657408576	1.301581608	1.029754288
ENSG00000242802	AP5Z1	1.472384945	0.721164953	1.029753712
ENSG00000273148	RP5-1068E13.7	0.294332	0.14418	1.029573347
ENSG00000196663	TECPR2	1.422547404	0.697038101	1.029167305
ENSG00000079308	TNS1	0.9294873	0.455518328	1.028926052
ENSG00000132669	RIN2	0.327757695	0.160659016	1.028627705
ENSG00000268510	IFNL3P1	0.542686	0.266045	1.028447401
ENSG00000103174	NAGPA	1.534272063	0.752157706	1.02844724
ENSG00000147206	NXF3	0.135877298	0.06661455	1.028395197
ENSG00000241839	PLEKHO2	6.09484161	2.988124444	1.028348497
ENSG00000270923	TAS2R6P	0.753364	0.369415	1.028104647
ENSG00000198270	TMEM116	0.484347518	0.23750954	1.028057084
ENSG00000224464	PGAM1P6	0.374284	0.183541	1.028031004
ENSG00000129946	SHC2	0.541579615	0.265689786	1.027430668
ENSG00000213514	RP11-428P16.2	0.626737	0.307475	1.027391053
ENSG00000185340	GAS2L1	6.316782425	3.099092285	1.027344165
ENSG00000198948	MFAP3L	3.568957683	1.751255036	1.027113596
ENSG00000272572	RP11-179B2.2	0.404185	0.198365	1.026858286
ENSG00000237950	RP11-7O11.3	0.593941293	0.291493702	1.026855624
ENSG00000228606	RP11-574F21.2	0.677237	0.332395	1.026762114
ENSG00000139579	NABP2	16.60463144	8.151070477	1.026524255
ENSG00000131165	CHMP1A	36.23030009	17.78637887	1.026423933
ENSG00000165194	PCDH19	1.000010381	0.490946	1.026378723
ENSG00000256663	RP11-424C20.2	8.511314	4.178906	1.026256569
ENSG00000266282	UBL5P2	2.767917	1.359192	1.026051416
ENSG00000081377	CDC14B	3.473509631	1.705852592	1.025901113
ENSG00000225406	AC080125.1	1.473435	0.723717	1.025685852
ENSG00000137776	SLTM	18.53766657	9.105540479	1.025643097
ENSG00000070413	DGCR2	13.70097841	6.730016587	1.025596956
ENSG00000119231	SENP5	7.251437974	3.563358881	1.025029322
ENSG00000206344	HCG27	0.304172674	0.14948335	1.024905747

ENSG00000100060	MFNG	0.860174687	0.422753498	1.024812984
ENSG00000189339	SLC35E2B	6.536508555	3.212692175	1.024737478
ENSG00000280216	RP3-333H23.9	1.627174	0.799811	1.024637504
ENSG00000247626	MARS2	5.493217	2.700111	1.024632567
ENSG00000213386	RP11-779O18.2	0.509852	0.250664	1.024323709
ENSG00000183773	AIFM3	0.246764501	0.121326971	1.024234569
ENSG00000152990	ADGRA3	11.29154109	5.551979781	1.024168182
ENSG00000109066	TMEM104	7.871037765	3.870417292	1.024064742
ENSG00000233487	RPSAP69	0.430153928	0.211546075	1.023881094
ENSG00000265137	AL121893.2	0.727089	0.357582	1.023857854
ENSG00000177042	TMEM80	0.96065736	0.472560395	1.023523231
ENSG00000281181	CH507-513H4.3	0.758388	0.373117	1.023308043
ENSG00000187796	CARD9	0.112633333	0.055421818	1.023107904
ENSG00000161395	PGAP3	1.975183199	0.972074656	1.022847447
ENSG00000224678	GAPDHP46	0.147466	0.072596	1.022420399
ENSG00000234941	RP11-508N22.11	2.714465	1.33631	1.022413156
ENSG00000164520	RAET1E	0.964551869	0.474899761	1.022235792
ENSG00000068615	REEP1	4.532935323	2.231917143	1.022162107
ENSG00000239672	NME1	60.35086516	29.72727431	1.021587264
ENSG00000232344	AC087163.2	0.386412	0.190342	1.021545962
ENSG00000150990	DHX37	7.642754837	3.764915613	1.021475224
ENSG00000176273	SLC35G1	2.96582769	1.461061967	1.021417415
ENSG00000230547	HMGB1P11	2.443463	1.20433	1.020696498
ENSG00000249072	RP11-777B9.5	2.090482	1.03043	1.020589119
ENSG00000168246	UBTD2	15.714455	7.746729	1.020433062
ENSG00000213205	STRADBP1	0.582201	0.287009	1.02042134
ENSG00000171016	PYGO1	0.076633978	0.037785131	1.020165574
ENSG00000278817	AC007325.4	4.870875	2.401819	1.020053526
ENSG00000168061	SAC3D1	5.877765867	2.898747313	1.019838315
ENSG00000079215	SLC1A3	16.33920907	8.059604161	1.01955726
ENSG00000177192	PUS1	6.926857945	3.417913008	1.019085409
ENSG00000277283	RP1-267D11.6	1.548275	0.763994	1.019028528
ENSG00000263503	RP11-707O23.5	0.81404	0.401737	1.018848347
ENSG00000138685	FGF2	1.611666	0.795647404	1.018351654
ENSG00000197933	ZNF823	2.777385674	1.371564915	1.017904621
ENSG00000063322	MED29	8.793070871	4.344398896	1.017210521
ENSG00000258736	RP11-982M15.7	0.139383874	0.06886717	1.017175359
ENSG00000280242	RP5-1037N22.2	0.623739	0.308203	1.017061563
ENSG00000185168	LINC00482	1.295101212	0.639978858	1.016968696
ENSG00000274114	CTC-281F24.3	0.21448792	0.106003678	1.016782081
ENSG00000235919	ASH1L-AS1	0.566863533	0.280209735	1.016494383
ENSG00000179348	GATA2	3.799661319	1.878290584	1.016450556
ENSG00000266117	FBXO36P1	0.323099	0.159718	1.016449374
ENSG00000143457	GOLPH3L	11.01558531	5.445408321	1.016434016
ENSG00000262481	TMEM256-PLSCR3	0.242077503	0.119675	1.016347205
ENSG00000265092	MIR4484	0.527358	0.260822	1.015717112
ENSG00000282100	HSP90AB4P	0.069521	0.034384	1.015711446
ENSG00000152763	WDR78	0.327140871	0.161807129	1.015636835

ENSG00000251203	RP11-14I17.1	0.378361	0.187143	1.015622303
ENSG00000204099	NEU4	0.019537762	0.00966615	1.015251959
ENSG00000144909	OSBPL11	9.944251	4.920048	1.015190321
ENSG00000214491	SEC14L6	0.17488	0.086531	1.015076326
ENSG00000183684	ALYREF	58.77698612	29.09142872	1.014657234
ENSG00000271147	ARMCX5-GPRASP2	0.914971674	0.452898216	1.014540224
ENSG00000111605	CPSF6	22.05445512	10.91838155	1.014311099
ENSG00000258301	RP11-488C13.5	0.975564766	0.483184275	1.01366415
ENSG00000145860	RNF145	11.95424271	5.920839827	1.013649009
ENSG00000184083	FAM120C	0.275160344	0.136300625	1.013480382
ENSG00000235499	AC073046.25	0.840392	0.416311	1.01340075
ENSG00000267288	RP13-890H12.2	2.836578355	1.405237543	1.01333769
ENSG00000261349	RP3-465N24.5	0.337936	0.167432	1.013174762
ENSG00000143543	JTB	22.79699487	11.29705318	1.012897162
ENSG00000124688	MAD2L1BP	7.421754758	3.67789488	1.012880084
ENSG00000178917	ZNF852	0.841729717	0.417197707	1.012625822
ENSG00000009790	TRAF3IP3	0.039403467	0.019531413	1.01252625
ENSG00000161940	BCL6B	0.124767249	0.0618796	1.01170351
ENSG00000276555	AC008740.1	0.166852	0.082754	1.011668027
ENSG00000276305	AC106788.1	0.166852	0.082754	1.011668027
ENSG00000139546	TARBP2	3.870447479	1.919839548	1.01151463
ENSG00000282386	RP11-422P24.12	0.599285	0.297282	1.011410151
ENSG00000196741	LINC01560	0.522366	0.259156	1.011240202
ENSG00000196072	BLOC1S2	16.05122268	7.964328827	1.011058504
ENSG00000165757	KIAA1462	13.55946528	6.728009306	1.011048681
ENSG00000205361	MT1DP	1.11918377	0.555373	1.010918
ENSG00000187713	TMEM203	3.61776	1.795328	1.01084926
ENSG00000278665	RP11-666O2.4	0.240725	0.119514	1.010206351
ENSG00000153237	CCDC148	0.399380906	0.198299512	1.010084237
ENSG00000114098	ARMC8	4.789152457	2.377910705	1.010075823
ENSG00000279278	CH17-264L24.1	0.740927	0.367917	1.009951069
ENSG00000230304	CICP6	0.116706	0.057969	1.00952523
ENSG00000231884	NDUFB1P1	8.214082	4.080087	1.009499434
ENSG00000267309	CTD-2630F21.1	0.200367	0.099532	1.009412581
ENSG00000155508	CNOT8	14.29187972	7.100528092	1.009197445
ENSG00000123219	CENPK	3.541764096	1.759925453	1.008953802
ENSG00000100079	LGALS2	0.745751702	0.370588127	1.008878705
ENSG00000256779	RP11-60C6.5	0.165304	0.082152	1.008754032
ENSG00000127084	FGD3	0.69028773	0.343067055	1.008707251
ENSG00000151233	GXYLT1	4.636667	2.305808	1.00781573
ENSG00000113448	PDE4D	0.292829824	0.14563102	1.007744809
ENSG00000120694	HSPH1	22.65146347	11.2665974	1.007552388
ENSG00000094841	UPRT	3.513911044	1.747949618	1.007414068
ENSG00000164081	TEX264	2.69527839	1.341172467	1.006939523
ENSG00000023697	DERA	7.024558505	3.495511905	1.006903806
ENSG00000141965	FEM1A	4.032769	2.006782	1.006886867
ENSG00000198835	GJC2	0.286053	0.142349	1.006850117
ENSG00000140848	CPNE2	8.197283937	4.079679856	1.006690027

ENSG00000225083	GRTP1-AS1	0.447184955	0.222593595	1.006459572
ENSG00000168802	CHTF8	19.97490927	9.944803699	1.006174151
ENSG00000169359	SLC33A1	4.63830772	2.309954334	1.005734205
ENSG00000238085	RP11-435F13.2	1.602343	0.798173	1.005409624
ENSG00000257543	RP11-321F8.4	0.120399	0.059975	1.005390252
ENSG00000118894	EEF2KMT	4.89017568	2.437303217	1.004600552
ENSG00000198646	NCOA6	8.217329743	4.095717797	1.004553346
ENSG00000132821	VSTM2L	0.166277	0.08288	1.004492716
ENSG00000240138	EEF1GP4	0.083075	0.041409	1.004470025
ENSG00000106608	URGCP	2.553613726	1.273058829	1.004241222
ENSG00000070778	PTPN21	2.486106385	1.239464487	1.004171097
ENSG00000128185	DGCR6L	13.80154099	6.881289112	1.004078595
ENSG00000166415	WDR72	2.199683491	1.096932366	1.003821376
ENSG00000115844	DLX2	2.100968264	1.047788438	1.003706922
ENSG00000131094	C1QL1	6.232525	3.108483	1.003606076
ENSG00000183496	MEX3B	2.972598849	1.482735079	1.003463932
ENSG00000258450	RP11-649E7.7	0.628596	0.313577	1.003313346
ENSG00000174469	CNTNAP2	3.217742469	1.605283786	1.003220502
ENSG00000240204	SMKR1	2.075147427	1.03535	1.003095282
ENSG00000100604	CHGA	0.031830853	0.015883504	1.002896615
ENSG00000137491	SLCO2B1	0.0368774	0.0184043	1.002694047
ENSG00000185920	PTCH1	1.356416305	0.676951906	1.002674785
ENSG00000076258	FMO4	1.531989578	0.764798937	1.00225406
ENSG00000171813	PWWP2B	2.717792	1.35679	1.002237605
ENSG00000200291	Y_RNA	0.998963	0.498758	1.002091262
ENSG00000169951	ZNF764	1.525414697	0.761650919	1.001999669
ENSG00000178695	KCTD12	2.914451	1.45556	1.001649836
ENSG00000105642	KCNN1	0.217295275	0.108539816	1.001432441
ENSG00000099810	MTAP	22.32234541	11.15080368	1.001340925
ENSG00000115392	FANCL	8.110646909	4.052000085	1.001182785
ENSG00000282393	CTD-3113P16.11	1.9506	0.974564	1.001089126
ENSG00000240459	RP11-477G18.1	0.129179	0.06456	1.000659073
ENSG00000230098	TCERG1L-AS1	0.215661	0.107782	1.000649041
ENSG00000278338	VWA8-AS1	0.047345115	0.023661921	1.000648389
ENSG00000157654	PALM2-AKAP2	0.676571692	0.338197534	1.000376676
ENSG00000168758	SEMA4C	3.914759074	1.95691559	1.000341994
ENSG00000137860	SLC28A2	0.221421113	0.110686968	1.000307421
ENSG00000175868	CALCB	0.054993605	0.027493324	1.000182509
ENSG00000239306	RBM14	12.67530116	6.3371625	1.00011111
ENSG00000131484	RP11-798G7.5	1.503939	0.751949	1.000039331
ENSG00000151704	KCNJ1	0.14268928	0.285483112	-1.000528451
ENSG00000154124	OTULIN	3.225441578	6.457121047	-1.001394387
ENSG00000236673	RP11-69I8.2	0.150479314	0.301367157	-1.00195702
ENSG00000196511	TPK1	0.391703736	0.784549186	-1.002101008
ENSG00000206885	SNORA75	0.921332	1.845402	-1.002142098
ENSG00000254929	RP11-144G6.12	1.560687669	3.12662503	-1.002424362
ENSG00000162971	TYW5	1.410495948	2.826854594	-1.002995155
ENSG00000129991	TNNI3	4.198202294	8.417680004	-1.003650981

ENSG00000218416	PP14571	0.066078101	0.132499451	-1.003742252
ENSG00000223855	HRAT92	0.043551	0.087332	-1.003804534
ENSG00000180245	RRH	0.24906	0.499456	-1.00386425
ENSG00000170165	CR848007.2	0.329807	0.661448	-1.004005723
ENSG00000260777	CTD-2008P7.1	0.039437	0.079123	-1.004547319
ENSG00000239127	SNORD125	1.314819	2.638376	-1.004785971
ENSG00000140199	SLC12A6	2.254870021	4.52548263	-1.005027386
ENSG00000228107	AP000692.9	0.923558	1.853745	-1.00516833
ENSG00000259563	CTD-2329K10.1	0.285369	0.572972	-1.005636018
ENSG00000197860	SGTB	6.937767569	13.93255371	-1.005916302
ENSG00000235837	AC073333.8	0.219377	0.440931	-1.007140632
ENSG00000227288	RP5-837I24.1	0.902458	1.814034	-1.0072698
ENSG00000257595	RP3-473L9.4	0.610130093	1.226527586	-1.007390887
ENSG00000053702	NRIP2	0.720698133	1.449085655	-1.00767586
ENSG00000273011	RP11-728K20.3	0.07939	0.159648	-1.007865278
ENSG00000172340	SUCLG2	12.34609103	24.830237	-1.008043698
ENSG00000250222	CTC-338M12.5	0.27418037	0.551538383	-1.008336003
ENSG00000254064	CTD-2530N21.4	2.706887	5.447309	-1.008909044
ENSG00000228283	KATNBL1P6	1.24914352	2.514181576	-1.009149602
ENSG00000187066	TMEM262	1.025083812	2.06340425	-1.009284622
ENSG00000108771	DHX58	0.486433087	0.979186267	-1.009341959
ENSG00000269947	RP11-849F2.9	0.735503	1.480687	-1.009463573
ENSG00000181984	GOLGA8CP	0.052337	0.105385	-1.0097664
ENSG00000108474	PIGL	1.298517914	2.615119146	-1.010010761
ENSG00000162601	MYSM1	9.428241494	18.99345142	-1.010441473
ENSG00000206948	SNORA36A	0.107962	0.217582	-1.011035603
ENSG00000078177	N4BP2	1.27450064	2.569345817	-1.011468983
ENSG00000013563	DNASE1L1	2.727501757	5.499520263	-1.011725648
ENSG00000198521	ZNF43	0.015299992	0.030869855	-1.012667795
ENSG00000197588	KLKP1	0.079857937	0.161125429	-1.012676501
ENSG00000166126	AMN	0.151187312	0.305056646	-1.012740091
ENSG00000064692	SNCAIP	0.038354422	0.077391136	-1.012775404
ENSG00000270020	RP11-463O9.9	0.158408	0.319642	-1.01281179
ENSG00000128245	YWHAH	54.21560603	109.4150673	-1.013031325
ENSG00000088970	KIZ	3.203514423	6.466550642	-1.013340875
ENSG00000172915	NBEA	0.981514264	1.981568086	-1.0135614
ENSG00000111291	GPRC5D	0.530663648	1.071416397	-1.013649653
ENSG00000154274	C4orf19	0.093340954	0.188462417	-1.013694734
ENSG00000279649	RP11-96D1.8	0.461905	0.9327	-1.013816954
ENSG00000135605	TEC	1.685944179	3.404776756	-1.014003437
ENSG00000230022	FNTAP2	0.576395	1.16436	-1.014407458
ENSG00000279516	FAM230C	0.008612	0.017399	-1.014584166
ENSG00000240718	RN7SL851P	0.167975	0.339369	-1.014608256
ENSG00000200075	SNORA25	0.400758	0.809677	-1.014615176
ENSG00000131386	GALNT15	0.008962277	0.018107071	-1.014616029
ENSG00000265355	MIR3136	0.652516	1.318321	-1.014616514
ENSG00000265569	AC011453.1	0.503923	1.018109	-1.014616816
ENSG00000272689	RP4-539M6.21	0.153765	0.310662	-1.014618628

ENSG00000277693	AP003900.6	0.018301241	0.036975728	-1.014637112
ENSG00000229349	ACTG1P9	1.942813	3.925497	-1.014728277
ENSG00000258758	RP11-613G13.1	0.214267	0.432964	-1.0148374
ENSG00000198929	NOS1AP	0.743656247	1.502740584	-1.014888181
ENSG00000119508	NR4A3	0.346499296	0.700463843	-1.015458159
ENSG00000280436	bP-2171C21.2	1.43341	2.898278	-1.015744659
ENSG00000102128	RAB40AL	0.132812	0.268549	-1.015799845
ENSG00000250116	RP11-417F21.1	0.759826	1.53695	-1.016329247
ENSG00000126458	RRAS	34.3400238	69.47155171	-1.016531282
ENSG00000275207	MIR6740	0.139627	0.282576	-1.017060993
ENSG00000238862	SNORD19B	3.606769	7.303686	-1.017917715
ENSG00000224888	RP5-1142A6.2	0.123568495	0.250266156	-1.018152243
ENSG00000177410	ZFAS1	49.10052538	99.52752963	-1.019357173
ENSG00000207241	SNORD45A	0.477706	0.96875	-1.020001408
ENSG00000140743	CDR2	7.574223427	15.36838213	-1.020795413
ENSG00000268201	CTD-3138B18.6	0.549679	1.115341	-1.020823593
ENSG00000174226	SNX31	0.129301088	0.262405745	-1.021064893
ENSG00000271580	RP11-536L3.4	0.75825	1.539398	-1.02162078
ENSG00000262815	RP11-565F19.4	0.099213	0.201492	-1.022121483
ENSG00000275413	CTC-529I10.1	4.880454	9.911742	-1.022123276
ENSG00000260362	RP11-297M9.1	0.343164	0.697006	-1.022272862
ENSG00000179862	CITED4	2.508456	5.096214	-1.022626228
ENSG00000267882	RP4-569M23.5	0.076612	0.155659	-1.022746705
ENSG00000243753	HLA-L	0.341316348	0.69358295	-1.022958918
ENSG00000133315	MACROD1	4.062529809	8.255888423	-1.023045074
ENSG00000120129	DUSP1	14.865063	30.211092	-1.023152755
ENSG00000237742	RP11-624M8.1	0.004380981	0.008906648	-1.023628661
ENSG00000063180	CA11	3.890283169	7.909242426	-1.023664344
ENSG00000181264	TMEM136	1.476081286	3.005550087	-1.025856891
ENSG00000275515	RP11-295D4.7	0.388562	0.79144	-1.026335165
ENSG00000167972	ABCA3	1.334461604	2.718231727	-1.026410654
ENSG00000198961	PJA2	22.23204725	45.2950833	-1.026713651
ENSG00000135406	PRPH	0.883079104	1.799179202	-1.026724307
ENSG00000224886	RP11-119F19.4	0.481342	0.980843	-1.026959915
ENSG00000174776	WDR49	0.026160327	0.053328667	-1.027530689
ENSG00000205702	CYP2D7	0.404439127	0.824687263	-1.027924548
ENSG00000276430	FAM25C	0.053183	0.108473	-1.028298921
ENSG00000274231	RP11-131M6.1	8.520402	17.380562	-1.028481328
ENSG00000260751	CTD-2196E14.6	0.994066	2.028462	-1.02897273
ENSG00000278610	RP11-455P21.3	0.099177	0.202438	-1.029402635
ENSG00000183644	C11orf88	0.007758838	0.015839858	-1.029646951
ENSG00000233872	AC005033.6	0.062603	0.127812	-1.029719595
ENSG00000254933	RP11-619A14.2	0.083028	0.169513	-1.029726066
ENSG00000254888	RP11-716D19.1	0.098636	0.201379	-1.029727045
ENSG00000274723	RP11-618L22.1	0.097577	0.199217	-1.02972773
ENSG00000278703	RP11-706P11.2	0.105813	0.216032	-1.029728144
ENSG00000277592	Metazoa_SRP	0.18374	0.375131	-1.029728755
ENSG00000225343	RPL21P66	0.109219	0.222986	-1.029729282

ENSG00000168672	FAM84B	7.583871693	15.48470089	-1.029837054
ENSG00000214189	ZNF788	0.013487449	0.027543406	-1.030089494
ENSG00000261669	CTD-2515A14.1	0.171079	0.349483	-1.0305596
ENSG00000223760	MED15P9	0.018230872	0.037249797	-1.030848971
ENSG00000100852	ARHGAP5	5.296453229	10.82971005	-1.03189613
ENSG00000235514	LLPHP2	0.066834	0.136668	-1.032021357
ENSG00000198092	TMPRSS11F	0.012675	0.025919	-1.03202431
ENSG00000235859	AC006978.6	0.871917	1.783259	-1.032253542
ENSG00000213846	AC098614.2	8.150647225	16.67346595	-1.032567501
ENSG00000235251	RP11-384B12.2	0.677349	1.386225	-1.033190172
ENSG00000185753	CXorf38	1.512974973	3.097616522	-1.033770429
ENSG00000269893	SNHG8	22.6631635	46.40210391	-1.033840962
ENSG00000235397	EPN2-AS1	0.470469	0.963398	-1.034032264
ENSG00000167103	PIP5KL1	1.389623984	2.848472372	-1.035493854
ENSG00000243918	EIF4BP8	3.219543	6.603238	-1.036317727
ENSG00000198900	TOP1	85.719299	175.861404	-1.036746935
ENSG00000186395	KRT10	4.528895	9.291739	-1.036789539
ENSG00000213931	HBE1	53.48562189	109.7355205	-1.036807569
ENSG00000180596	HIST1H2BC	0.452885121	0.929408107	-1.037167086
ENSG00000273416	RP4-597N16.4	0.205953	0.422896	-1.037987773
ENSG00000183826	BTBD9	0.812623459	1.6689249	-1.038260117
ENSG00000165475	CRYL1	4.941288238	10.15179107	-1.038775163
ENSG00000153291	SLC25A27	1.87777162	3.858514187	-1.039023801
ENSG00000150961	SEC24D	5.901022006	12.12768479	-1.039267419
ENSG00000148516	ZEB1	0.94175424	1.935845824	-1.039541528
ENSG00000143248	RGS5	0.981622372	2.017956138	-1.039654782
ENSG00000132681	ATP1A4	0.012148698	0.024976652	-1.039778354
ENSG00000256950	RP11-87C12.2	0.083390425	0.171475594	-1.040049606
ENSG00000068137	PLEKHH3	4.714580677	9.696326782	-1.04030886
ENSG00000276664	TUG1_1	2.388361	4.913284	-1.040666717
ENSG00000123552	USP45	3.514783492	7.234249756	-1.041407584
ENSG00000240038	AMY2B	0.098712034	0.203223103	-1.041766532
ENSG00000145220	LYAR	16.70686001	34.41170005	-1.042458558
ENSG00000033327	GAB2	1.521734102	3.134512594	-1.042524832
ENSG00000196182	STK40	6.562260334	13.52337567	-1.043190585
ENSG00000272402	RP1-30M3.6	0.932468	1.921613	-1.043191695
ENSG00000163803	PLB1	0.128260305	0.264323515	-1.04323003
ENSG00000275227	uc_338	0.427416	0.880861	-1.043273464
ENSG00000112245	PTP4A1	57.02873257	117.5617312	-1.043657635
ENSG00000131480	AOC2	3.062546	6.316601933	-1.044417144
ENSG00000204385	SLC44A4	0.015107386	0.0311647	-1.044658806
ENSG00000106302	HYAL4	0.018588756	0.038346728	-1.04467321
ENSG00000258343	RP11-536G4.2	0.110164997	0.227259305	-1.044673465
ENSG00000275812	RP4-697K14.15	0.155171	0.320103	-1.044677241
ENSG00000251733	SCARNA8	0.38852	0.80148	-1.044677653
ENSG00000254438	RP11-231N3.1	0.074409	0.1535	-1.04468962
ENSG00000256614	AK6P1	0.053238	0.109826	-1.044691356
ENSG00000228133	AC099684.1	0.024694493	0.050943571	-1.044710719

ENSG00000147421	HMBOX1	2.403602464	4.959104721	-1.044881385
ENSG00000155792	DEPTOR	2.686545946	5.543182108	-1.044961893
ENSG00000120915	EPHX2	2.116665711	4.367642519	-1.04506134
ENSG00000207168	SNORA15	0.244276	0.504252	-1.045632774
ENSG00000235010	RP11-140A10.3	0.063779515	0.131674051	-1.045806033
ENSG00000243244	STON1	0.414290437	0.855370351	-1.045906682
ENSG00000225602	MTOR-AS1	0.057366488	0.118455708	-1.046067624
ENSG00000214837	LINC01347	1.032191788	2.132116	-1.046574874
ENSG00000212579	SNORA40	2.896179	5.983138	-1.046751567
ENSG00000188738	FSIP2	0.012851539	0.026558291	-1.047221155
ENSG00000278419	RP11-298E9.7	1.019825	2.108097	-1.047619642
ENSG00000201754	SNORD52	1.528556	3.160304	-1.047893935
ENSG00000239388	ASB14	0.534466424	1.105150039	-1.048071024
ENSG00000239412	RPL21P71	0.539498	1.11606	-1.048725073
ENSG00000240531	RPL21P123	0.142917	0.295962	-1.050234418
ENSG00000229153	EPHA1-AS1	0.041488175	0.085924283	-1.050365719
ENSG00000165813	CCDC186	6.489298255	13.44023983	-1.050424501
ENSG00000202150	RNU6-407P	0.968985	2.008704	-1.051718748
ENSG00000260877	RP11-211G23.2	2.125619	4.407135	-1.051958062
ENSG00000261798	RP1-118J21.25	0.085111	0.17648	-1.052087189
ENSG00000212135	SNORD67	0.917049	1.901533	-1.052092249
ENSG00000259687	LINC01220	0.141772152	0.293969561	-1.052092608
ENSG00000266374	AC026512.1	1.41684	2.939619	-1.052952334
ENSG00000270898	GPR75-ASB3	1.072147962	2.22485261	-1.053205747
ENSG00000262372	RP11-669E14.6	0.608085	1.262022	-1.053392153
ENSG00000134152	KATNBL1	2.41241244	5.009505795	-1.054191704
ENSG00000281404	LINC01176	0.12444045	0.258420856	-1.054266991
ENSG00000237745	AC009963.5	1.138855	2.36535	-1.054469601
ENSG00000206113	CFAP99	0.029374435	0.061012812	-1.054551138
ENSG00000230223	ATXN8OS	0.166702055	0.346493308	-1.055555598
ENSG00000274372	RP11-94I2.4	1.359210694	2.825638927	-1.055808015
ENSG00000130303	BST2	1.123446134	2.335550746	-1.055831839
ENSG00000224462	C4BPAP1	1.310478	2.726557	-1.056987182
ENSG00000091592	NLRP1	0.808437664	1.682446723	-1.057352378
ENSG00000206828	U1	0.291722	0.607206	-1.057591856
ENSG00000176912	TYMSOS	0.404328299	0.841973881	-1.058248298
ENSG00000270926	RP11-551L14.7	0.1213	0.252623	-1.058406444
ENSG00000167615	LENG8	13.30603003	27.71592752	-1.058635094
ENSG00000205744	DENND1C	1.75674175	3.65966494	-1.058809445
ENSG00000215483	LINC00598	0.040078862	0.083496511	-1.058874363
ENSG00000147082	CCNB3	0.142844433	0.297622805	-1.059040266
ENSG00000237380	HOXD-AS2	0.067492272	0.140624666	-1.059055442
ENSG00000271581	XXbac-BPG248L24.12	0.555956	1.158489	-1.059201732
ENSG00000264049	MIR4737	0.301433	0.628163	-1.0593016
ENSG00000105664	COMP	0.016413097	0.034206319	-1.059415365
ENSG00000226812	RP5-881L22.5	0.056177161	0.117081386	-1.059456107
ENSG00000241818	RP11-1000B6.2	0.067862016	0.141434867	-1.059461624
ENSG00000229221	HNRNPA1P66	0.027247	0.056787	-1.059463305

ENSG00000167910	CYP7A1	0.017253	0.035958	-1.059465536
ENSG00000232082	RPS6KA2-IT1	0.043952	0.091603	-1.059466032
ENSG00000255381	AP001258.5	0.10387	0.216482	-1.059468042
ENSG00000278246	AC004556.2	0.450409	0.938728	-1.059471537
ENSG00000214380	RP11-457K10.2	0.082356	0.171644	-1.059473762
ENSG00000265702	RP11-156L14.1	0.013076962	0.02725465	-1.05947501
ENSG00000118004	COLEC11	0.01832099	0.038184116	-1.059475149
ENSG00000224294	RP3-326I13.1	0.022844	0.047611	-1.059479641
ENSG00000127129	EDN2	0.062106031	0.129440313	-1.059481735
ENSG00000237489	LINC00959	0.017902	0.037312	-1.05951892
ENSG00000174456	C12orf76	1.416747439	2.952918781	-1.059559082
ENSG00000176771	NCKAP5	0.005328202	0.011105589	-1.059565154
ENSG00000254263	RP11-473O4.4	0.639999	1.334154	-1.059783649
ENSG00000174010	KLHL15	4.003728	8.346814	-1.059881657
ENSG00000275143	SCARNA16	2.017903	4.207	-1.059934992
ENSG00000272017	RP1-199J3.7	0.154902	0.323066	-1.060473156
ENSG00000140057	AK7	0.496487794	1.035591789	-1.060625275
ENSG00000265490	RP11-806L2.6	0.278546	0.581049	-1.060744233
ENSG00000172840	PDP2	2.512773999	5.243972642	-1.061379239
ENSG00000179988	PSTK	0.743621648	1.553286328	-1.062683122
ENSG00000214510	SPINK13	0.173411811	0.362339915	-1.063141577
ENSG00000069702	TGFBR3	2.502182448	5.228996362	-1.063347078
ENSG00000268069	RP5-1057I20.4	0.52505	1.097495	-1.063687646
ENSG00000119929	CUTC	14.64517266	30.61501202	-1.063814047
ENSG00000108786	HSD17B1	0.247863146	0.518235353	-1.064063657
ENSG00000156976	EIF4A2	40.34585619	84.3856593	-1.064577339
ENSG00000246982	RP1-179N16.6	0.467363008	0.977667194	-1.064799896
ENSG00000174738	NR1D2	14.0881866	29.47389186	-1.06495165
ENSG00000104826	LHB	0.755171159	1.580226463	-1.065255754
ENSG00000146166	LGSN	0.039973495	0.08364882	-1.065301468
ENSG00000231084	CTA-253N17.1	0.257189	0.538326	-1.065651166
ENSG00000169031	COL4A3	0.414856026	0.868578619	-1.066045696
ENSG00000261535	CTA-305I2.1	0.063541	0.133063	-1.066349765
ENSG00000280339	RP11-736K20.4	0.031331	0.065631	-1.066786598
ENSG00000213626	LBH	0.022446019	0.047019793	-1.066808567
ENSG00000270890	RP3-468K18.6	2.053678	4.302065	-1.066819326
ENSG00000267336	EIF4A2P1	2.435166	5.103012	-1.067328913
ENSG00000277948	uc_338	0.139017	0.291344	-1.067462283
ENSG00000091409	ITGA6	32.22665832	67.55390746	-1.067784622
ENSG00000184731	FAM110C	5.407135015	11.33552891	-1.067915422
ENSG00000218226	TATDN2P2	1.203685	2.524691	-1.068648932
ENSG00000231240	KLF2P1	0.326827	0.685676	-1.068999853
ENSG00000116711	PLA2G4A	5.061827	10.623062	-1.069469564
ENSG00000152465	NMT2	6.982487857	14.65526135	-1.069605631
ENSG00000198315	ZKSCAN8	4.993023087	10.48008335	-1.069664708
ENSG00000238120	LINC01589	0.371506	0.780205	-1.070467733
ENSG00000249119	MTND6P4	209.383286	439.942505	-1.07116871
ENSG00000232530	RP1-102K2.6	0.251962	0.529426	-1.071222879

ENSG00000273771	RP11-236L14.2	0.218361	0.458957	-1.071643784
ENSG00000050405	LIMA1	14.62787842	30.74535949	-1.071648135
ENSG00000185522	LMNTD2	0.39245471	0.824948432	-1.071777764
ENSG00000115339	GALNT3	7.693300637	16.17447394	-1.072044199
ENSG00000227197	AC009518.8	0.0637	0.133952	-1.072350844
ENSG00000253167	KIAA0196-AS1	0.504565	1.061032	-1.072356128
ENSG00000008405	CRY1	3.985854516	8.381816051	-1.072373807
ENSG00000237765	FAM200B	1.277971815	2.688262877	-1.072818203
ENSG00000115159	GPD2	4.693913914	9.879781565	-1.073687762
ENSG00000271332	RP11-797J4.1	0.341585	0.719186	-1.074120314
ENSG00000275850	RP11-543P15.3	0.021206	0.044649	-1.074155347
ENSG00000107187	LHX3	0.016476229	0.034690568	-1.074157354
ENSG00000169253	RP11-220D10.1	2.408767	5.071672	-1.074166597
ENSG00000215039	CD27-AS1	0.509253836	1.072326068	-1.074286812
ENSG00000228857	AC104653.1	0.07736876	0.162926939	-1.074402106
ENSG00000226605	AC007098.1	0.305854	0.644229	-1.074730461
ENSG00000264026	RP11-1124B17.1	0.027767	0.058489	-1.074794009
ENSG00000105376	ICAM5	6.106380532	12.87193144	-1.075839145
ENSG00000204634	TBC1D8	10.04181501	21.18433789	-1.076977986
ENSG00000144821	MYH15	1.25956735	2.657255853	-1.077008878
ENSG00000188305	C19orf35	0.049828	0.105129	-1.077132121
ENSG00000168477	TNXB	0.392268092	0.827791995	-1.07742831
ENSG00000174938	SEZ6L2	6.196429503	13.07636157	-1.077452122
ENSG00000197124	ZNF682	0.004268954	0.009013585	-1.078218401
ENSG00000164713	BRI3	4.415310614	9.324383157	-1.078493355
ENSG00000105835	NAMPT	11.16895576	23.59102399	-1.078743734
ENSG00000213036	RP11-365D23.4	113.231384	239.275528	-1.079398971
ENSG00000232454	RP11-3J10.7	9.119267	19.276268	-1.079835993
ENSG00000170325	PRDM10	2.46814057	5.217578947	-1.079955961
ENSG00000219926	RP11-394A14.2	0.050108647	0.105957362	-1.080352347
ENSG00000275383	RP11-615I2.6	0.088311	0.186745	-1.08040456
ENSG00000179344	HLA-DQB1	0.775309507	1.639834692	-1.080706126
ENSG00000237872	POU5F1P4	0.072709	0.153799	-1.080840264
ENSG00000201684	RN7SKP239	0.324355	0.686138	-1.08092509
ENSG00000121060	TRIM25	8.189797195	17.32880048	-1.081272161
ENSG00000275293	Metazoa_SRP	0.318101	0.673124	-1.081387389
ENSG00000230310	CTD-2192J16.11	0.093731	0.198342	-1.081392029
ENSG00000215717	TMEM167B	5.967567546	12.62921851	-1.081550473
ENSG00000176142	TMEM39A	5.571782821	11.79226543	-1.081629973
ENSG00000204194	RPL12P1	7.734663	16.377026	-1.082263054
ENSG00000147251	DOCK11	0.366746031	0.776943022	-1.083027449
ENSG00000241860	RP11-34P13.13	3.597757788	7.622222473	-1.083113654
ENSG00000245080	RP11-320N21.1	0.052907213	0.112122653	-1.083541463
ENSG00000152583	SPARCL1	0.043328183	0.091834944	-1.08373748
ENSG00000143507	DUSP10	2.113398091	4.479584953	-1.083800522
ENSG00000225527	RP11-383B4.4	0.369706	0.783634	-1.083801537
ENSG00000235146	RP5-857K21.2	0.126639318	0.268471798	-1.084045156
ENSG00000229591	RP5-981O7.2	0.302504	0.641727	-1.085005465

ENSG00000122203	KIAA1191	14.98534807	31.79664596	-1.085321998
ENSG00000236194	AC003104.1	0.187119	0.397222	-1.085989471
ENSG00000232611	RP11-1114A5.4	0.818235	1.737158	-1.086141823
ENSG00000107864	CPEB3	0.653150465	1.386906726	-1.08638348
ENSG00000279356	RP11-429P3.8	0.167658	0.356049	-1.086554476
ENSG00000172361	CFAP53	2.115291	4.492661	-1.086714057
ENSG00000253948	RP11-410L14.2	1.06882	2.271638	-1.087714041
ENSG00000188388	GOLGA6L3	0.827235	1.758184	-1.087716929
ENSG00000118564	FBXL5	2.985784946	6.34608642	-1.08775691
ENSG00000120051	CFAP58	0.31501749	0.669643393	-1.087961089
ENSG00000242808	SOX2-OT	0.00840132	0.017860549	-1.088088508
ENSG00000274304	MIR7108	0.002926	0.006221	-1.088216737
ENSG00000278648	MIR675	0.110672	0.235302	-1.088223316
ENSG00000157766	ACAN	0.002333183	0.004961357	-1.088435256
ENSG00000131721	RHOXF2	0.008776	0.018662	-1.088468177
ENSG00000232771	PPP1R26P5	0.005557	0.011817	-1.088485677
ENSG00000224812	TMEM72-AS1	0.015424	0.0328	-1.088518858
ENSG00000077092	RARB	0.01376747	0.029277562	-1.088531992
ENSG00000064201	TSPAN32	0.01341922	0.028537319	-1.088549016
ENSG00000227039	ITGB2-AS1	0.020876837	0.044396975	-1.088558236
ENSG00000261239	ANKRD26P1	0.007939245	0.016883758	-1.088562276
ENSG00000279220	GPR1-AS	0.006163237	0.013106868	-1.088562951
ENSG00000279930	LA16c-312E8.4	0.023542	0.050065	-1.088565491
ENSG00000132874	SLC14A2	0.009409522	0.020010589	-1.088570307
ENSG00000280321	RP11-1110F20.1	0.030772	0.065441	-1.088576581
ENSG00000090104	RGS1	0.012362838	0.026291374	-1.088579565
ENSG00000279176	RP11-43D4.3	0.037016	0.07872	-1.088581217
ENSG00000185155	MIXL1	0.025084734	0.053346439	-1.088582343
ENSG00000274565	CTD-3035K23.7	0.044105	0.093796	-1.088584182
ENSG00000261451	RP11-981G7.1	0.010967	0.023323	-1.088584439
ENSG00000094755	GABRP	0.011014328	0.023423684	-1.088586597
ENSG00000203446	AC004988.1	0.028739	0.061118	-1.088587471
ENSG00000281904	CH17-132F21.5	0.025928	0.05514	-1.088588342
ENSG00000142515	KLK3	0.010971669	0.023333021	-1.088590071
ENSG00000276715	YWHAEP7	0.016977005	0.036104386	-1.088592178
ENSG00000280083	RP11-317J9.1	0.026061	0.055423	-1.088592361
ENSG00000268324	LRRC2-AS1	0.02215797	0.047122589	-1.088593109
ENSG00000241434	CTD-2224J9.4	0.042133	0.089603	-1.088596392
ENSG00000225893	RP11-6J24.3	0.034861	0.074138	-1.088599242
ENSG00000233684	AC079779.6	0.033818528	0.07192101	-1.088599404
ENSG00000179593	ALOX15B	0.016808412	0.035746071	-1.088601248
ENSG00000256124	LINC01152	0.025328216	0.053864969	-1.088601871
ENSG00000203797	DDO	0.023895	0.050817	-1.08860244
ENSG00000246016	LINC01513	0.105376	0.224101	-1.088602764
ENSG00000232814	COL4A2-AS1	0.094253	0.200446	-1.088603184
ENSG00000241228	RPL12P31	0.037701	0.080178	-1.08860364
ENSG00000251513	CTD-2215E18.1	0.028386	0.060368	-1.088604439
ENSG00000233261	LINC00264	0.023326	0.049607	-1.088604781

ENSG00000272817	RP11-402D21.2	0.089136	0.189564	-1.088604883
ENSG00000198768	APCDD1L	0.012911379	0.027458408	-1.088604883
ENSG00000234880	LINC00163	0.023618	0.050228	-1.08860503
ENSG00000125910	S1PR4	0.030587097	0.065049075	-1.088605326
ENSG00000276136	RP11-50I19.2	0.120039	0.255285	-1.088605565
ENSG00000228725	MTND2P12	0.040652	0.086454	-1.088606383
ENSG00000214919	RP11-674E16.4	0.032111	0.06829	-1.08860674
ENSG00000197647	ZNF433	0.008265052	0.017577184	-1.088608232
ENSG00000151322	NPAS3	0.005507616	0.011712982	-1.088608514
ENSG00000283045	RP11-764D10.2	0.153534	0.326519	-1.088608769
ENSG00000235076	GAPDHP52	0.05403	0.114905	-1.08860899
ENSG00000235007	RP11-344B5.4	0.052688	0.112051	-1.088609202
ENSG00000164430	MB21D1	0.01224936	0.026050588	-1.088609597
ENSG00000103490	PYCARD	0.030641963	0.06516596	-1.088609855
ENSG00000224511	LINC00365	0.016976831	0.036104473	-1.08861044
ENSG00000236053	LINC01067	0.076651	0.163013	-1.088610503
ENSG00000258983	RP11-507K2.2	0.056239	0.119603	-1.08861073
ENSG00000243107	AC000120.7	0.064061	0.136238	-1.088610937
ENSG00000256615	RP11-59N23.3	0.088056	0.187268	-1.08861118
ENSG00000279122	RP11-394B2.3	0.040555	0.086248	-1.088611208
ENSG00000248408	RP11-452C8.1	0.034343	0.073037	-1.088611438
ENSG00000275092	LA16c-329F2.2	0.140987	0.299836	-1.08861147
ENSG00000244429	RP11-12N13.1	0.085111	0.181005	-1.088612043
ENSG00000265625	RP11-68I3.11	0.096761	0.205781	-1.088612198
ENSG00000249012	RP11-731D1.1	0.104725	0.222718	-1.088612277
ENSG00000238260	RP11-46F15.2	0.081827	0.174021	-1.088612549
ENSG00000217767	NDUFAB1P1	0.110644	0.235306	-1.088612889
ENSG00000232104	RFX3-AS1	0.13033641	0.277185762	-1.088612993
ENSG00000241782	RP11-91P24.1	0.107376	0.228356	-1.088613129
ENSG00000226862	RP11-569A11.1	0.16209	0.344716	-1.088613175
ENSG00000229330	AC006947.1	0.105158	0.223639	-1.08861319
ENSG00000227007	LINC01247	0.006046	0.012858	-1.088613372
ENSG00000259665	RP11-323I15.3	0.068779	0.146272	-1.088613584
ENSG00000235112	HSPE1P27	0.171947	0.365679	-1.088613834
ENSG00000262136	CTD-2033A16.3	0.171947	0.365679	-1.088613834
ENSG00000233754	AP001628.7	0.064919	0.138063	-1.088614056
ENSG00000242757	CTD-2090I13.3	0.112852	0.240002	-1.088614442
ENSG00000236426	RP11-79M19.2	0.016698352	0.035512335	-1.088614451
ENSG00000124194	GDAP1L1	0.015035771	0.031976529	-1.08861451
ENSG00000256915	RP11-221N13.4	0.036302753	0.077204964	-1.08861465
ENSG00000243491	RP11-521D12.5	0.026141	0.055594	-1.088614857
ENSG00000279600	RP11-637C24.5	0.117003	0.24883	-1.088614912
ENSG00000214283	RAD51AP1P1	0.076268	0.162199	-1.088615152
ENSG00000276945	CTC-436P18.5	0.09585	0.203844	-1.088615156
ENSG00000259232	RP11-349G13.1	0.085684	0.182224	-1.088615247
ENSG00000246394	RP11-386I8.6	0.156483802	0.332793823	-1.088615324
ENSG00000271664	RP4-800G7.3	0.092961	0.1977	-1.088615378
ENSG00000259121	RP11-545M17.2	0.253215	0.538512	-1.088615624

ENSG00000228707	RP11-787B4.2	0.099993	0.212655	-1.088615769
ENSG00000276445	LLNLR-268E12.1	0.087151	0.185344	-1.088616289
ENSG00000266863	RN7SL123P	0.087151	0.185344	-1.088616289
ENSG00000152670	DDX4	0.013292461	0.028269075	-1.088616474
ENSG00000273123	RP11-9N20.3	0.092707	0.19716	-1.088616705
ENSG00000228040	RP3-522D1.2	0.326258	0.693853	-1.088616767
ENSG00000187492	CDHR4	0.024136833	0.051331815	-1.088616868
ENSG00000268392	AC003682.16	0.110165	0.234288	-1.088617118
ENSG00000187242	KRT12	0.054522	0.115952	-1.088617315
ENSG00000239919	SNRPGP3	0.112106	0.238416	-1.088617563
ENSG00000275479	RP11-334C17.6	0.295908	0.629308	-1.088617582
ENSG00000149124	GLYAT	0.032688535	0.069518764	-1.088617718
ENSG00000260070	RP11-182J23.1	0.072193	0.153533	-1.088617916
ENSG00000257779	RP11-848D3.4	0.233469	0.496519	-1.088617924
ENSG00000231855	GLUDP5	0.424135	0.902009	-1.088618288
ENSG00000201013	Y_RNA	0.450409	0.957886	-1.088618313
ENSG00000228509	AC006460.2	0.045240818	0.096213767	-1.088618334
ENSG00000231105	RP5-1071N3.1	0.061593	0.13099	-1.088618374
ENSG00000231272	RP4-799P18.4	0.112602	0.239471	-1.088618503
ENSG00000227368	AC079753.5	0.232615	0.494703	-1.088618517
ENSG00000270409	RP11-44D5.1	0.041752609	0.088795396	-1.088618539
ENSG00000248654	MTCO3P44	0.094078	0.200076	-1.088618825
ENSG00000277555	UPF3BP4	0.341585	0.72645	-1.08861888
ENSG00000278389	RP11-626G11.6	0.226205	0.481071	-1.088619014
ENSG00000232197	RP11-302L19.1	0.118639	0.25231	-1.088619044
ENSG00000256955	RP11-417L19.2	0.066099	0.140573	-1.08861917
ENSG00000258649	CTD-2142D14.1	0.070929	0.150845	-1.088619364
ENSG00000228598	MACC1-AS1	0.07965	0.169392	-1.088619474
ENSG00000234690	AC073283.4	0.014328904	0.030473345	-1.088619559
ENSG00000272861	RP11-332H14.1	0.130169	0.276831	-1.088619599
ENSG00000281756	C2-AS1	0.066793	0.142049	-1.088619856
ENSG00000211750	TRBV24-1	0.066793	0.142049	-1.088619856
ENSG00000244061	RP11-389C8.1	0.084827	0.180402	-1.088619887
ENSG00000212195	U3	0.259674	0.55225	-1.088619943
ENSG00000237085	AC127391.3	0.259674	0.55225	-1.088619943
ENSG00000185742	C11orf87	0.00865	0.018396	-1.088620065
ENSG00000249776	RP11-133F8.2	0.066880566	0.142235272	-1.088620304
ENSG00000248443	RP11-284A20.3	0.062144	0.132162	-1.088620413
ENSG00000236031	RP11-269F20.1	0.067771	0.144129	-1.088620683
ENSG00000257494	RP3-521E19.2	0.067771	0.144129	-1.088620683
ENSG00000230661	YY1P1	0.076192	0.162038	-1.088620753
ENSG00000280335	RP11-15F12.6	0.071283	0.151598	-1.08862076
ENSG00000230433	RP1-20B11.2	0.119615	0.254386	-1.088620957
ENSG00000244280	ECEL1P2	0.059597295	0.126745972	-1.088621146
ENSG00000231651	DLG3-AS1	0.211187054	0.449133012	-1.08862137
ENSG00000248494	LNX1-AS2	0.057380225	0.122030949	-1.088621545
ENSG00000123560	PLP1	0.008373643	0.01780829	-1.088621754
ENSG00000251523	RP11-724M22.1	0.104082	0.221352	-1.088621819

ENSG00000236928	RP11-792A8.1	0.146534	0.311635	-1.088621824
ENSG00000233554	B4GALT1-AS1	0.060375	0.1284	-1.088622013
ENSG00000206532	RP11-553A10.1	0.074682568	0.158828041	-1.088622204
ENSG00000281920	RP11-418H16.1	0.068409	0.145486	-1.088622284
ENSG00000212126	TAS2R50	0.050896	0.108241	-1.088622891
ENSG00000270333	RP11-566F5.2	0.214751	0.456713	-1.088623006
ENSG00000188394	GPR21	0.043501	0.092514	-1.088623137
ENSG00000231447	RP1-14D6.7	0.060019	0.127643	-1.088623235
ENSG00000261366	MANEA-AS1	0.089764	0.190902	-1.088623346
ENSG00000239542	RN7SL399P	0.182423	0.387961	-1.088623995
ENSG00000233339	AC018878.3	0.117543	0.24998	-1.088624049
ENSG00000173702	MUC13	0.014545574	0.030934234	-1.088624111
ENSG00000196440	ARMCX4	0.003812177	0.008107402	-1.08862458
ENSG00000254479	SLC25A1P1	0.054904	0.116765	-1.08862473
ENSG00000269475	CH17-38B12.4	0.053294	0.113341	-1.088624812
ENSG00000150676	CCDC83	0.064725551	0.137652623	-1.088624863
ENSG00000256916	RP11-817J15.2	0.036354459	0.077315519	-1.088625679
ENSG00000224121	ATG12P2	0.122346	0.260195	-1.088626304
ENSG00000275431	CTB-186H2.3	0.012878257	0.027388375	-1.088626315
ENSG00000260460	RP11-284F21.8	0.013921	0.029606	-1.088626735
ENSG00000148541	FAM13C	0.007536627	0.016028263	-1.088627109
ENSG00000230987	RP11-113I24.1	0.007213	0.01534	-1.088627154
ENSG00000257121	RP11-129B9.2	0.098827	0.210177	-1.08862765
ENSG00000267338	KB-7G2.9	0.093216	0.198244	-1.088627691
ENSG00000230516	RP11-555J4.3	0.060663	0.129013	-1.088627695
ENSG00000249263	PARP4P3	0.064222	0.136582	-1.088627866
ENSG00000124915	DKFZP434K028	0.017617171	0.037466741	-1.088628209
ENSG00000270429	KNOP1P2	0.038645	0.082187	-1.088628446
ENSG00000259374	NDUFB4P11	0.02227396	0.04737044	-1.088628984
ENSG00000178732	GP5	0.014402	0.030629	-1.088629093
ENSG00000279597	RP4-566L20.1	0.058839	0.125134	-1.088629203
ENSG00000128284	APOL3	0.025874807	0.055028437	-1.088629258
ENSG00000232732	AC073043.1	0.007646586	0.016262154	-1.088630646
ENSG00000178201	VN1R1	0.02163	0.046001	-1.088631558
ENSG00000278007	Metazoa_SRP	0.105593	0.224567	-1.088631742
ENSG00000268603	RP11-316O14.1	0.080025	0.170191	-1.08863207
ENSG00000263011	RP11-473M20.11	0.050559	0.107525	-1.088632297
ENSG00000241388	HNF1A-AS1	0.012133769	0.025805174	-1.088632573
ENSG00000259584	RP11-521C20.2	0.058907	0.125279	-1.088633615
ENSG00000224238	WARS2-IT1	0.024707	0.052545	-1.088633644
ENSG00000154589	LY96	0.083848	0.178322	-1.088636425
ENSG00000280326	RP11-642A1.1	0.033528	0.071305	-1.088636817
ENSG00000249541	RP11-692C23.1	0.046311	0.098491	-1.088636989
ENSG00000185442	FAM174B	0.010928788	0.023242601	-1.088638033
ENSG00000179141	MTUS2-AS1	0.01982688	0.042166457	-1.088638175
ENSG00000272321	KB-1517D11.4	0.019411	0.041282	-1.088638424
ENSG00000258806	OR11H7	0.054144	0.11515	-1.088641032
ENSG00000233627	C4A-AS1	0.024294	0.051667	-1.088643068

ENSG00000229776	C4B-AS1	0.024294	0.051667	-1.088643068
ENSG00000248305	RP11-310A13.2	0.060446	0.128553	-1.088644501
ENSG00000147160	AWAT2	0.020748181	0.044126035	-1.088645259
ENSG00000240021	TEX35	0.012604324	0.026806154	-1.088645523
ENSG00000165702	GFI1B	0.017721141	0.037688336	-1.088646586
ENSG00000166527	CLEC4D	0.02528341	0.053771366	-1.08864708
ENSG00000171051	FPR1	0.019620457	0.041727843	-1.088651718
ENSG00000232118	BACH1-AS1	0.028950853	0.061571331	-1.088652889
ENSG00000277420	CH17-408M7.1	0.010027	0.021325	-1.088655714
ENSG00000224295	OLFM5P	0.034435	0.073235	-1.08865762
ENSG00000259436	CTC-378H22.2	0.026033	0.055366	-1.088658721
ENSG00000101098	RIMS4	0.009776363	0.020792012	-1.088659573
ENSG00000133710	SPINK5	0.011617251	0.024707289	-1.08866804
ENSG00000111339	ART4	0.019522465	0.041519914	-1.088668211
ENSG00000253955	CTB-33O18.3	0.016886217	0.035913205	-1.088668295
ENSG00000154227	CERS3	0.009920971	0.021099711	-1.088670047
ENSG00000275450	RP11-4L24.3	0.008148	0.017329	-1.088670518
ENSG00000279943	FLJ38576	0.020697	0.044018	-1.08867193
ENSG00000081818	PCDHB4	0.013306	0.028299	-1.088674135
ENSG00000185038	MROH2A	0.00888201	0.01889016	-1.08867672
ENSG00000279982	RP11-45A17.3	0.01637	0.034816	-1.08869614
ENSG00000082175	PGR	0.006608036	0.014054126	-1.088700203
ENSG00000282164	PEG13	0.011992	0.025505	-1.088707812
ENSG00000112195	TREML2	0.015413	0.032781	-1.088712169
ENSG00000203989	RHOXF2B	0.008885	0.018897	-1.088713536
ENSG00000197934	CYYR1-AS1	0.006853406	0.014576123	-1.088713986
ENSG00000144218	AFF3	0.007991632	0.016997006	-1.088718633
ENSG00000223731	SUPT20HL1	0.00972	0.020673	-1.088719544
ENSG00000251405	CTB-109A12.1	0.196748673	0.418459124	-1.088732797
ENSG00000147100	SLC16A2	0.012231	0.026014	-1.088745887
ENSG00000142149	HUNK	0.006135765	0.013050632	-1.08880458
ENSG00000145451	GLRA3	0.005301835	0.011277019	-1.088822254
ENSG00000229816	DDX50P1	0.5969	1.269878	-1.089128742
ENSG00000231064	RP11-263K19.4	0.205437392	0.43710082	-1.089267294
ENSG00000166987	MBD6	5.233222699	11.13656609	-1.089532894
ENSG00000164199	ADGRV1	0.310775762	0.661429225	-1.089712803
ENSG00000226416	MRPL23-AS1	0.37806	0.804858	-1.090119057
ENSG00000132406	TMEM128	3.240762891	6.900134808	-1.090291078
ENSG00000231442	LARP1BP1	0.372265	0.792892	-1.090794388
ENSG00000280402	RP11-20I23.10	0.282865	0.602604	-1.091096572
ENSG00000171435	KSR2	0.698073199	1.487204552	-1.091152862
ENSG00000105856	HBP1	2.970407891	6.329898844	-1.091521392
ENSG00000197302	ZNF720	0.521791133	1.11200701	-1.091621549
ENSG00000233723	LINC01122	0.006348955	0.013534521	-1.092052642
ENSG00000151718	WWC2	2.302071105	4.907558053	-1.092072939
ENSG00000227617	CERS6-AS1	0.018456462	0.03935166	-1.092298476
ENSG00000142046	TMEM91	0.609224287	1.299238122	-1.092620508
ENSG00000239704	CDRT4	0.791988701	1.689681445	-1.093199527

ENSG00000239474	KLHL41	0.23233545	0.495760881	-1.093435137
ENSG00000274810	NPHP3-ACAD11	0.346385913	0.739141599	-1.093470512
ENSG00000198077	CYP2A7	0.055828424	0.11915068	-1.093715439
ENSG00000189129	PLAC9	0.248927931	0.531375113	-1.094002541
ENSG00000159713	TPPP3	0.311053668	0.663999532	-1.094018703
ENSG00000152578	GRIA4	0.025036669	0.053459986	-1.094416837
ENSG00000236953	ZDHHC20-IT1	0.275836	0.589214	-1.094980951
ENSG00000199676	Y_RNA	0.900818	1.92535	-1.09581317
ENSG00000279568	RP11-20I23.5	0.378952	0.809948	-1.095814167
ENSG00000236184	TCEA1P4	0.718818	1.536407	-1.095862001
ENSG00000141682	PMAIP1	27.68366339	59.17566001	-1.095969024
ENSG00000007237	GAS7	0.627310205	1.340933717	-1.095986989
ENSG00000139364	TMEM132B	0.337794997	0.722216391	-1.096283201
ENSG00000157827	FMNL2	8.63003503	18.4537815	-1.096478159
ENSG00000242960	FTH1P23	60.713741	129.858658	-1.09684723
ENSG00000276811	MIR6125	0.062295	0.133251	-1.096958082
ENSG00000281207	SLFNL1-AS1	0.716638	1.533005	-1.097045952
ENSG00000279231	RP11-341D18.6	0.067323	0.144037	-1.097268085
ENSG00000240305	RP11-1072C15.1	0.148801	0.318394	-1.097428927
ENSG00000153234	NR4A2	2.452101418	5.248117113	-1.097781265
ENSG00000282980	RP11-807C20.2	0.692924	1.483062	-1.09780988
ENSG00000224276	RP11-336K24.5	0.185649	0.397394	-1.097992546
ENSG00000164751	PEX2	3.551588871	7.603218995	-1.098145759
ENSG00000279187	RP11-455O6.5	0.123036	0.263407	-1.09821318
ENSG00000081923	ATP8B1	2.500023606	5.352316127	-1.098221612
ENSG00000243016	RP11-305F5.2	0.220452	0.471982	-1.098267275
ENSG00000232677	LINC00665	0.03159371	0.067641609	-1.098273602
ENSG00000262768	RP11-353N14.1	0.248192	0.531398	-1.098336184
ENSG00000275582	RP4-681N20.5	1.921333	4.114725	-1.09868843
ENSG00000279159	RP3-394A18.1	0.370177	0.792906	-1.098934583
ENSG00000264608	RP11-192H23.8	0.891239	1.90958	-1.099371091
ENSG00000250295	RDH10-AS1	0.146758873	0.314532609	-1.099761863
ENSG00000245958	RP11-33B1.1	1.08240793	2.320275733	-1.100051947
ENSG00000064607	SUGP2	7.248258603	15.53863537	-1.100153476
ENSG00000185291	IL3RA	0.260584018	0.558683075	-1.100281513
ENSG00000280672	AC016747.1	0.057817	0.124018	-1.100983871
ENSG00000235310	GXYLT1P6	0.040734	0.08739	-1.101234711
ENSG00000134531	EMP1	2.954114823	6.338578061	-1.101433332
ENSG00000226845	EEF1GP3	0.128426	0.275645	-1.101874125
ENSG00000187735	TCEA1	13.37517747	28.72763777	-1.102881333
ENSG00000243504	RPS23P1	0.058907	0.126532	-1.102991302
ENSG00000280233	RP11-159D12.3	0.17345	0.372603	-1.10311945
ENSG00000146192	FGD2	0.009911793	0.02129264	-1.103136803
ENSG00000272256	RP11-489E7.4	0.102684	0.220603	-1.103241009
ENSG00000249077	RP11-478C1.8	0.161943	0.347918	-1.103261212
ENSG00000248608	RP11-206P5.2	0.059414	0.127645	-1.103262202
ENSG00000158615	PPP1R15B	24.164551	51.916317	-1.103295852
ENSG00000270165	RP11-167P11.2	0.251734	0.541004	-1.103739176

ENSG00000108309	RUNDC3A	0.17150019	0.36859796	-1.103837913
ENSG00000262663	RP11-497H17.1	0.749305	1.61073	-1.104089698
ENSG00000244218	RN7SL81P	0.717617	1.542746	-1.104214581
ENSG00000181722	ZBTB20	0.617552204	1.327786745	-1.10439045
ENSG00000158793	NIT1	2.846608672	6.125805848	-1.105655466
ENSG00000269179	CTC-326K19.6	0.263603	0.567544	-1.106365455
ENSG00000260866	RP11-626K17.5	0.078415	0.168843	-1.10648081
ENSG00000267439	AC002398.11	0.104392673	0.224962564	-1.107664493
ENSG00000170523	KRT83	0.03393	0.073123	-1.107763831
ENSG00000279953	RP11-338K17.5	0.106243	0.229001	-1.107986108
ENSG00000253896	AC144568.2	0.082732082	0.178359671	-1.108270643
ENSG00000250659	RP11-864I4.3	0.385577	0.831517	-1.108726763
ENSG00000215158	RP11-1023L17.1	0.862995989	1.861297455	-1.108882873
ENSG00000244346	RP11-531F16.3	0.065612	0.141531	-1.109086482
ENSG00000223442	TH2LCRR	0.138433228	0.298669041	-1.109357421
ENSG00000215559	ANKRD20A11P	0.019006097	0.04100808	-1.109445886
ENSG00000233719	GOT2P3	0.170049	0.367102	-1.110230452
ENSG00000233337	UBE2FP3	2.098012	4.530013	-1.110492261
ENSG00000144136	SLC20A1	51.63071814	111.4950566	-1.110678176
ENSG00000198431	TXNRD1	69.19835518	149.4483364	-1.110837187
ENSG00000278143	NF1P7	0.016659	0.035983	-1.111013671
ENSG00000131263	RLIM	12.84035669	27.73755482	-1.111155334
ENSG00000221419	MIR1287	1.074476	2.321169	-1.111218308
ENSG00000232682	RP11-388P9.2	0.031652619	0.068382618	-1.111304766
ENSG00000226088	RP11-180O5.2	0.483293	1.044608	-1.111991653
ENSG00000164118	CEP44	1.274896071	2.755818799	-1.112101387
ENSG00000114315	HES1	6.106557219	13.20264103	-1.112395407
ENSG00000242021	RP11-268G12.3	0.543401307	1.175047879	-1.112629603
ENSG00000269911	RP1-172N19.5	0.40307	0.87174	-1.1128675
ENSG00000183914	DNAH2	0.029537666	0.063885414	-1.112930778
ENSG00000197956	S100A6	194.633942	421.0451069	-1.113211477
ENSG00000129048	ACKR4	0.267132256	0.578118562	-1.113811204
ENSG00000243004	AC005062.2	0.572466747	1.239182246	-1.114124582
ENSG00000229636	KRT8P21	0.080264	0.173868	-1.115167472
ENSG00000250983	RP11-91K8.2	0.713891	1.546812	-1.115522143
ENSG00000281902	AC091053.2	0.084526	0.183199	-1.115944544
ENSG00000250031	RP11-114M5.1	1.010009	2.189565	-1.11627613
ENSG00000256771	ZNF253	0.014503986	0.031443905	-1.116330916
ENSG00000250151	ARPC4-TTLL3	2.6791139	5.808277356	-1.116354429
ENSG00000239978	OR7E53P	0.068574	0.148671	-1.116389677
ENSG00000232712	KIZ-AS1	0.053939749	0.117007568	-1.117181131
ENSG00000275022	MIR6753	0.310343	0.673206	-1.11718443
ENSG00000229057	RPS3AP54	0.069341	0.150417	-1.117187081
ENSG00000127328	RAB3IP	1.575671824	3.41805235	-1.117207407
ENSG00000261789	RP11-709D24.5	0.030011	0.065126	-1.117742228
ENSG00000230010	RP4-568F9.6	0.019576013	0.042481472	-1.117746816
ENSG00000198355	PIM3	27.90542922	60.55772607	-1.117765196
ENSG00000171102	OBP2B	0.021972609	0.047683381	-1.117780351

ENSG00000122136	OBP2A	0.024401654	0.052955658	-1.117805905
ENSG00000164037	SLC9B1	0.207555504	0.45062872	-1.118442071
ENSG00000140511	HAPLN3	4.00752933	8.700889747	-1.118449859
ENSG00000152766	ANKRD22	0.792371176	1.720917732	-1.118929826
ENSG00000279878	RP11-286N22.16	0.797279	1.731857	-1.119163236
ENSG00000258027	NF1P4	0.015066	0.032751	-1.120242523
ENSG00000231312	AC007246.3	0.349161961	0.759069958	-1.12033646
ENSG00000267879	CTB-147C22.9	0.083561	0.181748	-1.121037826
ENSG00000185100	ADSSL1	1.138479296	2.476246037	-1.121046611
ENSG00000215089	KRT18P11	6.439865	14.007171	-1.121063257
ENSG00000170802	FOXN2	1.363883511	2.967278126	-1.121419729
ENSG00000259429	UBE2Q2P2	2.180837063	4.745689664	-1.121735775
ENSG00000107147	KCNT1	0.0490141	0.106672424	-1.121918537
ENSG00000052841	TTC17	5.385108334	11.72005741	-1.121932363
ENSG00000228288	PCAT6	0.898940079	1.95685338	-1.122238807
ENSG00000188459	WASF4P	4.384546	9.54451	-1.122243668
ENSG00000270871	AC015849.19	2.812567	6.123683	-1.122512138
ENSG00000230325	RP11-385F5.4	0.721201	1.570468	-1.122721245
ENSG00000197670	RP4-724E16.2	0.425194	0.92597	-1.122844214
ENSG00000222314	RNU6-1118P	0.215004	0.46835	-1.123223565
ENSG00000259411	HNRNPA1P45	0.18791	0.409334	-1.12323666
ENSG00000079482	OPHN1	0.866670839	1.888625233	-1.123780383
ENSG00000269439	CTD-3131K8.2	0.695651084	1.516234418	-1.124057036
ENSG00000198160	MIER1	2.455705014	5.353478318	-1.124339289
ENSG00000143919	CAMKMT	0.726837152	1.585216679	-1.124975983
ENSG00000090238	YPEL3	1.150336041	2.509293388	-1.125225792
ENSG00000265766	CXADRP3	0.066282	0.144598	-1.125358558
ENSG00000276591	GXYLT1P5	0.047878039	0.104481856	-1.12581646
ENSG00000254317	RP11-473O4.5	0.610644	1.332869	-1.126131541
ENSG00000258150	RP11-345J4.3	0.264925182	0.578870113	-1.12765469
ENSG00000230074	RP11-195F19.9	0.782129	1.709069	-1.127732162
ENSG00000233825	RP11-135A24.4	0.077075	0.168482	-1.128259578
ENSG00000278217	MALAT1	84.274406	184.297165	-1.12886742
ENSG00000273891	RP11-500G22.5	0.842138	1.841714	-1.128920472
ENSG00000271817	U3	0.822706	1.799534	-1.12917449
ENSG00000165125	TRPV6	0.166306003	0.363799519	-1.129303393
ENSG00000155367	PPM1J	2.349801047	5.140594567	-1.129396621
ENSG00000253829	RP11-723D22.3	0.252913	0.553792	-1.130703018
ENSG00000267834	RP11-167N5.5	0.307221	0.672967	-1.131258927
ENSG00000199471	Y_RNA	0.503923	1.103845	-1.131262395
ENSG00000261216	RP11-166B2.5	0.397627	0.871195	-1.131579951
ENSG00000279355	AGPAT4-IT1	0.534979	1.172323	-1.131815951
ENSG00000229399	RP11-378J18.6	0.336258	0.737012	-1.132119518
ENSG00000127415	IDUA	1.150654222	2.522183161	-1.132218688
ENSG00000230585	PHBP12	0.121749	0.266932	-1.132562345
ENSG00000266820	RP13-104F24.1	11.37423	24.939487	-1.132662907
ENSG00000269374	CTB-50E14.5	14.171915	31.08083	-1.132990313
ENSG00000229644	NAMPTP1	13.754598	30.171255	-1.133260733

ENSG00000252206	RNU7-40P	2.783386	6.105473	-1.133262072
ENSG00000234828	RP11-526A4.1	0.038116571	0.08362302	-1.133481819
ENSG00000122574	WIPF3	3.223932182	7.07468658	-1.13384484
ENSG00000230068	CDC42-IT1	0.073549	0.16141	-1.133952331
ENSG00000202227	RNU6-282P	0.909214	1.99668	-1.134911332
ENSG00000073331	ALPK1	1.305946278	2.868696903	-1.135299995
ENSG00000234584	AC019186.1	0.127007	0.279025	-1.135486377
ENSG00000180509	KCNE1	0.008908877	0.019575611	-1.135741801
ENSG00000204060	FOXO6	0.938969182	2.063567	-1.135990568
ENSG00000141979	CTD-3222D19.2	0.034936	0.076808	-1.136542148
ENSG00000273000	KB-1572G7.2	0.329717944	0.725295983	-1.137337453
ENSG00000127362	TAS2R3	0.134059	0.294935	-1.13752896
ENSG00000130513	GDF15	64.8807005	142.7593391	-1.137723826
ENSG00000166387	PPFIBP2	0.380849387	0.838058358	-1.137830137
ENSG00000163545	NUAK2	1.350133	2.972304	-1.138480146
ENSG00000156508	EEF1A1	279.3368948	615.2737126	-1.139222218
ENSG00000167470	MIDN	20.08759801	44.25044346	-1.13938685
ENSG00000260691	ANKRD20A1	0.323381735	0.712375022	-1.139398735
ENSG00000010361	FUZ	0.084539411	0.186255685	-1.139588504
ENSG00000252974	AC121334.1	0.932715	2.055012	-1.139638594
ENSG00000244199	EIF4EP3	0.871546	1.921718	-1.140747929
ENSG00000272693	RP11-479O9.4	0.043326	0.095536	-1.140811425
ENSG00000100336	APOL4	0.0220927	0.048717337	-1.140865505
ENSG00000228656	MYO5BP3	0.127871	0.282098	-1.141507327
ENSG00000256591	RP11-286N22.8	0.09349672	0.206273997	-1.141574306
ENSG00000174132	FAM174A	3.598678441	7.943468976	-1.142301985
ENSG00000204396	VWA7	1.329914635	2.937210234	-1.143112887
ENSG00000269495	CTB-147C22.8	0.051102613	0.112868467	-1.143173516
ENSG00000231512	LINC01347	0.052290406	0.115501468	-1.143293004
ENSG00000234975	FTH1P2	227.782639	503.139435	-1.143300477
ENSG00000180263	FGD6	3.181447697	7.027668902	-1.143362819
ENSG00000161381	PLXDC1	0.187903506	0.415214497	-1.14386483
ENSG00000253047	SNORA40	1.932233	4.270007	-1.143969362
ENSG00000240673	AC006539.2	0.22756	0.503397	-1.145449633
ENSG00000184009	ACTG1	1009.831959	2234.37405	-1.145755482
ENSG00000274869	RP11-195B21.3	0.408407	0.904281	-1.146763557
ENSG00000064932	SBNO2	6.478250169	14.34439039	-1.146810571
ENSG00000173258	ZNF483	0.457637595	1.013522734	-1.147100972
ENSG00000232401	LINC00112	0.097502	0.215943	-1.147146834
ENSG00000106633	GCK	0.098780063	0.21880635	-1.147362811
ENSG00000175352	NRIP3	0.011851043	0.026253738	-1.147508765
ENSG00000224396	METTL15P3	0.083368	0.184686	-1.147508877
ENSG00000199347	RNU5E-1	0.40717	0.902009	-1.14751056
ENSG00000223343	RP13-131K19.2	0.062481	0.138415	-1.147510847
ENSG00000241622	RARRES2P1	0.203161	0.450066	-1.147513101
ENSG00000250874	CTC-480C2.1	0.033627	0.074495	-1.147523521
ENSG00000224682	SOCS5P2	0.035953	0.079648	-1.147525978
ENSG00000254974	RP11-702H23.2	0.051758	0.114662	-1.147533573

ENSG00000158786	PLA2G2F	0.007586717	0.016807337	-1.14754354
ENSG00000139352	ASCL1	0.019765	0.043787	-1.147554654
ENSG00000205133	TRIQK	2.413215741	5.346853416	-1.147733228
ENSG00000279428	RP11-258F1.2	0.023409	0.051872	-1.14789269
ENSG00000177191	B3GNT8	0.051603	0.114363	-1.148093525
ENSG00000007384	RHBDF1	2.060082043	4.566075764	-1.148253004
ENSG00000267078	RP11-666A8.9	0.082192	0.182187	-1.148350135
ENSG00000064787	BCAS1	0.055833047	0.123764451	-1.148405793
ENSG00000173275	ZNF449	1.959647394	4.345306335	-1.148863799
ENSG00000164463	CREBRF	1.101946134	2.443475628	-1.148881012
ENSG00000166046	TCP11L2	0.52917543	1.173736955	-1.14929114
ENSG00000262434	RP11-676J12.8	0.53478	1.186201	-1.149331076
ENSG00000127561	SYNGR3	1.049587346	2.328120419	-1.149343451
ENSG00000112365	ZBTB24	1.324904	2.940105	-1.14997985
ENSG00000278820	AC024937.1	0.207188	0.459785	-1.150018954
ENSG00000215009	ACSM4	0.194853684	0.432417655	-1.150034218
ENSG00000254162	RP11-48B3.3	0.783039	1.73772	-1.150039569
ENSG00000112335	SNX3	29.40405529	65.27724423	-1.150565014
ENSG00000143355	LHX9	0.009338576	0.020731939	-1.150580644
ENSG00000269843	CTC-490E21.10	0.895562	1.989005	-1.151181684
ENSG00000213557	RP11-240E2.2	0.401812	0.892514	-1.151354149
ENSG00000099338	CATSPERG	0.220425017	0.489756133	-1.151775591
ENSG00000224647	AC026954.6	0.408557	0.907812	-1.151856188
ENSG00000008282	SYPL1	12.74370559	28.32501154	-1.152291701
ENSG00000049883	PTCD2	0.648723264	1.441935954	-1.152332004
ENSG00000157483	MYO1E	7.811780119	17.3675636	-1.152672133
ENSG00000238273	AC012360.6	0.240597725	0.535329935	-1.153805327
ENSG00000176239	OR51B6	0.625497	1.393647	-1.155790314
ENSG00000273302	RP11-493E12.2	1.33321	2.970814	-1.155954238
ENSG00000078369	GNB1	29.12312471	64.90453288	-1.156154083
ENSG00000109083	IFT20	2.369645506	5.283457801	-1.156811171
ENSG00000281126	AC091180.1	1.017924	2.269692	-1.156866684
ENSG00000272888	LINC01578	4.424558897	9.869912676	-1.157503687
ENSG00000101782	RIOK3	13.55698506	30.26304631	-1.158520845
ENSG00000242291	RPL36AP51	0.144045	0.321579	-1.158653614
ENSG00000227376	FTH1P16	65.602554	146.457901	-1.158662138
ENSG00000241157	RP11-3K24.1	0.141378	0.315703	-1.159010332
ENSG00000258571	PTTG4P	0.344128	0.768757	-1.15958236
ENSG00000258588	TRIM6-TRIM34	0.320825	0.716896	-1.159977276
ENSG00000005187	ACSM3	1.094237144	2.446606194	-1.160856469
ENSG00000258086	RP11-753H16.5	0.279649	0.625459	-1.161298145
ENSG00000103018	CYB5B	26.26178632	58.73683258	-1.161300418
ENSG00000254680	RP11-265D17.2	0.065943	0.147553	-1.161941826
ENSG00000266824	RP11-599B13.7	0.207963	0.465554	-1.162621645
ENSG00000251141	RP11-53O19.1	0.39180362	0.877554915	-1.163358681
ENSG00000103089	FA2H	1.921141626	4.304849959	-1.163999077
ENSG00000229326	AC069154.4	0.073951	0.165757	-1.164428236
ENSG00000229089	ANKRD20A8P	0.118136798	0.264827929	-1.164596864

ENSG00000166819	PLIN1	0.091417787	0.205015022	-1.165182823
ENSG00000268660	LETM1P2	0.074156	0.166321	-1.165335006
ENSG00000140043	PTGR2	1.309172854	2.936434321	-1.165409776
ENSG00000231993	EP300-AS1	0.445481	0.999713	-1.166150081
ENSG00000241112	RPL29P14	0.558126	1.252802	-1.166495661
ENSG00000185338	SOCS1	0.741215	1.664703	-1.167300826
ENSG00000153684	GOLGA8F	0.030189275	0.067803568	-1.167325068
ENSG00000273597	RP11-392A14.9	0.087444	0.196445	-1.167694147
ENSG00000185532	PRKG1	0.601845429	1.352481989	-1.168144468
ENSG00000178177	LCORL	3.711997356	8.342407614	-1.168268122
ENSG00000254132	MTND6P3	3.428451	7.706684	-1.168553334
ENSG00000116016	EPAS1	8.047765333	18.0950129	-1.168931994
ENSG00000123870	ZNF137P	0.083786626	0.18840414	-1.169038783
ENSG00000131067	GGT7	2.110657768	4.751907696	-1.17081414
ENSG00000167011	NAT16	0.660296905	1.486921345	-1.171141544
ENSG00000104327	CALB1	0.033655587	0.075820534	-1.171742593
ENSG00000215403	LL22NC01-81G9.3	0.060542	0.136392	-1.171750786
ENSG00000222960	RNU6-272P	0.411085	0.926293	-1.172031881
ENSG00000184545	DUSP8	2.713332327	6.114079644	-1.172069587
ENSG00000233025	CRYZP1	0.797571	1.797922	-1.172645574
ENSG00000202119	RNU6-302P	0.475666	1.072294	-1.172679704
ENSG00000138468	SENP7	2.763393835	6.229968369	-1.172783651
ENSG00000154065	ANKRD29	0.80712415	1.819861548	-1.172966189
ENSG00000273069	RP5-1186P10.2	0.109157	0.246129	-1.173010002
ENSG00000263786	RP11-649A18.4	3.866872	8.720613	-1.173262541
ENSG00000232549	SRD5A1P1	0.349021	0.787708	-1.174347084
ENSG00000260948	RP11-552M11.8	3.023079	6.827267	-1.175289493
ENSG00000226450	CYP2D8P	0.178535	0.403214	-1.175338804
ENSG00000270194	RP11-259K5.2	0.179842	0.406231	-1.175570353
ENSG00000241537	RP11-624D20.1	0.218204	0.49306	-1.176085669
ENSG00000104951	IL4I1	0.046078504	0.104122318	-1.176113548
ENSG00000197181	PIWIL2	0.106199801	0.240007877	-1.176300691
ENSG00000273860	CTD-3066C23.1	0.898763	2.031668	-1.176652029
ENSG00000278601	RP11-348N5.9	0.379055	0.857013	-1.176909893
ENSG00000256748	RP11-234B24.5	0.016729	0.037846	-1.177789619
ENSG00000259664	CTD-2147F2.2	0.027719	0.062712	-1.177866319
ENSG00000108622	ICAM2	0.014752567	0.033376702	-1.177875379
ENSG00000216921	AC131097.4	0.070983323	0.160595267	-1.177877347
ENSG00000239614	HMGN1P7	0.152851	0.345818	-1.177886973
ENSG00000230641	USP12-AS2	0.171478	0.387961	-1.177888136
ENSG00000255437	RP11-510I21.1	0.518644	1.173592	-1.178114432
ENSG00000162576	MXRA8	0.282451502	0.639551345	-1.179057017
ENSG00000133103	COG6	4.518094558	10.23070204	-1.179118777
ENSG00000075826	SEC31B	3.353227726	7.595234756	-1.179544093
ENSG00000264047	RN7SL455P	0.166045	0.376157	-1.179760657
ENSG00000223256	RNU6-785P	0.247304	0.560286	-1.179877869
ENSG00000275076	AL928970.1	0.678616	1.537584	-1.179997878
ENSG00000117143	UAP1	19.22048163	43.57310302	-1.180793369

ENSG00000248092	NNT-AS1	0.023577185	0.053462376	-1.181132487
ENSG00000247228	RP11-296I10.3	0.016413199	0.03722276	-1.181328577
ENSG00000134545	KLRC1	0.04716086	0.106984125	-1.181734811
ENSG00000020922	MRE11A	2.40483906	5.457689209	-1.182349895
ENSG00000112394	SLC16A10	0.542648697	1.23165151	-1.182503684
ENSG00000248367	CTB-129O4.1	0.723851	1.642927	-1.182503715
ENSG00000034152	MAP2K3	15.93168451	36.17295608	-1.183012685
ENSG00000170615	SLC26A5	0.063601225	0.144557205	-1.184514061
ENSG00000232187	FTH1P7	108.15522	245.928009	-1.185132757
ENSG00000225080	PFN1P4	0.338802	0.770747	-1.185814978
ENSG00000164414	SLC35A1	15.63629275	35.59147907	-1.186633387
ENSG00000229431	RP1-92O14.6	0.986364	2.245218	-1.18666348
ENSG00000232956	SNHG15	3.453799216	7.862287265	-1.186764861
ENSG00000183798	EMILIN3	0.017699	0.040291	-1.186789763
ENSG00000181143	MUC16	0.037786926	0.086027513	-1.186910951
ENSG00000123338	NCKAP1L	0.038138463	0.086829876	-1.186944828
ENSG00000182472	CAPN12	0.210708925	0.47998205	-1.187729034
ENSG00000254297	CTC-286N12.1	0.010996	0.025056	-1.188177306
ENSG00000137962	ARHGAP29	27.8236779	63.40120158	-1.188197046
ENSG00000183148	ANKRD20A2	0.338972866	0.772516201	-1.188395394
ENSG00000237310	GS1-124K5.4	0.737052	1.679741	-1.188400488
ENSG00000185250	PPIL6	0.211529218	0.482145525	-1.188611707
ENSG00000225292	RP11-57H14.3	1.063875	2.424948	-1.188625159
ENSG00000220804	AC093642.5	0.696388152	1.589057863	-1.190208095
ENSG00000278861	RP11-338K17.6	1.297918	2.96175	-1.190250629
ENSG00000244560	RP4-800G7.2	0.841318008	1.920483587	-1.190746503
ENSG00000203875	SNHG5	6.668081133	15.22278304	-1.190888574
ENSG00000188785	ZNF548	0.188104091	0.429487781	-1.191085866
ENSG00000178809	TRIM73	0.42198054	0.963534736	-1.191160209
ENSG00000135046	ANXA1	80.39930004	183.7640414	-1.192599644
ENSG00000214278	CTD-2228K2.2	0.058006	0.132648	-1.193328882
ENSG00000171346	KRT15	2.333114199	5.335553037	-1.193380891
ENSG00000231845	HMGB3P14	0.338175	0.773408	-1.193459677
ENSG00000213041	RP11-383G10.3	0.771967	1.765963	-1.193844035
ENSG00000118985	ELL2	7.931119308	18.14476074	-1.193956642
ENSG00000188732	FAM221A	0.084622501	0.193609185	-1.194034173
ENSG00000111752	PHC1	2.357387978	5.394056655	-1.194181458
ENSG00000236908	RP5-1063M23.2	0.133848	0.306269655	-1.194206851
ENSG00000236299	RP11-340I6.7	0.075128546	0.17192215	-1.194322345
ENSG00000202363	SNORA62	0.517202	1.183612	-1.194396469
ENSG00000224680	PLA2G12AP1	1.433696	3.281534	-1.194631233
ENSG00000189375	TBC1D28	0.084426238	0.193327102	-1.195280563
ENSG00000260585	RP13-192B19.2	0.13241	0.303257	-1.195528864
ENSG00000172014	ANKRD20A4	0.340967687	0.781248415	-1.196146333
ENSG00000075290	WNT8B	0.730786	1.674556	-1.196257722
ENSG00000152056	AP1S3	9.470473743	21.70723389	-1.196667397
ENSG00000167996	FTH1	217.0110118	497.511614	-1.196961954
ENSG00000201403	SNORD14B	5.078497	11.644488	-1.197173712

ENSG00000080822	CLDND1	31.23706148	71.64916524	-1.197691153
ENSG00000223571	DHRSX-IT1	0.029709	0.06817	-1.198236938
ENSG00000242687	AC004893.11	0.158010992	0.362585708	-1.198297137
ENSG00000277481	PKD1L3	0.027215	0.062458	-1.198484337
ENSG00000221963	APOL6	0.125819	0.288757	-1.19850612
ENSG00000259682	RP11-702L15.4	0.114549	0.262902	-1.198560253
ENSG00000280486	LLNLR-307A6.1	0.111778	0.256609	-1.198935504
ENSG00000004660	CAMKK1	1.287038572	2.95505778	-1.199131048
ENSG00000276241	CTB-91J4.1	0.289524623	0.664883695	-1.19941595
ENSG00000212533	SNORA75	1.434142	3.294137	-1.199712679
ENSG00000177879	AP3S1	87.28002815	200.4785719	-1.199724571
ENSG00000169762	TAPT1	0.482573083	1.108719652	-1.200075264
ENSG00000170667	RASA4B	0.654733087	1.505380511	-1.201149407
ENSG00000213689	TREX1	0.496806282	1.142312229	-1.201201716
ENSG00000254589	EIF4A2P3	0.6754	1.553251	-1.201476899
ENSG00000281643	AC027806.1	0.451245	1.037775	-1.201510835
ENSG00000221164	SNORA11	1.85294	4.261992	-1.201711719
ENSG00000204653	ASPDH	0.31556605	0.725855768	-1.201740901
ENSG00000146530	VWDE	2.32726786	5.354830911	-1.20220375
ENSG00000268650	AC068499.10	0.083147257	0.191416418	-1.202973996
ENSG00000118217	ATF6	6.936720495	15.97008454	-1.20304629
ENSG00000166710	B2M	28.34874213	65.29351278	-1.203654938
ENSG00000115137	DNAJC27	0.419768943	0.966874535	-1.203733259
ENSG00000131016	AKAP12	136.8940892	315.3342676	-1.203821801
ENSG00000269067	ZNF728	0.00464406	0.010699172	-1.204040566
ENSG00000218793	RP3-382I10.3	0.103658	0.238821	-1.204098238
ENSG00000206932	RNU6-4P	0.285399	0.65754	-1.20409838
ENSG00000168811	IL12A	1.97427651	4.553903223	-1.20577957
ENSG00000237701	ATP5JP1	0.316126	0.729451	-1.206311375
ENSG00000169429	CXCL8	1.853165877	4.27898742	-1.207277415
ENSG00000219507	FTH1P8	89.651382	207.072617	-1.207739058
ENSG00000213830	CFL1P5	1.090922	2.520594	-1.208215804
ENSG00000253313	C1orf210	2.179539135	5.036494611	-1.208396862
ENSG00000205810	KLRC3	1.214501725	2.806757892	-1.208540085
ENSG00000176381	PRR18	0.00743529	0.017186763	-1.208836926
ENSG00000166104	RP11-299H22.3	0.012018564	0.027782503	-1.20891207
ENSG00000241420	RN7SL505P	0.079905	0.184711	-1.208912098
ENSG00000233189	RPL12P29	0.10246	0.236851	-1.208918966
ENSG00000258930	RP5-1163L11.2	0.060718	0.140524	-1.210620373
ENSG00000213470	RP11-972K6.1	0.070262	0.162659	-1.211034101
ENSG00000236872	RP11-291L19.1	0.147207	0.340821	-1.211167955
ENSG00000086712	TXLNG	4.188920178	9.703155558	-1.211875608
ENSG00000242252	BGLAP	0.245094514	0.567794212	-1.212029948
ENSG00000219532	RP3-323K23.3	0.440279	1.020613	-1.212945984
ENSG00000201944	SNORA72	1.156732	2.681427	-1.212946328
ENSG00000238358	RP5-1121E10.2	0.151456	0.351146	-1.213172269
ENSG00000148154	UGCG	9.541882234	22.12792798	-1.21352258
ENSG00000059769	DNAJC25	3.837050799	8.903570648	-1.214386159

ENSG00000266708	RP11-661O13.1	0.312801	0.725935	-1.214595251
ENSG00000011007	TCEB3	9.425053878	21.87498296	-1.214709123
ENSG00000166343	MSS51	0.935544142	2.172486298	-1.215469446
ENSG00000274951	AC005538.1	0.700406	1.628581	-1.217352128
ENSG00000256690	RP11-727F15.9	0.478100046	1.111962035	-1.217723083
ENSG00000237264	FTH1P11	206.159058	479.496429	-1.217762221
ENSG00000188488	SERPINA5	1.37252226	3.194612781	-1.218811526
ENSG00000272296	SNORD96A	1.505194	3.503578	-1.218879572
ENSG00000212455	SNORA40	2.737129	6.37213	-1.21911227
ENSG00000169918	OTUD7A	0.440631757	1.026140935	-1.219583511
ENSG00000225951	ODF2-AS1	1.119445	2.607893	-1.22010103
ENSG00000100739	BDKRB1	0.09226982	0.215069409	-1.220871587
ENSG00000108551	RASD1	0.289421475	0.674702145	-1.221078777
ENSG00000250927	MESTP3	0.155633	0.362853	-1.2212372
ENSG00000258136	RP11-864J10.4	0.270289848	0.630229358	-1.221369634
ENSG00000146072	TNFRSF21	23.834574	55.589676	-1.221761133
ENSG00000252010	SCARNA5	0.228664	0.533362	-1.221885791
ENSG00000222881	Y_RNA	3.910692	9.124621	-1.22234072
ENSG00000253203	GUSBP3	0.882491	2.059575	-1.222693193
ENSG00000196420	S100A5	2.576757054	6.013897661	-1.222743797
ENSG00000239503	MARK2P8	0.219196	0.511644	-1.222918866
ENSG00000114857	NKTR	4.366486958	10.20460735	-1.224675736
ENSG00000188290	HES4	14.86282853	34.73659177	-1.224747509
ENSG00000249661	TNRC18P1	0.075857	0.177338	-1.225147488
ENSG00000206652	RNU1-1	0.253239	0.592026	-1.225160932
ENSG00000243970	PPIEL	0.487363	1.139829	-1.225748771
ENSG00000258439	RP11-173A8.2	0.281714	0.659032	-1.226117257
ENSG00000235721	AC013268.3	0.22904	0.536136	-1.226999436
ENSG00000117152	RGS4	0.126293991	0.295646227	-1.227085865
ENSG00000240975	RP11-313M3.1	0.087957	0.205973	-1.227584931
ENSG00000261886	RP11-63A1.1	0.108235	0.253476	-1.227682055
ENSG00000270067	CTC-487M23.5	0.759258	1.779748	-1.22901087
ENSG00000118298	CA14	0.233599195	0.547730713	-1.229431475
ENSG00000248394	FOSL1P1	1.881788	4.413874	-1.229941342
ENSG00000201900	RNY1P13	0.659227	1.546301	-1.229973941
ENSG00000224600	RP4-612B18.1	1.381469	3.241047	-1.230256753
ENSG00000257337	RP11-983P16.4	0.981406798	2.302805075	-1.230469127
ENSG00000270116	AP001429.1	1.254159	2.944975	-1.231535125
ENSG00000117016	RIMS3	2.599822	6.105484	-1.231692816
ENSG00000272910	RP11-15L13.4	0.636843	1.496176	-1.232270238
ENSG00000279660	RP11-849N15.3	0.076594	0.179994	-1.232645528
ENSG00000276248	RP11-230F18.5	1.097857	2.580465	-1.232940913
ENSG00000213785	RP11-449L13.2	0.188552	0.443335	-1.23343481
ENSG00000223886	RP11-251G23.2	0.384745	0.904736	-1.2335943
ENSG00000171302	CANT1	7.210027773	16.95511104	-1.23364351
ENSG00000255717	SNHG1	38.51650678	90.60927252	-1.234181831
ENSG00000197989	SNHG12	7.141213094	16.80330865	-1.23450426
ENSG00000212493	SNORD19	2.470359	5.816519	-1.235435291

ENSG00000266261	RP11-640I15.1	0.078439	0.184745	-1.235892271
ENSG00000265511	RP11-524F11.1	0.686112	1.616523	-1.23637803
ENSG00000236417	CTSLP1	0.409066	0.964124	-1.236885078
ENSG00000249563	RP11-751L19.1	0.148957	0.351133	-1.237121667
ENSG00000164221	CCDC112	7.451762645	17.57011617	-1.237470103
ENSG00000169026	MFSD7	0.1108786	0.261454831	-1.237580776
ENSG00000175175	PPM1E	0.107862	0.254424	-1.238048078
ENSG00000273145	CITF22-92A6.1	0.548178	1.293154	-1.238177759
ENSG00000255008	AP000442.4	0.054272	0.128064	-1.238584997
ENSG00000269946	RP11-2B6.3	0.21658	0.511266	-1.239174064
ENSG00000244060	RPS2P41	0.065836	0.15544	-1.239409216
ENSG00000185885	IFITM1	6.957217333	16.42624797	-1.239420688
ENSG00000172346	CSDC2	0.06264143	0.147928328	-1.2397093
ENSG00000241069	CTD-3141N22.1	1.992342	4.705972	-1.240027419
ENSG00000196850	PPTC7	10.05101783	23.74623606	-1.240357249
ENSG00000138964	PARVG	0.011367481	0.026859297	-1.240508899
ENSG00000223949	ROR1-AS1	0.020680615	0.048867019	-1.240582055
ENSG00000280117	RP11-158L12.6	0.010425	0.024634	-1.240603524
ENSG00000116157	GPX7	0.02708862	0.064009954	-1.240609383
ENSG00000200344	Y_RNA	0.444724	1.050884	-1.24062126
ENSG00000244623	OR2AE1	0.04277	0.101066	-1.240626624
ENSG00000217702	RP11-287D1.4	0.036239222	0.085633747	-1.240627487
ENSG00000215866	LINC01356	0.012563373	0.029687716	-1.240642274
ENSG00000078747	ITCH	14.72024973	34.78662804	-1.240730694
ENSG00000156510	HKDC1	0.461823588	1.091787942	-1.241278902
ENSG00000159479	MED8	8.099898966	19.15882578	-1.242033325
ENSG00000263968	RN7SL381P	0.669507	1.583661	-1.242092499
ENSG00000102349	KLF8	0.182076354	0.430708905	-1.242169576
ENSG00000185513	L3MBTL1	0.971068972	2.297444899	-1.242384587
ENSG00000212939	RP1-29C18.10	0.052282	0.123709	-1.242564226
ENSG00000263069	CTD-2047H16.4	0.32359771	0.765932544	-1.24301594
ENSG00000101871	MID1	2.966586008	7.023090451	-1.243302405
ENSG00000188060	RAB42	0.090118624	0.21334789	-1.243310653
ENSG00000260465	RP11-63M22.2	1.594466	3.775178	-1.243471335
ENSG00000230251	PHBP4	0.186579	0.441844	-1.243750477
ENSG00000225193	RPS12P26	2.289901	5.423443	-1.243923791
ENSG00000259366	CTD-2647L4.4	3.057398	7.243805	-1.244443341
ENSG00000097096	SYDE2	0.670621165	1.589038452	-1.244584115
ENSG00000154553	PDLIM3	1.264666823	2.997393252	-1.244951019
ENSG00000213453	FTH1P3	61.136078	144.996033	-1.245917521
ENSG00000182021	RP11-381O7.3	0.201634976	0.47833822	-1.246285157
ENSG00000163705	FANCD2OS	0.060188674	0.142821516	-1.246649402
ENSG00000073605	GSDMB	4.518466447	10.72517852	-1.247096546
ENSG00000164385	LINC01600	0.121281	0.288168	-1.248556584
ENSG00000167654	ATCAY	0.004898643	0.011659055	-1.250996778
ENSG00000132424	PNISR	10.1345684	24.12410346	-1.251190677
ENSG00000164236	ANKRD33B	3.811778788	9.075525169	-1.251516733
ENSG00000266913	CTC-548K16.2	0.050094638	0.119321223	-1.252122587

ENSG00000167414	GNG8	0.23563	0.561251	-1.252122878
ENSG00000230484	OR51A10P	0.733296	1.747799	-1.253071707
ENSG00000169239	CA5B	0.62518174	1.490353572	-1.25330709
ENSG00000182732	RGS6	0.263142545	0.627628212	-1.254065681
ENSG00000215869	RP11-364B6.2	0.043033678	0.102701706	-1.254922085
ENSG00000275894	RP3-453C12.14	0.885838	2.114442	-1.255162196
ENSG00000259388	CTD-2647E9.1	1.153592	2.754536	-1.255676253
ENSG00000230615	RP5-1198O20.4	0.022651653	0.05412726	-1.256738983
ENSG00000040933	INPP4A	1.324353149	3.164783313	-1.256818845
ENSG00000236394	RP11-229P13.15	0.087752	0.209737	-1.25707748
ENSG00000278570	NR2E3	0.087825534	0.209977593	-1.257523038
ENSG00000231252	RP11-436K8.1	0.023755633	0.056835646	-1.258526368
ENSG00000235369	RPL36AP15	0.765357	1.831146	-1.25854207
ENSG00000282432	RP11-369J21.13	0.706658	1.690708	-1.258543445
ENSG00000221883	ARIH2OS	0.127399	0.304809	-1.258551548
ENSG00000163121	NEURL3	0.055441161	0.132646062	-1.258552461
ENSG00000278890	LL22NC03-27C5.1	0.007955	0.019033	-1.258569143
ENSG00000274028	RP11-440D17.5	0.014901	0.035652	-1.258573858
ENSG00000273186	RP11-339B21.10	0.77402	1.852536	-1.259058828
ENSG00000116678	LEPR	1.041222956	2.492068353	-1.259064614
ENSG00000160460	SPTBN4	0.365063932	0.873755859	-1.259081086
ENSG00000147437	GNRH1	1.308512054	3.137060159	-1.261485981
ENSG00000280408	RP11-818O24.2	0.46518	1.117327	-1.264190495
ENSG00000142619	PADI3	0.412085	0.99028	-1.264894552
ENSG00000260310	RP11-27M24.2	0.117003	0.281178	-1.264938198
ENSG00000228513	AC023271.1	0.093387	0.224426	-1.264946186
ENSG00000258499	RP11-862G15.2	0.073549	0.176752	-1.264948908
ENSG00000006652	IFRD1	4.303835563	10.35023112	-1.265968121
ENSG00000243695	RP11-290L7.3	0.212067	0.510011	-1.266008225
ENSG00000184292	TACSTD2	0.858157	2.063993	-1.26612456
ENSG00000250068	RP11-576C12.1	0.317122	0.763436	-1.267469252
ENSG00000197978	GOLGA6L9	2.327768155	5.603966069	-1.267500847
ENSG00000250920	RP11-297P16.4	20.854095	50.213894	-1.267755903
ENSG00000218069	RSL24D1P1	4.534064	10.918517	-1.267900256
ENSG00000235245	RP11-122K13.12	1.144212	2.755761	-1.268096392
ENSG00000274094	MIR6845	0.835897	2.015558	-1.269782211
ENSG00000250820	RP11-91H12.4	0.060036	0.144785	-1.270012381
ENSG00000260851	RP11-403P17.5	0.060383292	0.145690751	-1.270687978
ENSG00000125409	TEKT3	0.242288938	0.584787042	-1.271182805
ENSG00000238405	RNA5SP311	0.711523	1.717393	-1.271237919
ENSG00000206611	SNORD24	1.299	3.135573	-1.271327677
ENSG00000237679	VDAC1P11	0.139785	0.337512	-1.27172924
ENSG00000243368	MCCC1-AS1	0.8112	1.958951	-1.271951754
ENSG00000187867	PALM3	1.080583377	2.609496066	-1.271960834
ENSG00000172137	CALB2	7.072833704	17.08875143	-1.272686746
ENSG00000237223	SULT1C2P1	0.022350299	0.054011976	-1.272985173
ENSG00000143318	CASQ1	0.010291399	0.0248711	-1.273031137
ENSG00000196114	RP3-391O22.3	0.112253	0.271281	-1.273034006

ENSG00000254639	CTD-2589M5.5	0.068589	0.165759	-1.273038078
ENSG00000200397	Y_RNA	0.219552	0.530593	-1.273042966
ENSG00000178404	CEP295NL	0.4208041	1.017740608	-1.27414924
ENSG00000214319	CXADRP1	0.150907	0.365087	-1.27458057
ENSG00000206356	RP11-93O17.2	0.048845	0.118264	-1.275728185
ENSG00000245148	ARAP1-AS2	0.158593	0.384128	-1.276258035
ENSG00000213417	KRTAP2-4	1.292391	3.130585	-1.276389664
ENSG00000197077	KIAA1671	2.80201006	6.788966077	-1.276729741
ENSG00000258644	SYNJ2BP-COX16	0.653903732	1.586651661	-1.278835266
ENSG00000279579	bP-2189O9.3	0.060233	0.146221086	-1.279525354
ENSG00000240729	CTD-2301A4.3	0.282282	0.685602	-1.280234181
ENSG00000230732	AC127904.2	1.066912	2.591343	-1.280258803
ENSG00000149609	C20orf144	0.08211778	0.199461463	-1.28034351
ENSG00000251603	RP11-164P12.4	1.582479	3.844004	-1.280423483
ENSG00000102878	HSF4	0.507990344	1.235015	-1.281655585
ENSG00000232671	RP11-126K1.2	0.245481	0.596892	-1.281858552
ENSG00000269292	CTB-12A17.3	0.401493	0.976391	-1.282084162
ENSG00000101096	NFATC2	0.63377301	1.542929663	-1.283634169
ENSG00000155719	OTOA	0.014120387	0.034383705	-1.283945383
ENSG00000164327	RICTOR	2.491323918	6.06671653	-1.284003292
ENSG00000166669	ATF7IP2	1.370674725	3.338548191	-1.28433462
ENSG00000227777	RP4-738P11.3	0.058061	0.141431	-1.284457051
ENSG00000166822	TMEM170A	2.257111593	5.498846775	-1.284651338
ENSG00000100346	CACNA1I	0.11144189	0.271530919	-1.284824851
ENSG00000140297	GCNT3	0.158630511	0.386598289	-1.285164969
ENSG00000185559	DLK1	0.012455697	0.030360684	-1.285398529
ENSG00000117228	GBP1	0.019881826	0.048471971	-1.285700476
ENSG00000279541	CTC-444N24.7	0.881418	2.148905	-1.28570344
ENSG00000173482	PTPRM	1.804584279	4.400495686	-1.28599952
ENSG00000127914	AKAP9	3.103318066	7.567673438	-1.286038265
ENSG00000232684	ATP11A-AS1	0.069658	0.169867	-1.286044652
ENSG00000267322	SNHG22	0.085579321	0.208718041	-1.286221166
ENSG00000171914	TLN2	0.54202135	1.322088211	-1.286396852
ENSG00000179930	ZNF648	0.13948	0.340237	-1.28648177
ENSG00000095585	BLNK	0.036419341	0.088843322	-1.286558532
ENSG00000254979	RP11-872D17.8	0.807439439	1.970379214	-1.287047352
ENSG00000138271	GPR87	0.624964084	1.525447427	-1.287387272
ENSG00000272800	RP11-438L19.1	0.235195	0.574218	-1.287741165
ENSG00000172638	EFEMP2	0.186903189	0.456388625	-1.287971648
ENSG00000123584	MAGEA9	0.025252	0.061698	-1.288826068
ENSG00000269068	RP11-256I23.2	0.842033	2.057376	-1.288856801
ENSG00000188536	HBA2	0.20632	0.504535598	-1.290072386
ENSG00000227212	PFN1P6	1.698877	4.156476	-1.290779477
ENSG00000231871	IPO9-AS1	0.052936088	0.129513801	-1.29078235
ENSG00000272512	RP11-54O7.17	1.204333	2.946951	-1.290988718
ENSG00000261064	RP11-1000B6.3	0.16116467	0.394416323	-1.291183745
ENSG00000178761	FAM219B	1.274245666	3.118633648	-1.29127064
ENSG00000174130	TLR6	0.177567261	0.434668215	-1.291548998

ENSG00000225347	SLC25A5P8	0.047231	0.115714	-1.292757437
ENSG00000186150	UBL4B	0.133611	0.32736	-1.292839263
ENSG00000251448	RP11-71E19.2	0.092174	0.225857	-1.292977863
ENSG00000180525	PRR26	0.014090209	0.034531339	-1.293213279
ENSG00000109511	ANXA10	2.193424935	5.375712422	-1.293270628
ENSG00000249992	TMEM158	10.72554	26.295475	-1.293764273
ENSG00000265460	RP11-690G19.4	0.048772308	0.119616556	-1.294282953
ENSG00000259314	CTD-3065B20.3	0.175107	0.429553	-1.29459939
ENSG00000265329	MIR4758	0.005735	0.014072	-1.294961997
ENSG00000199691	RN7SKP173	3.108134	7.630322	-1.295695237
ENSG00000275759	RP11-131L12.3	0.672865	1.65297	-1.296671557
ENSG00000249673	NOP14-AS1	0.631507297	1.55140583	-1.296704818
ENSG00000233523	PHBP5	0.157725	0.387582	-1.29709022
ENSG00000279980	GABARAPL3	0.205949	0.506119	-1.297189514
ENSG00000178764	ZHX2	0.641490976	1.576794785	-1.297494035
ENSG00000242992	FTH1P4	31.875252	78.357132	-1.297627846
ENSG00000236924	RP11-390F4.6	0.916287	2.252687	-1.297775416
ENSG00000230204	FTH1P5	65.845879	161.89386	-1.297883214
ENSG00000262420	RP11-490O6.2	0.13405	0.329623	-1.298045693
ENSG00000213244	HIST2H3DP1	0.030587	0.075239	-1.298562061
ENSG00000251880	RNU7-75P	3.710497	9.130658	-1.299106391
ENSG00000100307	CBX7	0.815524867	2.006931855	-1.299190857
ENSG00000223361	FTH1P10	141.0678489	347.1613276	-1.299217029
ENSG00000078795	PKD2L2	0.065275597	0.160654812	-1.299348537
ENSG00000255090	MIR100HG	1.3968126	3.438529535	-1.29965326
ENSG00000150995	ITPR1	1.086590413	2.675175138	-1.299825122
ENSG00000163157	TMOD4	0.051262748	0.126277038	-1.300609611
ENSG00000248445	SEMA6A-AS1	0.265255726	0.653732095	-1.301315636
ENSG00000233690	EBAG9P1	1.08541	2.675129	-1.301368357
ENSG00000226564	FTH1P20	134.441284	331.512451	-1.30208683
ENSG00000161298	ZNF382	0.00449713	0.011093804	-1.302677616
ENSG00000233343	ATP6V1G1P4	0.151929	0.374805	-1.302742923
ENSG00000201121	RNY1P12	0.998535	2.463773	-1.302984437
ENSG00000161912	ADCY10P1	0.192056261	0.473952067	-1.303212164
ENSG00000122861	PLAU	11.61120017	28.65533192	-1.303286507
ENSG00000074935	TUBE1	3.459695458	8.547152824	-1.304798869
ENSG00000112406	HECA	5.155621	12.739555	-1.305096767
ENSG00000222792	RNU6-860P	0.19445	0.48084	-1.306157681
ENSG00000214733	RP11-429J17.8	0.268031549	0.662934687	-1.306463917
ENSG00000128594	LRRC4	0.037831377	0.093597256	-1.306882956
ENSG00000198542	ITGBL1	0.288261476	0.713203351	-1.306935437
ENSG00000272573	MUSTN1	0.632260247	1.565586632	-1.308112923
ENSG00000124091	GCNT7	0.164023	0.406259	-1.308501644
ENSG00000168010	ATG16L2	0.722579507	1.789958014	-1.308697503
ENSG00000279594	RP11-950C14.10	0.064303	0.159319	-1.308960378
ENSG00000003147	ICA1	1.834977367	4.548259396	-1.309552268
ENSG00000216133	MIR939	0.241001	0.59763	-1.310213438
ENSG00000175509	RP11-395L14.13	0.10646	0.264052	-1.310510597

ENSG00000185046	ANKS1B	0.013814402	0.034275359	-1.310998679
ENSG00000270673	YTHDF3-AS1	0.212659	0.527637	-1.311003823
ENSG00000279259	RP11-334C17.3	0.113355	0.28125	-1.311005068
ENSG00000227321	MTND4P15	0.147811	0.366741	-1.311007924
ENSG00000231675	AC005042.3	0.43876	1.088631	-1.311011112
ENSG00000172215	CXCR6	0.121535175	0.301547547	-1.311011579
ENSG00000250547	RP11-719L21.1	0.075663	0.187732	-1.311014702
ENSG00000152672	CLEC4F	0.061692	0.153068	-1.311017385
ENSG00000124212	PTGIS	0.013684	0.033953	-1.311049044
ENSG00000231924	PSG1	0.00659482	0.016363915	-1.311112632
ENSG00000023171	GRAMD1B	0.674321628	1.673332882	-1.311215699
ENSG00000221826	PSG3	0.00208532	0.005174767	-1.311224923
ENSG00000262171	CTA-972D3.2	0.538937	1.337545	-1.311398888
ENSG00000225171	DUTP6	0.440659	1.094126	-1.312044313
ENSG00000143552	NUP210L	0.043213	0.107341	-1.312663937
ENSG00000134954	ETS1	2.255038534	5.602499353	-1.312918491
ENSG00000280316	AL365181.2	0.17568	0.43664	-1.31349434
ENSG00000275915	NPPA-AS1_3	0.537238	1.33631	-1.314621468
ENSG00000153208	MERTK	2.346274694	5.837926627	-1.31508415
ENSG00000159527	PGLYRP3	0.086779	0.215928	-1.315132467
ENSG00000198216	CACNA1E	0.005728234	0.014253377	-1.315141438
ENSG00000229859	PGA3	0.004545203	0.011313245	-1.315596057
ENSG00000178502	KLHL11	2.803678	6.978664	-1.315630206
ENSG00000166448	TMEM130	0.013971051	0.034819569	-1.317457722
ENSG00000232386	RP11-66B24.2	0.213020103	0.530916151	-1.317494447
ENSG00000258712	CXADRP2	0.337174	0.840716	-1.318125238
ENSG00000166949	SMAD3	6.58919115	16.42992556	-1.318152659
ENSG00000189283	FHIT	1.082448437	2.700016335	-1.318669833
ENSG00000276564	CTA-276F8.1	0.180032	0.44909	-1.318751235
ENSG00000170846	AC093323.3	2.388613396	5.961690512	-1.319548115
ENSG00000240499	RP5-1101C3.1	0.038562146	0.096273144	-1.319948057
ENSG00000047662	FAM184B	0.043734	0.109211	-1.320290964
ENSG00000205189	ZBTB10	3.499734557	8.73944103	-1.320295506
ENSG00000165269	AQP7	0.101051039	0.252348374	-1.320332634
ENSG00000234042	RP11-452D2.2	0.69789	1.743763	-1.321132408
ENSG00000204380	AC005042.4	0.114286577	0.285620749	-1.321444816
ENSG00000145390	USP53	3.802408525	9.504980814	-1.321770173
ENSG00000234134	RP11-383C5.5	0.261006	0.652485	-1.321861764
ENSG00000170442	KRT86	0.220365546	0.551027293	-1.3222251
ENSG00000187959	CPSF4L	0.045300049	0.113341355	-1.323089841
ENSG00000262648	PHBP15	0.145418	0.364059	-1.323966416
ENSG00000005700	IBTK	7.704570483	19.30456007	-1.325155241
ENSG00000139597	N4BP2L1	0.108523063	0.271948217	-1.325330291
ENSG00000207500	SNORD102	8.003938	20.060019	-1.325541075
ENSG00000162927	PUS10	0.728015702	1.826728003	-1.327220363
ENSG00000137818	RPLP1	36.38189589	91.33199583	-1.327899635
ENSG00000083168	KAT6A	3.114496334	7.825151505	-1.32911981
ENSG00000257803	RP11-575G13.2	0.205125	0.516639	-1.33265322

ENSG00000258539	RP11-12J10.3	0.456611	1.150207	-1.332856003
ENSG00000116690	PRG4	0.024426	0.061532	-1.332919259
ENSG00000279203	AC005785.5	0.837247	2.109929	-1.333469246
ENSG00000249844	OR7E43P	0.016596	0.041832	-1.333771413
ENSG00000139890	REM2	0.219483128	0.553345961	-1.334071721
ENSG00000266758	MIR3680-2	0.378309	0.953848	-1.334194286
ENSG00000112212	TSPO2	0.215883555	0.5443835	-1.334369993
ENSG00000235413	KRT18P63	0.135298	0.341336	-1.335052067
ENSG00000134827	TCN1	0.387240653	0.977027763	-1.335169141
ENSG00000271992	RP11-42O15.3	0.535175	1.350532	-1.335445195
ENSG00000270184	RP11-568J23.5	1.708478	4.312157	-1.335698035
ENSG00000174521	TTC9B	0.091513965	0.231086254	-1.336367632
ENSG00000223650	UHRF2P1	0.083797	0.211615	-1.336471394
ENSG00000254507	RP11-481A20.10	0.065576041	0.165610428	-1.336552804
ENSG00000188004	C1orf204	0.101345	0.256036	-1.337071762
ENSG00000167378	IRGQ	0.812516578	2.052839744	-1.337151852
ENSG00000124232	RBPJL	0.072208025	0.182556758	-1.338113988
ENSG00000103184	SEC14L5	0.047148606	0.119320477	-1.339554629
ENSG00000281146	AC109460.1	0.320277	0.810748	-1.339933362
ENSG00000260803	Z84812.4	0.282658	0.715584	-1.3400636
ENSG00000137225	CAPN11	0.006023276	0.015248821	-1.340077283
ENSG00000225726	AC007000.10	0.131682	0.333391	-1.340157006
ENSG00000110888	CAPRIN2	4.612313411	11.67755315	-1.340175558
ENSG00000105398	SULT2A1	0.010758	0.027238	-1.340210881
ENSG00000273076	RP3-508I15.22	0.244694	0.619914	-1.341089359
ENSG00000033122	LRRC7	0.111098523	0.28147868	-1.341186019
ENSG00000116194	ANGPTL1	0.023331713	0.059130619	-1.341613132
ENSG00000244556	ODCP	0.350901	0.890232	-1.343117301
ENSG00000257838	RP11-368J21.3	0.02869	0.072812	-1.34362827
ENSG00000151014	NOCT	6.16295129	15.64684425	-1.344178421
ENSG00000259628	RP11-467H10.2	0.295141	0.749461	-1.344449057
ENSG00000176225	RTTN	3.579320017	9.090442055	-1.344664915
ENSG00000010310	GIPR	1.516453868	3.8517961	-1.344829724
ENSG00000278757	U6	0.176179	0.447882	-1.346076717
ENSG00000184313	MROH7	0.175651654	0.446563437	-1.346147968
ENSG00000147894	C9orf72	2.782220803	7.079824029	-1.347476582
ENSG00000198062	POTEH	0.006954941	0.017702552	-1.347847156
ENSG00000237940	AC093642.3	0.219406783	0.558988052	-1.349209315
ENSG00000107679	PLEKHA1	1.459633746	3.720729655	-1.349979159
ENSG00000270977	AC015849.16	0.077172547	0.196750652	-1.350208786
ENSG00000224789	AC012363.4	0.24919	0.635363	-1.350334904
ENSG00000213816	CNN2P4	0.046167	0.11774	-1.350670641
ENSG00000275043	SNORD25	0.057736	0.147281	-1.351028262
ENSG00000216721	AC093850.1	0.506934	1.293718	-1.351653344
ENSG00000264050	RP11-22N12.2	0.071183	0.181663	-1.351659969
ENSG00000111300	NAA25	8.746224393	22.33766578	-1.352746168
ENSG00000143367	TUFT1	4.485452271	11.45814725	-1.353048417
ENSG00000003989	SLC7A2	1.018392161	2.602030275	-1.353344529

ENSG00000130203	APOE	0.213208085	0.544763356	-1.353367514
ENSG00000262979	CTD-2047H16.2	0.466094	1.191102	-1.353604117
ENSG00000267761	CTD-2130O13.1	0.251136829	0.641995876	-1.354090417
ENSG00000269924	RP11-697N18.4	0.163788	0.418858	-1.354631567
ENSG00000169427	KCNK9	0.080079755	0.204852658	-1.355077156
ENSG00000101255	TRIB3	6.13236097	15.69201019	-1.355515651
ENSG00000249889	ALG1L11P	0.029609	0.075773	-1.355648102
ENSG00000131471	AOC3	0.927300996	2.375997173	-1.357423511
ENSG00000250751	RP11-613C6.2	0.081434	0.208703	-1.357748165
ENSG00000117595	IRF6	8.192748477	20.99933606	-1.357924286
ENSG00000205100	HSP90AA4P	2.806918	7.194989	-1.358005563
ENSG00000186047	DLEU7	0.076567401	0.196291453	-1.358195168
ENSG00000172345	STARD5	0.429582626	1.101747553	-1.358786142
ENSG00000260233	SSSCA1-AS1	0.119509314	0.306553253	-1.359014655
ENSG00000276988	Metazoa_SRP	0.993687	2.549249	-1.359208901
ENSG00000256913	RP1-102E24.6	0.273868	0.702872	-1.359781279
ENSG00000277595	RP11-946P6.6	0.438855	1.126462	-1.359982397
ENSG00000179361	ARID3B	1.10872178	2.846461173	-1.360272036
ENSG00000260361	CTD-2313J17.5	0.024041	0.061728	-1.360428141
ENSG00000164142	FAM160A1	1.658051251	4.257257159	-1.360435637
ENSG00000214534	ZNF705E	0.143590937	0.368721392	-1.36056643
ENSG00000273245	RP11-434P11.2	0.766798	1.969131	-1.360640613
ENSG00000228808	HMGB3P4	0.491138	1.261409	-1.360835777
ENSG00000231563	RP11-245P10.4	0.133085	0.34198	-1.36156398
ENSG00000252059	AC012667.1	0.025565	0.065697	-1.361657467
ENSG00000239665	RP11-295P9.3	0.605985908	1.558560875	-1.362858357
ENSG00000197261	C6orf141	1.619281305	4.165609814	-1.363174075
ENSG00000249540	RP11-789L4.1	0.195998	0.504229	-1.363240162
ENSG00000181381	DDX60L	3.200215807	8.233697765	-1.363371296
ENSG00000267984	CTD-2616J11.9	0.234588	0.603574	-1.363401444
ENSG00000276788	SNORD26	0.281523	0.724823	-1.364375942
ENSG00000196565	HBG2	0.447012346	1.151074617	-1.364594773
ENSG00000281201	AC007279.1	1.042354	2.684379	-1.364743053
ENSG00000186526	CYP4F8	0.01444266	0.037197048	-1.36485164
ENSG00000130649	CYP2E1	0.088133352	0.227062393	-1.365328801
ENSG00000197483	ZNF628	0.706114207	1.819664733	-1.365699213
ENSG00000164300	SERINC5	5.619319116	14.49004116	-1.366594456
ENSG00000100191	SLC5A4	0.313028	0.807194	-1.36662374
ENSG00000281730	AC078950.1	0.364613	0.940287	-1.366735171
ENSG00000211591	MIR762	0.129789	0.334767	-1.366989204
ENSG00000261101	RP4-545K15.5	0.5431	1.400864	-1.367027133
ENSG00000178038	ALS2CL	1.340755908	3.458905972	-1.367269185
ENSG00000166012	TAF1D	11.91144458	30.75586075	-1.368512964
ENSG00000237721	AF064858.11	0.195985	0.506118	-1.368730545
ENSG00000280933	AC105339.3	0.19209	0.496259	-1.369310851
ENSG00000118292	C1orf54	0.303035536	0.782926914	-1.369390656
ENSG00000235725	AC007389.3	0.061468	0.158832	-1.369594153
ENSG00000243024	RPS11P6	0.024281639	0.062777733	-1.37038714

ENSG00000198590	C3orf35	0.704016905	1.820374962	-1.370553672
ENSG00000276252	MIR6510	0.640916	1.658439	-1.371618757
ENSG00000203325	RP11-277A4.4	0.128173	0.331769	-1.372086703
ENSG00000184471	C1QTNF8	0.056934889	0.147378624	-1.3721424
ENSG00000172519	OR10H5	0.036569	0.094663	-1.372179468
ENSG00000186723	OR10H1	0.036828	0.095337	-1.372233177
ENSG00000078319	PMS2P1	2.459211	6.36628	-1.372255087
ENSG00000243260	RN7SL558P	0.289376	0.749362	-1.372717547
ENSG00000068028	RASSF1	3.426322828	8.874549588	-1.373012812
ENSG00000254461	RP11-755F10.3	0.822169	2.12993	-1.373299136
ENSG00000276877	AL662801.1	1.856007	4.808266	-1.373314559
ENSG00000258479	LINC00640	1.045520894	2.708737709	-1.373398809
ENSG00000277883	NLRP3P	0.114053	0.295616	-1.374019961
ENSG00000224063	AC007319.1	0.117559249	0.304759626	-1.374283745
ENSG00000267234	CTB-184G21.3	0.085522	0.221805	-1.37492439
ENSG00000275927	RP11-567P19.1	0.150125	0.389356	-1.374925613
ENSG00000279623	RP11-399C16.3	0.086706	0.224964	-1.375490416
ENSG00000091138	SLC26A3	0.015049011	0.039046681	-1.375531221
ENSG00000260342	RP11-1035H13.3	15.710198	40.765549	-1.375649081
ENSG00000166762	CATSPER2	0.221157966	0.574349548	-1.376851821
ENSG00000066322	ELOVL1	14.55226464	37.84443151	-1.378837351
ENSG00000276203	ANKRD20A3	0.227033922	0.59093767	-1.380098099
ENSG00000224138	AC000123.4	2.234156	5.816614	-1.380449642
ENSG00000206639	Y_RNA	0.377121	0.981888	-1.380530982
ENSG00000118503	TNFAIP3	1.541099656	4.0139094	-1.381047896
ENSG00000006534	ALDH3B1	0.748363372	1.949407106	-1.381224555
ENSG00000179111	HES7	0.16308954	0.424871284	-1.381361586
ENSG00000126368	NR1D1	25.628601	66.833015	-1.382806231
ENSG00000248890	HHIP-AS1	0.06537697	0.170512177	-1.383020353
ENSG00000137473	TTC29	0.049797063	0.129941748	-1.383732455
ENSG00000215529	EFCAB8	0.044433204	0.115971897	-1.384065159
ENSG00000279605	RP11-661A12.8	0.071863	0.187658	-1.384784726
ENSG00000232545	KB-318B8.7	0.440185	1.150052	-1.385517206
ENSG00000222225	RNU6-447P	0.140944	0.368436	-1.386291971
ENSG00000267253	RP11-209M4.1	0.149196	0.39029	-1.387337642
ENSG00000238228	OR7E7P	0.054483	0.14261	-1.388197099
ENSG00000186472	PCLO	0.308373913	0.807215661	-1.388273441
ENSG00000251224	CNOT10-AS1	0.218438	0.571886	-1.388503735
ENSG00000257279	RP11-231I16.1	0.169331	0.443576	-1.389335193
ENSG00000243636	RP11-164O23.7	0.204252	0.535278	-1.389938154
ENSG00000269246	CTC-246B18.10	0.835521	2.189848	-1.39008274
ENSG00000123360	PDE1B	0.026723988	0.070048273	-1.390214137
ENSG00000233452	STXBP5-AS1	0.015240896	0.039949568	-1.3902322
ENSG00000258919	RP11-1029J19.4	0.139752	0.366646	-1.39151887
ENSG00000280721	AC133644.2	0.166739641	0.437853607	-1.392851464
ENSG00000124107	SLPI	3.296298	8.65747	-1.393098809
ENSG00000279560	AC007326.1	0.22893	0.601937	-1.394705967
ENSG00000214076	CPSF1P1	0.528134	1.388852	-1.394916943

ENSG00000244563	AC006011.4	0.95618	2.517857	-1.396842214
ENSG00000149243	KLHL35	0.081536743	0.214720715	-1.396939144
ENSG00000184206	GOLGA6L4	0.551708351	1.453286326	-1.397341247
ENSG00000235440	RP11-214J9.1	0.124137	0.327363	-1.398958085
ENSG00000272034	SNORD14A	3.098516	8.171605	-1.399042051
ENSG00000276500	BMS1P14	0.166824	0.439991	-1.399147158
ENSG00000196712	NF1	1.712175132	4.516434413	-1.399353981
ENSG00000257065	RP3-468K18.5	0.464782	1.226238	-1.399612916
ENSG00000281137	AC074033.1	1.269012	3.348257	-1.399704557
ENSG00000263847	RP11-143J12.3	1.301435	3.434345	-1.399931715
ENSG00000173083	HPSE	2.714831308	7.165360247	-1.400176684
ENSG00000173295	FAM86B3P	1.121398359	2.959762144	-1.400182378
ENSG00000135362	PRR5L	0.038464222	0.101542635	-1.400496579
ENSG00000208797	SNORD73A	3.257869	8.606486	-1.401495716
ENSG00000188739	RBM34	4.751791739	12.55683335	-1.401929171
ENSG00000224072	RP11-75A9.3	0.110692	0.292609	-1.402423186
ENSG00000215859	RP6-74O6.2	0.458985022	1.213366933	-1.402496918
ENSG00000262652	RP13-638C3.2	1.650614	4.365359	-1.403097522
ENSG00000261459	AC002310.11	0.066347	0.175474	-1.403154144
ENSG00000125731	SH2D3A	6.660098758	17.61818742	-1.403450031
ENSG00000167945	PRR25	0.042097	0.111393	-1.403869246
ENSG00000183775	KCTD16	0.019515998	0.051664174	-1.404506947
ENSG00000110931	CAMKK2	2.613344305	6.918815083	-1.404627773
ENSG00000259732	RP11-59H7.3	0.16081	0.425782	-1.404757837
ENSG00000258279	LINC00592	0.097502	0.258161	-1.404767354
ENSG00000229368	AC090587.4	0.193154	0.511424	-1.40476831
ENSG00000159186	AC007383.6	0.080025	0.211887	-1.404772399
ENSG00000165272	AQP3	3.298244303	8.733615146	-1.404880695
ENSG00000254873	RP11-770J1.5	0.034486075	0.09132373	-1.404975855
ENSG00000243960	RP11-552M11.4	0.36890367	0.977017967	-1.405140952
ENSG00000267756	RP11-411B10.4	0.167406	0.443398	-1.405251026
ENSG00000236591	RP11-162J8.3	0.234905	0.622283	-1.405493412
ENSG00000149295	DRD2	0.022852377	0.060546754	-1.405705354
ENSG00000246640	RP11-1094H24.4	0.322385	0.854352	-1.406045975
ENSG00000272211	RP11-347P5.1	0.311233	0.825424	-1.407140349
ENSG00000185565	LSAMP	0.019708581	0.052287679	-1.407647156
ENSG00000278264	MIR6803	0.360188	0.95562	-1.407686931
ENSG00000137558	PI15	0.071123775	0.188771639	-1.408238224
ENSG00000228737	AC008781.7	0.081958	0.217837	-1.410292335
ENSG00000269405	NXF2	0.007241408	0.019248581	-1.410409884
ENSG00000232586	KIAA1614-AS1	0.014138	0.037583	-1.410502185
ENSG00000177369	FLJ40194	0.019827635	0.052707914	-1.410506989
ENSG00000269437	NXF2B	0.004386394	0.011660516	-1.410524324
ENSG00000197617	VN1R5	0.094956	0.252428	-1.410540879
ENSG00000274967	Y_RNA	0.454431	1.208047	-1.410543425
ENSG00000243094	RP11-1084A12.1	0.251962	0.669809	-1.410543593
ENSG00000267939	CTD-2325M2.1	0.111222	0.29567	-1.410545683
ENSG00000278477	RP11-282M24.1	0.223229	0.593427	-1.410546107

ENSG00000280085	RP11-678K21.1	0.247069	0.656803	-1.410546711
ENSG00000224020	MIR181A2HG	0.153765	0.408766	-1.410548048
ENSG00000253013	SNORA31	0.150803	0.400892	-1.410548499
ENSG00000274300	MIR6864	0.290835	0.773151	-1.410549309
ENSG00000281350	Metazoa_SRP	0.154817	0.411563	-1.41054939
ENSG00000259449	NIFKP8	0.116467	0.309614	-1.410549472
ENSG00000225572	DOCK4-AS1	0.040815	0.108502	-1.410550273
ENSG00000274315	RP11-996F15.5	0.20237	0.537978	-1.410551741
ENSG00000263627	PPP4R1-AS1	0.053716	0.142798	-1.410551991
ENSG00000167914	GSDMA	0.045646814	0.121347028	-1.410552688
ENSG00000163810	TGM4	0.018701536	0.049716046	-1.410554788
ENSG00000264589	MAPT-AS1	0.021755196	0.057833939	-1.410556388
ENSG00000266237	RP11-25D3.1	0.024265	0.064506	-1.410556498
ENSG00000163464	CXCR1	0.040913	0.108763	-1.410556617
ENSG00000273361	RP11-378A13.2	0.08536	0.226921	-1.410558046
ENSG00000136487	GH2	0.035498258	0.094368675	-1.410559812
ENSG00000249807	CTD-2199O4.1	0.053972	0.14348	-1.410566596
ENSG00000235436	DPY19L2P4	0.025221	0.067048	-1.410568819
ENSG00000130561	SAG	0.008564533	0.0227685	-1.410592758
ENSG00000122012	SV2C	0.008462491	0.022497528	-1.410612231
ENSG00000092850	TEKT2	0.010291807	0.027361638	-1.41065831
ENSG00000128510	CPA4	23.67135067	62.95853064	-1.411259844
ENSG00000105251	SHD	0.110463065	0.293802752	-1.411283851
ENSG00000215156	RP11-1023L17.2	0.196376	0.522446	-1.411663306
ENSG00000259772	RP11-16E12.2	0.207282651	0.551593786	-1.412006831
ENSG00000218980	FTH1P15	46.699245	124.354149	-1.412983512
ENSG00000187904	AC097382.5	0.167071	0.445053	-1.413515819
ENSG00000251451	GTF2IP9	0.36705	0.978295	-1.414292966
ENSG00000224897	POT1-AS1	0.035447006	0.094584196	-1.415935355
ENSG00000165030	NFIL3	14.766979	39.409611	-1.416172797
ENSG00000279504	AD001527.4	0.098093	0.261894	-1.416760915
ENSG00000281021	RP1-12G14.9	0.55723	1.489455	-1.4184397
ENSG00000265693	RP11-815I9.5	0.109622	0.293018	-1.41845193
ENSG00000270392	PFN1P2	0.110518445	0.295482441	-1.418785237
ENSG00000230789	ARHGAP26-IT1	0.102317	0.273679	-1.419438869
ENSG00000100483	VCPKMT	4.420932676	11.8260481	-1.41954538
ENSG00000260037	CTD-2524L6.3	0.11247169	0.301319776	-1.421733454
ENSG00000271401	RP11-171I2.3	0.109927	0.294565	-1.42204024
ENSG00000137672	TRPC6	0.009191515	0.024631897	-1.422153143
ENSG00000248234	CTD-2108O9.4	0.177853	0.476824	-1.422771543
ENSG00000234324	RPL9P2	0.32765	0.878847	-1.423456494
ENSG00000246130	RP11-875O11.2	0.086887	0.233109	-1.423792463
ENSG00000229598	PRDX3P1	2.252641	6.044051	-1.423898422
ENSG00000226648	PLCG1-AS1	0.213319	0.572628	-1.424585746
ENSG00000267048	RP11-566K11.7	0.583995	1.56811	-1.424998843
ENSG00000116031	CD207	0.053919	0.144784	-1.425036535
ENSG00000278935	RP11-173D3.4	0.032599	0.087536	-1.425048752
ENSG00000185950	IRS2	2.80367	7.530171	-1.425366077

ENSG00000214435	AS3MT	0.564855	1.518107151	-1.426321147
ENSG00000213949	ITGA1	0.235699949	0.633703802	-1.426857228
ENSG00000252965	Y_RNA	0.960306	2.583489	-1.427754644
ENSG00000243989	ACY1	1.600922993	4.307427452	-1.427922584
ENSG00000241670	RPL23AP21	0.663144	1.784565	-1.428178363
ENSG00000112599	GUCA1B	2.253975	6.066137	-1.428306567
ENSG00000160318	CLDND2	0.243701974	0.655923324	-1.42840924
ENSG00000100027	YPEL1	0.359695535	0.968267763	-1.42862981
ENSG00000254416	RP11-347E10.1	0.381883944	1.028187657	-1.42889743
ENSG00000198380	GFPT1	7.715233074	20.77985587	-1.429404002
ENSG00000236601	RP4-669L17.2	0.083564082	0.225286248	-1.430804377
ENSG00000238363	SNORA13	0.4286	1.155656	-1.431008265
ENSG00000257509	RP11-762I7.4	0.481061	1.297306	-1.431227064
ENSG00000236244	RP4-799P18.3	0.131176	0.353769	-1.431303845
ENSG00000179954	SSC5D	0.069448122	0.187448728	-1.432488445
ENSG00000256709	RP1-19N1.1	0.378953	1.022897	-1.432570048
ENSG00000254154	RP4-798P15.3	0.312789934	0.844670087	-1.433193875
ENSG00000200677	SNORD18	0.887049	2.396767	-1.43400396
ENSG00000197461	PDGFA	1.945870185	5.258549642	-1.434249478
ENSG00000216636	RPL7P25	0.067297	0.181903	-1.434555238
ENSG00000252311	RNU1-103P	0.538517	1.457232	-1.436166787
ENSG00000182372	CLN8	1.119481704	3.029406889	-1.436204413
ENSG00000238386	RNU7-48P	0.533199	1.443214	-1.436539259
ENSG00000179454	KLHL28	1.705040228	4.616942352	-1.43713194
ENSG00000248405	PRR5-ARHGAP8	0.610150016	1.65231612	-1.437253825
ENSG00000172466	ZNF24	6.138414892	16.63047914	-1.43789167
ENSG00000188910	GJB3	9.070877284	24.59837658	-1.439249113
ENSG00000266933	AC005775.2	0.045507	0.123523	-1.44061931
ENSG00000184434	LRRC19	0.073757	0.200347	-1.441649025
ENSG00000132832	LINC01260	0.353363	0.959913	-1.44175267
ENSG00000259000	DOCK11P1	0.01287	0.034974	-1.442270754
ENSG00000164265	SCGB3A2	0.75752501	2.059097274	-1.44264656
ENSG00000101224	CDC25B	25.35625667	68.93272955	-1.442847367
ENSG00000106415	GLCCI1	0.203528831	0.553470078	-1.443272149
ENSG00000006062	MAP3K14	2.866805186	7.798308761	-1.443717408
ENSG00000219559	RP11-346C16.4	0.071555	0.194719	-1.444269178
ENSG00000252481	SCARNA13	0.543698	1.480005	-1.444724624
ENSG00000227417	RP11-397P13.6	0.162926	0.44357	-1.444944945
ENSG00000138347	MYPN	0.050463299	0.137507778	-1.446206803
ENSG00000277194	SNORD22	1.56042	4.253537	-1.446728609
ENSG00000258230	RP11-511H9.3	0.367848125	1.003168511	-1.447381827
ENSG00000198870	STKLD1	0.052914178	0.144386759	-1.448212206
ENSG00000255946	RP11-173P15.7	0.066348	0.181044	-1.448215482
ENSG00000115232	ITGA4	0.006441761	0.01758256	-1.448618208
ENSG00000278985	RP11-303E16.9	0.547363	1.495019	-1.449593998
ENSG00000260260	SNHG19	13.485596	36.845188	-1.450056932
ENSG00000260051	LA16c-390E6.4	0.710761	1.943616	-1.451306787
ENSG00000175793	SFN	79.914757	218.638779	-1.452015468

ENSG00000268499	CTB-102L5.8	0.226205	0.618878	-1.452024219
ENSG00000196361	ELAVL3	0.083169214	0.227577041	-1.452233517
ENSG00000257342	RP11-571M6.7	0.238227	0.651951	-1.452426603
ENSG00000262410	RP11-388C12.8	0.182339	0.499055	-1.452575653
ENSG00000066405	CLDN18	0.100838928	0.276281897	-1.45408835
ENSG00000160284	SPATC1L	0.042322684	0.11598907	-1.45448583
ENSG00000279481	RP11-181K12.1	0.062083	0.170185	-1.454833706
ENSG00000273013	CTD-2002J20.1	0.391661	1.073899	-1.455180932
ENSG00000213711	PHBP7	0.129325	0.354598	-1.455183208
ENSG00000235448	LURAP1L-AS1	0.0637	0.174757	-1.455984967
ENSG00000252620	RNU6ATAC24P	0.377009	1.034304	-1.455989412
ENSG00000279706	RP11-561O23.9	0.133382	0.365966	-1.456145635
ENSG00000280099	RP11-33E12.2	0.411438	1.12906	-1.456375204
ENSG00000232290	RP1-292B18.3	0.215923	0.59307	-1.457685469
ENSG00000280107	AL022393.9	0.234905	0.645445	-1.458216743
ENSG00000108846	ABCC3	2.809119097	7.722577682	-1.458964687
ENSG00000107960	OBFC1	19.49876572	53.60960315	-1.459108652
ENSG00000270257	RP11-17P16.2	1.385045	3.80914	-1.459532463
ENSG00000260799	KRT8P50	1.160952	3.193413	-1.459790822
ENSG00000264190	MIR5006	0.128526	0.353539	-1.459809135
ENSG00000270510	RP11-777F6.3	0.692689	1.905442	-1.459846026
ENSG00000273890	RP11-603J24.21	0.332297	0.914681	-1.460795415
ENSG00000263393	RP11-53I6.4	0.27611	0.76026	-1.461249748
ENSG00000165591	FAAH2	1.054854337	2.904681796	-1.461336333
ENSG00000199405	SNORA1	0.945215	2.604296	-1.462179003
ENSG00000151743	AMN1	7.090264295	19.53589807	-1.462216267
ENSG00000274162	SNX18P1Y	0.086487	0.2383	-1.462223751
ENSG00000131127	ZNF141	0.027468435	0.075690406	-1.462335754
ENSG00000262482	LA16c-321D4.2	1.939106	5.343901	-1.462501611
ENSG00000265610	CR536603.1	0.033994	0.093715	-1.462999853
ENSG00000229091	HSPA8P8	0.017472	0.048213	-1.464377441
ENSG00000221649	MIR1233-1	0.260688	0.719407	-1.464484023
ENSG00000221065	MIR1233-2	0.260688	0.719407	-1.464484023
ENSG00000115641	FHL2	8.194046556	22.62294017	-1.465138445
ENSG00000213533	TMEM110	1.695456498	4.681207488	-1.465206944
ENSG00000242737	RP11-562A8.1	0.180523	0.498492	-1.465387693
ENSG00000233056	ERVH48-1	0.089543183	0.247560608	-1.467126263
ENSG00000272814	RP11-15I20.1	0.185753	0.513553	-1.467127663
ENSG00000262228	RP11-676J12.4	0.145003	0.400892	-1.467130879
ENSG00000229786	SNRPFP2	0.405547	1.121223	-1.467132217
ENSG00000258520	RP11-363J20.2	0.044028	0.121725	-1.467132285
ENSG00000256021	TCEB1P31	0.108985	0.301314	-1.467138121
ENSG00000189099	PRSS48	0.051462	0.142279	-1.467143308
ENSG00000248483	POU5F2	0.005612404	0.015525893	-1.467985576
ENSG00000235095	RP11-1217F2.1	0.524128	1.450404	-1.468463721
ENSG00000231649	SPATA31B1P	0.006083	0.016846	-1.469551162
ENSG00000277631	PGM5P3-AS1	0.026807208	0.07430435	-1.470825703
ENSG00000276924	FP236383.9	0.103267	0.28629	-1.471097978

ENSG00000277765	AL353644.11	0.103267	0.28629	-1.471097978
ENSG00000269898	RP11-568J23.4	0.26402	0.732345	-1.471876227
ENSG00000189334	S100A14	65.98718994	183.0436871	-1.471930131
ENSG00000101203	COL20A1	0.013821123	0.038368686	-1.473054506
ENSG00000239636	RP4-728D4.2	0.183819414	0.51041623	-1.473385063
ENSG00000261205	NIFKP4	0.053373	0.148348	-1.474803467
ENSG00000266459	MIR4724	0.190621	0.52991	-1.475040289
ENSG00000223356	RP11-66D17.5	1.175432	3.269191	-1.475742588
ENSG00000214456	PLIN5	0.445573944	1.239717455	-1.476274578
ENSG00000124635	HIST1H2BJ	0.21007334	0.584628383	-1.476626788
ENSG00000118523	CTGF	11.824718	32.932846	-1.477721414
ENSG00000258940	RP11-407N17.5	0.074903	0.208677	-1.478176191
ENSG00000223044	RNU6-130P	0.494138	1.376659	-1.478185336
ENSG00000233217	MROH3P	0.019389	0.054018	-1.478201828
ENSG00000109736	MFSD10	6.880721192	19.18400622	-1.47927234
ENSG00000196668	LINC00173	1.314709378	3.665933787	-1.479436808
ENSG00000149571	KIRREL3	0.023138616	0.064544704	-1.479996132
ENSG00000267162	SDHDP1	0.143168	0.399624	-1.480934162
ENSG00000267302	RP11-178C3.2	0.723218537	2.02001792	-1.481864531
ENSG00000140993	TIGD7	0.064810979	0.181045846	-1.48204494
ENSG00000153495	TEX29	0.249555462	0.697383392	-1.482591526
ENSG00000143365	RORC	0.047351637	0.132355668	-1.482933777
ENSG00000279998	CH507-210P18.1	0.115137	0.321858	-1.483072801
ENSG00000279300	CTD-3149D2.2	0.756597	2.115069	-1.483107768
ENSG00000254548	RP11-429J17.5	0.741844	2.074248	-1.483400651
ENSG00000228140	RP3-467K16.4	0.022411	0.062712	-1.484534506
ENSG00000281155	AC096587.1	0.264939	0.741378	-1.484549075
ENSG00000142233	NTN5	0.491347788	1.375441326	-1.485078132
ENSG00000207524	RNU6-33P	0.518249	1.450802	-1.485133307
ENSG00000160321	ZNF208	0.007844481	0.021962441	-1.485288564
ENSG00000223732	RP11-321C24.1	0.161575	0.452474	-1.485630903
ENSG00000224271	RP11-191L9.4	0.214504132	0.601093874	-1.486584877
ENSG00000135480	KRT7	0.709164668	1.988043165	-1.487156517
ENSG00000249631	RP11-281P23.2	0.050492244	0.141599957	-1.487687143
ENSG00000175874	CREG2	0.175184084	0.491340435	-1.487851266
ENSG00000281205	LINC00950	0.282966	0.793651	-1.487876021
ENSG00000163406	SLC15A2	0.293083075	0.822072995	-1.487956843
ENSG00000065802	ASB1	3.024328527	8.485872358	-1.488448113
ENSG00000255185	PDXDC2P	0.304126942	0.853431682	-1.48860204
ENSG00000279432	RP11-135N5.3	0.292792	0.821689	-1.488716318
ENSG00000224827	LINC00265-2P	0.016272	0.045677	-1.489076315
ENSG00000196878	LAMB3	7.570225882	21.25099314	-1.489122012
ENSG00000249212	ATP1B1P1	0.166872	0.468453	-1.489162409
ENSG00000204323	SMIM5	0.059081626	0.16589292	-1.489470881
ENSG00000228237	EFCAB14-AS1	0.644770909	1.810724315	-1.489708352
ENSG00000181016	LSMEM1	0.560296454	1.573678408	-1.489878478
ENSG00000271810	RP11-426L16.10	1.857102656	5.215963767	-1.489880282
ENSG00000137819	PAQR5	7.707745426	21.68218575	-1.492129372

ENSG00000231583	AC108938.3	0.561356	1.579842	-1.492792389
ENSG00000253961	RP11-363L24.3	0.059088137	0.166371584	-1.493468626
ENSG00000264954	PRR29-AS1	0.031657	0.089153	-1.493758802
ENSG00000180712	RP11-290F5.2	0.051696705	0.145594223	-1.493808877
ENSG00000264924	RP11-799B12.2	0.162011	0.45638	-1.494143799
ENSG00000251229	RP11-503N18.5	0.849225	2.39252	-1.494312237
ENSG00000140795	MYLK3	0.028222457	0.079572363	-1.49542386
ENSG00000232368	FTLP2	21.479231	60.572151	-1.495712302
ENSG00000222009	BTBD19	0.950438204	2.680543551	-1.495860842
ENSG00000208308	SNORA40	3.702169	10.448082	-1.496795466
ENSG00000033867	SLC4A7	3.999611453	11.31234445	-1.499966195
ENSG00000253981	ALG1L13P	0.314553623	0.889904018	-1.500343763
ENSG00000262180	OCLM	0.670172	1.897892	-1.501794581
ENSG00000158869	FCER1G	0.236530584	0.669947343	-1.502020967
ENSG00000268764	CTC-450M9.1	0.246449	0.698238	-1.502429747
ENSG00000202335	SNORD50	0.530751	1.504814	-1.503478087
ENSG00000177699	RP11-16K12.1	0.009644	0.027345	-1.503573506
ENSG00000232843	SNX18P2	0.031496	0.089308	-1.503620797
ENSG00000074706	IPCEF1	0.018018622	0.051093414	-1.503648659
ENSG00000229905	RP11-206L10.4	0.167422	0.474741	-1.503651534
ENSG00000282855	RP11-50D9.4	0.495742	1.405728	-1.503656072
ENSG00000086717	PPEF1	0.043463527	0.123246545	-1.503670048
ENSG00000235831	BHLHE40-AS1	0.053949176	0.153100136	-1.504802748
ENSG00000272842	RP11-513M16.7	1.465318	4.15955	-1.50521367
ENSG00000196946	ZNF705A	0.02974053	0.084470533	-1.506017804
ENSG00000166473	PKD1L2	0.228596006	0.649520271	-1.506574358
ENSG00000132958	TPTE2	0.102065881	0.290221711	-1.507654777
ENSG00000182324	KCNJ14	0.33309329	0.947300468	-1.507895804
ENSG00000105609	LILRB5	0.023887287	0.067962456	-1.508495012
ENSG00000139725	RHOF	5.736726419	16.3225133	-1.508563594
ENSG00000124145	SDC4	39.478355	112.450508	-1.510156397
ENSG00000243323	PTPRVP	0.068395542	0.194840829	-1.510321818
ENSG00000212363	SNORA40	1.190362	3.393809	-1.511504997
ENSG00000135549	PKIB	2.672028035	7.624998908	-1.512801986
ENSG00000118596	SLC16A7	0.198248036	0.566433966	-1.514601209
ENSG00000268361	L34079.2	0.206039	0.589123	-1.515651435
ENSG00000280121	CTD-3137H5.4	0.1917	0.54834	-1.516220381
ENSG00000184613	NELL2	0.054097096	0.15473985	-1.51622172
ENSG00000279262	CTD-3137H5.5	0.192789	0.551456	-1.516223042
ENSG00000135709	KIAA0513	1.084450374	3.103431606	-1.516900317
ENSG00000243404	RPL35AP32	0.196966	0.564105	-1.518017109
ENSG00000272316	XXbac-BPGBPG55C20.2	0.995355	2.850902	-1.518135378
ENSG00000273828	RP11-394O2.3	0.217806	0.623907	-1.518287299
ENSG00000277877	RP11-11N7.5	1.124094545	3.224046545	-1.520109189
ENSG00000130383	FUT5	0.076310086	0.218921275	-1.5204665
ENSG00000228820	RPSAP1	0.129361	0.37134	-1.521337991
ENSG00000208772	SNORD94	0.74301	2.133218	-1.521577874
ENSG00000272815	RP11-219A15.4	0.005231	0.01502	-1.521726138

ENSG00000224729	PCOLCE-AS1	0.082335732	0.236420263	-1.521763115
ENSG00000235888	AF064858.8	0.175902	0.505176	-1.522014215
ENSG00000245711	NADK2-AS1	0.461961	1.327576	-1.522951488
ENSG00000226755	VPS25P1	0.074141	0.213073	-1.523004311
ENSG00000156194	PPEF2	0.039661793	0.114023172	-1.523505231
ENSG00000276015	KCNQ1OT1_5	0.006506	0.018716	-1.524429406
ENSG00000281394	SCARNA4	0.26829	0.771951	-1.524715993
ENSG00000244513	CTD-2013N24.2	0.491478718	1.41419757	-1.524782833
ENSG00000235066	AC009303.1	0.011886222	0.034203304	-1.524845426
ENSG00000264860	RP11-143K11.5	0.346233	0.996576	-1.525236594
ENSG00000243243	AC073130.3	1.254756177	3.61493766	-1.52656372
ENSG00000116717	GADD45A	23.73470992	68.38526022	-1.526686982
ENSG00000252213	SNORA74	0.489613	1.412508	-1.528545269
ENSG00000065923	SLC9A7	1.837752738	5.306100878	-1.529709433
ENSG00000232871	SEC1P	0.124809088	0.360445201	-1.530056956
ENSG00000274927	CH17-13I23.1	0.12787	0.369297	-1.530103714
ENSG00000260773	RP11-352G18.2	0.224712	0.649446	-1.531132405
ENSG00000171124	FUT3	0.195736093	0.565751428	-1.53125851
ENSG00000189223	PAX8-AS1	1.113163594	3.217896575	-1.531452327
ENSG00000149187	CELF1	4.577998343	13.23446319	-1.531510833
ENSG00000267065	CTD-2246P4.1	0.032996	0.095435	-1.532227318
ENSG00000228930	MTCO1P3	0.694521	2.008849	-1.532278902
ENSG00000231943	PGM5P4-AS1	0.03546788	0.102618467	-1.532705367
ENSG00000180332	KCTD4	0.080233	0.232152	-1.532802063
ENSG00000104723	TUSC3	0.156317102	0.45235284	-1.532972907
ENSG00000232430	RPL31P15	0.004624	0.013392	-1.53415813
ENSG00000256028	RP11-197N18.2	0.232597	0.674111	-1.535153678
ENSG00000207112	SNORA25	0.506868	1.469749	-1.535887806
ENSG00000214093	RP11-247I13.3	0.355271	1.030303	-1.536076845
ENSG00000222898	RN7SKP97	0.641935	1.862527	-1.536762212
ENSG00000214925	RP3-507I15.1	0.088222	0.255975	-1.536792543
ENSG00000229780	UBE2Q1-AS1	0.355012	1.030495	-1.537397809
ENSG00000066056	TIE1	0.027372019	0.079555796	-1.539265212
ENSG00000236998	RP11-203L2.3	0.081564	0.237242	-1.540355005
ENSG00000281827	AC005071.4	0.438413	1.275316	-1.540492281
ENSG00000266276	AC048380.1	0.051502	0.149871	-1.541020886
ENSG00000242279	RP11-659G9.1	0.046794	0.136324	-1.54264411
ENSG00000272254	RP11-531A24.7	0.116467	0.339337	-1.542797508
ENSG00000223553	SMPD4P1	0.013147908	0.038308273	-1.5428227
ENSG00000278662	GOLGA6L10	0.628217466	1.83046678	-1.542875633
ENSG00000273703	HIST1H2BM	0.072136	0.210232	-1.543190951
ENSG00000270016	RP11-932O9.8	0.297829	0.868025	-1.543252356
ENSG00000101236	RNF24	1.861239038	5.426028256	-1.543633209
ENSG00000261582	RP4-614O4.11	0.197988	0.577691	-1.544885027
ENSG00000280527	AL031587.1	0.655099	1.912974	-1.546032414
ENSG00000259916	RP11-407P15.2	0.02202	0.064338	-1.54685662
ENSG00000251666	ZNF346-IT1	0.220222	0.643635	-1.54728418
ENSG00000136826	KLF4	6.591579614	19.2723623	-1.547837279

ENSG00000273693	C2orf27AP1	0.014476	0.04233	-1.548017476
ENSG00000163815	CLEC3B	0.092120188	0.269377557	-1.548040392
ENSG00000279977	CTD-3222D19.4	0.055624	0.162657	-1.548053511
ENSG00000112964	GHR	0.015399702	0.045032385	-1.548060443
ENSG00000276166	CTD-2600O9.2	0.22795	0.666591	-1.54808443
ENSG00000232342	RP11-46O21.2	0.03310321	0.096880663	-1.549237634
ENSG00000147454	SLC25A37	8.791395736	25.7314316	-1.549367592
ENSG00000270441	RP11-694I15.7	0.41315	1.209329	-1.549469213
ENSG00000100968	NFATC4	0.168186076	0.493413354	-1.552738487
ENSG00000274417	MIR6515	0.419671	1.231363	-1.552925445
ENSG00000271156	RP11-642C5.1	0.301415	0.88461	-1.553290336
ENSG00000096996	IL12RB1	0.017762768	0.052131363	-1.553295141
ENSG00000275910	RP11-680G24.6	0.895793	2.63109	-1.554423301
ENSG00000141738	GRB7	4.406454696	12.94542229	-1.55475175
ENSG00000158710	TAGLN2	17.81097836	52.328951	-1.554842572
ENSG00000100330	MTMR3	1.377558234	4.04831581	-1.555208531
ENSG00000282924	RP11-104C4.6	0.102821	0.302232	-1.555521471
ENSG00000138678	GPAT3	9.593451574	28.24471176	-1.557858905
ENSG00000259677	RP11-493E3.1	0.171928	0.506382	-1.558421604
ENSG00000231556	RSL24D1P3	1.248928	3.678738	-1.558520622
ENSG00000260060	RP11-388M20.1	0.175968	0.518466	-1.558936286
ENSG00000212722	KRTAP4-9	0.080941	0.238561	-1.559415632
ENSG00000205359	SLCO6A1	0.051379258	0.151434609	-1.559437001
ENSG00000235244	DANT2	0.113379	0.334251	-1.559778427
ENSG00000215972	AL360176.1	0.366926	1.082411	-1.560687365
ENSG00000223547	ZNF844	0.017512	0.051705	-1.56195994
ENSG00000221444	AC139426.1	0.110378	0.326028	-1.562543222
ENSG00000228251	AC012442.6	0.069273	0.204615	-1.562546852
ENSG00000162753	SLC9C2	0.00372723	0.011009651	-1.562592872
ENSG00000265656	RP11-13N13.6	0.668832	1.976338	-1.563113923
ENSG00000162775	RBM15	4.994524128	14.760579	-1.563330183
ENSG00000180662	RPL21P8	0.095133	0.281224	-1.563701942
ENSG00000223624	RP1-125I3.4	0.363544	1.074679	-1.56370391
ENSG00000152049	KCNE4	0.012341446	0.03648296	-1.563711316
ENSG00000268460	DKFZp434J0226	0.024281389	0.071781846	-1.563768064
ENSG00000182752	PAPPA	0.022038368	0.06518962	-1.564624883
ENSG00000272288	RP11-140K17.3	1.056211525	3.125597625	-1.565233275
ENSG00000244270	RPL32P29	2.474881	7.323958	-1.56526436
ENSG00000273879	MIR6511B1	0.01722	0.050986	-1.566016016
ENSG00000273340	MICE	0.353222	1.047433	-1.568210856
ENSG00000135452	TSPAN31	1.744029627	5.176726247	-1.56961548
ENSG00000277737	FP325317.1	0.127553	0.378763	-1.570198575
ENSG00000276790	RP11-290H9.5	0.113103	0.335921	-1.570484791
ENSG00000163596	ICA1L	0.079744267	0.236895801	-1.570799918
ENSG00000243686	RPLP1P11	3.050787	9.062981	-1.570804203
ENSG00000269228	CTD-3214H19.12	1.695058	5.041009	-1.572377891
ENSG00000099840	IZUMO4	0.157564248	0.46866941	-1.572630411
ENSG00000274840	RP11-320G10.1	0.150137	0.447013	-1.574037228

ENSG00000134970	TMED7	46.28734803	137.8222897	-1.574119418
ENSG00000280976	Metazoa_SRP	0.070089	0.208959	-1.575959952
ENSG00000241984	RPL7AP2	0.137557	0.410148	-1.576115036
ENSG00000109321	AREG	51.90974122	154.8582938	-1.576871451
ENSG00000230002	ALMS1-IT1	0.887947	2.64948	-1.577163765
ENSG00000279069	RP11-159D12.6	3.336152	9.959521	-1.577891336
ENSG00000249249	AC010226.4	0.634714611	1.896511615	-1.579168249
ENSG00000227300	KRT16P2	0.033930961	0.101505436	-1.580882809
ENSG00000239152	RNA5SP310	0.402197	1.203715	-1.581519624
ENSG00000280067	CTD-2600H12.2	0.440924	1.320127	-1.582074816
ENSG00000222108	RNA5SP317	0.610608	1.829367	-1.583026135
ENSG00000152463	OLAH	0.021529893	0.064559782	-1.584294524
ENSG00000104808	DHDH	1.542424151	4.627639642	-1.585076979
ENSG00000100784	RPS6KA5	0.672949472	2.019499274	-1.585427537
ENSG00000130477	UNC13A	3.042033616	9.133775035	-1.586175161
ENSG00000221887	HMSD	0.124420998	0.37359424	-1.586242231
ENSG00000207770	MIR568	1.521529	4.568913	-1.58632914
ENSG00000235286	AP000925.2	0.091044	0.273404	-1.586398502
ENSG00000250337	LINC01021	1.542631976	4.637087326	-1.587824975
ENSG00000158246	FAM46B	4.109524	12.359503	-1.588577527
ENSG00000261780	CTD-2354A18.1	1.04700821	3.149743554	-1.588961617
ENSG00000236528	RP1-125I3.2	0.069759	0.209888	-1.589168421
ENSG00000274211	SOCS7	2.977753521	8.960137578	-1.589296542
ENSG00000073737	DHRS9	0.081434284	0.245057924	-1.589414588
ENSG00000272054	RP11-423P10.2	0.760609	2.29028	-1.590297072
ENSG00000088726	TMEM40	1.866189239	5.620823365	-1.590686191
ENSG00000272543	MIR4787	0.072709	0.219059	-1.59111363
ENSG00000237916	RP11-537E18.1	1.571072	4.733371	-1.591118706
ENSG00000265019	NCOR1P2	0.086558	0.260785	-1.59112182
ENSG00000276592	PHBP6	0.129506	0.390182	-1.591128285
ENSG00000273777	CEACAM20	0.087184059	0.26283575	-1.592025245
ENSG00000255843	AP000593.7	0.678065	2.046116	-1.593392454
ENSG00000078295	ADCY2	0.002902026	0.008763873	-1.594508004
ENSG00000280287	RP13-554M15.7	0.292662	0.884349	-1.595380393
ENSG00000135318	NT5E	6.536785413	19.76957931	-1.596628928
ENSG00000162645	GBP2	0.272219388	0.823363648	-1.596759934
ENSG00000131634	TMEM204	0.024366539	0.073699858	-1.59676048
ENSG00000240023	RP11-561B11.1	0.103509	0.313089	-1.596816608
ENSG00000212464	SNORA12	4.501026	13.614813	-1.596851364
ENSG00000186577	C6orf1	2.748039613	8.314750522	-1.597270176
ENSG00000125772	GPCPD1	4.50698765	13.64132477	-1.597748355
ENSG00000215946	MIR941-1	0.32517	0.984693	-1.598479841
ENSG00000281183	NPTN-IT1	2.831283	8.580108	-1.599539847
ENSG00000243414	TICAM2	0.04746909	0.143857685	-1.599581982
ENSG00000139278	GLIPR1	0.699131321	2.119667505	-1.600202604
ENSG00000239884	RN7SL608P	1.762213	5.345073	-1.600821337
ENSG00000121671	CRY2	1.597021402	4.848707957	-1.602216714
ENSG00000274943	RP11-351C21.2	0.17703	0.537491	-1.602246734

ENSG00000227033	AC105461.1	0.080513	0.244498	-1.602529013
ENSG00000172264	MACROD2	0.063422964	0.1926472	-1.602884021
ENSG00000249360	CTD-2233C11.3	0.020091	0.061038	-1.603158317
ENSG00000262120	RP11-510M2.7	0.209393	0.636632	-1.604246461
ENSG00000255050	RP11-661A12.9	0.3005	0.914215	-1.605168499
ENSG00000252835	SCARNA21	0.739643	2.25048	-1.605331739
ENSG00000131791	PRKAB2	4.878295186	14.854315	-1.606433114
ENSG00000172250	SERHL	0.845735862	2.576191458	-1.606960756
ENSG00000228709	AP001065.15	0.055808	0.170009	-1.607067272
ENSG00000234350	AC007405.4	0.048888214	0.149011577	-1.60786581
ENSG00000269956	MKNK1-AS1	0.302537	0.923125	-1.609414422
ENSG00000230572	AC027612.3	0.052443151	0.160147112	-1.61057149
ENSG00000258949	RP11-857B24.5	0.107448	0.328331	-1.611512342
ENSG00000241607	RP11-357K9.2	0.488454	1.492775	-1.611702122
ENSG00000234061	AC007969.4	0.127906	0.391028	-1.612187975
ENSG00000234428	RP11-666F17.1	0.094123	0.287902	-1.612958601
ENSG00000132840	BHMT2	0.007997706	0.024492124	-1.614659794
ENSG00000173200	PARP15	0.009242181	0.028303361	-1.61466808
ENSG00000281467	Clostridiales-1	0.326258	0.999148	-1.614685117
ENSG00000226272	ARHGAP26-AS1	0.0514102	0.157441206	-1.614686648
ENSG00000141161	UNC45B	0.008962	0.027447	-1.614755837
ENSG00000201183	RNVU1-3	0.107483	0.32943	-1.615863447
ENSG00000266941	AC104532.3	0.097216854	0.298280171	-1.617389723
ENSG00000238039	AF011889.2	0.226591	0.695824	-1.618631881
ENSG00000101049	SGK2	0.472393646	1.451091078	-1.619076609
ENSG00000234695	AC002076.10	0.033431	0.102696	-1.619121575
ENSG00000252192	SNORA9	0.426108	1.308961	-1.61913107
ENSG00000158578	ALAS2	0.145288206	0.446812736	-1.620752713
ENSG00000205309	NT5M	0.973900397	2.997945509	-1.622128024
ENSG00000259376	RP11-505E24.3	0.060122	0.185178	-1.622947803
ENSG00000258302	RP11-981P6.1	0.265338	0.817344	-1.623112097
ENSG00000265462	MIR3680-1	0.378309	1.165354	-1.623131266
ENSG00000232536	RP11-74C1.4	0.096761	0.298081	-1.623206835
ENSG00000141441	GAREM1	1.025456285	3.159898027	-1.62361201
ENSG00000199744	SNORD36A	1.666306	5.135622	-1.623885658
ENSG00000221299	Z83826.1	0.789177	2.432384	-1.623950189
ENSG00000267412	CTC-265F19.2	0.029799	0.091891	-1.624659651
ENSG00000249056	RP11-326I11.1	0.041787	0.128859	-1.624667211
ENSG00000269275	CTD-2105E13.15	0.186092	0.573856	-1.624672725
ENSG00000237892	KLF7-IT1	0.077704	0.239618	-1.624675515
ENSG00000091129	NRCAM	0.006211755	0.019156799	-1.624783793
ENSG00000164733	CTSB	14.52592407	44.8558434	-1.626665994
ENSG00000201487	SNORD45B	0.507423	1.567467	-1.627174251
ENSG00000279386	AC021106.1	0.575099	1.777519	-1.627982747
ENSG00000104626	ERI1	8.42459194	26.05156533	-1.628691347
ENSG00000272273	XXbac-BPG252P9.10	0.069535	0.215036	-1.62876697
ENSG00000225710	RP5-875O13.6	0.062514	0.193595	-1.63079047
ENSG00000272620	AFAP1-AS1	0.052672	0.163269	-1.632142745

ENSG00000264545	RP11-145E5.5	0.606378	1.88006	-1.632489387
ENSG00000106113	CRHR2	0.051017067	0.158184659	-1.63255782
ENSG00000275072	SNORD50B	1.187979	3.685876	-1.633498203
ENSG00000136688	IL36G	0.036881337	0.114515475	-1.634579714
ENSG00000171989	LDHAL6B	0.117846	0.366344	-1.636296197
ENSG00000124171	PARD6B	5.138298619	15.97517958	-1.636469507
ENSG00000124788	ATXN1	0.675789111	2.101581061	-1.636830094
ENSG00000114861	FOXP1	0.211954717	0.659231582	-1.637029283
ENSG00000198453	ZNF568	0.005393199	0.016779998	-1.637529406
ENSG00000187800	PEAR1	1.468117418	4.571926019	-1.638834701
ENSG00000261286	RP11-517C16.2	0.178146	0.554916	-1.639209311
ENSG00000283061	ABC7-481722F1.2	0.039380694	0.122722889	-1.639843913
ENSG00000269365	RP11-380M21.4	0.111899	0.34894	-1.640781844
ENSG00000256712	RP11-785H5.2	0.13523	0.421718	-1.64086336
ENSG00000231817	LINC01198	0.052931593	0.165175214	-1.641796231
ENSG00000253667	RP11-30L15.4	0.322229	1.005976	-1.642437642
ENSG00000134330	IAH1	2.008280012	6.271601893	-1.642873547
ENSG00000267416	CTD-2319I12.2	0.062457	0.195057	-1.642960594
ENSG00000104970	KIR3DX1	0.009533465	0.029779481	-1.643246042
ENSG00000258648	UBE2CP1	0.091871	0.28721	-1.644424544
ENSG00000274206	Metazoa_SRP	0.060375	0.188748	-1.644438168
ENSG00000278153	RP4-585I14.3	0.123235	0.385265	-1.644439074
ENSG00000224085	RPL23AP41	0.037042	0.115804	-1.644451186
ENSG00000276012	RP11-397O8.7	0.080675	0.25231	-1.645003809
ENSG00000272744	RP11-367N14.3	0.054589	0.170728	-1.645017511
ENSG00000242861	RP11-285F7.2	1.416106	4.432178	-1.646086563
ENSG00000232803	SLCO4A1-AS1	0.379042065	1.186529422	-1.646318006
ENSG00000224761	RP11-508N22.9	0.032061006	0.100381043	-1.646595234
ENSG00000256514	AP003419.11	0.323094	1.011698	-1.646752834
ENSG00000254629	RP11-160H12.3	0.094676	0.296615	-1.647520898
ENSG00000238269	PAGE2B	0.281418375	0.882597383	-1.649038939
ENSG00000250770	RP5-1063M23.1	0.109838	0.344499	-1.649122534
ENSG00000269194	AC006942.4	0.097877	0.30703	-1.649337841
ENSG00000260714	RP11-266L9.1	0.0139	0.043607	-1.649474859
ENSG00000254039	RP11-304F15.6	0.257703	0.808519	-1.649572345
ENSG00000225864	HCG4P11	0.357529	1.122748	-1.65090198
ENSG00000274080	CTA-315H11.2	0.757197	2.379758	-1.652074272
ENSG00000279145	RP11-547D13.1	0.1734	0.54506	-1.652311151
ENSG00000213600	XXcos-LUCA16.1	0.127588	0.401111	-1.652508885
ENSG00000254595	CTD-2010I16.1	1.808655	5.686525	-1.652630059
ENSG00000105808	RASA4	0.502079597	1.578636503	-1.652691009
ENSG00000272072	CTA-363E19.2	1.166086	3.667359	-1.653067307
ENSG00000255021	RP11-536I6.2	0.079707	0.250738	-1.653402322
ENSG00000230201	ATP6V0CP1	0.080709	0.253964	-1.653822541
ENSG00000186684	CYP27C1	0.011333236	0.035670541	-1.654173184
ENSG00000280217	RP11-638F5.2	0.025284	0.079581	-1.654199308
ENSG00000250131	RP11-130F10.1	0.023552	0.074131	-1.654227396
ENSG00000280266	RP11-331D5.2	0.032985	0.103822	-1.654230175

ENSG00000164535	DAGLB	3.172395196	9.999448832	-1.656276073
ENSG00000143869	GDF7	0.005221	0.016468	-1.65726729
ENSG00000187733	AMY1C	0.032226	0.101697	-1.657980089
ENSG00000241723	RP11-113A11.1	0.042179	0.133139	-1.658336441
ENSG00000166783	KIAA0430	5.230764274	16.51225863	-1.658443812
ENSG00000257671	RP3-416H24.1	0.090188	0.285122	-1.660571969
ENSG00000246250	RP11-613D13.5	0.328419262	1.038557032	-1.660969796
ENSG00000276724	RP11-1000B6.7	0.723148	2.291438	-1.663890405
ENSG00000256399	RP11-274J7.3	0.041719	0.132196	-1.663902042
ENSG00000253404	AC034243.1	0.086706	0.274752	-1.663926247
ENSG00000229808	RP11-456P18.2	0.037314	0.118240023	-1.663929524
ENSG00000269506	RP11-571I18.5	0.031534	0.099925	-1.663937484
ENSG00000143839	REN	0.035173	0.111458	-1.663959874
ENSG00000228661	AC090587.5	0.206057	0.65301	-1.66406161
ENSG00000188263	IL17REL	0.013769886	0.043647811	-1.664392636
ENSG00000171097	CCBL1	2.181918369	6.919597145	-1.665090919
ENSG00000197714	ZNF460	0.995365056	3.158882736	-1.666116738
ENSG00000259736	CRTC3-AS1	0.073392461	0.23299566	-1.666599309
ENSG00000250021	C15orf38-AP3S2	0.568314336	1.804893651	-1.667152818
ENSG00000233818	AP000695.4	0.075280153	0.239305784	-1.668513808
ENSG00000202198	RN7SK	0.940016	2.9908	-1.66977422
ENSG00000251675	CTC-458I2.2	0.121866	0.387945	-1.670556455
ENSG00000185022	MAFF	3.734394197	11.89500746	-1.671410047
ENSG00000213863	XX-C2158C12.2	0.139662	0.445055	-1.672044098
ENSG00000105516	DBP	2.52870522	8.0583238	-1.67208091
ENSG00000258941	RP11-407N17.3	0.139329	0.444062	-1.672265548
ENSG00000175820	CCDC168	0.004743	0.015128	-1.673349495
ENSG00000260664	AC004158.3	0.00968959	0.030908024	-1.673473914
ENSG00000198774	RASSF9	0.008958	0.028575	-1.673504927
ENSG00000152931	PART1	0.005219217	0.016648745	-1.673508019
ENSG00000161652	IZUMO2	0.011228396	0.035817515	-1.673513338
ENSG00000244094	SPRR2F	0.024913	0.079472	-1.673547892
ENSG00000205846	CLEC6A	0.03026	0.096529	-1.673550446
ENSG00000203785	SPRR2E	0.02311412	0.073733826	-1.673552181
ENSG00000099937	SERPIND1	0.019936054	0.063595868	-1.673553176
ENSG00000105707	HPN	0.015058336	0.048036015	-1.673554129
ENSG00000204161	C10orf128	0.012344691	0.039379572	-1.67355674
ENSG00000106565	TMEM176B	0.025718322	0.082041824	-1.673563031
ENSG00000259728	LINC00933	0.00565502	0.018039601	-1.673563371
ENSG00000241644	INMT	0.045228203	0.144278851	-1.67356527
ENSG00000230537	RP11-305L7.1	0.06245	0.199217	-1.673567287
ENSG00000273487	RP4-621B10.8	0.039363	0.125569	-1.673568258
ENSG00000267526	RP11-178C3.6	0.070348	0.224412	-1.67356851
ENSG00000280587	LINC01348	0.015603	0.049774	-1.673568888
ENSG00000234283	RP11-495P10.6	0.060591	0.193287	-1.673569187
ENSG00000121318	TAS2R10	0.048845	0.155817	-1.673569849
ENSG00000257883	RP11-497G19.1	0.058101	0.185344	-1.673570513
ENSG00000226915	AC068137.13	0.058101	0.185344	-1.673570513

ENSG00000249867	RP11-115J23.1	0.033661694	0.107381909	-1.673571249
ENSG00000231613	RP5-943J3.1	0.099993	0.318981	-1.673571485
ENSG00000238086	PPP1R26P1	0.014087	0.044938	-1.673571513
ENSG00000262920	RP11-1260E13.1	0.117816194	0.375837618	-1.673571616
ENSG00000215023	AC114730.5	0.068872	0.219704	-1.673571658
ENSG00000260469	C15orf59-AS1	0.032315154	0.103086446	-1.673571868
ENSG00000166292	TMEM100	0.017514192	0.055870936	-1.673573547
ENSG00000235993	AC007559.1	0.14337	0.457356	-1.673574404
ENSG00000232377	AC016910.1	0.048658	0.155221	-1.673574827
ENSG00000171885	AQP4	0.016979729	0.054166047	-1.673575404
ENSG00000239238	MEMO1P3	0.056177	0.179207	-1.673575504
ENSG00000248493	AC005351.1	0.032377	0.103284	-1.673575561
ENSG00000131737	KRT34	0.029067	0.092725	-1.673576184
ENSG00000242242	PVRL3-AS1	0.030568346	0.097514383	-1.673576539
ENSG00000277692	RP11-358N2.2	0.074628	0.238067	-1.673578725
ENSG00000120729	MYOT	0.035185866	0.112244664	-1.673578955
ENSG00000225535	LINC01393	0.027812123	0.088722088	-1.673579417
ENSG00000248079	DPH6-AS1	0.033243594	0.106048793	-1.673579917
ENSG00000236489	RP11-537I16.2	0.340443	1.086031	-1.67358011
ENSG00000277027	RMRP	0.578366	1.845018	-1.673580242
ENSG00000254459	RP11-91P24.7	0.087002	0.277541	-1.67358044
ENSG00000200114	RNA5SP123	0.427699	1.364383	-1.673580945
ENSG00000245812	RP11-175K6.1	0.015835632	0.050516535	-1.673581279
ENSG00000237609	AF064858.10	0.174302	0.556033	-1.673581384
ENSG00000265547	RP11-293E1.2	0.098445	0.314045	-1.673581461
ENSG00000220867	HSPE1P26	0.185077	0.590406	-1.673581764
ENSG00000230604	TSEN15P2	0.142168	0.453524	-1.673582127
ENSG00000090932	DLL3	0.019440756	0.062017126	-1.673582326
ENSG00000280378	RP4-758J24.6	0.145003	0.462568	-1.673582716
ENSG00000188029	CTSL3P	0.149694506	0.47753418	-1.673582728
ENSG00000273017	AP000240.9	0.112602	0.359207	-1.673583012
ENSG00000275270	AL133391.1	0.087678	0.279698	-1.673583143
ENSG00000146006	LRRTM2	0.025051254	0.07991504	-1.673584226
ENSG00000276054	RP11-378E13.3	0.023082	0.073633	-1.673584246
ENSG00000227279	AC015933.2	0.115673	0.369004	-1.6735843
ENSG00000179331	RAB39A	0.027812	0.088722	-1.673584393
ENSG00000233548	CYCSP44	0.087808	0.280113	-1.673584648
ENSG00000271075	RP11-589M4.4	0.166082	0.529812	-1.673584798
ENSG00000279121	RP11-83B20.5	0.096394	0.307503	-1.673585231
ENSG00000252174	RNU7-18P	0.229853	0.733246	-1.6735858
ENSG00000160255	ITGB2	0.007488045	0.023887351	-1.67358591
ENSG00000241134	BET1P1	0.070886	0.226131	-1.673586155
ENSG00000255763	MRPS18CP4	0.073443	0.234288	-1.673586166
ENSG00000229468	RPL39P18	0.065672	0.209498	-1.673586174
ENSG00000259499	RP11-616K22.2	0.077704	0.247881	-1.673586922
ENSG00000272650	RP11-571I18.4	0.170792	0.544838	-1.673586929
ENSG00000131914	LIN28A	0.037334	0.119098	-1.673587193
ENSG00000236512	RP3-336K20__B.2	0.11137	0.355278	-1.673587692

ENSG00000272677	RP11-127B20.3	0.112352972	0.35841396	-1.673588539
ENSG00000272906	RP11-533E19.7	0.102924	0.328335	-1.673589116
ENSG00000260072	CTD-2313J23.1	0.007818	0.02494	-1.673589975
ENSG00000111249	CUX2	0.013309468	0.042458214	-1.673590784
ENSG00000133321	RARRES3	0.039180816	0.124989947	-1.673592712
ENSG00000233061	TTLL7-IT1	0.017424	0.055584	-1.6735938
ENSG00000236576	RP11-22B10.3	0.046311	0.147736	-1.673594607
ENSG00000255621	RP11-377D9.3	0.02118	0.067566	-1.673594859
ENSG00000171595	DNAI2	0.017851947	0.056949307	-1.67359689
ENSG00000168959	GRM5	0.011758185	0.037509718	-1.673599039
ENSG00000234067	RPL5P10	0.028465	0.090807	-1.673614414
ENSG00000226026	RP11-57H12.3	0.020317426	0.064815706	-1.673625786
ENSG00000232382	OR5K1	0.023197	0.074002	-1.673626024
ENSG00000243316	GUCY2GP	0.015781306	0.050345124	-1.673635443
ENSG00000175573	C11orf68	12.30641261	39.25978122	-1.673641864
ENSG00000204849	SPATA31A1	0.004675344	0.014916527	-1.673767266
ENSG00000250302	LINC01618	0.070009	0.223424	-1.674171863
ENSG00000188100	FAM25A	0.580155	1.851858	-1.674463176
ENSG00000270580	PKD1P6	1.000161835	3.192613624	-1.674504506
ENSG00000226318	RP11-474D14.2	0.400232	1.278181	-1.675183722
ENSG00000212724	KRTAP2-3	26.358418	84.254028	-1.676481876
ENSG00000276490	RP11-400G3.5	0.030814	0.098524	-1.676889229
ENSG00000270379	HEATR9	0.045164445	0.144654967	-1.679356459
ENSG00000181885	CLDN7	20.88956904	66.92374039	-1.679735351
ENSG00000144560	VGLL4	1.869327343	5.993627864	-1.680910286
ENSG00000260350	RP11-152P23.2	0.047935	0.153708	-1.681040917
ENSG00000008294	SPAG9	8.186622845	26.26247941	-1.681662788
ENSG00000241772	AC092620.2	0.333116177	1.069536124	-1.682887889
ENSG00000127528	KLF2	21.95235347	70.48703471	-1.682982298
ENSG00000239473	RPL7P38	0.144631	0.464797	-1.684223946
ENSG00000139540	SLC39A5	0.032258809	0.103682149	-1.684402436
ENSG00000280604	PCBP3-OT1	0.083433	0.26819	-1.684565418
ENSG00000163435	ELF3	4.743184058	15.27281741	-1.687038466
ENSG00000238742	MIR2110	0.186619	0.60134	-1.68808505
ENSG00000277290	RP11-326C3.16	1.821688	5.873132	-1.688854173
ENSG00000158077	NLRP14	0.056115	0.181007	-1.689587119
ENSG00000274213	RP11-670E13.6	0.722492	2.332781	-1.690997356
ENSG00000279488	AC004623.3	1.469967	4.748006	-1.69153799
ENSG00000248319	RP11-205M3.3	0.01166	0.037692	-1.69269056
ENSG00000267592	CTC-507E2.2	0.217157	0.702364	-1.693480442
ENSG00000188396	TCTEX1D4	0.664242	2.148924	-1.693833608
ENSG00000279960	RP5-1181K21.4	0.025233	0.081638	-1.693929098
ENSG00000255152	MSH5-SAPCD1	0.687000252	2.223524718	-1.69446591
ENSG00000277675	AC004878.2	0.197017	0.638273	-1.6958535
ENSG00000117472	TSPAN1	1.698800803	5.507026533	-1.696756864
ENSG00000267132	HMGB3P27	0.014879	0.048235	-1.696802799
ENSG00000263466	RP1-56K13.2	0.095504	0.309614	-1.696837644
ENSG00000143110	C1orf162	0.147098301	0.477095513	-1.697497531

ENSG00000238390	SNORA81	0.655612	2.12813	-1.698672117
ENSG00000258960	CTD-2014B16.2	1.022817	3.320091	-1.69867474
ENSG00000260088	RP11-92G12.3	0.470237	1.528023	-1.700206294
ENSG00000139547	RDH16	0.277401573	0.901655875	-1.700600951
ENSG00000244040	IL12A-AS1	0.00800204	0.026027501	-1.701597064
ENSG00000186073	C15orf41	0.340105606	1.106595269	-1.70207297
ENSG00000266405	CBX3P2	0.035617	0.115891333	-1.70213477
ENSG00000280423	RP11-299J5.1	0.022703	0.073902	-1.702730459
ENSG00000111012	CYP27B1	1.69164001	5.513476724	-1.704539762
ENSG00000147852	VLDLR	0.016348974	0.053314943	-1.70533985
ENSG00000234367	PFN1P3	0.351463	1.146584	-1.705897328
ENSG00000136059	VILL	0.056179137	0.183283398	-1.705969749
ENSG00000112053	SLC26A8	0.01642613	0.053618418	-1.706736057
ENSG00000243056	EIF4EBP3	0.262334	0.856547	-1.70712761
ENSG00000179833	SERTAD2	24.68090382	80.61406966	-1.707636428
ENSG00000226532	RP5-1052M9.1	0.248422	0.811808	-1.708345614
ENSG00000206675	RNU6-32P	0.564547	1.845325	-1.708709328
ENSG00000277925	Telomerase-vert	0.79017	2.584935	-1.709893025
ENSG00000267740	AC024592.12	0.145341059	0.475711386	-1.710644234
ENSG00000268472	RP11-356J5.13	0.044962	0.147253	-1.71151891
ENSG00000250359	PTP4A1P4	0.658595	2.158098	-1.712296915
ENSG00000138079	SLC3A1	0.035898203	0.117632661	-1.712305171
ENSG00000272134	RP11-927P21.8	0.105149	0.344944	-1.713927037
ENSG00000279861	RP11-43N16.4	0.333798	1.096323	-1.715625694
ENSG00000151224	MAT1A	0.433229271	1.423644382	-1.716386188
ENSG00000272369	RP11-446N19.1	0.795253	2.613555	-1.716527706
ENSG00000231170	AC002451.3	0.019518941	0.064178543	-1.717216281
ENSG00000275029	HMGB1P24	0.077205536	0.25386779	-1.717301165
ENSG00000197632	SERPINB2	0.035931567	0.118166618	-1.717498785
ENSG00000130766	SESN2	8.011648	26.350258	-1.717646146
ENSG00000131044	TTLL9	0.025770126	0.084766345	-1.717791999
ENSG00000261395	HSPE1P5	0.660528	2.172781	-1.717851145
ENSG00000117226	GBP3	0.167719197	0.552230687	-1.719223233
ENSG00000233493	TMEM238	0.19869	0.654332	-1.719503563
ENSG00000130822	PNCK	0.059983769	0.197660191	-1.720378258
ENSG00000105388	CEACAM5	0.012252451	0.040376502	-1.720445525
ENSG00000280843	AC093249.1	1.437778	4.747959	-1.723466543
ENSG00000147642	SYBU	1.286825107	4.250205688	-1.723716673
ENSG00000237289	CKMT1B	1.905837635	6.300371867	-1.725011767
ENSG00000276756	CH17-125A10.1	0.037766629	0.12491214	-1.725729789
ENSG00000278273	RP11-763B22.11	0.04732	0.156511	-1.725742078
ENSG00000260592	CTA-363E6.6	0.075581	0.250039	-1.726057626
ENSG00000267764	RP11-484L8.1	0.116069	0.384009	-1.726157419
ENSG00000129437	KLK14	0.206834443	0.684317336	-1.726189044
ENSG00000224592	RP5-884C9.2	0.156260215	0.517484477	-1.727565082
ENSG00000124215	CDH26	0.035418318	0.117303942	-1.7276839
ENSG00000274403	CTD-2036P10.6	0.485497	1.608988	-1.72861928
ENSG00000262848	RP11-517A5.5	0.532	1.764901	-1.730089109

ENSG00000270157	RP5-894A10.6	0.836666	2.776822	-1.730710987
ENSG00000278341	RP5-1142A6.10	0.505692	1.678645	-1.730966301
ENSG00000279254	RP11-536C12.1	0.293273	0.973546	-1.731004891
ENSG00000264734	RP11-260A9.6	0.212067421	0.704318571	-1.731705121
ENSG00000165478	HEPACAM	0.004253916	0.014134252	-1.732332094
ENSG00000118849	RARRES1	0.02185175	0.072612523	-1.732469594
ENSG00000220996	AC034236.1	0.447439	1.486827	-1.732473876
ENSG00000271631	RP11-408O19.5	0.080099	0.266167	-1.732475577
ENSG00000125788	DEFB126	0.074596	0.247881	-1.732477516
ENSG00000265163	CDRT8	0.034875	0.115889	-1.732478512
ENSG00000198547	C20orf203	0.009625	0.031986	-1.732582142
ENSG00000187942	LDLRAD2	0.051893509	0.172462755	-1.732658834
ENSG00000258745	RP11-218E20.5	1.325447	4.406139	-1.733036025
ENSG00000234084	RP3-388E23.2	1.27167	4.231068	-1.734297534
ENSG00000272168	CASC15	0.007022837	0.023372483	-1.734685219
ENSG00000249237	RP11-376N17.4	0.112478	0.374446	-1.735114831
ENSG00000227060	LINC00629	0.016798293	0.055965835	-1.73623178
ENSG00000099860	GADD45B	6.941573402	23.13064663	-1.736470987
ENSG00000223528	RP11-223P11.3	0.019487599	0.064980327	-1.73744662
ENSG00000231459	LINC00032	0.015376	0.051278	-1.737659752
ENSG00000269967	RP11-84A19.4	1.003235	3.348488	-1.738850212
ENSG00000267197	CTC-429L19.3	0.019959	0.066704	-1.740733838
ENSG00000206811	SNORA10	0.705799	2.35884	-1.740748273
ENSG00000244926	ALKBH3-AS1	0.074578275	0.249342201	-1.741299747
ENSG00000125631	HTR5BP	0.060256974	0.201924653	-1.744616926
ENSG00000255817	RP11-196H14.4	0.477352	1.599735	-1.74470753
ENSG00000107831	FGF8	0.196121155	0.657824043	-1.745956572
ENSG00000267924	RP11-255H23.4	0.043395	0.145581	-1.74622135
ENSG00000188959	C9orf152	0.033159017	0.111252559	-1.746365396
ENSG00000223875	NBEAP3	0.653218	2.19222	-1.746756136
ENSG00000267096	CTD-2537I9.13	0.120577	0.404892	-1.747582398
ENSG00000261471	RP11-61F12.1	0.124626	0.418911	-1.749038687
ENSG00000153714	LURAP1L	0.587034795	1.97347223	-1.749218294
ENSG00000278122	AQP7P5	0.049492	0.166384	-1.749249458
ENSG00000268743	CTD-2538G9.5	0.41156	1.384599	-1.750293534
ENSG00000235635	AC016644.1	0.234545	0.789416	-1.750921014
ENSG00000264539	MIR548AR	2.196573	7.394658	-1.751228979
ENSG00000275740	RP11-449H3.3	0.15116	0.509057	-1.751750783
ENSG00000143217	PVRL4	0.116267705	0.391609	-1.751963494
ENSG00000259868	RP11-1012E15.2	0.252274617	0.850825029	-1.753867417
ENSG00000280341	LA16c-60D12.1	0.007659	0.025848	-1.754824712
ENSG00000234741	GAS5	39.73522978	134.1089563	-1.754914997
ENSG00000253764	RP11-439C15.4	0.055707122	0.188036682	-1.75508044
ENSG00000145349	CAMK2D	7.446332272	25.14069614	-1.755422699
ENSG00000259298	RP11-562A8.4	0.91193	3.081505	-1.75664014
ENSG00000232739	RP11-25G10.2	0.187689	0.63474	-1.757821661
ENSG00000248858	FLJ46284	0.063060687	0.213370506	-1.75854798
ENSG00000257173	AK6P2	0.21612	0.731949	-1.759910542

ENSG00000105327	BBC3	1.496597899	5.069090287	-1.760040205
ENSG00000252877	RNA5SP312	0.809513	2.742263	-1.760240787
ENSG00000252149	RNA5SP315	0.809513	2.742263	-1.760240787
ENSG00000277619	CTC-276P9.4	0.03482	0.118167	-1.762839088
ENSG00000269814	CTC-273B12.10	0.10538	0.357624	-1.762842474
ENSG00000261783	RP11-252K23.2	0.12792	0.434122	-1.762858692
ENSG00000205611	LINC01597	0.010698	0.036306	-1.762866882
ENSG00000275729	AC078814.1	0.423296	1.437465	-1.763788068
ENSG00000219361	RPSAP72	0.423661	1.439183	-1.764267817
ENSG00000276663	RP11-407G23.7	0.377406	1.282169	-1.764397169
ENSG00000183977	PP2D1	0.201301919	0.684599929	-1.765900216
ENSG00000244357	RN7SL145P	0.343893	1.170174	-1.766691414
ENSG00000259921	AC022819.3	0.109082	0.371231	-1.766904132
ENSG00000104419	NDRG1	11.79498475	40.24359366	-1.770585586
ENSG00000164742	ADCY1	0.003535586	0.012079765	-1.77257111
ENSG00000274557	AL365274.1	7.691672	26.332373	-1.775468391
ENSG00000206603	SNORA22	0.575348	1.971347	-1.776675003
ENSG00000236966	RP11-216M21.4	0.161746	0.554201	-1.776679279
ENSG00000225680	AL163953.2	0.043465	0.149092	-1.778276798
ENSG00000127831	VIL1	0.013680246	0.04692599	-1.77829306
ENSG00000237763	AMY1A	0.020994806	0.072016973	-1.778304475
ENSG00000146592	CREB5	0.250232513	0.858702428	-1.778889021
ENSG00000213904	LIPE-AS1	0.461172281	1.582743762	-1.779050002
ENSG00000254846	RP11-203M5.2	0.108753	0.373259	-1.779121848
ENSG00000202503	SNORD34	0.016793	0.057649	-1.779435598
ENSG00000164776	PHKG1	0.171457034	0.589488679	-1.781617014
ENSG00000259618	RP11-562A8.5	0.395814	1.362409	-1.783265324
ENSG00000207817	MIR614	19.308659	66.48407	-1.783760732
ENSG00000248968	CTD-2256P15.1	0.022837	0.078663	-1.784312066
ENSG00000238231	RP11-460I13.6	0.138682	0.477795	-1.784611209
ENSG00000177606	JUN	13.82364	47.639835	-1.785030865
ENSG00000271933	RP11-338I21.1	0.173936	0.600005	-1.786417961
ENSG00000223572	CKMT1A	2.13811264	7.378075774	-1.786906747
ENSG00000151303	RP11-96C23.13	0.155368	0.536731	-1.788509823
ENSG00000201217	Y_RNA	0.432393	1.494301	-1.78905571
ENSG00000150672	DLG2	0.017299744	0.059865226	-1.790967573
ENSG00000268112	CTD-3149D2.4	0.276931	0.959475	-1.792718656
ENSG00000202347	RNU1-16P	1.005511	3.484834	-1.793161071
ENSG00000272337	RNU6-90P	0.480153	1.664462	-1.793489837
ENSG00000182376	RP5-1142A6.8	0.020923121	0.07254819	-1.793841576
ENSG00000188162	OTOG	0.008745025	0.030322403	-1.793849697
ENSG00000154645	CHODL	0.015138767	0.052493354	-1.793887052
ENSG00000248751	RP1-130H16.18	0.116935275	0.405658578	-1.794555797
ENSG00000150593	PDCD4	4.78867223	16.63258523	-1.796314831
ENSG00000172940	SLC22A13	0.07033	0.244404	-1.797055775
ENSG00000240006	RP11-200A1.1	0.211587	0.736334	-1.799109329
ENSG00000230551	CTB-89H12.4	3.119777	10.858498	-1.799309742
ENSG00000230650	AC112229.1	0.245944	0.85662	-1.800325501

ENSG00000260618	RP11-23N2.4	0.29419744	1.025639906	-1.801667702
ENSG00000179979	CRIPAK	1.087651	3.792152	-1.801801087
ENSG00000223393	RP5-858B6.3	0.064101	0.223742	-1.80341733
ENSG00000271484	RP11-132J14.3	0.045443	0.158631	-1.803544748
ENSG00000252456	RNA5SP434	0.378567	1.32394	-1.806217181
ENSG00000180354	MTURN	0.692702867	2.422980988	-1.806474534
ENSG00000264270	RP11-474I11.7	0.12144729	0.424807435	-1.806478713
ENSG00000272205	RP11-277B15.3	0.149256	0.52216	-1.806703016
ENSG00000266017	MIR4477B	0.106819	0.374167	-1.808514041
ENSG00000270210	RP11-373D23.3	0.262691	0.921795	-1.811079169
ENSG00000274364	RP3-324O17.7	0.139825	0.490654	-1.811083691
ENSG00000231762	RP11-354K4.1	0.019155	0.067265	-1.812134997
ENSG00000228663	PSMD10P1	0.523162	1.838986	-1.813580837
ENSG00000267041	ZNF850	0.022925811	0.080617484	-1.814119965
ENSG00000277945	RP11-677M24.1	0.967197	3.401862	-1.814442943
ENSG00000184925	LCN12	0.054164156	0.190826252	-1.81684931
ENSG00000214856	KRT16P1	0.021448062	0.075566588	-1.816901169
ENSG00000263276	RP11-96D1.10	0.261861	0.922729	-1.81710579
ENSG00000230751	AC007036.4	0.232935	0.821051	-1.817544408
ENSG00000228314	CYP4F29P	0.024702503	0.087101613	-1.818042197
ENSG00000255780	RP11-1029F8.1	0.200378	0.707402	-1.819806178
ENSG00000234271	RP1-138B7.4	0.163128	0.575898	-1.819808878
ENSG00000168209	DDIT4	23.69862249	83.67303567	-1.819959574
ENSG00000181029	TRAPPC5	1.056261359	3.731900628	-1.820943715
ENSG00000237672	KRR1P1	0.882994	3.121752	-1.821880391
ENSG00000206069	TMEM211	0.107149724	0.379026322	-1.822669905
ENSG00000265496	MIR1539	0.87061	3.083246	-1.824351505
ENSG00000153233	PTPRR	0.079185197	0.280452551	-1.824454041
ENSG00000259135	RP11-671J11.4	0.00671514	0.023800393	-1.825496056
ENSG00000259648	RP11-643G16.4	0.026976947	0.095619471	-1.82557734
ENSG00000225360	RP11-149F8.4	0.107831	0.382207	-1.825582207
ENSG00000254034	RP11-662B19.2	0.278629	0.987601	-1.825582874
ENSG00000267193	RP11-116O18.3	0.062662943	0.222108938	-1.82558303
ENSG00000281319	AL136115.1	0.077803	0.275773	-1.825583524
ENSG00000271937	RP11-424N24.2	0.024949	0.088432	-1.825586527
ENSG00000177553	RP11-56N19.5	0.008174	0.028973	-1.825594927
ENSG00000163154	TNFAIP8L2	0.012961	0.045943	-1.825668032
ENSG00000171094	ALK	0.002202705	0.0078082	-1.825713655
ENSG00000251330	CTD-2283N19.1	0.09902	0.351142	-1.826262712
ENSG00000277873	RP11-33N14.3	0.348647	1.236382	-1.826285583
ENSG00000271366	AC002128.5	0.448384	1.590235	-1.826433275
ENSG00000124780	KCNK17	0.000401366	0.001424023	-1.826983729
ENSG00000158428	CATIP	0.026640206	0.094631481	-1.82871495
ENSG00000228060	RP11-69E11.8	0.979744	3.480731	-1.828913585
ENSG00000070031	SCT	0.130717	0.464716	-1.829902539
ENSG00000163359	COL6A3	0.005184528	0.01843366	-1.830057873
ENSG00000230946	HNRNPA1P68	0.103976	0.370386	-1.832779008
ENSG00000249328	RP11-26J3.1	0.029853197	0.106432629	-1.833983172

ENSG00000245330	KB-1471A8.1	0.026168122	0.093422013	-1.835952119
ENSG00000260281	ITFG1-AS1	0.113015521	0.403531712	-1.836161139
ENSG00000231861	OR5K2	0.120039	0.428626	-1.836216159
ENSG00000229447	RP11-490K7.4	1.56532	5.592868	-1.837130662
ENSG00000067082	KLF6	3.361101387	12.01534031	-1.837871543
ENSG00000236451	AC067956.1	0.403019	1.441951	-1.83910238
ENSG00000238832	snoU109	0.554594	1.985664	-1.840117607
ENSG00000260095	RP11-715J22.3	0.946131	3.389008	-1.840751186
ENSG00000263624	RP11-45M22.3	0.241273	0.864346	-1.840942465
ENSG00000253595	LINC01300	0.231884808	0.831150458	-1.841701359
ENSG00000244457	ENO1P1	5.588688	20.062702	-1.843934377
ENSG00000279086	RP11-667F14.1	0.051948	0.186615	-1.844924842
ENSG00000114796	KLHL24	1.505004276	5.408560742	-1.845477148
ENSG00000277568	uc_338	0.326258	1.172611	-1.845639313
ENSG00000263293	THCAT158	0.20843802	0.749379547	-1.846078149
ENSG00000215277	RNF212B	0.022444661	0.080832339	-1.848560288
ENSG00000258942	RP11-255G12.2	0.602174	2.170929	-1.85006022
ENSG00000145780	FEM1C	6.065976	21.881981	-1.850931658
ENSG00000224431	AC063976.7	0.171753	0.61965	-1.851118262
ENSG00000223505	RP11-397P13.7	0.117931	0.425705	-1.851911032
ENSG00000172243	CLEC7A	0.035470179	0.128131999	-1.852952294
ENSG00000202078	Y_RNA	0.270246	0.977043	-1.854148793
ENSG00000279442	LA16c-60D12.2	0.016474	0.05956	-1.85415286
ENSG00000280381	RP11-197N18.8	0.018288	0.066119	-1.854167596
ENSG00000253159	PCDHGA12	0.005284958	0.019107578	-1.854180906
ENSG00000088448	ANKRD10	13.37881635	48.37202071	-1.854222323
ENSG00000255443	RP1-68D18.4	2.376982	8.606223	-1.856249245
ENSG00000274386	RP5-994D16.11	0.248878283	0.90126001	-1.856503032
ENSG00000272659	AP000295.10	0.397742	1.441126	-1.85729166
ENSG00000103044	HAS3	0.360683096	1.308288184	-1.858876649
ENSG00000267033	CTD-2562J15.4	0.301776	1.094887	-1.859232002
ENSG00000102524	TNFSF13B	0.112641113	0.409262683	-1.861293636
ENSG00000269680	CTD-3128G10.6	0.496663	1.806449	-1.862817346
ENSG00000212304	SNORD12	0.713724	2.59688	-1.863341159
ENSG00000101695	RNF125	0.748767631	2.726129794	-1.864264279
ENSG00000277294	AL645608.1	0.912051	3.320817	-1.864351818
ENSG00000173809	TDRD12	0.010889656	0.039677097	-1.865348075
ENSG00000241599	RP11-34P13.9	0.389796	1.421108	-1.866225008
ENSG00000102962	CCL22	0.034753	0.126756	-1.866844612
ENSG00000267697	LUZP6	0.94772	3.456702	-1.866863446
ENSG00000274494	MIR6832	0.212067	0.773496	-1.866873692
ENSG00000187688	TRPV2	0.149256317	0.54487814	-1.868141623
ENSG00000139146	FAM60A	8.43674153	30.82326514	-1.869261887
ENSG00000256056	RP11-709A23.2	0.10969	0.400838	-1.869587278
ENSG00000254208	RP11-219B4.3	0.205827	0.752504	-1.870267006
ENSG00000272703	RP11-78A19.4	0.127241	0.465437	-1.871022287
ENSG00000178750	STX19	0.131856	0.482623	-1.871933449
ENSG00000250292	RP11-451F20.1	0.780084	2.855942	-1.872265293

ENSG00000227782	CTC-529I10.2	2.207837	8.087454	-1.873051936
ENSG00000213906	LTB4R2	0.493014944	1.806045521	-1.873130973
ENSG00000282917	RP11-121C2.3	0.173546531	0.636248422	-1.874267646
ENSG00000204086	RPA4	0.018923	0.069386	-1.874503772
ENSG00000263220	RP11-420A6.2	0.099406	0.364571	-1.874794967
ENSG00000257681	RP11-341G23.4	0.313959004	1.151652645	-1.875057553
ENSG00000227394	AC007386.3	0.03498	0.128327	-1.875222551
ENSG00000215183	MSMP	0.020356252	0.074902935	-1.879550327
ENSG00000265136	RP13-638C3.6	0.994778	3.66086	-1.879736095
ENSG00000185345	PARK2	0.077918306	0.287007895	-1.881056212
ENSG00000239256	RPL35AP35	0.037314	0.13755	-1.882167212
ENSG00000278773	ZNRD1-AS1_3	0.014093	0.051956	-1.882311611
ENSG00000267222	RP11-194N12.2	0.14882	0.549118	-1.883547779
ENSG00000277558	RP3-468O1.6	0.429518	1.585555	-1.884197425
ENSG00000274272	RP11-44M6.7	0.649085	2.396606	-1.884513429
ENSG00000261087	KB-1460A1.5	0.885685	3.271754	-1.885198687
ENSG00000255158	RP11-754B17.1	0.055808	0.206393	-1.886850191
ENSG00000130643	CALY	0.050727048	0.187629713	-1.8870612
ENSG00000274799	Metazoa_SRP	0.180483	0.66787	-1.887704358
ENSG00000272274	LINC00551	0.017698	0.065542	-1.888833362
ENSG00000272033	RP11-52A20.2	0.16132	0.597528	-1.889081008
ENSG00000125775	SDCBP2	0.210340716	0.779168037	-1.889206353
ENSG00000138623	SEMA7A	5.059761739	18.75193467	-1.889898092
ENSG00000270800	RPS10-NUDT3	8.818425	32.692299	-1.890357921
ENSG00000258521	RP11-638I2.9	0.121708	0.451287	-1.890621219
ENSG00000234065	MTND4P26	0.147558	0.547179	-1.890730723
ENSG00000229010	RP5-979D14.1	0.053509	0.198486	-1.891183779
ENSG00000246451	RP11-894P9.1	4.003703	14.882296	-1.894190256
ENSG00000174502	SLC26A9	0.018087489	0.067313905	-1.895912417
ENSG00000213122	RPL23AP46	0.034624	0.128859	-1.895948995
ENSG00000230328	RP11-35N6.6	0.032213	0.119887	-1.895960298
ENSG00000260010	ZNF720P1	0.069997	0.260508	-1.895962681
ENSG00000235610	AC013448.2	0.129343	0.481377	-1.895965234
ENSG00000164400	CSF2	0.129343	0.481377	-1.895965234
ENSG00000261774	RP11-328J14.2	0.16909	0.629308	-1.895974942
ENSG00000272950	RP11-307C18.1	0.176111	0.655439	-1.895976495
ENSG00000120669	SOHLH2	0.029947773	0.111457822	-1.895977226
ENSG00000181350	LRRC75A	0.589795476	2.196594074	-1.896981627
ENSG00000224452	RSL24D1P6	0.418846	1.560182	-1.897222533
ENSG00000166922	SCG5	0.11015388	0.410391363	-1.89748006
ENSG00000176700	SCAND2P	0.418231989	1.558608671	-1.89788343
ENSG00000277703	MIR7113	0.227806	0.849115	-1.898154213
ENSG00000257403	CTD-2547E10.3	0.099796	0.372123	-1.898725667
ENSG00000234945	GTF3C2-AS1	0.194558775	0.726390821	-1.900539924
ENSG00000224435	NF1P6	0.007839	0.029284	-1.901371099
ENSG00000255125	RP11-685M7.5	0.190603	0.712254	-1.901820992
ENSG00000260300	RP11-505K9.4	0.049979711	0.186832836	-1.902333557
ENSG00000173611	SCAI	0.496668449	1.857105505	-1.902700771

ENSG00000108255	CRYBA1	0.298735209	1.117117145	-1.90284129
ENSG00000274203	MIR6513	0.000104	0.000389	-1.903186627
ENSG00000204389	HSPA1A	2.71323	10.155175	-1.904131846
ENSG00000174885	NLRP6	0.022535883	0.084378412	-1.904649977
ENSG00000230454	U73166.2	0.131449067	0.492866	-1.906691563
ENSG00000274458	SNORA40	0.410351	1.539053	-1.90711254
ENSG00000228544	CCDC183-AS1	0.211921	0.795421	-1.908192095
ENSG00000271503	CCL5	0.100984722	0.379415931	-1.909643208
ENSG00000268434	AC011530.4	0.037103427	0.13940687	-1.909677296
ENSG00000196604	POTEF	0.278270877	1.045715235	-1.909928204
ENSG00000230084	RP4-613B23.1	0.170275192	0.639902622	-1.909984115
ENSG00000251682	RP11-631M6.3	0.170516	0.641022	-1.910466753
ENSG00000243480	AMY2A	0.045586626	0.171466851	-1.911247159
ENSG00000280970	AL034418.1	0.365835	1.376117	-1.911338124
ENSG00000253716	MINCR	0.944481559	3.556232897	-1.912755275
ENSG00000087086	FTL	473.7832401	1787.317288	-1.915496698
ENSG00000225761	RP11-417O11.5	0.379994	1.437919	-1.919933865
ENSG00000250156	CTC-498M16.2	0.085759454	0.324533916	-1.920001636
ENSG00000226647	RP11-336A10.2	0.136388366	0.516158547	-1.920093693
ENSG00000268279	RP11-434D12.1	0.120077435	0.45512442	-1.922295931
ENSG00000280483	AC022098.1	0.065654	0.249054	-1.923503766
ENSG00000186469	GNG2	0.008893977	0.033767355	-1.924728595
ENSG00000262833	RP11-28G8.1	0.013344	0.050674	-1.92505452
ENSG00000200731	RNU1-124P	0.098283	0.373246	-1.925112999
ENSG00000233677	DDX39BP1	0.074224	0.281878	-1.925113226
ENSG00000257512	RP11-486A14.1	0.012578067	0.047767425	-1.925116886
ENSG00000238835	SCARNA18	0.744452	2.827192	-1.925119126
ENSG00000201770	RNU6-384P	0.137028	0.52039	-1.925122519
ENSG00000262708	RP11-411G7.2	0.023991	0.091112	-1.925147784
ENSG00000167371	PRRT2	0.243618574	0.925987954	-1.926369296
ENSG00000225885	AC023590.1	0.059210619	0.225213474	-1.927365299
ENSG00000106034	CPED1	0.161969447	0.616189198	-1.927651696
ENSG00000174945	AMZ1	0.035862174	0.136486241	-1.92822068
ENSG00000242398	RN7SL800P	0.181772	0.691971	-1.928581592
ENSG00000254248	RP11-320N21.2	0.03205	0.122063	-1.929229692
ENSG00000226608	FTLP3	87.938622	335.177795	-1.930357745
ENSG00000224259	LINC01133	0.032263711	0.123336455	-1.934614997
ENSG00000237436	RP11-312B8.1	0.103019	0.393841	-1.934702867
ENSG00000139946	PELI2	0.789208877	3.019675448	-1.935914409
ENSG00000160868	CYP3A4	0.020254877	0.077536529	-1.936606846
ENSG00000248544	CTB-47B11.3	0.236286653	0.904865863	-1.937165805
ENSG00000138613	APH1B	0.243036264	0.931205926	-1.937928641
ENSG00000279198	CTD-2231E14.2	0.315806	1.211089	-1.939194403
ENSG00000196131	VN1R2	0.067628743	0.25948192	-1.939925571
ENSG00000234017	RP11-214N15.5	0.710857	2.729531	-1.941021809
ENSG00000238711	RNY4P25	0.190861	0.732882	-1.941058595
ENSG00000196967	ZNF585A	0.005484946	0.021063587	-1.941201863
ENSG00000196526	AFAP1	1.272235363	4.889636435	-1.942361606

ENSG00000236478	AC012513.4	0.085946	0.33034	-1.94244927
ENSG00000145244	CORIN	0.251285739	0.96646685	-1.943391448
ENSG00000229759	MRPS18AP1	0.56272	2.165952	-1.944512126
ENSG00000207034	Y_RNA	0.522013	2.0168	-1.949910383
ENSG00000246339	EXTL3-AS1	0.035833278	0.138444117	-1.949931827
ENSG00000206172	HBA1	0.150554649	0.582038248	-1.950826702
ENSG00000156413	FUT6	0.019270864	0.074582741	-1.952420535
ENSG00000218596	RP11-2J18.1	0.067323	0.260581	-1.952560524
ENSG00000123358	NR4A1	3.03281089	11.74792445	-1.953678446
ENSG00000113073	SLC4A9	0.089751035	0.347673286	-1.95373174
ENSG00000279339	CTD-2373H9.3	0.175116	0.678896	-1.954879679
ENSG00000129757	CDKN1C	2.716358131	10.54153262	-1.956339028
ENSG00000258376	RP4-647C14.2	0.058238	0.226275	-1.958044481
ENSG00000200914	RNA5SP435	0.273332	1.062366	-1.958554604
ENSG00000163827	LRRC2	0.007423212	0.028880043	-1.959957324
ENSG00000274751	LA16c-358B7.4	0.013162	0.051224	-1.960441188
ENSG00000278917	RP11-15A1.4	0.012179	0.047399	-1.960460942
ENSG00000273356	RP11-804H8.6	0.68191	2.654533	-1.96080483
ENSG00000213706	AL590762.7	0.133703	0.521301	-1.963084791
ENSG00000215146	RP11-313J2.1	0.007850019	0.03063637	-1.964477387
ENSG00000251201	TMED7-TICAM2	2.579349572	10.07058094	-1.965067694
ENSG00000241472	PTPRG-AS1	0.014782591	0.057771131	-1.9664496
ENSG00000214279	SCART1	0.15162743	0.592694584	-1.966758105
ENSG00000267682	CTD-3220F14.2	0.135723	0.531103	-1.968326454
ENSG00000153292	ADGRF1	0.71024501	2.782320499	-1.96989992
ENSG00000226314	ZNF192P1	0.281760407	1.103860622	-1.97001722
ENSG00000157514	TSC22D3	2.430592599	9.527314696	-1.970761545
ENSG00000280620	SCAANT1	1.021916	4.007042	-1.971261018
ENSG00000023445	BIRC3	1.122724358	4.409220938	-1.973519997
ENSG00000212440	SNORA75	1.020186	4.008527	-1.974239984
ENSG00000226186	TCEB1P21	0.103004	0.405904	-1.978438194
ENSG00000261592	RP11-178L8.3	0.250665	0.988517	-1.979505208
ENSG00000145782	ATG12	15.59296858	61.51786479	-1.980109817
ENSG00000275996	SNORD27	0.294953	1.164417	-1.981050819
ENSG00000261757	AC005592.3	0.124137	0.490385	-1.981981664
ENSG00000261609	GAN	2.040605219	8.069610298	-1.983501902
ENSG00000229873	OGFR-AS1	0.16696	0.66063	-1.98433998
ENSG00000128886	ELL3	0.651996581	2.583343757	-1.986303328
ENSG00000267127	RP11-795F19.5	0.052174458	0.206939504	-1.98779347
ENSG00000180667	YOD1	7.265803392	28.83062978	-1.988408119
ENSG00000201818	RNY4P17	0.477152	1.894219	-1.989082313
ENSG00000265080	MIR4800	0.123606	0.491595	-1.991721467
ENSG00000219222	RPL12P47	0.050095	0.199274	-1.992014969
ENSG00000266445	RP13-991F5.2	0.058306	0.232168	-1.99345288
ENSG00000231324	AP000696.2	0.155429	0.619066	-1.993837519
ENSG00000269807	AC007292.4	0.047522	0.189498	-1.995515163
ENSG00000259605	AC074212.5	0.276256631	1.102642583	-1.996884225
ENSG00000253806	CTD-2292P10.2	0.140488	0.560905	-1.997309538

ENSG00000262074	SNORD3B-2	0.026381	0.105367628	-1.997860544
ENSG00000230612	AC004237.1	0.130363086	0.52106671	-1.998932682
ENSG00000230149	RP3-508I15.19	0.177153	0.708282	-1.999327982
ENSG00000269813	CTD-3193O13.14	0.059591	0.238297	-1.999594426
ENSG00000275405	U1	0.147723	0.592026	-2.002766069
ENSG00000258725	PRC1-AS1	0.177476848	0.71143211	-2.003095252
ENSG00000236255	AC009404.2	1.372931966	5.503605097	-2.003116819
ENSG00000105339	DENND3	1.548813963	6.211269468	-2.003724295
ENSG00000282024	RP3-467D16.3	0.078593323	0.315436222	-2.004869673
ENSG00000244371	PFN1P8	0.514399	2.066481	-2.006216356
ENSG00000240974	CTD-2024I7.1	0.244694	0.983537	-2.0070006
ENSG00000233967	RP11-250B2.3	0.54434477	2.190261048	-2.008510227
ENSG00000265094	RP11-178F10.1	0.148522	0.597645	-2.008612133
ENSG00000258334	RP11-161H23.9	0.04537	0.182612	-2.008971008
ENSG00000259024	TVP23C-CDRT4	0.149829047	0.604168876	-2.011634519
ENSG00000124678	TCP11	0.043722276	0.176457586	-2.012881051
ENSG00000203706	SERTAD4-AS1	0.099359319	0.401052385	-2.013063507
ENSG00000271730	RP11-787I22.3	0.395157	1.597807	-2.015595285
ENSG00000270903	HNRNPA3P9	0.606664	2.453759	-2.016023951
ENSG00000231731	AC010976.2	0.091660172	0.370759286	-2.016115932
ENSG00000270739	RP11-608O8.2	0.046609	0.188532	-2.016128951
ENSG00000042980	ADAM28	0.018830974	0.076179784	-2.016300563
ENSG00000124224	PPP4R1L	0.57770877	2.340202618	-2.018219145
ENSG00000266347	AC068641.1	0.675045	2.734512	-2.018227809
ENSG00000213793	ZNF888	0.15703	0.63634	-2.018757605
ENSG00000214822	KRT16P3	0.02164818	0.087783668	-2.019706794
ENSG00000249463	RP11-1E22.1	0.115385	0.467901	-2.019747626
ENSG00000272980	RP11-517H2.6	0.216822	0.879389	-2.019990338
ENSG00000174500	GCSAM	1.080691549	4.384764843	-2.020544665
ENSG00000280347	AC000123.2	0.887998	3.60409	-2.021006706
ENSG00000177112	MRVI1-AS1	0.071827591	0.291626348	-2.021511037
ENSG00000257335	MGAM	0.024485926	0.099423841	-2.021639068
ENSG00000172260	NEGR1	0.003793553	0.015409102	-2.022161096
ENSG00000223390	RP11-91A18.4	0.066793	0.271696	-2.024224507
ENSG00000257542	OR7E47P	0.018222381	0.074138212	-2.024505832
ENSG00000268199	CTD-3137H5.1	0.106211	0.432315	-2.025149704
ENSG00000007545	CRAMP1	0.881905121	3.590275994	-2.025399394
ENSG00000228909	AC008281.1	0.083028	0.338212	-2.026257997
ENSG00000224117	PTPN2P2	0.10769	0.438848	-2.026837044
ENSG00000272968	RBAK-RBAKDN	0.257870814	1.051258979	-2.02739772
ENSG00000204131	NHSL2	0.041552575	0.169408663	-2.027497871
ENSG00000259198	RP11-133K1.6	0.012661	0.051697	-2.029689206
ENSG00000226761	TAS2R46	0.054727	0.223466	-2.029730666
ENSG00000265381	AL121987.1	0.061841	0.252562	-2.030002037
ENSG00000138587	MNS1	0.243428478	0.994273718	-2.03014512
ENSG00000180878	C11orf42	0.099842	0.408299	-2.031907295
ENSG00000232163	RPLP1P13	0.088516	0.36201	-2.032019387
ENSG00000227431	CSE1L-AS1	0.0238	0.097339	-2.03205638

ENSG00000234788	HSPA8P3	0.052201	0.213677	-2.033282276
ENSG00000273353	CTA-268H5.12	0.792776	3.247232	-2.034225269
ENSG00000270751	DEAR	0.239899	0.982652	-2.034253442
ENSG00000128482	RNF112	0.068567834	0.28097675	-2.034846899
ENSG00000119986	AVPI1	7.659932	31.392511	-2.035016941
ENSG00000182574	RP3-341D10.1	0.062768	0.257445	-2.036163105
ENSG00000226055	PAICSP1	0.083642	0.343397	-2.037577971
ENSG00000262962	KARSP3	0.005345	0.02199	-2.040585751
ENSG00000237457	LINC01351	0.106487703	0.438669957	-2.042449058
ENSG00000270857	RP11-757G14.3	0.174701	0.721194	-2.045499528
ENSG00000229990	RP11-574K11.8	0.117543	0.485331	-2.045780388
ENSG00000221338	AC108456.1	0.197017	0.814718	-2.047980662
ENSG00000177688	SUMO4	0.25273	1.046026	-2.049249879
ENSG00000146674	IGFBP3	0.324794439	1.34600397	-2.05108383
ENSG00000241570	PAQR9-AS1	0.011512443	0.047742614	-2.052083513
ENSG00000279463	RP11-157H4.1	0.113607	0.471139	-2.05210103
ENSG00000243708	PLA2G4B	0.25619717	1.064280379	-2.054551825
ENSG00000265827	MIR4680	0.793984	3.300982	-2.055713431
ENSG00000157224	CLDN12	2.131314106	8.864489341	-2.056294295
ENSG00000115112	TFCP2L1	0.404149907	1.683049974	-2.058115594
ENSG00000130066	SAT1	210.4252564	876.5127389	-2.058467183
ENSG00000267022	AC084219.2	0.060878	0.253634	-2.058755285
ENSG00000274775	RP1-274L7.4	0.002838	0.011829	-2.059381622
ENSG00000135218	CD36	0.007548044	0.031461044	-2.059391772
ENSG00000265992	ESRG	0.016209	0.067564	-2.059459655
ENSG00000229692	SOS1-IT1	0.26748436	1.116581664	-2.061562329
ENSG00000253550	RP11-115C21.4	0.201751	0.8435	-2.063812244
ENSG00000266038	MIR4659A	0.201072	0.841875	-2.065893845
ENSG00000259604	RP11-66B24.1	0.003800352	0.015915944	-2.066267924
ENSG00000273284	RP11-888D10.4	1.105204	4.63397	-2.067936016
ENSG00000235339	GEMIN2P2	0.21355	0.895701	-2.068443319
ENSG00000186265	BTLA	0.041300564	0.173284729	-2.068911125
ENSG00000254388	DUTP2	0.329781	1.38399	-2.069253332
ENSG00000127074	RGS13	0.008593317	0.036118205	-2.071439168
ENSG00000257940	RP11-248E9.6	0.045935	0.193427	-2.074123457
ENSG00000255933	RP11-495K9.5	0.349366	1.473834	-2.076762922
ENSG00000265311	AL020993.1	0.038468	0.162362	-2.077483287
ENSG00000265429	AC017074.3	0.311033	1.312924	-2.077643846
ENSG00000261574	RP1-168P16.2	0.121182	0.511767	-2.078311701
ENSG00000261240	RP11-304L19.4	0.212102	0.895784	-2.078392673
ENSG00000163728	TTC14	2.329521988	9.84280888	-2.079036134
ENSG00000004799	PDK4	1.088967397	4.601661207	-2.079194009
ENSG00000220924	OSTCP4	0.032495	0.137411	-2.080207846
ENSG00000207513	RNU1-3	0.147723	0.625026	-2.081021738
ENSG00000253607	RP11-557C18.3	0.142523	0.603074	-2.081140282
ENSG00000272829	XXbac-B135H6.18	0.128851	0.545315	-2.081386106
ENSG00000228547	OR7E26P	0.005059	0.021411	-2.081428034
ENSG00000236045	RP3-467K16.7	0.070009	0.296421	-2.082035355

ENSG00000259254	CTD-2339L15.3	0.088772	0.376243	-2.083488135
ENSG00000213872	AC092798.2	0.287873	1.22091	-2.084452467
ENSG00000264771	AC022819.1	0.066709	0.283109	-2.085404293
ENSG00000128016	ZFP36	30.32863563	128.860988	-2.087063059
ENSG00000238133	MLK7-AS1	0.078623061	0.334059865	-2.087082217
ENSG00000182050	MGAT4C	0.00183374	0.007797241	-2.088174405
ENSG00000196593	ANKRD20A19P	0.018204	0.077424842	-2.088541034
ENSG00000279717	LLNLF-158E9.1	0.018999	0.080807	-2.088556788
ENSG00000183709	IFNL2	0.020718	0.08812	-2.088584755
ENSG00000267075	RP11-434D2.3	0.011108	0.047246	-2.088593109
ENSG00000269916	RP11-193M21.1	0.055686	0.236851	-2.088593192
ENSG00000138722	MMRN1	0.018474429	0.078577867	-2.088593228
ENSG00000164076	CAMKV	0.013050362	0.055507801	-2.088600696
ENSG00000251111	FCF1P8	0.042627	0.181308	-2.088603154
ENSG00000279894	GHc-857G6.7	0.022158	0.094246	-2.08860372
ENSG00000237370	AC010907.2	0.036722	0.156192	-2.088604022
ENSG00000116981	NT5C1A	0.045977	0.195557	-2.08860494
ENSG00000173641	HSPB7	0.013016959	0.055365898	-2.08860523
ENSG00000276107	CTD-2033D15.2	0.069436	0.295337	-2.088606364
ENSG00000231859	AC007875.2	0.129179	0.549447	-2.088608767
ENSG00000256574	OR13A1	0.023411432	0.099577684	-2.088609249
ENSG00000204949	FAM83A-AS1	0.037785	0.160714	-2.088610082
ENSG00000226310	RP3-323P24.3	0.076536	0.325537	-2.088611119
ENSG00000090382	LYZ	0.057128673	0.242990714	-2.088614247
ENSG00000276769	MIR6752	0.14337	0.609809	-2.088614269
ENSG00000267765	CTD-3193K9.3	0.141575	0.602175	-2.088616284
ENSG00000143556	S100A7	0.094955494	0.403883685	-2.088616482
ENSG00000201998	SNORA23	0.559299	2.378924	-2.088617526
ENSG00000244184	RP11-314A20.2	0.107831	0.458649	-2.088618501
ENSG00000205090	TMEM240	0.036881333	0.156871303	-2.08861874
ENSG00000273632	AC027319.3	0.229262	0.975145	-2.088619508
ENSG00000229065	RP11-80I15.4	0.096212	0.409229	-2.088619635
ENSG00000229637	PRAC2	0.0774673	0.329500373	-2.088620738
ENSG00000225665	SAR1P3	0.085973	0.365679	-2.088622224
ENSG00000279380	AC069063.2	0.038616	0.16425	-2.088622734
ENSG00000271714	CTD-2377O17.1	0.025941	0.110338	-2.088623735
ENSG00000226247	SUPT4H1P1	0.074847	0.318356	-2.088624558
ENSG00000279544	AL133243.4	0.065482	0.278523	-2.088626177
ENSG00000271996	RP11-337N6.1	0.090241	0.383834	-2.088627551
ENSG00000273301	RP11-314B1.2	0.0198	0.084218	-2.088628185
ENSG00000235387	LINC00961	0.031573	0.134294	-2.088631595
ENSG00000259465	AHCYP7	0.038911	0.165506	-2.088633557
ENSG00000275512	RP11-322E11.2	0.020093	0.085465	-2.088640739
ENSG00000279162	CTD-3126B10.2	0.049654	0.211202	-2.088641649
ENSG00000133636	NTS	0.032729986	0.139216196	-2.088642177
ENSG00000274421	RP11-386J22.3	0.040847	0.173742	-2.088644521
ENSG00000267408	CTB-50L17.2	0.046078	0.195992	-2.088644764
ENSG00000280205	RP11-504I13.2	0.013518	0.057499	-2.088655146

ENSG00000183034	OTOP2	0.018320603	0.077927289	-2.08866165
ENSG00000167332	OR51E2	0.017721128	0.075377494	-2.088663363
ENSG00000138109	CYP2C9	0.007543742	0.032087671	-2.088666866
ENSG00000060982	BCAT1	0.004322286	0.018385119	-2.088672148
ENSG00000179603	GRM8	0.007882153	0.033527269	-2.088673233
ENSG00000056487	PHF21B	0.004423322	0.018815071	-2.088686564
ENSG00000255444	CYCSP27	0.012159	0.05172	-2.088697694
ENSG00000166159	LRTM2	0.071162728	0.302933204	-2.089805995
ENSG00000129538	RNASE1	1.226105624	5.226316084	-2.091711114
ENSG00000171777	RASGRP4	0.097826645	0.41744037	-2.093270757
ENSG00000266714	MYO15B	0.399093271	1.703118635	-2.093381075
ENSG00000241322	CDRT1	0.071180879	0.303824709	-2.09367755
ENSG00000276058	STMN1P1	0.578366	2.472324	-2.095813173
ENSG00000272389	CTC-487M23.7	0.145834	0.623394	-2.095817158
ENSG00000268949	MRPS17P1	0.00542	0.02317	-2.095893288
ENSG00000167751	KLK2	0.004413433	0.018874461	-2.096462382
ENSG00000228446	AC073052.1	0.25662	1.097571	-2.09660875
ENSG00000271871	AC005740.6	0.04361	0.186623	-2.097395904
ENSG00000233647	NENFP1	0.080822	0.346093	-2.098339805
ENSG00000162772	ATF3	4.814103249	20.61595533	-2.098422328
ENSG00000181126	HLA-V	0.204890541	0.878048714	-2.099447597
ENSG00000111863	ADTRP	0.055188525	0.236565771	-2.099801116
ENSG00000279530	AC092881.1	0.293858	1.261979	-2.102496823
ENSG00000281613	RP11-506B6.7	0.008757859	0.037823693	-2.110640094
ENSG00000266264	AL162311.1	0.036599	0.158104	-2.110997733
ENSG00000008118	CAMK1G	0.019018991	0.082161846	-2.111027885
ENSG00000249947	XBP1P1	0.156123	0.675677	-2.113650656
ENSG00000092847	AGO1	2.664558199	11.5357107	-2.114138639
ENSG00000275371	RP11-455F5.6	0.082203	0.356069	-2.114893886
ENSG00000212452	SNORD69	3.987677	17.290327	-2.116344692
ENSG00000253882	RP11-61L23.2	0.122389577	0.530754887	-2.116565049
ENSG00000106460	TMEM106B	2.06173195	8.943704852	-2.117015802
ENSG00000253347	CTD-2026D20.2	0.097229	0.421993	-2.117760479
ENSG00000163116	STPG2	0.008459282	0.036722742	-2.118066699
ENSG00000238840	U8	0.267875	1.163795	-2.119205103
ENSG00000251994	RNU2-27P	2.007174	8.720267	-2.119206621
ENSG00000106366	SERPINE1	13.233049	57.580688	-2.121439519
ENSG00000145029	NICN1	0.599434119	2.608692277	-2.121653664
ENSG00000249438	KRT18P45	0.007827	0.034105	-2.123451913
ENSG00000051108	HERPUD1	7.597226378	33.12881425	-2.124541851
ENSG00000261668	RP11-50D9.3	0.092442	0.403616	-2.126362988
ENSG00000271576	RP11-486G15.2	0.469224	2.049212	-2.126720533
ENSG00000267523	CTD-2537I9.12	0.451776112	1.974360578	-2.127705598
ENSG00000117983	MUC5B	0.013244972	0.057909438	-2.128353732
ENSG00000260645	RP11-250B2.5	0.571559	2.502342	-2.130304643
ENSG00000142871	CYR61	8.902815425	39.01223558	-2.131593123
ENSG00000244161	FLNB-AS1	0.884794	3.879642	-2.132510024
ENSG00000225420	AC104134.2	0.301284	1.322834	-2.134436068

ENSG00000177400	OR7E8P	0.00509	0.02237	-2.135827695
ENSG00000204264	PSMB8	0.060442097	0.265665127	-2.13598325
ENSG00000212238	RNA5SP244	0.672557	2.957301	-2.136552643
ENSG00000232493	RPL12P11	0.085335	0.375515	-2.137661051
ENSG00000260536	RP5-1085F17.4	0.14415	0.634412	-2.137849223
ENSG00000276757	RN7SL192P	0.170221	0.749362	-2.138253789
ENSG00000124466	LYPD3	0.601414818	2.648842901	-2.138929963
ENSG00000257221	RP11-689B22.2	0.042842605	0.189012573	-2.141364082
ENSG00000205209	SCGB2B2	0.016000164	0.07059157	-2.141409231
ENSG00000170629	DPY19L2P2	0.02623221	0.11574339	-2.141518545
ENSG00000173269	MMRN2	0.788206201	3.480251305	-2.142546481
ENSG00000222345	SNORD19	2.366681	10.461077	-2.144094223
ENSG00000279344	RP11-44F14.7	0.057893	0.25625	-2.146091181
ENSG00000167772	ANGPTL4	2.898581697	12.83098343	-2.146212696
ENSG00000268629	TEX13A	0.008871	0.039303	-2.147470788
ENSG00000234884	CTA-407F11.8	0.013008876	0.057636263	-2.147480432
ENSG00000227367	SLC9B1P4	0.019758	0.08754	-2.147505466
ENSG00000112619	PRPH2	0.016418	0.072742	-2.1475102
ENSG00000202343	SNORA2	0.090482	0.400892	-2.147510904
ENSG00000260882	RP11-10K17.3	0.111553	0.494251	-2.147514572
ENSG00000278816	RP5-1116H23.4	0.080362	0.356056	-2.14751879
ENSG00000230498	RP4-564M11.2	0.09831	0.435578	-2.14752101
ENSG00000115221	ITGB6	0.007685768	0.034053803	-2.147554648
ENSG00000142182	DNMT3L	0.003285198	0.014556521	-2.147613118
ENSG00000200742	Y_RNA	0.594629	2.636367	-2.148489487
ENSG00000170409	CTA-313A17.2	0.235261	1.043512	-2.149113106
ENSG00000215478	CES5AP1	0.065251132	0.289479277	-2.149385237
ENSG00000258967	HMGN1P3	0.203255	0.902009	-2.149850986
ENSG00000236252	RP11-15J10.8	0.046054931	0.204397765	-2.149951881
ENSG00000206910	SNORA29	0.551098	2.446025	-2.150058353
ENSG00000254205	RP11-92K15.1	0.139825	0.621097	-2.151196269
ENSG00000238650	SNORD54	0.986594	4.385721	-2.152285618
ENSG00000206989	SNORD63	1.43707	6.390553	-2.152810441
ENSG00000232648	RP11-367N14.2	0.107341	0.477807	-2.154226756
ENSG00000206585	RNVU1-7	0.147723	0.658027	-2.155252315
ENSG00000127311	HELB	0.408859374	1.825368527	-2.158511138
ENSG00000162148	PPP1R32	0.917236767	4.096080757	-2.158878068
ENSG00000240167	RPS7P7	0.080222	0.358276	-2.159001565
ENSG00000250286	RP11-94C24.8	0.059133887	0.264299472	-2.160116522
ENSG00000206634	SNORA22	0.691276	3.096449	-2.163280941
ENSG00000205929	C21orf62	0.005679731	0.025470969	-2.164959393
ENSG00000226145	KRT16P6	0.197402997	0.885599672	-2.165510792
ENSG00000231504	NMD3P2	0.133761	0.600232	-2.165862697
ENSG00000254907	RP11-484D2.2	0.147977	0.664573	-2.167054725
ENSG00000088053	GP6	0.167959538	0.755389282	-2.169106586
ENSG00000259351	RP11-111E14.1	0.094956	0.427108	-2.169269853
ENSG00000277864	SCARNA15	0.061855	0.278844	-2.172496104
ENSG00000196611	MMP1	0.103343	0.46648	-2.174374558

ENSG00000152154	TMEM178A	0.304553227	1.375144183	-2.174816595
ENSG00000273674	CTD-2378E12.1	0.038137993	0.172358795	-2.176114079
ENSG00000203801	LINC00222	0.036892701	0.167099007	-2.179295853
ENSG00000185186	LINC00313	0.011050025	0.050054981	-2.179463979
ENSG00000279430	RP11-190A12.9	0.110404	0.500116	-2.179470319
ENSG00000271218	RP3-523E19.2	0.487045	2.206254	-2.179471914
ENSG00000277687	RP11-427L15.2	1.036116	4.69536	-2.180050244
ENSG00000268983	AC005253.4	0.137428	0.623141	-2.18088267
ENSG00000131943	C19orf12	0.22719217	1.031181652	-2.182313477
ENSG00000279595	RP11-854K16.4	0.021843	0.099207	-2.183270905
ENSG00000006459	KDM7A	1.479098347	6.722218146	-2.184219379
ENSG00000232978	RP11-146N23.4	0.12444	0.56562	-2.184382834
ENSG00000207280	SNORD20	1.094269	4.979089	-2.185914369
ENSG00000201176	RNU6-853P	0.237833	1.082411	-2.186227594
ENSG00000237622	TCEB1P18	0.151027	0.687347	-2.186232121
ENSG00000167046	RP11-93B14.6	0.026331	0.120058	-2.188897322
ENSG00000280554	snoU18	0.006471	0.029509	-2.189094449
ENSG00000238266	LINC00707	0.099472	0.453801	-2.189697399
ENSG00000215472	RPL17-C18orf32	9.972121491	45.52276292	-2.190615758
ENSG00000229757	RP4-738P11.4	0.054431	0.248597	-2.19130844
ENSG00000243916	RP11-6K23.1	0.011634	0.053193	-2.192889196
ENSG00000271064	RP11-792A8.3	0.212067	0.969659	-2.192957345
ENSG00000141579	ZNF750	0.123368	0.564594	-2.19424557
ENSG00000280498	SNORA16A	0.187753	0.859261	-2.194260456
ENSG00000273768	U1	0.147723	0.676507	-2.195210396
ENSG00000260034	LCMT1-AS2	0.088160619	0.404549924	-2.198111499
ENSG00000275267	MIR6884	0.179637	0.825459	-2.200112025
ENSG00000279144	RP11-894J14.2	0.108521	0.500817	-2.206309288
ENSG00000267587	RP11-687F6.4	0.081958	0.378233	-2.206318557
ENSG00000230087	AC104389.31	0.271982	1.256915	-2.208304009
ENSG00000264296	RP11-116K4.1	0.052611	0.243238	-2.208932255
ENSG00000212327	RNU6-882P	0.197655	0.914269	-2.209634253
ENSG00000232059	RP11-173A6.2	0.48372	2.239729	-2.211080087
ENSG00000135077	HAVCR2	0.013379536	0.062129865	-2.215258808
ENSG00000272410	RP11-438J1.1	0.267391505	1.242515039	-2.216237777
ENSG00000139549	DHH	0.052579	0.244434	-2.216886365
ENSG00000263548	MIR5187	0.010715	0.049848	-2.217903772
ENSG00000237170	RPS7P15	0.08614	0.401111	-2.219246302
ENSG00000231439	WASIR2	0.048288	0.224928	-2.219726649
ENSG00000270424	RP11-1348G14.6	0.262072	1.220899	-2.219908729
ENSG00000249140	PRDX2P3	0.099013	0.461779	-2.221512704
ENSG00000223374	AC005104.3	1.403808	6.556678	-2.223619415
ENSG00000270110	RP5-1139B12.4	0.199854	0.93557	-2.226899153
ENSG00000202093	SNORD58C	0.798378	3.743941	-2.229413828
ENSG00000253908	RP11-252I14.2	0.296339	1.39122	-2.231030167
ENSG00000105146	AURKC	0.333056802	1.565253994	-2.232556632
ENSG00000102409	BEX4	0.400884	1.884709852	-2.233085697
ENSG00000166147	FBN1	0.074678458	0.351259311	-2.233772425

ENSG00000182584	ACTL10	0.050194	0.236444	-2.235911707
ENSG00000259322	RP11-762H8.1	0.577632	2.722702	-2.236816515
ENSG00000254779	EGLN1P1	0.082821	0.390633	-2.237745303
ENSG00000088827	SIGLEC1	0.129907055	0.612881384	-2.238128106
ENSG00000189143	CLDN4	9.314205764	43.95581415	-2.238549348
ENSG00000241535	CBX5P1	0.090725	0.428334	-2.239164142
ENSG00000223039	RN7SKP268	0.312464	1.476326	-2.240249443
ENSG00000279372	RP11-1182P23.5	0.115092	0.543925	-2.240620181
ENSG00000234911	TEX21P	0.103448	0.48903	-2.241017218
ENSG00000267279	RP11-879F14.2	0.226974271	1.073382872	-2.241564098
ENSG00000230912	RP3-508I15.10	0.124137	0.587328	-2.242233232
ENSG00000229647	AC007879.7	0.084544982	0.40006051	-2.242427198
ENSG00000253385	KB-1254G8.1	0.132198	0.62609	-2.243669708
ENSG00000026036	RTEL1-TNFRSF6B	6.188468062	29.31008983	-2.243743166
ENSG00000224967	AC009303.3	0.03181	0.150677	-2.243906937
ENSG00000234197	ETV5-AS1	0.363544	1.724043	-2.245593867
ENSG00000182676	PPP1R27	0.070249937	0.333163704	-2.24566241
ENSG00000196352	CD55	7.109001039	33.71485175	-2.245665503
ENSG00000164949	GEM	8.009243426	38.01866299	-2.246969924
ENSG00000267560	RP11-173A16.1	0.3995	1.89761	-2.247916109
ENSG00000213204	RP3-382I10.7	0.177701591	0.844148217	-2.248039733
ENSG00000238917	SNORD10	0.069308	0.329464	-2.24902704
ENSG00000249007	RP11-510N19.5	0.287698	1.369885	-2.251427689
ENSG00000226632	UBE2V1P1	0.455779	2.171105	-2.252023141
ENSG00000278449	MIR6892	0.054768	0.26149	-2.255350673
ENSG00000160862	AZGP1	0.05246943	0.250650721	-2.256129357
ENSG00000231104	RP11-354M20.3	0.226530485	1.083580045	-2.258028611
ENSG00000219693	RP11-262H14.11	0.034861	0.16681	-2.258519919
ENSG00000207073	Y_RNA	0.141099	0.675167	-2.258536628
ENSG00000196564	RP3-499B10.3	0.068409	0.327342	-2.258540675
ENSG00000221778	AC034236.2	0.541449	2.590877	-2.258543167
ENSG00000226465	RP13-401N8.1	0.132542	0.634226	-2.258547427
ENSG00000271959	CTD-3064M3.7	0.034377	0.1645	-2.258572029
ENSG00000183638	RP1L1	0.062237154	0.29787383	-2.258853377
ENSG00000267898	CTD-2639E6.9	0.085006047	0.406915735	-2.259092698
ENSG00000205959	RP11-689P11.2	0.07659141	0.366716969	-2.259412528
ENSG00000087266	SH3BP2	0.817920537	3.91922481	-2.260535736
ENSG00000232442	CTD-3184A7.4	1.48841	7.13356	-2.260850244
ENSG00000267001	AC006538.4	0.447713	2.146446	-2.261303763
ENSG00000225854	RP11-569G9.7	0.391283	1.877013	-2.262154306
ENSG00000228705	LINC00659	3.117864	14.960671	-2.262544978
ENSG00000255252	RP1-65P5.3	0.135687	0.651154	-2.262716281
ENSG00000144644	GADL1	0.010414378	0.050059305	-2.265061642
ENSG00000200530	SNORD35B	4.186061	20.163794	-2.26810188
ENSG00000241217	RN7SL809P	0.685471	3.301899	-2.268128454
ENSG00000244558	KCNK15-AS1	0.197424196	0.953289976	-2.271616312
ENSG00000240553	RP1-184J9.2	0.030568	0.147604	-2.27163775
ENSG00000270218	RP11-325L12.5	0.114062	0.551313	-2.273053386

ENSG00000258414	RP11-356O9.1	0.015172	0.073334	-2.273070957
ENSG00000070190	DAPP1	0.078336088	0.378702761	-2.273316944
ENSG00000256006	AC084117.3	0.205641	0.994215	-2.273429937
ENSG00000180638	SLC47A2	0.025404583	0.122924091	-2.274607007
ENSG00000277846	SNORD30	0.873109	4.225537	-2.274901019
ENSG00000202392	RN7SKP292	0.160556	0.778516	-2.27765011
ENSG00000279022	RP11-250B2.4	0.459559	2.238122	-2.283966682
ENSG00000212588	SNORA26	0.400495	1.951889	-2.285014876
ENSG00000207067	SNORA72	0.227392	1.110225	-2.287598681
ENSG00000226744	AC079781.5	0.753098	3.678437	-2.288183364
ENSG00000201428	RN7SKP71	0.240076	1.17403	-2.289906182
ENSG00000196335	STK31	0.008231767	0.040264665	-2.290240273
ENSG00000115297	TLX2	0.018340722	0.089725779	-2.290472105
ENSG00000262094	AC139099.5	0.044183354	0.216243886	-2.291084488
ENSG00000251991	RNU7-49P	0.705979	3.456731	-2.291711164
ENSG00000202529	SNORD18B	0.006464	0.031663	-2.292298851
ENSG00000204677	FAM153C	0.006493377	0.031827811	-2.293247007
ENSG00000235910	APOA1-AS	0.141897	0.696577	-2.29543875
ENSG00000253564	RP11-956J14.2	0.030581	0.150206	-2.296234954
ENSG00000247157	LINC01252	0.013418603	0.065920518	-2.29649311
ENSG00000203721	LINC00862	0.038237225	0.188274238	-2.299785886
ENSG00000231519	AC007285.7	0.149348	0.735906	-2.300843579
ENSG00000160223	ICOSLG	0.133335889	0.657018337	-2.300868483
ENSG00000250027	RP11-563E2.2	0.283881	1.402618	-2.304763949
ENSG00000215895	RP11-334L9.1	20.688129	102.279945	-2.305648209
ENSG00000242147	RP13-463N16.6	0.062799	0.310591	-2.306202534
ENSG00000240005	RP11-293A21.1	0.086411277	0.428186119	-2.308946524
ENSG00000063015	SEZ6	0.02715325	0.134685925	-2.31040232
ENSG00000226059	HMGB3P20	0.028276	0.140713	-2.315105668
ENSG00000115592	PRKAG3	0.015265509	0.075970341	-2.315160637
ENSG00000228763	LIMS1-AS1	0.073623	0.366938	-2.317307874
ENSG00000145779	TNFAIP8	0.167598195	0.836495653	-2.319351434
ENSG00000207923	MIR559	0.455946	2.277573	-2.320562422
ENSG00000267203	SNRPGP4	0.831231	4.154756	-2.321442389
ENSG00000146839	ZAN	0.005756091	0.028771461	-2.321477182
ENSG00000199477	SNORA31	1.399255	6.997302	-2.322139856
ENSG00000280718	AC027506.1	0.94543	4.731909	-2.32337978
ENSG00000274604	AC006478.1	0.94543	4.731909	-2.32337978
ENSG00000231189	AC013448.1	0.12598	0.630841	-2.324081711
ENSG00000237575	PYY2	0.047301	0.237983	-2.330915931
ENSG00000178397	FAM220A	0.850427651	4.28074503	-2.331601498
ENSG00000227408	AMYP1	0.04146	0.208794	-2.332288233
ENSG00000233002	AC005324.6	0.025232908	0.127134527	-2.332977521
ENSG00000141338	ABCA8	0.018107827	0.09125523	-2.333293809
ENSG00000144045	DQX1	0.03808502	0.191985454	-2.333701432
ENSG00000185689	C6orf201	0.088717421	0.447516481	-2.334651483
ENSG00000276635	AC233280.3	0.175266	0.884448	-2.33523117
ENSG00000280162	RP11-314N13.9	0.055655	0.281107	-2.33653617

ENSG00000128254	C22orf24	0.068650407	0.347250164	-2.338635194
ENSG00000240233	RN7SL587P	0.038037	0.1926	-2.340132334
ENSG00000275881	Metazoa_SRP	0.078016	0.395041	-2.340160457
ENSG00000179564	LSMEM2	0.014308	0.07245	-2.340163666
ENSG00000233306	TRGV2	0.019647	0.099487	-2.340198984
ENSG00000210825	SNORA40	0.154085	0.780251	-2.340211877
ENSG00000242435	UPK3BP1	0.099993	0.506348	-2.340230245
ENSG00000266501	RP11-766H1.1	9.06657	45.937874	-2.341055321
ENSG00000184144	CNTN2	0.023846811	0.120891675	-2.341846672
ENSG00000269427	CTC-429P9.1	0.463777902	2.35273446	-2.342832514
ENSG00000255052	FAM66D	0.008501	0.043139	-2.343288269
ENSG00000274598	RP11-423G4.10	0.245283	1.245424	-2.344117834
ENSG00000176406	RIMS2	0.040138985	0.204189821	-2.346834921
ENSG00000272741	RP11-447L10.1	0.255942	1.302431	-2.347318126
ENSG00000265145	SNORD53	0.613364	3.122338	-2.347811322
ENSG00000273352	RP11-61L19.3	0.406627	2.071138	-2.348645765
ENSG00000215193	PEX26	0.31211557	1.589821932	-2.348712951
ENSG00000248254	AC092597.3	0.17214	0.877095	-2.349150739
ENSG00000254545	RP11-84A19.3	0.081311	0.415509	-2.353357284
ENSG00000237877	LINC01473	0.105302948	0.53915454	-2.356153038
ENSG00000242866	STRC	0.148295254	0.761481468	-2.360336498
ENSG00000223476	VN1R42P	0.213054	1.094997	-2.361635874
ENSG00000227726	AP001271.3	0.475236	2.443343	-2.362140373
ENSG00000259895	RP11-715J22.2	0.015594	0.080252	-2.363546307
ENSG00000150656	CNDP1	0.006524215	0.033637631	-2.36619992
ENSG00000179715	PCED1B	0.104393667	0.538310429	-2.366404184
ENSG00000143878	RHOB	58.064594	299.444916	-2.366560012
ENSG00000225808	DNAJC19P5	1.288512	6.645181	-2.366602523
ENSG00000253173	RP11-152C15.1	0.062621	0.323023	-2.36691844
ENSG00000225205	AC093818.1	0.721038784	3.720370617	-2.367297579
ENSG00000232466	RP11-337A23.5	0.189395	0.978135	-2.368635352
ENSG00000108950	FAM20A	0.118624291	0.612964591	-2.369404275
ENSG00000214659	KRT8P26	0.039887	0.206133	-2.369584962
ENSG00000182218	HHIPL1	0.006495014	0.033566217	-2.369605421
ENSG00000176115	AQP7P4	0.034631	0.179734	-2.375727395
ENSG00000275554	AP002802.1	0.897339	4.657697	-2.37589177
ENSG00000231726	HMGN2P38	0.172855	0.897788	-2.376812477
ENSG00000157884	CIB4	0.018437782	0.095961797	-2.379795082
ENSG00000252376	RNA5SP395	0.181156	0.944816	-2.382800807
ENSG00000250746	RP11-39C10.1	21.526432	112.452225	-2.385131089
ENSG00000111729	CLEC4A	0.432865	2.262383	-2.385854126
ENSG00000179846	NKPD1	0.010683659	0.055904109	-2.387548538
ENSG00000207389	RNU1-4	0.147723	0.773528	-2.388559048
ENSG00000207005	RNU1-2	0.147723	0.773528	-2.388559048
ENSG00000177725	CTD-2530N21.5	0.006518	0.034211	-2.391959018
ENSG00000225173	XXbac-BPG308K3.5	0.279965	1.470185	-2.392679323
ENSG00000148426	PROSER2	1.931155814	10.14147173	-2.392730554
ENSG00000198286	CARD11	0.021625938	0.11359948	-2.393121618

ENSG00000279744	RP13-20L14.10	0.803559	4.223682	-2.394025358
ENSG00000258471	RP11-84C10.4	0.355433	1.868472	-2.394209405
ENSG00000186446	ZNF501	0.013016863	0.068455659	-2.394788035
ENSG00000260898	ADPGK-AS1	0.032438977	0.170688509	-2.395565703
ENSG00000172000	ZNF556	0.015484	0.081502	-2.39605725
ENSG00000114948	ADAM23	0.015853096	0.083607066	-2.398860249
ENSG00000257310	RP11-496I2.2	0.018767	0.098981	-2.398953571
ENSG00000251434	RP11-315A17.1	0.042272	0.222955	-2.398978277
ENSG00000222020	AC062017.1	0.131806003	0.695709103	-2.400068116
ENSG00000226263	ISM1-AS1	0.078062	0.412071	-2.400200605
ENSG00000246422	CTD-2024I7.13	0.061568	0.325008	-2.400222621
ENSG00000277566	RP11-545P7.9	0.264625	1.397566	-2.400895147
ENSG00000124406	ATP8A1	0.165100516	0.871960442	-2.400918057
ENSG00000213809	KLRK1	0.16610805	0.878820693	-2.403446847
ENSG00000236973	GAPDHP51	0.025448	0.13476	-2.404768151
ENSG00000131771	PPP1R1B	0.067057182	0.355574465	-2.406687949
ENSG00000130818	ZNF426	0.027047625	0.143516288	-2.407640641
ENSG00000259469	RP11-227D13.4	0.094253	0.500619	-2.409102603
ENSG00000230657	PRB4	0.005557	0.029542	-2.410389355
ENSG00000204866	IGFL2	0.020979918	0.111542442	-2.410511851
ENSG00000260467	RP11-405F3.4	0.012661	0.067314	-2.410515231
ENSG00000250869	RP11-453N18.1	0.029251	0.155519	-2.410532996
ENSG00000273008	RP11-351D16.3	0.033684	0.179088	-2.410533296
ENSG00000229463	LYST-AS1	0.019538	0.103878	-2.410535444
ENSG00000222726	RNU2-7P	0.057187	0.304048	-2.41053997
ENSG00000234699	RP11-225H22.4	0.077468	0.411877	-2.410541167
ENSG00000260357	DNM1P34	0.007360219	0.039132389	-2.410542647
ENSG00000241765	RPS26P45	0.149695	0.79589	-2.410543015
ENSG00000244018	RPL35P6	0.136818	0.727427	-2.410544429
ENSG00000259262	NDUFA3P4	0.203585	1.082411	-2.410545232
ENSG00000238168	RP11-686G8.1	0.128851	0.68507	-2.410547675
ENSG00000280662	AC092069.1	0.273635	1.454853	-2.41054871
ENSG00000230929	RP11-395C3.1	0.084545	0.449506	-2.410549032
ENSG00000172399	MYOZ2	0.019598	0.104198	-2.410549248
ENSG00000250075	RP11-584P21.2	0.008905672	0.047349394	-2.410549597
ENSG00000232077	LINC01031	0.065335	0.347372	-2.410553513
ENSG00000198650	TAT	0.018825926	0.100093593	-2.410556879
ENSG00000021461	CYP3A43	0.019300633	0.10261751	-2.410556896
ENSG00000281796	EHMT1-IT1	0.0291	0.154719	-2.410559317
ENSG00000007350	TKTL1	0.013943969	0.074137415	-2.410560519
ENSG00000279665	RP11-507J18.5	0.044257	0.235306	-2.410560543
ENSG00000183784	C9orf66	0.017442	0.092736	-2.410564023
ENSG00000260599	CTC-457E21.1	0.01517	0.080657	-2.410578661
ENSG00000176222	ZNF404	0.020530495	0.109158226	-2.410580572
ENSG00000233402	TARDBPP1	0.021153	0.11247	-2.41060604
ENSG00000254266	PKIA-AS1	0.007664666	0.040753793	-2.410639511
ENSG00000216977	RPL21P65	0.228875	1.220443	-2.414773125
ENSG00000044574	HSPA5	190.216049	1015.214783	-2.416074102

ENSG00000244482	LILRA6	0.043068737	0.229880971	-2.416174118
ENSG00000220392	FCF1P5	0.105593	0.563662	-2.416316111
ENSG00000270108	RP11-73M18.6	2.839861	15.161593	-2.41652912
ENSG00000260459	FTLP14	0.210315	1.124902	-2.419175667
ENSG00000250271	RP11-64D22.5	2.133433	11.412753	-2.419398134
ENSG00000165091	TMC1	0.032439164	0.173541323	-2.419470703
ENSG00000117242	PINK1-AS	0.03039	0.162601	-2.41966755
ENSG00000235085	AC091153.4	0.080863402	0.432970486	-2.420709882
ENSG00000197142	ACSL5	2.574638092	13.79314995	-2.421510407
ENSG00000176601	MAP3K19	0.016937752	0.090825652	-2.42285742
ENSG00000252874	Y_RNA	1.560817	8.370643	-2.423037053
ENSG00000099219	ERMP1	4.429792266	23.76533883	-2.42354802
ENSG00000225767	RP5-850O15.3	0.246892	1.327461	-2.426717481
ENSG00000163945	UVSSA	0.670605899	3.611986554	-2.429255443
ENSG00000183696	UPP1	1.296762842	6.990194718	-2.430417987
ENSG00000267571	AC104532.4	0.067363	0.363866	-2.433378955
ENSG00000206588	RNU1-28P	0.147723	0.798608	-2.434593058
ENSG00000228137	AP001469.7	0.182704	0.990314	-2.438377815
ENSG00000135870	RC3H1	1.115614941	6.04833745	-2.438699472
ENSG00000267504	RP11-845C23.2	0.090605	0.491558	-2.439699081
ENSG00000119125	GDA	5.323926689	28.88980635	-2.439997923
ENSG00000225636	HNRNPA3P15	0.024803	0.135037	-2.444768224
ENSG00000200485	Y_RNA	0.045863	0.249787	-2.445295762
ENSG00000239486	RP11-163E9.1	0.257344	1.401759	-2.445468281
ENSG00000277918	U1	0.060206	0.328023	-2.445817801
ENSG00000209582	SNORA48	0.186796	1.01789	-2.446046196
ENSG00000279700	RP13-554M15.2	0.315519	1.723869	-2.449851356
ENSG00000212432	SNORA75	0.359942	1.967354	-2.450420217
ENSG00000166278	C2	0.076830339	0.42082267	-2.453464404
ENSG00000100170	SLC5A1	0.019169848	0.105220192	-2.456500777
ENSG00000139160	METTL20	0.287802898	1.579822446	-2.456609399
ENSG00000237162	RP11-443F16.1	0.033284	0.182859	-2.457830906
ENSG00000251194	RP1-68D18.2	0.542062	2.980158	-2.458859042
ENSG00000259712	CTD-2184D3.5	0.881667	4.848685	-2.459287763
ENSG00000218459	RPS27P15	0.124716	0.686247	-2.460081375
ENSG00000199476	Y_RNA	0.207355	1.142545	-2.462076247
ENSG00000197013	ZNF429	0.004371063	0.024103771	-2.463202715
ENSG00000259644	RP11-680F8.4	0.264691	1.463586	-2.467126474
ENSG00000272195	RP11-156E8.1	0.058535	0.323665	-2.467129943
ENSG00000207344	SNORA22	0.121543	0.673141	-2.469441925
ENSG00000222937	SNORD63	4.900578	27.214901	-2.473372965
ENSG00000279283	RP11-417L19.5	0.204754	1.138397	-2.475040224
ENSG00000222047	C10orf55	0.160637078	0.894723962	-2.477637726
ENSG00000207525	Y_RNA	1.945142	10.892349	-2.485367729
ENSG00000263307	RP11-166B2.8	0.094185	0.528116	-2.487285632
ENSG00000281656	AC073130.2	0.212067	1.191601	-2.490309195
ENSG00000249532	RP11-148B6.1	0.605563742	3.407436703	-2.492336126
ENSG00000251023	RP11-549J18.1	0.112207	0.631892	-2.49351532

ENSG00000234818	AC092687.5	0.094926	0.534909	-2.494418281
ENSG00000232987	LINC01219	0.281973	1.592078	-2.497282088
ENSG00000260464	RP4-561L24.3	0.027091	0.153229	-2.499803813
ENSG00000117407	ARTN	1.29491972	7.329922685	-2.500935322
ENSG00000234203	RP5-1050D4.2	0.115936	0.656274	-2.500969662
ENSG00000269652	CTD-2195B23.3	0.246176	1.393815	-2.501277062
ENSG00000229348	HYI-AS1	0.179304	1.016114	-2.502582692
ENSG00000278144	NEAT1_1	0.18396	1.042902	-2.503139588
ENSG00000243531	RP11-740N7.3	0.06362	0.360803	-2.503659057
ENSG00000268095	ZNF649-AS1	0.022855	0.129717	-2.50478584
ENSG00000161405	IKZF3	0.01190028	0.067596603	-2.505955215
ENSG00000254477	AP000640.10	0.057903	0.32964	-2.509181314
ENSG00000175592	FOSL1	13.44515538	76.56387806	-2.509577478
ENSG00000214402	LCNL1	0.037431332	0.213155364	-2.509587082
ENSG00000105605	CACNG7	0.03671712	0.20913696	-2.50992324
ENSG00000228677	TTC3-AS1	0.109494	0.625639	-2.514478632
ENSG00000270462	RP11-342K6.3	0.122938	0.703305	-2.516219554
ENSG00000224177	LINC00570	0.030846327	0.176466309	-2.516222141
ENSG00000189433	GJB4	0.106093	0.60793	-2.518575744
ENSG00000202031	SNORD38A	1.608394	9.217499	-2.518754495
ENSG00000240322	RN7SL481P	0.146391	0.839864	-2.520328869
ENSG00000278658	MIR6826	0.11945	0.690313	-2.5308438
ENSG00000199975	RN7SKP243	0.151535	0.875736	-2.530844968
ENSG00000180190	TDRP	0.012959653	0.074896513	-2.530871432
ENSG00000224666	RP1-50J22.4	0.031457618	0.181900834	-2.531670831
ENSG00000250080	CTB-88F18.2	0.029385	0.169986	-2.532264128
ENSG00000259797	RP11-96D1.3	0.118916	0.688098	-2.532671209
ENSG00000240373	SEC62-AS1	0.547225	3.17129	-2.534863765
ENSG00000265706	SNORD53_SNORD92	0.495911	2.886429	-2.541132607
ENSG00000277102	MIR6836	0.001475	0.008591	-2.542111118
ENSG00000213085	CFAP45	0.240958333	1.404683142	-2.543389135
ENSG00000270300	PHACTR2P1	0.434094	2.531493	-2.543909108
ENSG00000140675	SLC5A2	0.176619745	1.030058279	-2.544007421
ENSG00000237862	RP1-63G5.7	0.152287	0.889615	-2.546388323
ENSG00000242329	RP11-59H1.1	0.184406	1.078489	-2.548053959
ENSG00000278195	SSTR3	0.022515085	0.131679966	-2.548072033
ENSG00000264853	RP11-16C1.2	0.018242	0.106689	-2.548075621
ENSG00000205108	FAM205A	0.012046	0.070453	-2.548106976
ENSG00000243810	RP5-1096J16.1	0.032258	0.188894	-2.549847978
ENSG00000257759	RP11-486O13.4	0.041438	0.243046	-2.552203112
ENSG00000230306	BANF1P2	0.096394	0.565805	-2.553289671
ENSG00000254856	NDUFA3P2	0.057038	0.335921	-2.558126687
ENSG00000278527	SNORD22	0.308325	1.816151	-2.55836038
ENSG00000249786	EAF1-AS1	0.0622618	0.366835012	-2.558712149
ENSG00000224316	GTF2IP5	0.06684	0.393873	-2.558946885
ENSG00000279863	RP11-521C22.2	0.693164	4.087362	-2.559901388
ENSG00000221170	MIR1304	0.094808	0.559336	-2.560634483
ENSG00000228369	TXNDC12-AS1	0.046861	0.276831	-2.562545861

ENSG00000280965	AC091167.2	0.109063	0.644292	-2.562552936
ENSG00000273811	AC005052.1	0.177889	1.050884	-2.562554221
ENSG00000273442	AC006946.17	0.133299	0.787468	-2.562555341
ENSG00000262099	CTC-524C5.5	0.023018	0.135981	-2.562570694
ENSG00000267709	AC024592.9	0.103272	0.610817	-2.564291064
ENSG00000169306	IL1RAPL1	0.235887673	1.398092737	-2.567288127
ENSG00000277039	Metazoa_SRP	0.0757	0.448721	-2.567453498
ENSG00000160588	MPZL3	0.909746388	5.397459	-2.568744057
ENSG00000140749	IGSF6	0.027667067	0.164192782	-2.569149098
ENSG00000250106	ANKRD33B-AS1	0.097502	0.579148	-2.570428355
ENSG00000228988	RP4-677H15.4	0.11137	0.663007	-2.573663439
ENSG00000227719	AC006042.6	0.450684	2.683677	-2.574022905
ENSG00000252558	RNU6-914P	0.164985	0.983111	-2.575019452
ENSG00000246731	MGC16275	0.118532723	0.706871652	-2.576162889
ENSG00000271086	NAMA	0.01966441	0.117351539	-2.577177928
ENSG00000267491	CTD-2373H9.5	0.252057	1.50629	-2.579177627
ENSG00000070526	ST6GALNAC1	0.014760683	0.088262638	-2.580043384
ENSG00000278456	RP11-66B24.9	3.43369	20.53512	-2.580261677
ENSG00000263506	MIR5193	0.051363	0.307842	-2.583388703
ENSG00000265020	MIR4651	1.00E-06	6.00E-06	-2.584962501
ENSG00000222255	RNU6-101P	0.479599	2.880232	-2.586284468
ENSG00000266341	RP5-890E16.4	0.2036	1.22341	-2.587098507
ENSG00000207129	RNA5SP187	0.615887	3.702026	-2.587577446
ENSG00000240238	RP11-112N19.2	0.010625	0.064026	-2.59119504
ENSG00000198883	PNMA5	0.022199366	0.134396353	-2.597903619
ENSG00000218896	TUBB8P2	0.220037	1.336066	-2.602173233
ENSG00000165810	BTNL9	0.172799838	1.050174859	-2.603455795
ENSG00000261840	RP11-146F11.1	0.086009113	0.523484643	-2.605585778
ENSG00000052850	ALX4	0.035138	0.21416	-2.607585061
ENSG00000164106	SCRG1	0.009432228	0.057570504	-2.609659403
ENSG00000231304	SGOL1-AS1	0.004802277	0.029315916	-2.609893631
ENSG00000256538	RP11-847H18.3	0.064101	0.392473	-2.614174639
ENSG00000179038	RP11-157K17.5	0.031830222	0.194955891	-2.614678626
ENSG00000167612	ANKRD33	0.013737345	0.084430867	-2.619667284
ENSG00000262413	RP11-498C9.3	0.061411369	0.377982773	-2.621742819
ENSG00000110448	CD5	0.015801	0.097454	-2.624705537
ENSG00000275520	LINC00684	0.029552161	0.182362213	-2.62547127
ENSG00000267138	AC005954.3	0.146119	0.901901	-2.625825295
ENSG00000157856	DRC1	0.033397216	0.206325574	-2.627122899
ENSG00000174876	AMY1B	0.022533072	0.139215217	-2.627200999
ENSG00000255587	RAB44	0.032456655	0.200595561	-2.627703471
ENSG00000230417	LINC00856	0.01621447	0.100939073	-2.638130931
ENSG00000197953	AADACL2	0.001405356	0.008751448	-2.638586137
ENSG00000251056	ANKRD20A17P	0.009719	0.060686	-2.642483945
ENSG00000199377	RNU5F-1	0.069602	0.434814	-2.643197725
ENSG00000149970	CNKSR2	0.001456581	0.009108381	-2.644608704
ENSG00000225745	AL773572.7	0.01413748	0.088431939	-2.645042495
ENSG00000133958	UNC79	0.020174467	0.126426015	-2.647690928

ENSG00000138166	DUSP5	52.83871859	331.5260089	-2.649454672
ENSG00000232788	AC078883.3	0.127229974	0.798286162	-2.649467408
ENSG00000216817	R3HDM2P2	0.012027	0.075707	-2.654149877
ENSG00000162630	B3GALT2	0.015546	0.09786	-2.654175861
ENSG00000237347	AC004461.4	0.063147	0.397511	-2.654208683
ENSG00000002587	HS3ST1	1.190320871	7.493246573	-2.654240397
ENSG00000205936	PPP1R12BP2	0.026549	0.167133	-2.654267192
ENSG00000254884	PRR13P2	0.371066	2.34078	-2.657241627
ENSG00000237399	PITRM1-AS1	0.204878136	1.293293318	-2.658211577
ENSG00000224323	DPRXP1	0.090728	0.574149	-2.661805424
ENSG00000266079	SNORA59B	0.036302631	0.230070106	-2.663927541
ENSG00000168542	COL3A1	0.007472592	0.047359242	-2.663965286
ENSG00000151962	RBM46	0.070523764	0.447033296	-2.664200909
ENSG00000274124	RP11-474C8.8	0.084827	0.537736	-2.664302614
ENSG00000233286	MTND3P10	0.149256	0.949093	-2.668760533
ENSG00000259818	RP5-1024G6.7	0.014174	0.090427	-2.673506648
ENSG00000243402	RP11-473O4.1	0.088516	0.564736	-2.673566438
ENSG00000172232	AZU1	0.014272758	0.09106089	-2.673567383
ENSG00000087245	MMP2	0.010317516	0.065826584	-2.673574707
ENSG00000237827	RP11-332O19.2	0.127241	0.811808	-2.673574942
ENSG00000267423	AC005616.2	0.087904	0.560835	-2.673575666
ENSG00000272541	XXbac-BPGBPG55C20.1	0.044028	0.280903	-2.673578817
ENSG00000261868	MFSD1P1	0.187169	1.194159	-2.673581529
ENSG00000274038	RP11-66H6.4	0.146632	0.935529	-2.673582393
ENSG00000233797	UFL1-AS1	0.092876	0.59256	-2.673583498
ENSG00000261584	RP11-457M11.5	0.014761	0.094177	-2.673584305
ENSG00000089199	CHGB	0.011222968	0.071603943	-2.673584766
ENSG00000171942	OR10H2	0.016297	0.103977	-2.673586117
ENSG00000248521	RP11-174F8.1	0.025499	0.162687	-2.673586398
ENSG00000280023	LLNLR-276H7.1	0.061173	0.390292	-2.673586957
ENSG00000127951	FGL2	0.070951	0.452677	-2.673587084
ENSG00000267430	RP11-635N19.2	0.044142	0.281634	-2.67359761
ENSG00000240211	RP11-758P17.3	0.058628	0.374556	-2.675519685
ENSG00000276952	RP5-965G21.6	0.687174	4.392276	-2.676221356
ENSG00000220563	PKMP3	0.082573	0.529851	-2.681844688
ENSG00000250277	RP11-468N14.11	0.031523	0.202516	-2.683559148
ENSG00000241003	RP11-22C11.1	0.100995	0.650011	-2.686180262
ENSG00000221290	MIR1182	1.023999	6.594894	-2.687135165
ENSG00000262888	RP11-462G12.2	0.0217	0.139846	-2.688072041
ENSG00000274544	SNORD28	0.312564	2.01519	-2.68869234
ENSG00000233924	RPSAP13	0.058167	0.376061	-2.692693894
ENSG00000162998	FRZB	0.211406	1.36722	-2.693157178
ENSG00000238364	RNU7-140P	0.475032	3.08487	-2.699113086
ENSG00000207234	RNU6-125P	0.875224	5.685186	-2.699483345
ENSG00000272529	RP11-415F23.4	0.025863	0.168943	-2.707575038
ENSG00000270909	RP11-24B13.2	0.042202	0.27597	-2.709128167
ENSG00000224745	RP11-380G5.2	0.177474	1.165717	-2.715537999
ENSG00000276528	HOTAIRM1_1	0.02443	0.160815	-2.718676211

ENSG00000154874	CCDC144B	0.018162744	0.119630431	-2.719530367
ENSG00000259248	USP3-AS1	0.080296483	0.52984275	-2.722155544
ENSG00000166763	STRCP1	0.105161	0.69428	-2.722917847
ENSG00000125804	FAM182A	0.017500645	0.115696013	-2.724859192
ENSG00000204055	RP11-247A12.2	0.060791785	0.402119193	-2.725674923
ENSG00000199574	SNORD18C	0.073873	0.489155	-2.727172617
ENSG00000249216	RP11-227F19.5	0.018721	0.124416	-2.732442624
ENSG00000266467	RN7SL220P	0.027145	0.180402	-2.732456944
ENSG00000250651	PABPC1P7	0.067279	0.447845	-2.734771332
ENSG00000134539	KLRD1	0.023102826	0.153901543	-2.735866425
ENSG00000202314	SNORD6	3.618749	24.198553	-2.741357832
ENSG00000200237	SNORA70	0.479939	3.212687	-2.742857475
ENSG00000259113	RP11-406H23.2	0.108006	0.723304	-2.74349067
ENSG00000267708	RP11-147L13.7	0.08219	0.550935	-2.74484734
ENSG00000206737	RNVU1-18	0.093102	0.625026	-2.74703214
ENSG00000275519	MIR6804	0.14221	0.955068	-2.747580539
ENSG00000234478	RP11-275I14.4	0.194944	1.310756	-2.749267493
ENSG00000272896	RP11-216L13.17	0.111881	0.754095	-2.75278123
ENSG00000180861	LINC01559	0.012875538	0.087010116	-2.756550468
ENSG00000226801	OSTCP8	0.113607	0.768318	-2.757651823
ENSG00000177034	MTX3	1.046547789	7.110406583	-2.764293866
ENSG00000164841	TMEM74	0.052364947	0.357416952	-2.770934755
ENSG00000268455	RP11-359H18.2	0.007977	0.054517	-2.772787992
ENSG00000212385	RNU6-817P	0.068514	0.46835	-2.773116345
ENSG00000279897	BIRC6-AS2	0.086412	0.591959	-2.776193678
ENSG00000199545	RNA5SP195	0.43501	2.988194	-2.780153343
ENSG00000216938	RPL7P58	0.031559	0.217124	-2.782395807
ENSG00000099834	CDHR5	0.13004542	0.894908024	-2.782723825
ENSG00000214145	LINC00887	0.110297828	0.761666728	-2.787755493
ENSG00000215182	MUC5AC	0.002917	0.020162	-2.789081465
ENSG00000276266	CH17-80A12.1	0.040298	0.278752	-2.790202012
ENSG00000273733	CTC-510F12.7	0.103658	0.718668	-2.793493985
ENSG00000160200	CBS	0.001725265	0.011962959	-2.793684438
ENSG00000274276	CBSL	0.002923988	0.020274885	-2.793684438
ENSG00000257769	CTB-193M12.1	0.192061	1.340392	-2.803018482
ENSG00000270953	RP11-2E11.9	0.844072	5.897003	-2.804543956
ENSG00000155761	SPAG17	0.016962987	0.118512443	-2.80457637
ENSG00000207494	Y_RNA	0.203585	1.422597	-2.804823853
ENSG00000271779	RP11-87N3.6	1.408195	9.848653	-2.806079295
ENSG00000255968	RP11-513G19.1	0.065672	0.460007	-2.808305518
ENSG00000233937	CTC-338M12.4	0.229646981	1.620359834	-2.818824504
ENSG00000228203	RNF144A-AS1	0.005475196	0.038657488	-2.819765432
ENSG00000173846	PLK3	5.847756387	41.2917315	-2.819897801
ENSG00000276381	Z98750.1	1.001767	7.095804	-2.824419168
ENSG00000269925	RP3-467L1.6	0.259014	1.837069	-2.82630383
ENSG00000216090	MIR937	0.076417	0.542947	-2.828845849
ENSG00000268439	RP3-461F17.3	0.408882717	2.909840385	-2.831181028
ENSG00000242602	CTD-2339M3.1	0.432137	3.077042	-2.83198347

ENSG00000107807	TLX1	0.014877429	0.105994672	-2.832794631
ENSG00000268884	CTD-2550O8.7	0.014909	0.106236	-2.833017333
ENSG00000174572	RP11-209A2.1	0.010852	0.077546	-2.837091413
ENSG00000256826	AC138744.2	0.444786	3.181017	-2.838304798
ENSG00000258938	RP11-317N8.5	0.035505	0.25411	-2.839359039
ENSG00000276517	AL133243.2	0.234488	1.681267	-2.841962856
ENSG00000172478	C2orf54	0.036722206	0.264168836	-2.846735636
ENSG00000259521	RP11-540O11.4	0.028593573	0.205934933	-2.848425759
ENSG00000272414	FAM47E-STBD1	0.108951073	0.784706269	-2.848472315
ENSG00000229771	RP4-644L1.2	0.030495	0.219855	-2.849907722
ENSG00000272574	RP11-359K18.4	0.33014	2.384535	-2.852558106
ENSG00000274153	MIR6727	0.09679	0.699404	-2.853196141
ENSG00000222399	RNU6-791P	0.046981	0.340335	-2.856806199
ENSG00000260558	RP11-63M22.1	0.051276	0.371574	-2.857293928
ENSG00000272379	RP1-257A7.5	0.047346	0.343259	-2.857983097
ENSG00000137193	PIM1	6.523386698	47.30695368	-2.858359205
ENSG00000279689	RP11-574K11.32	0.425497	3.090707	-2.860716028
ENSG00000260427	AGGF1P9	0.055747	0.405064	-2.861183797
ENSG00000258096	RP11-474P2.2	0.081174	0.590474	-2.862783924
ENSG00000188931	CFAP126	0.017306	0.126194	-2.866299105
ENSG00000213640	EEF1DP4	0.032388	0.238291	-2.879193176
ENSG00000120498	TEX11	0.015098067	0.111098355	-2.879401661
ENSG00000223642	AC008277.1	0.04376003	0.322150522	-2.880049307
ENSG00000230171	RPL22P18	0.135003	0.996277	-2.883555451
ENSG00000213999	MEF2B	0.487791311	3.607133245	-2.886516751
ENSG00000227516	RP5-973N23.4	0.126293	0.934688	-2.887710194
ENSG00000183704	SLC9B1P1	0.019034	0.141002	-2.889064945
ENSG00000237329	RP11-201O14.1	0.100387	0.745091	-2.891844183
ENSG00000282413	RP11-359E7.3	0.29874	2.221365	-2.89448414
ENSG00000154764	WNT7A	0.132047772	0.982537512	-2.895452529
ENSG00000278895	CTC-448F2.7	0.012925	0.096203	-2.895917603
ENSG00000253284	RP11-282K24.3	0.009441	0.070273	-2.895958905
ENSG00000213180	RPL36AP48	0.031769	0.236475	-2.895996087
ENSG00000221878	PSG7	0.001219414	0.009079293	-2.896391783
ENSG00000250260	RP11-325L7.2	0.02659	0.198223	-2.898168687
ENSG00000179094	PER1	6.292927434	46.96959887	-2.899924061
ENSG00000241738	ZNF90P1	0.036	0.269076	-2.901944905
ENSG00000140450	ARRDC4	8.547112	63.990227	-2.904342652
ENSG00000278931	bP-2189O9.2	0.015343	0.11493	-2.905102927
ENSG00000282863	RP11-90J7.3	0.007182668	0.053962877	-2.909375543
ENSG00000260977	RP11-333I13.1	0.103321	0.776541	-2.909928587
ENSG00000206596	RNU1-27P	0.116689	0.879129	-2.913406308
ENSG00000184163	FAM132A	0.199701645	1.507751862	-2.916480895
ENSG00000186300	ZNF555	0.112477744	0.850901	-2.919351727
ENSG00000271623	RP11-435I10.5	0.117206	0.888635	-2.922544539
ENSG00000280008	RP11-652L8.4	0.020654	0.156872	-2.925094753
ENSG00000260452	TPRKBP2	0.015001	0.113938	-2.925118405
ENSG00000251620	STPG2-AS1	0.013269	0.100783	-2.925120753

ENSG00000226699	RP11-122K13.7	0.105112	0.80126	-2.930343075
ENSG00000255980	AP000439.1	0.020564	0.157213	-2.934527697
ENSG00000272840	RP11-379B18.6	0.049122	0.37571	-2.935178309
ENSG00000209042	SNORD12C	0.535689	4.110625	-2.939890188
ENSG00000262623	RP5-1107A17.2	0.057104	0.439301	-2.943546072
ENSG00000177483	RBM44	0.122549622	0.945102907	-2.947105385
ENSG00000135443	KRT85	0.004690158	0.036239592	-2.949858129
ENSG00000279901	CTD-2270P14.2	0.102161	0.791419	-2.953597149
ENSG00000229190	RP11-390B4.3	0.091376	0.70943	-2.956773145
ENSG00000261068	RP11-7K24.3	0.596593	4.634021	-2.957445627
ENSG00000262999	CTD-3088G3.6	0.005806	0.04512	-2.958150591
ENSG00000254966	RP11-1081L13.4	0.097833	0.761542	-2.960530518
ENSG00000201499	RNU6-312P	0.402195	3.133997	-2.962036747
ENSG00000261200	RP11-989E6.10	0.025634	0.1998	-2.96242606
ENSG00000229152	ANKRD10-IT1	7.350646	57.350937	-2.963874108
ENSG00000238149	AC104978.1	0.032542	0.254685	-2.968339167
ENSG00000264290	RP11-68I3.4	0.04597	0.360033	-2.969364576
ENSG00000259083	RP11-407N17.4	0.036492	0.286181	-2.971275765
ENSG00000252355	RN7SKP287	0.33293	2.612435	-2.972104361
ENSG00000158055	GRHL3	0.152946878	1.200813925	-2.97291005
ENSG00000279862	RP11-423E7.2	0.067668	0.532308	-2.975715595
ENSG00000204581	AC096670.3	0.017795355	0.140011574	-2.975973506
ENSG00000199293	SNORA21	0.033177	0.261257	-2.97721435
ENSG00000226476	RP11-776H12.1	0.001293741	0.010190144	-2.977554071
ENSG00000213714	FAM209B	0.098272	0.774376	-2.978181918
ENSG00000181690	PLAG1	0.148666534	1.171817415	-2.978595973
ENSG00000231794	AC009542.2	0.020333902	0.160749663	-2.982856737
ENSG00000242199	RP11-71H17.1	0.060411	0.478573	-2.985855832
ENSG00000264549	SNORD95	0.092612	0.735533	-2.989519025
ENSG00000143199	ADCY10	0.009948609	0.079037177	-2.989964706
ENSG00000227245	RP11-136C24.2	0.039997	0.318981	-2.995506794
ENSG00000214803	RP11-37N22.1	0.017036	0.135867	-2.99553655
ENSG00000126860	EVI2A	0.02457605	0.196254177	-2.997398384
ENSG00000166825	ANPEP	0.015255658	0.12202337	-2.999741129
ENSG00000182111	ZNF716	0.002513	0.020133	-3.002079587
ENSG00000212743	RP11-563J2.3	0.020799	0.166763	-3.00321316
ENSG00000271852	SNORD11B	0.41848	3.391553	-3.018715458
ENSG00000262772	RP11-353N14.2	0.028465	0.231254	-3.022217315
ENSG00000229853	RP5-1049G16.4	0.059182	0.480904	-3.022518569
ENSG00000124664	SPDEF	0.077647476	0.631540927	-3.023865288
ENSG00000234068	PAGE2	0.06262561	0.510111623	-3.025988325
ENSG00000248954	RP11-304F15.4	0.055492	0.452576	-3.027808375
ENSG00000219433	BTBD10P2	0.112847	0.921795	-3.030077877
ENSG00000275149	RP11-427J23.1	0.050307	0.411445	-3.031868527
ENSG00000204529	GUCY2EP	0.012615393	0.103185803	-3.031987457
ENSG00000225194	LINC00092	0.00573863	0.046938784	-3.03200229
ENSG00000252652	Y_RNA	0.275687	2.255022	-3.032038365
ENSG00000261341	CTD-2568A17.1	0.021872678	0.17891094	-3.032039858

ENSG00000163520	FBLN2	0.031307295	0.256431052	-3.034000222
ENSG00000278496	RP11-144H23.2	0.028693	0.236823	-3.045038479
ENSG00000267174	CTC-510F12.4	0.044580701	0.369213724	-3.049964982
ENSG00000254972	RP11-167J8.3	0.02139	0.17742	-3.052160262
ENSG00000269984	RP11-362K14.5	0.467407	3.899641	-3.06059007
ENSG00000110852	CLEC2B	0.019293518	0.161459401	-3.064983324
ENSG00000238387	snoU13	0.073635	0.616225	-3.064993641
ENSG00000225731	AP001627.1	0.029017	0.242886	-3.06530906
ENSG00000279765	RP11-437B10.1	0.107520778	0.900118413	-3.065499321
ENSG00000249715	FER1L5	0.016617321	0.139163681	-3.066023021
ENSG00000203497	PDCD4-AS1	0.065419	0.548067	-3.066570659
ENSG00000275562	RP11-3K24.3	0.042825	0.359118	-3.067932815
ENSG00000115523	GNLY	0.033363846	0.282398822	-3.081376565
ENSG00000239149	SNORA59A	0.167422	1.417104	-3.081384618
ENSG00000280037	RP11-434P11.1	0.099213	0.841875	-3.085004964
ENSG00000273951	RP4-620E11.8	0.077704	0.659361	-3.085007785
ENSG00000211891	IGHE	0.036046	0.306632	-3.088597185
ENSG00000152580	IGSF10	0.004340167	0.036920471	-3.088598639
ENSG00000254722	FAM8A2P	0.014856	0.126376	-3.088604883
ENSG00000162873	KLHDC8A	0.013940164	0.118587986	-3.088638386
ENSG00000169302	STK32A	0.006544977	0.055679119	-3.08867635
ENSG00000273862	AC004477.1	0.075012	0.638345	-3.08914304
ENSG00000280039	RN7SKP23	0.131391	1.118255	-3.089310846
ENSG00000269845	RP11-420K14.6	0.018906244	0.162978018	-3.107742701
ENSG00000142615	CELA2A	0.050259512	0.43441516	-3.111605877
ENSG00000254592	RP5-1173A5.1	0.050701	0.438492	-3.112464414
ENSG00000237522	NONOP2	0.317533	2.746914	-3.112833305
ENSG00000241529	RN7SL767P	0.021196	0.18346	-3.113601607
ENSG00000278184	NF1P11	0.005051	0.043788	-3.11589461
ENSG00000197993	KEL	0.01297405	0.112544184	-3.11679064
ENSG00000265072	MIR3652	0.790008	6.8541	-3.117028073
ENSG00000255847	RP11-167N4.2	0.036046	0.31283	-3.117467793
ENSG00000263892	AL357874.1	0.000975	0.008469	-3.118717506
ENSG00000267255	CTB-50L17.5	0.064403	0.559717	-3.119497768
ENSG00000212418	RNY4P36	0.106034	0.924559	-3.124238445
ENSG00000227766	HCG4P5	0.054005	0.473615	-3.132549885
ENSG00000204837	RP11-204M4.2	0.035274	0.309488	-3.133206383
ENSG00000249379	RP1-27K12.4	0.482198	4.233626	-3.134196255
ENSG00000236878	MTATP6P26	0.076883	0.67528	-3.134749293
ENSG00000229601	AL590762.10	0.20117	1.771218	-3.138254708
ENSG00000278324	PVT1_3	0.080049	0.706814	-3.142375331
ENSG00000200488	RN7SKP203	0.114977	1.01639	-3.144036888
ENSG00000258927	RP11-1070N10.5	0.003564	0.031576	-3.147259182
ENSG00000203855	HSD3BP4	0.00818	0.072483	-3.14746992
ENSG00000227852	RP11-156G14.6	0.036793	0.326028	-3.147492652
ENSG00000252081	RNU6-277P	0.057619	0.510571	-3.147495069
ENSG00000126861	OMG	0.026529679	0.235551923	-3.150365964
ENSG00000141434	MEP1B	0.019704362	0.175451531	-3.154485565

ENSG00000206680	SNORD21	1.178328	10.498838	-3.155416571
ENSG00000235453	TOPORS-AS1	0.079921572	0.712996986	-3.157239114
ENSG00000258417	RP11-240B13.2	0.002109	0.01884	-3.159167965
ENSG00000207468	SNORA19	0.397627	3.568573	-3.165859657
ENSG00000279685	MAPT-IT1	0.016875	0.151451	-3.165891697
ENSG00000079112	CDH17	0.053753456	0.483322913	-3.168557965
ENSG00000125385	RP11-297B17.2	0.035362	0.318277	-3.170011128
ENSG00000207973	MIR589	0.164513	1.486948	-3.176080699
ENSG00000255240	RP11-142C4.6	0.013762958	0.124983084	-3.182870332
ENSG00000225163	LINC00618	0.189586	1.722442656	-3.183531616
ENSG00000235908	RHOA-IT1	0.409095	3.717762	-3.183926607
ENSG00000264294	SNORD55	0.141825	1.292022	-3.187446867
ENSG00000274448	AL109620.1	0.087678	0.801007	-3.191528056
ENSG00000242958	RP11-662B19.1	0.148179	1.356439	-3.194411262
ENSG00000281114	AL593848.2	0.193779	1.775029	-3.195358457
ENSG00000275381	RP11-401N16.2	0.100595	0.924506	-3.200124084
ENSG00000255150	EID3	0.207896	1.912702	-3.201678213
ENSG00000277264	MIR6833	0.025031	0.230678	-3.204092586
ENSG00000108556	CHRNE	0.019271889	0.178275203	-3.209536116
ENSG00000183598	HIST2H3D	0.030959	0.286843	-3.211830534
ENSG00000278202	RP11-152F13.8	0.166685	1.545188	-3.212586192
ENSG00000183134	PTGDR2	0.006132	0.057058	-3.217999576
ENSG00000145975	FAM217A	0.016219365	0.152015235	-3.228426659
ENSG00000215704	CELA2B	0.058055876	0.544855652	-3.230360059
ENSG00000276334	AL133243.1	0.249928	2.355128	-3.236221028
ENSG00000158517	NCF1	0.00182317	0.017185902	-3.236704888
ENSG00000206602	SNORD58A	0.470278	4.433524	-3.236868137
ENSG00000270894	AC015849.13	0.060446	0.570772	-3.239195785
ENSG00000244699	RP11-803B1.2	0.045977	0.435722	-3.24442372
ENSG00000119946	CNNM1	0.007936351	0.075446906	-3.24891399
ENSG00000237207	RBM17P3	0.035582	0.339251	-3.253133559
ENSG00000221879	MRPS21P3	0.09713	0.927485	-3.255335083
ENSG00000236714	AC005592.1	0.420717	4.021661	-3.256869457
ENSG00000274678	RP11-2C24.7	0.093046	0.890466	-3.258544491
ENSG00000234358	AC003080.4	0.007373	0.070561	-3.258547345
ENSG00000229992	HMGB3P9	0.0291	0.278493	-3.258550007
ENSG00000280379	RP11-573M3.2	0.065842	0.630122	-3.258551116
ENSG00000120054	CPN1	0.026971541	0.258126696	-3.258569526
ENSG00000142748	FCN3	0.023487042	0.225179769	-3.261140284
ENSG00000102287	GABRE	0.056455776	0.542582488	-3.264649397
ENSG00000201586	RNU6-593P	0.454431	4.3683	-3.264938779
ENSG00000160886	LY6K	0.011564658	0.11165409	-3.271241565
ENSG00000257550	RP11-793H13.3	0.004875	0.047123	-3.272957265
ENSG00000273189	CTD-3148I10.15	0.022049	0.213996	-3.278798699
ENSG00000239602	RP11-449H3.1	0.158555	1.553837	-3.292779892
ENSG00000262905	RP5-1029F21.2	0.085973	0.849421	-3.304524222
ENSG00000172901	LVRN	0.246630045	2.437247741	-3.304832438
ENSG00000234902	AC007879.2	0.046480334	0.459654086	-3.305856216

ENSG00000236051	MYCBP2-AS1	0.030098281	0.298076803	-3.307931113
ENSG00000170848	PSG6	0.001414357	0.014039128	-3.311235103
ENSG00000240871	KRTAP4-7	0.035607897	0.354355979	-3.31493027
ENSG00000222317	RNA5SP118	0.326856	3.257162	-3.316888391
ENSG00000264085	MIR5004	0.009529	0.095018	-3.317804114
ENSG00000279981	RP11-196B3.2	0.020883	0.208828	-3.321914278
ENSG00000069696	DRD4	0.133114769	1.332648253	-3.323553485
ENSG00000278963	RP11-294J22.7	0.309021	3.138035	-3.344084656
ENSG00000255104	TBC1D26	0.055585004	0.564474987	-3.344142031
ENSG00000241913	RP5-1073F15.1	0.035927	0.369293	-3.36162554
ENSG00000221493	MIR320C1	0.196645	2.02952	-3.367473148
ENSG00000267026	RP11-92C4.3	0.101062009	1.045326168	-3.370640497
ENSG00000186197	EDARADD	0.013659839	0.141453771	-3.372318257
ENSG00000273658	uc_338	0.184569	1.917718	-3.377158423
ENSG00000235833	AC159540.14	0.09902	1.029062	-3.377466146
ENSG00000279827	RP11-886P16.10	0.00505	0.052646	-3.381968628
ENSG00000229487	ALG13-AS1	0.156604	1.638815	-3.387460035
ENSG00000256011	RP11-392P7.7	0.027586	0.289031	-3.389216053
ENSG00000270248	CTC-471J1.10	0.062221	0.656327	-3.398941297
ENSG00000145777	TSLP	0.011886084	0.125380144	-3.398963554
ENSG00000187260	WDR86	0.025171443	0.266320038	-3.403301174
ENSG00000185303	SFTPA2	0.018955028	0.200760156	-3.404820485
ENSG00000154678	PDE1C	0.003682607	0.039158541	-3.410527784
ENSG00000226543	MYL6P1	0.112353	1.194714	-3.410554744
ENSG00000182836	PLCXD3	0.006582929	0.070004657	-3.410649351
ENSG00000254397	CTD-2371O3.2	0.348956	3.717099	-3.41306007
ENSG00000277326	MIR6805	0.056339	0.600563	-3.414109733
ENSG00000255471	RP11-736K20.5	0.083573675	0.899825465	-3.428524715
ENSG00000232043	RP4-530I15.9	0.211117	2.278558	-3.432006434
ENSG00000279762	RP11-227G15.12	0.036891848	0.399222366	-3.435818572
ENSG00000185862	EVI2B	0.024563967	0.268619484	-3.450948496
ENSG00000225873	LINC00694	0.117549504	1.28631326	-3.451901677
ENSG00000202441	RNY4P10	1.060338	11.69229	-3.462961392
ENSG00000235779	AC079779.5	0.007376261	0.081703867	-3.469442839
ENSG00000270823	RP11-2E11.6	0.019323	0.2142	-3.470567477
ENSG00000207195	Y_RNA	1.366402	15.163502	-3.472149085
ENSG00000280328	RP11-972P1.7	0.209999	2.330712	-3.472316382
ENSG00000279021	RP11-391L3.4	0.095693	1.065638	-3.477160229
ENSG00000281691	RBM5-AS1	0.108799	1.214659	-3.480814151
ENSG00000212443	SNORA53	0.174764	1.951817	-3.481337857
ENSG00000229677	RP11-383F6.1	0.035101	0.392889	-3.484537739
ENSG00000235602	POU5F1P3	0.120395	1.347993	-3.484965621
ENSG00000201616	RNU1-91P	0.312246	3.512855	-3.491889032
ENSG00000277168	Metazoa_SRP	0.017578	0.198244	-3.495434365
ENSG00000163216	SPRR2D	0.016049953	0.18125547	-3.497383585
ENSG00000280198	RP11-384M20.1	0.079042	0.892767	-3.497592344
ENSG00000225532	XX-C2158C6.3	0.011594	0.132163	-3.510868044
ENSG00000007062	PROM1	0.429954643	4.905096424	-3.512025119

ENSG00000256176	RP11-627K11.3	0.02577	0.29458	-3.514895042
ENSG00000207690	MIR630	0.005216	0.059635	-3.515143528
ENSG00000204025	TRPC5OS	0.017282139	0.199207243	-3.526916445
ENSG00000124469	CEACAM8	0.005968319	0.069098505	-3.533257872
ENSG00000201644	Y_RNA	0.000542	0.006281	-3.534629513
ENSG00000223549	MTND5P28	0.03193	0.373479	-3.548042667
ENSG00000115474	KCNJ13	0.034193047	0.399949446	-3.548042771
ENSG00000179165	PXT1	0.024144	0.282413	-3.548069884
ENSG00000105085	MED26	0.145272889	1.707403	-3.554966219
ENSG00000253406	AC012613.2	0.022455	0.272152	-3.599304011
ENSG00000253307	RP11-10J21.4	0.035896	0.437417	-3.607114297
ENSG00000227262	HCG4B	0.10169	1.248978	-3.618498346
ENSG00000160870	CYP3A7	0.003603706	0.044525659	-3.62708373
ENSG00000265611	MIR5010	0.034224	0.426271	-3.638690617
ENSG00000258667	HIF1A-AS2	0.051565	0.642714	-3.639712832
ENSG00000206834	SNORA1	0.314117	3.931425	-3.645678403
ENSG00000200350	RNU6-1285P	0.155112	1.948339	-3.650862512
ENSG00000274596	MIR6872	0.109454	1.377897	-3.654071464
ENSG00000232750	RP11-177A2.5	0.056223	0.711119	-3.660858662
ENSG00000262966	RP11-85B7.2	0.008973	0.113546	-3.661542662
ENSG00000273999	RBM17P2	0.030188	0.383145	-3.665843394
ENSG00000276096	Metazoa_SRP	0.057315	0.727701	-3.66636113
ENSG00000263338	RP11-235E17.3	0.034537	0.441427	-3.675960199
ENSG00000260179	RP5-902P8.12	0.065335	0.845503	-3.693881917
ENSG00000271454	RP11-290L7.5	0.020375	0.263742	-3.694255369
ENSG00000167476	JSRP1	0.030415872	0.396660032	-3.705006753
ENSG00000222365	SNORD12B	1.372908	17.952513	-3.70887895
ENSG00000281991	TMEM265	0.842723	11.081964	-3.717011275
ENSG00000251175	RP11-45L9.1	0.017253109	0.228013938	-3.724193758
ENSG00000270136	MINOS1-NBL1	0.007380987	0.097616763	-3.725243309
ENSG00000265573	AC004943.1	0.006826	0.090514	-3.729028635
ENSG00000257507	RP11-956E11.1	0.019252	0.255889	-3.732437897
ENSG00000278638	CTC-268N12.2	0.022683	0.301508	-3.732512917
ENSG00000207733	MIR637	0.341133	4.53654	-3.733186151
ENSG00000196136	SERPINA3	0.011536506	0.154756616	-3.745722835
ENSG00000274322	RP11-314N13.10	0.064881687	0.870832665	-3.746512281
ENSG00000237186	RP11-229A12.2	0.023419	0.315052	-3.749838589
ENSG00000212643	ZRSR1	0.024338	0.332144	-3.77052633
ENSG00000234630	LL22NC03-2H8.4	0.048565	0.662811	-3.770608679
ENSG00000225706	PTPRD-AS1	0.001573698	0.021700051	-3.785467406
ENSG00000169075	RP3-509L4.3	0.017229	0.238175	-3.789111116
ENSG00000259771	RP11-429D19.1	0.094242	1.303361	-3.789722765
ENSG00000280329	AC026449.1	0.12926	1.792262	-3.79343375
ENSG00000259617	RP11-540O11.6	0.101792	1.412282	-3.794332103
ENSG00000240068	RPL21P42	0.020183	0.280401	-3.796278957
ENSG00000176761	ZNF285B	0.014402	0.200544	-3.799577726
ENSG00000225442	MPRIP-AS1	0.048106	0.672088	-3.804361395
ENSG00000224337	FAM8A3P	0.020101	0.280969	-3.805071783

ENSG00000259396	RP11-16O9.2	0.020774	0.292904	-3.817576959
ENSG00000207031	SNORD59A	1.971219	27.800123	-3.817931294
ENSG00000122733	PHF24	0.026266601	0.371665082	-3.822701702
ENSG00000173867	RP11-97O12.7	0.08633761	1.22174986	-3.822815975
ENSG00000242265	PEG10	0.278110691	3.939496439	-3.824280121
ENSG00000273493	RP11-80H18.4	0.079319	1.124583	-3.825579844
ENSG00000234459	AC002064.4	0.088056	1.250971	-3.828483225
ENSG00000277402	MIR6891	0.016161	0.230223	-3.832443594
ENSG00000187472	RP11-67P15.1	0.068134	0.979241	-3.845217152
ENSG00000233602	ERI3-IT1	0.082223	1.201257	-3.868859016
ENSG00000199053	MIR324	1.208785	17.698915	-3.872031353
ENSG00000267160	RP11-1072C15.4	0.039624	0.583971	-3.881450295
ENSG00000223849	RP11-80I15.1	0.020893	0.311037	-3.895994642
ENSG00000260643	RP11-303E16.8	0.426	6.34446	-3.896572041
ENSG00000208028	MIR616	0.472233	7.052407	-3.900544969
ENSG00000259645	RP11-253M7.6	0.073337	1.110991	-3.921162069
ENSG00000256361	RP11-613F22.6	0.117543	1.797352	-3.934612449
ENSG00000250906	RP11-632F7.3	0.236044	3.631591	-3.943474015
ENSG00000275820	MIR6778	0.047912	0.741378	-3.951750362
ENSG00000137440	FGFBP1	0.255418	3.96645	-3.956916259
ENSG00000278493	CTD-2382E5.6	0.394421	6.13013	-3.958109397
ENSG00000269032	AC016629.7	0.015946	0.250206	-3.971849907
ENSG00000249251	PGAM1P8	0.373057	5.89636	-3.982356626
ENSG00000269540	CTD-2126E3.5	0.037673	0.600894	-3.995507688
ENSG00000268533	AC004076.7	0.097689	1.564665	-4.001513874
ENSG00000224463	AC079354.6	0.04627	0.744032	-4.007215667
ENSG00000238300	SNORD121B	0.087969	1.446447	-4.039374439
ENSG00000267082	CTC-510F12.2	0.227237	3.756533	-4.047132109
ENSG00000227382	EIF4A2P2	0.022357	0.36974	-4.047712612
ENSG00000171951	SCG2	1.626282139	27.02444083	-4.054615294
ENSG00000261114	RP11-325K4.2	0.582503	9.679755	-4.054633147
ENSG00000213742	ZNF337-AS1	0.116481297	1.93874236	-4.056950867
ENSG00000274976	RP11-8P13.5	0.305855	5.114771	-4.063749883
ENSG00000240625	RN7SL403P	0.010489	0.177444	-4.080414747
ENSG00000237737	DCTN1-AS1	0.002886457	0.049107465	-4.088570489
ENSG00000214773	RP11-717D12.1	0.035642	0.606392	-4.088600521
ENSG00000213096	ZNF254	0.028729274	0.493292136	-4.101848854
ENSG00000278986	RP11-723J4.3	0.002353	0.040606	-4.109119692
ENSG00000265068	AL591377.1	0.014203	0.246689	-4.118425789
ENSG00000260007	RP11-315D16.2	0.496629	8.717173	-4.133619928
ENSG00000234136	AC055764.1	0.034433	0.609784	-4.146434509
ENSG00000277622	MIR6734	0.169813	3.01544	-4.150349716
ENSG00000200312	RN7SKP255	0.040652	0.73227	-4.170977455
ENSG00000243509	TNFRSF6B	1.614915	29.501162	-4.191241644
ENSG00000229999	RP5-1042K10.10	0.005699474	0.104241053	-4.192951047
ENSG00000250033	SLC7A11-AS1	0.098663059	1.811961427	-4.198898421
ENSG00000160888	IER2	2.080986774	38.5382299	-4.210950608
ENSG00000275652	MIR6796	0.065048	1.24578	-4.259400806

ENSG00000228686	RP11-492I21.1	0.080916	1.553367	-4.262829909
ENSG00000275651	MIR6851	0.136975	2.640217	-4.268672001
ENSG00000200623	SNORD18A	0.014691	0.284382	-4.274825645
ENSG00000168060	NAALADL1	0.012585322	0.247579169	-4.298075876
ENSG00000239300	RP11-400L8.2	0.116097	2.293981255	-4.304451004
ENSG00000201785	SNORD117	0.445694	8.850828	-4.311686982
ENSG00000236047	AC073410.1	0.082955	1.652101	-4.315829137
ENSG00000249180	CTC-506B8.1	0.090082	1.806786	-4.326042971
ENSG00000265236	SNORD84	0.112462	2.29333	-4.349934453
ENSG00000013725	CD6	0.004271221	0.087361547	-4.354278108
ENSG00000226625	RBM17P1	0.014257	0.293379	-4.363023264
ENSG00000133742	CA1	0.000625535	0.013037849	-4.381472243
ENSG00000203546	RP11-176H8.1	0.004520791	0.095425049	-4.399720731
ENSG00000221273	MIR1237	0.019959	0.424475	-4.410568242
ENSG00000279816	AC010287.1	0.185232	4.007992	-4.435474369
ENSG00000175197	DDIT3	8.00214119	174.7382179	-4.448665288
ENSG00000225511	LINC00475	0.002374675	0.052465235	-4.465559803
ENSG00000256588	RP11-613F22.8	0.077556	1.715781	-4.467483213
ENSG00000278067	MIR6742	0.00821	0.182885	-4.47741072
ENSG00000221866	PLXNA4	0.009326517	0.207794563	-4.477675691
ENSG00000276372	MIR6785	0.014152	0.319268	-4.495690102
ENSG00000271745	AC093668.2	0.110472	2.52982	-4.51728208
ENSG00000253754	RP11-35G22.1	0.113862	2.611595	-4.519572933
ENSG00000128564	VGF	1.679913736	39.10688578	-4.540963596
ENSG00000121898	CPXM2	0.010474755	0.25044692	-4.579516481
ENSG00000206914	Y_RNA	0.119756	2.865205	-4.58046852
ENSG00000226849	RP4-635E18.7	0.018977	0.458678	-4.595157866
ENSG00000220069	RPL7P27	0.007068	0.177896	-4.653588222
ENSG00000281910	SNORA50A	0.374237	9.646192	-4.687935419
ENSG00000185567	AHNAK2	0.702167374	18.19434916	-4.695531671
ENSG00000254577	RP11-484D2.4	0.25031	6.533957	-4.706169127
ENSG00000271639	RP11-289A15.1	0.014438	0.381042	-4.722007212
ENSG00000257298	RP3-405J10.3	0.053996	1.42599	-4.722967517
ENSG00000234787	LINC00458	0.000833167	0.022144198	-4.732179346
ENSG00000253532	CTD-2340D6.1	0.003421	0.090959	-4.732726384
ENSG00000279851	CH507-216K13.2	0.00166	0.04445	-4.742928272
ENSG00000243265	RPL23AP55	0.01319	0.359207	-4.767298995
ENSG00000196507	TCEAL3	0.003974474	0.108945585	-4.776699918
ENSG00000247473	CARS-AS1	0.053174751	1.457709424	-4.776817992
ENSG00000235663	SAPCD1-AS1	0.013248	0.369294	-4.80092334
ENSG00000267387	CTD-2240E14.4	0.027225	0.760147	-4.803274485
ENSG00000200924	RNU6-1048P	0.133186	3.763147	-4.820425302
ENSG00000260689	HNRNPA3P11	0.008174	0.247231	-4.918673599
ENSG00000275884	MIR6808	0.004313	0.130899	-4.92361855
ENSG00000261714	CTD-2562G15.2	0.010437	0.330268	-4.983858202
ENSG00000264831	MIR4260	0.034726	1.102353	-4.988426233
ENSG00000072858	SIDT1	0.006314705	0.20437397	-5.016352376
ENSG00000173406	DAB1	0.000219859	0.00717069	-5.027461217

ENSG00000281428	AL136303.2	0.012725	0.456642	-5.165325997
ENSG00000278799	MIR6823	0.008344	0.301656	-5.17602131
ENSG00000261131	RP11-93O14.2	0.015855	0.588285	-5.213505469
ENSG00000259402	RP11-30K9.4	0.029028	1.111473	-5.258883917
ENSG00000221008	AC136698.1	0.002989	0.11558	-5.273085068
ENSG00000237232	ZNF295-AS1	0.009708584	0.384832904	-5.308827453
ENSG00000272084	RP5-1126H10.2	0.001984	0.079542	-5.325232907
ENSG00000266618	MIR4742	0.047902	1.933977	-5.335340935
ENSG00000276727	RP11-123K3.9	0.072605	2.994	-5.365861507
ENSG00000263715	CRHR1	0.018368015	0.777810457	-5.404150987
ENSG00000278027	MIR6883	0.134418	5.734747	-5.414931592
ENSG00000232934	RP11-324O2.3	0.016423216	0.720419334	-5.45502837
ENSG00000243649	CFB	0.010185445	0.478579314	-5.55417714
ENSG00000234737	KRT18P15	0.136018	6.478124	-5.573706595
ENSG00000255275	RP13-279N23.2	0.025619	1.319883	-5.687052075
ENSG00000225218	AP001628.6	0.02143	1.106355	-5.690038722
ENSG00000276908	MIR7106	0.014428	0.748742	-5.69752545
ENSG00000236516	KLF2P4	0.003324	0.177604	-5.739599882
ENSG00000242150	RP11-134G8.6	0.182926	9.898018	-5.757807615
ENSG00000200959	SNORA74A	0.01702	0.958184	-5.814999781
ENSG00000188000	OR7D2	0.015471	0.921258	-5.895966886
ENSG00000228224	NACAP1	0.145556878	8.828434877	-5.922502774
ENSG00000258838	ERCC6-PGBD3	0.014467652	0.99991361	-6.110900741
ENSG00000228886	RP11-290D2.3	0.016418	1.214612	-6.209073325
ENSG00000277892	MIR6746	0.016158	1.196109	-6.209956421
ENSG00000206693	SNORA56	0.019008	1.433154	-6.236443092
ENSG00000278598	MIR6775	0.014753	1.278499	-6.437298868
ENSG00000238923	RNU7-1	0.051877	4.512981	-6.442841842
ENSG00000275682	AC137723.1	0.01131	1.082411	-6.580505666
ENSG00000274111	MIR6777	0.007712	0.754408	-6.612096113
ENSG00000261796	ISY1-RAB43	0.030106	2.963476	-6.621095525
ENSG00000263155	MYZAP	0.105448474	10.43359821	-6.62855476
ENSG00000275273	MIR6780A	0.01497	1.559944	-6.703276207
ENSG00000277719	MIR7705	0.314532	34.045635	-6.758119229
ENSG00000204422	XXbac-BPG32J3.20	0.009387	1.106322	-6.880891475
ENSG00000274603	Y_RNA	0.004669	0.625614	-7.066015399
ENSG00000238449	AC019131.1	1.00E-06	0.000139	-7.118941073
ENSG00000260798	RP11-354M1.2	0.001585	0.244595	-7.269768262
ENSG00000266304	RP11-484N16.1	0.034461	5.598093	-7.343827077
ENSG00000249087	ZNF436-AS1	0.003450565	0.574187563	-7.378545675
ENSG00000250767	RP11-1084J3.3	1.00E-06	0.000199	-7.636624621
ENSG00000273705	MIR6843	1.00E-06	0.000223	-7.8008999
ENSG00000274629	RP11-92F20.1	0.000726181	0.172295989	-7.890344016
ENSG00000280459	Metazoa_SRP	0.001535	0.364365	-7.891001918
ENSG00000243781	RP11-193H5.2	0.228657	67.896477	-8.214007822
ENSG00000264772	RP11-186B7.4	0.025912	8.528206	-8.362478104
ENSG00000272884	RP11-104H15.10	0.002749	0.955231	-8.44079894
ENSG00000273211	RP13-131K19.7	1.00E-06	0.000386	-8.592457037

ENSG00000229183	PGA4	1.00E-06	0.000560783	-9.131299363
ENSG00000241163	LINC00877	1.00E-06	0.000577438	-9.173522977
ENSG00000274083	MIR6837	0.000146	0.090632	-9.277908342
ENSG00000036565	SLC18A1	1.00E-06	0.000645902	-9.335170438
ENSG00000265555	RP11-638L3.3	1.00E-06	0.00084	-9.714245518
ENSG00000267552	CTD-2528L19.4	1.00E-06	0.000985152	-9.944201929
ENSG00000270726	AJ271736.10	1.00E-06	0.001041	-10.02375435
ENSG00000145863	GABRA6	1.00E-06	0.001158157	-10.1776155
ENSG00000203709	C1orf132	1.00E-06	0.0013146	-10.36040768
ENSG00000204960	BLACE	1.00E-06	0.001542	-10.59058705
ENSG00000239572	RP11-451B8.1	1.00E-06	0.001728	-10.7548875
ENSG00000162997	PRORSD1P	0.000154416	0.293684804	-10.89323432
ENSG00000169554	ZEB2	1.00E-06	0.002464422	-11.26703341
ENSG00000243064	ABCC13	1.00E-06	0.002523848	-11.30140924
ENSG00000225657	RP5-843L14.1	1.00E-06	0.002535506	-11.30805799
ENSG00000118997	DNAH7	1.00E-06	0.00253762	-11.3092602
ENSG00000221593	AC026150.2	1.00E-06	0.002717	-11.40779885
ENSG00000221405	AC123768.1	1.00E-06	0.002717	-11.40779885
ENSG00000165323	FAT3	1.00E-06	0.002806135	-11.45436889
ENSG00000183979	NPB	0.000201346	0.576016081	-11.4822189
ENSG00000179869	ABCA13	1.00E-06	0.002903991	-11.50382128
ENSG00000274044	AC011718.2	1.00E-06	0.002936857	-11.52005738
ENSG00000278920	RP3-412A9.17	1.00E-06	0.002994	-11.54785851
ENSG00000131042	LILRB2	1.00E-06	0.002996983	-11.54929516
ENSG00000250305	KIAA1456	1.00E-06	0.003025513	-11.56296401
ENSG00000072133	RPS6KA6	1.00E-06	0.003182278	-11.63584418
ENSG00000254300	LINC01111	1.00E-06	0.003255	-11.66844183
ENSG00000274728	AC091565.1	1.00E-06	0.00326	-11.67065625
ENSG00000279766	RP11-642A1.2	0.000314	1.066221	-11.72945432
ENSG00000147481	SNTG1	1.00E-06	0.003396111	-11.72966773
ENSG00000243130	PSG11	1.00E-06	0.003441724	-11.74891576
ENSG00000223614	ZNF735	1.00E-06	0.003447	-11.75112558
ENSG00000197123	ZNF679	1.00E-06	0.003454	-11.75405237
ENSG00000242290	RP11-197K3.1	1.00E-06	0.003500971	-11.77353957
ENSG00000179826	MRGPRX3	1.00E-06	0.003522767	-11.78249349
ENSG00000213384	EIF4E2P1	1.00E-06	0.003537	-11.7883105
ENSG00000242173	ARHGDIG	1.00E-06	0.003564637	-11.79953958
ENSG00000177910	SPATA31C2	1.00E-06	0.003583	-11.80695233
ENSG00000281450	PANDAR	1.00E-06	0.003594	-11.81137469
ENSG00000219492	RP11-1396O13.13	1.00E-06	0.003604	-11.8153833
ENSG00000268027	AC006129.2	1.00E-06	0.00375806	-11.87577255
ENSG00000197503	LINC00477	1.00E-06	0.003798	-11.89102419
ENSG00000151789	ZNF385D	1.00E-06	0.003973288	-11.9561175
ENSG00000079102	RUNX1T1	1.00E-06	0.003998507	-11.96524588
ENSG00000116147	TNR	1.00E-06	0.004175453	-12.02771701
ENSG00000196090	PTPRT	1.00E-06	0.004181373	-12.02976117
ENSG00000079841	RIMS1	1.00E-06	0.004232051	-12.04714124
ENSG00000221250	AC120045.1	1.00E-06	0.004347	-12.08580438

ENSG00000249487	LINC01586	1.00E-06	0.004447927	-12.11891738
ENSG00000248779	RP11-53O19.2	1.00E-06	0.004579	-12.16081685
ENSG00000171060	C8orf74	1.00E-06	0.004656757	-12.18510995
ENSG00000154080	CHST9	1.00E-06	0.004697723	-12.19774592
ENSG00000257097	CLIP1-AS1	1.00E-06	0.004787416	-12.22503137
ENSG00000164692	COL1A2	1.00E-06	0.004849486	-12.24361604
ENSG00000249647	C5orf66-AS2	1.00E-06	0.004857664	-12.24604693
ENSG00000264423	RN7SL718P	1.00E-06	0.005001	-12.28800089
ENSG00000251676	RP11-614F17.2	1.00E-06	0.005033922	-12.29746725
ENSG00000135917	SLC19A3	1.00E-06	0.005077946	-12.31002922
ENSG00000101204	CHRNA4	1.00E-06	0.005081879	-12.31114635
ENSG00000205056	RP11-693J15.5	1.00E-06	0.005093	-12.3143
ENSG00000128438	TBC1D27	1.00E-06	0.005154083	-12.33149996
ENSG00000166573	GALR1	1.00E-06	0.005254595	-12.35936381
ENSG00000198643	FAM3D	1.00E-06	0.005280021	-12.36632805
ENSG00000244222	OR7E121P	1.00E-06	0.005295	-12.37041497
ENSG00000105428	ZNRF4	1.00E-06	0.005314	-12.37558251
ENSG00000231667	OR7E111P	1.00E-06	0.005337	-12.3818133
ENSG00000099260	PALMD	1.00E-06	0.005383259	-12.39426415
ENSG00000198879	SFMBT2	1.00E-06	0.005437745	-12.40879289
ENSG00000270432	OR7E108P	1.00E-06	0.005461	-12.41494944
ENSG00000259783	RP11-1006G14.2	1.00E-06	0.005467357	-12.41662784
ENSG00000130368	MAS1	1.00E-06	0.0055	-12.4252159
ENSG00000101825	MXRA5	1.00E-06	0.005527	-12.4322809
ENSG00000183206	POTEC	1.00E-06	0.005551533	-12.4386705
ENSG00000254817	OR7E160P	1.00E-06	0.005597	-12.45043803
ENSG00000234458	OR7E130P	1.00E-06	0.005614	-12.45481335
ENSG00000232420	IL9RP2	1.00E-06	0.005775	-12.49560523
ENSG00000162456	KNCN	1.00E-06	0.005778063	-12.49637022
ENSG00000177324	BEND2	1.00E-06	0.005836092	-12.51078693
ENSG00000050628	PTGER3	1.00E-06	0.00584187	-12.51221456
ENSG00000166351	POTED	1.00E-06	0.005865958	-12.51815091
ENSG00000250425	OR7E35P	1.00E-06	0.00602	-12.55554777
ENSG00000261727	RP11-1166P10.6	1.00E-06	0.006055855	-12.56411484
ENSG00000117707	PROX1	1.00E-06	0.006062249	-12.56563747
ENSG00000222036	POTEM	1.00E-06	0.006064756	-12.56623393
ENSG00000261259	RP11-19N8.2	1.00E-06	0.006168	-12.59058705
ENSG00000184716	SERINC4	1.00E-06	0.006180652	-12.59354324
ENSG00000196167	COLCA1	1.00E-06	0.006197061	-12.59736846
ENSG00000205847	OR7E91P	1.00E-06	0.00625	-12.60964047
ENSG00000267120	AD000671.6	1.00E-06	0.006302	-12.62159404
ENSG00000225930	DKFZP434L187	1.00E-06	0.006339606	-12.63017757
ENSG00000082293	COL19A1	1.00E-06	0.006358964	-12.63457593
ENSG00000144868	TMEM108	1.00E-06	0.006424305	-12.6493246
ENSG00000273167	RP11-307N16.6	1.00E-06	0.00651	-12.66844183
ENSG00000221358	AC026150.1	1.00E-06	0.00652	-12.67065625
ENSG00000172554	SNTG2	1.00E-06	0.006539349	-12.67493127
ENSG00000280061	CTD-2001J20.1	1.00E-06	0.006595	-12.68715694

ENSG00000213726	RPS2P52	1.00E-06	0.006601	-12.68846888
ENSG00000183747	ACSM2A	1.00E-06	0.006605788	-12.68951503
ENSG00000186020	ZNF529	1.00E-06	0.006622324	-12.69312188
ENSG00000266988	RP11-749H17.1	1.00E-06	0.006643132	-12.69764777
ENSG00000274910	MIR6831	1.00E-06	0.006682	-12.70606427
ENSG00000231953	RP4-706G24.1	1.00E-06	0.006938	-12.76030413
ENSG00000187048	CYP4A11	1.00E-06	0.006955874	-12.76401609
ENSG00000277870	FAM230A	1.00E-06	0.006967076	-12.76633763
ENSG00000075884	ARHGAP15	1.00E-06	0.006989347	-12.77094191
ENSG00000130844	ZNF331	1.00E-06	0.007003213	-12.7738012
ENSG00000241956	CTC-340A15.2	1.00E-06	0.007050975	-12.78360709
ENSG00000278100	Metazoa_SRP	1.00E-06	0.007059	-12.78524811
ENSG00000213561	RP11-386I14.2	1.00E-06	0.007065	-12.78647385
ENSG00000139973	SYT16	1.00E-06	0.007078927	-12.78931507
ENSG00000166704	ZNF606	1.00E-06	0.007088464	-12.79125726
ENSG00000148600	CDHR1	1.00E-06	0.007091044	-12.79178242
ENSG00000226758	DISC1-IT1	1.00E-06	0.007103	-12.79421277
ENSG00000280418	AC007326.10	1.00E-06	0.007141	-12.8019104
ENSG00000228862	RP11-91I20.3	1.00E-06	0.007147769	-12.80327722
ENSG00000204965	PCDHA5	1.00E-06	0.007151258	-12.8039813
ENSG00000077943	ITGA8	1.00E-06	0.007189301	-12.81163585
ENSG00000154478	GPR26	1.00E-06	0.007202	-12.81418188
ENSG00000244244	RPS3AP42	1.00E-06	0.00721	-12.81578354
ENSG00000152254	G6PC2	1.00E-06	0.00727399	-12.82853131
ENSG00000273945	MIR6853	1.00E-06	0.007314	-12.83644491
ENSG00000258763	RP11-110A12.2	1.00E-06	0.007493142	-12.87135516
ENSG00000174898	CATSPERD	1.00E-06	0.007502986	-12.87324911
ENSG00000113889	KNG1	1.00E-06	0.007524854	-12.87744793
ENSG00000072840	EVC	1.00E-06	0.00757181	-12.88642244
ENSG00000196616	ADH1B	1.00E-06	0.007605645	-12.8928548
ENSG00000165970	SLC6A5	1.00E-06	0.007607782	-12.89326016
ENSG00000162687	KCNT2	1.00E-06	0.007622214	-12.89599433
ENSG00000234680	VN2R3P	1.00E-06	0.007666	-12.90425828
ENSG00000276584	MIR6737	1.00E-06	0.007732	-12.91662592
ENSG00000248441	LINC01197	1.00E-06	0.007740192	-12.91815368
ENSG00000164794	KCNV1	1.00E-06	0.007754	-12.92072502
ENSG00000095917	TPSD1	1.00E-06	0.007771904	-12.92405237
ENSG00000234570	ZFRP1	1.00E-06	0.007836	-12.93590168
ENSG00000276270	MIR6799	1.00E-06	0.007843	-12.93718989
ENSG00000177138	FAM9B	1.00E-06	0.007908909	-12.94926297
ENSG00000102383	ZDHHC15	1.00E-06	0.007935455	-12.95409715
ENSG00000237352	LINC01358	1.00E-06	0.008041423	-12.97323506
ENSG00000135824	RGS8	1.00E-06	0.008095785	-12.9829553
ENSG00000184261	KCNK12	1.00E-06	0.008096497	-12.98308218
ENSG00000091128	LAMB4	1.00E-06	0.008161688	-12.99465177
ENSG00000229656	RP11-462L8.1	1.00E-06	0.008166142	-12.99543903
ENSG00000235142	RP1-60O19.1	1.00E-06	0.008187489	-12.99920539
ENSG00000242668	RN7SL317P	1.00E-06	0.008225	-13.00579996

ENSG00000226785	AC073218.1	1.00E-06	0.008273	-13.01419487
ENSG00000138675	FGF5	1.00E-06	0.008301274	-13.01911707
ENSG00000125675	GRIA3	1.00E-06	0.008347486	-13.02712605
ENSG00000126952	NXF5	1.00E-06	0.008359795	-13.02925188
ENSG00000183098	GPC6	1.00E-06	0.008367706	-13.03061646
ENSG00000277057	MIR6779	1.00E-06	0.008456	-13.04575966
ENSG00000104321	TRPA1	1.00E-06	0.008486863	-13.05101569
ENSG00000153822	KCNJ16	1.00E-06	0.008535968	-13.05933913
ENSG00000157873	TNFRSF14	1.00E-06	0.008702258	-13.08717414
ENSG00000154016	GRAP	1.00E-06	0.008708966	-13.08828571
ENSG00000151577	DRD3	1.00E-06	0.008758032	-13.09639107
ENSG00000130201	EXOC3L2	1.00E-06	0.008784339	-13.10071804
ENSG00000136750	GAD2	1.00E-06	0.008807976	-13.10459481
ENSG00000265954	MIR4749	1.00E-06	0.008873	-13.11520625
ENSG00000214835	RPL23AP6	1.00E-06	0.008944	-13.12670447
ENSG00000162692	VCAM1	1.00E-06	0.008947197	-13.12722008
ENSG00000229240	LINC00710	1.00E-06	0.008956392	-13.12870203
ENSG00000179776	CDH5	1.00E-06	0.009062344	-13.14566858
ENSG00000235716	KRT18P46	1.00E-06	0.009098	-13.15133372
ENSG00000139144	PIK3C2G	1.00E-06	0.009134433	-13.15709945
ENSG00000246223	LINC01550	1.00E-06	0.009251446	-13.17546318
ENSG00000120907	ADRA1A	1.00E-06	0.009254216	-13.17589508
ENSG00000072832	CRMP1	1.00E-06	0.00929398	-13.18208089
ENSG00000102290	PCDH11X	1.00E-06	0.009295343	-13.18229238
ENSG00000244380	RP11-24C3.2	1.00E-06	0.009298815	-13.18283123
ENSG00000204963	PCDHA7	1.00E-06	0.009350602	-13.19084352
ENSG00000137077	CCL21	1.00E-06	0.009372	-13.19414124
ENSG00000175928	LRRN1	1.00E-06	0.009392441	-13.19728445
ENSG00000022355	GABRA1	1.00E-06	0.00953464	-13.21896268
ENSG00000160183	TMPRSS3	1.00E-06	0.009561512	-13.22302304
ENSG00000078053	AMPH	1.00E-06	0.009602664	-13.22921892
ENSG00000258654	RP11-509A17.3	1.00E-06	0.00960926	-13.23020962
ENSG00000253311	AC011343.1	1.00E-06	0.009652	-13.2366122
ENSG00000227195	MIR663AHG	1.00E-06	0.00968645	-13.2417523
ENSG00000163145	C1QTNF7	1.00E-06	0.009693667	-13.24282684
ENSG00000166947	EPB42	1.00E-06	0.009709497	-13.24518089
ENSG00000157404	KIT	1.00E-06	0.009728761	-13.24804033
ENSG00000102230	PCYT1B	1.00E-06	0.009742716	-13.25010826
ENSG00000269891	ARHGAP19-SLIT1	1.00E-06	0.009799336	-13.25846825
ENSG00000110786	PTPN5	1.00E-06	0.009845452	-13.26524176
ENSG00000181092	ADIPOQ	1.00E-06	0.009855	-13.26664016
ENSG00000259006	RP11-566K11.4	1.00E-06	0.00989	-13.27175481
ENSG00000249003	CLUHP4	1.00E-06	0.00994	-13.27903014
ENSG00000269155	RP3-431P23.5	1.00E-06	0.009953	-13.28091573
ENSG00000164197	RNF180	1.00E-06	0.009960065	-13.28193941
ENSG00000244681	MTHFD2P1	1.00E-06	0.00998901	-13.28612598
ENSG00000162383	SLC1A7	1.00E-06	0.010009676	-13.2891076
ENSG00000213203	GIMAP1	1.00E-06	0.010162534	-13.31097257

ENSG00000175426	PCSK1		1.00E-06	0.010197876	-13.31598103
ENSG00000198844	ARHGEF15		1.00E-06	0.010223206	-13.31956014
ENSG00000184304	PRKD1		1.00E-06	0.010225113	-13.31982914
ENSG00000260249	RP11-401P9.5		1.00E-06	0.010259966	-13.32473831
ENSG00000171649	ZIK1		1.00E-06	0.010295424	-13.32971565
ENSG00000257501	RP11-1016B18.1		1.00E-06	0.010304696	-13.33101437
ENSG00000225594	AC098826.4		1.00E-06	0.010341	-13.33608808
ENSG00000228016	RAPGEF4-AS1		1.00E-06	0.010374113	-13.34070044
ENSG00000248724	NPHP3-AS1		1.00E-06	0.01037797	-13.34123672
ENSG00000178235	SLITRK1		1.00E-06	0.01043	-13.34845154
ENSG00000233766	AC098617.1		1.00E-06	0.010498676	-13.35791973
ENSG00000235881	AC114776.3		1.00E-06	0.01059	-13.37041497
ENSG00000282815	TEX13C		1.00E-06	0.010623	-13.37490363
ENSG00000132704	FCRL2		1.00E-06	0.010699647	-13.38527562
ENSG00000280309	RP11-60L3.3		1.00E-06	0.01077	-13.39473063
ENSG00000250312	ZNF718		1.00E-06	0.010776957	-13.39566229
ENSG00000196437	ZNF569		1.00E-06	0.010789662	-13.39736212
ENSG00000243766	HOTTIP		1.00E-06	0.010789892	-13.39739285
ENSG00000100368	CSF2RB		1.00E-06	0.010824065	-13.40195484
ENSG00000108018	SORCS1		1.00E-06	0.010862751	-13.40710185
ENSG00000274317	RP11-93G5.1		1.00E-06	0.010914	-13.41389233
ENSG00000235711	ANKRD34C		1.00E-06	0.010931	-13.41613777
ENSG00000204970	PCDHA1		1.00E-06	0.011027091	-13.42876458
ENSG00000205300	RP11-352D3.2		1.00E-06	0.011045	-13.4311058
ENSG00000278900	RP11-1250I15.2		1.00E-06	0.011063	-13.43345504
ENSG00000178562	CD28		1.00E-06	0.011078928	-13.43553069
ENSG00000225491	UBE2Q2P4Y		1.00E-06	0.011205	-13.45185503
ENSG00000223636	UBE2Q2P5Y		1.00E-06	0.011205	-13.45185503
ENSG00000076344	RGS11		1.00E-06	0.011280028	-13.46148307
ENSG00000104332	SFRP1		1.00E-06	0.011367279	-13.47259935
ENSG00000260802	LINC00890		1.00E-06	0.011374508	-13.47351655
ENSG00000251247	ZNF345		1.00E-06	0.011422687	-13.47961441
ENSG00000173077		1-Dec	1.00E-06	0.011497943	-13.48908812
ENSG00000137573	SULF1		1.00E-06	0.011510093	-13.49061181
ENSG00000206052	DOK6		1.00E-06	0.011531335	-13.4932719
ENSG00000278522	POTEB3		1.00E-06	0.011561721	-13.49706849
ENSG00000204362	RP11-380J14.1		1.00E-06	0.011574	-13.49859993
ENSG00000171812	COL8A2		1.00E-06	0.011589	-13.50046846
ENSG00000266433	TBC1D3P5		1.00E-06	0.01161667	-13.50390893
ENSG00000231507	LINC01353		1.00E-06	0.011638889	-13.50666572
ENSG00000230804	C2orf27AP3		1.00E-06	0.011684	-13.51224664
ENSG00000268951	RP11-149F8.3		1.00E-06	0.011704	-13.51471405
ENSG00000255350	MTCO1P15		1.00E-06	0.011822	-13.52918651
ENSG00000168405	CMAHP		1.00E-06	0.011842652	-13.53170458
ENSG00000241469	LINC00635		1.00E-06	0.011902656	-13.53899597
ENSG00000134160	TRPM1		1.00E-06	0.011908121	-13.5396582
ENSG00000229526	KRT16P4		1.00E-06	0.011947232	-13.54438876
ENSG00000115526	CHST10		1.00E-06	0.012061557	-13.55812853

ENSG00000182107	TMEM30B	1.00E-06	0.012105	-13.56331546
ENSG00000225823	RPL7P45	1.00E-06	0.01214	-13.5674808
ENSG00000184330	S100A7A	1.00E-06	0.012142875	-13.56782237
ENSG00000204688	OR2H1	1.00E-06	0.012163181	-13.57023299
ENSG00000244301	AOX3P	1.00E-06	0.01218086	-13.57232837
ENSG00000259828	RP11-63E9.1	1.00E-06	0.012189	-13.57329215
ENSG00000141052	MYOCD	1.00E-06	0.01219581	-13.57409791
ENSG00000198440	ZNF583	1.00E-06	0.012220461	-13.57701104
ENSG00000187398	LUZP2	1.00E-06	0.012229156	-13.57803728
ENSG00000090659	CD209	1.00E-06	0.012263763	-13.58211415
ENSG00000241684	ADAMTS9-AS2	1.00E-06	0.012272881	-13.58318636
ENSG00000249745	HMGB1P28	1.00E-06	0.012325	-13.58930003
ENSG00000095110	NXPE1	1.00E-06	0.012536454	-13.61384177
ENSG00000278998	RP11-1430O6.1	1.00E-06	0.012546	-13.61493985
ENSG00000260816	RP11-319G9.3	1.00E-06	0.012633	-13.62490966
ENSG00000155495	MAGEC1	1.00E-06	0.012675	-13.62969813
ENSG00000180708	OR10K2	1.00E-06	0.01268	-13.63026713
ENSG00000030304	MUSK	1.00E-06	0.012745948	-13.63775108
ENSG00000267719	HP09025	1.00E-06	0.012774	-13.64092274
ENSG00000240770	C21orf91-OT1	1.00E-06	0.012874389	-13.65221632
ENSG00000174279	EVX2	1.00E-06	0.012876	-13.65239686
ENSG00000270455	PABPC1P5	1.00E-06	0.012916	-13.65687173
ENSG00000120341	SEC16B	1.00E-06	0.012936272	-13.65913434
ENSG00000249526	CTB-35F21.1	1.00E-06	0.012959	-13.66166677
ENSG00000197291	RAMP2-AS1	1.00E-06	0.012984402	-13.66449196
ENSG00000147655	RSPO2	1.00E-06	0.013152386	-13.68303689
ENSG00000185002	RFX6	1.00E-06	0.013222898	-13.69075074
ENSG00000260386	LINC01225	1.00E-06	0.013242315	-13.69286775
ENSG00000240089	BMS1P3	1.00E-06	0.013245	-13.69316022
ENSG00000183292	TISP43	1.00E-06	0.013258322	-13.69461059
ENSG00000279414	bP-2171C21.4	1.00E-06	0.013291	-13.69816204
ENSG00000104938	CLEC4M	1.00E-06	0.013310827	-13.7003126
ENSG00000279378	RP11-70D24.1	1.00E-06	0.013323	-13.70163136
ENSG00000133019	CHRM3	1.00E-06	0.013340983	-13.70357732
ENSG00000263394	RP11-160E2.19	1.00E-06	0.013345	-13.70401169
ENSG00000232079	AL035610.1	1.00E-06	0.01335299	-13.70487525
ENSG00000169347	GP2	1.00E-06	0.013369896	-13.70670065
ENSG00000236922	LINC01378	1.00E-06	0.013372979	-13.70703331
ENSG00000258699	RP11-356K23.2	1.00E-06	0.013393	-13.70919154
ENSG00000260862	RP11-22H5.2	1.00E-06	0.013406	-13.71059122
ENSG00000177283	FZD8	1.00E-06	0.013429	-13.71306426
ENSG00000279811	RP11-862L9.2	1.00E-06	0.013537	-13.72462043
ENSG00000269433	OPN1MW3	1.00E-06	0.013544	-13.72536626
ENSG00000105392	CRX	1.00E-06	0.013550833	-13.72609395
ENSG00000229316	HMGB1P45	1.00E-06	0.013592	-13.73047014
ENSG00000282511	CLUHP10	1.00E-06	0.013622	-13.73365092
ENSG00000231560	AC091814.3	1.00E-06	0.013681	-13.73988607
ENSG00000115155	OTOF	1.00E-06	0.01372754	-13.74478554

ENSG00000272259	RP11-305P22.9	1.00E-06	0.013749756	-13.74711841
ENSG00000184719	RNLS	1.00E-06	0.013760331	-13.7482275
ENSG00000243478	AOX2P	1.00E-06	0.013763809	-13.74859213
ENSG00000005381	MPO	1.00E-06	0.013814653	-13.75391166
ENSG00000260613	RP3-522J7.6	1.00E-06	0.013815	-13.75394794
ENSG00000204174	NPY4R	1.00E-06	0.013834	-13.75593074
ENSG00000264717	CH17-360D5.1	1.00E-06	0.013834	-13.75593074
ENSG00000280118	RP11-385H1.1	1.00E-06	0.013887	-13.76144735
ENSG00000261175	CTD-2015G9.2	1.00E-06	0.01390958	-13.76379122
ENSG00000136859	ANGPTL2	1.00E-06	0.01391674	-13.76453363
ENSG00000267107	PCAT19	1.00E-06	0.013934484	-13.76637196
ENSG00000197430	OPALIN	1.00E-06	0.013952883	-13.76827565
ENSG00000149090	PAMR1	1.00E-06	0.014009269	-13.77409411
ENSG00000261402	RP11-378I6.1	1.00E-06	0.014079	-13.78125725
ENSG00000256195	RP11-64D24.4	1.00E-06	0.014105	-13.78391905
ENSG00000225783	MIAT	1.00E-06	0.01411292	-13.78472889
ENSG00000117501	MROH9	1.00E-06	0.014127251	-13.78619318
ENSG00000223443	USP17L2	1.00E-06	0.014168	-13.7903485
ENSG00000257720	ILF2P2	1.00E-06	0.014212	-13.79482197
ENSG00000230031	POTEB2	1.00E-06	0.014257517	-13.79943518
ENSG00000228679	RP4-676J13.2	1.00E-06	0.01426	-13.79968636
ENSG00000226954	RP5-983L19.2	1.00E-06	0.014337	-13.80745555
ENSG00000180176	TH	1.00E-06	0.014351798	-13.80894384
ENSG00000248810	RP11-362F19.1	1.00E-06	0.014432151	-13.81699876
ENSG00000189325	C6orf222	1.00E-06	0.014486	-13.82237166
ENSG00000230246	SPATA31C1	1.00E-06	0.014526775	-13.82642679
ENSG00000187979	AC008079.9	1.00E-06	0.014556	-13.82932634
ENSG00000183230	CTNNA3	1.00E-06	0.014580348	-13.83173749
ENSG00000280395	RP5-898I4.1	1.00E-06	0.014655	-13.83910535
ENSG00000278862	RP11-571O6.2	1.00E-06	0.014657	-13.83930222
ENSG00000223387	RP11-408H1.3	1.00E-06	0.014759258	-13.8493326
ENSG00000251609	SETP12	1.00E-06	0.014791	-13.85243197
ENSG00000269296	AC005614.3	1.00E-06	0.014815	-13.85477101
ENSG00000276289	KCNE1B	1.00E-06	0.01483132	-13.85635937
ENSG00000229719	MIR194-2HG	1.00E-06	0.01484	-13.85720347
ENSG00000056998	GYG2	1.00E-06	0.014843212	-13.85751567
ENSG00000259646	AC140725.7	1.00E-06	0.014856	-13.8587581
ENSG00000261296	RP11-299H22.6	1.00E-06	0.014933899	-13.86630329
ENSG00000235379	RPL7P31	1.00E-06	0.01495	-13.86785786
ENSG00000196381	ZNF781	1.00E-06	0.014962388	-13.86905287
ENSG00000277630	BX072566.1	1.00E-06	0.014985877	-13.87131591
ENSG00000253706	RP11-758M4.4	1.00E-06	0.014987658	-13.87148731
ENSG00000235997	AC109642.1	1.00E-06	0.015034	-13.87594129
ENSG00000276760	AC136352.4	1.00E-06	0.01504249	-13.87675573
ENSG00000237505	PKN2-AS1	1.00E-06	0.015062126	-13.87863778
ENSG00000218565	RP11-12A2.1	1.00E-06	0.015064	-13.87881728
ENSG00000267784	RP11-171I2.1	1.00E-06	0.015076	-13.87996608
ENSG00000271715	CTD-2256P15.5	1.00E-06	0.015103	-13.88254753

ENSG00000279774	RP11-278H7.5	1.00E-06	0.015117	-13.88388424
ENSG00000231635	ATP5BP1	1.00E-06	0.015129	-13.88502901
ENSG00000229951	FLJ31356	1.00E-06	0.015148	-13.88683971
ENSG00000125851	PCSK2	1.00E-06	0.015261353	-13.8975953
ENSG00000167554	ZNF610	1.00E-06	0.015262162	-13.89767169
ENSG00000205231	TTLL10-AS1	1.00E-06	0.015323	-13.90341116
ENSG00000274234	RN7SL818P	1.00E-06	0.015324	-13.90350531
ENSG00000278242	RN7SL725P	1.00E-06	0.015324	-13.90350531
ENSG00000280780	JAKMIP2-AS1	1.00E-06	0.015419259	-13.91244584
ENSG00000279143	AC139099.7	1.00E-06	0.015445	-13.91485225
ENSG00000263711	RP11-169F17.1	1.00E-06	0.015472274	-13.91739765
ENSG00000205436	EXOC3L4	1.00E-06	0.015551505	-13.9247666
ENSG00000171451	DSEL	1.00E-06	0.015559	-13.92546172
ENSG00000250280	RP11-305O6.3	1.00E-06	0.015655	-13.93433589
ENSG00000181195	PENK	1.00E-06	0.015664387	-13.93520066
ENSG00000224074	LINC00691	1.00E-06	0.015705071	-13.93894288
ENSG00000280711	JADRR	1.00E-06	0.015723	-13.94058889
ENSG00000204941	PSG5	1.00E-06	0.015733804	-13.94157989
ENSG00000260734	RP11-510M2.4	1.00E-06	0.015740048	-13.94215233
ENSG00000004468	CD38	1.00E-06	0.015790196	-13.94674149
ENSG00000206531	CD200R1L	1.00E-06	0.015824309	-13.94985486
ENSG00000271389	OSBPL9P1	1.00E-06	0.015842	-13.95146686
ENSG00000111783	RFX4	1.00E-06	0.015957606	-13.96195659
ENSG00000144229	THSD7B	1.00E-06	0.016007711	-13.96647942
ENSG00000156920	ADGRG4	1.00E-06	0.016009474	-13.96663828
ENSG00000214514	KRT42P	1.00E-06	0.016027514	-13.96826301
ENSG00000105366	SIGLEC8	1.00E-06	0.016043267	-13.96968032
ENSG00000203987	RP11-188C12.3	1.00E-06	0.016045044	-13.96984014
ENSG00000229243	AC098973.1	1.00E-06	0.016047	-13.97001599
ENSG00000205927	OLIG2	1.00E-06	0.016145538	-13.97884786
ENSG00000007264	MATK	1.00E-06	0.016243507	-13.98757556
ENSG00000182263	FIGN	1.00E-06	0.016330712	-13.99530005
ENSG00000280302	RP11-64C12.1	1.00E-06	0.016336	-13.99576715
ENSG00000162992	NEUROD1	1.00E-06	0.016341306	-13.99623563
ENSG00000229120	CYCSP4	1.00E-06	0.016351	-13.99709125
ENSG00000276422	RP11-144A16.8	1.00E-06	0.016366491	-13.99845739
ENSG00000104760	FGL1	1.00E-06	0.016395179	-14.00098404
ENSG00000160883	HK3	1.00E-06	0.016450162	-14.00581415
ENSG00000168418	KCNG4	1.00E-06	0.016517982	-14.01174985
ENSG00000179796	LRRC3B	1.00E-06	0.016545722	-14.01417061
ENSG00000081479	LRP2	1.00E-06	0.016576039	-14.01681171
ENSG00000187815	ZFP69	1.00E-06	0.016662938	-14.02435513
ENSG00000218725	B3GALNT2P1	1.00E-06	0.016717	-14.02902835
ENSG00000100842	EFS	1.00E-06	0.016719878	-14.02927667
ENSG00000232524	RP11-332K15.1	1.00E-06	0.0168	-14.03617361
ENSG00000212066	AL645941.1	1.00E-06	0.01682	-14.03789009
ENSG00000247699	CTB-127C13.1	1.00E-06	0.016854882	-14.04087894
ENSG00000257171	RP11-754I20.3	1.00E-06	0.0169	-14.04473563

ENSG00000122188	LAX1	1.00E-06	0.016923317	-14.04672478
ENSG00000279160	RP11-387A1.6	1.00E-06	0.016939	-14.04806109
ENSG00000225321	LINC01427	1.00E-06	0.016958293	-14.04970334
ENSG00000204290	BTNL2	1.00E-06	0.016959286	-14.04978782
ENSG00000230549	USP17L1	1.00E-06	0.016987	-14.05214347
ENSG00000167094	TTC16	1.00E-06	0.016992584	-14.05261759
ENSG00000111732	AICDA	1.00E-06	0.017026217	-14.0554703
ENSG00000227679	RP11-453N3.1	1.00E-06	0.01704	-14.05663772
ENSG00000134202	GSTM3	1.00E-06	0.017091156	-14.06096234
ENSG00000163687	DNASE1L3	1.00E-06	0.017192064	-14.0694551
ENSG00000084734	GCKR	1.00E-06	0.017203111	-14.07038189
ENSG00000125998	FAM83C	1.00E-06	0.017208	-14.07079181
ENSG00000231484	RP11-548K12.12	1.00E-06	0.017287	-14.0773999
ENSG00000224743	TEX26-AS1	1.00E-06	0.017315667	-14.07979033
ENSG00000157211	CDCP2	1.00E-06	0.017329371	-14.08093165
ENSG00000213075	RPL31P11	1.00E-06	0.017368436	-14.08418027
ENSG00000177947	ODF3	1.00E-06	0.0174021	-14.08697379
ENSG00000233485	RP3-467K16.2	1.00E-06	0.01743	-14.08928495
ENSG00000159648	TEPP	1.00E-06	0.017441341	-14.09022337
ENSG00000279526	AC011239.2	1.00E-06	0.017446	-14.09060867
ENSG00000196778	OR52K1	1.00E-06	0.017514	-14.096221
ENSG00000228918	LINC01344	1.00E-06	0.017532232	-14.09772202
ENSG00000261467	RP11-731K22.1	1.00E-06	0.017546	-14.09885455
ENSG00000114200	BCHE	1.00E-06	0.017560272	-14.10002759
ENSG00000271167	LINC01109	1.00E-06	0.017565	-14.10041596
ENSG00000132297	HHLA1	1.00E-06	0.017620473	-14.10496506
ENSG00000279132	FAM231C	1.00E-06	0.017652	-14.10754403
ENSG00000239265	CLRN1-AS1	1.00E-06	0.017663563	-14.10848879
ENSG00000121797	CCRL2	1.00E-06	0.017680601	-14.1098797
ENSG00000237847	FAM231A	1.00E-06	0.017687	-14.11040174
ENSG00000268674	FAM231B	1.00E-06	0.017687	-14.11040174
ENSG00000233817	RP11-168K11.3	1.00E-06	0.017785	-14.11837335
ENSG00000065325	GLP2R	1.00E-06	0.017884588	-14.12642928
ENSG00000214869	TPM3P4	1.00E-06	0.01792	-14.12928302
ENSG00000105697	HAMP	1.00E-06	0.017980254	-14.13412577
ENSG00000231072	GAPDHP64	1.00E-06	0.017986	-14.13458675
ENSG00000236283	AC013463.2	1.00E-06	0.018004158	-14.13604254
ENSG00000279006	CTA-243E7.2	1.00E-06	0.018041	-14.13899169
ENSG00000247775	SNCA-AS1	1.00E-06	0.018099879	-14.14369243
ENSG00000182824	AC008132.15	1.00E-06	0.018100564	-14.14374699
ENSG00000226321	CROCC2	1.00E-06	0.018105956	-14.14417669
ENSG00000131002	TXLNGY	1.00E-06	0.018136427	-14.14660264
ENSG00000157060	SHCBP1L	1.00E-06	0.018149108	-14.14761102
ENSG00000155897	ADCY8	1.00E-06	0.018158665	-14.14837052
ENSG00000226258	GRM7-AS3	1.00E-06	0.018197748	-14.15147232
ENSG00000242560	RN7SL797P	1.00E-06	0.018222	-14.15339369
ENSG00000225564	LINC01492	1.00E-06	0.018239343	-14.15476615
ENSG00000216917	RP5-988G15.1	1.00E-06	0.018284	-14.1582941

ENSG00000256994	RP11-871F6.3	1.00E-06	0.018346	-14.16317792
ENSG00000255331	RP11-736I10.2	1.00E-06	0.018359	-14.16419986
ENSG00000273675	RP11-406H23.5	1.00E-06	0.01839	-14.16663386
ENSG00000240747	KRBOX1	1.00E-06	0.018390103	-14.16664191
ENSG00000245466	CTD-2540L5.6	1.00E-06	0.018402	-14.16757495
ENSG00000215196	AC091878.1	1.00E-06	0.018402	-14.16757495
ENSG00000280890	ELDR	1.00E-06	0.018402	-14.16757495
ENSG00000179142	CYP11B2	1.00E-06	0.018433	-14.17000327
ENSG00000204277	RP11-219G17.4	1.00E-06	0.018446	-14.17102038
ENSG00000233917	POTEB	1.00E-06	0.018454891	-14.17171558
ENSG00000246548	RP11-7F17.5	1.00E-06	0.018509	-14.17593933
ENSG00000109047	RCVRN	1.00E-06	0.01852858	-14.17746467
ENSG00000264956	RP11-1109M24.5	1.00E-06	0.018534337	-14.17791289
ENSG00000251587	LDHAP1	1.00E-06	0.018549	-14.17905379
ENSG00000260505	RP11-410C4.5	1.00E-06	0.018585	-14.18185107
ENSG00000151650	VENTX	1.00E-06	0.018591	-14.18231675
ENSG00000253500	AF121898.3	1.00E-06	0.018598082	-14.18286626
ENSG00000196458	ZNF605	1.00E-06	0.018600221	-14.18303213
ENSG00000242428	C3orf67-AS1	1.00E-06	0.018603084	-14.18325421
ENSG00000224310	LINC01567	1.00E-06	0.018617	-14.18433299
ENSG00000224655	AC018737.3	1.00E-06	0.018649759	-14.18686935
ENSG00000172985	SH3RF3	1.00E-06	0.018652	-14.18704271
ENSG00000234724	HDAC1P1	1.00E-06	0.018727	-14.19283218
ENSG00000165606	DRGX	1.00E-06	0.018727352	-14.19285927
ENSG00000236097	BNIP3P2	1.00E-06	0.018759	-14.1952953
ENSG00000138068	SULT6B1	1.00E-06	0.018789054	-14.19760484
ENSG00000281560	LSINCT5	1.00E-06	0.01881	-14.19921223
ENSG00000186367	KIAA1024L	1.00E-06	0.018852	-14.20242997
ENSG00000278913	CTD-2299N12.1	1.00E-06	0.018864	-14.203348
ENSG00000111058	ACSS3	1.00E-06	0.018921808	-14.20776236
ENSG00000198771	RCSD1	1.00E-06	0.018929767	-14.20836904
ENSG00000182487	NCF1B	1.00E-06	0.018969926	-14.21142646
ENSG00000265631	BNIP3P3	1.00E-06	0.019023	-14.21545716
ENSG00000176029	C11orf16	1.00E-06	0.019099029	-14.22121166
ENSG00000271696	RP11-548K12.13	1.00E-06	0.019131	-14.22362467
ENSG00000237202	AJ239321.3	1.00E-06	0.019191	-14.22814227
ENSG00000273824	RP11-81H14.1	1.00E-06	0.019232	-14.23122118
ENSG00000237358	AC007064.25	1.00E-06	0.019273	-14.23429354
ENSG00000111341	MGP	1.00E-06	0.019277231	-14.2346102
ENSG00000240069	GPAA1P1	1.00E-06	0.019377	-14.24205761
ENSG00000261432	LINC01613	1.00E-06	0.019468	-14.24881706
ENSG00000261245	RP11-120K18.3	1.00E-06	0.019503	-14.25140844
ENSG00000254957	RP11-179A10.2	1.00E-06	0.01951	-14.25192616
ENSG00000176584	DMBT1P1	1.00E-06	0.019537469	-14.25395597
ENSG00000236564	YWHAQP5	1.00E-06	0.019581	-14.25716682
ENSG00000262480	SAMD11P1	1.00E-06	0.01965	-14.26224169
ENSG00000255450	CTD-2063L20.1	1.00E-06	0.019803	-14.27343138
ENSG00000178789	CD300LB	1.00E-06	0.019896604	-14.28023462

ENSG00000234075	RPL35AP	1.00E-06	0.019922	-14.28207487
ENSG00000261826	RP11-691G17.1	1.00E-06	0.019978	-14.28612454
ENSG00000156269	NAA11	1.00E-06	0.020037079	-14.29038458
ENSG00000281531	Metazoa_SRP	1.00E-06	0.020045	-14.2909548
ENSG00000168671	UGT3A2	1.00E-06	0.020089182	-14.29413117
ENSG00000224129	DPPA2P2	1.00E-06	0.020111	-14.2956972
ENSG00000250910	AC097467.2	1.00E-06	0.020122895	-14.29655022
ENSG00000226194	RP1-137D17.1	1.00E-06	0.020157242	-14.29901062
ENSG00000251165	F11-AS1	1.00E-06	0.020171647	-14.30004124
ENSG00000269711	CTD-3214H19.16	1.00E-06	0.020191	-14.30142474
ENSG00000280255	RP5-1007F24.1	1.00E-06	0.020263	-14.30656016
ENSG00000187533	PRR27	1.00E-06	0.020285379	-14.30815266
ENSG00000214188	ST7-OT4	1.00E-06	0.020315345	-14.31028222
ENSG00000278972	CTD-3022L24.1	1.00E-06	0.020323	-14.31082576
ENSG00000178187	ZNF454	1.00E-06	0.020330975	-14.3113918
ENSG00000233392	AC104809.4	1.00E-06	0.020376958	-14.31465106
ENSG00000110148	CCKBR	1.00E-06	0.020383877	-14.31514087
ENSG00000269332	GOLGA2P9	1.00E-06	0.020438253	-14.31898424
ENSG00000261649	GOLGA6L7P	1.00E-06	0.020461856	-14.32064936
ENSG00000225860	RP11-1018N14.2	1.00E-06	0.020462	-14.32065954
ENSG00000233864	TTTY15	1.00E-06	0.020485	-14.32228027
ENSG00000176571	CNBD1	1.00E-06	0.020525727	-14.32514571
ENSG00000163586	FABP1	1.00E-06	0.020597007	-14.33014711
ENSG00000184624	ZNF72P	1.00E-06	0.020598	-14.33021664
ENSG00000234647	RP11-503C24.4	1.00E-06	0.02062571	-14.33215613
ENSG00000266256	LINC00683	1.00E-06	0.020646891	-14.33363695
ENSG00000228980	LINC01205	1.00E-06	0.020672697	-14.33543903
ENSG00000229323	DLEU1-AS1	1.00E-06	0.02073	-14.3394325
ENSG00000188011	RTP5	1.00E-06	0.020768074	-14.34207979
ENSG00000270749	RP11-368M16.10	1.00E-06	0.020807	-14.34478135
ENSG00000106331	PAX4	1.00E-06	0.020847943	-14.34761742
ENSG00000236243	RPL6P29	1.00E-06	0.020856	-14.34817487
ENSG00000236494	RP11-89N17.4	1.00E-06	0.02087661	-14.34959985
ENSG00000233183	RP3-468B3.2	1.00E-06	0.02088377	-14.35009452
ENSG00000280032	RP11-832A4.7	1.00E-06	0.020919	-14.35252627
ENSG00000185905	C16orf54	1.00E-06	0.020936	-14.35369821
ENSG00000272662	RP11-190C22.8	1.00E-06	0.020939	-14.35390492
ENSG00000270496	BNIP3P7	1.00E-06	0.020977	-14.35652075
ENSG00000261235	RP11-510J16.5	1.00E-06	0.021017162	-14.35928026
ENSG00000225443	AC004938.5	1.00E-06	0.021086	-14.36399782
ENSG00000204603	LINC01257	1.00E-06	0.021123783	-14.3665806
ENSG00000266733	TBC1D29	1.00E-06	0.021129928	-14.36700021
ENSG00000233848	RP3-348I23.2	1.00E-06	0.021155	-14.36871107
ENSG00000267257	RP11-1151B14.4	1.00E-06	0.021199	-14.37170859
ENSG00000248360	LINC00504	1.00E-06	0.021244533	-14.37480401
ENSG00000104941	RSPH6A	1.00E-06	0.021248691	-14.37508635
ENSG00000166840	GLYATL1	1.00E-06	0.021258165	-14.37572947
ENSG00000268221	OPN1MW	1.00E-06	0.021274132	-14.37681265

ENSG00000151360	ALLC	1.00E-06	0.021290672	-14.37793386
ENSG00000211897	IGHG3	1.00E-06	0.021341	-14.38134016
ENSG00000279020	C18orf15	1.00E-06	0.021358	-14.38248894
ENSG00000273825	CH17-164J11.1	1.00E-06	0.021453	-14.38889179
ENSG00000102076	OPN1LW	1.00E-06	0.02146	-14.38936246
ENSG00000166160	OPN1MW2	1.00E-06	0.02146	-14.38936246
ENSG00000258529	RP11-108O10.8	1.00E-06	0.0214742	-14.39031674
ENSG00000269706	CTB-60B18.15	1.00E-06	0.021476	-14.39043769
ENSG00000168852	TPTE2P5	1.00E-06	0.021501022	-14.39211765
ENSG00000072315	TRPC5	1.00E-06	0.021503	-14.39225033
ENSG00000182938	OTOP3	1.00E-06	0.021511	-14.39278697
ENSG00000239413	RPS27P23	1.00E-06	0.021562	-14.39620338
ENSG00000279606	RP11-502F1.1	1.00E-06	0.021596	-14.3984765
ENSG00000178222	RNF212	1.00E-06	0.021635243	-14.40109572
ENSG00000279024	RP11-1193F23.1	1.00E-06	0.021709	-14.40600565
ENSG00000255366	RP11-1134I14.8	1.00E-06	0.021726	-14.40713496
ENSG00000156096	UGT2B4	1.00E-06	0.021743955	-14.40832678
ENSG00000225872	LINC01529	1.00E-06	0.021751872	-14.40885196
ENSG00000275767	CH17-248H7.3	1.00E-06	0.021811	-14.4127683
ENSG00000142789	CELA3A	1.00E-06	0.02181364	-14.41294289
ENSG00000136918	WDR38	1.00E-06	0.021850959	-14.41540897
ENSG00000204296	C6orf10	1.00E-06	0.021875434	-14.417024
ENSG00000266818	RP11-454P7.1	1.00E-06	0.021883	-14.41752291
ENSG00000146910	CNPY1	1.00E-06	0.021884716	-14.41763602
ENSG00000110777	POU2AF1	1.00E-06	0.021893677	-14.41822668
ENSG00000081051	AFP	1.00E-06	0.021920786	-14.42001188
ENSG00000278954	RP13-415G19.2	1.00E-06	0.021947	-14.42173613
ENSG00000101349	PAK7	1.00E-06	0.021960459	-14.42262056
ENSG00000267099	NTF6G	1.00E-06	0.021964	-14.4228532
ENSG00000244039	RP11-379K17.2	1.00E-06	0.021964	-14.4228532
ENSG00000185105	MYADML2	1.00E-06	0.02201912	-14.42646917
ENSG00000127412	TRPV5	1.00E-06	0.022024675	-14.42683311
ENSG00000223738	RP11-781E19.1	1.00E-06	0.02209	-14.4311058
ENSG00000174495	AC005017.2	1.00E-06	0.02209	-14.4311058
ENSG00000280264	AC004158.1	1.00E-06	0.022129	-14.43365064
ENSG00000279537	RP11-133K1.8	1.00E-06	0.022162	-14.43580046
ENSG00000124827	GCM2	1.00E-06	0.022171	-14.43638622
ENSG00000184224	C11orf72	1.00E-06	0.02223556	-14.44058112
ENSG00000105851	PIK3CG	1.00E-06	0.022268759	-14.44273356
ENSG00000206384	COL6A6	1.00E-06	0.022271882	-14.44293587
ENSG00000245870	LINC00682	1.00E-06	0.022283606	-14.44369508
ENSG00000279684	RP11-755E23.2	1.00E-06	0.022362	-14.4487616
ENSG00000150630	VEGFC	1.00E-06	0.022363696	-14.44887101
ENSG00000213924	HNRNPH1P2	1.00E-06	0.022364	-14.44889063
ENSG00000233410	LINC01222	1.00E-06	0.022436	-14.45352787
ENSG00000277095	CH17-333M13.1	1.00E-06	0.022463	-14.455263
ENSG00000269964	MEI4	1.00E-06	0.02255	-14.46083981
ENSG00000133101	CCNA1	1.00E-06	0.022550349	-14.46086213

ENSG00000280073	CTD-2525P14.5	1.00E-06	0.0226	-14.46403515
ENSG00000119673	ACOT2	1.00E-06	0.022616461	-14.4650856
ENSG00000268066	FMR1-AS1	1.00E-06	0.022673	-14.46868767
ENSG00000281196	LINC00934	1.00E-06	0.022731031	-14.47237548
ENSG00000250372	MARK2P4	1.00E-06	0.022739	-14.47288119
ENSG00000235295	FLJ41941	1.00E-06	0.022749	-14.47351551
ENSG00000148156	ACTL7B	1.00E-06	0.022749	-14.47351551
ENSG00000253967	RP11-333A23.4	1.00E-06	0.022865	-14.4808533
ENSG00000279933	RP4-671O14.5	1.00E-06	0.022986	-14.48846781
ENSG00000260604	RP1-140K8.5	1.00E-06	0.022997	-14.48915805
ENSG00000182162	P2RY8	1.00E-06	0.023061227	-14.49318164
ENSG00000263904	RP11-344B2.3	1.00E-06	0.023129	-14.49741527
ENSG00000100665	SERPINA4	1.00E-06	0.023148327	-14.49862029
ENSG00000214031	RP11-169L17.3	1.00E-06	0.023187	-14.50102855
ENSG00000233038	AC011899.9	1.00E-06	0.023218066	-14.50296016
ENSG00000153802	TMPRSS11D	1.00E-06	0.023330517	-14.50993064
ENSG00000147117	ZNF157	1.00E-06	0.023348	-14.51101135
ENSG00000268894	PLCE1-AS1	1.00E-06	0.02335789	-14.51162233
ENSG00000268104	SLC6A14	1.00E-06	0.023468	-14.51840728
ENSG00000100565	LRRC74A	1.00E-06	0.023479677	-14.51912496
ENSG00000251210	RP11-168E17.1	1.00E-06	0.023482083	-14.51927275
ENSG00000269779	CTD-2542C24.2	1.00E-06	0.023597	-14.52631583
ENSG00000270954	RPSAP75	1.00E-06	0.023631	-14.52839306
ENSG00000263429	LINC00675	1.00E-06	0.023644442	-14.52921346
ENSG00000233867	SLC9B1P3	1.00E-06	0.023666	-14.53052826
ENSG00000105509	HAS1	1.00E-06	0.02366759	-14.53062521
ENSG00000198049	AVPR1B	1.00E-06	0.023809829	-14.53926963
ENSG00000260239	RP11-274H24.1	1.00E-06	0.023821	-14.53994636
ENSG00000139648	KRT71	1.00E-06	0.023894	-14.54436077
ENSG00000243499	RPS6P21	1.00E-06	0.023894	-14.54436077
ENSG00000178125	PPP1R42	1.00E-06	0.023914652	-14.5456072
ENSG00000274167	AL031601.5	1.00E-06	0.024031869	-14.55266121
ENSG00000229775	RP11-298H24.1	1.00E-06	0.024053419	-14.55395436
ENSG00000156265	MAP3K7CL	1.00E-06	0.024069267	-14.5549046
ENSG00000281721	LINC01080	1.00E-06	0.02414	-14.55913806
ENSG00000267019	NTF6A	1.00E-06	0.02416	-14.56033283
ENSG00000237551	AC096775.2	1.00E-06	0.02416	-14.56033283
ENSG00000197826	C4orf22	1.00E-06	0.024172182	-14.56106006
ENSG00000211892	IGHG4	1.00E-06	0.024182	-14.56164595
ENSG00000223917	AC009237.2	1.00E-06	0.024247	-14.56551864
ENSG00000172482	AGXT	1.00E-06	0.024275043	-14.56718622
ENSG00000144191	CNGA3	1.00E-06	0.024275155	-14.56719289
ENSG00000149534	MS4A2	1.00E-06	0.024307961	-14.56914128
ENSG00000249781	CTD-2143L24.1	1.00E-06	0.024372676	-14.57297706
ENSG00000281514	Metazoa_SRP	1.00E-06	0.024429	-14.57630719
ENSG00000214851	LINC00612	1.00E-06	0.024456	-14.57790084
ENSG00000204460	AC079586.1	1.00E-06	0.024456	-14.57790084
ENSG00000279502	RP11-354E23.4	1.00E-06	0.024481	-14.57937487

ENSG00000279391	RP11-236J17.7	1.00E-06	0.0245	-14.58049413
ENSG00000251435	C1GALT1P2	1.00E-06	0.024511	-14.58114172
ENSG00000225017	AP004289.1	1.00E-06	0.024545	-14.58314155
ENSG00000230753	ZNF341-AS1	1.00E-06	0.024635266	-14.58843743
ENSG00000267058	RP11-15A1.3	1.00E-06	0.024679	-14.59099632
ENSG00000268240	RP11-678G14.4	1.00E-06	0.024725452	-14.59370928
ENSG00000264813	CTD-2501B8.1	1.00E-06	0.024801	-14.59811067
ENSG00000057149	SERPINB3	1.00E-06	0.024803076	-14.59823143
ENSG00000280404	AC005086.3	1.00E-06	0.024826	-14.59956421
ENSG00000230630	DNM3OS	1.00E-06	0.024837	-14.6002033
ENSG00000231371	AKR1B1P8	1.00E-06	0.024863	-14.60171276
ENSG00000239961	LILRA4	1.00E-06	0.024929047	-14.60554008
ENSG00000179855	GIPC3	1.00E-06	0.024969	-14.60785042
ENSG00000270708	RP11-568G11.5	1.00E-06	0.025006	-14.60998668
ENSG00000137634	NXPE4	1.00E-06	0.025021123	-14.61085889
ENSG00000279030	RP11-212I21.3	1.00E-06	0.025056	-14.6128685
ENSG00000224721	AC007182.6	1.00E-06	0.025068	-14.61355928
ENSG00000153446	C16orf89	1.00E-06	0.025135698	-14.61745014
ENSG00000264801	ERVFRD-3	1.00E-06	0.025141	-14.61775441
ENSG00000236983	LINC00614	1.00E-06	0.025173	-14.61958954
ENSG00000258283	RP11-386G11.3	1.00E-06	0.02524	-14.62342429
ENSG00000168634	WFDC13	1.00E-06	0.025246	-14.6237672
ENSG00000242175	RN7SL127P	1.00E-06	0.025256	-14.62433855
ENSG00000278908	RP11-523J2.1	1.00E-06	0.025361	-14.63032401
ENSG00000250501	RP11-98O2.1	1.00E-06	0.025384523	-14.63166156
ENSG00000264609	AL603910.1	1.00E-06	0.025468	-14.63639805
ENSG00000109906	ZBTB16	1.00E-06	0.025480261	-14.63709245
ENSG00000181013	C17orf47	1.00E-06	0.025493	-14.63781354
ENSG00000282865	RP11-306O1.2	1.00E-06	0.025564	-14.64182597
ENSG00000265740	RN7SL339P	1.00E-06	0.025598	-14.64374347
ENSG00000278454	ZFAT-AS1_3	1.00E-06	0.025745	-14.65200465
ENSG00000267872	RP11-157B13.7	1.00E-06	0.025846	-14.6576534
ENSG00000227517	LINC01483	1.00E-06	0.025938248	-14.66279343
ENSG00000171827	ZNF570	1.00E-06	0.025994511	-14.66591937
ENSG00000203334	RP11-25I9.3	1.00E-06	0.026019	-14.66727789
ENSG00000019169	MARCO	1.00E-06	0.026031703	-14.66798206
ENSG00000236847	AC018892.3	1.00E-06	0.026032	-14.66799854
ENSG00000237990	CNTN4-AS1	1.00E-06	0.026045063	-14.66872228
ENSG00000132010	ZNF20	1.00E-06	0.026094508	-14.6714586
ENSG00000135423	GLS2	1.00E-06	0.026138846	-14.67390782
ENSG00000229572	EIF4BP4	1.00E-06	0.026148	-14.67441298
ENSG00000280515	SALRNA2	1.00E-06	0.026195	-14.67700384
ENSG00000248668	OXCT1-AS1	1.00E-06	0.026208094	-14.67772484
ENSG00000251408	RP11-586D19.2	1.00E-06	0.026266	-14.68090889
ENSG00000247011	RP11-700H6.1	1.00E-06	0.026349	-14.68546059
ENSG00000267260	CTD-2162K18.4	1.00E-06	0.026399951	-14.68824764
ENSG00000279620	RP11-1102P22.2	1.00E-06	0.0264	-14.68825031
ENSG00000261028	AC012322.1	1.00E-06	0.0264	-14.68825031

ENSG00000250640	OR7E41P	1.00E-06	0.026426	-14.68967045
ENSG00000255639	RP11-234B24.6	1.00E-06	0.026513265	-14.69442671
ENSG00000231102	RP11-298J23.5	1.00E-06	0.02653	-14.69533706
ENSG00000226548	AC016722.3	1.00E-06	0.026562	-14.69707616
ENSG00000235097	LINC00330	1.00E-06	0.026582	-14.69816204
ENSG00000277498	RP11-681L4.2	1.00E-06	0.026608	-14.69957245
ENSG00000237267	LINC01519	1.00E-06	0.026674	-14.70314657
ENSG00000234210	AC006372.4	1.00E-06	0.026732	-14.70628016
ENSG00000229569	RP11-481G8.2	1.00E-06	0.026819	-14.71096782
ENSG00000253327	RAD21-AS1	1.00E-06	0.026926	-14.71671231
ENSG00000152430	BOLL	1.00E-06	0.026942736	-14.71760874
ENSG00000184033	CTAG1B	1.00E-06	0.026978835	-14.71954046
ENSG00000249852	AC145676.2	1.00E-06	0.027006	-14.72099235
ENSG00000178386	ZNF223	1.00E-06	0.027017183	-14.72158965
ENSG00000234835	PHBP13	1.00E-06	0.02706	-14.72387422
ENSG00000176320	RP11-404O13.5	1.00E-06	0.027074	-14.72462043
ENSG00000268651	CTAG1A	1.00E-06	0.027114	-14.72675034
ENSG00000213927	CCL27	1.00E-06	0.027132149	-14.72771569
ENSG00000213035	RPL23AP80	1.00E-06	0.02723	-14.73290936
ENSG00000187054	TMPRSS11A	1.00E-06	0.027264985	-14.73476176
ENSG00000259008	RP11-932A10.1	1.00E-06	0.027299	-14.73656048
ENSG00000264958	ALOX12P1	1.00E-06	0.027303	-14.73677186
ENSG00000125845	BMP2	1.00E-06	0.027354	-14.73946419
ENSG00000196364	PRSS29P	1.00E-06	0.027528606	-14.74864395
ENSG00000224403	DPPA2P3	1.00E-06	0.027542	-14.74934571
ENSG00000260416	RP5-963E22.5	1.00E-06	0.027542	-14.74934571
ENSG00000163424	C3orf30	1.00E-06	0.027556865	-14.75012413
ENSG00000263761	GDF2	1.00E-06	0.027683	-14.75671268
ENSG00000229717	RP11-556N4.1	1.00E-06	0.027683	-14.75671268
ENSG00000280134	RP11-113E21.3	1.00E-06	0.027712	-14.75822321
ENSG00000260062	GOLGA2P11	1.00E-06	0.027717194	-14.75849357
ENSG00000177453	NIM1K	1.00E-06	0.02781591	-14.76362268
ENSG00000249367	FABP5P12	1.00E-06	0.027826	-14.76414592
ENSG00000196475	GK2	1.00E-06	0.027869	-14.76637362
ENSG00000260455	NBAT1	1.00E-06	0.027897	-14.76782236
ENSG00000182397	DNM1P46	1.00E-06	0.027929039	-14.76947834
ENSG00000179571	NBPF17P	1.00E-06	0.027941	-14.77009603
ENSG00000248869	RP11-138I17.1	1.00E-06	0.027968955	-14.77153873
ENSG00000275340	FGD5P1	1.00E-06	0.027975684	-14.77188577
ENSG00000269741	CTC-518B2.8	1.00E-06	0.02799812	-14.77304234
ENSG00000259251	RP11-643M14.1	1.00E-06	0.028012595	-14.773788
ENSG00000223910	ZNF32-AS3	1.00E-06	0.028013	-14.77380887
ENSG00000197253	TPSB2	1.00E-06	0.028025633	-14.77445933
ENSG00000261233	ABCD1P3	1.00E-06	0.028029	-14.77463265
ENSG00000271499	CTC-559E9.9	1.00E-06	0.028085	-14.77751218
ENSG00000279805	CTA-212A2.1	1.00E-06	0.028173	-14.78202558
ENSG00000237469	TUBB8P10	1.00E-06	0.02821	-14.78391905
ENSG00000160654	CD3G	1.00E-06	0.028216737	-14.78426355

ENSG00000257322	RP11-511B23.2	1.00E-06	0.028276196	-14.78730045
ENSG00000260997	RP4-647J21.1	1.00E-06	0.02832	-14.78953364
ENSG00000283001	RP11-317C20.9	1.00E-06	0.028378	-14.7924853
ENSG00000228252	COL6A4P2	1.00E-06	0.028379913	-14.79258257
ENSG00000279257	C17orf100	1.00E-06	0.028425	-14.79487273
ENSG00000180061	TMEM150B	1.00E-06	0.028434515	-14.79535557
ENSG00000143536	CRNN	1.00E-06	0.028454	-14.79634386
ENSG00000255526	NEDD8-MDP1	1.00E-06	0.028523598	-14.79986833
ENSG00000255146	RP11-659P15.1	1.00E-06	0.028635	-14.80549198
ENSG00000261055	RP11-195M16.3	1.00E-06	0.02865	-14.80624752
ENSG00000228784	LINC00954	1.00E-06	0.02871704	-14.80961943
ENSG00000258352	RP11-23J18.1	1.00E-06	0.028721114	-14.80982411
ENSG00000223841	RP11-448K10.1	1.00E-06	0.028726	-14.8100695
ENSG00000260868	RP11-394I13.1	1.00E-06	0.028726	-14.8100695
ENSG00000262628	OR1D5	1.00E-06	0.028818	-14.81468259
ENSG00000255095	OR1D4	1.00E-06	0.028818	-14.81468259
ENSG00000240063	RP11-225N10.1	1.00E-06	0.028833	-14.81543333
ENSG00000254235	RP11-115J16.1	1.00E-06	0.028878189	-14.81769266
ENSG00000116218	NPHS2	1.00E-06	0.028879202	-14.81774327
ENSG00000120949	TNFRSF8	1.00E-06	0.028957484	-14.82164865
ENSG00000260466	RP4-536B24.2	1.00E-06	0.02903	-14.82525695
ENSG00000232884	AF127936.3	1.00E-06	0.029049816	-14.82624143
ENSG00000267659	LINC01482	1.00E-06	0.02907195	-14.82734023
ENSG00000105501	SIGLEC5	1.00E-06	0.029160328	-14.83171931
ENSG00000176907	C8orf4	1.00E-06	0.029192	-14.83328544
ENSG00000005243	COPZ2	1.00E-06	0.029238601	-14.83558665
ENSG00000251614	HSPA8P19	1.00E-06	0.029254	-14.83634628
ENSG00000241652	RN7SL253P	1.00E-06	0.029254	-14.83634628
ENSG00000230023	RP11-10N16.2	1.00E-06	0.029271	-14.83718441
ENSG00000237234	RP1-142L7.5	1.00E-06	0.029409245	-14.8439821
ENSG00000278561	PTPN20CP	1.00E-06	0.029413	-14.84416632
ENSG00000156689	GLYATL2	1.00E-06	0.029445941	-14.84578117
ENSG00000259084	RP11-1070N10.6	1.00E-06	0.029477714	-14.847337
ENSG00000272213	MIR484	1.00E-06	0.029521	-14.84945397
ENSG00000257654	RP11-497G19.2	1.00E-06	0.029574	-14.85204177
ENSG00000224652	LINC00885	1.00E-06	0.029606	-14.85360196
ENSG00000215043	GLULP6	1.00E-06	0.029639	-14.85520915
ENSG00000233379	RP11-318G21.4	1.00E-06	0.03	-14.87267488
ENSG00000268745	RP11-298J23.9	1.00E-06	0.030043	-14.87474126
ENSG00000137707	BTG4	1.00E-06	0.030050336	-14.87509352
ENSG00000229031	MTCO1P25	1.00E-06	0.030083	-14.87666083
ENSG00000255352	PPP1R10P1	1.00E-06	0.030151	-14.87991823
ENSG00000253148	RGS21	1.00E-06	0.030168	-14.88073144
ENSG00000219273	RP1-217P22.2	1.00E-06	0.030235	-14.88393196
ENSG00000254090	MTND2P32	1.00E-06	0.03024	-14.88417052
ENSG00000270623	RP11-316M21.9	1.00E-06	0.030265	-14.88536273
ENSG00000226397	C12orf77	1.00E-06	0.030289361	-14.88652354
ENSG00000259356	RP11-90E5.1	1.00E-06	0.030388	-14.89121411

ENSG00000266505	RP11-629E24.2	1.00E-06	0.030405	-14.89202097
ENSG00000279790	RP11-374N7.2	1.00E-06	0.030577	-14.90015925
ENSG00000092051	JPH4	1.00E-06	0.030593734	-14.90094859
ENSG00000218109	KIRREL3-AS3	1.00E-06	0.030612	-14.90180968
ENSG00000135838	NPL	1.00E-06	0.030677464	-14.90489161
ENSG00000249201	CTD-3080P12.3	1.00E-06	0.030733033	-14.90750253
ENSG00000274956	UG0898H09	1.00E-06	0.03075	-14.90829879
ENSG00000162989	KCNJ3	1.00E-06	0.030762172	-14.90886974
ENSG00000234323	RP11-308N19.1	1.00E-06	0.030767823	-14.90913473
ENSG00000259476	RP11-50C13.2	1.00E-06	0.030803	-14.91078325
ENSG00000180116	C12orf40	1.00E-06	0.030803804	-14.91082089
ENSG00000274841	Metazoa_SRP	1.00E-06	0.030816	-14.91139199
ENSG00000277215	SPANXA2-OT1	1.00E-06	0.030816	-14.91139199
ENSG00000229160	AC009229.6	1.00E-06	0.030838	-14.91242158
ENSG00000112936	C7	1.00E-06	0.030878125	-14.91429751
ENSG00000274424	RN7SL196P	1.00E-06	0.030997	-14.91984097
ENSG00000277031	RN7SL796P	1.00E-06	0.030997	-14.91984097
ENSG00000277464	RN7SL286P	1.00E-06	0.030997	-14.91984097
ENSG00000276832	RP11-187C18.4	1.00E-06	0.03105	-14.92230565
ENSG00000260120	RP11-177B4.2	1.00E-06	0.031086	-14.92397737
ENSG00000254936	AF131215.3	1.00E-06	0.031086	-14.92397737
ENSG00000225638	PABPC1P12	1.00E-06	0.031104	-14.9248125
ENSG00000235862	RP11-338C15.5	1.00E-06	0.031134	-14.92620332
ENSG00000211695	TRGV9	1.00E-06	0.031193	-14.92893469
ENSG00000105141	CASP14	1.00E-06	0.031200893	-14.92929969
ENSG00000279202	CTA-113A6.1	1.00E-06	0.03123	-14.93064495
ENSG00000229369	RP13-225O21.5	1.00E-06	0.031241	-14.93115301
ENSG00000261821	CTD-2311M21.3	1.00E-06	0.031295	-14.93364456
ENSG00000256218	RP11-1038A11.2	1.00E-06	0.031338	-14.93562549
ENSG00000169777	TAS2R1	1.00E-06	0.031338052	-14.93562789
ENSG00000183566	BPIFA4P	1.00E-06	0.031374293	-14.93729534
ENSG00000186827	TNFRSF4	1.00E-06	0.031447086	-14.94063872
ENSG00000166428	PLD4	1.00E-06	0.031492642	-14.94272717
ENSG00000266217	CTSLP2	1.00E-06	0.031593	-14.94731732
ENSG00000274331	RP11-403A3.1	1.00E-06	0.031686	-14.95155793
ENSG00000213892	CEACAM16	1.00E-06	0.031797307	-14.95661695
ENSG00000266965	RP11-712P20.2	1.00E-06	0.031854	-14.95918693
ENSG00000235740	PHACTR2-AS1	1.00E-06	0.031854	-14.95918693
ENSG00000273104	CMP21-97G8.1	1.00E-06	0.032082	-14.96947646
ENSG00000249497	RP11-1079K10.5	1.00E-06	0.03209	-14.96983617
ENSG00000171671	SHANK2-AS3	1.00E-06	0.032157	-14.9728452
ENSG00000228754	RP11-534L6.3	1.00E-06	0.032204	-14.97495227
ENSG00000200243	RN7SKP79	1.00E-06	0.032215	-14.97544497
ENSG00000275805	RP11-349H17.2	1.00E-06	0.032253	-14.97714574
ENSG00000259498	RP11-244F12.3	1.00E-06	0.032272	-14.97799537
ENSG00000248801	C8orf34-AS1	1.00E-06	0.032329749	-14.98057469
ENSG00000232862	RP4-665N4.4	1.00E-06	0.032331	-14.98063051
ENSG00000223714	RP5-1172N10.2	1.00E-06	0.032407	-14.98401785

ENSG00000231046	RP11-428F8.2	1.00E-06	0.032497	-14.98801892
ENSG00000219491	TPT1P8	1.00E-06	0.032505	-14.98837403
ENSG00000227497	PABPC1P6	1.00E-06	0.032549	-14.9903256
ENSG00000106809	OGN	1.00E-06	0.032593328	-14.99228906
ENSG00000185615	PDIA2	1.00E-06	0.032741659	-14.99883981
ENSG00000230821	KB-288A10.17	1.00E-06	0.032781	-15.00057224
ENSG00000165816	VWA2	1.00E-06	0.032790329	-15.00098274
ENSG00000157150	TIMP4	1.00E-06	0.0328	-15.00140819
ENSG00000256079	RP11-318G8.2	1.00E-06	0.0329	-15.00579996
ENSG00000213373	LINC00671	1.00E-06	0.032967046	-15.00873699
ENSG00000281548	LINC00895	1.00E-06	0.033061	-15.01284274
ENSG00000236911	RP11-78B10.2	1.00E-06	0.033061637	-15.01287056
ENSG00000158764	ITLN2	1.00E-06	0.033080697	-15.013702
ENSG00000256312	RP13-977J11.8	1.00E-06	0.03322	-15.01976445
ENSG00000265206	RP5-1171I10.5	1.00E-06	0.033305	-15.02345116
ENSG00000249275	RP11-364P22.2	1.00E-06	0.033325	-15.02431725
ENSG00000253875	RP11-16P20.3	1.00E-06	0.033362	-15.02591816
ENSG00000229727	AC013460.1	1.00E-06	0.033417993	-15.02833747
ENSG00000102001	CACNA1F	1.00E-06	0.033590949	-15.03578494
ENSG00000254018	RP11-785H20.1	1.00E-06	0.03366	-15.03874756
ENSG00000258308	RP11-554E23.2	1.00E-06	0.033722679	-15.04143151
ENSG00000172465	TCEAL1	1.00E-06	0.033783185	-15.04401774
ENSG00000230935	RPS3P1	1.00E-06	0.033825	-15.04580231
ENSG00000224029	RP11-136K14.2	1.00E-06	0.033878	-15.04806109
ENSG00000094796	KRT31	1.00E-06	0.03391	-15.04942316
ENSG00000105610	KLF1	1.00E-06	0.033995	-15.05303495
ENSG00000236806	RPL7AP15	1.00E-06	0.034039	-15.05490103
ENSG00000198523	PLN	1.00E-06	0.034079	-15.05659538
ENSG00000224015	AC063976.3	1.00E-06	0.034092	-15.05714562
ENSG00000124490	CRISP2	1.00E-06	0.034226559	-15.06282862
ENSG00000253299	RP11-316J7.4	1.00E-06	0.034227	-15.06284723
ENSG00000167741	GGT6	1.00E-06	0.034242268	-15.06349065
ENSG00000261496	RP13-514E23.1	1.00E-06	0.034276	-15.06491114
ENSG00000249995	ECM1P2	1.00E-06	0.034319	-15.0667199
ENSG00000183571	PGPEP1L	1.00E-06	0.034339543	-15.0675832
ENSG00000224895	VPS26BP1	1.00E-06	0.034408	-15.07045642
ENSG00000187912	CLEC17A	1.00E-06	0.034580907	-15.07768809
ENSG00000197083	ZNF300P1	1.00E-06	0.034615649	-15.07913678
ENSG00000281131	SCHLAP1	1.00E-06	0.034673	-15.08152505
ENSG00000225112	RP11-295P9.6	1.00E-06	0.034823	-15.08775288
ENSG00000111404	RERGL	1.00E-06	0.034938701	-15.09253835
ENSG00000277232	GTSE1-AS1	1.00E-06	0.034939636	-15.09257697
ENSG00000218274	RP3-407E4.3	1.00E-06	0.034962	-15.0935001
ENSG00000234682	VDAC2P4	1.00E-06	0.035015	-15.09568547
ENSG00000260514	RP11-368N21.1	1.00E-06	0.035029	-15.09626218
ENSG00000145879	SPINK7	1.00E-06	0.03502945	-15.09628071
ENSG00000271609	CTB-31N19.4	1.00E-06	0.035144	-15.10099078
ENSG00000273802	HIST1H2BG	1.00E-06	0.035281	-15.10660383

ENSG00000227496	RP11-145A3.1	1.00E-06	0.035373	-15.11036096
ENSG00000231605	LINC01363	1.00E-06	0.035424199	-15.11244759
ENSG00000241544	RP11-6F2.5	1.00E-06	0.035429	-15.11264312
ENSG00000225843	NIPA2P1	1.00E-06	0.035489	-15.1150843
ENSG00000273957	RP11-346J10.3	1.00E-06	0.035512	-15.11601899
ENSG00000236554	ASNSP3	1.00E-06	0.035638	-15.12112875
ENSG00000264359	NEK4P2	1.00E-06	0.035652	-15.12169539
ENSG00000271522	RP11-36B6.1	1.00E-06	0.035676	-15.12266625
ENSG00000120279	MYCT1	1.00E-06	0.035723	-15.12456562
ENSG00000117594	HSD11B1	1.00E-06	0.035770279	-15.12647375
ENSG00000231477	CTB-187M2.1	1.00E-06	0.035881	-15.13093248
ENSG00000218189	POM121L14P	1.00E-06	0.035895	-15.13149528
ENSG00000261327	RP11-863P13.3	1.00E-06	0.035912772	-15.13220938
ENSG00000233037	AC009237.10	1.00E-06	0.036009	-15.13606991
ENSG00000261632	RP11-390D11.1	1.00E-06	0.036035	-15.13711122
ENSG00000137975	CLCA2	1.00E-06	0.0361122	-15.14019868
ENSG00000095932	SMIM24	1.00E-06	0.036127862	-15.14082428
ENSG00000280372	bP-21201H5.2	1.00E-06	0.036201	-15.14374193
ENSG00000186026	ZNF284	1.00E-06	0.036321	-15.1485163
ENSG00000266158	RN7SL785P	1.00E-06	0.036366	-15.15030263
ENSG00000233167	EEF1A1P26	1.00E-06	0.03653	-15.15679413
ENSG00000237851	RP1-67K17.4	1.00E-06	0.036568	-15.1582941
ENSG00000258629	CTD-2014B16.1	1.00E-06	0.036593	-15.15928008
ENSG00000279796	RP11-317F20.3	1.00E-06	0.036642	-15.16121063
ENSG00000236511	LINC01231	1.00E-06	0.03665	-15.16152558
ENSG00000261837	RP11-264L1.3	1.00E-06	0.036744	-15.16522107
ENSG00000225626	C9orf135-AS1	1.00E-06	0.036767238	-15.1661332
ENSG00000228014	ZNF680P1	1.00E-06	0.036842	-15.16906376
ENSG00000254415	SIGLEC14	1.00E-06	0.036911499	-15.17178269
ENSG00000267353	CTD-2162K18.3	1.00E-06	0.03695	-15.17328674
ENSG00000253525	CTD-2114J12.1	1.00E-06	0.036968	-15.17398937
ENSG00000260756	RP11-609N14.1	1.00E-06	0.037097	-15.1790149
ENSG00000237704	AP004289.2	1.00E-06	0.037145	-15.18088041
ENSG00000141744	PNMT	1.00E-06	0.037145387	-15.18089544
ENSG00000212721	KRTAP4-11	1.00E-06	0.037151	-15.18111342
ENSG00000232458	LINC01450	1.00E-06	0.037153	-15.18119109
ENSG00000268790	CTC-429P9.4	1.00E-06	0.037158801	-15.18141632
ENSG00000214457	RPL7P29	1.00E-06	0.037171	-15.18188988
ENSG00000237539	GS1-594A7.5	1.00E-06	0.037202	-15.18309256
ENSG00000265125	RP11-31I22.3	1.00E-06	0.037291063	-15.18654229
ENSG00000125975	C20orf173	1.00E-06	0.037324028	-15.18781705
ENSG00000232800	SLC7A15P	1.00E-06	0.037325	-15.18785464
ENSG00000250511	RP11-380D23.1	1.00E-06	0.037402	-15.1908278
ENSG00000253679	KB-1410C5.2	1.00E-06	0.037591	-15.19809967
ENSG00000186895	FGF3	1.00E-06	0.037617	-15.19909718
ENSG00000221932	HEPN1	1.00E-06	0.037741	-15.20384503
ENSG00000228286	AP000593.5	1.00E-06	0.037767	-15.20483857
ENSG00000260477	LINC01584	1.00E-06	0.03792613	-15.21090456

ENSG00000236304	AP001189.4	1.00E-06	0.037953	-15.21192631
ENSG00000277467	RN7SL628P	1.00E-06	0.03806	-15.21598794
ENSG00000278696	RN7SL82P	1.00E-06	0.03806	-15.21598794
ENSG00000257037	RARSP1	1.00E-06	0.038167	-15.22003817
ENSG00000120329	SLC25A2	1.00E-06	0.038194	-15.2210584
ENSG00000261863	RP11-141J13.5	1.00E-06	0.038263	-15.22366237
ENSG00000261105	LMO7-AS1	1.00E-06	0.038330939	-15.22622173
ENSG00000254278	RP11-278I4.2	1.00E-06	0.038437	-15.23020812
ENSG00000170788	DYDC1	1.00E-06	0.038501617	-15.23263141
ENSG00000265315	RN7SL199P	1.00E-06	0.03852	-15.23332008
ENSG00000259019	EIF4BP1	1.00E-06	0.038548	-15.23436839
ENSG00000145708	CRHBP	1.00E-06	0.038616004	-15.23691127
ENSG00000238008	COX6CP10	1.00E-06	0.038657	-15.23844206
ENSG00000238649	SNORD42A	1.00E-06	0.038692	-15.23974768
ENSG00000259810	AC002519.8	1.00E-06	0.038769	-15.2426159
ENSG00000219867	RP11-40G16.1	1.00E-06	0.038789	-15.24335996
ENSG00000246523	RP11-736K20.6	1.00E-06	0.038851541	-15.24568419
ENSG00000272797	RP11-368I23.3	1.00E-06	0.038918	-15.24814995
ENSG00000156222	SLC28A1	1.00E-06	0.038961317	-15.24975482
ENSG00000224525	RP11-561I11.3	1.00E-06	0.039074	-15.25392133
ENSG00000254381	TUBB8P5	1.00E-06	0.039258	-15.26069906
ENSG00000165621	OXGR1	1.00E-06	0.039280688	-15.26153259
ENSG00000233671	AC083899.1	1.00E-06	0.039332	-15.26341593
ENSG00000254980	RP11-794P6.6	1.00E-06	0.039361	-15.26447925
ENSG00000265261	RP11-162A12.3	1.00E-06	0.039403	-15.26601785
ENSG00000228408	RP1-111D6.3	1.00E-06	0.039475029	-15.26865271
ENSG00000262322	MTND4P34	1.00E-06	0.039476	-15.26868819
ENSG00000280427	AC104057.1	1.00E-06	0.039692	-15.27656064
ENSG00000102109	PCSK1N	1.00E-06	0.03970664	-15.27709266
ENSG00000280410	AC104073.2	1.00E-06	0.039736	-15.27815903
ENSG00000148377	IDI2	1.00E-06	0.039824	-15.28135051
ENSG00000263551	RP11-78F17.1	1.00E-06	0.039912219	-15.28454287
ENSG00000189152	GRAPL	1.00E-06	0.040029444	-15.28877395
ENSG00000260971	RP11-504A18.1	1.00E-06	0.04006	-15.2898748
ENSG00000279067	GS1-25M2.1	1.00E-06	0.040118	-15.29196206
ENSG00000235382	MTND4P31	1.00E-06	0.040298	-15.29842062
ENSG00000232018	AL132709.8	1.00E-06	0.040328227	-15.29950234
ENSG00000259582	RP11-461F11.3	1.00E-06	0.040506408	-15.30586255
ENSG00000225398	PGM5P4	1.00E-06	0.04054	-15.30705847
ENSG00000235746	CTBP2P2	1.00E-06	0.04057	-15.30812568
ENSG00000257345	RP11-511B23.1	1.00E-06	0.040666264	-15.31154483
ENSG00000234389	AC007278.3	1.00E-06	0.040784	-15.31571566
ENSG00000133661	SFTPD	1.00E-06	0.040969696	-15.32226958
ENSG00000188822	CNR2	1.00E-06	0.041203	-15.33046176
ENSG00000279714	CTA-212A2.2	1.00E-06	0.041234	-15.3315468
ENSG00000105550	FGF21	1.00E-06	0.041251	-15.33214147
ENSG00000167346	MMP26	1.00E-06	0.041376836	-15.33653572
ENSG00000227745	AC098826.5	1.00E-06	0.041567	-15.34315101

ENSG00000251518	CTD-2130F23.2	1.00E-06	0.041632	-15.34540525
ENSG00000000938	FGR	1.00E-06	0.041751186	-15.34952957
ENSG00000245479	LINC01585	1.00E-06	0.041792	-15.35093918
ENSG00000229877	PAICSP5	1.00E-06	0.041803	-15.35131886
ENSG00000254536	RP11-108K14.8	1.00E-06	0.04183	-15.35225038
ENSG00000257859	CASC18	1.00E-06	0.041856045	-15.35314837
ENSG00000226308	RP4-813D12.3	1.00E-06	0.041856965	-15.35318009
ENSG00000104313	EYA1	1.00E-06	0.041872516	-15.35371598
ENSG00000214930	KRT8P6	1.00E-06	0.042149	-15.36321078
ENSG00000224683	RPL36AP29	1.00E-06	0.04215	-15.36324501
ENSG00000236745	YRDCP2	1.00E-06	0.04215	-15.36324501
ENSG00000205625	RP11-15G8.1	1.00E-06	0.042245	-15.36649298
ENSG00000181963	OR52K2	1.00E-06	0.042268	-15.36727823
ENSG00000203411	Metazoa_SRP	1.00E-06	0.042376	-15.37095979
ENSG00000268292	AC006547.15	1.00E-06	0.042485	-15.37466594
ENSG00000185966	LCE3E	1.00E-06	0.042517	-15.37575218
ENSG00000265794	RP11-227G15.3	1.00E-06	0.042528232	-15.37613325
ENSG00000125492	BARHL1	1.00E-06	0.042538	-15.37646458
ENSG00000215606	KRT18P35	1.00E-06	0.042648	-15.38019047
ENSG00000185888	PRSS38	1.00E-06	0.042715	-15.38245516
ENSG00000230225	MTND5P14	1.00E-06	0.042715	-15.38245516
ENSG00000255210	RP11-344L21.1	1.00E-06	0.042749	-15.38360305
ENSG00000213849	AC104306.1	1.00E-06	0.042791	-15.38501977
ENSG00000279946	CH17-76K2.6	1.00E-06	0.042813	-15.38576131
ENSG00000119938	PPP1R3C	1.00E-06	0.042885	-15.3881855
ENSG00000186038	HTR3E	1.00E-06	0.042902083	-15.38876008
ENSG00000226807	MROH5	1.00E-06	0.042941792	-15.39009478
ENSG00000224342	AC007879.1	1.00E-06	0.042984	-15.39151212
ENSG00000280265	CTA-363E6.3	1.00E-06	0.04309	-15.39506548
ENSG00000107165	TYRP1	1.00E-06	0.043111271	-15.39577746
ENSG00000243468	INGX	1.00E-06	0.043400314	-15.40541787
ENSG00000226690	AC005281.1	1.00E-06	0.043575024	-15.41121384
ENSG00000094661	OR1I1	1.00E-06	0.043576	-15.41124615
ENSG00000198443	KRTAP4-1	1.00E-06	0.04361	-15.41237137
ENSG00000237887	RPL23AP32	1.00E-06	0.043627	-15.41293365
ENSG00000105467	SYNGR4	1.00E-06	0.043645968	-15.41356076
ENSG00000226056	MTND4P32	1.00E-06	0.043751	-15.41702837
ENSG00000257198	FAM205BP	1.00E-06	0.043908917	-15.42222633
ENSG00000237159	CNTFR-AS1	1.00E-06	0.043956281	-15.42378171
ENSG00000259055	RP11-1140I5.1	1.00E-06	0.044024	-15.42600261
ENSG00000251665	RP11-700H6.2	1.00E-06	0.044036446	-15.42641042
ENSG00000219712	RP11-532F6.2	1.00E-06	0.044108	-15.42875272
ENSG00000267311	RP11-99A1.2	1.00E-06	0.04428	-15.4343676
ENSG00000237651	C2orf74	1.00E-06	0.044296746	-15.4349131
ENSG00000235347	RP1-37M3.8	1.00E-06	0.044397	-15.43817457
ENSG00000188408	MAGEB5	1.00E-06	0.044507	-15.44174464
ENSG00000188771	PLET1	1.00E-06	0.044543	-15.44291111
ENSG00000200597	RNU1-87P	1.00E-06	0.044569	-15.44375297

ENSG00000129244	ATP1B2	1.00E-06	0.044605186	-15.44492384
ENSG00000139209	SLC38A4	1.00E-06	0.044653687	-15.44649168
ENSG00000197838	CYP2A13	1.00E-06	0.044919	-15.45503819
ENSG00000143452	HORMAD1	1.00E-06	0.045006649	-15.45785052
ENSG00000257639	CTB-31N19.2	1.00E-06	0.045101	-15.4608718
ENSG00000260133	CA5AP1	1.00E-06	0.045101	-15.4608718
ENSG00000236525	AC007278.2	1.00E-06	0.045101	-15.4608718
ENSG00000165078	CPA6	1.00E-06	0.045195505	-15.46389167
ENSG00000188133	TMEM215	1.00E-06	0.04534	-15.46849677
ENSG00000279927	CTA-212A2.4	1.00E-06	0.045378	-15.4697054
ENSG00000228755	PABPC1P8	1.00E-06	0.045403	-15.47050001
ENSG00000235180	LINC00601	1.00E-06	0.045409	-15.47069065
ENSG00000234465	PINLYP	1.00E-06	0.04547969	-15.47293479
ENSG00000258546	CENPUP2	1.00E-06	0.045633	-15.47778988
ENSG00000255028	RP11-708B6.2	1.00E-06	0.045637672	-15.47793758
ENSG00000274393	AL583842.1	1.00E-06	0.045644	-15.47813761
ENSG00000278721	AC138775.5	1.00E-06	0.045644	-15.47813761
ENSG00000153498	SPACA7	1.00E-06	0.045667217	-15.47887126
ENSG00000244378	RPS2P45	1.00E-06	0.045709181	-15.48019634
ENSG00000214329	SLC9B1P2	1.00E-06	0.04571	-15.4802222
ENSG00000259192	AC109631.1	1.00E-06	0.045736	-15.48104257
ENSG00000186431	FCAR	1.00E-06	0.045747546	-15.48140675
ENSG00000099769	IGFALS	1.00E-06	0.045806036	-15.48325011
ENSG00000204555	CFTRP3	1.00E-06	0.045952	-15.48784003
ENSG00000259347	RP11-798K3.2	1.00E-06	0.04598165	-15.48877061
ENSG00000237413	MGC27382	1.00E-06	0.046026973	-15.49019196
ENSG00000212989	RP11-351E7.2	1.00E-06	0.046106	-15.49266689
ENSG00000276651	AC007950.2	1.00E-06	0.046119	-15.49307361
ENSG00000226455	RP3-512E2.2	1.00E-06	0.046306353	-15.49892252
ENSG00000250878	METTL21EP	1.00E-06	0.046317166	-15.49925935
ENSG00000268931	RP11-886P16.6	1.00E-06	0.046601	-15.50807329
ENSG00000118094	TREH	1.00E-06	0.046602851	-15.50813061
ENSG00000239600	AP000797.2	1.00E-06	0.046615	-15.50850665
ENSG00000259289	RP11-798K3.3	1.00E-06	0.046668	-15.51014602
ENSG00000183145	RIPPLY3	1.00E-06	0.046675744	-15.51038539
ENSG00000267927	CTD-2525I3.2	1.00E-06	0.046818	-15.51477569
ENSG00000279496	CTC-251I16.1	1.00E-06	0.046899	-15.51726954
ENSG00000183535	COL18A1-AS1	1.00E-06	0.046993379	-15.52016989
ENSG00000261644	RP11-327F22.2	1.00E-06	0.047061	-15.52224436
ENSG00000265408	RP11-361L15.4	1.00E-06	0.047346	-15.53095493
ENSG00000272209	RP3-500L14.2	1.00E-06	0.04735	-15.53107681
ENSG00000128606	LRRC17	1.00E-06	0.047360862	-15.53140772
ENSG00000205682	RP11-798L4.1	1.00E-06	0.047378	-15.53192968
ENSG00000269590	CTD-2192J16.24	1.00E-06	0.047446	-15.53399884
ENSG00000279687	CH507-9B2.8	1.00E-06	0.047454	-15.53424208
ENSG00000204542	C6orf15	1.00E-06	0.047475	-15.53488038
ENSG00000174697	LEP	1.00E-06	0.047475	-15.53488038
ENSG00000147896	IFNK	1.00E-06	0.047475	-15.53488038

ENSG00000220377	GSTA8P	1.00E-06	0.047578	-15.53800701
ENSG00000232373	MTCYBP3	1.00E-06	0.047599	-15.53864364
ENSG00000254997	KRTAP5-9	1.00E-06	0.047641	-15.53991608
ENSG00000131885	KRT17P1	1.00E-06	0.0476826	-15.54117528
ENSG00000230162	CT45A11P	1.00E-06	0.047726	-15.54248781
ENSG00000261501	RP11-63B13.1	1.00E-06	0.047768	-15.54375685
ENSG00000236790	LINC00299	1.00E-06	0.047774921	-15.54396587
ENSG00000248896	CTD-2135J3.3	1.00E-06	0.047800345	-15.54473342
ENSG00000273979	Metazoa_SRP	1.00E-06	0.047929	-15.54861122
ENSG00000225366	TDGF1P3	1.00E-06	0.047929	-15.54861122
ENSG00000239774	RP11-496B10.3	1.00E-06	0.048064	-15.5526691
ENSG00000163631	ALB	1.00E-06	0.048079201	-15.55312529
ENSG00000233716	AC074367.1	1.00E-06	0.048107	-15.55395921
ENSG00000183514	TDGF1P2	1.00E-06	0.04815	-15.55524818
ENSG00000221063	MIR1296	1.00E-06	0.048322	-15.56039255
ENSG00000249429	CTD-2050E21.1	1.00E-06	0.048322	-15.56039255
ENSG00000258325	RP4-816N1.6	1.00E-06	0.048360218	-15.56153313
ENSG00000171819	ANGPTL7	1.00E-06	0.048365	-15.56167578
ENSG00000236325	AC005300.5	1.00E-06	0.048365	-15.56167578
ENSG00000204246	OR13C3	1.00E-06	0.048845	-15.57592327
ENSG00000260338	LINC01570	1.00E-06	0.0488891	-15.57722523
ENSG00000251544	MTND5P12	1.00E-06	0.048929	-15.57840218
ENSG00000258851	RP11-894P9.2	1.00E-06	0.048934	-15.5785496
ENSG00000171402	XAGE3	1.00E-06	0.049022027	-15.58114252
ENSG00000229494	AC012494.1	1.00E-06	0.049067	-15.58246545
ENSG00000280432	AP000962.1	1.00E-06	0.049156	-15.5850799
ENSG00000240915	RP11-659E9.2	1.00E-06	0.049201	-15.58640002
ENSG00000224413	AP001476.2	1.00E-06	0.049245	-15.58768963
ENSG00000255020	AF131216.5	1.00E-06	0.049245	-15.58768963
ENSG00000248125	CTB-73N10.1	1.00E-06	0.049245783	-15.58771258
ENSG00000236213	AC006369.2	1.00E-06	0.04938	-15.59163922
ENSG00000095970	TREM2	1.00E-06	0.049425534	-15.59296894
ENSG00000250582	SMAD1-AS2	1.00E-06	0.049652	-15.59956421
ENSG00000228317	RP11-235C23.5	1.00E-06	0.049747	-15.60232191
ENSG00000261448	CTD-2576D5.4	1.00E-06	0.049765	-15.60284382
ENSG00000257517	RP4-601P9.1	1.00E-06	0.049766	-15.60287281
ENSG00000215533	LINC00189	1.00E-06	0.049788462	-15.60352382
ENSG00000257680	RP11-367O10.1	1.00E-06	0.049789	-15.60353942
ENSG00000281000	SNORD3D	1.00E-06	0.049881	-15.60620277
ENSG00000223609	HBD	1.00E-06	0.049941375	-15.60794792
ENSG00000262636	CTD-3088G3.4	1.00E-06	0.049958	-15.6084281
ENSG00000203363	AC012454.4	1.00E-06	0.050041	-15.610823
ENSG00000226226	PRELID3BP1	1.00E-06	0.050111	-15.61283971
ENSG00000270846	RP11-674I16.1	1.00E-06	0.050111	-15.61283971
ENSG00000237835	AC007064.22	1.00E-06	0.050251	-15.61686469
ENSG00000264617	AC144838.3	1.00E-06	0.050297	-15.61818473
ENSG00000263438	RP11-202D1.3	1.00E-06	0.050297561	-15.61820083
ENSG00000269948	RP11-248J23.6	1.00E-06	0.050321778	-15.61889528

ENSG00000243193	CTA-360L10.1	1.00E-06	0.050408026	-15.62136584
ENSG00000212297	RNU6-821P	1.00E-06	0.05058	-15.62627942
ENSG00000271840	RP1-224A6.9	1.00E-06	0.050647	-15.6281892
ENSG00000212190	RNU6-298P	1.00E-06	0.050738	-15.63077903
ENSG00000242973	RP11-446F17.3	1.00E-06	0.050826275	-15.63328687
ENSG00000236592	S100A11P2	1.00E-06	0.050883	-15.63489611
ENSG00000168081	PNOC	1.00E-06	0.050913076	-15.63574862
ENSG00000249396	RP11-1C1.4	1.00E-06	0.051071	-15.64021669
ENSG00000274076	RN7SL539P	1.00E-06	0.051083	-15.64055563
ENSG00000172362	OR5B12	1.00E-06	0.051347	-15.64799237
ENSG00000240751	RP11-553D4.2	1.00E-06	0.051396	-15.64936846
ENSG00000200656	SNORA5B	1.00E-06	0.05146	-15.65116384
ENSG00000226395	RP11-218D6.3	1.00E-06	0.051543	-15.65348889
ENSG00000278961	CH507-396I9.3	1.00E-06	0.051543	-15.65348889
ENSG00000243627	AP000322.53	1.00E-06	0.051543	-15.65348889
ENSG00000156755	IGKV1OR-2	1.00E-06	0.051543	-15.65348889
ENSG00000266049	RP11-703M24.5	1.00E-06	0.05159299	-15.65488745
ENSG00000179082	C9orf106	1.00E-06	0.051626	-15.6558102
ENSG00000236782	RP11-96L14.7	1.00E-06	0.051672576	-15.6571112
ENSG00000227205	PFN1P9	1.00E-06	0.05187	-15.66261275
ENSG00000223715	LINC01208	1.00E-06	0.05203818	-15.66728288
ENSG00000267593	RP11-108P20.4	1.00E-06	0.052082	-15.66849723
ENSG00000253725	RP11-21I4.2	1.00E-06	0.05214	-15.67010296
ENSG00000253646	CTD-2311A18.1	1.00E-06	0.052149	-15.67035197
ENSG00000237065	NANOGP4	1.00E-06	0.052165	-15.67079454
ENSG00000248747	RP11-586L23.1	1.00E-06	0.05219	-15.67148578
ENSG00000275875	RP11-613E4.5	1.00E-06	0.05229	-15.67424745
ENSG00000226430	USP17L7	1.00E-06	0.052305	-15.67466124
ENSG00000232869	TRBV29-1	1.00E-06	0.052504	-15.68013972
ENSG00000279548	CTA-243E7.3	1.00E-06	0.052569	-15.68192467
ENSG00000260349	RP11-473I1.5	1.00E-06	0.052606	-15.68293974
ENSG00000267334	CTD-2534I21.8	1.00E-06	0.052646	-15.6840363
ENSG00000187229	RP11-161I2.1	1.00E-06	0.052697	-15.68543321
ENSG00000279493	CH507-9B2.2	1.00E-06	0.052749	-15.68685612
ENSG00000174156	GSTA3	1.00E-06	0.0528	-15.68825031
ENSG00000100652	SLC10A1	1.00E-06	0.052826	-15.68896055
ENSG00000227158	AC073621.2	1.00E-06	0.052852	-15.68967045
ENSG00000202071	Y_RNA	1.00E-06	0.05306	-15.69533706
ENSG00000229658	PABPC1P9	1.00E-06	0.053158	-15.69799921
ENSG00000282535	AC092192.1	1.00E-06	0.053164	-15.69816204
ENSG00000203908	KHDC3L	1.00E-06	0.053164	-15.69816204
ENSG00000226926	PDZPH1P	1.00E-06	0.053215625	-15.69956228
ENSG00000235241	RP11-108M9.5	1.00E-06	0.053269	-15.70100858
ENSG00000260410	RP11-505K9.3	1.00E-06	0.053321	-15.70241622
ENSG00000276950	GSTTP1	1.00E-06	0.053372858	-15.70381865
ENSG00000272027	RP11-529E15.1	1.00E-06	0.053408262	-15.70477532
ENSG00000157093	LYZL4	1.00E-06	0.053426132	-15.70525796
ENSG00000249948	GBA3	1.00E-06	0.053530216	-15.70806584

ENSG00000226991	AC112229.6	1.00E-06	0.053532	-15.70811393
ENSG00000223812	RP11-197K6.1	1.00E-06	0.053637628	-15.71095783
ENSG00000279446	AC005915.1	1.00E-06	0.053638	-15.71096782
ENSG00000187747	OR52B6	1.00E-06	0.053691	-15.71239266
ENSG00000206073	SERPINB4	1.00E-06	0.053771541	-15.7145552
ENSG00000253298	AC008703.1	1.00E-06	0.053798	-15.71526492
ENSG00000224354	MTND2P5	1.00E-06	0.053798	-15.71526492
ENSG00000235271	LINC01422	1.00E-06	0.053829845	-15.71611866
ENSG00000260660	RP11-69H7.2	1.00E-06	0.053871942	-15.71724645
ENSG00000236274	RP4-728D4.3	1.00E-06	0.053958	-15.71954925
ENSG00000254140	RP11-731F5.1	1.00E-06	0.053958938	-15.71957434
ENSG00000263862	LINC01543	1.00E-06	0.054121	-15.72390088
ENSG00000251249	RP11-73G16.1	1.00E-06	0.054174422	-15.72532425
ENSG00000249425	RP11-502M1.2	1.00E-06	0.054235	-15.72693656
ENSG00000227091	RP5-1028L10.1	1.00E-06	0.054283134	-15.72821641
ENSG00000170782	OR10A4	1.00E-06	0.054392	-15.73110685
ENSG00000280094	OR1B1	1.00E-06	0.054392	-15.73110685
ENSG00000254141	RP11-642D21.1	1.00E-06	0.054423	-15.73192887
ENSG00000269021	CTD-3187F8.12	1.00E-06	0.054454	-15.73275041
ENSG00000249699	RP11-415C15.2	1.00E-06	0.054468	-15.73312127
ENSG00000186103	ARGFX	1.00E-06	0.054557	-15.73547669
ENSG00000231121	RP1-34H18.1	1.00E-06	0.054612	-15.73693037
ENSG00000253645	CTD-2544N14.3	1.00E-06	0.054624	-15.73724734
ENSG00000269635	AC004257.1	1.00E-06	0.054707	-15.73943782
ENSG00000274186	RN7SL248P	1.00E-06	0.054834	-15.7427831
ENSG00000276544	RN7SL453P	1.00E-06	0.054834	-15.7427831
ENSG00000273946	RN7SL733P	1.00E-06	0.054834	-15.7427831
ENSG00000249833	CCDC37-AS1	1.00E-06	0.054889	-15.74422944
ENSG00000280707	RP11-568A7.4	1.00E-06	0.054945027	-15.7457013
ENSG00000216378	RP3-344J20.1	1.00E-06	0.055001	-15.74717023
ENSG00000255394	C8orf49	1.00E-06	0.055001	-15.74717023
ENSG00000266588	RP1-56K13.5	1.00E-06	0.055056134	-15.7486157
ENSG00000151631	AKR1C6P	1.00E-06	0.055057	-15.74863838
ENSG00000234382	RP11-40F6.1	1.00E-06	0.055067	-15.74890039
ENSG00000122852	SFTPA1	1.00E-06	0.055202531	-15.7524468
ENSG00000214976	VDAC2P1	1.00E-06	0.055363	-15.7566345
ENSG00000163982	OTOP1	1.00E-06	0.055378	-15.75702533
ENSG00000233521	RP5-1172A22.1	1.00E-06	0.055508507	-15.76042126
ENSG00000213435	ATP6V0CP3	1.00E-06	0.055627	-15.76349768
ENSG00000213954	ATP5HP3	1.00E-06	0.055679	-15.76484568
ENSG00000231725	VN1R110P	1.00E-06	0.055737	-15.76634773
ENSG00000207712	MIR627	1.00E-06	0.055794	-15.76782236
ENSG00000203523	TAS2R2P	1.00E-06	0.055904	-15.77066389
ENSG00000256686	RP11-443N24.2	1.00E-06	0.056083	-15.7752759
ENSG00000215131	C16orf90	1.00E-06	0.056199194	-15.77826182
ENSG00000204049	RP11-126H7.4	1.00E-06	0.056317	-15.78128286
ENSG00000279633	RP11-442G21.2	1.00E-06	0.056317	-15.78128286
ENSG00000217786	RP3-508D13.1	1.00E-06	0.056375	-15.78276791

ENSG00000228429	RP5-859M6.1	1.00E-06	0.05639	-15.78315172
ENSG00000244062	RP11-404G16.2	1.00E-06	0.056464	-15.78504371
ENSG00000280551	RP11-358D17.2	1.00E-06	0.056553	-15.78731594
ENSG00000226770	AC000124.1	1.00E-06	0.056553	-15.78731594
ENSG00000232791	OR11I1P	1.00E-06	0.05673	-15.79182424
ENSG00000227261	YWHAZP7	1.00E-06	0.056951	-15.79743355
ENSG00000258590	NBEAP1	1.00E-06	0.057021641	-15.79922195
ENSG00000282742	RP11-539L10.5	1.00E-06	0.057029	-15.79940811
ENSG00000263941	RN7SL32P	1.00E-06	0.057046	-15.79983811
ENSG00000259967	RP11-2E17.2	1.00E-06	0.05706	-15.80019213
ENSG00000226846	LINC00348	1.00E-06	0.05709	-15.80095044
ENSG00000248659	RP11-263I1.1	1.00E-06	0.05709	-15.80095044
ENSG00000227742	CALR4P	1.00E-06	0.05721	-15.80397972
ENSG00000172769	OR5B3	1.00E-06	0.05721	-15.80397972
ENSG00000258010	RP11-210M15.1	1.00E-06	0.05724	-15.80473605
ENSG00000172377	OR9I1	1.00E-06	0.05727	-15.80549198
ENSG00000213358	AC092933.4	1.00E-06	0.05727	-15.80549198
ENSG00000165202	OR1Q1	1.00E-06	0.05727	-15.80549198
ENSG00000223730	RP1-127B20.4	1.00E-06	0.057338621	-15.80721958
ENSG00000282964	RP11-438H24.2	1.00E-06	0.057385	-15.80838606
ENSG00000279906	AP002962.1	1.00E-06	0.057453	-15.81009461
ENSG00000280382	RP11-804H8.7	1.00E-06	0.057473	-15.81059674
ENSG00000226798	RP11-289F5.1	1.00E-06	0.057473	-15.81059674
ENSG00000265279	CLUHP6	1.00E-06	0.057514	-15.81162556
ENSG00000280842	AL121989.1	1.00E-06	0.057575	-15.81315489
ENSG00000237107	RP11-256G5.1	1.00E-06	0.057636	-15.81468259
ENSG00000176716	OR10AB1P	1.00E-06	0.057698	-15.81623369
ENSG00000232282	MTND1P32	1.00E-06	0.057698	-15.81623369
ENSG00000250039	RP11-17E2.2	1.00E-06	0.05776	-15.81778312
ENSG00000220349	RP3-431A14.4	1.00E-06	0.05776	-15.81778312
ENSG00000259062	ACTN1-AS1	1.00E-06	0.057820779	-15.81930042
ENSG00000227776	AKR1D1P1	1.00E-06	0.057821	-15.81930594
ENSG00000253245	MTND1P36	1.00E-06	0.057821	-15.81930594
ENSG00000257156	RP11-13A1.3	1.00E-06	0.057825	-15.81940574
ENSG00000173124	ACSM6	1.00E-06	0.057841568	-15.81981905
ENSG00000232090	AC016724.6	1.00E-06	0.057848	-15.81997946
ENSG00000258913	RP11-260M19.2	1.00E-06	0.058063889	-15.82535358
ENSG00000142484	TM4SF5	1.00E-06	0.058069	-15.82548057
ENSG00000252118	RNU6ATAC39P	1.00E-06	0.058116	-15.82664779
ENSG00000261158	CTD-2583P5.3	1.00E-06	0.058382	-15.83323601
ENSG00000231082	RP11-514F8.2	1.00E-06	0.058509	-15.83637094
ENSG00000250804	RP11-747H12.3	1.00E-06	0.058509	-15.83637094
ENSG00000255713	OR4D2	1.00E-06	0.058572	-15.83792354
ENSG00000225380	IGKV1OR9-2	1.00E-06	0.058615	-15.83898229
ENSG00000253474	RP11-10H3.1	1.00E-06	0.058699396	-15.84105804
ENSG00000251623	RP11-826N14.4	1.00E-06	0.058827	-15.84419084
ENSG00000274515	CTD-2026K11.5	1.00E-06	0.058855	-15.84487736
ENSG00000267227	RP11-640I15.2	1.00E-06	0.058858	-15.8449509

ENSG00000215450	RP5-890O15.3	1.00E-06	0.058949	-15.84717972
ENSG00000239924	RPL29P22	1.00E-06	0.058955	-15.84732655
ENSG00000266978	CTD-2369P2.5	1.00E-06	0.058955	-15.84732655
ENSG00000259133	RP11-1085N6.3	1.00E-06	0.058986458	-15.84809616
ENSG00000231957	GNAI2P2	1.00E-06	0.059083	-15.85045546
ENSG00000226206	RP5-1050E16.2	1.00E-06	0.059083	-15.85045546
ENSG00000227660	UST-AS1	1.00E-06	0.059123	-15.85143186
ENSG00000250102	RP11-314N14.1	1.00E-06	0.05921291	-15.85362414
ENSG00000236853	OR2R1P	1.00E-06	0.059213	-15.85362633
ENSG00000229005	HNF4A-AS1	1.00E-06	0.059342447	-15.85677681
ENSG00000253733	LZTS1-AS1	1.00E-06	0.05946	-15.85963184
ENSG00000235422	KATNBL1P3	1.00E-06	0.059539	-15.86154737
ENSG00000269931	RP5-1069C8.3	1.00E-06	0.05967	-15.86471816
ENSG00000234315	OSTCP5	1.00E-06	0.059736	-15.86631302
ENSG00000229941	AC012499.1	1.00E-06	0.059891	-15.8700516
ENSG00000232353	RP11-655G22.1	1.00E-06	0.059934512	-15.87109936
ENSG00000265893	AL132772.1	1.00E-06	0.060134	-15.87589331
ENSG00000250384	UBE2CP3	1.00E-06	0.060134	-15.87589331
ENSG00000262884	CTD-3060P21.1	1.00E-06	0.060165	-15.87663685
ENSG00000233878	AC073133.1	1.00E-06	0.060179	-15.87697251
ENSG00000259804	CTD-2012K14.7	1.00E-06	0.0602	-15.87747587
ENSG00000179799	OR7E22P	1.00E-06	0.060256	-15.87881728
ENSG00000254887	CTC-378H22.1	1.00E-06	0.060335	-15.88070752
ENSG00000121335	PRB2	1.00E-06	0.060368675	-15.88151251
ENSG00000224709	OR11M1P	1.00E-06	0.06047	-15.88393196
ENSG00000229047	AF127577.10	1.00E-06	0.060562	-15.88612523
ENSG00000111405	ENDOU	1.00E-06	0.060605121	-15.88715207
ENSG00000206743	RNU6-484P	1.00E-06	0.060696	-15.88931382
ENSG00000227290	LINC01364	1.00E-06	0.060878	-15.89363334
ENSG00000111701	APOBEC1	1.00E-06	0.060878413	-15.89364313
ENSG00000268994	RP1-172N19.1	1.00E-06	0.06108	-15.89841244
ENSG00000254001	RP11-91P17.1	1.00E-06	0.061180133	-15.90077563
ENSG00000257277	RP11-434H14.1	1.00E-06	0.061222672	-15.90177839
ENSG00000275386	CTC-788C1.2	1.00E-06	0.061269	-15.90286968
ENSG00000229672	RP11-184A2.3	1.00E-06	0.061322	-15.90411713
ENSG00000227254	VDAC1P12	1.00E-06	0.061361	-15.90503437
ENSG00000259009	TPRX2P	1.00E-06	0.061431	-15.90667925
ENSG00000223322	AL033378.1	1.00E-06	0.0615	-15.90829879
ENSG00000280175	AL354828.2	1.00E-06	0.061626	-15.91125153
ENSG00000240031	OR9A3P	1.00E-06	0.061640354	-15.91158753
ENSG00000241963	RN7SL655P	1.00E-06	0.061748	-15.91410479
ENSG00000267170	CALM2P1	1.00E-06	0.061782	-15.91489895
ENSG00000228166	MTND1P11	1.00E-06	0.061782	-15.91489895
ENSG00000226774	RP11-654E17.2	1.00E-06	0.061782	-15.91489895
ENSG00000260681	CTA-363E6.5	1.00E-06	0.061852	-15.91653263
ENSG00000281674	AC087651.2	1.00E-06	0.061852	-15.91653263
ENSG00000276308	RP11-116D17.4	1.00E-06	0.061888	-15.91737208
ENSG00000259782	CTD-2270L9.2	1.00E-06	0.062074	-15.92170149

ENSG00000219582	HNRNPA1P58	1.00E-06	0.062105	-15.9224218
ENSG00000258131	RP11-541G9.1	1.00E-06	0.062338	-15.92782425
ENSG00000258670	RP11-1042B17.3	1.00E-06	0.062351	-15.92812508
ENSG00000224999	VTA1P1	1.00E-06	0.062495	-15.93145315
ENSG00000214428	NPM1P10	1.00E-06	0.062567	-15.93311431
ENSG00000227474	RP11-295P9.2	1.00E-06	0.062639	-15.93477356
ENSG00000266578	RP11-838N2.5	1.00E-06	0.062639	-15.93477356
ENSG00000274913	AC107977.1	1.00E-06	0.062748	-15.93728186
ENSG00000280062	LA16c-380F5.1	1.00E-06	0.06276	-15.93755773
ENSG00000257878	RP11-256L6.3	1.00E-06	0.062785	-15.9381323
ENSG00000203531	AC016712.1	1.00E-06	0.062785	-15.9381323
ENSG00000233332	RP4-799P18.2	1.00E-06	0.062821	-15.93895929
ENSG00000270164	LINC01480	1.00E-06	0.062857649	-15.9398007
ENSG00000163440	PDCL2	1.00E-06	0.062858	-15.93980875
ENSG00000275186	RP11-196B3.3	1.00E-06	0.062887	-15.94047419
ENSG00000272384	RP11-44N11.2	1.00E-06	0.062931	-15.94148325
ENSG00000277651	CTD-2014B16.5	1.00E-06	0.063009	-15.94327029
ENSG00000261706	LINC00165	1.00E-06	0.063053	-15.94427739
ENSG00000277997	RP11-356M6.1	1.00E-06	0.063206	-15.9477739
ENSG00000233419	RP4-781L3.1	1.00E-06	0.063276	-15.94937078
ENSG00000262543	RP3-422G23.4	1.00E-06	0.063299	-15.94989509
ENSG00000250362	AC008592.5	1.00E-06	0.063670967	-15.95834806
ENSG00000242295	RP11-522B15.1	1.00E-06	0.063672	-15.95837146
ENSG00000165623	UCMA	1.00E-06	0.063978632	-15.96530253
ENSG00000245293	RP11-286E11.1	1.00E-06	0.064098732	-15.96800821
ENSG00000234199	LINC01191	1.00E-06	0.064124	-15.9685768
ENSG00000188257	PLA2G2A	1.00E-06	0.064238148	-15.97114269
ENSG00000254826	CTD-2530H12.2	1.00E-06	0.064276	-15.97199253
ENSG00000217331	RP11-304C16.3	1.00E-06	0.064353	-15.97371978
ENSG00000260089	ADAM3B	1.00E-06	0.064429	-15.97542258
ENSG00000232164	AC092669.3	1.00E-06	0.064467	-15.97627323
ENSG00000257346	RP11-386G11.8	1.00E-06	0.064506072	-15.97714734
ENSG00000249743	RP11-60A8.1	1.00E-06	0.064671017	-15.98083169
ENSG00000226406	RBMX2P1	1.00E-06	0.064738	-15.98232518
ENSG00000236504	AC087499.7	1.00E-06	0.064815	-15.98404011
ENSG00000274937	CTD-2311M21.4	1.00E-06	0.064893	-15.98577524
ENSG00000235548	AC073551.1	1.00E-06	0.064893	-15.98577524
ENSG00000234683	RP5-837I24.5	1.00E-06	0.064945	-15.98693084
ENSG00000249510	KB-1247B1.1	1.00E-06	0.064971	-15.98750829
ENSG00000269918	AF131215.9	1.00E-06	0.06503	-15.9888178
ENSG00000235101	SETP9	1.00E-06	0.065049	-15.98923926
ENSG00000254761	RP11-672A2.1	1.00E-06	0.065049	-15.98923926
ENSG00000213014	VN2R17P	1.00E-06	0.065049	-15.98923926
ENSG00000229088	MTND1P10	1.00E-06	0.065049	-15.98923926
ENSG00000278001	RP11-157L3.11	1.00E-06	0.065206	-15.9927171
ENSG00000249646	OR7E94P	1.00E-06	0.065284113	-15.99444434
ENSG00000240152	RP11-16N2.1	1.00E-06	0.065284212	-15.99444653
ENSG00000255663	RP11-212D19.4	1.00E-06	0.065408	-15.99717948

ENSG00000188886	ASTL	1.00E-06	0.065442128	-15.99793204
ENSG00000228162	AC097713.3	1.00E-06	0.065484	-15.99885483
ENSG00000260828	HMGB3P32	1.00E-06	0.065601	-16.00143019
ENSG00000207235	Y_RNA	1.00E-06	0.065601	-16.00143019
ENSG00000233438	AC109829.1	1.00E-06	0.065601	-16.00143019
ENSG00000176302	FOXR1	1.00E-06	0.065627321	-16.00200892
ENSG00000225383	SFTA1P	1.00E-06	0.065680194	-16.00317077
ENSG00000276795	RP11-374M1.10	1.00E-06	0.065681	-16.00318847
ENSG00000261656	BEAN1-AS1	1.00E-06	0.065759097	-16.00490288
ENSG00000224854	CDKN2A-AS1	1.00E-06	0.06576	-16.00492268
ENSG00000214688	C10orf105	1.00E-06	0.065813198	-16.00608932
ENSG00000253572	RP11-107N7.1	1.00E-06	0.065964	-16.00939126
ENSG00000248148	RP11-357F12.1	1.00E-06	0.066001	-16.01020026
ENSG00000186207	LCE5A	1.00E-06	0.066081	-16.0119479
ENSG00000259694	RP13-262C2.3	1.00E-06	0.066081	-16.0119479
ENSG00000259551	RP11-182J1.10	1.00E-06	0.066081	-16.0119479
ENSG00000227838	RP1-213J1P__B.1	1.00E-06	0.066081	-16.0119479
ENSG00000248371	CTC-347C20.2	1.00E-06	0.066161655	-16.01370771
ENSG00000254636	ARMS2	1.00E-06	0.066162	-16.01371523
ENSG00000234793	AC114730.7	1.00E-06	0.066238	-16.01537149
ENSG00000258989	RP11-47I22.4	1.00E-06	0.066332	-16.01741741
ENSG00000243179	AC110769.3	1.00E-06	0.066406	-16.01902598
ENSG00000234832	RP3-322G13.7	1.00E-06	0.066487	-16.02078466
ENSG00000272551	RP11-324L17.1	1.00E-06	0.066569	-16.02256288
ENSG00000258863	SETP1	1.00E-06	0.066609	-16.0234295
ENSG00000263558	RN7SL716P	1.00E-06	0.066641	-16.02412243
ENSG00000264125	RP11-354P11.4	1.00E-06	0.066777	-16.02706366
ENSG00000238825	RNU1-13P	1.00E-06	0.066815	-16.0278844
ENSG00000257918	RP11-482D24.3	1.00E-06	0.066898	-16.02967546
ENSG00000241354	RP11-1042B17.2	1.00E-06	0.066954	-16.03088263
ENSG00000255082	GRM5-AS1	1.00E-06	0.067021568	-16.03233782
ENSG00000270174	RP1-256G22.2	1.00E-06	0.067147	-16.03503532
ENSG00000224958	PGM5-AS1	1.00E-06	0.067202424	-16.03622565
ENSG00000229862	RP11-505P4.7	1.00E-06	0.067314035	-16.03861971
ENSG00000250407	CTB-99A3.1	1.00E-06	0.067357	-16.03954026
ENSG00000224055	GAPDHP55	1.00E-06	0.067397	-16.04039675
ENSG00000239877	IGSF11-AS1	1.00E-06	0.067482	-16.04221511
ENSG00000240542	KRTAP9-1	1.00E-06	0.067561	-16.04390306
ENSG00000262974	RP11-457I16.4	1.00E-06	0.067651	-16.04582364
ENSG00000235128	AC013474.4	1.00E-06	0.067735	-16.04761387
ENSG00000275443	RP11-759A24.3	1.00E-06	0.067741	-16.04774166
ENSG00000274717	RP1-47A17.1	1.00E-06	0.067821	-16.04944444
ENSG00000267992	CTB-189B5.3	1.00E-06	0.067905	-16.05123019
ENSG00000250714	RP11-1149O23.4	1.00E-06	0.067952	-16.05222839
ENSG00000279629	RP11-426J5.1	1.00E-06	0.067991	-16.05305617
ENSG00000205076	LGALS7	1.00E-06	0.068075859	-16.05485566
ENSG00000251687	RP11-181K12.2	1.00E-06	0.068162	-16.05668005
ENSG00000226786	RP1-167F1.2	1.00E-06	0.068162	-16.05668005

ENSG00000244640	RP11-436J20.1	1.00E-06	0.06817	-16.05684936
ENSG00000264544	AL589988.1	1.00E-06	0.068552	-16.06491114
ENSG00000280253	AC009758.1	1.00E-06	0.068724	-16.06852639
ENSG00000165794	SLC39A2	1.00E-06	0.068724107	-16.06852863
ENSG00000236254	MTND4P14	1.00E-06	0.06875	-16.06907209
ENSG00000231212	RP11-111F5.3	1.00E-06	0.068767811	-16.0694458
ENSG00000226851	AC004112.4	1.00E-06	0.068894291	-16.07209682
ENSG00000256379	TRAV8-5	1.00E-06	0.069063	-16.07562538
ENSG00000230088	KRT16P5	1.00E-06	0.069119	-16.07679472
ENSG00000168928	CTRB2	1.00E-06	0.069207928	-16.0786497
ENSG00000264443	RP4-594I10.3	1.00E-06	0.069226	-16.07902637
ENSG00000186842	LINC00846	1.00E-06	0.069258	-16.07969311
ENSG00000235277	AF127577.8	1.00E-06	0.069296	-16.08048446
ENSG00000255084	RP11-843A23.1	1.00E-06	0.069443	-16.08354165
ENSG00000222932	RNU6-172P	1.00E-06	0.069833	-16.09162133
ENSG00000263508	RP11-963H4.3	1.00E-06	0.069923163	-16.09348282
ENSG00000258847	CTD-2014B16.3	1.00E-06	0.070014	-16.09535581
ENSG00000237251	AC072061.2	1.00E-06	0.070195	-16.09908065
ENSG00000203266	RP11-560A15.3	1.00E-06	0.070286	-16.10094973
ENSG00000228914	OR1H1P	1.00E-06	0.070745	-16.11034057
ENSG00000262118	MTCO1P28	1.00E-06	0.070838	-16.11223586
ENSG00000267717	SRSF10P1	1.00E-06	0.070838	-16.11223586
ENSG00000237087	AC068134.6	1.00E-06	0.070855	-16.11258204
ENSG00000197046	SIGLEC15	1.00E-06	0.0709206	-16.11391713
ENSG00000259283	RP11-26L20.3	1.00E-06	0.070931	-16.11412867
ENSG00000264808	RP11-802D6.1	1.00E-06	0.071025	-16.11603931
ENSG00000243521	RPL5P33	1.00E-06	0.071044	-16.11642519
ENSG00000255772	LINC01479	1.00E-06	0.071151793	-16.11861249
ENSG00000233048	RP5-1069C8.2	1.00E-06	0.071197	-16.11952883
ENSG00000230555	RP11-517P14.2	1.00E-06	0.071211	-16.11981249
ENSG00000254275	LINC00824	1.00E-06	0.071258038	-16.12076514
ENSG00000251696	AL583832.1	1.00E-06	0.071368	-16.12298972
ENSG00000248176	RP11-472K22.1	1.00E-06	0.071399	-16.12361625
ENSG00000236101	RAC1P7	1.00E-06	0.071778	-16.1312541
ENSG00000166856	GPR182	1.00E-06	0.071783302	-16.13136067
ENSG00000272203	AC004775.5	1.00E-06	0.071873	-16.13316229
ENSG00000267289	CTD-2623N2.11	1.00E-06	0.072161	-16.13893171
ENSG00000224565	RP1-148H17.1	1.00E-06	0.072161	-16.13893171
ENSG00000185053	SGCZ	1.00E-06	0.072192271	-16.13955677
ENSG00000207134	RNU6-106P	1.00E-06	0.072257	-16.14084974
ENSG00000201260	RNU6-1075P	1.00E-06	0.072257	-16.14084974
ENSG00000207306	RNU6-1152P	1.00E-06	0.072257	-16.14084974
ENSG00000235024	AC097468.7	1.00E-06	0.072422	-16.1441404
ENSG00000249441	RP11-94H6.1	1.00E-06	0.072463	-16.14495691
ENSG00000164047	CAMP	1.00E-06	0.072645	-16.14857588
ENSG00000272986	RP11-46J23.1	1.00E-06	0.072645	-16.14857588
ENSG00000235733	RP3-522P13.3	1.00E-06	0.072645	-16.14857588
ENSG00000263218	CTD-2561B21.7	1.00E-06	0.072706169	-16.14979016

ENSG00000252072	RNA5SP320	1.00E-06	0.072742	-16.15050097
ENSG00000279509	RP11-329J18.5	1.00E-06	0.072841	-16.15246311
ENSG00000212568	RNU6-1254P	1.00E-06	0.072855	-16.15274037
ENSG00000280755	AC009950.1	1.00E-06	0.072896	-16.15355203
ENSG00000266340	RP11-848P1.4	1.00E-06	0.072917	-16.15396759
ENSG00000115607	IL18RAP	1.00E-06	0.072987858	-16.15536886
ENSG00000253773	C8orf37-AS1	1.00E-06	0.073034784	-16.15629612
ENSG00000197254	AP000445.1	1.00E-06	0.073037	-16.15633989
ENSG00000179774	ATOH7	1.00E-06	0.073136	-16.1582941
ENSG00000270734	RP11-719K4.7	1.00E-06	0.073136	-16.1582941
ENSG00000270755	RP11-138E2.1	1.00E-06	0.073334	-16.16219461
ENSG00000248647	RP11-331K21.1	1.00E-06	0.07333419	-16.16219834
ENSG00000205293	LINC01602	1.00E-06	0.073393	-16.16335485
ENSG00000230709	AC104024.1	1.00E-06	0.073767	-16.17068794
ENSG00000272963	OR7A19P	1.00E-06	0.073834	-16.1719977
ENSG00000267069	RP11-64C12.8	1.00E-06	0.073834	-16.1719977
ENSG00000165131	C7orf34	1.00E-06	0.073880519	-16.17290638
ENSG00000267627	RP11-905K4.1	1.00E-06	0.074036405	-16.17594722
ENSG00000249189	AC010492.4	1.00E-06	0.074138	-16.17792558
ENSG00000249984	CTC-529L17.2	1.00E-06	0.074138	-16.17792558
ENSG00000244203	FOXP1-AS1	1.00E-06	0.074222	-16.17955926
ENSG00000259161	CTD-2017C7.3	1.00E-06	0.074239	-16.17988966
ENSG00000279487	AC234582.1	1.00E-06	0.074253	-16.18016169
ENSG00000229747	RP11-567B20.2	1.00E-06	0.074341	-16.18187047
ENSG00000255921	RP11-662I13.2	1.00E-06	0.074341	-16.18187047
ENSG00000244757	RP11-45A16.4	1.00E-06	0.074341	-16.18187047
ENSG00000179002	TAS1R2	1.00E-06	0.074354	-16.18212274
ENSG00000254484	CTA-797E19.1	1.00E-06	0.074383	-16.18268532
ENSG00000270036	RP11-685G9.4	1.00E-06	0.074752	-16.18982456
ENSG00000250331	LINC01340	1.00E-06	0.074752	-16.18982456
ENSG00000196970	NXF4	1.00E-06	0.074814	-16.19102065
ENSG00000250519	RP11-680H20.2	1.00E-06	0.07485527	-16.19181626
ENSG00000226114	NDUFB4P5	1.00E-06	0.074959	-16.19381409
ENSG00000237336	RP11-230L22.4	1.00E-06	0.074959	-16.19381409
ENSG00000172296	SPTLC3	1.00E-06	0.075083013	-16.19619892
ENSG00000263499	RP11-118E18.4	1.00E-06	0.075125	-16.19700547
ENSG00000228919	AC097381.1	1.00E-06	0.075166978	-16.19781138
ENSG00000256134	EGLN3P1	1.00E-06	0.075167	-16.1978118
ENSG00000237661	RP11-169L17.5	1.00E-06	0.075272	-16.19982568
ENSG00000164123	C4orf45	1.00E-06	0.075272545	-16.19983613
ENSG00000249196	RP11-669N7.2	1.00E-06	0.075310602	-16.20056536
ENSG00000230611	HMGB1P27	1.00E-06	0.075392	-16.20212382
ENSG00000277427	XXbac-BPG154L12.5	1.00E-06	0.075556	-16.20525871
ENSG00000279036	RP11-81K2.2	1.00E-06	0.075629	-16.20665192
ENSG00000156234	CXCL13	1.00E-06	0.075640201	-16.20686558
ENSG00000215692	AC114730.8	1.00E-06	0.075677	-16.20756728
ENSG00000175514	GPR152	1.00E-06	0.075746	-16.20888208
ENSG00000188477	AC003003.5	1.00E-06	0.075822662	-16.21034148

ENSG00000224593	RP11-30B1.1	1.00E-06	0.075905644	-16.21191954
ENSG00000254237	RP11-115J16.2	1.00E-06	0.075946197	-16.2126901
ENSG00000249691	RP11-445J14.1	1.00E-06	0.076001	-16.21373078
ENSG00000203435	E2F3P2	1.00E-06	0.076118	-16.21595003
ENSG00000259066	RP11-371E8.4	1.00E-06	0.0761286	-16.21615093
ENSG00000244493	SLC9A9-AS2	1.00E-06	0.076132	-16.21621536
ENSG00000233632	RP1-302D9.5	1.00E-06	0.07615	-16.21655642
ENSG00000242268	RP11-368I23.2	1.00E-06	0.076227	-16.21801448
ENSG00000253642	RP11-317N12.1	1.00E-06	0.076333845	-16.22003524
ENSG00000279835	RP4-811M8.1	1.00E-06	0.076441	-16.22205903
ENSG00000248528	CTC-458G6.2	1.00E-06	0.076549	-16.22409591
ENSG00000261045	RP11-673P17.4	1.00E-06	0.076644	-16.22588524
ENSG00000274686	Metazoa_SRP	1.00E-06	0.076739	-16.22767234
ENSG00000237324	RP11-439H8.4	1.00E-06	0.07687584	-16.23024265
ENSG00000250551	RP11-254I22.1	1.00E-06	0.076876	-16.23024565
ENSG00000232361	RP11-632K21.3	1.00E-06	0.076876	-16.23024565
ENSG00000184374	COLEC10	1.00E-06	0.077129853	-16.23500174
ENSG00000234142	RP11-276E17.2	1.00E-06	0.077205	-16.23640666
ENSG00000270249	RP11-514P8.7	1.00E-06	0.077255	-16.23734069
ENSG00000264041	RN7SL670P	1.00E-06	0.077316	-16.23847938
ENSG00000266491	AC109335.1	1.00E-06	0.077316	-16.23847938
ENSG00000257514	RP11-755O11.2	1.00E-06	0.077425399	-16.24051929
ENSG00000236947	RP11-98G7.1	1.00E-06	0.077627045	-16.24427176
ENSG00000280474	RP11-216B9.8	1.00E-06	0.077901	-16.24935423
ENSG00000250470	AP000351.3	1.00E-06	0.077983	-16.25087204
ENSG00000149021	SCGB1A1	1.00E-06	0.078112649	-16.25326858
ENSG00000279127	RP3-442L6.4	1.00E-06	0.078172	-16.25436433
ENSG00000261044	AP006547.3	1.00E-06	0.078209	-16.25504702
ENSG00000218016	ZNF192P2	1.00E-06	0.078266	-16.25609809
ENSG00000226542	AC114814.4	1.00E-06	0.078322	-16.25712999
ENSG00000273214	RP5-1039K5.18	1.00E-06	0.078322	-16.25712999
ENSG00000282301	CYP3A7-CYP3A51P	1.00E-06	0.078359733	-16.25782486
ENSG00000270361	RP11-307C12.13	1.00E-06	0.078436	-16.25922834
ENSG00000231826	AC016735.2	1.00E-06	0.078463083	-16.2597264
ENSG00000278924	RP11-227L6.1	1.00E-06	0.078527	-16.26090116
ENSG00000261227	AC140912.1	1.00E-06	0.078738696	-16.2647852
ENSG00000279819	RP11-142L16.2	1.00E-06	0.078778	-16.26550517
ENSG00000266369	RP11-344E13.4	1.00E-06	0.078893	-16.26760968
ENSG00000280288	RP11-756D7.2	1.00E-06	0.07895	-16.26865165
ENSG00000277608	RP11-321L2.3	1.00E-06	0.079008	-16.26971112
ENSG00000227078	AC004448.2	1.00E-06	0.079099	-16.27137184
ENSG00000234361	RP11-52J3.3	1.00E-06	0.079472	-16.27815903
ENSG00000275874	LINC00162	1.00E-06	0.079741	-16.28303408
ENSG00000258052	AC025263.3	1.00E-06	0.079789	-16.28390224
ENSG00000257953	RP11-620J15.1	1.00E-06	0.079849	-16.28498672
ENSG00000238181	AHCYP2	1.00E-06	0.079932	-16.28648557
ENSG00000278607	RP11-405M12.3	1.00E-06	0.079962	-16.28702694
ENSG00000253663	NPM1P52	1.00E-06	0.079965	-16.28708106

ENSG00000232023	AC009410.1	1.00E-06	0.080059	-16.28877597
ENSG00000254792	RP11-119D9.4	1.00E-06	0.080178	-16.29091881
ENSG00000277763	CTD-2588E21.1	1.00E-06	0.080178	-16.29091881
ENSG00000241932	RP11-14K2.1	1.00E-06	0.080178	-16.29091881
ENSG00000242767	ZBTB20-AS4	1.00E-06	0.080178	-16.29091881
ENSG00000227017	AC007036.6	1.00E-06	0.080298	-16.29307643
ENSG00000256499	CTC-465D4.1	1.00E-06	0.080369	-16.29435151
ENSG00000267749	CTC-265F19.1	1.00E-06	0.080537	-16.29736411
ENSG00000235433	MTATP6P17	1.00E-06	0.080537	-16.29736411
ENSG00000115009	CCL20	1.00E-06	0.080597101	-16.29844033
ENSG00000228802	AC073641.2	1.00E-06	0.080739	-16.3009781
ENSG00000213774	AC010904.1	1.00E-06	0.080751	-16.30119251
ENSG00000224493	RP11-87O11.1	1.00E-06	0.080777	-16.30165695
ENSG00000228097	MTATP6P11	1.00E-06	0.080777	-16.30165695
ENSG00000168828	OR13J1	1.00E-06	0.080838	-16.30274601
ENSG00000240775	RP11-170N16.2	1.00E-06	0.08087	-16.30331699
ENSG00000206972	RNU6-17P	1.00E-06	0.080928	-16.30435132
ENSG00000200153	RNU6-23P	1.00E-06	0.080928	-16.30435132
ENSG00000267723	CTD-2189E23.1	1.00E-06	0.081138	-16.30809012
ENSG00000185903	OR11N1P	1.00E-06	0.081212	-16.3094053
ENSG00000235644	RPL10P5	1.00E-06	0.081307	-16.31109194
ENSG00000251186	RP11-689P11.3	1.00E-06	0.081507	-16.31463635
ENSG00000275232	RP11-897M7.4	1.00E-06	0.081647	-16.31711226
ENSG00000207360	RNU6-14P	1.00E-06	0.081692	-16.31790718
ENSG00000228275	ARMCX3-AS1	1.00E-06	0.081733	-16.31863107
ENSG00000226729	RPL35AP30	1.00E-06	0.081753	-16.31898405
ENSG00000231327	AC016700.5	1.00E-06	0.081753	-16.31898405
ENSG00000227688	HNRNPA3P2	1.00E-06	0.081753	-16.31898405
ENSG00000259705	RP11-227D13.1	1.00E-06	0.081891	-16.32141728
ENSG00000270071	AP001172.2	1.00E-06	0.081973	-16.32286118
ENSG00000254758	AP000889.1	1.00E-06	0.082001	-16.32335388
ENSG00000259367	RP11-815J21.4	1.00E-06	0.082001	-16.32335388
ENSG00000236062	GSTM5P1	1.00E-06	0.082001	-16.32335388
ENSG00000253069	AC021205.1	1.00E-06	0.082001	-16.32335388
ENSG00000249174	RP11-124N3.3	1.00E-06	0.082001	-16.32335388
ENSG00000231404	RAC1P3	1.00E-06	0.082114	-16.32534059
ENSG00000282111	RP5-1065P14.3	1.00E-06	0.082126	-16.32555141
ENSG00000259672	RP11-69G7.1	1.00E-06	0.08225	-16.32772806
ENSG00000237963	RP11-151G12.2	1.00E-06	0.08225	-16.32772806
ENSG00000272461	RP11-689C9.1	1.00E-06	0.082376	-16.32993645
ENSG00000248300	RP11-74M11.2	1.00E-06	0.082376	-16.32993645
ENSG00000234208	CTA-256D12.11	1.00E-06	0.082399	-16.33033921
ENSG00000228000	RPL7AP65	1.00E-06	0.082483	-16.33180919
ENSG00000247872	SPCS2P3	1.00E-06	0.082501	-16.33212399
ENSG00000228054	RANP5	1.00E-06	0.082627	-16.33432567
ENSG00000237385	RP11-165J3.5	1.00E-06	0.082743	-16.33634965
ENSG00000260307	PABPC1P13	1.00E-06	0.082974	-16.34037172
ENSG00000228019	RP11-166O4.4	1.00E-06	0.083043	-16.34157094

ENSG00000253692	IGHEP1	1.00E-06	0.083135	-16.34316836
ENSG00000279416	RP11-849I19.2	1.00E-06	0.083135	-16.34316836
ENSG00000274554	RP11-240G22.5	1.00E-06	0.083189	-16.34410515
ENSG00000236095	RP11-49O14.2	1.00E-06	0.083369	-16.34722341
ENSG00000224853	LINC00393	1.00E-06	0.083390746	-16.34759967
ENSG00000240296	AC010492.5	1.00E-06	0.083391	-16.34760407
ENSG00000214657	RP11-120J4.1	1.00E-06	0.083399	-16.34774246
ENSG00000273727	U1	1.00E-06	0.083519	-16.34981682
ENSG00000239397	RP11-101K23.1	1.00E-06	0.083519	-16.34981682
ENSG00000124610	HIST1H1A	1.00E-06	0.083519	-16.34981682
ENSG00000253539	RP11-402L5.1	1.00E-06	0.083519	-16.34981682
ENSG00000226490	AC138647.1	1.00E-06	0.083519	-16.34981682
ENSG00000271771	RP11-1250I15.3	1.00E-06	0.083649	-16.35206067
ENSG00000227911	RP11-141M1.1	1.00E-06	0.084038	-16.35875421
ENSG00000226183	RANP7	1.00E-06	0.084038	-16.35875421
ENSG00000226928	RPS14P4	1.00E-06	0.084159	-16.36082994
ENSG00000230902	FAM204CP	1.00E-06	0.0843	-16.36324501
ENSG00000261332	RP11-297C4.1	1.00E-06	0.084417	-16.36524594
ENSG00000264067	RP11-401O9.3	1.00E-06	0.084431	-16.36548518
ENSG00000235335	AC016723.4	1.00E-06	0.084526604	-16.36711787
ENSG00000256084	RP11-134N1.2	1.00E-06	0.084563	-16.36773894
ENSG00000274879	RP11-411G2.1	1.00E-06	0.084658	-16.36935879
ENSG00000258675	RP11-299L17.3	1.00E-06	0.084962	-16.37453011
ENSG00000251171	RP11-327O17.2	1.00E-06	0.084962	-16.37453011
ENSG00000257890	RP11-114F10.2	1.00E-06	0.085634	-16.3858961
ENSG00000240423	LINC00636	1.00E-06	0.085634	-16.3858961
ENSG00000247774	PCED1B-AS1	1.00E-06	0.085767144	-16.38813745
ENSG00000237754	RP11-521C10.1	1.00E-06	0.085769	-16.38816868
ENSG00000226780	RP11-38J22.3	1.00E-06	0.085906	-16.39047128
ENSG00000232655	CTA-397C4.2	1.00E-06	0.085906	-16.39047128
ENSG00000171773	NXNL1	1.00E-06	0.085973	-16.39159603
ENSG00000269621	RP11-98D18.15	1.00E-06	0.086042	-16.39275344
ENSG00000271290	RP11-345E19.2	1.00E-06	0.086042	-16.39275344
ENSG00000265246	RP11-663N22.1	1.00E-06	0.086382	-16.3984431
ENSG00000265123	RN7SL200P	1.00E-06	0.086442	-16.39944483
ENSG00000231403	AC099344.3	1.00E-06	0.086454	-16.39964509
ENSG00000233544	EIF3KP2	1.00E-06	0.086454	-16.39964509
ENSG00000261684	RP11-265N6.1	1.00E-06	0.086489	-16.40022904
ENSG00000227343	RPL15P1	1.00E-06	0.086593	-16.40196278
ENSG00000280036	RP11-540O11.8	1.00E-06	0.08699	-16.40856194
ENSG00000278737	RP11-325L12.7	1.00E-06	0.087011	-16.40891018
ENSG00000251123	RP11-580J4.1	1.00E-06	0.087011	-16.40891018
ENSG00000273744	U4	1.00E-06	0.087091	-16.41023602
ENSG00000169469	SPRR1B	1.00E-06	0.087291	-16.41354529
ENSG00000230952	AC099344.2	1.00E-06	0.087444	-16.41607178
ENSG00000256568	RP11-800A3.3	1.00E-06	0.087574	-16.41821499
ENSG00000271394	7SK	1.00E-06	0.087645	-16.41938417
ENSG00000170099	SERPINA6	1.00E-06	0.08766878	-16.41977555

ENSG00000279841	RP11-274A11.4	1.00E-06	0.087953	-16.42444517
ENSG00000278863	RP11-567L7.3	1.00E-06	0.088144	-16.42757475
ENSG00000244355	LY6G6D	1.00E-06	0.088144239	-16.42757867
ENSG00000218180	SLC25A5P7	1.00E-06	0.088187	-16.42827838
ENSG00000250170	RASA2-IT1	1.00E-06	0.088432	-16.4322809
ENSG00000256341	RP11-21A7A.3	1.00E-06	0.088475	-16.43298224
ENSG00000224441	AC068831.3	1.00E-06	0.088481	-16.43308007
ENSG00000199731	RNU6-1079P	1.00E-06	0.088561	-16.43438389
ENSG00000249474	RP11-49I4.3	1.00E-06	0.088709	-16.43679286
ENSG00000236648	RP11-473A10.2	1.00E-06	0.088722	-16.43700427
ENSG00000279431	RP11-218M11.7	1.00E-06	0.088722	-16.43700427
ENSG00000235872	RP11-335O4.3	1.00E-06	0.088762	-16.43765455
ENSG00000243819	RN7SL832P	1.00E-06	0.088763	-16.43767081
ENSG00000260113	RP11-146F11.2	1.00E-06	0.088868	-16.4393764
ENSG00000213486	AC108039.1	1.00E-06	0.088868	-16.4393764
ENSG00000224987	RP1-191J18.65	1.00E-06	0.088884	-16.43963612
ENSG00000266371	RP11-142O6.1	1.00E-06	0.088898	-16.43986334
ENSG00000267857	RP5-1023B21.1	1.00E-06	0.089014	-16.44174464
ENSG00000275853	Metazoa_SRP	1.00E-06	0.089014	-16.44174464
ENSG00000250084	RP11-485B17.5	1.00E-06	0.089014	-16.44174464
ENSG00000268051	CTC-244M17.1	1.00E-06	0.08916	-16.444109
ENSG00000233479	AC017074.1	1.00E-06	0.08916	-16.444109
ENSG00000204475	NCR3	1.00E-06	0.089307748	-16.44649772
ENSG00000220702	RP1-32I10.10	1.00E-06	0.089308	-16.44650179
ENSG00000238287	RP11-656D10.3	1.00E-06	0.089456	-16.44889063
ENSG00000249207	RP11-360F5.1	1.00E-06	0.089506	-16.44969678
ENSG00000228367	RP11-524L6.2	1.00E-06	0.089564	-16.45063134
ENSG00000228692	RP5-826L7.1	1.00E-06	0.089901	-16.45604954
ENSG00000253355	KB-1460A1.2	1.00E-06	0.089901409	-16.4560561
ENSG00000258999	RP11-114N19.3	1.00E-06	0.090051	-16.45845468
ENSG00000234265	RP11-157E14.1	1.00E-06	0.090051	-16.45845468
ENSG00000281325	AC016549.1	1.00E-06	0.090101	-16.4592555
ENSG00000254418	RP11-21L19.1	1.00E-06	0.090201	-16.46085581
ENSG00000260082	RP11-2C24.5	1.00E-06	0.090201	-16.46085581
ENSG00000267388	AF038458.5	1.00E-06	0.090201	-16.46085581
ENSG00000277843	AC106753.1	1.00E-06	0.090201	-16.46085581
ENSG00000276893	AL136231.1	1.00E-06	0.090201	-16.46085581
ENSG00000276898	RP11-118H15.1	1.00E-06	0.090201	-16.46085581
ENSG00000236975	RP5-1065P14.2	1.00E-06	0.090351	-16.46325295
ENSG00000257764	RP11-1143G9.4	1.00E-06	0.090351	-16.46325295
ENSG00000269576	HNRNPMP2	1.00E-06	0.090351	-16.46325295
ENSG00000266938	AC119403.1	1.00E-06	0.090503	-16.465678
ENSG00000237628	MTCO2P19	1.00E-06	0.090503	-16.465678
ENSG00000226827	NPM1P11	1.00E-06	0.090518	-16.46591709
ENSG00000280444	RP11-83B20.8	1.00E-06	0.090654	-16.46808306
ENSG00000213115	AC104131.1	1.00E-06	0.090654	-16.46808306
ENSG00000249252	RP11-665G4.1	1.00E-06	0.090698467	-16.46879055
ENSG00000272149	RP11-627J17.1	1.00E-06	0.090763	-16.46981667

ENSG00000258323	RP1-267L14.3	1.00E-06	0.090807	-16.47051589
ENSG00000241353	PPP1R2P4	1.00E-06	0.090959	-16.47292877
ENSG00000130167	TSPAN16	1.00E-06	0.091009564	-16.47373055
ENSG00000203307	RP11-375H19.2	1.00E-06	0.091112	-16.47535346
ENSG00000275147	RP11-324D17.3	1.00E-06	0.091112	-16.47535346
ENSG00000212101	AL080276.1	1.00E-06	0.091287	-16.4781218
ENSG00000267905	CTD-2616J11.16	1.00E-06	0.091436763	-16.4804867
ENSG00000145839	IL9	1.00E-06	0.091574	-16.48265042
ENSG00000091010	POU4F3	1.00E-06	0.091574	-16.48265042
ENSG00000278549	MIR6736	1.00E-06	0.09173	-16.48510602
ENSG00000260377	RP11-646E18.4	1.00E-06	0.09173	-16.48510602
ENSG00000241391	RN7SL234P	1.00E-06	0.09173	-16.48510602
ENSG00000215159	RP11-629N8.3	1.00E-06	0.091886	-16.48755744
ENSG00000258125	RP11-1041F24.1	1.00E-06	0.092027	-16.48976958
ENSG00000259475	RP11-654A16.3	1.00E-06	0.092042	-16.49000471
ENSG00000250855	RP11-269F21.1	1.00E-06	0.092059	-16.49027115
ENSG00000278047	FP671120.6	1.00E-06	0.092162	-16.4918844
ENSG00000277145	AL592188.7	1.00E-06	0.092162	-16.4918844
ENSG00000226520	KIRREL-IT1	1.00E-06	0.092199	-16.49246348
ENSG00000259649	RP11-351M8.1	1.00E-06	0.09242339	-16.49597038
ENSG00000255808	RP11-31L22.3	1.00E-06	0.092448	-16.49635449
ENSG00000236856	AC105393.1	1.00E-06	0.092665	-16.49973691
ENSG00000272516	RP11-573G6.9	1.00E-06	0.092672	-16.49984589
ENSG00000259067	CTD-3051D23.3	1.00E-06	0.092672	-16.49984589
ENSG00000243385	CTD-2110K23.1	1.00E-06	0.092672	-16.49984589
ENSG00000267585	RP11-115P21.1	1.00E-06	0.09272	-16.50059295
ENSG00000267650	CTD-2553C6.1	1.00E-06	0.092752	-16.50109077
ENSG00000217447	RP3-366N23.4	1.00E-06	0.092991	-16.50480347
ENSG00000280080	U51561.1	1.00E-06	0.093151	-16.50728364
ENSG00000236242	MYO16-AS1	1.00E-06	0.093311	-16.50975954
ENSG00000237691	IFNWP2	1.00E-06	0.093311	-16.50975954
ENSG00000253911	KB-1205A7.2	1.00E-06	0.093498	-16.51264788
ENSG00000259327	CTD-2184D3.6	1.00E-06	0.09354	-16.51329581
ENSG00000241358	RP11-758I14.3	1.00E-06	0.09355674	-16.51355397
ENSG00000234223	AC003988.1	1.00E-06	0.093634	-16.51474487
ENSG00000254180	AB015752.3	1.00E-06	0.093634	-16.51474487
ENSG00000259756	RP11-625H11.2	1.00E-06	0.093796	-16.51723878
ENSG00000178997	EXD1	1.00E-06	0.093878007	-16.51849959
ENSG00000233583	RP4-635A23.3	1.00E-06	0.09396	-16.51975909
ENSG00000259389	H3F3AP1	1.00E-06	0.093981	-16.5200815
ENSG00000259163	RP11-1078H9.5	1.00E-06	0.094081048	-16.52161651
ENSG00000168070	C11orf85	1.00E-06	0.094082081	-16.52163235
ENSG00000226889	RP11-474I16.8	1.00E-06	0.094123	-16.52225968
ENSG00000257452	RP1-71H24.1	1.00E-06	0.094123	-16.52225968
ENSG00000253264	PCAT2	1.00E-06	0.094123	-16.52225968
ENSG00000265733	SNORA74	1.00E-06	0.09424	-16.52405192
ENSG00000223111	SNORA74	1.00E-06	0.09424	-16.52405192
ENSG00000256108	RP13-672B3.5	1.00E-06	0.094287	-16.52477125

ENSG00000238092	CEACAMP6	1.00E-06	0.094287	-16.52477125
ENSG00000232810	TNF	1.00E-06	0.094291	-16.52483245
ENSG00000272563	RP11-480C16.1	1.00E-06	0.094314	-16.52518432
ENSG00000212712	AP002414.1	1.00E-06	0.094369	-16.52602539
ENSG00000267557	CTC-379B2.4	1.00E-06	0.094451	-16.52727845
ENSG00000242657	RN7SL581P	1.00E-06	0.09458	-16.52924752
ENSG00000228201	AL022341.3	1.00E-06	0.094607	-16.52965931
ENSG00000282952	RP11-17G2.1	1.00E-06	0.094617	-16.5298118
ENSG00000274269	RP4-545L17.12	1.00E-06	0.094617	-16.5298118
ENSG00000248956	HMGB1P44	1.00E-06	0.094617	-16.5298118
ENSG00000214875	MED28P1	1.00E-06	0.094782	-16.53232548
ENSG00000249036	RP11-625I7.1	1.00E-06	0.094782	-16.53232548
ENSG00000253965	CTC-329D1.3	1.00E-06	0.094948	-16.53484999
ENSG00000271163	RP4-607J2.1	1.00E-06	0.094948	-16.53484999
ENSG00000224416	IFNA22P	1.00E-06	0.094948	-16.53484999
ENSG00000237205	RPL7P34	1.00E-06	0.095015	-16.53586767
ENSG00000250734	RP11-404E16.1	1.00E-06	0.09509	-16.53700601
ENSG00000237631	RP11-72B4.2	1.00E-06	0.095115	-16.53738526
ENSG00000229294	RP5-1044H5.1	1.00E-06	0.095134	-16.53767342
ENSG00000159516	SPRR2G	1.00E-06	0.095282	-16.53991608
ENSG00000254362	RP11-14I17.3	1.00E-06	0.095282	-16.53991608
ENSG00000233255	AC019181.2	1.00E-06	0.09545	-16.54245758
ENSG00000214676	RPL9P16	1.00E-06	0.09545	-16.54245758
ENSG00000278886	RP11-108A14.1	1.00E-06	0.09545	-16.54245758
ENSG00000274470	AC079917.1	1.00E-06	0.095507	-16.54331886
ENSG00000254985	RSF1-IT2	1.00E-06	0.095619	-16.5450097
ENSG00000225533	PAWRP1	1.00E-06	0.095789	-16.54757237
ENSG00000261191	RP11-16L14.2	1.00E-06	0.095789	-16.54757237
ENSG00000269303	CTD-2527I21.7	1.00E-06	0.095789	-16.54757237
ENSG00000226423	AC093642.4	1.00E-06	0.095789	-16.54757237
ENSG00000237766	GGTA2P	1.00E-06	0.096214	-16.55395921
ENSG00000258375	RP11-617J18.1	1.00E-06	0.096214	-16.55395921
ENSG00000234257	SOD2P1	1.00E-06	0.0963	-16.55524818
ENSG00000254187	CTB-78F1.1	1.00E-06	0.0963	-16.55524818
ENSG00000278099	U1	1.00E-06	0.096433	-16.55723931
ENSG00000254003	CTB-167B5.1	1.00E-06	0.096551	-16.55900358
ENSG00000259398	RP11-430B1.1	1.00E-06	0.096593	-16.55963102
ENSG00000255296	RP11-757C15.4	1.00E-06	0.096644	-16.56039255
ENSG00000229456	RLIMP1	1.00E-06	0.096644	-16.56039255
ENSG00000239199	RPL21P6	1.00E-06	0.096644	-16.56039255
ENSG00000265964	RP11-293E1.1	1.00E-06	0.096644	-16.56039255
ENSG00000264910	RN7SL525P	1.00E-06	0.096644	-16.56039255
ENSG00000226533	BTBD9-AS1	1.00E-06	0.096644	-16.56039255
ENSG00000258935	RP11-1078H9.2	1.00E-06	0.096816	-16.56295787
ENSG00000233021	RP11-490E15.2	1.00E-06	0.09699	-16.56554839
ENSG00000251568	ALG3P1	1.00E-06	0.097165	-16.56814911
ENSG00000235815	RP1-125N5.2	1.00E-06	0.097165	-16.56814911
ENSG00000273133	RP11-799M12.2	1.00E-06	0.097339	-16.57073033

ENSG00000249293	CTC-575I10.1	1.00E-06	0.097339	-16.57073033
ENSG00000251164	HULC	1.00E-06	0.097339	-16.57073033
ENSG00000261898	RP11-314A20.5	1.00E-06	0.097406	-16.57172302
ENSG00000265374	RP11-57J16.1	1.00E-06	0.097514	-16.57332174
ENSG00000268318	CTD-3187F8.11	1.00E-06	0.097603025	-16.57463824
ENSG00000227372	TP73-AS1	1.00E-06	0.097649849	-16.57533019
ENSG00000280026	AC005618.8	1.00E-06	0.097691	-16.57593804
ENSG00000120160	EQTN	1.00E-06	0.097696444	-16.57601843
ENSG00000258987	RP11-131H24.4	1.00E-06	0.097867	-16.57853486
ENSG00000261367	RP11-455F5.4	1.00E-06	0.097867	-16.57853486
ENSG00000230401	AC090505.4	1.00E-06	0.097867	-16.57853486
ENSG00000250338	RP11-395I6.2	1.00E-06	0.097867	-16.57853486
ENSG00000225384	RP13-643D4.1	1.00E-06	0.097867	-16.57853486
ENSG00000260719	AC009133.17	1.00E-06	0.098045	-16.58115644
ENSG00000224574	COL18A1-AS2	1.00E-06	0.098045	-16.58115644
ENSG00000249409	RP11-501E14.1	1.00E-06	0.098045159	-16.58115878
ENSG00000255118	RP11-703H8.7	1.00E-06	0.098198	-16.58340602
ENSG00000259106	RP11-242P2.2	1.00E-06	0.098222	-16.58375858
ENSG00000255672	RP11-45F15.2	1.00E-06	0.098401	-16.58638536
ENSG00000251188	RP11-478C6.6	1.00E-06	0.098401	-16.58638536
ENSG00000239705	RP11-65N13.8	1.00E-06	0.098401	-16.58638536
ENSG00000184867	ARMCX2	1.00E-06	0.098619862	-16.58959061
ENSG00000230427	RP11-313A24.1	1.00E-06	0.09876	-16.59163922
ENSG00000232715	LINC01022	1.00E-06	0.098901	-16.59369749
ENSG00000258480	RP11-662J14.2	1.00E-06	0.098941	-16.59428086
ENSG00000261630	RP11-357N13.3	1.00E-06	0.098941	-16.59428086
ENSG00000269806	GLTSCR1-AS1	1.00E-06	0.098941	-16.59428086
ENSG00000250101	RP11-1252I4.2	1.00E-06	0.098941	-16.59428086
ENSG00000275870	MIR6132	1.00E-06	0.099304	-16.59956421
ENSG00000225933	AC133965.1	1.00E-06	0.099374	-16.60058082
ENSG00000230698	CTD-2330K9.2	1.00E-06	0.099487	-16.6022204
ENSG00000212663	RP1-122K4.3	1.00E-06	0.099487	-16.6022204
ENSG00000278792	AC132192.1	1.00E-06	0.099532	-16.60287281
ENSG00000232518	AC074366.3	1.00E-06	0.099669842	-16.60486942
ENSG00000229414	KCNQ1-AS1	1.00E-06	0.099853	-16.60751815
ENSG00000259418	RP11-109D20.1	1.00E-06	0.099853	-16.60751815
ENSG00000267269	RP11-2N1.1	1.00E-06	0.099853	-16.60751815
ENSG00000259935	RP11-519C12.1	1.00E-06	0.100224	-16.6128685
ENSG00000269220	LINC00528	1.00E-06	0.100224	-16.6128685
ENSG00000279836	RP11-648O15.2	1.00E-06	0.100409	-16.61552906
ENSG00000237930	AC007563.4	1.00E-06	0.100558	-16.61766834
ENSG00000237513	RP11-325F22.2	1.00E-06	0.100571117	-16.61785651
ENSG00000226969	RP11-547D24.3	1.00E-06	0.100595288	-16.61820321
ENSG00000232230	TPM4P1	1.00E-06	0.100596	-16.61821341
ENSG00000126233	SLURP1	1.00E-06	0.100783	-16.62089278
ENSG00000279243	CMB9-22P13.2	1.00E-06	0.100972	-16.62359576
ENSG00000272226	RP11-63G10.3	1.00E-06	0.10116	-16.62627942
ENSG00000254566	RP11-514F3.4	1.00E-06	0.10116	-16.62627942

ENSG00000266541	RP11-881L2.1	1.00E-06	0.101349	-16.62897233
ENSG00000266989	FTLP5	1.00E-06	0.101349	-16.62897233
ENSG00000231976	LINC00202-2	1.00E-06	0.101484662	-16.63090218
ENSG00000231208	ZBTB46-AS1	1.00E-06	0.10154	-16.63168864
ENSG00000254171	RP11-67M9.1	1.00E-06	0.10154	-16.63168864
ENSG00000266849	AC083906.2	1.00E-06	0.101681	-16.6336906
ENSG00000215244	RP11-563J2.2	1.00E-06	0.101717	-16.63420129
ENSG00000227514	RP11-709P2.1	1.00E-06	0.101801	-16.63539221
ENSG00000234185	RP5-1132H15.1	1.00E-06	0.10181	-16.63551975
ENSG00000254688	RP11-47J17.3	1.00E-06	0.101822	-16.63568978
ENSG00000241886	RP11-242C19.2	1.00E-06	0.102078	-16.63931244
ENSG00000182327	GLTPD2	1.00E-06	0.102633	-16.64713516
ENSG00000273797	RP11-1046B16.3	1.00E-06	0.102696	-16.64802046
ENSG00000229037	RP11-272G22.3	1.00E-06	0.102696	-16.64802046
ENSG00000236527	ARF4P2	1.00E-06	0.102891	-16.65075727
ENSG00000106560	GIMAP2	1.00E-06	0.103234167	-16.65556101
ENSG00000237906	MUPP	1.00E-06	0.103268	-16.65603375
ENSG00000224222	RP11-262I2.2	1.00E-06	0.103284	-16.65625725
ENSG00000256757	RP11-159N11.3	1.00E-06	0.103284	-16.65625725
ENSG00000207653	MIR558	1.00E-06	0.103635	-16.66115179
ENSG00000254865	RP11-748C4.1	1.00E-06	0.103679	-16.66176418
ENSG00000213318	RP11-331F4.1	1.00E-06	0.103679	-16.66176418
ENSG00000277581	RP4-616B8.6	1.00E-06	0.103679	-16.66176418
ENSG00000274852	RN7SL422P	1.00E-06	0.103679	-16.66176418
ENSG00000273444	RP5-1147A1.2	1.00E-06	0.103704	-16.66211202
ENSG00000242061	CTD-2555K7.1	1.00E-06	0.103792	-16.66333572
ENSG00000233191	AC006372.6	1.00E-06	0.103792	-16.66333572
ENSG00000232792	FTH1P25	1.00E-06	0.103856	-16.66422504
ENSG00000279281	RP11-226M10.3	1.00E-06	0.103857	-16.66423893
ENSG00000274031	RP11-413H22.3	1.00E-06	0.103878	-16.66453062
ENSG00000239035	snoU13	1.00E-06	0.104078	-16.66730562
ENSG00000233469	ST6GALNAC4P1	1.00E-06	0.104119	-16.66787384
ENSG00000255629	RP11-196H14.3	1.00E-06	0.104133	-16.66806781
ENSG00000232793	DNAJC19P8	1.00E-06	0.10448	-16.67286728
ENSG00000279484	KLHL30-AS1	1.00E-06	0.104547	-16.67379214
ENSG00000263427	RP11-599B13.3	1.00E-06	0.104682	-16.67565387
ENSG00000211787	TRAV8-3	1.00E-06	0.104749	-16.67657695
ENSG00000280767	RP13-465B17.5	1.00E-06	0.105088	-16.68123841
ENSG00000271040	RP5-933K21.3	1.00E-06	0.105293	-16.68405
ENSG00000227220	RP11-69I8.3	1.00E-06	0.10548	-16.68660995
ENSG00000238411	CR381670.1	1.00E-06	0.105601	-16.68826397
ENSG00000278297	CU459202.1	1.00E-06	0.105601	-16.68826397
ENSG00000277372	CU463998.3	1.00E-06	0.105601	-16.68826397
ENSG00000222437	AC118282.3	1.00E-06	0.105601	-16.68826397
ENSG00000258623	CTD-2325P2.3	1.00E-06	0.105658	-16.68904248
ENSG00000233928	RP11-305F18.1	1.00E-06	0.10570377	-16.6896673
ENSG00000275119	RP11-711K1.8	1.00E-06	0.105704	-16.68967045
ENSG00000273141	RP11-820I16.4	1.00E-06	0.105704	-16.68967045

ENSG00000280097	RP11-83B20.7	1.00E-06	0.105911	-16.69249291
ENSG00000258091	RP11-290L1.6	1.00E-06	0.106118	-16.69530986
ENSG00000270518	CTB-159G17.3	1.00E-06	0.106118	-16.69530986
ENSG00000258181	RP11-493L12.4	1.00E-06	0.1061187	-16.69531938
ENSG00000259742	CTD-2014N11.1	1.00E-06	0.106327	-16.69814847
ENSG00000271138	IGLVIVOR22-1	1.00E-06	0.106333	-16.69822988
ENSG00000224100	AP001630.5	1.00E-06	0.106507	-16.70058873
ENSG00000234997	AC016745.3	1.00E-06	0.106536	-16.70098149
ENSG00000228039	KB-1125A3.10	1.00E-06	0.106536	-16.70098149
ENSG00000279527	RP11-83B20.2	1.00E-06	0.106746	-16.70382248
ENSG00000236509	RPL21P133	1.00E-06	0.106876	-16.70557839
ENSG00000218682	AC010150.1	1.00E-06	0.106957	-16.70667138
ENSG00000254451	RP11-560G2.1	1.00E-06	0.107046677	-16.70788048
ENSG00000259267	RP11-432J9.6	1.00E-06	0.107334597	-16.71175564
ENSG00000253582	RP11-649G15.2	1.00E-06	0.107382	-16.71239266
ENSG00000222889	RN7SKP29	1.00E-06	0.107446	-16.71325225
ENSG00000267015	RP11-800A18.4	1.00E-06	0.10781	-16.71813148
ENSG00000272854	RP4-593H12.1	1.00E-06	0.108025	-16.72100571
ENSG00000259336	RP11-323I15.5	1.00E-06	0.10808	-16.72174005
ENSG00000271234	RP5-894D12.4	1.00E-06	0.108241	-16.72388755
ENSG00000203364	RP11-370F5.4	1.00E-06	0.108241	-16.72388755
ENSG00000171496	OR1L8	1.00E-06	0.108241	-16.72388755
ENSG00000176998	HCG4	1.00E-06	0.108458	-16.72677695
ENSG00000271180	RP11-665C16.8	1.00E-06	0.108477	-16.72702966
ENSG00000276533	RP11-139H15.5	1.00E-06	0.108676	-16.72967385
ENSG00000268133	AC003002.4	1.00E-06	0.108762	-16.73081506
ENSG00000258691	RP11-404P21.8	1.00E-06	0.108873684	-16.73229576
ENSG00000272592	RP11-63A2.2	1.00E-06	0.108895	-16.73257819
ENSG00000261304	CTD-2125J1.1	1.00E-06	0.108908	-16.73275041
ENSG00000266149	RP11-789C17.3	1.00E-06	0.108944	-16.73322722
ENSG00000108823	SGCA	1.00E-06	0.109035494	-16.73443833
ENSG00000265799	RP11-387H17.6	1.00E-06	0.1091229	-16.73559437
ENSG00000253796	RP11-1084E5.1	1.00E-06	0.1094144	-16.7394431
ENSG00000255256	RP5-916O11.2	1.00E-06	0.109556	-16.74130897
ENSG00000242941	RPL12P7	1.00E-06	0.109556	-16.74130897
ENSG00000263834	MIR4635	1.00E-06	0.109611	-16.74203306
ENSG00000272627	RP11-354E23.5	1.00E-06	0.109778	-16.74422944
ENSG00000258337	RP11-115H15.2	1.00E-06	0.109778	-16.74422944
ENSG00000258672	RP11-543C4.3	1.00E-06	0.109778	-16.74422944
ENSG00000203886	CYP17A1-AS1	1.00E-06	0.110225	-16.75009195
ENSG00000253168	RP11-326L2.1	1.00E-06	0.11045	-16.75303389
ENSG00000275152	CCL16	1.00E-06	0.11047489	-16.75335897
ENSG00000250697	CTD-2066L21.3	1.00E-06	0.110563264	-16.75451258
ENSG00000249013	FTH1P21	1.00E-06	0.110572	-16.75462657
ENSG00000228033	AC010967.2	1.00E-06	0.110675	-16.75596985
ENSG00000254295	CTC-308K20.2	1.00E-06	0.110675	-16.75596985
ENSG00000184106	TREML3P	1.00E-06	0.110675	-16.75596985
ENSG00000235790	RP11-73M7.6	1.00E-06	0.110732755	-16.75672251

ENSG00000232085	RP11-261C10.4	1.00E-06	0.110902531	-16.75893276
ENSG00000237225	OR13K1P	1.00E-06	0.111016	-16.76040809
ENSG00000174586	ZNF497	1.00E-06	0.111101478	-16.76151848
ENSG00000253739	RNU6-323P	1.00E-06	0.111276	-16.76378294
ENSG00000202024	RNU6-934P	1.00E-06	0.111276	-16.76378294
ENSG00000258249	RP11-497G19.3	1.00E-06	0.111359	-16.76485864
ENSG00000228467	RP11-402N8.1	1.00E-06	0.111359	-16.76485864
ENSG00000231838	RP11-449H15.2	1.00E-06	0.111359	-16.76485864
ENSG00000164049	FBXW12	1.00E-06	0.111375032	-16.76506633
ENSG00000277966	HOXA11-AS1_4	1.00E-06	0.111493	-16.76659361
ENSG00000199357	Y_RNA	1.00E-06	0.111589	-16.76783529
ENSG00000254178	RP11-299D14.2	1.00E-06	0.111589	-16.76783529
ENSG00000231073	RP11-316M1.3	1.00E-06	0.111771	-16.77018639
ENSG00000273044	RP4-569D19.8	1.00E-06	0.11182	-16.77081872
ENSG00000244712	RP11-874G11.1	1.00E-06	0.111973	-16.77279137
ENSG00000237135	DDX10P1	1.00E-06	0.111993	-16.77304904
ENSG00000262516	RP11-95J11.1	1.00E-06	0.112051	-16.773796
ENSG00000231604	RP5-897D18.1	1.00E-06	0.112051	-16.773796
ENSG00000227649	MTND6P32	1.00E-06	0.112051	-16.773796
ENSG00000256314	RP11-338E21.3	1.00E-06	0.112283	-16.77677999
ENSG00000249167	CTB-118N6.2	1.00E-06	0.112283	-16.77677999
ENSG00000218521	RP11-420A21.1	1.00E-06	0.112283	-16.77677999
ENSG00000254152	CTD-3107M8.2	1.00E-06	0.112283	-16.77677999
ENSG00000253744	AC025442.3	1.00E-06	0.112517	-16.77978347
ENSG00000254351	ARL2BPP5	1.00E-06	0.112517	-16.77978347
ENSG00000230587	AC093609.1	1.00E-06	0.112562	-16.78036034
ENSG00000271392	RP1-161P9.5	1.00E-06	0.112751	-16.7827807
ENSG00000235432	RP5-930J4.5	1.00E-06	0.112986	-16.7857845
ENSG00000224907	RP11-451M19.2	1.00E-06	0.112986	-16.7857845
ENSG00000242488	RP11-270M14.1	1.00E-06	0.112986	-16.7857845
ENSG00000268480	CTD-2586B10.1	1.00E-06	0.113223	-16.78880753
ENSG00000227824	AC122136.2	1.00E-06	0.113223	-16.78880753
ENSG00000248489	CTD-2007H13.3	1.00E-06	0.113223	-16.78880753
ENSG00000143119	CD53	1.00E-06	0.113226813	-16.78885612
ENSG00000228384	AC007040.6	1.00E-06	0.11346	-16.79182424
ENSG00000224019	RPL21P32	1.00E-06	0.11346	-16.79182424
ENSG00000227070	RP11-191G24.1	1.00E-06	0.113699181	-16.79486233
ENSG00000134020	PEBP4	1.00E-06	0.113934921	-16.79785047
ENSG00000229427	ANKRD26P4	1.00E-06	0.113938	-16.79788946
ENSG00000266901	RP11-567M16.5	1.00E-06	0.114178	-16.80092517
ENSG00000251085	RP11-893F2.6	1.00E-06	0.114189	-16.80106415
ENSG00000232490	OSBPL10-AS1	1.00E-06	0.114246731	-16.80179336
ENSG00000279954	WI2-85898F10.2	1.00E-06	0.114247	-16.80179676
ENSG00000257530	RP11-843B15.2	1.00E-06	0.114399	-16.80371492
ENSG00000235544	RPS11P1	1.00E-06	0.114419	-16.80396712
ENSG00000230437	RP11-102J14.1	1.00E-06	0.114419	-16.80396712
ENSG00000229954	MTND2P2	1.00E-06	0.114541	-16.80550458
ENSG00000228995	MTND1P9	1.00E-06	0.114662	-16.80702782

ENSG00000253194	RP11-351A11.1	1.00E-06	0.114905	-16.81008205
ENSG00000271225	BNIP3P4	1.00E-06	0.114947	-16.81060929
ENSG00000254286	RP11-89K10.1	1.00E-06	0.114953012	-16.81068474
ENSG00000230988	RPL23AP11	1.00E-06	0.115396	-16.81623369
ENSG00000223882	ABCC5-AS1	1.00E-06	0.115396	-16.81623369
ENSG00000281046	Metazoa_SRP	1.00E-06	0.115404	-16.8163337
ENSG00000182586	LINC00334	1.00E-06	0.115585624	-16.81860245
ENSG00000273848	MIR7847	1.00E-06	0.115597	-16.81874443
ENSG00000261399	LA16c-329F2.1	1.00E-06	0.115643	-16.81931842
ENSG00000283108	CTC-325H20.8	1.00E-06	0.1157664	-16.82085706
ENSG00000257078	RP3-461F17.2	1.00E-06	0.115814	-16.82145014
ENSG00000272922	RP11-329B9.5	1.00E-06	0.115889	-16.82238411
ENSG00000267005	AC002984.2	1.00E-06	0.115973	-16.82342944
ENSG00000279306	RP5-915N17.11	1.00E-06	0.116014	-16.82393939
ENSG00000238054	RP11-316I3.2	1.00E-06	0.116138	-16.82548057
ENSG00000229753	RP11-28P17.3	1.00E-06	0.116138	-16.82548057
ENSG00000268754	RP11-514D23.2	1.00E-06	0.116388	-16.82858279
ENSG00000233109	RP11-22C8.1	1.00E-06	0.116388	-16.82858279
ENSG00000238031	AC090505.1	1.00E-06	0.116639	-16.83169073
ENSG00000241187	CTC-209L16.1	1.00E-06	0.116639	-16.83169073
ENSG00000272855	RP5-1102E8.3	1.00E-06	0.116891	-16.83480433
ENSG00000231449	AC097359.2	1.00E-06	0.116891	-16.83480433
ENSG00000224667	AC013470.8	1.00E-06	0.117144	-16.83792354
ENSG00000266987	RP11-354P11.8	1.00E-06	0.117261	-16.83936374
ENSG00000116544	DLGAP3	1.00E-06	0.117576489	-16.84324007
ENSG00000282527	RP11-389G6.5	1.00E-06	0.117654	-16.84419084
ENSG00000234999	SNRPCP19	1.00E-06	0.117654	-16.84419084
ENSG00000254653	RP11-702F3.1	1.00E-06	0.118081	-16.84941732
ENSG00000200885	RNU1-146P	1.00E-06	0.118081	-16.84941732
ENSG00000154252	GAL3ST2	1.00E-06	0.118117	-16.84985709
ENSG00000258485	SRMP2	1.00E-06	0.118167	-16.85046767
ENSG00000244232	RN7SL698P	1.00E-06	0.118277	-16.85181003
ENSG00000265403	AC015884.1	1.00E-06	0.11831	-16.8522125
ENSG00000264715	AC104996.1	1.00E-06	0.11831	-16.8522125
ENSG00000265295	AC084125.1	1.00E-06	0.11831	-16.8522125
ENSG00000277611	RP1-138B7.6	1.00E-06	0.118641	-16.85624314
ENSG00000015413	DPEP1	1.00E-06	0.118641777	-16.85625259
ENSG00000231397	AC004941.5	1.00E-06	0.118685	-16.85677809
ENSG00000230839	RP5-968J1.1	1.00E-06	0.118878	-16.85912222
ENSG00000129596	CDO1	1.00E-06	0.119169243	-16.8626524
ENSG00000233256	RP11-445K13.2	1.00E-06	0.119208	-16.86312153
ENSG00000178882	FAM101A	1.00E-06	0.119396573	-16.8654019
ENSG00000278942	LA16c-390H2.1	1.00E-06	0.119978	-16.87241036
ENSG00000182393	IFNL1	1.00E-06	0.119987	-16.87251858
ENSG00000226627	SHANK2-AS1	1.00E-06	0.120161	-16.8746092
ENSG00000241129	RPL22P19	1.00E-06	0.120267549	-16.87588789
ENSG00000235266	RP11-753C18.8	1.00E-06	0.120268	-16.87589331
ENSG00000225513	RP11-165N19.2	1.00E-06	0.120268	-16.87589331

ENSG00000281731	RP11-384K6.8	1.00E-06	0.120475708	-16.87838276
ENSG00000227773	ASH1L-IT1	1.00E-06	0.120805	-16.88232064
ENSG00000259560	RP11-648K4.2	1.00E-06	0.120805	-16.88232064
ENSG00000233060	AC016700.2	1.00E-06	0.120805	-16.88232064
ENSG00000265897	AC018696.1	1.00E-06	0.120805	-16.88232064
ENSG00000282602	CTA-286B10.7	1.00E-06	0.120805	-16.88232064
ENSG00000258858	RP11-982M15.5	1.00E-06	0.12094	-16.88393196
ENSG00000237300	MTCO1P19	1.00E-06	0.120976	-16.88436134
ENSG00000258068	RP11-328C8.5	1.00E-06	0.121075	-16.88554148
ENSG00000152592	DMP1	1.00E-06	0.121075	-16.88554148
ENSG00000217512	RP11-477E3.2	1.00E-06	0.121075	-16.88554148
ENSG00000279087	RP11-83B20.10	1.00E-06	0.121347	-16.88877892
ENSG00000244146	RP11-366M4.2	1.00E-06	0.121347	-16.88877892
ENSG00000270393	AC000095.9	1.00E-06	0.121369	-16.88904045
ENSG00000266109	MIR4440	1.00E-06	0.121495	-16.89053742
ENSG00000255340	RP11-484D2.5	1.00E-06	0.121894	-16.89526759
ENSG00000230058	RP11-172E9.2	1.00E-06	0.121894	-16.89526759
ENSG00000229453	SPINK8	1.00E-06	0.121894	-16.89526759
ENSG00000254261	RP11-18A15.1	1.00E-06	0.122168	-16.89850692
ENSG00000273855	RP11-133K1.12	1.00E-06	0.122444	-16.90176256
ENSG00000233975	RP11-288L9.1	1.00E-06	0.122723	-16.90504613
ENSG00000280013	CH507-42P11.2	1.00E-06	0.122883	-16.90692582
ENSG00000213527	RP11-591L14.1	1.00E-06	0.122933	-16.90751272
ENSG00000273455	RP11-305O4.3	1.00E-06	0.123281	-16.91159094
ENSG00000253695	RP11-177H2.2	1.00E-06	0.123281	-16.91159094
ENSG00000278705	HIST1H4B	1.00E-06	0.123563	-16.91488728
ENSG00000265110	MIR4731	1.00E-06	0.123704	-16.91653263
ENSG00000254420	RP11-452H21.1	1.00E-06	0.123845	-16.9181761
ENSG00000267535	LINC00868	1.00E-06	0.124058951	-16.92066631
ENSG00000254287	RP11-44K6.4	1.00E-06	0.124075	-16.92085293
ENSG00000227673	RP11-243J18.2	1.00E-06	0.124098	-16.92112034
ENSG00000238263	RP11-402L1.12	1.00E-06	0.12413	-16.92149231
ENSG00000184111	RP11-366I13.3	1.00E-06	0.12413	-16.92149231
ENSG00000232455	LARS2-AS1	1.00E-06	0.124242	-16.92279343
ENSG00000260865	CTA-331F8.1	1.00E-06	0.124311	-16.92359444
ENSG00000270075	RP11-127L20.5	1.00E-06	0.124424	-16.92490527
ENSG00000236263	RP11-263K19.6	1.00E-06	0.1246	-16.92694454
ENSG00000235808	MYL6P2	1.00E-06	0.124701	-16.92811351
ENSG00000235191	NUCB1-AS1	1.00E-06	0.124989	-16.93144161
ENSG00000227616	AC063976.1	1.00E-06	0.125021	-16.93181092
ENSG00000217783	LDHAL6FP	1.00E-06	0.125048	-16.93212246
ENSG00000266318	MIR5692A1	1.00E-06	0.125497	-16.93729335
ENSG00000266082	AL138925.1	1.00E-06	0.125862	-16.94148325
ENSG00000265801	RP11-720N19.2	1.00E-06	0.125862	-16.94148325
ENSG00000264365	RP11-621L6.2	1.00E-06	0.125995	-16.94300696
ENSG00000259415	RP11-7M10.2	1.00E-06	0.126155	-16.94483786
ENSG00000171860	C3AR1	1.00E-06	0.12615511	-16.94483912
ENSG00000231542	TAB3-AS1	1.00E-06	0.126303	-16.94652938

ENSG00000237409	RP11-226L15.1	1.00E-06	0.126449	-16.9481961
ENSG00000269720	CTD-2521M24.5	1.00E-06	0.126449	-16.9481961
ENSG00000226217	RPL19P1	1.00E-06	0.126449	-16.9481961
ENSG00000272646	RP11-188P17.2	1.00E-06	0.126746	-16.95158069
ENSG00000233662	CALM2P4	1.00E-06	0.127044	-16.95496872
ENSG00000278313	RP11-1006G14.5	1.00E-06	0.127044	-16.95496872
ENSG00000146453	PNLDC1	1.00E-06	0.127296184	-16.95782965
ENSG00000271398	RP5-1092A3.5	1.00E-06	0.127342	-16.9583488
ENSG00000213126	AC092642.1	1.00E-06	0.127342	-16.9583488
ENSG00000250140	CTD-2517O10.5	1.00E-06	0.127342	-16.9583488
ENSG00000234181	RP11-182N22.8	1.00E-06	0.127342	-16.9583488
ENSG00000249854	RP11-420O16.1	1.00E-06	0.127643	-16.9617549
ENSG00000216642	RP1-95L4.3	1.00E-06	0.127844	-16.96402493
ENSG00000208024	MIR199A2	1.00E-06	0.127921	-16.9648936
ENSG00000237852	RP4-630A11.3	1.00E-06	0.127944	-16.96515297
ENSG00000230470	GS1-115G20.1	1.00E-06	0.127944	-16.96515297
ENSG00000264105	MIR3688-2	1.00E-06	0.128028	-16.96609984
ENSG00000225316	LINC00350	1.00E-06	0.128247	-16.96856555
ENSG00000188163	FAM166A	1.00E-06	0.128273226	-16.96886055
ENSG00000220347	RP11-230C9.1	1.00E-06	0.128327	-16.96946522
ENSG00000282996	RP11-351M16.4	1.00E-06	0.1284	-16.97028568
ENSG00000218813	RP11-59D5__B.3	1.00E-06	0.1284	-16.97028568
ENSG00000258048	RP11-530C5.1	1.00E-06	0.128422	-16.97053285
ENSG00000236538	ZNF863P	1.00E-06	0.128488	-16.9712741
ENSG00000276972	RP11-116D17.5	1.00E-06	0.128553	-16.97200375
ENSG00000277597	RP11-961A15.3	1.00E-06	0.12866	-16.97320407
ENSG00000239983	RP11-79E3.1	1.00E-06	0.128785	-16.97460504
ENSG00000274719	RP11-86K22.2	1.00E-06	0.128859	-16.97543378
ENSG00000243518	RP11-639F1.2	1.00E-06	0.128859	-16.97543378
ENSG00000265764	AL121932.1	1.00E-06	0.128859	-16.97543378
ENSG00000226254	PTP4A1P3	1.00E-06	0.128859	-16.97543378
ENSG00000125571	IL37	1.00E-06	0.129012334	-16.97714947
ENSG00000261823	RP11-48G14.1	1.00E-06	0.129013	-16.97715692
ENSG00000249166	RP11-1C1.6	1.00E-06	0.129107	-16.9782077
ENSG00000266111	RP11-296K13.4	1.00E-06	0.129166	-16.97886684
ENSG00000280148	RP3-468K18.7	1.00E-06	0.129219	-16.97945869
ENSG00000259276	RP11-815J21.3	1.00E-06	0.129264	-16.97996102
ENSG00000224796	RPL32P1	1.00E-06	0.129361	-16.98104321
ENSG00000227356	LINC00866	1.00E-06	0.129475	-16.98231403
ENSG00000275645	RP11-817O13.9	1.00E-06	0.129475	-16.98231403
ENSG00000267486	GLUD1P4	1.00E-06	0.129475	-16.98231403
ENSG00000268536	AC005523.3	1.00E-06	0.129475	-16.98231403
ENSG00000279465	RP11-44D5.2	1.00E-06	0.129561	-16.98327198
ENSG00000280221	AC008686.1	1.00E-06	0.129664	-16.98441846
ENSG00000271983	RP11-28H5.2	1.00E-06	0.129802	-16.98595309
ENSG00000262495	RP11-46I8.1	1.00E-06	0.130097	-16.98922817
ENSG00000225337	RP11-561O23.7	1.00E-06	0.130097	-16.98922817
ENSG00000269792	CTB-60E11.9	1.00E-06	0.130435	-16.99297152

ENSG00000214374	RPLP2P1	1.00E-06	0.130526	-16.99397769
ENSG00000248202	RP11-455B3.1	1.00E-06	0.130567871	-16.99444041
ENSG00000213262	VDAC2P3	1.00E-06	0.130726	-16.99618658
ENSG00000224220	AC104699.1	1.00E-06	0.130726	-16.99618658
ENSG00000266718	RP11-466A19.1	1.00E-06	0.130937	-16.9985133
ENSG00000255148	RP4-791M13.4	1.00E-06	0.130966	-16.9988328
ENSG00000257754	RP11-650K20.2	1.00E-06	0.131042	-16.99966976
ENSG00000263098	RP11-567O16.1	1.00E-06	0.131042	-16.99966976
ENSG00000223911	AC009480.3	1.00E-06	0.13136	-17.00316651
ENSG00000249131	CTB-35F21.2	1.00E-06	0.13136	-17.00316651
ENSG00000183733	FIGLA	1.00E-06	0.131507	-17.00478007
ENSG00000261529	RP13-487P22.1	1.00E-06	0.131672	-17.00658906
ENSG00000279931	RP11-13J12.2	1.00E-06	0.131681	-17.00668767
ENSG00000248545	RP11-302F12.3	1.00E-06	0.131681	-17.00668767
ENSG00000226453	RP11-379B8.1	1.00E-06	0.131681	-17.00668767
ENSG00000264963	RN7SL440P	1.00E-06	0.13254	-17.0160683
ENSG00000154316	TDH	1.00E-06	0.132586751	-17.0165771
ENSG00000273980	RP13-49I15.6	1.00E-06	0.132648	-17.0172434
ENSG00000228122	MTND1P17	1.00E-06	0.132648	-17.0172434
ENSG00000223815	DIAPH3-AS2	1.00E-06	0.132721	-17.01803714
ENSG00000260308	RP11-104C4.4	1.00E-06	0.132856	-17.01950386
ENSG00000260500	CTD-3193O13.1	1.00E-06	0.132886	-17.01982959
ENSG00000223716	RP11-113O24.3	1.00E-06	0.132928	-17.0202855
ENSG00000215184	RP11-8L18.2	1.00E-06	0.132974	-17.02078466
ENSG00000230618	H3F3AP3	1.00E-06	0.132974	-17.02078466
ENSG00000227053	RP11-395B7.4	1.00E-06	0.132974	-17.02078466
ENSG00000260616	RP11-327F22.4	1.00E-06	0.132974962	-17.0207951
ENSG00000170590	LINC00266-3	1.00E-06	0.133112	-17.02228111
ENSG00000260046	RP11-454K7.3	1.00E-06	0.13322	-17.02345116
ENSG00000254913	RP4-791M13.5	1.00E-06	0.133302	-17.0243389
ENSG00000277797	RP11-244G12.1	1.00E-06	0.133559	-17.02711767
ENSG00000256944	RP11-881M11.1	1.00E-06	0.133631	-17.0278952
ENSG00000217527	RPS16P5	1.00E-06	0.133961	-17.03145353
ENSG00000241941	RPL32P26	1.00E-06	0.134294	-17.03503532
ENSG00000263311	RP11-498D10.8	1.00E-06	0.134294	-17.03503532
ENSG00000279182	XX-C00717C00720L.1	1.00E-06	0.134299	-17.03508904
ENSG00000260892	CTD-2026K11.1	1.00E-06	0.134438	-17.03658146
ENSG00000236896	RP11-535C21.3	1.00E-06	0.134628	-17.03861897
ENSG00000279276	CTA-345G4.1	1.00E-06	0.134964	-17.04221511
ENSG00000278071	RP11-736N17.10	1.00E-06	0.135127	-17.04395645
ENSG00000248152	RP11-650J17.1	1.00E-06	0.135301	-17.04581298
ENSG00000232124	AP001057.1	1.00E-06	0.13547	-17.04761387
ENSG00000258746	RP11-454K7.2	1.00E-06	0.13564	-17.04942316
ENSG00000207432	U8	1.00E-06	0.13564	-17.04942316
ENSG00000271329	RP5-891H21.5	1.00E-06	0.135981	-17.05304556
ENSG00000277852	RP11-421K23.1	1.00E-06	0.135981	-17.05304556
ENSG00000255456	CTD-2517M22.9	1.00E-06	0.135981	-17.05304556
ENSG00000260583	AP000223.42	1.00E-06	0.13603	-17.05356533

ENSG00000232173	OR2H5P	1.00E-06	0.136079	-17.05408492
ENSG00000224391	LINC01280	1.00E-06	0.136216	-17.05553665
ENSG00000224064	RP11-344H11.5	1.00E-06	0.136324	-17.05668005
ENSG00000241950	RPL29P23	1.00E-06	0.136438	-17.05788599
ENSG00000237249	RP1-102G20.2	1.00E-06	0.137014	-17.06396379
ENSG00000271981	RP11-573G6.8	1.00E-06	0.137014	-17.06396379
ENSG00000231228	CTD-2593A12.4	1.00E-06	0.137297	-17.06694058
ENSG00000239215	RPL27P12	1.00E-06	0.137362	-17.06762342
ENSG00000267808	AC018755.17	1.00E-06	0.137362	-17.06762342
ENSG00000231729	ARHGEF9-IT1	1.00E-06	0.137362	-17.06762342
ENSG00000104435	STMN2	1.00E-06	0.137478715	-17.06884875
ENSG00000225549	RP11-210H10__A.1	1.00E-06	0.137711	-17.07128428
ENSG00000201343	Y_RNA	1.00E-06	0.137762	-17.07181847
ENSG00000242634	RPS24P16	1.00E-06	0.138063	-17.07496721
ENSG00000279957	RP11-141B14.2	1.00E-06	0.138415	-17.07864077
ENSG00000213291	RP11-420H19.3	1.00E-06	0.138549	-17.08003677
ENSG00000101336	HCK	1.00E-06	0.13862662	-17.0808448
ENSG00000211716	TRBV9	1.00E-06	0.13877	-17.08233619
ENSG00000162877	PM20D1	1.00E-06	0.139165578	-17.08644288
ENSG00000237556	KCND3-AS1	1.00E-06	0.139306987	-17.08790809
ENSG00000234273	AC073071.1	1.00E-06	0.139486	-17.0897608
ENSG00000248188	RP11-442P12.2	1.00E-06	0.139846	-17.09347946
ENSG00000229822	RP5-1174J21.2	1.00E-06	0.139846	-17.09347946
ENSG00000229014	RPL30P13	1.00E-06	0.139967	-17.0947272
ENSG00000267446	CTB-39G8.2	1.00E-06	0.140209	-17.09721943
ENSG00000241525	AC108004.3	1.00E-06	0.140254534	-17.09768789
ENSG00000256827	RP11-214K3.5	1.00E-06	0.140256	-17.09770296
ENSG00000258781	RP11-496I2.4	1.00E-06	0.140377	-17.09894705
ENSG00000253111	RP11-136O12.2	1.00E-06	0.140751445	-17.10279021
ENSG00000255491	RP11-1082L8.4	1.00E-06	0.141031	-17.10565279
ENSG00000186008	RPS4XP21	1.00E-06	0.141435	-17.10977965
ENSG00000206772	RNU6-44P	1.00E-06	0.141624	-17.11170624
ENSG00000255174	RP11-481A20.4	1.00E-06	0.141677	-17.11224604
ENSG00000236209	AC104135.2	1.00E-06	0.141863	-17.11413884
ENSG00000228453	RPS15AP9	1.00E-06	0.142049	-17.11602915
ENSG00000280006	AC009060.2	1.00E-06	0.142049	-17.11602915
ENSG00000230273	AC087163.3	1.00E-06	0.142049	-17.11602915
ENSG00000231069	BX842568.4	1.00E-06	0.142174	-17.11729813
ENSG00000264421	RP11-401F2.4	1.00E-06	0.142423	-17.11982262
ENSG00000225728	RP6-191P20.3	1.00E-06	0.142423	-17.11982262
ENSG00000248607	RP11-158I23.1	1.00E-06	0.142798	-17.12361625
ENSG00000134200	TSHB	1.00E-06	0.143112	-17.12678512
ENSG00000265118	CTD-2370N5.3	1.00E-06	0.1431213	-17.12687887
ENSG00000173239	LIPM	1.00E-06	0.143270839	-17.12838547
ENSG00000250378	RP11-119J18.1	1.00E-06	0.143556	-17.1312541
ENSG00000199550	Y_RNA	1.00E-06	0.143682	-17.13251981
ENSG00000242995	RP11-1085N6.1	1.00E-06	0.143792	-17.13362389
ENSG00000280250	RP11-6L6.6	1.00E-06	0.143938	-17.13508799

ENSG00000278708	HOTTIP_2	1.00E-06	0.143938	-17.13508799
ENSG00000280170	AP000687.1	1.00E-06	0.144321	-17.13892171
ENSG00000222515	RN7SKP240	1.00E-06	0.144321	-17.13892171
ENSG00000230065	AC010974.3	1.00E-06	0.144707	-17.14277519
ENSG00000242628	AC009228.1	1.00E-06	0.145011445	-17.14580724
ENSG00000244167	RP4-733B9.1	1.00E-06	0.145095	-17.14663828
ENSG00000275489	C17orf98	1.00E-06	0.145578	-17.15143282
ENSG00000218454	HNRNPA1P19	1.00E-06	0.146272	-17.1582941
ENSG00000230747	AC021188.4	1.00E-06	0.146272	-17.1582941
ENSG00000265750	RP11-799B12.4	1.00E-06	0.14653	-17.16083654
ENSG00000196433	ASMT	1.00E-06	0.146588139	-17.16140885
ENSG00000267231	AC005786.5	1.00E-06	0.146668	-17.16219461
ENSG00000250882	CTC-459M5.1	1.00E-06	0.146847	-17.16395427
ENSG00000252132	RNU6-795P	1.00E-06	0.147123	-17.16666328
ENSG00000271710	NDUFA8P1	1.00E-06	0.147468	-17.1700424
ENSG00000251129	RP11-734I18.1	1.00E-06	0.147468	-17.1700424
ENSG00000226890	LA16c-395F10.1	1.00E-06	0.14805	-17.17572496
ENSG00000249096	RP11-290F5.1	1.00E-06	0.148650497	-17.18156476
ENSG00000230947	AP000356.2	1.00E-06	0.148683	-17.18188018
ENSG00000273068	CTA-714B7.7	1.00E-06	0.148683	-17.18188018
ENSG00000230290	ARMC2-AS1	1.00E-06	0.148683	-17.18188018
ENSG00000242102	RN7SL152P	1.00E-06	0.148922	-17.18419737
ENSG00000218198	RPS20P32	1.00E-06	0.148983	-17.18478819
ENSG00000276704	RP11-128N14.4	1.00E-06	0.149092	-17.18584332
ENSG00000259701	RP11-108K3.3	1.00E-06	0.149092	-17.18584332
ENSG00000230702	RP11-681L4.1	1.00E-06	0.149918	-17.19381409
ENSG00000226229	RPLP0P1	1.00E-06	0.149918	-17.19381409
ENSG00000224986	PPP1R8P1	1.00E-06	0.150335	-17.1978214
ENSG00000225965	RP11-508N22.6	1.00E-06	0.150335	-17.1978214
ENSG00000279360	RP11-946P6.4	1.00E-06	0.150335	-17.1978214
ENSG00000231301	RPL13AP	1.00E-06	0.150451	-17.19893417
ENSG00000215812	ZNF847P	1.00E-06	0.150754	-17.20183676
ENSG00000237605	RP11-343H5.6	1.00E-06	0.151175	-17.20586005
ENSG00000178107	RP11-998D10.4	1.00E-06	0.151175	-17.20586005
ENSG00000250488	RP11-6C14.1	1.00E-06	0.151175	-17.20586005
ENSG00000241985	WWTR1-IT1	1.00E-06	0.151202	-17.2061177
ENSG00000280167	RP11-867G2.4	1.00E-06	0.151598	-17.20989119
ENSG00000264113	RN7SL784P	1.00E-06	0.151917	-17.2129238
ENSG00000226705	SDCBPP1	1.00E-06	0.152024	-17.21393957
ENSG00000231207	RP1-144C9.2	1.00E-06	0.152452	-17.21799555
ENSG00000251399	RP11-234K19.1	1.00E-06	0.152452	-17.21799555
ENSG00000228045	DNAJA1P6	1.00E-06	0.152883	-17.22206847
ENSG00000233601	NCOR1P3	1.00E-06	0.153099	-17.22410533
ENSG00000228289	RP4-640E24.1	1.00E-06	0.153316	-17.22614874
ENSG00000259926	RP11-44F14.5	1.00E-06	0.153316	-17.22614874
ENSG00000223678	RP11-311H10.4	1.00E-06	0.153316	-17.22614874
ENSG00000266905	RP11-1058N17.1	1.00E-06	0.153751	-17.23023627
ENSG00000263749	OOSP1P2	1.00E-06	0.154189	-17.23434032

ENSG00000221520	MIR1285-1	1.00E-06	0.15463	-17.23846072
ENSG00000273407	RP11-757A13.1	1.00E-06	0.15463	-17.23846072
ENSG00000235802	HCFC1-AS1	1.00E-06	0.15463	-17.23846072
ENSG00000129824	RPS4Y1	1.00E-06	0.154630167	-17.23846228
ENSG00000281186	LINC00706	1.00E-06	0.154852	-17.24053049
ENSG00000249451	RP11-94C24.6	1.00E-06	0.155073	-17.24258799
ENSG00000270324	RP11-180P8.4	1.00E-06	0.155519	-17.24673132
ENSG00000279599	AC005514.2	1.00E-06	0.156117	-17.25226812
ENSG00000272138	LINC01607	1.00E-06	0.156393888	-17.25482461
ENSG00000272510	RP4-680D5.8	1.00E-06	0.156418	-17.25504702
ENSG00000223787	RP4-593M8.1	1.00E-06	0.156418	-17.25504702
ENSG00000251532	CTD-2245E15.3	1.00E-06	0.156418	-17.25504702
ENSG00000280440	AC002064.7	1.00E-06	0.156418	-17.25504702
ENSG00000237663	DNAJC19P7	1.00E-06	0.157327	-17.26340676
ENSG00000255369	RP11-740D6.3	1.00E-06	0.157327	-17.26340676
ENSG00000244294	RN7SL740P	1.00E-06	0.157327	-17.26340676
ENSG00000269235	ZNF350-AS1	1.00E-06	0.157363997	-17.26374598
ENSG00000248295	DDX43P1	1.00E-06	0.157787	-17.26761882
ENSG00000229032	RP11-91A18.1	1.00E-06	0.158247	-17.27181862
ENSG00000225928	CACYBPP1	1.00E-06	0.158247	-17.27181862
ENSG00000254450	ALG9-IT1	1.00E-06	0.158247	-17.27181862
ENSG00000261419	RP11-57A19.4	1.00E-06	0.158647	-17.27546071
ENSG00000273553	CTD-2049O4.2	1.00E-06	0.158712	-17.27605169
ENSG00000270384	U1	1.00E-06	0.158797	-17.27682413
ENSG00000267436	AC005786.7	1.00E-06	0.158894	-17.27770512
ENSG00000276601	Metazoa_SRP	1.00E-06	0.158955	-17.27825887
ENSG00000200674	RN7SKP160	1.00E-06	0.159178	-17.28028143
ENSG00000263540	MIR5582	1.00E-06	0.159178	-17.28028143
ENSG00000218337	RP4-724P12.1	1.00E-06	0.159178	-17.28028143
ENSG00000229502	RP11-52J3.2	1.00E-06	0.15933441	-17.28169834
ENSG00000280224	CTA-722E9.1	1.00E-06	0.159491	-17.28311549
ENSG00000279061	RP3-332O11.2	1.00E-06	0.159648	-17.28453495
ENSG00000256812	CAPNS2	1.00E-06	0.159805	-17.28595302
ENSG00000223754	AC008073.9	1.00E-06	0.159935	-17.28712617
ENSG00000183249	NF1P3	1.00E-06	0.160357	-17.29092781
ENSG00000218153	KRT18P22	1.00E-06	0.160714	-17.29413608
ENSG00000175319	NF1P5	1.00E-06	0.161074	-17.29736411
ENSG00000263940	RN7SL275P	1.00E-06	0.161554	-17.30165695
ENSG00000278825	RN7SL640P	1.00E-06	0.161739	-17.30330807
ENSG00000273717	RN7SL343P	1.00E-06	0.161739	-17.30330807
ENSG00000276522	RN7SL462P	1.00E-06	0.161739	-17.30330807
ENSG00000281439	Metazoa_SRP	1.00E-06	0.161967	-17.30534038
ENSG00000260444	RP11-483E23.7	1.00E-06	0.162038	-17.30597266
ENSG00000258044	RP11-530C5.2	1.00E-06	0.16228	-17.30812568
ENSG00000240589	RN7SL258P	1.00E-06	0.162362	-17.30885449
ENSG00000238007	AC024619.2	1.00E-06	0.162394721	-17.30914521
ENSG00000255309	RP11-756D7.1	1.00E-06	0.162524	-17.31029325
ENSG00000222998	RN7SKP259	1.00E-06	0.162524	-17.31029325

ENSG00000134007	ADAM20	1.00E-06	0.162652	-17.31142904
ENSG00000277382	RP11-318A15.8	1.00E-06	0.16274	-17.31220937
ENSG00000237687	LINC00686	1.00E-06	0.163013	-17.3146275
ENSG00000277324	RP11-850A17.1	1.00E-06	0.163359	-17.31768641
ENSG00000234108	RP11-115A15.2	1.00E-06	0.163506	-17.31898405
ENSG00000261070	RP11-554A11.8	1.00E-06	0.164001	-17.32334509
ENSG00000235619	RPL36AP33	1.00E-06	0.164108	-17.32428604
ENSG00000275680	Metazoa_SRP	1.00E-06	0.1645	-17.32772806
ENSG00000214772	RP11-174G6.1	1.00E-06	0.164751	-17.3299277
ENSG00000203469	RP5-1113E3.3	1.00E-06	0.164981	-17.33194036
ENSG00000253015	RN7SKP64	1.00E-06	0.165002	-17.33212399
ENSG00000279795	RP11-586K12.1	1.00E-06	0.165169	-17.33358341
ENSG00000255003	CYCSP28	1.00E-06	0.165228	-17.33409867
ENSG00000258908	RP11-203M5.8	1.00E-06	0.165312	-17.33483193
ENSG00000176378	PFN1P10	1.00E-06	0.165506	-17.33652399
ENSG00000258871	RP3-514A23.2	1.00E-06	0.165506	-17.33652399
ENSG00000237306	HSPE1P22	1.00E-06	0.165506	-17.33652399
ENSG00000264083	RP11-227G15.9	1.00E-06	0.165668	-17.33793544
ENSG00000259631	RP11-557C18.4	1.00E-06	0.166014	-17.34094538
ENSG00000187166	H1FNT	1.00E-06	0.166525	-17.34537926
ENSG00000241869	RN7SL363P	1.00E-06	0.166525	-17.34537926
ENSG00000272407	RP3-511B24.5	1.00E-06	0.16681	-17.34784625
ENSG00000258007	RP11-350G24.1	1.00E-06	0.167123	-17.35055077
ENSG00000269445	AC067969.1	1.00E-06	0.167288	-17.35197444
ENSG00000265388	RN7SL391P	1.00E-06	0.167698	-17.35550596
ENSG00000240739	RP11-69M1.1	1.00E-06	0.168375	-17.36131842
ENSG00000252941	RNA5SP340	1.00E-06	0.1686	-17.36324501
ENSG00000277235	RP4-550H1.7	1.00E-06	0.1686	-17.36324501
ENSG00000231293	RPL36AP6	1.00E-06	0.1686	-17.36324501
ENSG00000259359	RP11-327J17.2	1.00E-06	0.168631348	-17.36351322
ENSG00000199075	MIR26A1	1.00E-06	0.168687	-17.36398927
ENSG00000267235	ZNF861P	1.00E-06	0.168924	-17.36601479
ENSG00000280598	Y_RNA	1.00E-06	0.169127	-17.36774747
ENSG00000281621	Y_RNA	1.00E-06	0.169127	-17.36774747
ENSG00000271589	RP11-130N24.2	1.00E-06	0.169127	-17.36774747
ENSG00000237141	DNAJC19P1	1.00E-06	0.169657	-17.37226143
ENSG00000243642	RN7SL526P	1.00E-06	0.169657	-17.37226143
ENSG00000202341	RNU6-1051P	1.00E-06	0.16979	-17.37339197
ENSG00000271410	RP11-506H20.2	1.00E-06	0.170016	-17.375311
ENSG00000201581	RN7SKP78	1.00E-06	0.170191	-17.37679522
ENSG00000263105	RP11-95P2.3	1.00E-06	0.170191	-17.37679522
ENSG00000229623	METTL21AP1	1.00E-06	0.170458	-17.37905678
ENSG00000212024	MIR550A3	1.00E-06	0.170907	-17.38285196
ENSG00000240439	RN7SL91P	1.00E-06	0.171103	-17.38450553
ENSG00000241745	RN7SL788P	1.00E-06	0.171268	-17.3858961
ENSG00000261561	RP11-536P16.2	1.00E-06	0.171268	-17.3858961
ENSG00000273313	RBAKDN	1.00E-06	0.171445	-17.3873863
ENSG00000266268	RP11-172F10.1	1.00E-06	0.171538864	-17.38817594

ENSG00000273155	RP11-38C17.1	1.00E-06	0.171682	-17.38937926
ENSG00000255749	GNAI2P1	1.00E-06	0.171811	-17.39046288
ENSG00000217195	RP11-277I20.2	1.00E-06	0.171811	-17.39046288
ENSG00000262141	CTC-479C5.11	1.00E-06	0.172359	-17.39505711
ENSG00000258591	RP11-545M17.3	1.00E-06	0.172516	-17.39637065
ENSG00000260084	RP11-615I2.1	1.00E-06	0.172774	-17.3985266
ENSG00000241307	RP11-674E16.1	1.00E-06	0.172898	-17.39956166
ENSG00000261513	RP11-432I5.8	1.00E-06	0.172959	-17.40007056
ENSG00000207771	MIR550A1	1.00E-06	0.172963	-17.40010393
ENSG00000278637	HIST1H4A	1.00E-06	0.173463	-17.40426844
ENSG00000277157	HIST1H4D	1.00E-06	0.173463	-17.40426844
ENSG00000272043	RP11-44N11.3	1.00E-06	0.173463	-17.40426844
ENSG00000240254	B4GALT4-AS1	1.00E-06	0.173698	-17.40622162
ENSG00000281918	Metazoa_SRP	1.00E-06	0.174021	-17.40890189
ENSG00000277051	Metazoa_SRP	1.00E-06	0.174021	-17.40890189
ENSG00000226441	PLCL2-AS1	1.00E-06	0.174021	-17.40890189
ENSG00000224658	RP11-631F7.1	1.00E-06	0.174021	-17.40890189
ENSG00000198821	CD247	1.00E-06	0.174409287	-17.41211733
ENSG00000250472	TRIM36-IT1	1.00E-06	0.174463	-17.41256158
ENSG00000205644	CTD-2375G15.1	1.00E-06	0.175425	-17.42049484
ENSG00000266019	MIR3609	1.00E-06	0.175892	-17.42433034
ENSG00000238090	AC006195.2	1.00E-06	0.175907	-17.42445337
ENSG00000233635	AC037445.1	1.00E-06	0.17605793	-17.42569068
ENSG00000212044	AL360091.1	1.00E-06	0.17648	-17.42914517
ENSG00000275278	RP11-946L16.2	1.00E-06	0.177086	-17.43409063
ENSG00000254571	RP11-60I3.4	1.00E-06	0.17718	-17.43485624
ENSG00000261049	RP11-357N13.1	1.00E-06	0.177192	-17.43495394
ENSG00000186118	TEX38	1.00E-06	0.177413809	-17.43675878
ENSG00000243051	RN7SL269P	1.00E-06	0.177444	-17.43700427
ENSG00000241939	RN7SL517P	1.00E-06	0.177444	-17.43700427
ENSG00000225907	RPS27P16	1.00E-06	0.177709	-17.43915722
ENSG00000225024	AC015977.5	1.00E-06	0.177931	-17.44095836
ENSG00000225903	RP1-144F13.3	1.00E-06	0.178028	-17.44174464
ENSG00000199629	RNU1-14P	1.00E-06	0.178028	-17.44174464
ENSG00000271081	RP11-465K4.4	1.00E-06	0.178028	-17.44174464
ENSG00000229282	RP1-40E16.2	1.00E-06	0.178252	-17.44355874
ENSG00000233998	SETP5	1.00E-06	0.179117	-17.45054274
ENSG00000241229	RN7SL443P	1.00E-06	0.179157	-17.45086489
ENSG00000232101	AC108059.2	1.00E-06	0.179207	-17.45126747
ENSG00000253019	RN7SKP35	1.00E-06	0.179207	-17.45126747
ENSG00000226291	AC091729.8	1.00E-06	0.179207	-17.45126747
ENSG00000264313	RN7SL644P	1.00E-06	0.179705	-17.45527102
ENSG00000274079	Metazoa_SRP	1.00E-06	0.179765	-17.45575263
ENSG00000277965	Metazoa_SRP	1.00E-06	0.179765	-17.45575263
ENSG00000259547	CYCSP2	1.00E-06	0.179803	-17.45605757
ENSG00000240905	RN7SL798P	1.00E-06	0.179803	-17.45605757
ENSG00000254432	RP11-33I11.2	1.00E-06	0.179803	-17.45605757
ENSG00000224477	RP1-81D8.3	1.00E-06	0.179849725	-17.45643243

ENSG00000241246	RN7SL302P	1.00E-06	0.180402	-17.46085581
ENSG00000243704	RN7SL105P	1.00E-06	0.181005	-17.46567002
ENSG00000275337	RN7SL680P	1.00E-06	0.181005	-17.46567002
ENSG00000248802	RP11-184M15.2	1.00E-06	0.1813087	-17.46808863
ENSG00000255231	MRPS36P4	1.00E-06	0.181612	-17.47050001
ENSG00000243420	RN7SL734P	1.00E-06	0.181612	-17.47050001
ENSG00000279366	RP11-380M21.3	1.00E-06	0.181612	-17.47050001
ENSG00000235318	MTND4LP13	1.00E-06	0.181612	-17.47050001
ENSG00000222678	RN7SKP213	1.00E-06	0.181612	-17.47050001
ENSG00000243373	RN7SL173P	1.00E-06	0.181637	-17.47069859
ENSG00000225043	RPL18AP2	1.00E-06	0.181899	-17.47277809
ENSG00000241809	CTD-2207L17.1	1.00E-06	0.181981	-17.47342831
ENSG00000231705	RP11-432J24.2	1.00E-06	0.182224	-17.47535346
ENSG00000274507	RP5-965G21.5	1.00E-06	0.182224	-17.47535346
ENSG00000243227	RN7SL55P	1.00E-06	0.182224	-17.47535346
ENSG00000239279	RN7SL184P	1.00E-06	0.182224	-17.47535346
ENSG00000247970	RP11-543C4.1	1.00E-06	0.182312	-17.47605
ENSG00000274886	SEPT14P17	1.00E-06	0.18261	-17.47840625
ENSG00000270640	RP11-373D23.2	1.00E-06	0.18284	-17.4802222
ENSG00000256502	RP11-955H22.2	1.00E-06	0.182891	-17.48062456
ENSG00000137976	DNASE2B	1.00E-06	0.18333525	-17.48412467
ENSG00000243437	RN7SL370P	1.00E-06	0.18346	-17.48510602
ENSG00000243562	RN7SL838P	1.00E-06	0.18346	-17.48510602
ENSG00000272030	RP1-178F15.4	1.00E-06	0.18346099	-17.4851138
ENSG00000279242	RP11-195O1.5	1.00E-06	0.183554	-17.48584503
ENSG00000271573	RP11-96C23.12	1.00E-06	0.184083	-17.48999687
ENSG00000240791	MTND4LP7	1.00E-06	0.184083	-17.48999687
ENSG00000235089	RP3-445O10.1	1.00E-06	0.184611	-17.49412899
ENSG00000201967	RN7SKP22	1.00E-06	0.184711	-17.49491026
ENSG00000259125	LRP1-AS	1.00E-06	0.18471211	-17.49491893
ENSG00000277391	MIR6881	1.00E-06	0.18515	-17.49833502
ENSG00000242894	RN7SL634P	1.00E-06	0.185344	-17.49984589
ENSG00000217159	LARP1P1	1.00E-06	0.185344	-17.49984589
ENSG00000240235	RN7SL794P	1.00E-06	0.185557	-17.5015029
ENSG00000239701	RP3-461F17.1	1.00E-06	0.185981	-17.50479572
ENSG00000241625	RN7SL18P	1.00E-06	0.185981	-17.50479572
ENSG00000244307	RN7SL395P	1.00E-06	0.185981	-17.50479572
ENSG00000261270	RP11-325K4.3	1.00E-06	0.186077	-17.50554022
ENSG00000275213	Metazoa_SRP	1.00E-06	0.186623	-17.50976727
ENSG00000263882	RN7SL774P	1.00E-06	0.186623	-17.50976727
ENSG00000160951	PTGER1	1.00E-06	0.186623	-17.50976727
ENSG00000253929	CASC21	1.00E-06	0.186623	-17.50976727
ENSG00000240205	RN7SL865P	1.00E-06	0.187268	-17.51474487
ENSG00000224087	AC018804.3	1.00E-06	0.187268	-17.51474487
ENSG00000223012	RN7SKP264	1.00E-06	0.187268	-17.51474487
ENSG00000263921	AC013737.1	1.00E-06	0.18734	-17.51529944
ENSG00000265501	AC005549.2	1.00E-06	0.18734	-17.51529944
ENSG00000266399	AC027307.2	1.00E-06	0.18734	-17.51529944

ENSG00000273384	RP5-1098D14.1	1.00E-06	0.187593	-17.51724647
ENSG00000162771	FAM71A	1.00E-06	0.187837	-17.51912175
ENSG00000235424	SUMO2P10	1.00E-06	0.187918	-17.51974374
ENSG00000266176	RP11-855A2.5	1.00E-06	0.188245	-17.52225202
ENSG00000257101	LRRC37A13P	1.00E-06	0.188573	-17.5247636
ENSG00000212542	RNA5SP496	1.00E-06	0.188677	-17.52555904
ENSG00000264271	RN7SL488P	1.00E-06	0.189232	-17.52979655
ENSG00000255115	RP11-91P24.3	1.00E-06	0.189232	-17.52979655
ENSG00000254522	RP11-113K21.1	1.00E-06	0.189232	-17.52979655
ENSG00000270387	RP11-315D13.1	1.00E-06	0.189232	-17.52979655
ENSG00000254946	RP11-531H8.1	1.00E-06	0.189564	-17.53232548
ENSG00000243173	RN7SL861P	1.00E-06	0.189897	-17.53485759
ENSG00000239253	RPS4XP23	1.00E-06	0.190298	-17.53790087
ENSG00000227875	RPL23AP89	1.00E-06	0.191014	-17.54331886
ENSG00000253024	RNU6-1238P	1.00E-06	0.191014	-17.54331886
ENSG00000237349	RP5-1065J22.2	1.00E-06	0.191238	-17.5450097
ENSG00000270813	NANOGNBP3	1.00E-06	0.191577	-17.54756484
ENSG00000274216	Metazoa_SRP	1.00E-06	0.191917	-17.55012299
ENSG00000279433	RP11-4B16.1	1.00E-06	0.192087	-17.55140036
ENSG00000207042	RNU6-196P	1.00E-06	0.192204	-17.55227884
ENSG00000186973	FAM183A	1.00E-06	0.192599988	-17.55524809
ENSG00000275642	Metazoa_SRP	1.00E-06	0.1926	-17.55524818
ENSG00000269889	RP11-816J6.3	1.00E-06	0.1926	-17.55524818
ENSG00000274023	RP11-732M18.4	1.00E-06	0.192943	-17.55781518
ENSG00000223692	DIP2A-IT1	1.00E-06	0.192953	-17.55788995
ENSG00000275014	RN7SL166P	1.00E-06	0.193287	-17.56038508
ENSG00000276675	TTC28-AS1_4	1.00E-06	0.193287	-17.56038508
ENSG00000220643	RP3-391O22.1	1.00E-06	0.193287	-17.56038508
ENSG00000220871	DNAJC19P6	1.00E-06	0.193287	-17.56038508
ENSG00000263475	AL353805.1	1.00E-06	0.193287	-17.56038508
ENSG00000259598	RP11-275I4.1	1.00E-06	0.193465	-17.56171306
ENSG00000172247	C1QTNF4	1.00E-06	0.193846	-17.56455144
ENSG00000279864	RP11-570J4.1	1.00E-06	0.19398	-17.56554839
ENSG00000229626	PIGCP2	1.00E-06	0.194445	-17.56900261
ENSG00000260487	RP11-297C4.3	1.00E-06	0.194752	-17.57127862
ENSG00000257570	RP11-133N21.12	1.00E-06	0.194834	-17.57188593
ENSG00000231384	AC007919.19	1.00E-06	0.194876	-17.5721969
ENSG00000275291	U1	1.00E-06	0.194878	-17.57221171
ENSG00000201699	RNU1-59P	1.00E-06	0.194878	-17.57221171
ENSG00000225655	PGM5-AS1	1.00E-06	0.195174	-17.57440135
ENSG00000256512	RP11-860B13.3	1.00E-06	0.195194	-17.57454918
ENSG00000226899	RP11-298J20.3	1.00E-06	0.195616	-17.57766485
ENSG00000266385	RP11-227G15.8	1.00E-06	0.196088	-17.58114172
ENSG00000231193	RP11-462B18.2	1.00E-06	0.196445	-17.58376592
ENSG00000254246	CTB-120L21.1	1.00E-06	0.196802	-17.58638536
ENSG00000224733	RP1-86D1.5	1.00E-06	0.19716	-17.58900736
ENSG00000199460	RNU6-1216P	1.00E-06	0.197262	-17.58975354
ENSG00000274428	U1	1.00E-06	0.197315	-17.59014111

ENSG00000251934	RNU6-1143P	1.00E-06	0.197748	-17.59330358
ENSG00000260498	RP4-536B24.4	1.00E-06	0.198066367	-17.59562439
ENSG00000274848	Metazoa_SRP	1.00E-06	0.198244	-17.59691768
ENSG00000140459	CYP11A1	1.00E-06	0.19895305	-17.60206849
ENSG00000242430	RN7SL694P	1.00E-06	0.198973	-17.60221315
ENSG00000243423	RP5-837J1.1	1.00E-06	0.199707	-17.60752538
ENSG00000279397	RP11-196B3.4	1.00E-06	0.199829	-17.60840644
ENSG00000230526	RP11-472G21.2	1.00E-06	0.199988	-17.60955391
ENSG00000258800	CTD-2302E22.2	1.00E-06	0.199989	-17.60956112
ENSG00000228577	AC010731.2	1.00E-06	0.200316	-17.61191813
ENSG00000248984	AC004054.1	1.00E-06	0.200446	-17.6128541
ENSG00000268564	AC003956.1	1.00E-06	0.201064	-17.61729527
ENSG00000266667	RP11-849N15.4	1.00E-06	0.201192	-17.61821341
ENSG00000243005	RN7SL16P	1.00E-06	0.201192	-17.61821341
ENSG00000197334	Metazoa_SRP	1.00E-06	0.201192	-17.61821341
ENSG00000221552	MIR1303	1.00E-06	0.201379	-17.61955372
ENSG00000222208	RNA5SP129	1.00E-06	0.201806	-17.62260954
ENSG00000270531	RP11-169K17.2	1.00E-06	0.201942	-17.62358147
ENSG00000257137	C12orf80	1.00E-06	0.202200259	-17.62542532
ENSG00000252225	Y_RNA	1.00E-06	0.202208	-17.62548055
ENSG00000201725	RNU6-304P	1.00E-06	0.20232	-17.62627942
ENSG00000280914	Metazoa_SRP	1.00E-06	0.202699	-17.62897945
ENSG00000200304	RNU6-1255P	1.00E-06	0.202952	-17.63077903
ENSG00000273336	OR7M1P	1.00E-06	0.203079	-17.63168154
ENSG00000276867	CTD-2358C21.5	1.00E-06	0.203079	-17.63168154
ENSG00000204904	LINC01545	1.00E-06	0.203461273	-17.63439469
ENSG00000279799	AC006077.3	1.00E-06	0.203692	-17.63602979
ENSG00000244043	RPS27P27	1.00E-06	0.203748	-17.63642637
ENSG00000227692	MED28P3	1.00E-06	0.203844	-17.63710597
ENSG00000280981	Metazoa_SRP	1.00E-06	0.203993	-17.63816012
ENSG00000244158	RP1-93H18.6	1.00E-06	0.204228	-17.63982115
ENSG00000280743	Metazoa_SRP	1.00E-06	0.204228	-17.63982115
ENSG00000226772	CTA-747E2.10	1.00E-06	0.204333	-17.64056269
ENSG00000206698	RNU1-73P	1.00E-06	0.204602	-17.64246072
ENSG00000236889	RP11-38J22.1	1.00E-06	0.204887	-17.64446892
ENSG00000278700	Metazoa_SRP	1.00E-06	0.204975	-17.64508844
ENSG00000253579	SUMO2P16	1.00E-06	0.205002	-17.64527846
ENSG00000223923	AC010136.2	1.00E-06	0.205175	-17.64649543
ENSG00000231961	RP5-967N21.7	1.00E-06	0.205397	-17.64805558
ENSG00000258531	BANF1P1	1.00E-06	0.205781	-17.65075026
ENSG00000266308	RN7SL510P	1.00E-06	0.205781	-17.65075026
ENSG00000226645	AP006216.10	1.00E-06	0.205917	-17.65170341
ENSG00000235262	KDM5C-IT1	1.00E-06	0.206446	-17.65540494
ENSG00000215841	RP11-800A3.2	1.00E-06	0.206567	-17.65625027
ENSG00000277385	Metazoa_SRP	1.00E-06	0.206567	-17.65625027
ENSG00000200785	SNORD8	1.00E-06	0.206642	-17.65677399
ENSG00000282625	MRPL57P8	1.00E-06	0.207358	-17.66176418
ENSG00000279786	CTD-2014D20.1	1.00E-06	0.208614	-17.67047645

ENSG00000183308	AC005037.3	1.00E-06	0.20875259	-17.67143457
ENSG00000268407	AC006115.6	1.00E-06	0.20898	-17.67300535
ENSG00000259483	RP11-930O11.2	1.00E-06	0.209364	-17.67565387
ENSG00000237158	RP11-472F14.4	1.00E-06	0.209364	-17.67565387
ENSG00000255372	CTD-3064C13.1	1.00E-06	0.209769	-17.67844196
ENSG00000222511	Y_RNA	1.00E-06	0.210177	-17.68124528
ENSG00000278486	YRDCP1	1.00E-06	0.210422	-17.68292602
ENSG00000235224	RP11-236P24.1	1.00E-06	0.210586	-17.68405
ENSG00000178462	TUBAL3	1.00E-06	0.210703	-17.68485133
ENSG00000262223	RP11-1055B8.3	1.00E-06	0.211087483	-17.68748151
ENSG00000254983	RP11-573E11.2	1.00E-06	0.211408	-17.68967045
ENSG00000257849	RP11-547C5.1	1.00E-06	0.211408	-17.68967045
ENSG00000277510	Metazoa_SRP	1.00E-06	0.211408	-17.68967045
ENSG00000279679	RP11-278J6.5	1.00E-06	0.211408	-17.68967045
ENSG00000216718	RP3-522P13.1	1.00E-06	0.21242	-17.69656008
ENSG00000201432	Y_RNA	1.00E-06	0.212617	-17.69789743
ENSG00000240983	RP11-742D12.1	1.00E-06	0.213004	-17.700521
ENSG00000277721	Metazoa_SRP	1.00E-06	0.213073	-17.70098826
ENSG00000259654	RP11-245C17.2	1.00E-06	0.21337	-17.70299782
ENSG00000273549	Metazoa_SRP	1.00E-06	0.213509	-17.70393736
ENSG00000234614	AL450992.2	1.00E-06	0.213915	-17.70667812
ENSG00000250831	CTD-2232E5.2	1.00E-06	0.213915	-17.70667812
ENSG00000232874	RP11-135A1.2	1.00E-06	0.214014	-17.70734565
ENSG00000259242	AC002306.1	1.00E-06	0.214416	-17.71005304
ENSG00000277958	Metazoa_SRP	1.00E-06	0.214947	-17.71362145
ENSG00000236132	CTA-440B3.1	1.00E-06	0.215574	-17.71782366
ENSG00000274011	Metazoa_SRP	1.00E-06	0.215619	-17.71812479
ENSG00000253399	AC078852.2	1.00E-06	0.215991	-17.72061167
ENSG00000275485	RP11-261P13.6	1.00E-06	0.216482	-17.72388755
ENSG00000237373	BRWD1-IT1	1.00E-06	0.216925	-17.7268368
ENSG00000250103	RP11-380D23.2	1.00E-06	0.217262754	-17.72908134
ENSG00000260182	RP11-616M22.5	1.00E-06	0.217351	-17.72966721
ENSG00000237501	RP11-816B4.1	1.00E-06	0.217351	-17.72966721
ENSG00000232598	RP1-90K10.4	1.00E-06	0.217789	-17.73257156
ENSG00000228076	RP11-475E11.2	1.00E-06	0.218228	-17.73547669
ENSG00000240098	RN7SL351P	1.00E-06	0.218228	-17.73547669
ENSG00000267131	RP11-332H18.5	1.00E-06	0.21825	-17.73562213
ENSG00000267703	CTD-2013N17.4	1.00E-06	0.218642	-17.73821104
ENSG00000252824	SNORA48	1.00E-06	0.218914	-17.7400047
ENSG00000281517	Metazoa_SRP	1.00E-06	0.219111	-17.74130239
ENSG00000231559	RP3-354J5.3	1.00E-06	0.219111	-17.74130239
ENSG00000250971	RP11-696F12.1	1.00E-06	0.219479	-17.74372338
ENSG00000188820	FAM26F	1.00E-06	0.219987386	-17.74706128
ENSG00000237921	AC004543.2	1.00E-06	0.220002	-17.74715711
ENSG00000201492	RNA5SP78	1.00E-06	0.220152	-17.74814043
ENSG00000254315	RP11-267M23.3	1.00E-06	0.22045	-17.75009195
ENSG00000250948	RP11-1079K10.2	1.00E-06	0.220901	-17.75304042
ENSG00000233713	RP11-379P1.4	1.00E-06	0.221188	-17.75491359

ENSG00000264102	MIR4688	1.00E-06	0.221355	-17.75600244
ENSG00000263159	RP11-462G12.4	1.00E-06	0.221805	-17.75893236
ENSG00000278388	GS1-345D13.1	1.00E-06	0.221805	-17.75893236
ENSG00000224764	RP11-54O15.3	1.00E-06	0.222261	-17.7618953
ENSG00000265212	AC007009.2	1.00E-06	0.222281	-17.76202511
ENSG00000212027	MIR374B	1.00E-06	0.222281	-17.76202511
ENSG00000179363	TMEM31	1.00E-06	0.223024	-17.76683944
ENSG00000277198	RN7SL270P	1.00E-06	0.223416	-17.76937298
ENSG00000280431	RP11-218I21.2	1.00E-06	0.224256	-17.77478706
ENSG00000266624	MIR1234	1.00E-06	0.224379	-17.77557813
ENSG00000280823	Metazoa_SRP	1.00E-06	0.224567	-17.77678641
ENSG00000266975	FARSA-AS1	1.00E-06	0.224567	-17.77678641
ENSG00000234055	RP11-247A12.1	1.00E-06	0.224567	-17.77678641
ENSG00000256633	RP11-169D4.2	1.00E-06	0.224925	-17.7790845
ENSG00000249752	RP11-563M4.1	1.00E-06	0.225033	-17.77977706
ENSG00000273035	RP11-449G16.1	1.00E-06	0.226921	-17.7918306
ENSG00000202260	RN7SKP69	1.00E-06	0.227129	-17.7931524
ENSG00000270549	RP11-293K19.1	1.00E-06	0.227397	-17.7948537
ENSG00000276680	HYMAI	1.00E-06	0.227397	-17.7948537
ENSG00000277588	MIR6806	1.00E-06	0.228322	-17.80071035
ENSG00000272239	CTB-108O6.2	1.00E-06	0.228356	-17.80092517
ENSG00000244159	RP11-1070A24.1	1.00E-06	0.228356	-17.80092517
ENSG00000231804	RP11-317B17.3	1.00E-06	0.228356	-17.80092517
ENSG00000263913	AC004812.1	1.00E-06	0.228783	-17.80362033
ENSG00000258210	RP11-478C19.2	1.00E-06	0.228839	-17.80397342
ENSG00000278370	RP11-387D10.4	1.00E-06	0.228839	-17.80397342
ENSG00000236160	GS1-541M1.2	1.00E-06	0.229452	-17.80783286
ENSG00000271236	SUMO2P15	1.00E-06	0.22972	-17.80951694
ENSG00000259112	NDUFC2-KCTD14	1.00E-06	0.229777949	-17.80988083
ENSG00000221066	SNORD111	1.00E-06	0.2303	-17.81315489
ENSG00000237410	AP001092.4	1.00E-06	0.230792	-17.81623369
ENSG00000250266	LINC01612	1.00E-06	0.230792	-17.81623369
ENSG00000223203	RNA5SP221	1.00E-06	0.231194	-17.81874443
ENSG00000223018	AL096700.1	1.00E-06	0.231412	-17.82010415
ENSG00000254267	KB-1458E12.1	1.00E-06	0.232357	-17.82598358
ENSG00000240328	RP11-841C19.1	1.00E-06	0.232623	-17.82763422
ENSG00000283057	RP11-417N10.5	1.00E-06	0.233278	-17.83169073
ENSG00000248979	LAMTOR3P2	1.00E-06	0.233278	-17.83169073
ENSG00000221717	AL583860.1	1.00E-06	0.233461	-17.83282204
ENSG00000252490	RN7SKP66	1.00E-06	0.234288	-17.83792354
ENSG00000271984	RP3-337O18.9	1.00E-06	0.235264	-17.84392105
ENSG00000278108	MIR6757	1.00E-06	0.235306	-17.84417858
ENSG00000264370	MIR3125	1.00E-06	0.23591	-17.84787705
ENSG00000264864	MIR3613	1.00E-06	0.236389	-17.85080338
ENSG00000259544	RP11-158M2.2	1.00E-06	0.236709	-17.85275503
ENSG00000261053	NAMPTP3	1.00E-06	0.236777	-17.85316942
ENSG00000273210	AP001437.1	1.00E-06	0.237371	-17.85678416
ENSG00000221069	AC000029.1	1.00E-06	0.237892	-17.85994723

ENSG00000276951	RP11-11A9.1	1.00E-06	0.238	-17.86060205
ENSG00000257653	RP11-579D7.2	1.00E-06	0.238298	-17.86240732
ENSG00000216548	RP11-471B18.1	1.00E-06	0.238416	-17.86312153
ENSG00000278206	RP1-20N2.8	1.00E-06	0.238682	-17.86473025
ENSG00000223341	RN7SKP3	1.00E-06	0.238856	-17.86578159
ENSG00000263782	AC117481.1	1.00E-06	0.238974	-17.86649414
ENSG00000238140	RP11-253A20.1	1.00E-06	0.239471	-17.86949143
ENSG00000200135	Y_RNA	1.00E-06	0.239471	-17.86949143
ENSG00000201098	RNY1	1.00E-06	0.239471	-17.86949143
ENSG00000234460	XXyac-YM21GA2.4	1.00E-06	0.239471	-17.86949143
ENSG00000279013	RP11-254A24.2	1.00E-06	0.240297	-17.87445911
ENSG00000252473	SNORA67	1.00E-06	0.240535	-17.87588731
ENSG00000201365	Y_RNA	1.00E-06	0.240535	-17.87588731
ENSG00000229046	HMGN1P2	1.00E-06	0.240924	-17.87821859
ENSG00000267638	RP11-546M21.6	1.00E-06	0.241205	-17.87990029
ENSG00000267637	RP11-619I22.1	1.00E-06	0.242693	-17.88877297
ENSG00000244391	RN7SL330P	1.00E-06	0.243278	-17.89224634
ENSG00000221553	AC004924.1	1.00E-06	0.243361	-17.89273846
ENSG00000249593	CTB-46B19.2	1.00E-06	0.244434566	-17.89908879
ENSG00000254131	RP11-1007J8.1	1.00E-06	0.24483	-17.90142082
ENSG00000273342	KB-1440D3.14	1.00E-06	0.245444	-17.90503437
ENSG00000258412	RP11-404P21.5	1.00E-06	0.245688	-17.90646787
ENSG00000254501	AP003068.9	1.00E-06	0.245839	-17.90735428
ENSG00000248399	RP11-503N18.4	1.00E-06	0.246003	-17.90831638
ENSG00000227895	ISX-AS1	1.00E-06	0.24649	-17.91116959
ENSG00000263985	AC116407.1	1.00E-06	0.246689	-17.91233386
ENSG00000266429	AL442639.1	1.00E-06	0.246689	-17.91233386
ENSG00000258914	CTD-2134A5.3	1.00E-06	0.247164	-17.9151091
ENSG00000267581	CTC-559E9.4	1.00E-06	0.24756	-17.9174187
ENSG00000221344	AC068946.1	1.00E-06	0.247781	-17.91870604
ENSG00000273982	RP11-332H18.7	1.00E-06	0.247881	-17.91928817
ENSG00000221962	TMEM14EP	1.00E-06	0.249127	-17.92652186
ENSG00000257058	RP11-864I4.4	1.00E-06	0.249148	-17.92664347
ENSG00000276563	RP11-568K15.2	1.00E-06	0.249404	-17.92812508
ENSG00000251935	RN7SKP179	1.00E-06	0.249404	-17.92812508
ENSG00000253413	RP11-960H2.2	1.00E-06	0.249404	-17.92812508
ENSG00000266990	LLNLR-284B4.2	1.00E-06	0.249917	-17.93108952
ENSG00000277299	RP11-837J7.4	1.00E-06	0.250162	-17.93250313
ENSG00000274314	MIR6749	1.00E-06	0.250994	-17.93729335
ENSG00000206755	SNORA30	1.00E-06	0.251723	-17.94147752
ENSG00000272440	RP11-379F4.6	1.00E-06	0.251723	-17.94147752
ENSG00000242041	RP11-70F11.2	1.00E-06	0.251799	-17.94191303
ENSG00000239742	RN7SL672P	1.00E-06	0.252676	-17.94692911
ENSG00000185467	KPNA7	1.00E-06	0.252808	-17.94768259
ENSG00000278518	RP11-108K14.12	1.00E-06	0.254431	-17.95691493
ENSG00000199535	RNA5SP305	1.00E-06	0.254685	-17.95835447
ENSG00000264966	MIR5094	1.00E-06	0.254685	-17.95835447
ENSG00000261092	RP11-21B23.2	1.00E-06	0.254685	-17.95835447

ENSG00000258687	RP11-218E20.2	1.00E-06	0.255051	-17.96042623
ENSG00000280904	SNORD19B	1.00E-06	0.255579	-17.96340977
ENSG00000219881	GAPDHP42	1.00E-06	0.255983	-17.96568848
ENSG00000207326	Y_RNA	1.00E-06	0.25636	-17.96781165
ENSG00000201749	Y_RNA	1.00E-06	0.25636	-17.96781165
ENSG00000212342	SNORA12	1.00E-06	0.256725	-17.96986427
ENSG00000271370	RP11-253I19.4	1.00E-06	0.257037	-17.97161652
ENSG00000239482	RP11-90K6.1	1.00E-06	0.257308217	-17.973138
ENSG00000276003	FAM13A-AS1_2	1.00E-06	0.258075	-17.97743087
ENSG00000235579	AC007283.4	1.00E-06	0.25824	-17.97835296
ENSG00000273530	AC016821.2	1.00E-06	0.258486	-17.97972662
ENSG00000269480	CTD-3032J10.3	1.00E-06	0.261387	-17.99582787
ENSG00000271283	CTC-412M14.6	1.00E-06	0.261452	-17.99618658
ENSG00000249234	RP11-452J21.2	1.00E-06	0.261452	-17.99618658
ENSG00000227413	RP5-1042K10.12	1.00E-06	0.262218	-18.0004072
ENSG00000281681	Metazoa_SRP	1.00E-06	0.262402	-18.00141919
ENSG00000278400	RP11-474N8.9	1.00E-06	0.262721	-18.003172
ENSG00000216331	HIST1H1PS1	1.00E-06	0.262721	-18.003172
ENSG00000267625	RP11-1094M14.14	1.00E-06	0.263289	-18.00628772
ENSG00000188038	NRN1L	1.00E-06	0.263958857	-18.00995355
ENSG00000221217	AL359851.1	1.00E-06	0.264589	-18.01339356
ENSG00000253630	CTC-370J7.1	1.00E-06	0.265297	-18.01724884
ENSG00000221211	AC078794.1	1.00E-06	0.266308	-18.02273624
ENSG00000275743	TRBV14	1.00E-06	0.266603	-18.02433349
ENSG00000260436	RP11-20I23.7	1.00E-06	0.266654	-18.02460944
ENSG00000258955	LINC00519	1.00E-06	0.267719	-18.03036001
ENSG00000251128	WWC2-AS1	1.00E-06	0.267924	-18.03146429
ENSG00000235450	RP11-142A5.1	1.00E-06	0.267924	-18.03146429
ENSG00000219298	RP11-472G23.3	1.00E-06	0.268064	-18.03221796
ENSG00000232936	RP11-80H5.2	1.00E-06	0.268365797	-18.03384129
ENSG00000260033	CTC-527H23.1	1.00E-06	0.269256	-18.03861897
ENSG00000272710	CTD-2026G6.3	1.00E-06	0.269256	-18.03861897
ENSG00000252507	RNU7-81P	1.00E-06	0.270603	-18.04581831
ENSG00000221469	AL133260.1	1.00E-06	0.270603	-18.04581831
ENSG00000265024	AC073089.1	1.00E-06	0.270603	-18.04581831
ENSG00000266772	AC211469.1	1.00E-06	0.270603	-18.04581831
ENSG00000213540	RP11-486G15.1	1.00E-06	0.271151	-18.04873697
ENSG00000236003	AC007050.17	1.00E-06	0.271502	-18.0506033
ENSG00000270839	CTC-571O20.1	1.00E-06	0.271508	-18.05063518
ENSG00000269050	CTC-360G5.6	1.00E-06	0.271561	-18.05091678
ENSG00000237604	AP001056.1	1.00E-06	0.271623	-18.05124612
ENSG00000228525	RP5-1087E8.2	1.00E-06	0.271962	-18.05304556
ENSG00000216813	RP11-812I20.2	1.00E-06	0.272647	-18.05667476
ENSG00000269125	RP11-98F14.11	1.00E-06	0.273576	-18.06158215
ENSG00000221555	AC068669.1	1.00E-06	0.273713	-18.06230443
ENSG00000214626	POLR3DP1	1.00E-06	0.274284	-18.06531094
ENSG00000141040	ZNF287	1.00E-06	0.274395968	-18.06589976
ENSG00000202415	RN7SKP269	1.00E-06	0.274844	-18.06825346

ENSG00000248221	STX18-IT1	1.00E-06	0.275596	-18.07219542
ENSG00000166220	TBATA	1.00E-06	0.275742882	-18.07296412
ENSG00000262959	RPL23AP86	1.00E-06	0.276105	-18.07485749
ENSG00000278668	RP11-227G15.11	1.00E-06	0.276125	-18.07496199
ENSG00000267124	CTD-3113P16.5	1.00E-06	0.276236	-18.07554182
ENSG00000279853	RP5-844F9.1	1.00E-06	0.276509	-18.07696691
ENSG00000259340	CTD-2027G2.1	1.00E-06	0.276528	-18.07706604
ENSG00000229915	AC016999.2	1.00E-06	0.276941	-18.07921913
ENSG00000207175	RNU1-67P	1.00E-06	0.277203	-18.08058335
ENSG00000272914	RP11-330O11.3	1.00E-06	0.277541	-18.08234139
ENSG00000277077	RP11-101P17.15	1.00E-06	0.277541	-18.08234139
ENSG00000221265	MIR1255A	1.00E-06	0.277787	-18.08361956
ENSG00000270140	RP5-1021I20.6	1.00E-06	0.277917	-18.08429456
ENSG00000273813	RP11-11A9.2	1.00E-06	0.279209	-18.09098592
ENSG00000270914	RP5-1077B9.5	1.00E-06	0.279332	-18.09162133
ENSG00000252637	RNA5SP268	1.00E-06	0.279332	-18.09162133
ENSG00000227583	RP11-119F7.3	1.00E-06	0.280409	-18.09717313
ENSG00000273274	ZBTB8B	1.00E-06	0.281356	-18.1020372
ENSG00000222276	RNU2-33P	1.00E-06	0.281878	-18.10471136
ENSG00000238898	RNU1-80P	1.00E-06	0.282369	-18.10722218
ENSG00000272715	RP4-753F5.1	1.00E-06	0.282614	-18.10847341
ENSG00000234612	H2AFZP5	1.00E-06	0.283353	-18.11224095
ENSG00000223125	RNU2-32P	1.00E-06	0.283353	-18.11224095
ENSG00000225096	XXbac-BPG55C20.7	1.00E-06	0.284087	-18.11597329
ENSG00000260012	RP11-329J18.4	1.00E-06	0.284845	-18.11981756
ENSG00000228639	AC005152.3	1.00E-06	0.285268291	-18.12195987
ENSG00000260132	LA16c-312E8.2	1.00E-06	0.285596	-18.12361625
ENSG00000275005	RP11-234G16.5	1.00E-06	0.285848	-18.12488867
ENSG00000238705	MIR1976	1.00E-06	0.286214	-18.12673472
ENSG00000264497	RP11-640N20.5	1.00E-06	0.287074	-18.13106315
ENSG00000222679	RNU6-1267P	1.00E-06	0.287789	-18.13465192
ENSG00000276108	AL512506.1	1.00E-06	0.287875	-18.13508298
ENSG00000265129	AC064837.1	1.00E-06	0.287875	-18.13508298
ENSG00000280403	AL137860.1	1.00E-06	0.288643	-18.13892671
ENSG00000256155	RP11-277P12.9	1.00E-06	0.2889	-18.14021068
ENSG00000277794	Metazoa_SRP	1.00E-06	0.289436	-18.14288485
ENSG00000277216	AC022073.1	1.00E-06	0.289482	-18.14311412
ENSG00000263933	AL365502.1	1.00E-06	0.289482	-18.14311412
ENSG00000206582	Y_RNA	1.00E-06	0.29013	-18.14633996
ENSG00000279513	RP4-675C20.4	1.00E-06	0.290191	-18.14664325
ENSG00000248416	RP11-1191J2.4	1.00E-06	0.29247	-18.15792912
ENSG00000234523	NDUFB1P2	1.00E-06	0.295203	-18.17134786
ENSG00000258748	CTD-2223O18.1	1.00E-06	0.295203	-18.17134786
ENSG00000229263	AC004691.5	1.00E-06	0.296822	-18.1792385
ENSG00000252335	RNU6-62P	1.00E-06	0.297133	-18.18074932
ENSG00000278520	MIR7851	1.00E-06	0.297663	-18.18332038
ENSG00000267852	CTB-133G6.2	1.00E-06	0.298185	-18.18584816
ENSG00000229723	LINC01054	1.00E-06	0.298238	-18.18610456

ENSG00000233674	RP11-184I16.3	1.00E-06	0.29956	-18.19248547
ENSG00000260710	RP11-616M22.7	1.00E-06	0.299961	-18.19441541
ENSG00000225711	RP11-345I18.4	1.00E-06	0.300253	-18.19581914
ENSG00000258013	RPL3P13	1.00E-06	0.30067	-18.1978214
ENSG00000222465	RNU2-5P	1.00E-06	0.30067	-18.1978214
ENSG00000233541	RPL31P47	1.00E-06	0.300919	-18.19901568
ENSG00000229357	CYCSP51	1.00E-06	0.302939	-18.2086678
ENSG00000213548	AC005522.6	1.00E-06	0.303451	-18.21110405
ENSG00000274408	5S_rRNA	1.00E-06	0.303603	-18.21182652
ENSG00000238765	RNA5SP57	1.00E-06	0.303603	-18.21182652
ENSG00000206847	Y_RNA	1.00E-06	0.303835	-18.21292854
ENSG00000277817	MIR6738	1.00E-06	0.304428	-18.21574153
ENSG00000254550	OMP	1.00E-06	0.305803	-18.22224303
ENSG00000263768	AC060226.1	1.00E-06	0.306343	-18.22478836
ENSG00000234469	CLDN34	1.00E-06	0.306774	-18.22681669
ENSG00000206731	SNORA36	1.00E-06	0.306826	-18.22706121
ENSG00000250289	VWA8P1	1.00E-06	0.307503	-18.23024096
ENSG00000276242	AC117507.1	1.00E-06	0.307842	-18.23183055
ENSG00000273900	RP11-459O16.7	1.00E-06	0.308582	-18.23529438
ENSG00000258978	HIF1AP1	1.00E-06	0.30926	-18.23846072
ENSG00000264999	MIR5089	1.00E-06	0.30926	-18.23846072
ENSG00000230480	AC093142.2	1.00E-06	0.310146	-18.24258799
ENSG00000277575	RP11-292B8.2	1.00E-06	0.311083	-18.24694003
ENSG00000262870	CYCSP40	1.00E-06	0.311899	-18.2507194
ENSG00000212332	RNU6-780P	1.00E-06	0.312234	-18.25226812
ENSG00000201659	RNU12-2P	1.00E-06	0.312234	-18.25226812
ENSG00000200814	RNU6-595P	1.00E-06	0.313596	-18.25854763
ENSG00000199536	RNU6-315P	1.00E-06	0.313596	-18.25854763
ENSG00000252329	SCARNA16	1.00E-06	0.314655	-18.26341134
ENSG00000274822	MIR6762	1.00E-06	0.314655	-18.26341134
ENSG00000263774	AC017104.1	1.00E-06	0.314655	-18.26341134
ENSG00000270377	RP11-384L8.2	1.00E-06	0.314655	-18.26341134
ENSG00000273571	ST7-OT4_2	1.00E-06	0.314655	-18.26341134
ENSG00000263196	MTND3P13	1.00E-06	0.315571	-18.26760511
ENSG00000211491	MIR320D1	1.00E-06	0.315703	-18.26820844
ENSG00000238065	AC010740.1	1.00E-06	0.316495	-18.27182318
ENSG00000227436	FCF1P1	1.00E-06	0.316851	-18.27344504
ENSG00000279357	RP11-475D10.4	1.00E-06	0.318356	-18.28028143
ENSG00000238880	RNU7-59P	1.00E-06	0.3194	-18.28500479
ENSG00000206775	SNORD37	1.00E-06	0.319411	-18.28505447
ENSG00000256262	USP30-AS1	1.00E-06	0.319915	-18.28732911
ENSG00000249639	CTB-138E5.1	1.00E-06	0.319999	-18.28770787
ENSG00000271100	RP11-697H9.5	1.00E-06	0.32024	-18.288794
ENSG00000265461	AC068014.2	1.00E-06	0.320305	-18.28908679
ENSG00000280137	RP11-297C4.6	1.00E-06	0.320369	-18.28937503
ENSG00000228839	PIK3IP1-AS1	1.00E-06	0.321038547	-18.292387
ENSG00000233222	RP11-216N14.9	1.00E-06	0.321126	-18.29277995
ENSG00000237343	RP11-763B22.4	1.00E-06	0.321543	-18.29465216

ENSG00000238282	RP5-1164C1.2	1.00E-06	0.322146	-18.29735516
ENSG00000270031	RP3-426I6.6	1.00E-06	0.322612	-18.29944058
ENSG00000233776	LINC01251	1.00E-06	0.322942	-18.30091556
ENSG00000228132	RP11-279E1.1	1.00E-06	0.323107	-18.30165248
ENSG00000267418	TCEB1P29	1.00E-06	0.324075	-18.30596821
ENSG00000274934	SMAD5-AS1_4	1.00E-06	0.324075	-18.30596821
ENSG00000269752	CTD-3149D2.3	1.00E-06	0.324506	-18.30788563
ENSG00000231148	HMGB1P7	1.00E-06	0.325431	-18.31199216
ENSG00000238594	snoU13	1.00E-06	0.325774	-18.31351194
ENSG00000252612	Y_RNA	1.00E-06	0.325887	-18.31401228
ENSG00000255931	RP11-286N22.10	1.00E-06	0.326271	-18.31571124
ENSG00000200688	Y_RNA	1.00E-06	0.326766	-18.31789835
ENSG00000281726	GRIK4_3p_UTR	1.00E-06	0.326978	-18.31883404
ENSG00000225185	RP11-481K9.4	1.00E-06	0.327804	-18.32247393
ENSG00000207205	RNVU1-15	1.00E-06	0.328003	-18.32334948
ENSG00000267647	MAP1LC3P	1.00E-06	0.328003	-18.32334948
ENSG00000222267	RNU6-892P	1.00E-06	0.328003	-18.32334948
ENSG00000267332	BOLA3P2	1.00E-06	0.32841	-18.32513853
ENSG00000264171	MIR4305	1.00E-06	0.328968	-18.32758773
ENSG00000244383	FAM3D-AS1	1.00E-06	0.329602	-18.33036547
ENSG00000225794	AC073321.4	1.00E-06	0.331012	-18.33652399
ENSG00000252568	RNU7-28P	1.00E-06	0.331707	-18.33954993
ENSG00000280567	Metazoa_SRP	1.00E-06	0.332708	-18.34389703
ENSG00000240311	LA16c-306E5.1	1.00E-06	0.33349	-18.34728398
ENSG00000256288	RP11-277P12.10	1.00E-06	0.333562	-18.34759542
ENSG00000263709	RP11-321A17.4	1.00E-06	0.333732	-18.3483305
ENSG00000207393	RNU6-136P	1.00E-06	0.333827	-18.34874112
ENSG00000199066	MIR330	1.00E-06	0.333936	-18.34921211
ENSG00000243426	RN7SL454P	1.00E-06	0.334077	-18.34982114
ENSG00000270986	RP11-123C21.2	1.00E-06	0.334224	-18.35045581
ENSG00000257256	RP3-405J10.4	1.00E-06	0.334301	-18.35078814
ENSG00000280376	RP11-93O14.3	1.00E-06	0.334766	-18.35279348
ENSG00000264748	AC025470.1	1.00E-06	0.335032	-18.35393937
ENSG00000199426	RNU1-108P	1.00E-06	0.336152	-18.35875421
ENSG00000256159	RP11-820K3.4	1.00E-06	0.336849	-18.36174249
ENSG00000220557	HMGB1P13	1.00E-06	0.337199	-18.36324073
ENSG00000265507	MIR4435-1	1.00E-06	0.338253	-18.3677432
ENSG00000266139	MIR4435-2	1.00E-06	0.338253	-18.3677432
ENSG00000252892	RNU6-548P	1.00E-06	0.338841	-18.37024893
ENSG00000281288	AC008906.1	1.00E-06	0.340618	-18.37779515
ENSG00000279363	AL162426.1	1.00E-06	0.340759	-18.37839224
ENSG00000245311	ARNTL2-AS1	1.00E-06	0.341422	-18.3811965
ENSG00000217442	SYCE3	1.00E-06	0.34181352	-18.38284994
ENSG00000275849	AL353602.1	1.00E-06	0.342942	-18.38760508
ENSG00000212525	RNA5SP212	1.00E-06	0.343944	-18.39181416
ENSG00000184502	GAST	1.00E-06	0.34545	-18.39811739
ENSG00000274115	MIR6081	1.00E-06	0.345925	-18.40009976
ENSG00000253585	KB-1184D12.1	1.00E-06	0.346173	-18.40113368

ENSG00000200130	SNORA63	1.00E-06	0.346927	-18.4042726
ENSG00000281075	Metazoa_SRP	1.00E-06	0.34855	-18.4110061
ENSG00000231485	RP4-535B20.1	1.00E-06	0.349165	-18.41354943
ENSG00000237418	AC069257.6	1.00E-06	0.349165	-18.41354943
ENSG00000199290	Y_RNA	1.00E-06	0.350191	-18.41778248
ENSG00000225062	CATIP-AS1	1.00E-06	0.350914908	-18.42076172
ENSG00000201916	Y_RNA	1.00E-06	0.351052	-18.42132522
ENSG00000252917	SNORA74	1.00E-06	0.351432	-18.42288604
ENSG00000273925	RP11-227D13.5	1.00E-06	0.351886	-18.42474859
ENSG00000267084	RP11-666A8.11	1.00E-06	0.352213	-18.42608863
ENSG00000264553	MIR4257	1.00E-06	0.352412	-18.42690352
ENSG00000242493	RN7SL37P	1.00E-06	0.352577	-18.42757884
ENSG00000236542	MED28P7	1.00E-06	0.353315	-18.43059547
ENSG00000200142	Y_RNA	1.00E-06	0.353441	-18.43110988
ENSG00000265592	RN7SL92P	1.00E-06	0.353659	-18.43199945
ENSG00000243705	RP11-178L8.1	1.00E-06	0.353729	-18.43228498
ENSG00000253708	RP11-51J9.4	1.00E-06	0.353932	-18.43311268
ENSG00000243384	RP11-475O23.2	1.00E-06	0.355649	-18.44009458
ENSG00000225460	RP13-93L13.1	1.00E-06	0.356056	-18.44174464
ENSG00000270789	RP11-632K20.8	1.00E-06	0.356294	-18.44270866
ENSG00000223576	RP11-61K9.2	1.00E-06	0.35676	-18.44459434
ENSG00000242659	RP11-271C24.2	1.00E-06	0.357231	-18.44649776
ENSG00000221760	MIR548J	1.00E-06	0.357582	-18.44791459
ENSG00000271333	RP11-382B18.5	1.00E-06	0.357858	-18.44902771
ENSG00000235939	RP11-123B3.2	1.00E-06	0.359421	-18.45531518
ENSG00000264222	RP11-757O6.1	1.00E-06	0.359922	-18.45732476
ENSG00000207730	MIR200B	1.00E-06	0.360803	-18.46085181
ENSG00000248133	MTND4LP22	1.00E-06	0.360803	-18.46085181
ENSG00000248432	RP11-269F21.2	1.00E-06	0.360803	-18.46085181
ENSG00000265751	RP11-296E23.1	1.00E-06	0.360832	-18.46096776
ENSG00000274001	RP11-5G9.5	1.00E-06	0.361607	-18.46406308
ENSG00000270722	U1	1.00E-06	0.363004	-18.46962592
ENSG00000278621	CTD-2033D15.3	1.00E-06	0.36302	-18.46968951
ENSG00000252634	RN7SKP219	1.00E-06	0.365679	-18.48021825
ENSG00000275254	FTX_3	1.00E-06	0.368167	-18.49000079
ENSG00000266599	RP11-466A19.3	1.00E-06	0.368762	-18.49233047
ENSG00000276883	AL137852.1	1.00E-06	0.369004	-18.49327693
ENSG00000225037	EIF1AX-AS1	1.00E-06	0.369394	-18.49480091
ENSG00000263849	MIR4744	1.00E-06	0.369603	-18.49561694
ENSG00000232754	RP1-85F18.6	1.00E-06	0.370037	-18.49731001
ENSG00000272426	RP11-108M9.6	1.00E-06	0.370689	-18.49984978
ENSG00000229646	RP11-330A16.1	1.00E-06	0.370689	-18.49984978
ENSG00000281791	AC091770.1	1.00E-06	0.370689	-18.49984978
ENSG00000252944	RNU6-897P	1.00E-06	0.371112	-18.50149513
ENSG00000206645	Y_RNA	1.00E-06	0.371415	-18.50267256
ENSG00000243687	RP11-432B6.1	1.00E-06	0.372794	-18.50801911
ENSG00000265962	GACAT2	1.00E-06	0.372955	-18.50864204
ENSG00000236434	RP11-296A18.6	1.00E-06	0.373246	-18.50976727

ENSG00000273577	AC021037.1	1.00E-06	0.373246	-18.50976727
ENSG00000260443	CTD-3057O21.1	1.00E-06	0.373246	-18.50976727
ENSG00000230226	RP4-697K14.3	1.00E-06	0.374238	-18.51359653
ENSG00000264540	RN7SL405P	1.00E-06	0.374537	-18.51474872
ENSG00000267376	ATP5LP6	1.00E-06	0.374952	-18.51634639
ENSG00000232347	RP11-488L18.8	1.00E-06	0.375866	-18.51985889
ENSG00000266969	RP11-773H22.4	1.00E-06	0.378789	-18.53103491
ENSG00000225450	RP3-508I15.14	1.00E-06	0.379374	-18.53326128
ENSG00000252722	SCARNA20	1.00E-06	0.380083	-18.53595497
ENSG00000275646	SMAD5-AS1_2	1.00E-06	0.380083	-18.53595497
ENSG00000264397	MIR3180-5	1.00E-06	0.380258	-18.53661907
ENSG00000252929	RNU6-218P	1.00E-06	0.381421	-18.54102475
ENSG00000233860	RP11-359D14.2	1.00E-06	0.382059	-18.54343592
ENSG00000279228	RP11-345J4.4	1.00E-06	0.383735	-18.54975083
ENSG00000278887	RP11-347C12.4	1.00E-06	0.383735	-18.54975083
ENSG00000277371	Metazoa_SRP	1.00E-06	0.383834	-18.55012299
ENSG00000222586	AC010999.1	1.00E-06	0.385516	-18.55643121
ENSG00000199709	RNU4-23P	1.00E-06	0.386575	-18.56038882
ENSG00000221303	SNORA79	1.00E-06	0.386575	-18.56038882
ENSG00000233299	HIGD1AP4	1.00E-06	0.386575	-18.56038882
ENSG00000199240	RNA5SP46	1.00E-06	0.387863	-18.56518763
ENSG00000276198	AC012476.1	1.00E-06	0.388256	-18.56664869
ENSG00000238171	AC068196.1	1.00E-06	0.389824	-18.57246339
ENSG00000277672	RP11-386G11.11	1.00E-06	0.390762	-18.57593065
ENSG00000281851	Z98744.3	1.00E-06	0.392178	-18.58114908
ENSG00000281884	Metazoa_SRP	1.00E-06	0.393604	-18.58638536
ENSG00000228204	RP4-724E13.2	1.00E-06	0.393899631	-18.58746854
ENSG00000254445	HSPB2-C11orf52	1.00E-06	0.394464477	-18.58953586
ENSG00000199627	RNU6-1010P	1.00E-06	0.394524	-18.58975354
ENSG00000279236	RP11-120K19.3	1.00E-06	0.394585	-18.58997659
ENSG00000213440	H2AFZP1	1.00E-06	0.3946	-18.59003143
ENSG00000207313	SNORA2B	1.00E-06	0.395041	-18.59164287
ENSG00000274761	AC005150.3	1.00E-06	0.397945	-18.60220952
ENSG00000281203	AC005024.1	1.00E-06	0.397945	-18.60220952
ENSG00000125954	CHURC1-FNTB	1.00E-06	0.399166201	-18.60663004
ENSG00000201544	SNORA16B	1.00E-06	0.400892	-18.6128541
ENSG00000206647	SNORA2	1.00E-06	0.400892	-18.6128541
ENSG00000206937	SNORA70B	1.00E-06	0.400892	-18.6128541
ENSG00000206869	SNORA70F	1.00E-06	0.400892	-18.6128541
ENSG00000207962	MIR30C1	1.00E-06	0.401344	-18.6144798
ENSG00000237738	RNF216-IT1	1.00E-06	0.401525	-18.61513029
ENSG00000200354	SNORA71D	1.00E-06	0.403884	-18.62358147
ENSG00000281030	AC006088.1	1.00E-06	0.404274	-18.6249739
ENSG00000272359	RNU4-89P	1.00E-06	0.406921	-18.63438921
ENSG00000259735	RP11-356M20.3	1.00E-06	0.407658	-18.6369998
ENSG00000242325	RPS12P31	1.00E-06	0.407943	-18.63800806
ENSG00000252496	RNA5SP33	1.00E-06	0.408665	-18.64055916
ENSG00000244310	RP11-734K21.3	1.00E-06	0.408845	-18.64119447

ENSG00000279707	RP11-380M21.1	1.00E-06	0.409229	-18.64254886
ENSG00000251899	AC104169.1	1.00E-06	0.410004	-18.64527846
ENSG00000207016	SNORA36C	1.00E-06	0.410004	-18.64527846
ENSG00000225551	RP11-19J3.5	1.00E-06	0.410004	-18.64527846
ENSG00000277524	SMAD5-AS1_3	1.00E-06	0.410268	-18.64620711
ENSG00000222430	Y_RNA	1.00E-06	0.411316	-18.64988767
ENSG00000243514	RPL32P33	1.00E-06	0.412086	-18.65258593
ENSG00000277396	Metazoa_SRP	1.00E-06	0.413133	-18.65624678
ENSG00000236140	RP11-89F3.2	1.00E-06	0.414782	-18.66199376
ENSG00000266872	RP11-19P22.8	1.00E-06	0.414943	-18.66255364
ENSG00000252777	SCARNA24	1.00E-06	0.416311	-18.66730215
ENSG00000252906	SCARNA3	1.00E-06	0.416311	-18.66730215
ENSG00000274245	RP11-357P18.2	1.00E-06	0.417991	-18.67311235
ENSG00000199697	RNU6-446P	1.00E-06	0.418668	-18.67544713
ENSG00000253058	RNA5SP437	1.00E-06	0.419539	-18.6784454
ENSG00000228471	AC018865.11	1.00E-06	0.419539	-18.6784454
ENSG00000207624	MIR194-1	1.00E-06	0.42023	-18.68081963
ENSG00000271018	RP11-60E8.2	1.00E-06	0.420626	-18.6821785
ENSG00000249412	CTC-563A5.2	1.00E-06	0.420702	-18.68243915
ENSG00000236900	TIMM9P1	1.00E-06	0.422817	-18.68967386
ENSG00000223254	Y_RNA	1.00E-06	0.424871	-18.69666535
ENSG00000280295	RP11-358B23.6	1.00E-06	0.425563	-18.6990132
ENSG00000264229	RNU4ATAC	1.00E-06	0.426146	-18.70098826
ENSG00000221245	SNORA11	1.00E-06	0.426146	-18.70098826
ENSG00000237137	RP11-408A13.2	1.00E-06	0.426146	-18.70098826
ENSG00000234812	RP11-146D12.6	1.00E-06	0.427268	-18.70478175
ENSG00000276257	RP11-327I22.3	1.00E-06	0.427268	-18.70478175
ENSG00000231307	RPS3P2	1.00E-06	0.427715	-18.70629028
ENSG00000251783	RNU6-1170P	1.00E-06	0.428879	-18.71021115
ENSG00000200839	RNA5SP48	1.00E-06	0.429528	-18.71239266
ENSG00000279963	AL049872.1	1.00E-06	0.429528	-18.71239266
ENSG00000222872	RNU4-78P	1.00E-06	0.429528	-18.71239266
ENSG00000280625	AC092944.1	1.00E-06	0.429528	-18.71239266
ENSG00000223280	RNU6-57P	1.00E-06	0.430862	-18.71686634
ENSG00000231335	AC107072.2	1.00E-06	0.432613	-18.72271749
ENSG00000239912	RPL39P36	1.00E-06	0.433658	-18.7261982
ENSG00000277579	CTC-268N12.3	1.00E-06	0.434289	-18.72829589
ENSG00000212624	SNORA26	1.00E-06	0.434738	-18.72978668
ENSG00000266778	AC068312.1	1.00E-06	0.435196	-18.73130577
ENSG00000201863	SNORA51	1.00E-06	0.436456	-18.73547669
ENSG00000233851	LATS2-AS1	1.00E-06	0.436732	-18.73638872
ENSG00000253618	GRPEL2-AS1	1.00E-06	0.436822	-18.73668599
ENSG00000270118	RP11-57A19.5	1.00E-06	0.437633	-18.739362
ENSG00000243560	RN7SL364P	1.00E-06	0.43774	-18.7397147
ENSG00000233603	JTBP1	1.00E-06	0.438223	-18.74130568
ENSG00000249791	CTC-428G20.2	1.00E-06	0.438815	-18.74325332
ENSG00000222085	AC024568.1	1.00E-06	0.440004	-18.74715711
ENSG00000221611	SNORD88	1.00E-06	0.440101	-18.74747512

ENSG00000256427	RP11-118B22.4	1.00E-06	0.444561	-18.76202187
ENSG00000206991	RNU6-610P	1.00E-06	0.445104	-18.76378294
ENSG00000206589	RNU6-354P	1.00E-06	0.445104	-18.76378294
ENSG00000207052	RNU6-378P	1.00E-06	0.445104	-18.76378294
ENSG00000274289	AL365510.1	1.00E-06	0.447082	-18.77017994
ENSG00000212237	RNA5SP18	1.00E-06	0.447277	-18.77080905
ENSG00000273212	AC000068.10	1.00E-06	0.448133	-18.77356744
ENSG00000280685	AP000887.1	1.00E-06	0.449086	-18.77663222
ENSG00000271732	RP5-1182A14.5	1.00E-06	0.450206	-18.78022576
ENSG00000207789	MIR26A2	1.00E-06	0.451005	-18.7827839
ENSG00000201704	RNA5SP396	1.00E-06	0.451005	-18.7827839
ENSG00000201736	RNA5SP160	1.00E-06	0.451005	-18.7827839
ENSG00000201420	RNA5SP512	1.00E-06	0.451005	-18.7827839
ENSG00000235385	GS1-600G8.5	1.00E-06	0.451873825	-18.78556046
ENSG00000269321	CTC-453G23.4	1.00E-06	0.45226	-18.78679288
ENSG00000278151	FAM13A-AS1_1	1.00E-06	0.454794	-18.7948537
ENSG00000199646	RNU6-1272P	1.00E-06	0.45522	-18.79620442
ENSG00000252390	RNU5F-4P	1.00E-06	0.455752	-18.79788946
ENSG00000274804	AL353644.3	1.00E-06	0.456888	-18.80148103
ENSG00000271415	RPL23AP90	1.00E-06	0.458649	-18.80703097
ENSG00000279962	RP11-720L3.1	1.00E-06	0.458649	-18.80703097
ENSG00000252297	RNU6-875P	1.00E-06	0.459514	-18.80974929
ENSG00000261244	RP11-114H24.7	1.00E-06	0.459948	-18.81111124
ENSG00000265820	MIR3177	1.00E-06	0.462004	-18.81754582
ENSG00000233158	RPS24P6	1.00E-06	0.462731	-18.81981423
ENSG00000259202	RP11-342M21.2	1.00E-06	0.463325	-18.821665
ENSG00000261267	RP11-44I10.3	1.00E-06	0.464669	-18.82584387
ENSG00000224349	RP11-462G2.2	1.00E-06	0.465219	-18.82755049
ENSG00000272439	RNU6-91P	1.00E-06	0.465336	-18.82791328
ENSG00000221333	MIR548K	1.00E-06	0.466556	-18.83169073
ENSG00000206863	RNU5A-6P	1.00E-06	0.466556	-18.83169073
ENSG00000223138	RNA5SP450	1.00E-06	0.466923	-18.83282513
ENSG00000264384	RN7SL431P	1.00E-06	0.468453	-18.83754478
ENSG00000211514	MIR454	1.00E-06	0.470614	-18.84418471
ENSG00000211581	MIR765	1.00E-06	0.474741	-18.85678112
ENSG00000223262	RNA5SP492	1.00E-06	0.474741	-18.85678112
ENSG00000268575	RP1-283E3.8	1.00E-06	0.474923	-18.8573341
ENSG00000119147	C2orf40	1.00E-06	0.475784663	-18.85994924
ENSG00000265102	MIR3942	1.00E-06	0.476658	-18.86259498
ENSG00000212331	RNA5SP297	1.00E-06	0.478943	-18.86949444
ENSG00000200138	RNY1P10	1.00E-06	0.478943	-18.86949444
ENSG00000206713	Y_RNA	1.00E-06	0.478943	-18.86949444
ENSG00000202417	Y_RNA	1.00E-06	0.478943	-18.86949444
ENSG00000267323	SLC25A1P5	1.00E-06	0.479689	-18.87173983
ENSG00000217653	RP11-63K6.1	1.00E-06	0.480275	-18.87350119
ENSG00000226767	RP11-328P23.3	1.00E-06	0.482469	-18.88007672
ENSG00000206791	RNU1-129P	1.00E-06	0.482872	-18.88128128
ENSG00000235627	SNRPFP4	1.00E-06	0.483059	-18.88183988

ENSG00000202251	Y_RNA	1.00E-06	0.48322	-18.88232064
ENSG00000229044	RP11-266K22.2	1.00E-06	0.48417	-18.88515416
ENSG00000231018	EIF4A1P3	1.00E-06	0.485014	-18.88766687
ENSG00000251864	Y_RNA	1.00E-06	0.485567	-18.88931085
ENSG00000266862	AC004595.1	1.00E-06	0.487085	-18.89381403
ENSG00000264045	AC073127.1	1.00E-06	0.487085	-18.89381403
ENSG00000221039	MIR1286	1.00E-06	0.487572	-18.89525575
ENSG00000280215	RP11-279N21.1	1.00E-06	0.487572	-18.89525575
ENSG00000265031	MIR1273E	1.00E-06	0.488146	-18.89695318
ENSG00000252622	RNU6-881P	1.00E-06	0.488146	-18.89695318
ENSG00000281278	AC139451.2	1.00E-06	0.489662	-18.90142672
ENSG00000278375	AP000721.1	1.00E-06	0.490692	-18.90445823
ENSG00000266107	MIR4525	1.00E-06	0.490692	-18.90445823
ENSG00000276580	MIR8055	1.00E-06	0.49099	-18.90533412
ENSG00000207440	RNU6-541P	1.00E-06	0.492004	-18.90831052
ENSG00000244730	RP13-1056D16.2	1.00E-06	0.492004	-18.90831052
ENSG00000207009	Y_RNA	1.00E-06	0.492004	-18.90831052
ENSG00000200091	RN7SKP163	1.00E-06	0.492004	-18.90831052
ENSG00000252062	RNU6-469P	1.00E-06	0.49298	-18.91116959
ENSG00000272762	RP11-155D18.12	1.00E-06	0.494008	-18.91417488
ENSG00000238966	SNORD112	1.00E-06	0.496105	-18.92028597
ENSG00000253039	RNA5SP47	1.00E-06	0.496519	-18.9214894
ENSG00000207729	MIR556	1.00E-06	0.496519	-18.9214894
ENSG00000202100	Y_RNA	1.00E-06	0.496519	-18.9214894
ENSG00000235183	RP11-613C6.4	1.00E-06	0.496519	-18.9214894
ENSG00000275845	AC096922.2	1.00E-06	0.496519	-18.9214894
ENSG00000252641	RNU6-678P	1.00E-06	0.496519	-18.9214894
ENSG00000266252	AL031768.1	1.00E-06	0.497909	-18.92552257
ENSG00000234219	CDCA4P4	1.00E-06	0.499819	-18.93104622
ENSG00000222344	RNU6-613P	1.00E-06	0.501116	-18.93478508
ENSG00000201618	RNA5SP505	1.00E-06	0.501116	-18.93478508
ENSG00000228812	LAMA5-AS1	1.00E-06	0.501337809	-18.93542352
ENSG00000262343	RP11-353N14.3	1.00E-06	0.50368	-18.94214792
ENSG00000280357	RP11-269C23.4	1.00E-06	0.503777	-18.94242573
ENSG00000230355	AC009474.2	1.00E-06	0.504538	-18.94460341
ENSG00000207441	RNU6-21P	1.00E-06	0.5058	-18.94820751
ENSG00000201113	RNU6-647P	1.00E-06	0.5058	-18.94820751
ENSG00000207087	RNU6-242P	1.00E-06	0.5058	-18.94820751
ENSG00000252021	Y_RNA	1.00E-06	0.5058	-18.94820751
ENSG00000276147	MIR548AY	1.00E-06	0.5058	-18.94820751
ENSG00000253437	RNU6-988P	1.00E-06	0.5058	-18.94820751
ENSG00000201548	Y_RNA	1.00E-06	0.50738	-18.95270713
ENSG00000252008	RNU6-927P	1.00E-06	0.508733	-18.95654916
ENSG00000263678	AC072031.1	1.00E-06	0.509369	-18.95835163
ENSG00000265324	AC005020.1	1.00E-06	0.509369	-18.95835163
ENSG00000252377	RNU6-504P	1.00E-06	0.509982	-18.9600868
ENSG00000222398	RNU6-554P	1.00E-06	0.510571	-18.96175207
ENSG00000199570	RNU6-228P	1.00E-06	0.510571	-18.96175207

ENSG00000240435	RPS12P27	1.00E-06	0.512298	-18.96662373
ENSG00000266594	MIR4766	1.00E-06	0.512721	-18.96781446
ENSG00000221070	AC008625.1	1.00E-06	0.512721	-18.96781446
ENSG00000227050	RP11-460I13.2	1.00E-06	0.513033	-18.9686921
ENSG00000254274	TDGF1P5	1.00E-06	0.513727	-18.97064237
ENSG00000277325	MIR6756	1.00E-06	0.515433	-18.97542538
ENSG00000223113	RNA5SP247	1.00E-06	0.515433	-18.97542538
ENSG00000278399	RP11-74M13.5	1.00E-06	0.516481	-18.97835575
ENSG00000241499	RP11-85G20.2	1.00E-06	0.517075	-18.98001403
ENSG00000259528	RP11-272D12.2	1.00E-06	0.51929	-18.98618092
ENSG00000200575	RNU6-414P	1.00E-06	0.52039	-18.98923371
ENSG00000206887	RNU6-1008P	1.00E-06	0.52039	-18.98923371
ENSG00000200560	RNU6-288P	1.00E-06	0.52039	-18.98923371
ENSG00000200086	RNU6-433P	1.00E-06	0.52039	-18.98923371
ENSG00000200882	RNU6-681P	1.00E-06	0.52039	-18.98923371
ENSG00000262265	RP5-867C24.4	1.00E-06	0.523197	-18.99699474
ENSG00000199201	Y_RNA	1.00E-06	0.525442	-19.003172
ENSG00000271762	RP11-213H15.4	1.00E-06	0.526612	-19.00638087
ENSG00000265672	MIR4722	1.00E-06	0.528382	-19.01122179
ENSG00000276325	AC131160.2	1.00E-06	0.53016	-19.0160683
ENSG00000207092	Y_RNA	1.00E-06	0.530593	-19.01724612
ENSG00000207049	Y_RNA	1.00E-06	0.530593	-19.01724612
ENSG00000216136	AC092896.1	1.00E-06	0.530593	-19.01724612
ENSG00000206604	RNU6-425P	1.00E-06	0.530593	-19.01724612
ENSG00000200847	Y_RNA	1.00E-06	0.530593	-19.01724612
ENSG00000207363	Y_RNA	1.00E-06	0.530593	-19.01724612
ENSG00000254495	AP000487.4	1.00E-06	0.532964	-19.02367856
ENSG00000238344	SNORD126	1.00E-06	0.534176	-19.02695563
ENSG00000226944	RP1-120G22.11	1.00E-06	0.535062	-19.02934655
ENSG00000254606	RP11-22P4.2	1.00E-06	0.535504	-19.03053783
ENSG00000280570	AC117945.1	1.00E-06	0.535847	-19.0314616
ENSG00000238366	Y_RNA	1.00E-06	0.535847	-19.0314616
ENSG00000207352	RNU6-540P	1.00E-06	0.535847	-19.0314616
ENSG00000276621	AC100821.1	1.00E-06	0.535847	-19.0314616
ENSG00000252723	RNU6-677P	1.00E-06	0.535847	-19.0314616
ENSG00000272788	RP11-674N23.4	1.00E-06	0.537875	-19.03691141
ENSG00000206816	Y_RNA	1.00E-06	0.541205	-19.04581564
ENSG00000238444	RNU6-893P	1.00E-06	0.541205	-19.04581564
ENSG00000275940	MRPL57P3	1.00E-06	0.541205	-19.04581564
ENSG00000268471	DKFZP434I0714	1.00E-06	0.541205	-19.04581564
ENSG00000242651	RN7SL862P	1.00E-06	0.544838	-19.0554678
ENSG00000210077	MT-TV	1.00E-06	0.545983	-19.05849651
ENSG00000266075	RN7SL574P	1.00E-06	0.546672	-19.06031596
ENSG00000252319	Y_RNA	1.00E-06	0.546672	-19.06031596
ENSG00000265891	AC017028.7	1.00E-06	0.546672	-19.06031596
ENSG00000252606	RNU6-1150P	1.00E-06	0.546672	-19.06031596
ENSG00000221261	MIR1208	1.00E-06	0.548619	-19.06544506
ENSG00000242085	RPS20P33	1.00E-06	0.550379	-19.0700659

ENSG00000266825	AL590085.1	1.00E-06	0.55225	-19.07496199
ENSG00000265679	AL139328.1	1.00E-06	0.55225	-19.07496199
ENSG00000274668	AC015909.1	1.00E-06	0.55225	-19.07496199
ENSG00000267108	RP11-861E21.1	1.00E-06	0.55225	-19.07496199
ENSG00000252102	SNORA63	1.00E-06	0.55225	-19.07496199
ENSG00000241693	RN7SL704P	1.00E-06	0.553057	-19.07706865
ENSG00000275789	MIR8064	1.00E-06	0.553232	-19.07752508
ENSG00000222160	Y_RNA	1.00E-06	0.553388	-19.07793183
ENSG00000186105	LRRC70	1.00E-06	0.553478	-19.07816645
ENSG00000221874	ZNF816-ZNF321P	1.00E-06	0.554306	-19.0803231
ENSG00000236073	RP11-487E1.2	1.00E-06	0.557676	-19.08906766
ENSG00000234272	RPL30P2	1.00E-06	0.557944	-19.0897608
ENSG00000207701	MIR597	1.00E-06	0.557944	-19.0897608
ENSG00000201451	Y_RNA	1.00E-06	0.557944	-19.0897608
ENSG00000282022	RP11-259O18.5	1.00E-06	0.558349	-19.09080765
ENSG00000211878	TRAJ11	1.00E-06	0.559246	-19.09312351
ENSG00000270512	RP5-1136G2.1	1.00E-06	0.562569	-19.10167053
ENSG00000199605	RNY4P24	1.00E-06	0.563755	-19.1047088
ENSG00000212264	SNORD65	1.00E-06	0.564073	-19.10552236
ENSG00000261872	RP5-867C24.5	1.00E-06	0.564986	-19.10785559
ENSG00000242889	RN7SL449P	1.00E-06	0.567698	-19.11476413
ENSG00000212325	Y_RNA	1.00E-06	0.56969	-19.11981756
ENSG00000273725	Metazoa_SRP	1.00E-06	0.56969	-19.11981756
ENSG00000221739	MIR1203	1.00E-06	0.573041	-19.12827884
ENSG00000263620	RP11-599B13.6	1.00E-06	0.573366	-19.12909683
ENSG00000255520	RP11-390K5.3	1.00E-06	0.573651	-19.12981377
ENSG00000276573	RP11-128N14.5	1.00E-06	0.577609	-19.1397337
ENSG00000255972	RP11-324E6.8	1.00E-06	0.579863	-19.14535256
ENSG00000226302	RP11-528N21.1	1.00E-06	0.581211	-19.14870248
ENSG00000216191	MIR8485	1.00E-06	0.582837	-19.15273294
ENSG00000281551	snoMe28S-Am2634	1.00E-06	0.582837	-19.15273294
ENSG00000254094	AC078852.1	1.00E-06	0.585364	-19.1589745
ENSG00000269971	RP3-426I6.5	1.00E-06	0.588266	-19.16610913
ENSG00000249019	RP11-539G18.1	1.00E-06	0.588349	-19.16631267
ENSG00000263982	RP11-504I13.3	1.00E-06	0.588644	-19.16703586
ENSG00000201301	RNA5SP130	1.00E-06	0.58868	-19.16712409
ENSG00000265621	AC013476.1	1.00E-06	0.589312	-19.16867212
ENSG00000220240	RP11-76H14.5	1.00E-06	0.59256	-19.17660172
ENSG00000267168	RP11-318A15.7	1.00E-06	0.592794	-19.17717132
ENSG00000200737	Y_RNA	1.00E-06	0.593889	-19.17983379
ENSG00000200985	RNA5SP493	1.00E-06	0.594732	-19.18188018
ENSG00000265267	AL121652.3	1.00E-06	0.595934	-19.18479303
ENSG00000264678	MIR3140	1.00E-06	0.60134	-19.1978214
ENSG00000252658	RNU6-786P	1.00E-06	0.60134	-19.1978214
ENSG00000265690	RP11-5A19.5	1.00E-06	0.602778142	-19.20126758
ENSG00000210741	MIR196A1	1.00E-06	0.603058	-19.20193724
ENSG00000224419	KRT18P27	1.00E-06	0.603682	-19.20342926
ENSG00000255886	RP11-196H14.2	1.00E-06	0.60442	-19.20519187

ENSG00000276978	AC083826.1	1.00E-06	0.60767	-19.21292854
ENSG00000222601	Y_RNA	1.00E-06	0.608096	-19.21393957
ENSG00000277900	Metazoa_SRP	1.00E-06	0.608138	-19.21403921
ENSG00000201592	snoU2_19	1.00E-06	0.608856	-19.21574153
ENSG00000276942	AL591428.1	1.00E-06	0.609633	-19.21758147
ENSG00000264994	SNORD92	1.00E-06	0.611244	-19.22138887
ENSG00000275912	AC004837.1	1.00E-06	0.615006	-19.23024096
ENSG00000207493	SNORA46	1.00E-06	0.617375	-19.23578754
ENSG00000278359	MIR7155	1.00E-06	0.618521	-19.23846305
ENSG00000238829	RNU7-45P	1.00E-06	0.618521	-19.23846305
ENSG00000278040	uc_338	1.00E-06	0.618521	-19.23846305
ENSG00000222364	RNU6-96P	1.00E-06	0.620131	-19.24221349
ENSG00000212461	SNORA17	1.00E-06	0.620917	-19.24404091
ENSG00000265647	AL358815.1	1.00E-06	0.623207	-19.24935191
ENSG00000239671	CTD-3193K9.1	1.00E-06	0.627484	-19.25921915
ENSG00000264354	MIR3134	1.00E-06	0.628968	-19.26262709
ENSG00000251779	Y_RNA	1.00E-06	0.629566	-19.2639981
ENSG00000199836	RNU1-47P	1.00E-06	0.630853	-19.26694435
ENSG00000171570	RAB4B-EGLN2	1.00E-06	0.632428837	-19.27054363
ENSG00000277962	AC127030.1	1.00E-06	0.633845	-19.27377056
ENSG00000278504	UPF3BP2	1.00E-06	0.635329	-19.27714435
ENSG00000238460	AC068587.2	1.00E-06	0.636282	-19.27930678
ENSG00000275942	MIAT_exon5_2	1.00E-06	0.636712	-19.28028143
ENSG00000199169	MIR367	1.00E-06	0.636712	-19.28028143
ENSG00000256007	ARAP1-AS1	1.00E-06	0.638356	-19.28400169
ENSG00000276752	MIR29B2	1.00E-06	0.644292	-19.29735516
ENSG00000251892	RNU7-84P	1.00E-06	0.645955	-19.30107414
ENSG00000228728	RP11-316K19.3	1.00E-06	0.646379	-19.3020208
ENSG00000281646	AC008781.1	1.00E-06	0.652055	-19.31463413
ENSG00000237003	RP4-612C19.1	1.00E-06	0.652963	-19.31664172
ENSG00000238516	Y_RNA	1.00E-06	0.653733	-19.318342
ENSG00000238737	AC100756.1	1.00E-06	0.654229	-19.31943619
ENSG00000236890	XXbac-B476C20.11	1.00E-06	0.654601	-19.32025628
ENSG00000223238	RNA5SP294	1.00E-06	0.656007	-19.32335168
ENSG00000264075	MIR4783	1.00E-06	0.660006	-19.33211961
ENSG00000228526	MIR34AHG	1.00E-06	0.660434226	-19.33305536
ENSG00000281359	AC104650.2	1.00E-06	0.662484	-19.33752609
ENSG00000235258	NDUFB4P6	1.00E-06	0.665416	-19.34389703
ENSG00000221230	MIR548L	1.00E-06	0.667067	-19.34747215
ENSG00000207612	MIR604	1.00E-06	0.66787	-19.34920779
ENSG00000270269	IMMP1LP1	1.00E-06	0.672071	-19.35825413
ENSG00000201227	RNA5-8SP3	1.00E-06	0.672305	-19.35875635
ENSG00000200999	SNORD74	1.00E-06	0.676507	-19.36774534
ENSG00000273090	RP11-78I14.1	1.00E-06	0.677073	-19.36895186
ENSG00000206784	Y_RNA	1.00E-06	0.680099	-19.37538524
ENSG00000226999	AC073325.2	1.00E-06	0.680762	-19.37679098
ENSG00000235189	RP4-537K23.4	1.00E-06	0.683321	-19.38220394
ENSG00000279995	RP11-1250I15.1	1.00E-06	0.683801	-19.38321701

ENSG00000279074	RP11-322E11.3	1.00E-06	0.68507	-19.38589188
ENSG00000269482	Z69720.2	1.00E-06	0.687973	-19.39199242
ENSG00000241464	RPL39P38	1.00E-06	0.69095	-19.39822179
ENSG00000202408	RNU1-122P	1.00E-06	0.693853	-19.40427052
ENSG00000260593	RP11-432I5.2	1.00E-06	0.698329	-19.41354736
ENSG00000200842	Y_RNA	1.00E-06	0.699257	-19.41546327
ENSG00000234112	RP11-145A3.4	1.00E-06	0.702649	-19.42244466
ENSG00000264173	MIR3175	1.00E-06	0.702864	-19.42288604
ENSG00000280746	AC024896.1	1.00E-06	0.70773	-19.43283955
ENSG00000200394	SNORA38B	1.00E-06	0.710591	-19.43865989
ENSG00000281855	AC105009.1	1.00E-06	0.710996	-19.43948192
ENSG00000275162	LLNLR-260G6.1	1.00E-06	0.712371	-19.44226926
ENSG00000231241	RPS3AP3	1.00E-06	0.715643	-19.44888055
ENSG00000272158	RP11-840I19.5	1.00E-06	0.720792	-19.45922347
ENSG00000281182	AC087392.1	1.00E-06	0.721608	-19.46085581
ENSG00000278914	KB-1517D11.3	1.00E-06	0.721608	-19.46085581
ENSG00000238854	SNORD5	1.00E-06	0.721608	-19.46085581
ENSG00000235852	AC005540.3	1.00E-06	0.726501	-19.47060526
ENSG00000228487	RP13-225O21.2	1.00E-06	0.728476291	-19.47452249
ENSG00000275017	RP11-317B17.4	1.00E-06	0.730799	-19.47911513
ENSG00000252316	RNY4	1.00E-06	0.732882	-19.48322141
ENSG00000265106	Z93241.1	1.00E-06	0.737549	-19.49237937
ENSG00000274389	MIR302B	1.00E-06	0.741378	-19.49984978
ENSG00000277827	AC124864.1	1.00E-06	0.742224	-19.50149513
ENSG00000240808	CTD-3236F5.1	1.00E-06	0.742758	-19.50253271
ENSG00000250956	CTB-88F18.3	1.00E-06	0.74649	-19.50976341
ENSG00000274949	RP5-1009E24.9	1.00E-06	0.751621	-19.51964585
ENSG00000261002	RP11-546B15.1	1.00E-06	0.752723464	-19.52176042
ENSG00000279238	AL138706.2	1.00E-06	0.754717	-19.52557625
ENSG00000237080	EHMT2-AS1	1.00E-06	0.756522	-19.52902251
ENSG00000206650	SNORA70G	1.00E-06	0.762261	-19.53992554
ENSG00000271490	RP11-60E8.4	1.00E-06	0.767146	-19.54914165
ENSG00000252700	RNU7-110P	1.00E-06	0.768162	-19.55105107
ENSG00000249870	RP11-481C4.1	1.00E-06	0.769327	-19.55323742
ENSG00000266041	MIR4690	1.00E-06	0.771598	-19.55748988
ENSG00000188223	AC002398.9	1.00E-06	0.772168	-19.55855524
ENSG00000278524	MIR6810	1.00E-06	0.773151	-19.56039068
ENSG00000201821	RNU4-9P	1.00E-06	0.778712	-19.57073033
ENSG00000199872	RNU6-942P	1.00E-06	0.778931	-19.57113601
ENSG00000222915	RNU6-564P	1.00E-06	0.780585	-19.57419621
ENSG00000266498	RP11-45M22.5	1.00E-06	0.787351	-19.58664741
ENSG00000252904	SNORA76	1.00E-06	0.790081	-19.59164104
ENSG00000227383	RP11-571F15.2	1.00E-06	0.79112	-19.59353702
ENSG00000252817	AL590431.1	1.00E-06	0.794681	-19.60001633
ENSG00000208892	SNORA49	1.00E-06	0.79589	-19.60220952
ENSG00000233330	RP1-12G14.7	1.00E-06	0.795986	-19.60238353
ENSG00000179766	ATP8B5P	1.00E-06	0.809685807	-19.62700266
ENSG00000258311	RP11-644F5.10	1.00E-06	0.814457865	-19.63548054

ENSG00000261528	AC002400.1	1.00E-06	0.820008	-19.64527846
ENSG00000232179	MTATP6P29	1.00E-06	0.820008	-19.64527846
ENSG00000264095	AP000560.1	1.00E-06	0.828873	-19.66079154
ENSG00000278925	AC005753.1	1.00E-06	0.835453	-19.67219914
ENSG00000252363	RNU7-43P	1.00E-06	0.845633	-19.68967215
ENSG00000271014	RP11-717A5.1	1.00E-06	0.845633	-19.68967215
ENSG00000238719	RNU7-96P	1.00E-06	0.845633	-19.68967215
ENSG00000260635	RP11-21M24.3	1.00E-06	0.85013	-19.69732395
ENSG00000275108	U7	1.00E-06	0.859056	-19.71239266
ENSG00000206762	RNU6-418P	1.00E-06	0.85986	-19.71374226
ENSG00000259799	RP11-554A11.9	1.00E-06	0.869674032	-19.73011523
ENSG00000187762	HSPE1P11	1.00E-06	0.872912	-19.73547669
ENSG00000207588	MIR593	1.00E-06	0.876753	-19.74181094
ENSG00000283036	RP11-101C21.4	1.00E-06	0.878541	-19.74475009
ENSG00000221944	TIGD1	1.00E-06	0.881598	-19.74976143
ENSG00000252328	Vault	1.00E-06	0.883601	-19.75303553
ENSG00000251798	RNU6-863P	1.00E-06	0.884663	-19.75476846
ENSG00000278590	RN7SL113P	1.00E-06	0.890545	-19.76432899
ENSG00000279807	CTC-510F12.3	1.00E-06	0.897037	-19.77480797
ENSG00000266370	MIR3657	1.00E-06	0.897383	-19.77536433
ENSG00000211513	MIR320E	1.00E-06	0.898787	-19.77761973
ENSG00000201028	RNU6-151P	1.00E-06	0.900323	-19.78008315
ENSG00000206651	Y_RNA	1.00E-06	0.900412	-19.78022576
ENSG00000234193	AC097461.4	1.00E-06	0.900434	-19.78026101
ENSG00000207956	MIR579	1.00E-06	0.905691	-19.7886594
ENSG00000274520	Six3os1_1	1.00E-06	0.908229	-19.79269658
ENSG00000280729	AL160231.1	1.00E-06	0.909225	-19.79427783
ENSG00000201728	RNA5SP479	1.00E-06	0.915886	-19.80480851
ENSG00000276176	MIR6090	1.00E-06	0.920049	-19.81135117
ENSG00000201294	RNU6-1019P	1.00E-06	0.920555	-19.81214439
ENSG00000200003	RNU6-986P	1.00E-06	0.923232	-19.8163337
ENSG00000202279	Y_RNA	1.00E-06	0.927781	-19.82342478
ENSG00000202222	Y_RNA	1.00E-06	0.929149	-19.82555044
ENSG00000273184	RP11-212P7.3	1.00E-06	0.935809	-19.83585458
ENSG00000238622	SNORD97	1.00E-06	0.949724	-19.85714879
ENSG00000206859	RNU6-767P	1.00E-06	0.950903	-19.85893866
ENSG00000277937	AC103724.1	1.00E-06	0.955642	-19.86611074
ENSG00000199875	Y_RNA	1.00E-06	0.957886	-19.86949444
ENSG00000264535	AC093788.1	1.00E-06	0.968473	-19.8853523
ENSG00000258615	RP11-725G5.3	1.00E-06	0.979429	-19.90158139
ENSG00000252487	RNY4P20	1.00E-06	0.989301	-19.91605001
ENSG00000207926	MIR135A1	1.00E-06	0.998223	-19.92900262
ENSG00000259052	AL157871.2	1.00E-06	1.004096	-19.93746578
ENSG00000252262	RNA5SP61	1.00E-06	1.006642	-19.94111927
ENSG00000207248	RNU6-1005P	1.00E-06	1.011598	-19.94820466
ENSG00000206848	RNU6-890P	1.00E-06	1.011598	-19.94820466
ENSG00000232608	TIMM9P2	1.00E-06	1.014437	-19.95224784
ENSG00000200816	SNORA38	1.00E-06	1.02501	-19.96720655

ENSG00000233799	AC139887.4	1.00E-06	1.04048	-19.9888178
ENSG00000201563	Y_RNA	1.00E-06	1.040779	-19.98923233
ENSG00000204666	FLJ26850	1.00E-06	1.041188	-19.98979916
ENSG00000200788	Y_RNA	1.00E-06	1.053674	-20.00699714
ENSG00000271509	RP11-382A18.3	1.00E-06	1.056391	-20.01071248
ENSG00000281443	AP001187.1	1.00E-06	1.061187	-20.01724748
ENSG00000199732	Y_RNA	1.00E-06	1.061187	-20.01724748
ENSG00000253030	MIR2116	1.00E-06	1.068881	-20.02766981
ENSG00000223188	Y_RNA	1.00E-06	1.072294	-20.03226909
ENSG00000201376	SNORA70	1.00E-06	1.082411	-20.04581698
ENSG00000206881	RNU6-190P	1.00E-06	1.082411	-20.04581698
ENSG00000264373	RP11-227G15.6	1.00E-06	1.094437	-20.06175748
ENSG00000252391	RNU6-638P	1.00E-06	1.113634	-20.08684373
ENSG00000207003	RNU6-611P	1.00E-06	1.113634	-20.08684373
ENSG00000200972	RNU5A-8P	1.00E-06	1.129885	-20.10774451
ENSG00000201207	Y_RNA	1.00E-06	1.129885	-20.10774451
ENSG00000183542	KLRC4	1.00E-06	1.13512	-20.11441339
ENSG00000253022	RNU6-731P	1.00E-06	1.170174	-20.15829164
ENSG00000200355	SNORA72	1.00E-06	1.174709	-20.16387198
ENSG00000207199	SNORD38	1.00E-06	1.176534	-20.16611158
ENSG00000274694	MIR6873	1.00E-06	1.177637	-20.16746347
ENSG00000201957	SNORA25	1.00E-06	1.183887	-20.17509995
ENSG00000231296	RP1-39G22.4	1.00E-06	1.193072	-20.18624968
ENSG00000222370	SNORA36B	1.00E-06	1.206351	-20.2022183
ENSG00000232698	AP001058.3	1.00E-06	1.22244	-20.22133223
ENSG00000202255	Y_RNA	1.00E-06	1.237041	-20.23846189
ENSG00000214889	RPS9P1	1.00E-06	1.239914	-20.24180863
ENSG00000265321	MIR4263	1.00E-06	1.251944	-20.2557386
ENSG00000222588	SNORA19	1.00E-06	1.258618	-20.26340905
ENSG00000278392	AL031320.1	1.00E-06	1.262813	-20.26820959
ENSG00000259132	RP11-298I3.5	1.00E-06	1.275607	-20.28275249
ENSG00000281239	AC016987.1	1.00E-06	1.283923	-20.29212725
ENSG00000250197	HMGN1P15	1.00E-06	1.284216	-20.29245645
ENSG00000207551	MIR608	1.00E-06	1.288069	-20.29677845
ENSG00000201458	RNU4-4P	1.00E-06	1.290567	-20.29957361
ENSG00000206897	SNORA9	1.00E-06	1.300016	-20.31009795
ENSG00000231795	ITCH-IT1	1.00E-06	1.304745	-20.31533644
ENSG00000264314	MIR548AT	1.00E-06	1.32502	-20.33758271
ENSG00000258728	RP11-195F19.29	1.00E-06	1.325496	-20.33810089
ENSG00000275640	MIR6793	1.00E-06	1.340127	-20.3539383
ENSG00000263793	MIR3115	1.00E-06	1.343344	-20.35739736
ENSG00000206913	SNORA7	1.00E-06	1.354961	-20.3698199
ENSG00000265724	MIR4284	1.00E-06	1.363035	-20.37839118
ENSG00000222249	RNU6-262P	1.00E-06	1.371675	-20.38750726
ENSG00000273196	RP11-717A5.2	1.00E-06	1.423444	-20.44095431
ENSG00000252756	RNU6-577P	1.00E-06	1.424805	-20.44233305
ENSG00000271977	AC226119.4	1.00E-06	1.433301	-20.45091018
ENSG00000272923	RP11-391L3.1	1.00E-06	1.444223	-20.46186209

ENSG00000207997	MIR644A	1.00E-06	1.45089	-20.46850671
ENSG00000257531	RP3-405J10.2	1.00E-06	1.466601	-20.484045
ENSG00000277723	MIR6827	1.00E-06	1.476849	-20.4940909
ENSG00000274986	MIR6750	1.00E-06	1.515375	-20.53124342
ENSG00000223086	RNA5SP155	1.00E-06	1.527122	-20.54238389
ENSG00000264496	AC108218.1	1.00E-06	1.538722	-20.55330117
ENSG00000239317	RP11-449H3.2	1.00E-06	1.541753	-20.55614022
ENSG00000216901	ZNF603P	1.00E-06	1.555827	-20.56925022
ENSG00000280697	Y_RNA	1.00E-06	1.564789	-20.5775367
ENSG00000252474	RNU6-539P	1.00E-06	1.576327	-20.58813541
ENSG00000206635	RNU6-1062P	1.00E-06	1.58821	-20.59897025
ENSG00000251730	SNORA4	1.00E-06	1.597732	-20.607594
ENSG00000208037	MIR320A	1.00E-06	1.623616	-20.63077903
ENSG00000259171	RP11-903H12.5	1.00E-06	1.624686	-20.63172949
ENSG00000239002	SCARNA10	1.00E-06	1.63353	-20.63956152
ENSG00000250135	RP4-622L5.2	1.00E-06	1.642035	-20.64705345
ENSG00000281135	MIR664B	1.00E-06	1.648924	-20.65309347
ENSG00000207008	SNORA54	1.00E-06	1.654416	-20.65789061
ENSG00000282977	PCBP2-OT1	1.00E-06	1.663729	-20.66598903
ENSG00000238311	snoU13	1.00E-06	1.675655	-20.67629371
ENSG00000281348	CTD-2574D22.6	1.00E-06	1.678426	-20.6786775
ENSG00000249047	COX6B1P5	1.00E-06	1.695342	-20.69314491
ENSG00000252759	Y_RNA	1.00E-06	1.702542	-20.69925896
ENSG00000252840	SNORA44	1.00E-06	1.703795	-20.70032033
ENSG00000264928	AC118463.1	1.00E-06	1.706878	-20.70292851
ENSG00000252614	RNU6-807P	1.00E-06	1.709602	-20.70522907
ENSG00000252349	SNORA31	1.00E-06	1.712826	-20.70794717
ENSG00000271755	RP1-153G14.4	1.00E-06	1.76519	-20.75139205
ENSG00000254806	SYS1-DBNDD2	1.00E-06	1.78264353	-20.76558681
ENSG00000266482	AC023157.1	1.00E-06	1.814952	-20.79149996
ENSG00000264991	AP001205.1	1.00E-06	1.816604	-20.79281253
ENSG00000273834	MIR612	1.00E-06	1.823755	-20.7984805
ENSG00000252096	SNORA31	1.00E-06	1.844107	-20.81449094
ENSG00000240801	AC132217.4	1.00E-06	1.846855	-20.81663917
ENSG00000207342	Y_RNA	1.00E-06	1.858685	-20.82585086
ENSG00000258964	RP11-618G20.1	1.00E-06	1.864029	-20.82999287
ENSG00000274621	MIR6867	1.00E-06	1.874017	-20.83770261
ENSG00000202400	SNORD82	1.00E-06	1.879481	-20.8419029
ENSG00000267228	RP11-49K24.6	1.00E-06	1.88264564	-20.84433004
ENSG00000275686	AC016656.1	1.00E-06	1.885489	-20.8465073
ENSG00000275982	Y_RNA	1.00E-06	1.888461	-20.84877956
ENSG00000264056	MIR5685	1.00E-06	1.959301	-20.90190762
ENSG00000200997	RNU1-85P	1.00E-06	1.980019	-20.91708284
ENSG00000280871	AL021918.1	1.00E-06	1.986074	-20.92148795
ENSG00000277634	Y_RNA	1.00E-06	2.013517	-20.94128622
ENSG00000201435	RNU4-24P	1.00E-06	2.016866	-20.9436838
ENSG00000251246	RP11-540D14.8	1.00E-06	2.018777	-20.94505012
ENSG00000265866	AC067805.1	1.00E-06	2.060743	-20.97473316

ENSG00000252130	RNU6-1045P	1.00E-06	2.073778	-20.98383003
ENSG00000202014	Y_RNA	1.00E-06	2.122374	-21.01724748
ENSG00000266997	RP11-886H22.1	1.00E-06	2.267609	-21.11274047
ENSG00000238793	SNORD124	1.00E-06	2.427737	-21.21118071
ENSG00000271905	AC137590.1	1.00E-06	2.504647	-21.25617586
ENSG00000206941	SNORD15A	1.00E-06	2.523864	-21.26720274
ENSG00000201724	Y_RNA	1.00E-06	2.564842	-21.29043852
ENSG00000251992	SCARNA17	1.00E-06	2.578242	-21.29795625
ENSG00000252414	RNU6-100P	1.00E-06	2.624335	-21.32352046
ENSG00000270585	RP11-568G11.4	1.00E-06	2.654755	-21.34014729
ENSG00000276169	MIR6871	1.00E-06	2.736094	-21.38368636
ENSG00000206715	RNU6-444P	1.00E-06	2.736094	-21.38368636
ENSG00000273885	snoU2-30	1.00E-06	2.829731	-21.43223348
ENSG00000266802	MIR4419A	1.00E-06	2.867685	-21.45145513
ENSG00000212163	SNORD91A	1.00E-06	2.901540733	-21.46838775
ENSG00000275339	RP3-425C14.6	1.00E-06	3.166582	-21.59449501
ENSG00000272647	GS1-259H13.13	1.00E-06	3.250452	-21.63220892
ENSG00000261832	RP11-435I10.4	1.00E-06	3.252975	-21.6333283
ENSG00000185847	LINC01405	1.00E-06	3.377111318	-21.6873583
ENSG00000273745	MIR6870	1.00E-06	3.396079	-21.69543859
ENSG00000252787	SNORD19	1.00E-06	3.568606	-21.7669292
ENSG00000257184	HOXA10-HOXA9	1.00E-06	3.704961	-21.82102693
ENSG00000278129	ZNF8	1.00E-06	3.711512	-21.8235756
ENSG00000178631	ACTG1P1	1.00E-06	4.24795	-22.01833535
ENSG00000256646	RP11-111K18.1	1.00E-06	4.932635456	-22.23392724
ENSG00000240731	RP5-890O3.9	1.00E-06	5.233849	-22.31944087
ENSG00000272160	RNU4-5P	1.00E-06	5.476196	-22.38474265
ENSG00000282304	RP11-1223D19.3	1.00E-06	5.954334675	-22.50550888
ENSG00000222224	Y_RNA	1.00E-06	5.975349	-22.51059155
ENSG00000211459	MT-RNR1	1.00E-06	18.222021	-24.11917964
ENSG00000210049	MT-TF	1.00E-06	77.605797	-26.20966109
ENSG00000210100	MT-TI	1.00E-06	185.17067	-27.46428036
ENSG00000198868	MTND4LP30	1.00E-06	186.548447	-27.47497511
